# Supplementary material for: MTCH2 modulates CPT1 activity to regulate lipid metabolism of adipocytes
Source: Nat Commun. 2025 Oct 3;16:8831. doi: 10.1038/s41467-025-63880-7 (PMC12494973; doi:10.1038/s41467-025-63880-7)
Supplement: Supplementary file 1 — Supplementary Information [file 41467_2025_63880_MOESM1_ESM.pdf]

MTCH2 modulates CPT1 activity to regulate lipid metabolism of adipocytes

Chunyan Wu[1],Tongtong Wang[1], Adhideb Ghosh[1], Fen Long[1], Anand Kumar Sharma[1], Tina Dahlby[1], Falko Noé[1], Ilenia Severi[2], Georgia Colleluori[2], Saverio Cinti[2], Antonio Giordano[2], Lianggong Ding[1], Radhika Khandelwal[1], Sarantos Kostidis[3], Martin Giera[3], Lucia Balazova[4], Vincent Gardeux[5,6], Laith Abu-Nawwas[5,6], Bart Deplancke[5,6], Sabita Chourasia[7], Sandra Kleiner[8], Bradford S Hamilton[8], Juan Manuel Alcántara Alcántara[9,10,11], Jonatan R Ruiz[9,10,11], Matthias Blüher[12,13], Anton Pekcec[8], Miroslav Balaz[4,14], Atan Gross[7], Heike Neubauer[8] & Christian Wolfrum[1,15,*]

*Correspondence: christian.wolfrum@ntu.edu.sg

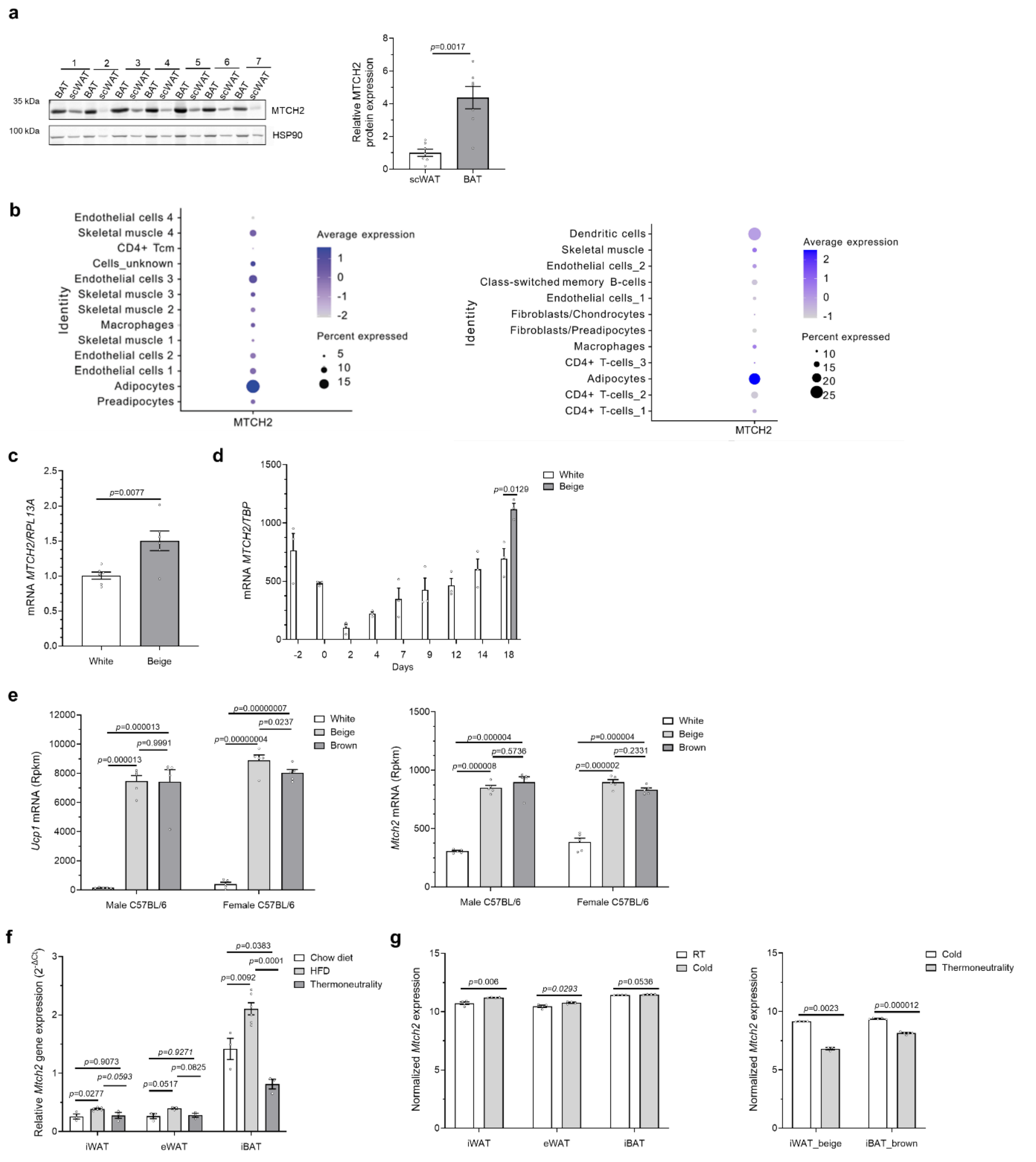

Supplementary Figure 1. MTCH2 expression levels in human and murine adipocytes. a MTCH2 protein expression levels in human subcutaneous white adipose tissue (scWAT) and supraclavicular brown adipose tissue (BAT) from seven different donors. n=7. **b** *MTCH2* expression levels in human scWAT (left panel) and supraclavicular BAT (right panel) population as revealed by snRNA-seq analyzed from previous publications[10,47]. **c** *MTCH2* expression levels in hMADS differentiated white (in white) and beige (in grey) adipocytes. n=6 for each group. **d** *MTCH2* expression levels during hMADS differentiation. beige adipocytes in grey. n=3 for each group. **e** *Ucp1* and *Mtch2* expression levels in sorted white adipocytes (in white), beige adipocytes (in grey), brown adipocytes (in dark grey). n=5 for each group. **f** *Mtch2* expression on HFD (in grey) and thermoneutrality (in dark grey) in different murine adipose tissue depots. **g** *Mtch2* expression during cold exposure (in grey) and thermoneutrality (in dark grey) in different murine adipose tissue depots._n=4 for each group for iWAT, eWAT, iBAT under RT and cold. For iWAT_beige,_n=4 under cold and n=3 under thermoneutrality. n=5 for iBAT_brown. Data are presented as mean ± SEM and analyzed using two-tailed Student's *t*-test (**a**,**c**,**d**,**g**), and two-way ANOVA with Tukey's post hoc multiple comparison test (**e**,**f**). Source data are provided as a Source Data file.

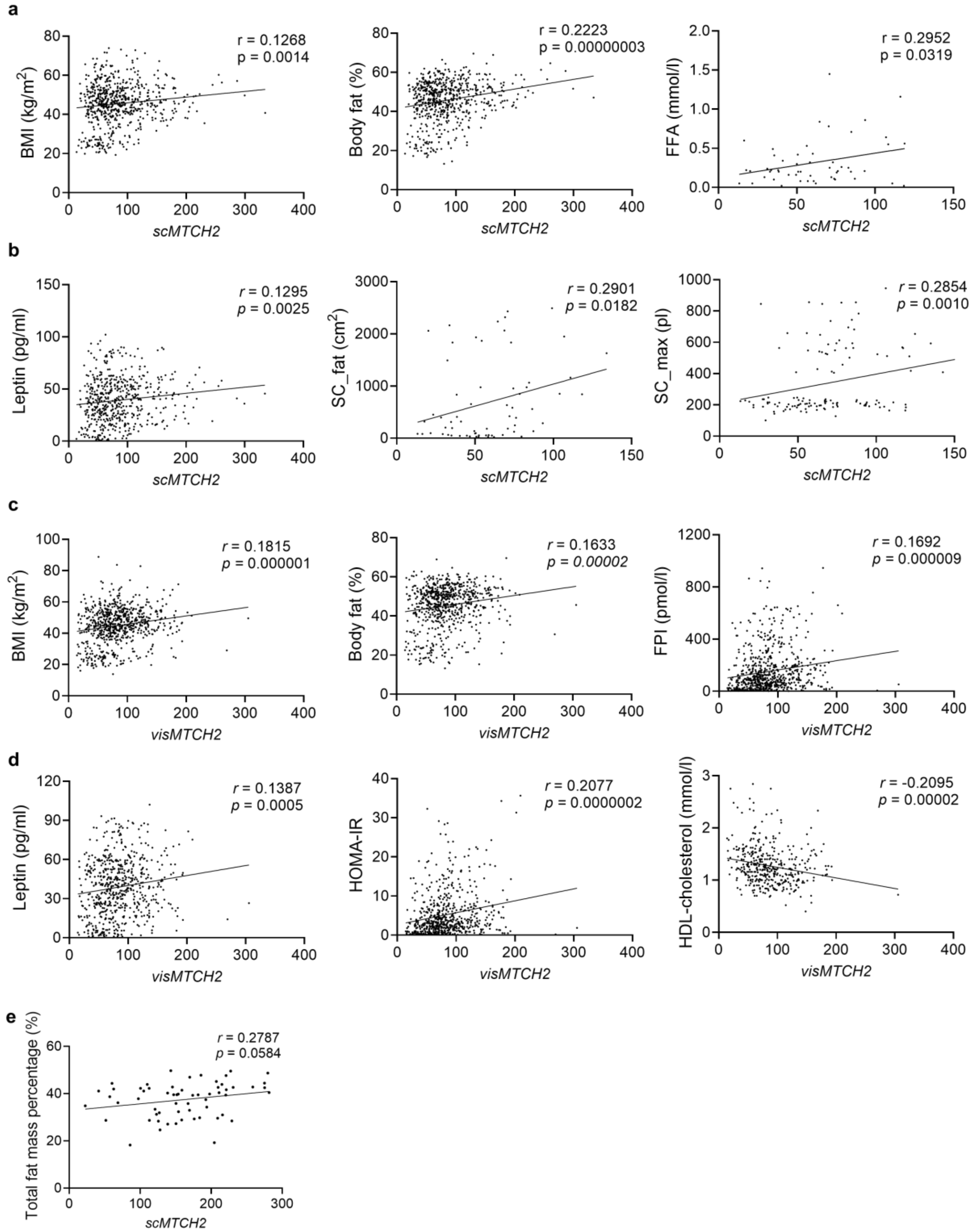

Supplementary Figure 2. ***MTCH2*** **expression in humans is linked to a metabolic profile associated with obesity. a-d** LOBB CSC study. Correlation of *MTCH2* expression in human subcutaneous white adipose tissue (scWAT; *scMTCH2*) with (**a**) body mass index (BMI) (n=635 individuals), body fat (n=613 individuals), free fatty acids (FFA) (n=53 individuals), (**b**) leptin (n=545 individuals), SC_fat (n=66 individuals) and SC_max (n=130 individuals). Correlation of *MTCH2* expression in human visceral WAT (visWAT, *visMTCH2*) with (**c**) BMI (n=721 individuals), body fat (n=691 individuals), fasting plasma insulin (FPI) (n=683 individuals), (**d**) leptin (n=625 individuals), homeostatic model assessment for insulin resistance (HOMA-IR) (n=615 individuals) and HDL-cholesterol (n=404 individuals). **e** Correlation of sc*MTCH2* expression in human with total fat mass percentage (n=60 individuals) in ACTIBATE study. Data are presented by a two-tailed Pearson test (**a-e**). Source data are provided as a Source Data file.

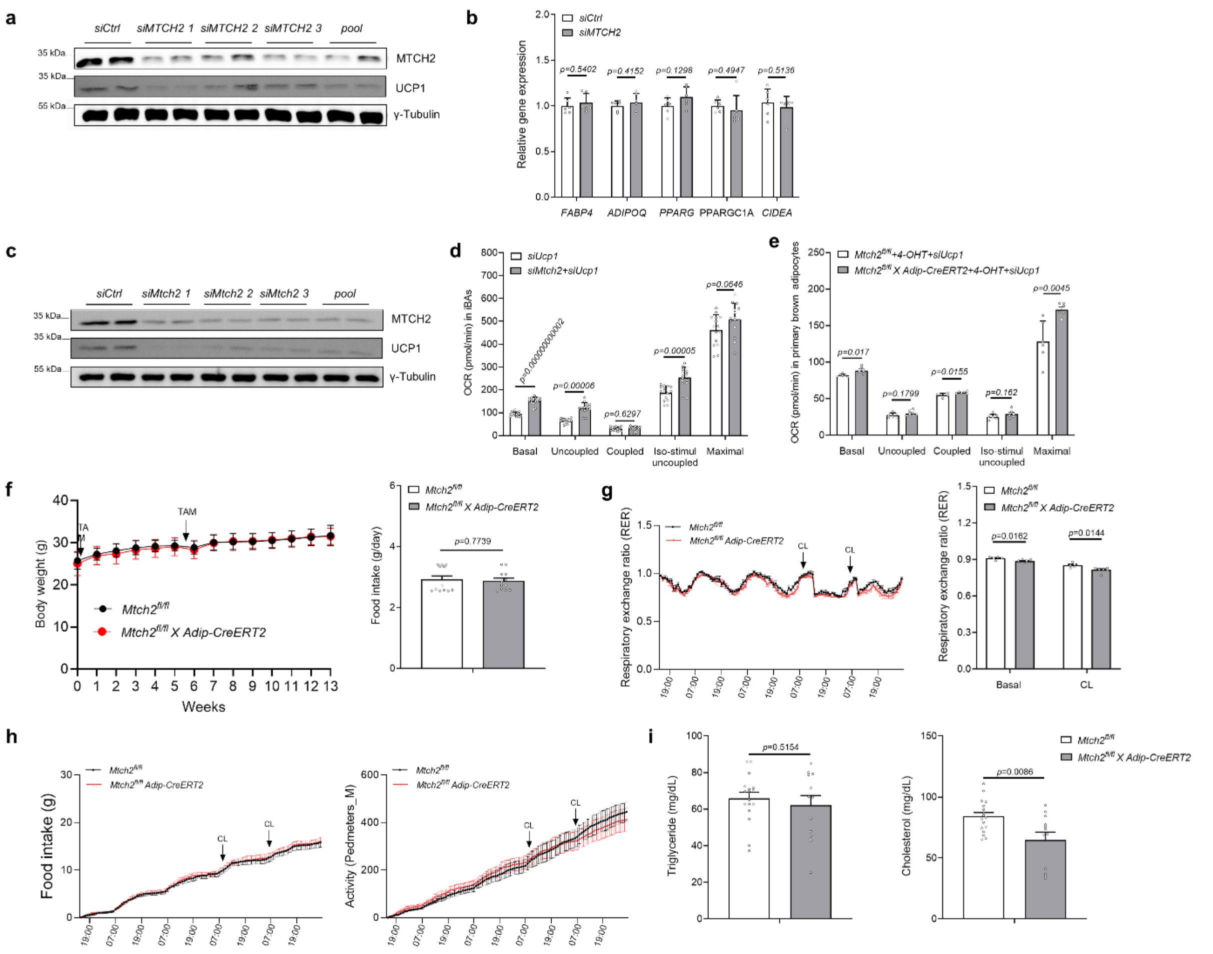

Supplementary Figure 3. The effects of *MTCH2* ablation in human and mouse adipocytes. a MTCH2 and UCP1 protein levels in hMADS adipocytes with different *MTCH2* siRNA mediated knockdown (KD). n=2 for each group. Experiments were repeated two times. **b** The effects of *MTCH2* KD on the expression of adipogenic and thermogenic marker genes in hMADS adipocytes. Control group (siCtrl) in white and *MTCH2* KD group (siMTCH2) in grey. n=6 for each group. **c** MTCH2 and UCP1 protein levels in iBAs with different *Mtch2* siRNA mediated KD. n=2 for each group. Experiments were repeated two times. **d** The effects of *Mtch2* on cellular respiration in iBAs with *Mtch2* and *Ucp1* KD. Control group (siUcp1) in white and Ucp1/Mtch2 double KD group (siMtch2+siUcp1) in grey. n=15 for each group. **e** The effects of *Mtch2* on cellular respiration in primary brown adipocytes with *Mtch2* and *Ucp1* KD. *Mtch2* KD was mediated by 4-hydroxytamoxifen (4-OHT). Control group (siUcp1) in white and Ucp1/Mtch2 double KD group (siMtch2+siUcp1) in grey. n=6 for each group. **f** Mice body weight (control group mice in black line and *Mtch2* KO mice in red line) and food intake (control group mice in white and *Mtch2* KO mice in grey) fed on a chow diet. n=12 for each group. **g** Respiratory exchange ratio (RER). Control group mice n=5 in black line and white bar, *Mtch2* KO mice n=6 in red line and grey bar. **h** Food intake and activity of mice checked by metabolic cages. n=5 for control group mice in black lines and n=6 for *Mtch2* KO mice in red lines. **i** Plasma triglyceride and cholesterol levels in adipose tissue specific *Mtch2* KO mice (*Mtch2$^{fl/fl}$Adip-CreERT2*) after 13 weeks on an HFD. n=17 for control group mice in white and n=13 for *Mtch2* KO mice in grey. Data are presented as mean ± SEM and analyzed using two-tailed Student's *t*-test. Source data are provided as a Source Data file.

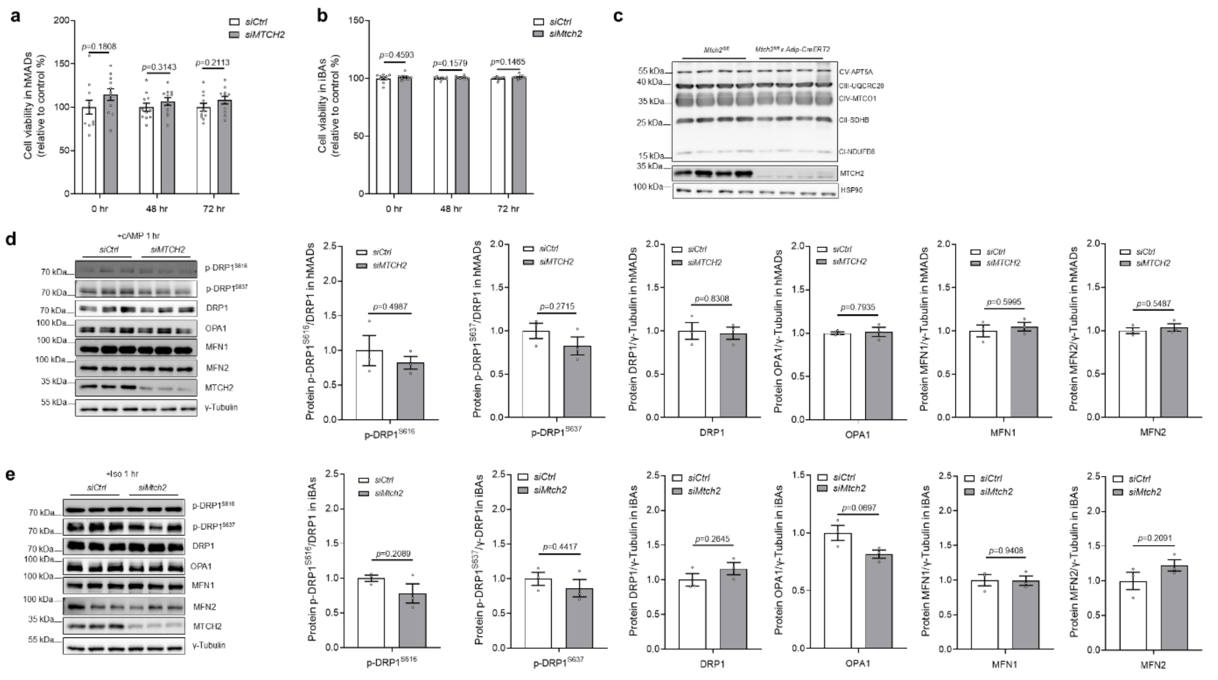

Supplementary Figure 4. MTCH2 regulates mitochondrial function independent of cell apoptosis, OXPHOS complexes levels or mitochondrial dynamics in human and mouse adipocytes. a The cell viability in hMADS with *MTCH2* knockdown (KD). Control group (siCtrl) in white and *MTCH2* KD group (siMTCH2) in grey. n=11 for each group. **b** The cell viability in iBAs with *Mtch2* KD. Control group (siCtrl) in white and *Mtch2* KD group (siMtch2) in grey. n=10 for each group. **c** The effects of *Mtch2* silencing on cellular levels of individual OXPHOS proteins in iBAT from *MTCH2*$^{fl/fl}$*Adip-CreERT2* and control littermates. n=4 for each group. Experiments were repeated two times. **d** The effects of *MTCH2* KD on the expression levels of core indicated mitochondrial dynamics proteins in cAMP-stimulated hMADS. Control group (siCtrl) in white and *MTCH2* KD group (siMTCH2) in grey. n=3 for each group. Experiments were repeated two times. **e** The effects of *Mtch2* KD on the expression levels of core indicated mitochondrial dynamics proteins in isoproterenol-stimulated iBAs. Control group (siCtrl) in white and *Mtch2* KD group (siMtch2) in grey. n=3 for each group. Experiments were repeated two times. Data are presented as mean ± SEM and analyzed using two-tailed Student's *t*-test. Source data are provided as a Source Data file.

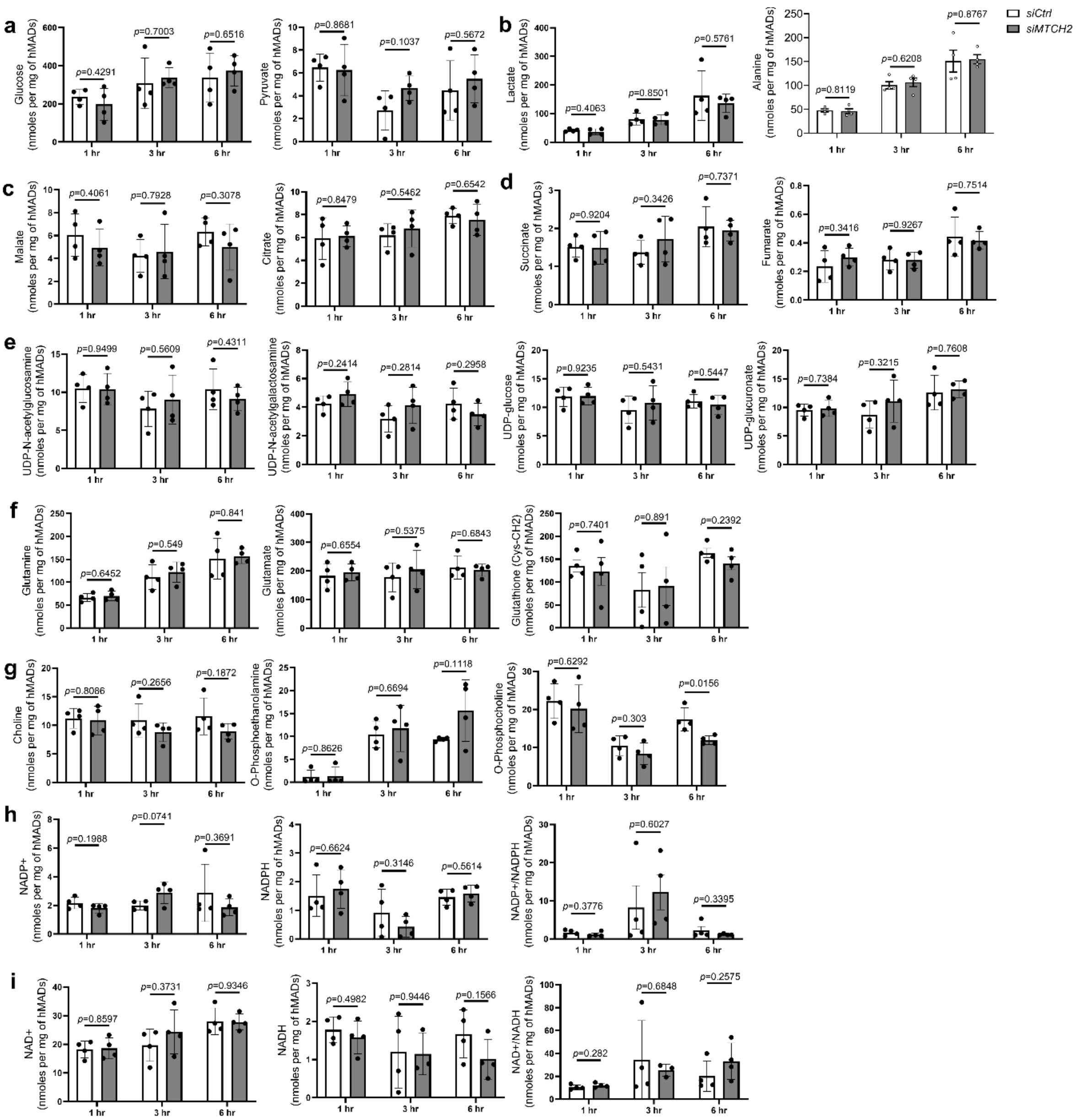

Supplementary Figure 5. The effects of *MTCH2* knockdown (KD) on major metabolites in human adipocytes. a Glucose and pyruvate levels in hMADS adipocytes with *MTCH2* KD. Control group (siCtrl) in white and *MTCH2* KD group (siMTCH2) in grey. n=4 for each group. **b** Lactate and alanine levels. Control group (siCtrl) in white and *MTCH2* KD group (siMTCH2) in grey. n=4 for each group. **c** Malate and citrate levels. Control group (siCtrl) in white and *MTCH2* KD group (siMTCH2) in grey. n=4 for each group. **d** Succinate and fumarate levels. Control group (siCtrl) in white and *MTCH2* KD group (siMTCH2) in grey. n=4 for each group. **e** The levels of UDP-N-acetylglucosamine, UDP-N-acetylgalactosamine, UDP-glucose, and UDP-glucuronate. Control group (siCtrl) in white and *MTCH2* KD group (siMTCH2) in grey. n=4 for each group. **f** Glutamate, glutamine, and glutathione levels. Control group (siCtrl) in white and *MTCH2* KD group (siMTCH2) in grey. n=4 for each group. **g** Choline, phosphoethanolamine, and phosphocholine levels. Control group (siCtrl) in white and *MTCH2* KD group (siMTCH2) in grey. n=4 for each group. **h** $NADP^+$, NADPH levels, and $NADP^+$/NADPH ratios. Control group (siCtrl) in white and *MTCH2* KD group (siMTCH2) in grey. n=4 for each group. **I** NAD^+, NADH levels, and NAD^+/NADH ratios. Control group (siCtrl) in white and *MTCH2* KD group (siMTCH2) in grey. n=3 for NADH and NAD^+/NADH levels in *MTCH2* knockdown group at 3 hr and n=4 for other groups. Data are presented as mean ± SEM and analyzed using two-tailed Student's *t*-test. Source data are provided as a Source Data file.

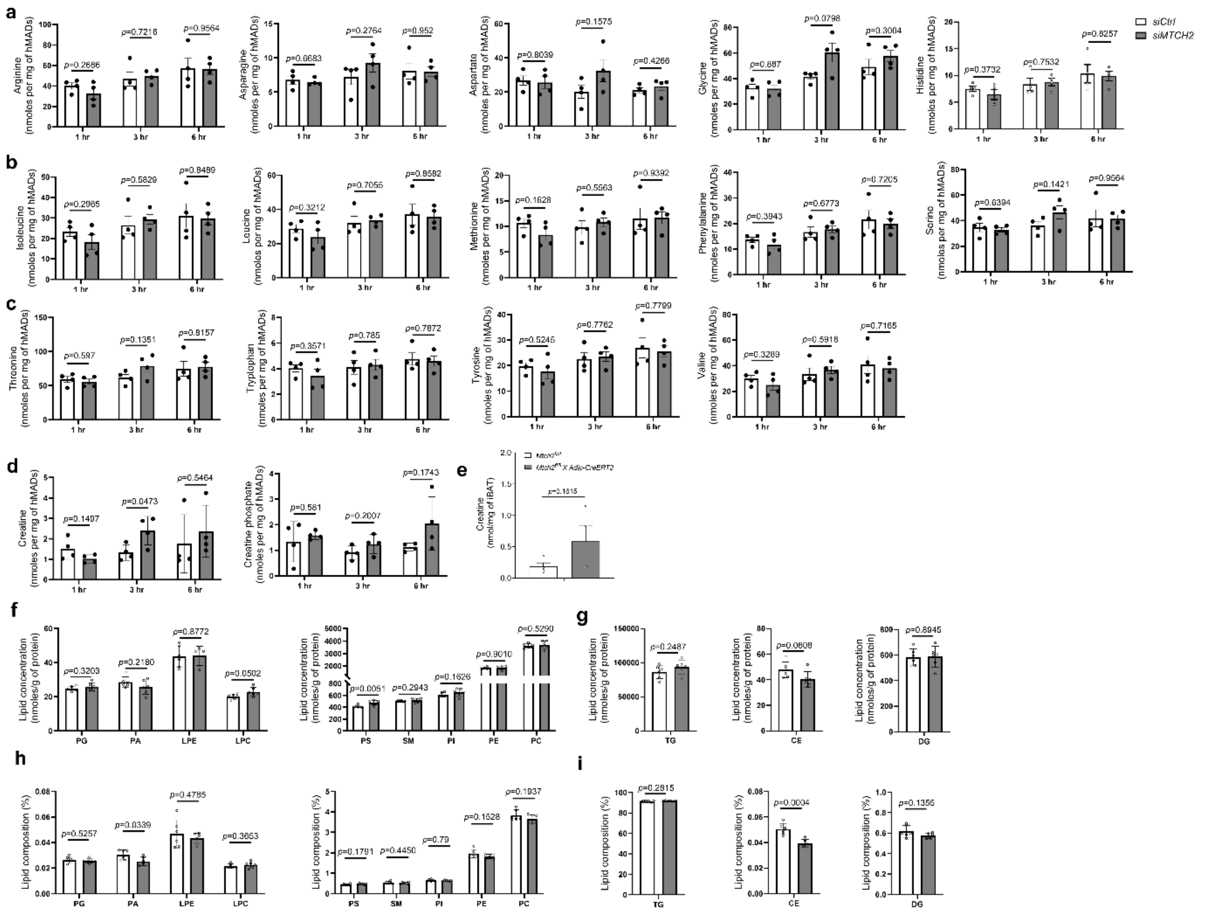

Supplementary Figure 6. The effects of MTCH2 deficiency on amino acids, creatine, membrane lipids and storage lipids levels. a-c Amino acids levels in hMADS adipocytes with *MTCH2* knockdown (KD) Control group (siCtrl) in white and *MTCH2* KD group (siMTCH2) in grey. n=4 for each group. **d** Creatine and creatine phosphate levels in hMADS adipocytes with *MTCH2* KD. Control group (siCtrl) in white and *MTCH2* KD group (siMTCH2) in grey. n=4 for each group. **e** The effects of MTCH2 deficiency on creatine in iBAT from *Mtch2*$^{fl/fl}$*Adip-CreERT2* and control littermates. Control mice group in white and *Mtch2* knockout group in grey. n=4 for each group. **f**,**g** The effects of MTCH2 deficiency on absolute (f) membrane lipids and (**g**) storage lipids levels in hMADS adipocytes with *MTCH2* KD. Control group (siCtrl) in white and *MTCH2* KD group (siMTCH2) in grey. n=6 for each group. **h**,**i** The effects of MTCH2 deficiency on (h) membrane lipids and (**i**) storage lipid composition levels in hMADS adipocytes with *MTCH2* KD. Control group (siCtrl) in white and *MTCH2* KD group (siMTCH2) in grey. n=6 for each group. Data are presented as mean ± SEM and analyzed using two-tailed Student's *t*-test. Source data are provided as a Source Data file.

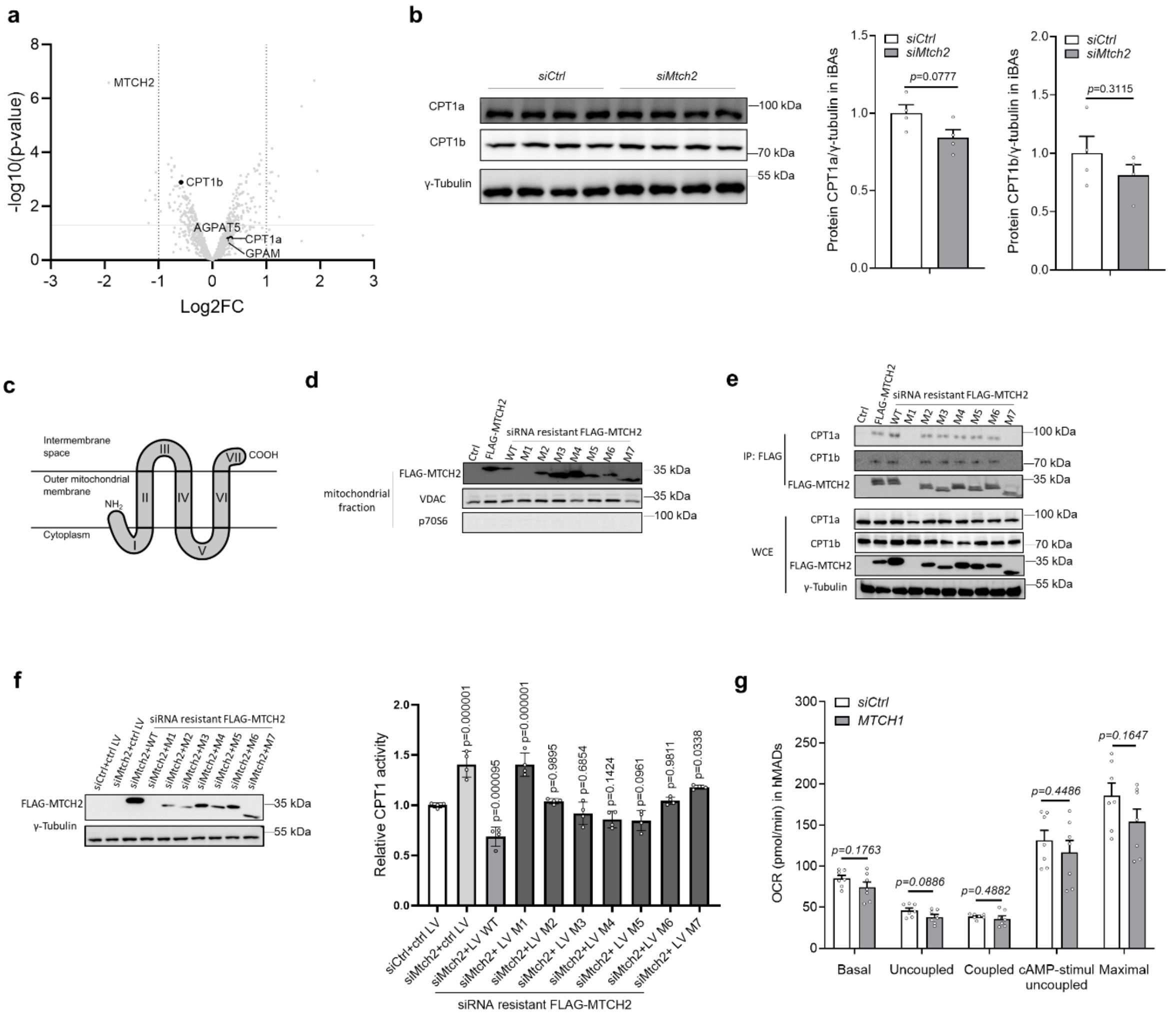

Supplementary Figure 7. The C-terminus of MTCH2 is essential for both its interaction with CPT1 and the regulation of CPT1 activity. a AGPAT5, CPT1a, CPT1b, and GPAM levels in mitochondria proteomics in iBAs. n=4 for each group. **b** CPT1a and CPT1b protein levels in iBAs with *Mtch2* knockdown (KD) analyzed by immunoblot. Control group (siCtrl) in white and *Mtch2* KD group (siMtch2) in grey. n=4 for each group. Experiments were repeated three times. **c** Schematic representation of seven truncation mutants (M1–M7) generated based on the published structure of MTCH2, using the siRNA-resistant FLAG-tagged wild-type (WT) construct as the backbone. **d** Western blot analysis of mitochondrial fractions isolated from HEK293T cells transfected with either WT or truncation mutants. Voltage-dependent anion channel (VDAC) was used as a marker for the outer mitochondrial membrane, and p70S6 kinase (p70S6) served as a cytosolic protein marker. Experiments were repeated two times. **e** Co-immunoprecipitation performed on HEK293T whole-cell extracts (WCE) using anti-FLAG antibody, following transfection with either WT or truncation mutants. Experiments were repeated three times. **f** CPT1 activity measured in mitochondria isolated from iBAs infected with lentiviral WT or truncation mutants, followed by siRNA-mediated KD of endogenous *Mtch2*. Activity was assessed in the presence of 20 µM malonyl-CoA._n=4 for each group. Experiments were repeated three times. **g** Effects of MTCH1 on mitochondrial respirations in hMADS adipocytes. Control group (siCtrl) in white and *MTCH1* KD group (siMTHC1) in grey._n=7 for each group. Data are presented as mean ± SEM and analyzed using two-tailed Student's *t*-test (**b,g**), and one -way ANOVA (**f**). Source data are provided as a Source Data file.

Supplementary Table 1. List of genes differentially expressed between supraclavicular BAT and subcutaneous WAT identified using two-tailed DESeq2 analysis (n=6; FDR<0.05).

number	ID	Gene symbol	log_2FoldChange	P value	FDR adjusted P
1	ENSG00000171303.6	KCNK3	5.08	3.41E-104	9.42E-100
2	ENSG00000144810.15	COL8A1	-2.51	4.64E-67	6.41E-63
3	ENSG00000198712.1	MT-CO2	2.31	4.51E-65	4.15E-61
4	ENSG00000198814.12	GK	3.53	3.82E-60	2.64E-56
5	ENSG00000138029.13	HADHB	1.96	3.60E-56	1.99E-52
6	ENSG00000198804.2	MT-CO1	2.25	6.90E-54	3.17E-50
7	ENSG00000205560.12	CPT1B	2.85	2.56E-53	1.01E-49
8	ENSG00000178772.6	CPN2	3.05	4.09E-52	1.41E-48
9	ENSG00000168781.22	PPIP5K1	1.89	5.22E-52	1.60E-48
10	ENSG00000160216.18	AGPAT3	1.42	8.84E-50	2.44E-46
11	ENSG00000121769.7	FABP3	3.94	3.26E-49	8.19E-46
12	ENSG00000109424.3	UCP1	8.73	2.03E-48	4.50E-45
13	ENSG00000105953.14	OGDH	1.68	2.12E-48	4.50E-45
14	ENSG00000048540.14	LMO3	2.26	2.70E-48	5.32E-45
15	ENSG00000237973.1	MTCO1P12	2.24	2.33E-47	4.29E-44
16	ENSG00000139182.14	CLSTN3	3.14	1.68E-46	2.90E-43
17	ENSG00000248527.1	MTATP6P1	1.85	1.38E-45	2.24E-42
18	ENSG00000138395.14	CDK15	3.32	3.83E-44	5.87E-41
19	ENSG00000198886.2	MT-ND4	1.79	3.04E-43	4.41E-40
20	ENSG00000084710.13	EFR3B	2.89	4.03E-43	5.57E-40
21	ENSG00000170549.3	IRX1	-4.07	6.24E-43	8.21E-40
22	ENSG00000152207.7	CYSLTR2	3.88	1.08E-42	1.36E-39
23	ENSG00000198786.2	MT-ND5	1.74	5.46E-42	6.55E-39
24	ENSG00000137642.12	SORL1	1.95	2.65E-40	2.93E-37
25	ENSG00000167315.17	ACAA2	1.86	2.61E-40	2.93E-37
26	ENSG00000198763.3	MT-ND2	1.62	1.06E-39	1.13E-36
27	ENSG00000010256.10	UQCRC1	2.09	1.25E-39	1.28E-36
28	ENSG00000008735.13	MAPK8IP2	3.77	1.39E-39	1.38E-36
29	ENSG00000072778.19	ACADVL	1.49	6.54E-39	6.22E-36
30	ENSG00000181035.13	SLC25A42	2.11	2.61E-38	2.40E-35
31	ENSG00000110955.8	ATP5F1B	2.26	2.57E-37	2.29E-34
32	ENSG00000198899.2	MT-ATP6	1.90	2.97E-37	2.56E-34
33	ENSG00000100412.15	ACO2	2.39	4.11E-37	3.44E-34
34	ENSG00000198888.2	MT-ND1	1.75	4.72E-37	3.84E-34
35	ENSG00000178741.11	COX5A	2.15	9.03E-36	7.13E-33
36	ENSG00000068976.13	PYGM	2.81	3.61E-33	2.77E-30
37	ENSG00000178537.9	SLC25A20	2.06	5.65E-33	4.21E-30
38	ENSG00000225630.1	MTND2P28	1.83	7.72E-33	5.61E-30
39	ENSG00000198727.2	MT-CYB	1.74	2.18E-32	1.54E-29
40	ENSG00000084754.11	HADHA	1.54	2.95E-32	2.04E-29
41	ENSG00000172831.11	CES2	2.17	3.42E-32	2.30E-29
42	ENSG00000117054.13	ACADM	2.25	3.52E-32	2.31E-29
43	ENSG00000136143.15	SUCLA2	1.78	9.48E-32	6.09E-29
44	ENSG00000232821.1	AC003986.2	-2.76	1.98E-31	1.24E-28
45	ENSG00000221818.8	EBF2	1.79	6.12E-31	3.76E-28
46	ENSG00000109107.13	ALDOC	-3.09	9.75E-31	5.85E-28
47	ENSG00000198938.2	MT-CO3	2.38	1.47E-30	8.66E-28

48	ENSG00000023228.13	NDUFS1	1.81	3.70E-30	2.13E-27
49	ENSG00000212907.2	MT-ND4L	1.82	3.96E-30	2.23E-27
50	ENSG00000220586.2	TUBBP9	11.04	5.83E-30	3.22E-27
51	ENSG00000186335.8	SLC36A2	4.04	6.39E-30	3.46E-27
52	ENSG00000140740.10	UQCRC2	1.56	7.71E-30	4.09E-27
53	ENSG00000135903.19	PAX3	-3.30	2.29E-29	1.20E-26
54	ENSG00000163344.5	PMVK	1.44	5.38E-29	2.75E-26
55	ENSG00000167107.12	ACSF2	1.74	2.85E-28	1.43E-25
56	ENSG00000143171.12	RXRG	3.38	3.09E-28	1.48E-25
57	ENSG00000198910.13	L1CAM	2.03	3.11E-28	1.48E-25
58	ENSG00000186493.12	C5orf38	-3.26	3.02E-28	1.48E-25
59	ENSG00000073578.16	SDHA	1.77	3.78E-28	1.77E-25
60	ENSG00000130540.13	SULT4A1	5.19	5.12E-28	2.36E-25
61	ENSG00000084674.14	APOB	-5.82	5.23E-28	2.37E-25
62	ENSG00000197444.9	OGDHL	8.19	9.24E-28	4.12E-25
63	ENSG00000117118.9	SDHB	2.16	1.03E-27	4.53E-25
64	ENSG00000166411.13	IDH3A	1.66	1.05E-27	4.54E-25
65	ENSG00000152234.15	ATP5F1A	1.64	1.99E-27	8.46E-25
66	ENSG00000126602.10	TRAP1	1.47	3.31E-27	1.39E-24
67	ENSG00000164309.14	CMYA5	1.82	3.96E-27	1.63E-24
68	ENSG00000119673.14	ACOT2	1.56	4.76E-27	1.93E-24
69	ENSG00000179091.4	CYC1	1.77	5.92E-27	2.37E-24
70	ENSG00000198840.2	MT-ND3	1.50	6.89E-27	2.72E-24
71	ENSG00000055163.19	CYFIP2	3.06	8.28E-27	3.22E-24
72	ENSG00000131143.8	COX4I1	1.69	1.40E-26	5.37E-24
73	ENSG00000113790.10	EHHADH	1.49	1.49E-26	5.62E-24
74	ENSG00000085998.13	POMGNT1	1.42	1.75E-26	6.55E-24
75	ENSG00000168710.17	AHCYL1	1.30	1.91E-26	7.02E-24
76	ENSG00000122971.8	ACADS	1.28	2.02E-26	7.33E-24
77	ENSG00000198695.2	MT-ND6	1.75	2.90E-26	1.04E-23
78	ENSG00000112294.12	ALDH5A1	2.38	2.98E-26	1.06E-23
79	ENSG00000115255.10	REEP6	3.45	3.73E-26	1.30E-23
80	ENSG00000175497.16	DPP10	2.18	4.32E-26	1.49E-23
81	ENSG00000165678.20	GHITM	1.60	4.69E-26	1.60E-23
82	ENSG00000155011.8	DKK2	-2.54	6.35E-26	2.14E-23
83	ENSG00000113448.18	PDE4D	2.27	7.97E-26	2.65E-23
84	ENSG00000167792.12	NDUFV1	1.49	1.17E-25	3.84E-23
85	ENSG00000152926.14	ZNF117	1.22	1.43E-25	4.65E-23
86	ENSG00000179915.22	NRXN1	2.04	1.59E-25	5.11E-23
87	ENSG00000120053.11	GOT1	2.06	1.82E-25	5.79E-23
88	ENSG00000102763.17	VWA8	1.14	2.23E-25	6.99E-23
89	ENSG00000120075.5	HOXB5	-2.49	2.47E-25	7.67E-23
90	ENSG00000169021.5	UQCRFS1	1.87	3.60E-25	1.10E-22
91	ENSG00000171503.11	ETFDH	1.61	4.49E-25	1.36E-22
92	ENSG00000147684.8	NDUFB9	1.60	6.76E-25	2.03E-22
93	ENSG00000110717.12	NDUFS8	1.57	1.59E-24	4.73E-22
94	ENSG00000091140.13	DLD	1.42	3.14E-24	9.21E-22
95	ENSG00000152782.16	PANK1	2.32	3.25E-24	9.45E-22
96	ENSG00000150594.6	ADRA2A	-2.36	3.58E-24	1.03E-21
97	ENSG00000158864.12	NDUFS2	1.51	8.57E-24	2.44E-21

98	ENSG00000135744.7	AGT	3.19	9.30E-24	2.60E-21
99	ENSG00000188488.13	SERPINA5	-2.51	9.31E-24	2.60E-21
100	ENSG00000139304.13	PTPRQ	-2.11	1.02E-23	2.82E-21
101	ENSG00000198626.15	RYR2	2.60	1.25E-23	3.42E-21
102	ENSG00000169851.15	PCDH7	-2.33	2.17E-23	5.87E-21
103	ENSG00000260027.4	HOXB7	-2.69	9.43E-23	2.53E-20
104	ENSG00000112541.14	PDE10A	2.33	1.35E-22	3.58E-20
105	ENSG00000123472.12	ATPAF1	1.02	2.20E-22	5.80E-20
106	ENSG00000131828.13	PDHA1	1.94	6.14E-22	1.60E-19
107	ENSG00000255794.7	RMST	5.50	7.59E-22	1.96E-19
108	ENSG00000171314.8	PGAM1	1.09	1.10E-21	2.81E-19
109	ENSG00000184313.19	MROH7	2.61	1.34E-21	3.40E-19
110	ENSG00000132313.14	MRPL35	1.16	3.30E-21	8.29E-19
111	ENSG00000004455.16	AK2	0.98	3.53E-21	8.77E-19
112	ENSG00000119689.14	DLST	1.14	3.95E-21	9.75E-19
113	ENSG00000245694.9	CRNDE	-2.49	4.39E-21	1.07E-18
114	ENSG00000005022.5	SLC25A5	1.67	4.97E-21	1.19E-18
115	ENSG00000164050.12	PLXNB1	-1.35	4.97E-21	1.19E-18
116	ENSG00000106992.18	AK1	1.76	5.50E-21	1.31E-18
117	ENSG00000229344.1	MTCO2P12	2.25	5.98E-21	1.41E-18
118	ENSG00000072274.12	TFRC	1.77	6.57E-21	1.54E-18
119	ENSG00000172115.8	CYCS	1.74	8.02E-21	1.86E-18
120	ENSG00000156709.13	AIFM1	1.50	9.87E-21	2.27E-18
121	ENSG00000075239.13	ACAT1	1.26	1.16E-20	2.65E-18
122	ENSG00000185010.14	F8	0.96	2.02E-20	4.57E-18
123	ENSG00000119421.6	NDUFA8	1.41	2.85E-20	6.41E-18
124	ENSG00000132305.20	IMMT	1.30	3.69E-20	8.22E-18
125	ENSG00000166224.16	SGPL1	1.05	3.83E-20	8.45E-18
126	ENSG00000052723.11	SIKE1	0.98	6.00E-20	1.31E-17
127	ENSG00000255639.3	AC005833.1	2.16	8.28E-20	1.80E-17
128	ENSG00000225972.1	MTND1P23	1.96	9.43E-20	2.03E-17
129	ENSG00000111775.2	COX6A1	1.55	9.78E-20	2.09E-17
130	ENSG00000182902.13	SLC25A18	2.07	1.21E-19	2.57E-17
131	ENSG00000189043.9	NDUFA4	1.52	1.36E-19	2.86E-17
132	ENSG00000091986.15	CCDC80	-1.28	1.76E-19	3.68E-17
133	ENSG00000151729.10	SLC25A4	1.29	1.81E-19	3.76E-17
134	ENSG00000114054.13	PCCB	1.30	1.94E-19	3.99E-17
135	ENSG00000250479.8	CHCHD10	2.21	2.25E-19	4.59E-17
136	ENSG00000144381.16	HSPD1	1.35	2.34E-19	4.76E-17
137	ENSG00000007202.14	KIAA0100	0.64	2.46E-19	4.95E-17
138	ENSG00000177614.10	PGBD5	2.51	2.93E-19	5.86E-17
139	ENSG00000144278.14	GALNT13	-2.68	3.13E-19	6.22E-17
140	ENSG00000135940.6	COX5B	1.80	3.40E-19	6.72E-17
141	ENSG00000161267.11	BDH1	2.18	3.79E-19	7.42E-17
142	ENSG00000132361.16	CLUH	1.10	4.02E-19	7.81E-17
143	ENSG00000125148.6	MT2A	2.52	5.18E-19	9.96E-17
144	ENSG00000204370.10	SDHD	1.42	5.19E-19	9.96E-17
145	ENSG00000108528.13	SLC25A11	1.19	6.24E-19	1.19E-16
146	ENSG00000112695.11	COX7A2	1.67	7.18E-19	1.35E-16
147	ENSG00000115159.15	GPD2	1.66	7.20E-19	1.35E-16

148	ENSG00000184752.12	NDUFA12	1.11	8.25E-19	1.54E-16
149	ENSG00000113013.13	HSPA9	1.29	8.42E-19	1.56E-16
150	ENSG00000162390.17	ACOT11	1.61	8.51E-19	1.57E-16
151	ENSG00000135077.8	HAVCR2	2.39	1.65E-18	3.02E-16
152	ENSG00000101019.21	UQCC1	1.12	1.91E-18	3.47E-16
153	ENSG00000115226.9	FNDC4	2.05	1.94E-18	3.51E-16
154	ENSG00000131368.7	MRPS25	0.75	2.24E-18	4.02E-16
155	ENSG00000235026.5	DPP10-AS1	8.07	2.59E-18	4.61E-16
156	ENSG00000072062.13	PRKACA	0.93	2.70E-18	4.77E-16
157	ENSG00000155846.16	PPARGC1B	1.74	4.83E-18	8.50E-16
158	ENSG00000154305.16	MIA3	0.87	5.82E-18	1.02E-15
159	ENSG00000237289.9	CKMT1B	6.77	7.29E-18	1.27E-15
160	ENSG00000088682.13	COQ9	1.63	8.14E-18	1.41E-15
161	ENSG00000241837.6	ATP5PO	1.27	9.14E-18	1.57E-15
162	ENSG00000109519.12	GRPEL1	1.42	9.78E-18	1.67E-15
163	ENSG00000155966.13	AFF2	-2.75	1.00E-17	1.70E-15
164	ENSG00000111799.20	COL12A1	-1.84	1.03E-17	1.73E-15
165	ENSG00000106004.4	HOXA5	2.08	1.20E-17	2.00E-15
166	ENSG00000139155.8	SLCO1C1	1.63	1.26E-17	2.10E-15
167	ENSG00000127540.11	UQCR11	1.37	1.28E-17	2.12E-15
168	ENSG00000005249.12	PRKAR2B	0.99	1.34E-17	2.20E-15
169	ENSG00000108179.13	PPIF	1.72	1.42E-17	2.33E-15
170	ENSG00000213593.9	TMX2	1.23	1.44E-17	2.34E-15
171	ENSG00000127884.4	ECHS1	1.73	1.78E-17	2.88E-15
172	ENSG00000104325.6	DECR1	1.76	1.93E-17	3.10E-15
173	ENSG00000242110.7	AMACR	1.20	1.96E-17	3.13E-15
174	ENSG00000101049.15	SGK2	1.76	2.05E-17	3.25E-15
175	ENSG00000168827.14	GFM1	0.89	2.39E-17	3.75E-15
176	ENSG00000084636.17	COL16A1	-1.44	2.39E-17	3.75E-15
177	ENSG00000148090.11	AUH	1.41	2.91E-17	4.54E-15
178	ENSG00000176894.9	PXMP2	2.33	3.04E-17	4.72E-15
179	ENSG00000067225.17	PKM	1.54	3.20E-17	4.93E-15
180	ENSG00000101542.9	CDH20	-2.52	3.54E-17	5.43E-15
181	ENSG00000197142.10	ACSL5	1.67	4.10E-17	6.26E-15
182	ENSG00000143252.14	SDHC	1.08	4.59E-17	6.96E-15
183	ENSG00000146477.5	SLC22A3	1.35	4.88E-17	7.36E-15
184	ENSG00000114023.15	FAM162A	1.76	5.34E-17	8.01E-15
185	ENSG00000198744.5	MTCO3P12	2.51	6.11E-17	9.12E-15
186	ENSG00000080493.16	SLC4A4	2.03	6.44E-17	9.55E-15
187	ENSG00000139180.10	NDUFA9	1.08	7.08E-17	1.05E-14
188	ENSG00000131730.15	CKMT2	3.11	7.30E-17	1.07E-14
189	ENSG00000050426.15	LETMD1	1.12	7.37E-17	1.08E-14
190	ENSG00000232177.1	MTND4P24	2.35	7.90E-17	1.15E-14
191	ENSG00000122691.12	TWIST1	-2.01	8.17E-17	1.18E-14
192	ENSG00000101846.6	STS	1.35	1.16E-16	1.67E-14
193	ENSG00000117984.13	CTSD	1.01	1.17E-16	1.68E-14
194	ENSG00000166220.12	TBATA	7.12	1.25E-16	1.77E-14
195	ENSG00000136960.12	ENPP2	-1.34	1.32E-16	1.87E-14
196	ENSG00000173660.11	UQCRH	1.41	1.46E-16	2.05E-14
197	ENSG00000138095.18	LRPPRC	1.12	1.67E-16	2.34E-14

198	ENSG00000228253.1	MT-ATP8	1.92	1.76E-16	2.46E-14
199	ENSG00000172270.19	BSG	1.24	2.58E-16	3.57E-14
200	ENSG00000248383.4	PCDHAC1	-2.57	2.69E-16	3.71E-14
201	ENSG00000066855.15	MTFR1	1.06	3.00E-16	4.13E-14
202	ENSG00000131174.5	COX7B	1.42	3.30E-16	4.51E-14
203	ENSG00000178814.16	OPLAH	1.66	4.29E-16	5.83E-14
204	ENSG00000176842.14	IRX5	-6.15	4.79E-16	6.48E-14
205	ENSG00000121057.12	AKAP1	1.27	4.94E-16	6.66E-14
206	ENSG00000101166.15	PRELID3B	1.01	5.55E-16	7.44E-14
207	ENSG00000152556.16	PFKM	1.43	5.77E-16	7.69E-14
208	ENSG00000110693.17	SOX6	1.64	5.92E-16	7.86E-14
209	ENSG00000213619.9	NDUFS3	1.31	5.97E-16	7.88E-14
210	ENSG00000280587.1	LINC01348	3.84	7.89E-16	1.04E-13
211	ENSG00000046653.14	GPM6B	1.46	7.99E-16	1.05E-13
212	ENSG00000090266.12	NDUFB2	1.20	8.24E-16	1.07E-13
213	ENSG00000135638.13	EMX1	4.70	9.24E-16	1.20E-13
214	ENSG00000062485.18	CS	1.39	9.38E-16	1.21E-13
215	ENSG00000140374.15	ETFA	1.01	1.02E-15	1.31E-13
216	ENSG00000177150.12	FAM210A	1.32	1.06E-15	1.36E-13
217	ENSG00000146701.11	MDH2	1.34	1.19E-15	1.51E-13
218	ENSG00000107262.21	BAG1	1.06	1.27E-15	1.61E-13
219	ENSG00000103423.13	DNAJA3	1.03	1.29E-15	1.63E-13
220	ENSG00000163958.13	ZDHHC19	7.86	1.36E-15	1.71E-13
221	ENSG00000242114.5	MTFP1	4.56	2.05E-15	2.56E-13
222	ENSG00000133315.10	MACROD1	1.72	2.24E-15	2.78E-13
223	ENSG00000156467.9	UQCRB	1.36	2.76E-15	3.41E-13
224	ENSG00000114378.16	HYAL1	1.79	4.28E-15	5.27E-13
225	ENSG00000116678.19	LEPR	1.64	4.93E-15	6.05E-13
226	ENSG00000163110.14	PDLIM5	0.91	5.05E-15	6.17E-13
227	ENSG00000180211.5	FO393411.1	7.88	6.18E-15	7.52E-13
228	ENSG00000005020.12	SKAP2	1.07	6.34E-15	7.67E-13
229	ENSG00000101365.20	IDH3B	1.14	8.46E-15	1.02E-12
230	ENSG00000180530.10	NRIP1	-1.12	9.39E-15	1.13E-12
231	ENSG00000108511.9	HOXB6	-1.83	9.81E-15	1.17E-12
232	ENSG00000196850.5	PPTC7	1.43	1.09E-14	1.30E-12
233	ENSG00000078070.12	MCCC1	1.21	1.15E-14	1.36E-12
234	ENSG00000198959.11	TGM2	3.43	1.38E-14	1.62E-12
235	ENSG00000004779.9	NDUFAB1	1.32	1.38E-14	1.62E-12
236	ENSG00000210082.2	MT-RNR2	1.13	1.60E-14	1.87E-12
237	ENSG00000206579.8	XKR4	1.13	1.64E-14	1.91E-12
238	ENSG00000120992.17	LYPLA1	1.11	2.00E-14	2.32E-12
239	ENSG00000116688.16	MFN2	0.86	2.08E-14	2.41E-12
240	ENSG00000170873.18	MTSS1	1.41	2.21E-14	2.54E-12
241	ENSG00000185432.11	METTL7A	0.91	2.35E-14	2.70E-12
242	ENSG00000111817.17	DSE	-0.89	2.37E-14	2.71E-12
243	ENSG00000182180.13	MRPS16	0.95	2.66E-14	3.02E-12
244	ENSG00000145494.11	NDUFS6	1.00	2.82E-14	3.19E-12
245	ENSG00000116459.10	ATP5PB	1.38	3.00E-14	3.38E-12
246	ENSG00000067829.18	IDH3G	1.57	3.24E-14	3.63E-12
247	ENSG00000125872.7	LRRN4	3.22	3.40E-14	3.80E-12

248	ENSG00000110435.11	PDHX	1.22	3.42E-14	3.80E-12
249	ENSG00000259494.1	MRPL46	1.15	3.45E-14	3.83E-12
250	ENSG00000166136.15	NDUFB8	1.10	4.12E-14	4.56E-12
251	ENSG00000231453.1	LINC01305	-5.90	4.37E-14	4.81E-12
252	ENSG00000166825.13	ANPEP	-1.43	4.80E-14	5.26E-12
253	ENSG00000103876.12	FAH	0.94	4.94E-14	5.39E-12
254	ENSG00000111271.14	ACAD10	1.07	5.06E-14	5.50E-12
255	ENSG00000241468.7	ATP5MF	0.92	5.64E-14	6.10E-12
256	ENSG00000164919.10	COX6C	1.28	6.19E-14	6.64E-12
257	ENSG00000125445.10	MRPS7	1.17	6.17E-14	6.64E-12
258	ENSG00000118596.11	SLC16A7	1.15	6.20E-14	6.64E-12
259	ENSG00000122873.11	CISD1	1.83	6.39E-14	6.76E-12
260	ENSG00000115541.10	HSPE1	1.47	6.38E-14	6.76E-12
261	ENSG00000172551.10	MUCL1	-22.79	6.38E-14	6.76E-12
262	ENSG00000112992.16	NNT	1.16	6.45E-14	6.79E-12
263	ENSG00000183605.16	SFXN4	1.22	8.13E-14	8.54E-12
264	ENSG00000105135.15	ILVBL	1.30	1.08E-13	1.13E-11
265	ENSG00000151552.11	QDPR	1.07	1.21E-13	1.26E-11
266	ENSG00000106554.12	CHCHD3	0.97	1.26E-13	1.31E-11
267	ENSG00000089225.19	TBX5	1.65	1.28E-13	1.32E-11
268	ENSG00000171195.10	MUC7	-22.47	1.43E-13	1.47E-11
269	ENSG00000130312.6	MRPL34	1.21	1.68E-13	1.72E-11
270	ENSG00000147853.16	AK3	0.95	2.15E-13	2.20E-11
271	ENSG00000136854.20	STXBP1	0.98	2.23E-13	2.27E-11
272	ENSG00000135070.14	ISCA1	0.63	2.28E-13	2.31E-11
273	ENSG00000124935.3	SCGB1D2	-22.23	2.54E-13	2.57E-11
274	ENSG00000235698.1	PA2G4P2	4.13	3.15E-13	3.18E-11
275	ENSG00000224837.1	GCSHP5	1.43	3.44E-13	3.45E-11
276	ENSG00000137274.12	BPHL	1.67	3.49E-13	3.49E-11
277	ENSG00000197576.13	HOXA4	1.70	3.55E-13	3.54E-11
278	ENSG00000146085.7	MUT	0.92	3.59E-13	3.57E-11
279	ENSG00000213462.4	ERV3-1	1.04	3.60E-13	3.57E-11
280	ENSG00000007923.15	DNAJC11	0.75	3.63E-13	3.58E-11
281	ENSG00000178952.10	TUFM	0.99	4.24E-13	4.17E-11
282	ENSG00000020181.17	ADGRA2	-1.03	4.61E-13	4.51E-11
283	ENSG00000075415.12	SLC25A3	0.94	4.99E-13	4.87E-11
284	ENSG00000166473.17	PKD1L2	-1.30	5.47E-13	5.32E-11
285	ENSG00000107331.16	ABCA2	1.04	5.88E-13	5.70E-11
286	ENSG00000262655.3	SPON1	-1.46	5.95E-13	5.75E-11
287	ENSG00000211459.2	MT-RNR1	1.11	6.43E-13	6.19E-11
288	ENSG00000091483.6	FH	1.32	6.59E-13	6.31E-11
289	ENSG00000053770.11	AP5M1	0.90	6.79E-13	6.49E-11
290	ENSG00000120332.15	TNN	0.98	7.93E-13	7.55E-11
291	ENSG00000196365.11	LONP1	0.77	1.01E-12	9.56E-11
292	ENSG00000115286.19	NDUFS7	1.10	1.02E-12	9.64E-11
293	ENSG00000125246.15	CLYBL	1.36	1.09E-12	1.02E-10
294	ENSG00000172379.20	ARNT2	2.48	1.21E-12	1.14E-10
295	ENSG00000250111.3	AC107982.1	-21.56	1.30E-12	1.21E-10
296	ENSG00000164823.10	OSGIN2	-1.32	1.45E-12	1.35E-10
297	ENSG00000115155.17	OTOF	6.73	1.55E-12	1.44E-10

298	ENSG00000104823.8	ECH1	1.14	1.58E-12	1.46E-10
299	ENSG00000165629.19	ATP5F1C	1.11	1.60E-12	1.48E-10
300	ENSG00000140990.14	NDUFB10	1.16	1.63E-12	1.50E-10
301	ENSG00000182512.4	GLRX5	1.23	1.65E-12	1.51E-10
302	ENSG00000119013.8	NDUFB3	1.23	1.72E-12	1.57E-10
303	ENSG00000023330.14	ALAS1	1.32	1.76E-12	1.60E-10
304	ENSG00000138031.14	ADCY3	1.25	1.83E-12	1.66E-10
305	ENSG00000127184.12	COX7C	1.25	1.88E-12	1.71E-10
306	ENSG00000154640.14	BTG3	1.72	2.01E-12	1.81E-10
307	ENSG00000274611.3	TBC1D3	-21.37	2.04E-12	1.84E-10
308	ENSG00000130414.11	NDUFA10	0.82	2.21E-12	1.98E-10
309	ENSG00000000971.15	CFH	-1.03	2.21E-12	1.98E-10
310	ENSG00000089199.9	CHGB	3.10	2.60E-12	2.31E-10
311	ENSG00000162391.11	FAM151A	4.20	2.75E-12	2.44E-10
312	ENSG00000177508.11	IRX3	-2.48	2.76E-12	2.44E-10
313	ENSG00000116717.12	GADD45A	-1.04	2.77E-12	2.45E-10
314	ENSG00000162078.11	ZG16B	-21.22	2.86E-12	2.51E-10
315	ENSG00000066583.11	ISOC1	0.98	2.88E-12	2.52E-10
316	ENSG00000233101.10	HOXB-AS3	-3.25	3.26E-12	2.85E-10
317	ENSG00000142611.16	PRDM16	1.61	3.30E-12	2.88E-10
318	ENSG00000131495.8	NDUFA2	1.13	4.19E-12	3.64E-10
319	ENSG00000198796.6	ALPK2	-1.53	4.22E-12	3.65E-10
320	ENSG00000122420.9	PTGFR	-2.42	4.40E-12	3.79E-10
321	ENSG00000163528.12	CHCHD4	1.04	4.61E-12	3.96E-10
322	ENSG00000152642.10	GPD1L	1.05	5.95E-12	5.10E-10
323	ENSG00000177646.18	ACAD9	0.65	6.27E-12	5.36E-10
324	ENSG00000011028.13	MRC2	-1.19	7.00E-12	5.96E-10
325	ENSG00000003137.8	CYP26B1	-1.43	7.44E-12	6.32E-10
326	ENSG00000153956.15	CACNA2D1	1.26	8.00E-12	6.78E-10
327	ENSG00000115137.11	DNAJC27	1.71	9.00E-12	7.60E-10
328	ENSG00000143512.12	HHIPL2	4.37	9.07E-12	7.64E-10
329	ENSG00000168924.14	LETM1	0.95	9.99E-12	8.39E-10
330	ENSG00000074410.13	CA12	3.33	1.03E-11	8.60E-10
331	ENSG00000066027.11	PPP2R5A	0.71	1.04E-11	8.71E-10
332	ENSG00000164405.10	UQCRQ	1.59	1.08E-11	8.98E-10
333	ENSG00000111669.14	TPI1	0.71	1.14E-11	9.44E-10
334	ENSG00000125454.11	SLC25A19	1.52	1.19E-11	9.80E-10
335	ENSG00000284691.1	AC073111.5	1.63	1.22E-11	1.00E-09
336	ENSG00000084090.13	STARD7	0.78	1.26E-11	1.04E-09
337	ENSG00000186010.18	NDUFA13	1.35	1.32E-11	1.09E-09
338	ENSG00000134020.7	PEBP4	6.66	1.37E-11	1.12E-09
339	ENSG00000095015.5	MAP3K1	0.93	1.38E-11	1.12E-09
340	ENSG00000110318.13	CEP126	-1.18	1.41E-11	1.15E-09
341	ENSG00000173436.14	MINOS1	1.03	1.43E-11	1.16E-09
342	ENSG00000243655.2	AC132825.2	2.35	1.46E-11	1.18E-09
343	ENSG00000254695.1	AC087379.2	6.09	1.52E-11	1.23E-09
344	ENSG00000115828.16	QPCT	-2.80	1.58E-11	1.26E-09
345	ENSG00000140564.11	FURIN	0.80	1.61E-11	1.29E-09
346	ENSG00000125356.6	NDUFA1	1.00	1.84E-11	1.47E-09
347	ENSG00000133026.12	MYH10	-1.07	2.06E-11	1.64E-09

348	ENSG00000007216.14	SLC13A2	-20.35	2.16E-11	1.71E-09
349	ENSG00000185038.14	MROH2A	8.97	2.21E-11	1.75E-09
350	ENSG00000255135.3	AP002360.1	1.32	2.22E-11	1.75E-09
351	ENSG00000148053.15	NTRK2	-0.79	2.32E-11	1.82E-09
352	ENSG00000159069.13	FBXW5	1.19	2.40E-11	1.89E-09
353	ENSG00000198836.9	OPA1	0.68	2.53E-11	1.98E-09
354	ENSG00000069849.10	ATP1B3	1.58	2.54E-11	1.98E-09
355	ENSG00000185818.7	NAT8L	-0.79	2.59E-11	2.01E-09
356	ENSG00000087245.12	MMP2	-1.17	2.74E-11	2.13E-09
357	ENSG00000140465.13	CYP1A1	7.37	2.85E-11	2.20E-09
358	ENSG00000105997.22	HOXA3	1.30	3.03E-11	2.34E-09
359	ENSG00000112304.10	ACOT13	1.13	3.05E-11	2.34E-09
360	ENSG00000099864.17	PALM	-0.72	3.13E-11	2.40E-09
361	ENSG00000107959.15	PITRM1	0.81	3.24E-11	2.48E-09
362	ENSG00000138207.13	RBP4	1.15	3.47E-11	2.65E-09
363	ENSG00000078668.13	VDAC3	0.66	3.56E-11	2.70E-09
364	ENSG00000170906.15	NDUFA3	1.15	3.65E-11	2.77E-09
365	ENSG00000166183.15	ASPG	4.54	3.78E-11	2.86E-09
366	ENSG00000278535.4	DHRS11	1.38	3.78E-11	2.86E-09
367	ENSG00000113924.11	HGD	3.79	4.17E-11	3.13E-09
368	ENSG00000105825.12	TFPI2	1.97	4.18E-11	3.14E-09
369	ENSG00000112149.9	CD83	1.96	4.44E-11	3.32E-09
370	ENSG00000130997.16	POLN	1.42	4.55E-11	3.40E-09
371	ENSG00000213585.10	VDAC1	0.77	4.74E-11	3.53E-09
372	ENSG00000152580.8	IGSF10	-1.57	4.81E-11	3.57E-09
373	ENSG00000139508.14	SLC46A3	0.76	4.94E-11	3.66E-09
374	ENSG00000038427.15	VCAN	-1.06	5.11E-11	3.78E-09
375	ENSG00000178982.9	EIF3K	0.90	5.13E-11	3.78E-09
376	ENSG00000163541.11	SUCLG1	1.20	5.21E-11	3.82E-09
377	ENSG00000176340.3	COX8A	1.62	5.94E-11	4.35E-09
378	ENSG00000110917.7	MLEC	0.53	6.24E-11	4.56E-09
379	ENSG00000151376.16	ME3	1.00	6.62E-11	4.83E-09
380	ENSG00000153130.17	SCOC	0.63	6.88E-11	5.00E-09
381	ENSG00000124299.13	PEPD	1.20	7.31E-11	5.30E-09
382	ENSG00000167815.11	PRDX2	1.11	7.81E-11	5.65E-09
383	ENSG00000004142.11	POLDIP2	0.81	7.86E-11	5.66E-09
384	ENSG00000060971.17	ACAA1	0.69	8.70E-11	6.26E-09
385	ENSG00000154723.12	ATP5PF	1.04	8.75E-11	6.28E-09
386	ENSG00000138495.6	COX17	1.29	9.09E-11	6.51E-09
387	ENSG00000115295.19	CLIP4	-0.68	9.32E-11	6.65E-09
388	ENSG00000166762.17	CATSPER2	1.04	9.55E-11	6.80E-09
389	ENSG00000154655.15	L3MBTL4	-1.13	9.65E-11	6.85E-09
390	ENSG00000059691.11	GATB	1.05	9.76E-11	6.91E-09
391	ENSG00000265354.3	TIMM23	0.80	9.81E-11	6.93E-09
392	ENSG00000181061.13	HIGD1A	1.00	1.03E-10	7.25E-09
393	ENSG00000211679.2	IGLC3	6.90	1.10E-10	7.73E-09
394	ENSG00000104313.18	EYA1	1.73	1.12E-10	7.81E-09
395	ENSG00000079739.16	PGM1	0.91	1.15E-10	8.03E-09
396	ENSG00000211448.11	DIO2	4.33	1.19E-10	8.32E-09
397	ENSG00000153944.10	MSI2	0.87	1.28E-10	8.90E-09

398	ENSG00000241973.10	PI4KA	0.61	1.28E-10	8.90E-09
399	ENSG00000262814.7	MRPL12	1.52	1.35E-10	9.32E-09
400	ENSG00000109819.8	PPARGC1A	2.59	1.44E-10	9.92E-09
401	ENSG00000157087.18	ATP2B2	3.01	1.54E-10	1.06E-08
402	ENSG00000054148.17	PHPT1	1.05	1.66E-10	1.14E-08
403	ENSG00000120162.9	MOB3B	-1.20	1.67E-10	1.15E-08
404	ENSG00000081377.16	CDC14B	-0.96	1.75E-10	1.20E-08
405	ENSG00000214837.8	LINC01347	2.05	1.78E-10	1.21E-08
406	ENSG00000148334.14	PTGES2	1.14	1.80E-10	1.22E-08
407	ENSG00000172201.11	ID4	-1.64	1.89E-10	1.28E-08
408	ENSG00000120662.15	MTRF1	1.20	1.95E-10	1.32E-08
409	ENSG00000099977.14	DDT	1.47	2.14E-10	1.44E-08
410	ENSG00000083123.14	BCKDHB	1.28	2.17E-10	1.46E-08
411	ENSG00000278455.1	AL606534.5	6.40	2.19E-10	1.47E-08
412	ENSG00000101084.17	RAB5IF	0.96	2.19E-10	1.47E-08
413	ENSG00000225383.7	SFTA1P	2.86	2.26E-10	1.51E-08
414	ENSG00000204568.11	MRPS18B	1.01	2.29E-10	1.53E-08
415	ENSG00000215021.8	PHB2	0.98	2.34E-10	1.56E-08
416	ENSG00000100889.11	PCK2	1.49	2.35E-10	1.56E-08
417	ENSG00000076662.9	ICAM3	2.20	2.43E-10	1.61E-08
418	ENSG00000140505.6	CYP1A2	2.91	2.65E-10	1.75E-08
419	ENSG00000143612.20	C1orf43	0.57	2.79E-10	1.84E-08
420	ENSG00000243055.1	GK-AS1	3.80	2.85E-10	1.87E-08
421	ENSG00000095321.16	CRAT	0.86	3.12E-10	2.05E-08
422	ENSG00000111110.11	PPM1H	1.06	3.25E-10	2.12E-08
423	ENSG00000117640.17	MTFR1L	0.68	3.57E-10	2.33E-08
424	ENSG00000173085.14	COQ2	1.13	3.65E-10	2.38E-08
425	ENSG00000197956.9	S100A6	-0.88	3.66E-10	2.38E-08
426	ENSG00000119723.16	COQ6	1.11	4.00E-10	2.59E-08
427	ENSG00000176907.4	TCIM	1.56	4.04E-10	2.61E-08
428	ENSG00000137409.19	MTCH1	0.55	4.10E-10	2.64E-08
429	ENSG00000187240.14	DYNC2H1	-0.64	4.18E-10	2.69E-08
430	ENSG00000123091.4	RNF11	0.52	4.26E-10	2.74E-08
431	ENSG00000172366.19	MCRIP2	1.30	4.47E-10	2.87E-08
432	ENSG00000246430.6	LINC00968	-3.72	4.64E-10	2.97E-08
433	ENSG00000164107.8	HAND2	-4.77	4.69E-10	2.99E-08
434	ENSG00000132676.15	DAP3	0.63	4.77E-10	3.03E-08
435	ENSG00000165795.23	NDRG2	0.90	4.88E-10	3.10E-08
436	ENSG00000184613.10	NELL2	-2.69	4.92E-10	3.12E-08
437	ENSG00000141385.9	AFG3L2	0.84	5.00E-10	3.16E-08
438	ENSG00000166816.14	LDHD	1.68	5.20E-10	3.28E-08
439	ENSG00000136003.15	ISCU	0.79	5.22E-10	3.28E-08
440	ENSG00000253552.7	HOXA-AS2	1.27	5.89E-10	3.69E-08
441	ENSG00000229920.2	RPS4XP5	-1.28	6.05E-10	3.79E-08
442	ENSG00000163520.13	FBLN2	-0.74	6.08E-10	3.80E-08
443	ENSG00000228872.2	AC096664.1	-3.31	6.22E-10	3.87E-08
444	ENSG00000065978.18	YBX1	0.69	6.25E-10	3.89E-08
445	ENSG00000141232.4	TOB1	1.35	6.27E-10	3.89E-08
446	ENSG00000166391.14	MOGAT2	2.91	6.34E-10	3.93E-08
447	ENSG00000115252.18	PDE1A	1.15	7.06E-10	4.36E-08

448	ENSG00000109099.14	PMP22	-0.66	7.70E-10	4.74E-08
449	ENSG00000164347.17	GFM2	1.01	7.95E-10	4.89E-08
450	ENSG00000173153.13	ESRRA	0.97	8.24E-10	5.06E-08
451	ENSG00000173915.15	ATP5MD	1.14	8.93E-10	5.47E-08
452	ENSG00000126267.9	COX6B1	1.28	9.13E-10	5.58E-08
453	ENSG00000167283.7	ATP5MG	1.10	9.38E-10	5.70E-08
454	ENSG00000113845.9	TIMMDC1	0.69	9.36E-10	5.70E-08
455	ENSG00000119729.11	RHOQ	0.77	9.93E-10	6.03E-08
456	ENSG00000115353.10	TACR1	-1.10	1.03E-09	6.25E-08
457	ENSG00000134115.12	CNTN6	-3.44	1.29E-09	7.80E-08
458	ENSG00000114999.7	TTL	0.62	1.30E-09	7.84E-08
459	ENSG00000132300.18	PTCD3	0.90	1.34E-09	8.06E-08
460	ENSG00000132329.10	RAMP1	6.67	1.36E-09	8.15E-08
461	ENSG00000114251.14	WNT5A	1.71	1.36E-09	8.15E-08
462	ENSG00000072954.6	TMEM38A	1.74	1.38E-09	8.24E-08
463	ENSG00000106078.18	COBL	4.45	1.42E-09	8.50E-08
464	ENSG00000114853.13	ZBTB47	-0.87	1.45E-09	8.63E-08
465	ENSG00000178568.14	ERBB4	-2.29	1.45E-09	8.63E-08
466	ENSG00000116774.11	OLFML3	-1.00	1.49E-09	8.84E-08
467	ENSG00000081181.7	ARG2	2.78	1.52E-09	8.99E-08
468	ENSG00000157600.11	TMEM164	1.12	1.53E-09	9.03E-08
469	ENSG00000166949.15	SMAD3	-0.66	1.54E-09	9.10E-08
470	ENSG00000196196.2	HRCT1	-1.46	1.59E-09	9.32E-08
471	ENSG00000064726.9	BTBD1	0.59	1.64E-09	9.62E-08
472	ENSG00000063854.12	HAGH	0.80	1.67E-09	9.77E-08
473	ENSG00000164258.11	NDUFS4	0.94	1.69E-09	9.86E-08
474	ENSG00000160789.19	LMNA	-0.63	1.71E-09	9.94E-08
475	ENSG00000008513.15	ST3GAL1	0.85	1.71E-09	9.97E-08
476	ENSG00000186687.15	LYRM7	0.69	1.77E-09	1.02E-07
477	ENSG00000053438.9	NNAT	-2.28	1.80E-09	1.04E-07
478	ENSG00000184983.10	NDUFA6	1.10	1.90E-09	1.10E-07
479	ENSG00000241220.1	AC078788.1	3.83	1.92E-09	1.11E-07
480	ENSG00000136521.12	NDUFB5	0.82	2.10E-09	1.20E-07
481	ENSG00000121440.14	PDZRN3	-1.06	2.10E-09	1.20E-07
482	ENSG00000216490.3	IFI30	1.42	2.11E-09	1.21E-07
483	ENSG00000176871.8	WSB2	0.72	2.11E-09	1.21E-07
484	ENSG00000178127.12	NDUFV2	1.03	2.18E-09	1.24E-07
485	ENSG00000104853.15	CLPTM1	0.77	2.23E-09	1.27E-07
486	ENSG00000073060.15	SCARB1	-0.96	2.26E-09	1.29E-07
487	ENSG00000174807.3	CD248	-0.91	2.42E-09	1.37E-07
488	ENSG00000270307.1	MTATP6P2	1.74	2.46E-09	1.39E-07
489	ENSG00000175756.13	AURKAIP1	1.15	2.55E-09	1.44E-07
490	ENSG00000013561.17	RNF14	0.58	2.60E-09	1.47E-07
491	ENSG00000117592.8	PRDX6	0.71	2.72E-09	1.53E-07
492	ENSG00000060718.21	COL11A1	-5.28	2.74E-09	1.54E-07
493	ENSG00000012061.15	ERCC1	0.85	2.91E-09	1.63E-07
494	ENSG00000162643.12	WDR63	3.37	2.92E-09	1.63E-07
495	ENSG00000158286.12	RNF207	1.48	3.09E-09	1.72E-07
496	ENSG00000166228.8	PCBD1	1.22	3.09E-09	1.72E-07
497	ENSG00000108018.15	SORCS1	1.84	3.10E-09	1.72E-07

498	ENSG00000172137.18	CALB2	-1.34	3.13E-09	1.73E-07
499	ENSG00000196912.12	ANKRD36B	-0.71	3.18E-09	1.76E-07
500	ENSG00000186951.16	PPARA	1.36	3.24E-09	1.79E-07
501	ENSG00000198542.13	ITGBL1	-1.45	3.25E-09	1.79E-07
502	ENSG00000023734.10	STRAP	0.49	3.34E-09	1.84E-07
503	ENSG00000092607.14	TBX15	-1.09	3.34E-09	1.84E-07
504	ENSG00000230437.1	AL136460.1	6.38	3.36E-09	1.84E-07
505	ENSG00000198060.9	MARCH5	0.72	3.38E-09	1.85E-07
506	ENSG00000184347.14	SLIT3	-0.71	3.40E-09	1.85E-07
507	ENSG00000134343.12	ANO3	-1.84	3.48E-09	1.89E-07
508	ENSG00000102924.11	CBLN1	1.86	3.50E-09	1.90E-07
509	ENSG00000184076.13	UQCR10	1.42	3.66E-09	1.98E-07
510	ENSG00000133318.13	RTN3	0.67	3.79E-09	2.05E-07
511	ENSG00000005194.14	CIAPIN1	0.71	3.80E-09	2.06E-07
512	ENSG00000152270.8	PDE3B	0.81	3.90E-09	2.10E-07
513	ENSG00000225792.1	AC004540.2	3.44	3.99E-09	2.15E-07
514	ENSG00000138078.15	PREPL	0.66	4.06E-09	2.18E-07
515	ENSG00000134375.10	TIMM17A	0.83	4.08E-09	2.19E-07
516	ENSG00000172172.7	MRPL13	0.96	4.12E-09	2.20E-07
517	ENSG00000161281.10	COX7A1	1.21	4.22E-09	2.26E-07
518	ENSG00000128928.8	IVD	0.78	4.31E-09	2.30E-07
519	ENSG00000144837.8	PLA1A	2.46	4.75E-09	2.53E-07
520	ENSG00000163050.16	COQ8A	1.18	4.76E-09	2.53E-07
521	ENSG00000123684.12	LPGAT1	-0.92	5.08E-09	2.69E-07
522	ENSG00000170561.12	IRX2	-2.57	5.12E-09	2.71E-07
523	ENSG00000104980.7	TIMM44	1.08	5.18E-09	2.73E-07
524	ENSG00000232320.6	AC009299.2	-1.14	5.17E-09	2.73E-07
525	ENSG00000108828.15	VAT1	-0.53	5.28E-09	2.78E-07
526	ENSG00000116690.12	PRG4	1.53	5.30E-09	2.78E-07
527	ENSG00000157884.10	CIB4	5.90	5.41E-09	2.84E-07
528	ENSG00000100280.16	AP1B1	0.66	5.48E-09	2.87E-07
529	ENSG00000158747.14	NBL1	-1.12	5.63E-09	2.94E-07
530	ENSG00000137809.16	ITGA11	-1.09	5.70E-09	2.97E-07
531	ENSG00000071794.15	HLTF	0.62	5.91E-09	3.07E-07
532	ENSG00000177133.10	LINC00982	1.90	6.00E-09	3.11E-07
533	ENSG00000182255.6	KCNA4	-2.37	6.01E-09	3.12E-07
534	ENSG00000259070.5	LINC00639	1.25	6.27E-09	3.24E-07
535	ENSG00000139910.19	NOVA1	-1.06	6.49E-09	3.35E-07
536	ENSG00000105364.13	MRPL4	1.11	6.78E-09	3.49E-07
537	ENSG00000102144.14	PGK1	0.69	6.85E-09	3.52E-07
538	ENSG00000125354.22	SEPT6	0.87	7.26E-09	3.72E-07
539	ENSG00000137038.7	DMAC1	0.84	7.43E-09	3.80E-07
540	ENSG00000130349.9	C6orf203	0.93	7.45E-09	3.81E-07
541	ENSG00000104419.14	NDRG1	-0.70	7.56E-09	3.86E-07
542	ENSG00000224389.9	C4B	3.05	7.59E-09	3.86E-07
543	ENSG00000144152.12	FBLN7	-1.70	7.59E-09	3.86E-07
544	ENSG00000151498.11	ACAD8	0.78	8.04E-09	4.08E-07
545	ENSG00000152256.13	PDK1	1.13	8.08E-09	4.09E-07
546	ENSG00000124532.14	MRS2	0.74	8.23E-09	4.16E-07
547	ENSG00000131016.16	AKAP12	-0.90	8.53E-09	4.31E-07

548	ENSG00000143416.20	SELENBP1	0.73	8.83E-09	4.45E-07
549	ENSG00000139644.12	TMBIM6	0.46	9.16E-09	4.61E-07
550	ENSG00000134056.11	MRPS36	1.35	9.22E-09	4.63E-07
551	ENSG00000181218.5	HIST3H2A	3.92	9.51E-09	4.76E-07
552	ENSG00000224370.1	AC093392.2	2.02	9.54E-09	4.77E-07
553	ENSG00000136463.7	TACO1	0.93	1.06E-08	5.31E-07
554	ENSG00000160785.13	SLC25A44	1.17	1.09E-08	5.44E-07
555	ENSG00000130204.12	TOMM40	0.74	1.09E-08	5.44E-07
556	ENSG00000116898.11	MRPS15	0.86	1.14E-08	5.67E-07
557	ENSG00000182732.17	RGS6	1.18	1.16E-08	5.76E-07
558	ENSG00000042445.13	RETSAT	0.74	1.20E-08	5.93E-07
559	ENSG00000167363.13	FN3K	1.03	1.20E-08	5.95E-07
560	ENSG00000180185.11	FAHD1	1.15	1.21E-08	5.98E-07
561	ENSG00000250305.8	TRMT9B	1.13	1.24E-08	6.11E-07
562	ENSG00000271811.1	Z97200.1	-1.12	1.27E-08	6.22E-07
563	ENSG00000116353.15	MECR	1.01	1.37E-08	6.74E-07
564	ENSG00000158882.14	TOMM40L	1.00	1.41E-08	6.89E-07
565	ENSG00000125648.14	SLC25A23	0.99	1.41E-08	6.90E-07
566	ENSG00000091262.15	ABCC6	0.72	1.45E-08	7.08E-07
567	ENSG00000141576.15	RNF157	1.37	1.47E-08	7.14E-07
568	ENSG00000243678.11	NME2	0.76	1.48E-08	7.18E-07
569	ENSG00000112039.4	FANCE	0.97	1.55E-08	7.52E-07
570	ENSG00000124203.6	ZNF831	2.70	1.57E-08	7.60E-07
571	ENSG00000176490.4	DIRAS1	4.35	1.57E-08	7.61E-07
572	ENSG00000108242.12	CYP2C18	3.43	1.65E-08	7.95E-07
573	ENSG00000160688.18	FLAD1	0.81	1.67E-08	8.05E-07
574	ENSG00000182118.7	FAM89A	0.71	1.71E-08	8.23E-07
575	ENSG00000165637.13	VDAC2	0.98	1.80E-08	8.62E-07
576	ENSG00000067704.9	IARS2	0.67	1.81E-08	8.69E-07
577	ENSG00000111640.14	GAPDH	0.95	1.84E-08	8.79E-07
578	ENSG00000112335.14	SNX3	0.53	1.84E-08	8.79E-07
579	ENSG00000136111.13	TBC1D4	0.45	1.85E-08	8.80E-07
580	ENSG00000100216.5	TOMM22	0.59	1.87E-08	8.92E-07
581	ENSG00000248098.11	BCKDHA	1.46	1.89E-08	8.97E-07
582	ENSG00000137547.8	MRPL15	1.22	1.89E-08	8.97E-07
583	ENSG00000162670.10	BRINP3	3.44	1.97E-08	9.34E-07
584	ENSG00000134240.11	HMGCS2	6.99	2.05E-08	9.69E-07
585	ENSG00000100580.7	TMED8	0.58	2.05E-08	9.69E-07
586	ENSG00000117305.14	HMGCL	0.56	2.11E-08	9.95E-07
587	ENSG00000111666.10	CHPT1	0.88	2.13E-08	1.00E-06
588	ENSG00000132554.19	RGS22	-0.98	2.14E-08	1.01E-06
589	ENSG00000091136.13	LAMB1	0.83	2.17E-08	1.02E-06
590	ENSG00000124302.12	CHST8	4.74	2.18E-08	1.02E-06
591	ENSG00000185567.6	AHNAK2	-1.30	2.30E-08	1.08E-06
592	ENSG00000196177.12	ACADSB	0.79	2.32E-08	1.08E-06
593	ENSG00000165264.10	NDUFB6	0.90	2.40E-08	1.12E-06
594	ENSG00000085760.14	MTIF2	0.81	2.41E-08	1.12E-06
595	ENSG00000128654.13	MTX2	0.90	2.42E-08	1.12E-06
596	ENSG00000134779.14	TPGS2	-0.62	2.42E-08	1.12E-06
597	ENSG00000173040.12	EVC2	-0.78	2.50E-08	1.16E-06

598	ENSG00000244731.7	C4A	2.64	2.62E-08	1.21E-06
599	ENSG00000026508.18	CD44	-0.84	2.63E-08	1.21E-06
600	ENSG00000149925.18	ALDOA	0.71	2.74E-08	1.26E-06
601	ENSG00000197747.8	S100A10	-0.89	2.78E-08	1.28E-06
602	ENSG00000102802.9	MEDAG	-0.87	2.97E-08	1.36E-06
603	ENSG00000165283.15	STOML2	0.63	3.00E-08	1.38E-06
604	ENSG00000122592.7	HOXA7	1.46	3.03E-08	1.38E-06
605	ENSG00000027001.9	MIPEP	0.92	3.03E-08	1.39E-06
606	ENSG00000136546.14	SCN7A	1.53	3.07E-08	1.40E-06
607	ENSG00000010932.16	FMO1	-1.56	3.08E-08	1.40E-06
608	ENSG00000244187.7	TMEM141	1.18	3.09E-08	1.40E-06
609	ENSG00000011465.16	DCN	-0.85	3.12E-08	1.42E-06
610	ENSG00000124523.15	SIRT5	0.68	3.19E-08	1.44E-06
611	ENSG00000198732.10	SMOC1	1.15	3.24E-08	1.47E-06
612	ENSG00000178209.15	PLEC	-0.48	3.25E-08	1.47E-06
613	ENSG00000255248.8	MIR100HG	-0.84	3.37E-08	1.52E-06
614	ENSG00000155970.11	MICU3	1.03	3.42E-08	1.54E-06
615	ENSG00000100348.9	TXN2	0.79	3.44E-08	1.55E-06
616	ENSG00000142910.15	TINAGL1	0.77	3.48E-08	1.56E-06
617	ENSG00000108561.8	C1QBP	1.05	3.49E-08	1.56E-06
618	ENSG00000124172.9	ATP5F1E	0.77	3.54E-08	1.58E-06
619	ENSG00000127920.5	GNG11	0.82	3.69E-08	1.64E-06
620	ENSG00000205726.14	ITSN1	-0.88	3.75E-08	1.67E-06
621	ENSG00000162763.3	LRRC52	6.35	3.78E-08	1.68E-06
622	ENSG00000156411.9	ATP5MPL	1.00	3.81E-08	1.69E-06
623	ENSG00000156381.8	ANKRD9	1.24	3.82E-08	1.69E-06
624	ENSG00000266524.2	GDF10	-2.38	3.84E-08	1.70E-06
625	ENSG00000169155.9	ZBTB43	0.65	3.97E-08	1.75E-06
626	ENSG00000144843.11	ADPRH	1.02	4.15E-08	1.83E-06
627	ENSG00000278970.1	HEIH	0.94	4.15E-08	1.83E-06
628	ENSG00000004961.14	HCCS	0.89	4.17E-08	1.83E-06
629	ENSG00000134452.19	FBH1	0.49	4.17E-08	1.83E-06
630	ENSG00000087460.24	GNAS	0.64	4.32E-08	1.89E-06
631	ENSG00000259495.2	AC016705.2	1.97	4.38E-08	1.92E-06
632	ENSG00000233184.6	AC093157.1	1.43	4.54E-08	1.98E-06
633	ENSG00000214435.7	AS3MT	1.13	4.58E-08	1.99E-06
634	ENSG00000143507.17	DUSP10	1.05	4.58E-08	1.99E-06
635	ENSG00000217236.1	SP9	-6.18	4.61E-08	2.01E-06
636	ENSG00000150433.9	TMEM218	0.94	4.73E-08	2.05E-06
637	ENSG00000175110.11	MRPS22	0.67	4.93E-08	2.14E-06
638	ENSG00000138018.17	SELENOI	1.05	5.15E-08	2.23E-06
639	ENSG00000158966.14	CACHD1	-0.90	5.17E-08	2.23E-06
640	ENSG00000100994.11	PYGB	-0.88	5.27E-08	2.27E-06
641	ENSG00000140545.14	MFGE8	-0.75	5.43E-08	2.34E-06
642	ENSG00000148965.9	SAA4	6.93	5.63E-08	2.42E-06
643	ENSG00000112996.10	MRPS30	0.62	5.68E-08	2.44E-06
644	ENSG00000115290.9	GRB14	1.77	5.93E-08	2.54E-06
645	ENSG00000140459.17	CYP11A1	1.85	5.95E-08	2.54E-06
646	ENSG00000180817.11	PPA1	0.81	5.95E-08	2.54E-06
647	ENSG00000135537.16	AFG1L	1.00	6.19E-08	2.64E-06

648	ENSG00000104369.4	JPH1	2.07	6.56E-08	2.80E-06
649	ENSG00000255282.6	WTAPP1	-3.30	6.66E-08	2.83E-06
650	ENSG00000136478.7	TEX2	0.55	7.07E-08	3.00E-06
651	ENSG00000278934.1	AC117489.1	-1.16	7.17E-08	3.04E-06
652	ENSG00000088766.11	CRLS1	0.84	7.27E-08	3.08E-06
653	ENSG00000172927.7	MYEOV	-2.90	7.39E-08	3.13E-06
654	ENSG00000243989.8	ACY1	1.07	7.59E-08	3.21E-06
655	ENSG00000198130.15	HIBCH	0.77	7.65E-08	3.23E-06
656	ENSG00000162543.5	UBXN10	-3.16	8.38E-08	3.53E-06
657	ENSG00000158445.9	KCNB1	-1.02	8.41E-08	3.53E-06
658	ENSG00000112096.17	SOD2	0.44	8.48E-08	3.56E-06
659	ENSG00000254254.5	AC012349.1	-0.77	8.51E-08	3.56E-06
660	ENSG00000140905.10	GCSH	0.87	8.55E-08	3.58E-06
661	ENSG00000169738.7	DCXR	1.06	9.12E-08	3.81E-06
662	ENSG00000256694.1	AC026369.2	1.59	9.33E-08	3.89E-06
663	ENSG00000102543.14	CDADC1	0.78	9.51E-08	3.96E-06
664	ENSG00000171612.6	SLC25A33	0.96	9.75E-08	4.06E-06
665	ENSG00000239911.2	PRKAG2-AS1	1.48	9.80E-08	4.07E-06
666	ENSG00000198721.12	ECI2	0.80	9.89E-08	4.10E-06
667	ENSG00000159199.13	ATP5MC1	0.78	9.95E-08	4.12E-06
668	ENSG00000035862.12	TIMP2	-0.75	9.96E-08	4.12E-06
669	ENSG00000123545.5	NDUFAF4	1.08	1.00E-07	4.14E-06
670	ENSG00000152527.13	PLEKHH2	-0.87	1.07E-07	4.42E-06
671	ENSG00000178425.13	NT5DC1	0.64	1.10E-07	4.52E-06
672	ENSG00000106689.10	LHX2	5.66	1.10E-07	4.53E-06
673	ENSG00000198300.13	PEG3	1.02	1.14E-07	4.68E-06
674	ENSG00000136522.13	MRPL47	1.03	1.16E-07	4.77E-06
675	ENSG00000128923.10	MINDY2	0.66	1.17E-07	4.78E-06
676	ENSG00000103356.15	EARS2	0.74	1.21E-07	4.93E-06
677	ENSG00000102901.12	CENPT	-0.58	1.23E-07	5.03E-06
678	ENSG00000169020.9	ATP5ME	1.11	1.26E-07	5.15E-06
679	ENSG00000169083.16	AR	-0.76	1.27E-07	5.17E-06
680	ENSG00000175445.15	LPL	0.75	1.29E-07	5.26E-06
681	ENSG00000167701.13	GPT	1.18	1.35E-07	5.47E-06
682	ENSG00000092068.19	SLC7A8	0.83	1.47E-07	5.97E-06
683	ENSG00000009694.13	TENM1	-0.87	1.50E-07	6.06E-06
684	ENSG00000162542.13	TMCO4	0.70	1.51E-07	6.10E-06
685	ENSG00000197448.13	GSTK1	0.54	1.55E-07	6.26E-06
686	ENSG00000124198.8	ARFGEF2	0.52	1.61E-07	6.49E-06
687	ENSG00000145824.12	CXCL14	-1.08	1.62E-07	6.50E-06
688	ENSG00000162496.8	DHRS3	0.57	1.69E-07	6.77E-06
689	ENSG00000019144.18	PHLDB1	-1.13	1.70E-07	6.82E-06
690	ENSG00000129422.14	MTUS1	0.65	1.75E-07	6.99E-06
691	ENSG00000239887.4	C1orf226	-1.78	1.77E-07	7.07E-06
692	ENSG00000134013.15	LOXL2	-0.81	1.85E-07	7.39E-06
693	ENSG00000145730.20	PAM	-0.59	1.87E-07	7.47E-06
694	ENSG00000184227.7	ACOT1	1.13	1.91E-07	7.58E-06
695	ENSG00000138792.9	ENPEP	0.62	1.94E-07	7.72E-06
696	ENSG00000108788.11	MLX	0.84	1.95E-07	7.75E-06
697	ENSG00000155755.18	TMEM237	-0.73	1.96E-07	7.77E-06

698	ENSG00000137675.4	MMP27	-2.49	2.03E-07	8.03E-06
699	ENSG00000002834.17	LASP1	-0.65	2.05E-07	8.10E-06
700	ENSG00000196839.12	ADA	-0.85	2.15E-07	8.50E-06
701	ENSG00000186854.10	TRABD2A	-1.17	2.16E-07	8.52E-06
702	ENSG00000187109.13	NAP1L1	-0.44	2.22E-07	8.73E-06
703	ENSG00000064763.10	FAR2	1.09	2.28E-07	8.94E-06
704	ENSG00000092148.12	HECTD1	0.44	2.28E-07	8.94E-06
705	ENSG00000196199.13	MPHOSPH8	-0.43	2.29E-07	8.96E-06
706	ENSG00000139132.14	FGD4	0.64	2.34E-07	9.15E-06
707	ENSG00000129596.4	CDO1	1.06	2.43E-07	9.49E-06
708	ENSG00000113140.10	SPARC	-0.82	2.52E-07	9.84E-06
709	ENSG00000171357.5	LURAP1	1.12	2.53E-07	9.87E-06
710	ENSG00000130520.10	LSM4	0.68	2.60E-07	1.01E-05
711	ENSG00000099624.7	ATP5F1D	1.11	2.73E-07	1.06E-05
712	ENSG00000164292.12	RHOBTB3	0.85	2.78E-07	1.08E-05
713	ENSG00000187048.12	CYP4A11	-1.37	2.79E-07	1.08E-05
714	ENSG00000186340.15	THBS2	-1.25	2.87E-07	1.11E-05
715	ENSG00000189060.5	H1F0	-0.72	2.90E-07	1.12E-05
716	ENSG00000164062.12	APEH	0.73	3.09E-07	1.19E-05
717	ENSG00000256566.1	AL049650.1	-6.63	3.18E-07	1.22E-05
718	ENSG00000137124.7	ALDH1B1	1.10	3.21E-07	1.23E-05
719	ENSG00000140937.13	CDH11	-1.29	3.33E-07	1.28E-05
720	ENSG00000183648.9	NDUFB1	1.07	3.39E-07	1.30E-05
721	ENSG00000204257.14	HLA-DMA	0.89	3.41E-07	1.31E-05
722	ENSG00000235434.1	AL391883.1	3.88	3.49E-07	1.34E-05
723	ENSG00000074800.15	ENO1	0.65	3.67E-07	1.40E-05
724	ENSG00000138032.20	PPM1B	0.77	3.73E-07	1.42E-05
725	ENSG00000180190.11	TDRP	0.84	3.83E-07	1.46E-05
726	ENSG00000178031.16	ADAMTSL1	-1.25	3.94E-07	1.50E-05
727	ENSG00000126353.3	CCR7	3.74	4.18E-07	1.59E-05
728	ENSG00000197594.12	ENPP1	-1.01	4.22E-07	1.60E-05
729	ENSG00000248485.1	PCP4L1	2.05	4.23E-07	1.60E-05
730	ENSG00000143367.15	TUFT1	-1.37	4.25E-07	1.61E-05
731	ENSG00000278962.1	AC092645.1	-1.62	4.37E-07	1.65E-05
732	ENSG00000100554.11	ATP6V1D	0.81	4.44E-07	1.67E-05
733	ENSG00000168502.17	MTCL1	-1.72	4.46E-07	1.68E-05
734	ENSG00000128609.14	NDUFA5	0.61	4.67E-07	1.76E-05
735	ENSG00000109084.13	TMEM97	1.53	4.79E-07	1.80E-05
736	ENSG00000135298.13	ADGRB3	0.90	4.79E-07	1.80E-05
737	ENSG00000182768.8	NGRN	0.49	4.90E-07	1.84E-05
738	ENSG00000167863.11	ATP5PD	0.77	5.06E-07	1.89E-05
739	ENSG00000130300.8	PLVAP	-0.79	5.21E-07	1.95E-05
740	ENSG00000106976.20	DNM1	-1.01	5.28E-07	1.97E-05
741	ENSG00000124785.8	NRN1	-0.65	5.45E-07	2.03E-05
742	ENSG00000150938.9	CRIM1	-0.83	5.59E-07	2.08E-05
743	ENSG00000175356.13	SCUBE2	-1.11	5.59E-07	2.08E-05
744	ENSG00000164692.17	COL1A2	-0.89	5.74E-07	2.13E-05
745	ENSG00000128709.12	HOXD9	-2.42	5.78E-07	2.14E-05
746	ENSG00000165731.18	RET	-1.29	5.82E-07	2.16E-05
747	ENSG00000197757.7	HOXC6	-1.60	5.93E-07	2.19E-05

748	ENSG00000142875.19	PRKACB	0.48	5.97E-07	2.20E-05
749	ENSG00000215458.8	AATBC	1.77	6.00E-07	2.21E-05
750	ENSG00000258818.3	RNASE4	-0.74	6.26E-07	2.30E-05
751	ENSG00000114529.12	C3orf52	-1.92	6.35E-07	2.33E-05
752	ENSG00000186583.11	SPATC1	3.71	6.50E-07	2.39E-05
753	ENSG00000151806.13	GUF1	0.61	6.62E-07	2.43E-05
754	ENSG00000170262.12	MRAP	1.39	6.82E-07	2.50E-05
755	ENSG00000174840.8	PDE12	0.45	6.87E-07	2.51E-05
756	ENSG00000103005.11	USB1	0.73	6.88E-07	2.51E-05
757	ENSG00000136867.10	SLC31A2	-1.16	7.00E-07	2.56E-05
758	ENSG00000112651.11	MRPL2	0.67	7.14E-07	2.60E-05
759	ENSG00000064042.17	LIMCH1	-0.94	7.16E-07	2.60E-05
760	ENSG00000131446.16	MGAT1	0.51	7.31E-07	2.66E-05
761	ENSG00000234840.1	LINC01239	-1.16	7.56E-07	2.74E-05
762	ENSG00000071282.11	LMCD1	-0.86	7.69E-07	2.79E-05
763	ENSG00000182742.5	HOXB4	-0.68	7.74E-07	2.80E-05
764	ENSG00000186472.19	PCLO	-2.92	7.75E-07	2.80E-05
765	ENSG00000198843.12	SELENOT	0.45	7.88E-07	2.84E-05
766	ENSG00000091129.19	NRCAM	-4.91	8.13E-07	2.93E-05
767	ENSG00000101439.8	CST3	0.99	8.15E-07	2.93E-05
768	ENSG00000103342.12	GSPT1	0.48	8.16E-07	2.94E-05
769	ENSG00000167085.11	PHB	0.55	8.18E-07	2.94E-05
770	ENSG00000114125.13	RNF7	0.89	8.82E-07	3.16E-05
771	ENSG00000022267.16	FHL1	-0.81	8.84E-07	3.17E-05
772	ENSG00000151914.19	DST	-0.40	9.17E-07	3.28E-05
773	ENSG00000165948.10	IFI27L1	0.80	9.31E-07	3.33E-05
774	ENSG00000125967.16	NECAB3	0.94	9.33E-07	3.33E-05
775	ENSG00000151366.12	NDUFC2	0.66	9.87E-07	3.52E-05
776	ENSG00000102225.15	CDK16	0.64	9.99E-07	3.56E-05
777	ENSG00000224273.2	AC005077.2	3.80	1.02E-06	3.64E-05
778	ENSG00000163597.14	SNHG16	0.62	1.03E-06	3.66E-05
779	ENSG00000105996.6	HOXA2	1.51	1.03E-06	3.66E-05
780	ENSG00000138669.9	PRKG2	-2.34	1.04E-06	3.67E-05
781	ENSG00000264522.5	OTUD7B	-0.72	1.09E-06	3.84E-05
782	ENSG00000204922.4	UQCC3	0.79	1.09E-06	3.86E-05
783	ENSG00000197712.11	FAM114A1	-0.72	1.12E-06	3.94E-05
784	ENSG00000156469.8	MTERF3	1.26	1.13E-06	3.97E-05
785	ENSG00000133083.14	DCLK1	-0.68	1.13E-06	3.97E-05
786	ENSG00000124942.13	AHNAK	-0.64	1.14E-06	4.01E-05
787	ENSG00000136842.13	TMOD1	1.30	1.16E-06	4.05E-05
788	ENSG00000128482.15	RNF112	-1.77	1.16E-06	4.08E-05
789	ENSG00000006695.10	COX10	0.82	1.17E-06	4.09E-05
790	ENSG00000169100.13	SLC25A6	0.75	1.19E-06	4.14E-05
791	ENSG00000231490.1	RPL7L1P2	6.32	1.20E-06	4.18E-05
792	ENSG00000049167.14	ERCC8	0.73	1.20E-06	4.19E-05
793	ENSG00000132423.11	COQ3	1.32	1.22E-06	4.24E-05
794	ENSG00000227143.1	LINC01153	-4.94	1.30E-06	4.53E-05
795	ENSG00000135617.3	PRADC1	0.92	1.35E-06	4.68E-05
796	ENSG00000269954.2	AC022239.1	3.08	1.36E-06	4.72E-05
797	ENSG00000184979.9	USP18	-1.03	1.38E-06	4.77E-05

798	ENSG00000065989.15	PDE4A	0.64	1.39E-06	4.81E-05
799	ENSG00000100568.10	VTI1B	0.46	1.40E-06	4.82E-05
800	ENSG00000089486.16	CDIP1	0.68	1.40E-06	4.82E-05
801	ENSG00000163644.14	PPM1K	0.61	1.40E-06	4.82E-05
802	ENSG00000214456.8	PLIN5	0.70	1.40E-06	4.82E-05
803	ENSG00000165672.6	PRDX3	0.67	1.40E-06	4.82E-05
804	ENSG00000126500.3	FLRT1	1.50	1.41E-06	4.84E-05
805	ENSG00000136783.9	NIPSNAP3A	0.60	1.41E-06	4.84E-05
806	ENSG00000258603.3	AC005225.2	1.95	1.43E-06	4.91E-05
807	ENSG00000187134.13	AKR1C1	0.64	1.50E-06	5.12E-05
808	ENSG00000174501.14	ANKRD36C	-0.85	1.50E-06	5.14E-05
809	ENSG00000138821.12	SLC39A8	1.36	1.55E-06	5.28E-05
810	ENSG00000244005.12	NFS1	0.71	1.55E-06	5.28E-05
811	ENSG00000111962.7	UST	-1.20	1.57E-06	5.35E-05
812	ENSG00000124253.10	PCK1	1.69	1.58E-06	5.37E-05
813	ENSG00000168803.15	ADAL	0.72	1.59E-06	5.38E-05
814	ENSG00000233377.1	MTND4P20	3.22	1.61E-06	5.45E-05
815	ENSG00000148672.8	GLUD1	0.45	1.61E-06	5.45E-05
816	ENSG00000014641.17	MDH1	1.01	1.63E-06	5.51E-05
817	ENSG00000011376.10	LARS2	0.71	1.63E-06	5.51E-05
818	ENSG00000134508.12	CABLES1	0.78	1.66E-06	5.58E-05
819	ENSG00000107537.13	PHYH	0.70	1.66E-06	5.58E-05
820	ENSG00000262454.3	MIR193BHG	0.67	1.68E-06	5.64E-05
821	ENSG00000163170.11	BOLA3	1.22	1.71E-06	5.73E-05
822	ENSG00000184828.9	ZBTB7C	-0.99	1.72E-06	5.78E-05
823	ENSG00000120262.9	CCDC170	-0.94	1.73E-06	5.80E-05
824	ENSG00000226085.3	UQCRFS1P1	1.73	1.74E-06	5.81E-05
825	ENSG00000187546.13	AGMO	1.11	1.74E-06	5.81E-05
826	ENSG00000170370.11	EMX2	-0.93	1.75E-06	5.84E-05
827	ENSG00000149451.17	ADAM33	-0.99	1.77E-06	5.90E-05
828	ENSG00000172037.13	LAMB2	-0.55	1.80E-06	5.98E-05
829	ENSG00000229604.2	MTATP8P2	2.07	1.80E-06	6.00E-05
830	ENSG00000148948.7	LRRC4C	-2.13	1.82E-06	6.06E-05
831	ENSG00000064655.18	EYA2	1.31	1.84E-06	6.09E-05
832	ENSG00000257923.10	CUX1	0.47	1.84E-06	6.11E-05
833	ENSG00000162745.10	OLFML2B	-0.93	1.86E-06	6.15E-05
834	ENSG00000166963.12	MAP1A	-0.84	1.86E-06	6.16E-05
835	ENSG00000133028.11	SCO1	0.51	1.87E-06	6.17E-05
836	ENSG00000260549.1	MT1L	3.03	1.90E-06	6.28E-05
837	ENSG00000228305.2	AC016734.1	-2.43	1.98E-06	6.54E-05
838	ENSG00000138698.14	RAP1GDS1	0.69	2.00E-06	6.57E-05
839	ENSG00000156218.12	ADAMTSL3	0.47	2.04E-06	6.70E-05
840	ENSG00000089063.14	TMEM230	0.48	2.05E-06	6.72E-05
841	ENSG00000183160.8	TMEM119	-1.20	2.08E-06	6.83E-05
842	ENSG00000147883.10	CDKN2B	-2.10	2.12E-06	6.95E-05
843	ENSG00000121022.13	COPS5	0.48	2.14E-06	7.00E-05
844	ENSG00000008441.16	NFIX	-0.53	2.14E-06	7.01E-05
845	ENSG00000161981.10	SNRNP25	1.08	2.16E-06	7.04E-05
846	ENSG00000131844.15	MCCC2	0.56	2.21E-06	7.19E-05
847	ENSG00000166938.12	DIS3L	0.47	2.21E-06	7.19E-05

848	ENSG00000166482.11	MFAP4	-0.93	2.28E-06	7.41E-05
849	ENSG00000076351.12	SLC46A1	0.77	2.31E-06	7.51E-05
850	ENSG00000034713.7	GABARAPL2	0.47	2.37E-06	7.67E-05
851	ENSG00000125741.4	OPA3	0.67	2.44E-06	7.92E-05
852	ENSG00000116729.13	WLS	-0.62	2.45E-06	7.94E-05
853	ENSG00000143951.15	WDPCP	-0.57	2.51E-06	8.12E-05
854	ENSG00000174697.4	LEP	-1.72	2.53E-06	8.19E-05
855	ENSG00000172260.14	NEGR1	-0.53	2.58E-06	8.30E-05
856	ENSG00000122707.11	RECK	-0.92	2.58E-06	8.30E-05
857	ENSG00000143387.12	CTSK	-1.27	2.58E-06	8.30E-05
858	ENSG00000253276.2	CCDC71L	-0.81	2.60E-06	8.37E-05
859	ENSG00000145779.7	TNFAIP8	-0.66	2.67E-06	8.58E-05
860	ENSG00000115364.13	MRPL19	0.56	2.68E-06	8.59E-05
861	ENSG00000168291.12	PDHB	0.94	2.71E-06	8.69E-05
862	ENSG00000214290.8	COLCA2	-1.77	2.72E-06	8.69E-05
863	ENSG00000149547.14	EI24	0.45	2.77E-06	8.86E-05
864	ENSG00000116117.17	PARD3B	-0.68	2.81E-06	8.97E-05
865	ENSG00000257178.5	AC103702.1	-0.85	2.83E-06	9.01E-05
866	ENSG00000072506.12	HSD17B10	0.80	2.87E-06	9.13E-05
867	ENSG00000106153.12	CHCHD2	0.61	2.89E-06	9.19E-05
868	ENSG00000103319.11	EEF2K	-0.59	2.92E-06	9.27E-05
869	ENSG00000143382.14	ADAMTSL4	-0.72	2.94E-06	9.32E-05
870	ENSG00000106624.10	AEBP1	-0.78	3.04E-06	9.64E-05
871	ENSG00000150347.15	ARID5B	-0.83	3.07E-06	9.73E-05
872	ENSG00000159403.16	C1R	-0.83	3.08E-06	9.75E-05
873	ENSG00000250137.1	AC093607.1	2.35	3.13E-06	9.90E-05
874	ENSG00000122176.11	FMOD	-0.82	3.19E-06	1.01E-04
875	ENSG00000147324.10	MFHAS1	1.04	3.20E-06	1.01E-04
876	ENSG00000151136.14	BTBD11	-2.26	3.23E-06	1.02E-04
877	ENSG00000171421.12	MRPL36	0.67	3.29E-06	1.04E-04
878	ENSG00000067177.14	PHKA1	0.81	3.34E-06	1.05E-04
879	ENSG00000125378.15	BMP4	-1.17	3.34E-06	1.05E-04
880	ENSG00000181856.14	SLC2A4	1.14	3.35E-06	1.05E-04
881	ENSG00000161813.21	LARP4	0.61	3.39E-06	1.06E-04
882	ENSG00000231312.6	AC007388.1	-0.83	3.44E-06	1.08E-04
883	ENSG00000157570.11	TSPAN18	0.61	3.56E-06	1.11E-04
884	ENSG00000101605.12	MYOM1	0.84	3.57E-06	1.11E-04
885	ENSG00000256540.1	AC007406.3	1.34	3.59E-06	1.12E-04
886	ENSG00000131389.17	SLC6A6	1.12	3.61E-06	1.12E-04
887	ENSG00000142156.14	COL6A1	-0.67	3.61E-06	1.12E-04
888	ENSG00000176438.12	SYNE3	-0.64	3.63E-06	1.13E-04
889	ENSG00000029993.14	HMGB3	1.16	3.70E-06	1.15E-04
890	ENSG00000064393.15	HIPK2	0.50	3.70E-06	1.15E-04
891	ENSG00000184602.5	SNN	0.67	3.72E-06	1.15E-04
892	ENSG00000069702.10	TGFBR3	-0.69	3.73E-06	1.15E-04
893	ENSG00000143369.14	ECM1	-0.98	3.75E-06	1.16E-04
894	ENSG00000162817.6	C1orf115	-0.74	3.87E-06	1.19E-04
895	ENSG00000270550.1	IGHV3-30	5.77	3.92E-06	1.21E-04
896	ENSG00000233608.3	TWIST2	-0.87	3.95E-06	1.22E-04
897	ENSG00000100033.16	PRODH	-1.12	3.95E-06	1.22E-04

898	ENSG00000197253.13	TPSB2	-1.69	4.03E-06	1.24E-04
899	ENSG00000143653.9	SCCPDH	0.78	4.07E-06	1.25E-04
900	ENSG00000158163.14	DZIP1L	-1.36	4.07E-06	1.25E-04
901	ENSG00000236333.3	TRHDE-AS1	-0.96	4.10E-06	1.26E-04
902	ENSG00000159884.11	CCDC107	-0.85	4.11E-06	1.26E-04
903	ENSG00000149534.8	MS4A2	-1.35	4.13E-06	1.26E-04
904	ENSG00000088543.14	C3orf18	0.91	4.16E-06	1.27E-04
905	ENSG00000145555.14	MYO10	0.78	4.21E-06	1.28E-04
906	ENSG00000163382.11	NAXE	0.85	4.26E-06	1.30E-04
907	ENSG00000185742.6	C11orf87	4.46	4.29E-06	1.30E-04
908	ENSG00000048052.21	HDAC9	-0.77	4.30E-06	1.31E-04
909	ENSG00000066405.12	CLDN18	-1.32	4.37E-06	1.32E-04
910	ENSG00000168702.17	LRP1B	1.87	4.44E-06	1.35E-04
911	ENSG00000099795.6	NDUFB7	0.80	4.49E-06	1.36E-04
912	ENSG00000182985.17	CADM1	-0.67	4.49E-06	1.36E-04
913	ENSG00000103647.12	CORO2B	-1.03	4.49E-06	1.36E-04
914	ENSG00000244227.7	LRRC77P	1.44	4.62E-06	1.39E-04
915	ENSG00000123243.14	ITIH5	-0.86	4.63E-06	1.40E-04
916	ENSG00000184845.3	DRD1	2.69	4.67E-06	1.41E-04
917	ENSG00000164307.12	ERAP1	0.59	4.68E-06	1.41E-04
918	ENSG00000171680.21	PLEKHG5	-1.07	4.74E-06	1.42E-04
919	ENSG00000247626.4	MARS2	1.32	4.76E-06	1.43E-04
920	ENSG00000185880.12	TRIM69	0.48	4.76E-06	1.43E-04
921	ENSG00000169894.17	MUC3A	1.58	4.83E-06	1.45E-04
922	ENSG00000134440.11	NARS	0.49	4.93E-06	1.47E-04
923	ENSG00000158828.7	PINK1	0.51	4.97E-06	1.48E-04
924	ENSG00000197375.12	SLC22A5	0.99	5.04E-06	1.51E-04
925	ENSG00000182621.17	PLCB1	0.70	5.18E-06	1.54E-04
926	ENSG00000132953.16	XPO4	0.46	5.18E-06	1.54E-04
927	ENSG00000154917.10	RAB6B	0.95	5.35E-06	1.59E-04
928	ENSG00000143374.16	TARS2	0.70	5.38E-06	1.60E-04
929	ENSG00000122490.18	PQLC1	-0.76	5.47E-06	1.63E-04
930	ENSG00000125037.12	EMC3	0.65	5.49E-06	1.63E-04
931	ENSG00000165775.17	FUNDC2	0.53	5.53E-06	1.64E-04
932	ENSG00000105607.12	GCDH	0.76	5.54E-06	1.64E-04
933	ENSG00000117593.10	DARS2	0.68	5.54E-06	1.64E-04
934	ENSG00000146729.9	NIPSNAP2	0.54	5.57E-06	1.64E-04
935	ENSG00000139112.10	GABARAPL1	0.62	5.60E-06	1.65E-04
936	ENSG00000112799.8	LY86	2.92	5.70E-06	1.68E-04
937	ENSG00000256340.8	ABCC6P1	1.61	5.89E-06	1.73E-04
938	ENSG00000205669.3	ACOT6	2.61	6.08E-06	1.79E-04
939	ENSG00000085721.12	RRN3	0.61	6.30E-06	1.85E-04
940	ENSG00000120068.6	HOXB8	-3.44	6.32E-06	1.86E-04
941	ENSG00000173366.11	AC097637.1	6.10	6.55E-06	1.92E-04
942	ENSG00000165646.13	SLC18A2	-1.86	6.62E-06	1.94E-04
943	ENSG00000137824.15	RMDN3	0.77	6.67E-06	1.95E-04
944	ENSG00000172340.14	SUCLG2	0.64	6.74E-06	1.97E-04
945	ENSG00000004866.20	ST7	0.72	6.76E-06	1.97E-04
946	ENSG00000152620.12	NADK2	0.46	6.84E-06	2.00E-04
947	ENSG00000160055.19	TMEM234	-0.58	6.88E-06	2.01E-04

948	ENSG00000211450.9	SELENOH	0.51	6.98E-06	2.03E-04
949	ENSG00000228204.2	AC004830.1	4.32	7.13E-06	2.07E-04
950	ENSG00000107295.9	SH3GL2	3.45	7.14E-06	2.07E-04
951	ENSG00000115355.16	CCDC88A	0.50	7.14E-06	2.07E-04
952	ENSG00000231806.3	PCAT7	2.00	7.16E-06	2.08E-04
953	ENSG00000121281.12	ADCY7	-0.83	7.53E-06	2.18E-04
954	ENSG00000130725.7	UBE2M	0.68	7.59E-06	2.19E-04
955	ENSG00000100347.14	SAMM50	0.61	7.67E-06	2.22E-04
956	ENSG00000248339.1	LINC02504	-1.38	7.68E-06	2.22E-04
957	ENSG00000280071.3	GATD3B	0.92	7.72E-06	2.23E-04
958	ENSG00000074071.14	MRPS34	0.81	7.76E-06	2.23E-04
959	ENSG00000127824.13	TUBA4A	1.47	7.78E-06	2.24E-04
960	ENSG00000182154.7	MRPL41	0.93	7.80E-06	2.24E-04
961	ENSG00000134504.13	KCTD1	1.13	8.03E-06	2.31E-04
962	ENSG00000002549.12	LAP3	0.70	8.08E-06	2.32E-04
963	ENSG00000185164.14	NOMO2	0.65	8.12E-06	2.33E-04
964	ENSG00000173517.10	PEAK1	-0.34	8.14E-06	2.33E-04
965	ENSG00000120093.11	HOXB3	-0.71	8.15E-06	2.33E-04
966	ENSG00000136235.16	GPNMB	-0.93	8.35E-06	2.38E-04
967	ENSG00000157404.15	KIT	-1.62	8.34E-06	2.38E-04
968	ENSG00000137714.2	FDX1	0.83	8.47E-06	2.41E-04
969	ENSG00000262413.1	AC145207.2	-5.21	8.58E-06	2.44E-04
970	ENSG00000064666.14	CNN2	-0.88	8.66E-06	2.46E-04
971	ENSG00000189398.5	OR7E12P	-1.51	8.68E-06	2.47E-04
972	ENSG00000169715.14	MT1E	1.86	8.85E-06	2.51E-04
973	ENSG00000071991.8	CDH19	1.35	8.87E-06	2.51E-04
974	ENSG00000189056.13	RELN	1.28	8.98E-06	2.54E-04
975	ENSG00000240409.1	MTATP8P1	2.68	9.05E-06	2.56E-04
976	ENSG00000156261.12	CCT8	0.44	9.12E-06	2.57E-04
977	ENSG00000163681.14	SLMAP	0.44	9.12E-06	2.57E-04
978	ENSG00000163815.5	CLEC3B	-0.72	9.11E-06	2.57E-04
979	ENSG00000159346.12	ADIPOR1	0.54	9.17E-06	2.58E-04
980	ENSG00000107957.16	SH3PXD2A	0.85	9.23E-06	2.60E-04
981	ENSG00000132563.15	REEP2	1.43	9.25E-06	2.60E-04
982	ENSG00000158062.20	UBXN11	-0.70	9.33E-06	2.62E-04
983	ENSG00000139292.12	LGR5	-3.21	9.58E-06	2.69E-04
984	ENSG00000198612.10	COPS8	0.61	9.65E-06	2.71E-04
985	ENSG00000144290.16	SLC4A10	2.13	9.77E-06	2.74E-04
986	ENSG00000074696.12	HACD3	0.51	9.86E-06	2.76E-04
987	ENSG00000132718.8	SYT11	0.53	9.89E-06	2.76E-04
988	ENSG00000119471.14	HSDL2	0.53	1.00E-05	2.80E-04
989	ENSG00000139793.18	MBNL2	0.54	1.00E-05	2.80E-04
990	ENSG00000183978.7	COA3	0.76	1.02E-05	2.84E-04
991	ENSG00000155760.2	FZD7	-1.04	1.06E-05	2.94E-04
992	ENSG00000171596.6	NMUR1	-1.41	1.06E-05	2.96E-04
993	ENSG00000176658.16	MYO1D	-0.68	1.07E-05	2.99E-04
994	ENSG00000176046.8	NUPR1	-0.57	1.08E-05	2.99E-04
995	ENSG00000165030.3	NFIL3	1.02	1.08E-05	3.00E-04
996	ENSG00000078804.12	TP53INP2	0.52	1.10E-05	3.04E-04
997	ENSG00000148082.9	SHC3	-1.79	1.10E-05	3.05E-04

998	ENSG00000223756.6	TSSC2	-0.64	1.10E-05	3.05E-04
999	ENSG00000154814.13	OXNAD1	0.51	1.12E-05	3.09E-04
1000	ENSG00000113575.9	PPP2CA	0.46	1.13E-05	3.12E-04
1001	ENSG00000101311.15	FERMT1	2.37	1.14E-05	3.13E-04
1002	ENSG00000248709.2	AC008549.1	1.36	1.14E-05	3.14E-04
1003	ENSG00000136270.13	TBRG4	0.67	1.14E-05	3.14E-04
1004	ENSG00000129515.18	SNX6	0.47	1.15E-05	3.16E-04
1005	ENSG00000184564.9	SLITRK6	1.82	1.16E-05	3.18E-04
1006	ENSG00000114850.6	SSR3	0.44	1.16E-05	3.18E-04
1007	ENSG00000169282.17	KCNAB1	0.59	1.16E-05	3.19E-04
1008	ENSG00000172349.17	IL16	-0.91	1.17E-05	3.19E-04
1009	ENSG00000230552.5	AC092162.2	-1.16	1.17E-05	3.20E-04
1010	ENSG00000110274.15	CEP164	-0.57	1.20E-05	3.27E-04
1011	ENSG00000155380.11	SLC16A1	0.80	1.20E-05	3.28E-04
1012	ENSG00000017427.16	IGF1	-1.06	1.20E-05	3.28E-04
1013	ENSG00000181991.15	MRPS11	0.68	1.22E-05	3.33E-04
1014	ENSG00000272235.1	AL590438.1	1.73	1.24E-05	3.37E-04
1015	ENSG00000162378.12	ZYG11B	0.47	1.25E-05	3.39E-04
1016	ENSG00000081985.11	IL12RB2	1.32	1.25E-05	3.39E-04
1017	ENSG00000233038.6	AC011899.2	2.95	1.31E-05	3.56E-04
1018	ENSG00000169314.14	C22orf15	2.58	1.33E-05	3.60E-04
1019	ENSG00000161634.11	DCD	-6.62	1.34E-05	3.64E-04
1020	ENSG00000162687.17	KCNT2	-0.97	1.35E-05	3.66E-04
1021	ENSG00000181830.8	SLC35C1	-0.84	1.37E-05	3.69E-04
1022	ENSG00000134248.13	LAMTOR5	0.63	1.37E-05	3.71E-04
1023	ENSG00000100181.22	TPTEP1	0.51	1.38E-05	3.71E-04
1024	ENSG00000128050.8	PAICS	0.44	1.38E-05	3.71E-04
1025	ENSG00000165644.10	COMTD1	2.29	1.38E-05	3.73E-04
1026	ENSG00000129255.15	MPDU1	0.69	1.39E-05	3.73E-04
1027	ENSG00000169760.17	NLGN1	-0.98	1.40E-05	3.75E-04
1028	ENSG00000239268.2	AC092691.1	-1.96	1.41E-05	3.79E-04
1029	ENSG00000166780.10	C16orf45	-1.02	1.42E-05	3.82E-04
1030	ENSG00000146830.10	GIGYF1	-0.56	1.44E-05	3.84E-04
1031	ENSG00000164128.6	NPY1R	-1.18	1.45E-05	3.87E-04
1032	ENSG00000198765.11	SYCP1	2.28	1.45E-05	3.88E-04
1033	ENSG00000075213.10	SEMA3A	-1.02	1.49E-05	3.97E-04
1034	ENSG00000047249.17	ATP6V1H	0.57	1.50E-05	4.00E-04
1035	ENSG00000171124.13	FUT3	1.03	1.51E-05	4.02E-04
1036	ENSG00000274827.4	LINC01297	0.70	1.53E-05	4.07E-04
1037	ENSG00000162733.17	DDR2	-0.68	1.53E-05	4.07E-04
1038	ENSG00000105355.8	PLIN3	-0.85	1.54E-05	4.09E-04
1039	ENSG00000171914.16	TLN2	-0.61	1.54E-05	4.10E-04
1040	ENSG00000181610.12	MRPS23	0.64	1.57E-05	4.17E-04
1041	ENSG00000126432.13	PRDX5	0.52	1.59E-05	4.20E-04
1042	ENSG00000152284.4	TCF7L1	-0.58	1.58E-05	4.20E-04
1043	ENSG00000138080.13	EMILIN1	-0.85	1.59E-05	4.20E-04
1044	ENSG00000154380.17	ENAH	0.43	1.61E-05	4.25E-04
1045	ENSG00000177694.15	NAALADL2	-0.47	1.61E-05	4.25E-04
1046	ENSG00000261408.7	TEN1-CDK3	-0.76	1.62E-05	4.26E-04
1047	ENSG00000215018.9	COL28A1	0.94	1.63E-05	4.29E-04

1048	ENSG00000118898.15	PPL	-1.54	1.63E-05	4.29E-04
1049	ENSG00000150779.11	TIMM8B	0.81	1.65E-05	4.32E-04
1050	ENSG00000198420.9	TCAF1	0.51	1.64E-05	4.32E-04
1051	ENSG00000198223.16	CSF2RA	-1.76	1.64E-05	4.32E-04
1052	ENSG00000196535.16	MYO18A	-0.57	1.65E-05	4.33E-04
1053	ENSG00000025039.14	RRAGD	0.60	1.66E-05	4.33E-04
1054	ENSG00000163931.15	TKT	0.60	1.67E-05	4.36E-04
1055	ENSG00000089847.12	ANKRD24	1.05	1.67E-05	4.37E-04
1056	ENSG00000163638.13	ADAMTS9	0.74	1.68E-05	4.40E-04
1057	ENSG00000225378.1	AC015977.2	5.59	1.76E-05	4.60E-04
1058	ENSG00000116288.12	PARK7	0.46	1.77E-05	4.60E-04
1059	ENSG00000274265.4	AC245297.3	-0.59	1.76E-05	4.60E-04
1060	ENSG00000166595.11	CIAO2B	0.61	1.79E-05	4.65E-04
1061	ENSG00000114331.13	ACAP2	0.49	1.81E-05	4.71E-04
1062	ENSG00000108559.11	NUP88	0.47	1.82E-05	4.72E-04
1063	ENSG00000224525.2	AL591686.1	2.26	1.82E-05	4.73E-04
1064	ENSG00000130222.10	GADD45G	3.85	1.84E-05	4.75E-04
1065	ENSG00000131269.16	ABCB7	0.48	1.85E-05	4.80E-04
1066	ENSG00000185608.8	MRPL40	0.75	1.88E-05	4.85E-04
1067	ENSG00000144029.11	MRPS5	0.52	1.88E-05	4.87E-04
1068	ENSG00000105197.10	TIMM50	0.48	1.90E-05	4.91E-04
1069	ENSG00000154518.9	ATP5MC3	0.55	1.91E-05	4.93E-04
1070	ENSG00000152785.6	BMP3	-2.91	1.93E-05	4.96E-04
1071	ENSG00000255154.7	HTD2	0.82	1.94E-05	4.99E-04
1072	ENSG00000101489.19	CELF4	1.02	1.94E-05	4.99E-04
1073	ENSG00000088832.16	FKBP1A	0.41	1.94E-05	4.99E-04
1074	ENSG00000253139.1	AC013644.1	-2.76	1.95E-05	5.01E-04
1075	ENSG00000278766.2	AC092683.2	-1.00	1.96E-05	5.02E-04
1076	ENSG00000116132.11	PRRX1	-0.68	1.98E-05	5.06E-04
1077	ENSG00000102738.7	MRPS31	0.61	1.98E-05	5.07E-04
1078	ENSG00000164946.19	FREM1	-0.88	2.06E-05	5.28E-04
1079	ENSG00000118971.7	CCND2	-1.00	2.07E-05	5.28E-04
1080	ENSG00000116096.5	SPR	0.91	2.08E-05	5.30E-04
1081	ENSG00000168477.19	TNXB	-0.82	2.08E-05	5.30E-04
1082	ENSG00000167799.9	NUDT8	1.49	2.12E-05	5.39E-04
1083	ENSG00000129474.15	AJUBA	-0.99	2.16E-05	5.51E-04
1084	ENSG00000130595.18	TNNT3	2.33	2.18E-05	5.54E-04
1085	ENSG00000116711.9	PLA2G4A	0.99	2.21E-05	5.60E-04
1086	ENSG00000188690.13	UROS	0.45	2.22E-05	5.64E-04
1087	ENSG00000135002.11	RFK	0.59	2.26E-05	5.74E-04
1088	ENSG00000138131.3	LOXL4	-1.16	2.27E-05	5.75E-04
1089	ENSG00000157184.6	CPT2	0.68	2.29E-05	5.79E-04
1090	ENSG00000087077.13	TRIP6	-0.65	2.31E-05	5.85E-04
1091	ENSG00000240303.7	ACAD11	0.49	2.33E-05	5.89E-04
1092	ENSG00000156502.13	SUPV3L1	0.71	2.34E-05	5.90E-04
1093	ENSG00000148730.6	EIF4EBP2	0.52	2.34E-05	5.91E-04
1094	ENSG00000249035.6	CLMAT3	-0.85	2.37E-05	5.96E-04
1095	ENSG00000170381.13	SEMA3E	-1.39	2.37E-05	5.96E-04
1096	ENSG00000154997.8	SEPT14	0.76	2.40E-05	6.04E-04
1097	ENSG00000233766.7	AC098617.1	1.82	2.42E-05	6.07E-04

1098	ENSG00000164237.8	CMBL	0.76	2.44E-05	6.12E-04
1099	ENSG00000006007.11	GDE1	0.67	2.48E-05	6.22E-04
1100	ENSG00000124275.14	MTRR	0.44	2.51E-05	6.30E-04
1101	ENSG00000140092.14	FBLN5	-0.62	2.52E-05	6.31E-04
1102	ENSG00000013306.15	SLC25A39	0.69	2.54E-05	6.36E-04
1103	ENSG00000049759.17	NEDD4L	-0.94	2.55E-05	6.37E-04
1104	ENSG00000116221.15	MRPL37	0.60	2.56E-05	6.39E-04
1105	ENSG00000254632.1	AP003119.1	4.50	2.56E-05	6.39E-04
1106	ENSG00000251432.6	AC108062.1	0.82	2.58E-05	6.42E-04
1107	ENSG00000128567.16	PODXL	0.52	2.58E-05	6.43E-04
1108	ENSG00000168807.16	SNTB2	-0.62	2.59E-05	6.44E-04
1109	ENSG00000234261.3	AL138720.1	2.30	2.60E-05	6.45E-04
1110	ENSG00000085662.13	AKR1B1	0.53	2.61E-05	6.47E-04
1111	ENSG00000163083.5	INHBB	-1.82	2.61E-05	6.48E-04
1112	ENSG00000263155.5	MYZAP	-0.60	2.61E-05	6.48E-04
1113	ENSG00000089157.15	RPLP0	0.57	2.62E-05	6.49E-04
1114	ENSG00000235292.1	AL109838.1	3.52	2.62E-05	6.49E-04
1115	ENSG00000122644.12	ARL4A	0.66	2.66E-05	6.56E-04
1116	ENSG00000182158.14	CREB3L2	-0.57	2.66E-05	6.57E-04
1117	ENSG00000225096.2	AL445250.1	0.84	2.68E-05	6.60E-04
1118	ENSG00000135678.11	CPM	-0.54	2.68E-05	6.60E-04
1119	ENSG00000169435.13	RASSF6	1.31	2.72E-05	6.70E-04
1120	ENSG00000197728.11	RPS26	0.65	2.72E-05	6.70E-04
1121	ENSG00000266074.8	BAHCC1	-0.59	2.75E-05	6.77E-04
1122	ENSG00000144642.21	RBMS3	-0.39	2.82E-05	6.93E-04
1123	ENSG00000165669.13	FAM204A	-0.48	2.84E-05	6.96E-04
1124	ENSG00000204564.11	C6orf136	0.80	2.85E-05	6.99E-04
1125	ENSG00000138600.9	SPPL2A	0.44	2.88E-05	7.05E-04
1126	ENSG00000109743.10	BST1	-0.84	2.88E-05	7.05E-04
1127	ENSG00000214944.9	ARHGEF28	-0.62	2.92E-05	7.13E-04
1128	ENSG00000151892.14	GFRA1	-1.06	2.92E-05	7.13E-04
1129	ENSG00000175198.16	PCCA	0.58	2.93E-05	7.16E-04
1130	ENSG00000090621.13	PABPC4	0.50	2.94E-05	7.17E-04
1131	ENSG00000235033.7	AL590999.1	1.12	2.95E-05	7.19E-04
1132	ENSG00000164088.17	PPM1M	-0.54	2.96E-05	7.22E-04
1133	ENSG00000205189.11	ZBTB10	0.36	3.02E-05	7.34E-04
1134	ENSG00000185000.11	DGAT1	0.66	3.02E-05	7.34E-04
1135	ENSG00000125459.15	MSTO1	1.05	3.03E-05	7.37E-04
1136	ENSG00000272079.2	AC004233.3	2.56	3.06E-05	7.42E-04
1137	ENSG00000150054.18	MPP7	0.85	3.06E-05	7.42E-04
1138	ENSG00000232931.5	LINC00342	-0.61	3.08E-05	7.46E-04
1139	ENSG00000187193.8	MT1X	1.09	3.09E-05	7.48E-04
1140	ENSG00000167880.7	EVPL	-3.27	3.14E-05	7.59E-04
1141	ENSG00000184956.15	MUC6	-0.79	3.16E-05	7.64E-04
1142	ENSG00000144908.13	ALDH1L1	0.78	3.18E-05	7.67E-04
1143	ENSG00000164172.18	MOCS2	0.47	3.19E-05	7.70E-04
1144	ENSG00000197858.10	GPAA1	0.60	3.24E-05	7.81E-04
1145	ENSG00000230630.5	DNM3OS	-0.55	3.24E-05	7.81E-04
1146	ENSG00000136630.12	HLX	0.75	3.26E-05	7.83E-04
1147	ENSG00000104765.15	BNIP3L	-0.43	3.28E-05	7.89E-04

1148	ENSG00000060762.18	MPC1	1.09	3.32E-05	7.96E-04
1149	ENSG00000147155.10	EBP	0.98	3.35E-05	8.03E-04
1150	ENSG00000118407.14	FILIP1	0.72	3.35E-05	8.03E-04
1151	ENSG00000103855.17	CD276	-0.69	3.35E-05	8.03E-04
1152	ENSG00000168268.10	NT5DC2	1.02	3.37E-05	8.06E-04
1153	ENSG00000134046.11	MBD2	-0.55	3.40E-05	8.13E-04
1154	ENSG00000102743.14	SLC25A15	1.09	3.43E-05	8.18E-04
1155	ENSG00000151148.13	UBE3B	0.36	3.45E-05	8.23E-04
1156	ENSG00000140848.16	CPNE2	-0.55	3.49E-05	8.33E-04
1157	ENSG00000134250.19	NOTCH2	-0.55	3.52E-05	8.39E-04
1158	ENSG00000130560.8	UBAC1	0.61	3.54E-05	8.42E-04
1159	ENSG00000168497.4	CAVIN2	0.52	3.59E-05	8.54E-04
1160	ENSG00000169515.6	CCDC8	-0.86	3.70E-05	8.79E-04
1161	ENSG00000163762.6	TM4SF18	0.66	3.72E-05	8.84E-04
1162	ENSG00000133401.15	PDZD2	-0.48	3.80E-05	9.02E-04
1163	ENSG00000144229.11	THSD7B	0.67	3.82E-05	9.05E-04
1164	ENSG00000161202.18	DVL3	-0.44	3.84E-05	9.09E-04
1165	ENSG00000157379.13	DHRS1	-0.54	3.89E-05	9.19E-04
1166	ENSG00000101224.17	CDC25B	-0.67	3.88E-05	9.19E-04
1167	ENSG00000118156.12	ZNF541	-1.59	3.89E-05	9.20E-04
1168	ENSG00000145685.13	LHFPL2	0.65	3.91E-05	9.22E-04
1169	ENSG00000187097.12	ENTPD5	0.52	3.94E-05	9.29E-04
1170	ENSG00000103494.13	RPGRIP1L	-0.83	3.98E-05	9.37E-04
1171	ENSG00000119508.17	NR4A3	1.66	3.99E-05	9.38E-04
1172	ENSG00000170270.4	GON7	0.89	4.06E-05	9.53E-04
1173	ENSG00000152977.9	ZIC1	-2.74	4.06E-05	9.53E-04
1174	ENSG00000229221.1	HNRNPA1P66	-2.35	4.13E-05	9.70E-04
1175	ENSG00000164530.14	PI16	-1.20	4.17E-05	9.77E-04
1176	ENSG00000173457.10	PPP1R14B	1.11	4.21E-05	9.85E-04
1177	ENSG00000095209.11	TMEM38B	0.88	4.21E-05	9.85E-04
1178	ENSG00000172057.9	ORMDL3	-0.86	4.21E-05	9.85E-04
1179	ENSG00000187098.15	MITF	-0.74	4.22E-05	9.86E-04
1180	ENSG00000166402.8	TUB	-0.72	4.24E-05	9.90E-04
1181	ENSG00000168653.10	NDUFS5	0.68	4.30E-05	1.00E-03
1182	ENSG00000165730.15	STOX1	-1.53	4.31E-05	1.01E-03
1183	ENSG00000030304.13	MUSK	-1.92	4.37E-05	1.02E-03
1184	ENSG00000136371.10	MTHFS	0.87	4.40E-05	1.02E-03
1185	ENSG00000119938.8	PPP1R3C	1.78	4.45E-05	1.04E-03
1186	ENSG00000103226.17	NOMO3	0.82	4.46E-05	1.04E-03
1187	ENSG00000163430.11	FSTL1	-0.53	4.48E-05	1.04E-03
1188	ENSG00000106993.11	CDC37L1	-0.71	4.49E-05	1.04E-03
1189	ENSG00000106245.10	BUD31	0.58	4.52E-05	1.05E-03
1190	ENSG00000213214.4	ARHGEF35	-0.66	4.55E-05	1.06E-03
1191	ENSG00000188269.9	OR7A5	-0.99	4.57E-05	1.06E-03
1192	ENSG00000177119.15	ANO6	0.54	4.57E-05	1.06E-03
1193	ENSG00000106333.12	PCOLCE	-0.71	4.64E-05	1.07E-03
1194	ENSG00000241685.9	ARPC1A	0.55	4.67E-05	1.08E-03
1195	ENSG00000221866.9	PLXNA4	-0.59	4.71E-05	1.09E-03
1196	ENSG00000114686.8	MRPL3	0.46	4.73E-05	1.09E-03
1197	ENSG00000165197.4	VEGFD	1.37	4.74E-05	1.09E-03

1198	ENSG00000141447.17	OSBPL1A	0.37	4.76E-05	1.10E-03
1199	ENSG00000276490.1	AL583836.1	3.89	4.78E-05	1.10E-03
1200	ENSG00000127838.13	PNKD	0.63	4.91E-05	1.13E-03
1201	ENSG00000148795.6	CYP17A1	1.48	4.98E-05	1.14E-03
1202	ENSG00000112186.11	CAP2	-0.86	4.98E-05	1.14E-03
1203	ENSG00000108576.9	SLC6A4	1.84	5.06E-05	1.16E-03
1204	ENSG00000204580.13	DDR1	-1.32	5.09E-05	1.17E-03
1205	ENSG00000104327.7	CALB1	0.89	5.11E-05	1.17E-03
1206	ENSG00000169288.17	MRPL1	0.66	5.17E-05	1.18E-03
1207	ENSG00000100418.7	DESI1	0.54	5.17E-05	1.18E-03
1208	ENSG00000276997.4	AL513314.2	0.87	5.18E-05	1.18E-03
1209	ENSG00000115419.12	GLS	-0.55	5.20E-05	1.19E-03
1210	ENSG00000171385.9	KCND3	0.84	5.22E-05	1.19E-03
1211	ENSG00000140009.18	ESR2	-0.72	5.27E-05	1.20E-03
1212	ENSG00000171444.17	MCC	0.34	5.29E-05	1.20E-03
1213	ENSG00000151276.23	MAGI1	-0.54	5.28E-05	1.20E-03
1214	ENSG00000277797.1	AL359693.1	-3.93	5.28E-05	1.20E-03
1215	ENSG00000118729.11	CASQ2	0.94	5.30E-05	1.20E-03
1216	ENSG00000154767.14	XPC	-0.43	5.35E-05	1.21E-03
1217	ENSG00000175879.8	HOXD8	-0.67	5.36E-05	1.21E-03
1218	ENSG00000162461.7	SLC25A34	1.25	5.41E-05	1.22E-03
1219	ENSG00000103121.8	CMC2	0.55	5.41E-05	1.22E-03
1220	ENSG00000166831.8	RBPMS2	0.80	5.45E-05	1.23E-03
1221	ENSG00000245060.6	LINC00847	0.93	5.58E-05	1.26E-03
1222	ENSG00000186469.8	GNG2	-0.96	5.62E-05	1.27E-03
1223	ENSG00000116171.17	SCP2	0.52	5.65E-05	1.27E-03
1224	ENSG00000203485.12	INF2	-0.56	5.67E-05	1.28E-03
1225	ENSG00000121897.14	LIAS	0.54	5.69E-05	1.28E-03
1226	ENSG00000172164.14	SNTB1	0.54	5.70E-05	1.28E-03
1227	ENSG00000186377.7	CYP4X1	-0.92	5.79E-05	1.30E-03
1228	ENSG00000134755.15	DSC2	-0.99	5.80E-05	1.30E-03
1229	ENSG00000086666.18	ZFAND6	0.54	5.83E-05	1.31E-03
1230	ENSG00000211598.2	IGKV4-1	4.73	5.88E-05	1.32E-03
1231	ENSG00000128833.12	MYO5C	-0.66	5.89E-05	1.32E-03
1232	ENSG00000105245.9	NUMBL	-0.57	6.01E-05	1.34E-03
1233	ENSG00000135111.15	TBX3	0.68	6.07E-05	1.36E-03
1234	ENSG00000122696.13	SLC25A51	0.46	6.08E-05	1.36E-03
1235	ENSG00000137693.13	YAP1	-0.52	6.07E-05	1.36E-03
1236	ENSG00000184292.6	TACSTD2	-4.89	6.10E-05	1.36E-03
1237	ENSG00000229515.1	FLT1P1	5.43	6.22E-05	1.39E-03
1238	ENSG00000102755.11	FLT1	0.47	6.24E-05	1.39E-03
1239	ENSG00000163145.12	C1QTNF7	-1.00	6.26E-05	1.39E-03
1240	ENSG00000170396.7	ZNF804A	5.15	6.27E-05	1.40E-03
1241	ENSG00000147576.16	ADHFE1	0.62	6.30E-05	1.40E-03
1242	ENSG00000144909.7	OSBPL11	0.90	6.33E-05	1.41E-03
1243	ENSG00000135094.10	SDS	1.38	6.41E-05	1.42E-03
1244	ENSG00000110660.14	SLC35F2	-1.07	6.46E-05	1.43E-03
1245	ENSG00000220884.2	MESTP1	-1.95	6.46E-05	1.43E-03
1246	ENSG00000149084.12	HSD17B12	0.88	6.49E-05	1.44E-03
1247	ENSG00000152518.7	ZFP36L2	-0.74	6.50E-05	1.44E-03

1248	ENSG00000066468.22	FGFR2	-0.92	6.58E-05	1.45E-03
1249	ENSG00000069188.16	SDK2	1.47	6.66E-05	1.47E-03
1250	ENSG00000066136.20	NFYC	0.58	6.71E-05	1.48E-03
1251	ENSG00000171033.12	PKIA	1.02	6.72E-05	1.48E-03
1252	ENSG00000146425.10	DYNLT1	0.44	6.74E-05	1.48E-03
1253	ENSG00000143774.16	GUK1	0.65	6.82E-05	1.50E-03
1254	ENSG00000136840.18	ST6GALNAC4	1.25	6.84E-05	1.50E-03
1255	ENSG00000165092.12	ALDH1A1	-0.83	6.86E-05	1.51E-03
1256	ENSG00000142303.13	ADAMTS10	-0.92	6.86E-05	1.51E-03
1257	ENSG00000138385.15	SSB	-0.46	6.87E-05	1.51E-03
1258	ENSG00000166833.20	NAV2	-0.71	6.93E-05	1.52E-03
1259	ENSG00000231607.10	DLEU2	0.64	6.96E-05	1.52E-03
1260	ENSG00000175550.7	DRAP1	0.49	6.96E-05	1.52E-03
1261	ENSG00000168453.14	HR	2.33	6.99E-05	1.53E-03
1262	ENSG00000177663.13	IL17RA	-0.52	6.99E-05	1.53E-03
1263	ENSG00000147606.8	SLC26A7	1.28	7.00E-05	1.53E-03
1264	ENSG00000126934.13	MAP2K2	0.47	7.05E-05	1.54E-03
1265	ENSG00000115380.19	EFEMP1	-0.54	7.12E-05	1.55E-03
1266	ENSG00000112773.15	TENT5A	-0.62	7.16E-05	1.56E-03
1267	ENSG00000010319.6	SEMA3G	-0.64	7.17E-05	1.56E-03
1268	ENSG00000122547.10	EEPD1	-0.82	7.19E-05	1.56E-03
1269	ENSG00000144848.10	ATG3	0.51	7.21E-05	1.57E-03
1270	ENSG00000166888.11	STAT6	-0.43	7.21E-05	1.57E-03
1271	ENSG00000109180.14	OCIAD1	0.41	7.26E-05	1.57E-03
1272	ENSG00000123384.13	LRP1	-0.62	7.26E-05	1.57E-03
1273	ENSG00000154447.14	SH3RF1	-0.59	7.28E-05	1.58E-03
1274	ENSG00000044524.10	EPHA3	0.67	7.29E-05	1.58E-03
1275	ENSG00000105379.9	ETFB	0.71	7.34E-05	1.59E-03
1276	ENSG00000167216.16	KATNAL2	0.81	7.36E-05	1.59E-03
1277	ENSG00000107863.17	ARHGAP21	-0.47	7.42E-05	1.60E-03
1278	ENSG00000133943.20	DGLUCY	0.56	7.47E-05	1.61E-03
1279	ENSG00000068697.6	LAPTM4A	-0.43	7.51E-05	1.62E-03
1280	ENSG00000112855.15	HARS2	0.52	7.54E-05	1.62E-03
1281	ENSG00000215483.10	LINC00598	0.79	7.59E-05	1.63E-03
1282	ENSG00000172428.10	COPS9	0.61	7.70E-05	1.66E-03
1283	ENSG00000154719.13	MRPL39	0.57	7.70E-05	1.66E-03
1284	ENSG00000166165.12	CKB	1.43	7.72E-05	1.66E-03
1285	ENSG00000106571.13	GLI3	-0.74	7.75E-05	1.66E-03
1286	ENSG00000163873.9	GRIK3	1.30	7.81E-05	1.68E-03
1287	ENSG00000158604.14	TMED4	0.52	7.84E-05	1.68E-03
1288	ENSG00000149792.8	MRPL49	0.51	7.85E-05	1.68E-03
1289	ENSG00000034510.5	TMSB10	-0.47	7.98E-05	1.71E-03
1290	ENSG00000173614.13	NMNAT1	0.75	8.01E-05	1.71E-03
1291	ENSG00000140538.16	NTRK3	0.90	8.04E-05	1.72E-03
1292	ENSG00000160439.15	RDH13	0.75	8.07E-05	1.72E-03
1293	ENSG00000100593.17	ISM2	5.03	8.19E-05	1.75E-03
1294	ENSG00000229905.1	AL669831.2	1.01	8.19E-05	1.75E-03
1295	ENSG00000169231.13	THBS3	-0.50	8.24E-05	1.75E-03
1296	ENSG00000099622.13	CIRBP	-0.35	8.30E-05	1.77E-03
1297	ENSG00000115648.13	MLPH	-0.79	8.35E-05	1.78E-03

1298	ENSG00000172572.6	PDE3A	0.61	8.41E-05	1.79E-03
1299	ENSG00000105993.14	DNAJB6	0.40	8.42E-05	1.79E-03
1300	ENSG00000115762.16	PLEKHB2	0.56	8.49E-05	1.80E-03
1301	ENSG00000189221.9	MAOA	0.53	8.62E-05	1.83E-03
1302	ENSG00000090263.15	MRPS33	0.75	8.70E-05	1.84E-03
1303	ENSG00000138336.8	TET1	-0.63	8.69E-05	1.84E-03
1304	ENSG00000254132.1	MTND6P3	1.41	8.81E-05	1.86E-03
1305	ENSG00000171055.14	FEZ2	-0.40	8.91E-05	1.88E-03
1306	ENSG00000089693.10	MLF2	0.37	9.00E-05	1.90E-03
1307	ENSG00000205339.9	IPO7	0.38	9.01E-05	1.90E-03
1308	ENSG00000158683.8	PKD1L1	1.12	9.04E-05	1.91E-03
1309	ENSG00000125863.19	MKKS	0.52	9.08E-05	1.91E-03
1310	ENSG00000057294.14	PKP2	-1.41	9.10E-05	1.91E-03
1311	ENSG00000138035.14	PNPT1	0.45	9.15E-05	1.92E-03
1312	ENSG00000242866.10	STRC	0.99	9.23E-05	1.94E-03
1313	ENSG00000099998.17	GGT5	-0.77	9.23E-05	1.94E-03
1314	ENSG00000068120.14	COASY	0.63	9.24E-05	1.94E-03
1315	ENSG00000136040.8	PLXNC1	0.88	9.28E-05	1.94E-03
1316	ENSG00000272168.6	CASC15	0.73	9.29E-05	1.95E-03
1317	ENSG00000206344.7	HCG27	-0.89	9.38E-05	1.96E-03
1318	ENSG00000155463.12	OXA1L	0.45	9.40E-05	1.97E-03
1319	ENSG00000196932.11	TMEM26	2.83	9.44E-05	1.97E-03
1320	ENSG00000149716.12	LTO1	-1.05	9.54E-05	1.99E-03
1321	ENSG00000103546.18	SLC6A2	-1.18	9.55E-05	1.99E-03
1322	ENSG00000168528.11	SERINC2	1.43	9.65E-05	2.01E-03
1323	ENSG00000114923.16	SLC4A3	2.32	9.69E-05	2.02E-03
1324	ENSG00000064309.14	CDON	-0.59	9.82E-05	2.04E-03
1325	ENSG00000205885.7	C1RL-AS1	-0.87	9.81E-05	2.04E-03
1326	ENSG00000196262.13	PPIA	0.46	9.83E-05	2.04E-03
1327	ENSG00000231512.6	SEPT14P21	3.58	9.88E-05	2.05E-03
1328	ENSG00000099250.17	NRP1	0.51	9.88E-05	2.05E-03
1329	ENSG00000198722.14	UNC13B	0.35	1.00E-04	2.08E-03
1330	ENSG00000198944.5	SOWAHA	-2.06	1.00E-04	2.08E-03
1331	ENSG00000174099.11	MSRB3	-0.55	1.01E-04	2.09E-03
1332	ENSG00000152154.10	TMEM178A	-1.56	1.02E-04	2.10E-03
1333	ENSG00000107819.13	SFXN3	-0.53	1.02E-04	2.11E-03
1334	ENSG00000224549.1	AC017067.1	-2.07	1.02E-04	2.11E-03
1335	ENSG00000006652.13	IFRD1	0.99	1.03E-04	2.12E-03
1336	ENSG00000078269.14	SYNJ2	-0.89	1.03E-04	2.12E-03
1337	ENSG00000073605.18	GSDMB	-1.03	1.04E-04	2.14E-03
1338	ENSG00000117528.12	ABCD3	0.39	1.06E-04	2.19E-03
1339	ENSG00000186081.11	KRT5	-5.44	1.06E-04	2.19E-03
1340	ENSG00000235743.1	AL139231.1	3.14	1.06E-04	2.19E-03
1341	ENSG00000163328.13	GPR155	0.56	1.07E-04	2.20E-03
1342	ENSG00000253495.1	CYCSP23	-4.54	1.07E-04	2.20E-03
1343	ENSG00000176635.17	HORMAD2	4.37	1.09E-04	2.23E-03
1344	ENSG00000170323.8	FABP4	0.50	1.09E-04	2.23E-03
1345	ENSG00000163359.15	COL6A3	-0.62	1.09E-04	2.23E-03
1346	ENSG00000079308.18	TNS1	-0.60	1.09E-04	2.24E-03
1347	ENSG00000214736.7	TOMM6	0.46	1.10E-04	2.24E-03

1348	ENSG00000269028.3	MTRNR2L12	1.26	1.11E-04	2.27E-03
1349	ENSG00000272129.1	AL359715.3	1.01	1.12E-04	2.28E-03
1350	ENSG00000136159.3	NUDT15	0.56	1.12E-04	2.30E-03
1351	ENSG00000107897.18	ACBD5	0.53	1.13E-04	2.31E-03
1352	ENSG00000127720.7	METTL25	0.70	1.13E-04	2.31E-03
1353	ENSG00000123297.17	TSFM	0.75	1.14E-04	2.32E-03
1354	ENSG00000185787.14	MORF4L1	-0.39	1.14E-04	2.32E-03
1355	ENSG00000240045.1	DWORF	1.78	1.14E-04	2.32E-03
1356	ENSG00000183018.8	SPNS2	0.84	1.14E-04	2.32E-03
1357	ENSG00000246363.2	LINC02458	2.94	1.15E-04	2.33E-03
1358	ENSG00000133195.11	SLC39A11	-0.71	1.15E-04	2.33E-03
1359	ENSG00000146411.5	SLC2A12	1.60	1.15E-04	2.34E-03
1360	ENSG00000134153.9	EMC7	0.52	1.15E-04	2.34E-03
1361	ENSG00000183837.9	PNMA3	-2.24	1.15E-04	2.34E-03
1362	ENSG00000108021.20	FAM208B	0.43	1.16E-04	2.35E-03
1363	ENSG00000233429.9	HOTAIRM1	0.78	1.17E-04	2.37E-03
1364	ENSG00000149761.8	NUDT22	0.74	1.18E-04	2.39E-03
1365	ENSG00000115361.7	ACADL	0.55	1.18E-04	2.39E-03
1366	ENSG00000198513.11	ATL1	-0.76	1.18E-04	2.39E-03
1367	ENSG00000068305.17	MEF2A	0.33	1.19E-04	2.40E-03
1368	ENSG00000107960.10	STN1	0.65	1.20E-04	2.42E-03
1369	ENSG00000165688.11	PMPCA	0.76	1.20E-04	2.42E-03
1370	ENSG00000186094.16	AGBL4	1.06	1.20E-04	2.42E-03
1371	ENSG00000167779.8	IGFBP6	-0.76	1.21E-04	2.43E-03
1372	ENSG00000109089.7	CDR2L	-0.90	1.21E-04	2.43E-03
1373	ENSG00000011201.11	ANOS1	-0.56	1.23E-04	2.47E-03
1374	ENSG00000146938.15	NLGN4X	0.76	1.24E-04	2.49E-03
1375	ENSG00000167969.12	ECI1	0.94	1.25E-04	2.50E-03
1376	ENSG00000125730.16	C3	-0.68	1.25E-04	2.51E-03
1377	ENSG00000119705.9	SLIRP	0.60	1.26E-04	2.52E-03
1378	ENSG00000133065.10	SLC41A1	0.55	1.27E-04	2.53E-03
1379	ENSG00000164708.5	PGAM2	2.56	1.27E-04	2.55E-03
1380	ENSG00000148841.16	ITPRIP	0.86	1.29E-04	2.58E-03
1381	ENSG00000164494.11	PDSS2	0.68	1.30E-04	2.60E-03
1382	ENSG00000115738.9	ID2	0.95	1.33E-04	2.65E-03
1383	ENSG00000164687.10	FABP5	0.88	1.33E-04	2.65E-03
1384	ENSG00000138442.9	WDR12	0.50	1.33E-04	2.65E-03
1385	ENSG00000114115.9	RBP1	1.27	1.35E-04	2.68E-03
1386	ENSG00000071205.11	ARHGAP10	-0.44	1.35E-04	2.68E-03
1387	ENSG00000139410.14	SDSL	1.56	1.36E-04	2.71E-03
1388	ENSG00000084733.10	RAB10	0.31	1.36E-04	2.71E-03
1389	ENSG00000141026.5	MED9	0.55	1.37E-04	2.72E-03
1390	ENSG00000255690.2	TRIL	0.74	1.37E-04	2.72E-03
1391	ENSG00000103966.10	EHD4	-0.50	1.43E-04	2.83E-03
1392	ENSG00000105819.13	PMPCB	0.52	1.43E-04	2.84E-03
1393	ENSG00000104763.18	ASAH1	0.80	1.44E-04	2.85E-03
1394	ENSG00000083720.12	OXCT1	0.57	1.44E-04	2.85E-03
1395	ENSG00000169599.12	NFU1	0.50	1.45E-04	2.86E-03
1396	ENSG00000163684.11	RPP14	0.44	1.46E-04	2.87E-03
1397	ENSG00000152208.12	GRID2	-4.47	1.46E-04	2.88E-03

1398	ENSG00000031081.10	ARHGAP31	0.46	1.47E-04	2.90E-03
1399	ENSG00000170955.9	CAVIN3	-0.73	1.47E-04	2.90E-03
1400	ENSG00000110871.14	COQ5	0.50	1.47E-04	2.90E-03
1401	ENSG00000125733.17	TRIP10	-0.79	1.47E-04	2.90E-03
1402	ENSG00000066926.10	FECH	0.65	1.48E-04	2.91E-03
1403	ENSG00000105492.15	SIGLEC6	-2.75	1.48E-04	2.92E-03
1404	ENSG00000169976.6	SF3B5	0.72	1.49E-04	2.93E-03
1405	ENSG00000277534.1	AC007996.1	1.27	1.50E-04	2.95E-03
1406	ENSG00000180660.7	MAB21L1	0.93	1.51E-04	2.96E-03
1407	ENSG00000109390.11	NDUFC1	0.84	1.52E-04	2.97E-03
1408	ENSG00000185215.8	TNFAIP2	-0.55	1.52E-04	2.97E-03
1409	ENSG00000135749.18	PCNX2	-0.72	1.53E-04	2.99E-03
1410	ENSG00000145431.10	PDGFC	-0.46	1.53E-04	2.99E-03
1411	ENSG00000108381.10	ASPA	0.47	1.53E-04	2.99E-03
1412	ENSG00000268658.5	LINC00664	1.37	1.54E-04	3.00E-03
1413	ENSG00000120254.15	MTHFD1L	-1.15	1.54E-04	3.00E-03
1414	ENSG00000131067.16	GGT7	-0.73	1.55E-04	3.03E-03
1415	ENSG00000167110.17	GOLGA2	-0.31	1.59E-04	3.11E-03
1416	ENSG00000100644.16	HIF1A	0.44	1.60E-04	3.11E-03
1417	ENSG00000275700.4	AATF	-0.35	1.61E-04	3.14E-03
1418	ENSG00000138399.17	FASTKD1	0.65	1.63E-04	3.16E-03
1419	ENSG00000154736.5	ADAMTS5	-0.69	1.63E-04	3.18E-03
1420	ENSG00000118961.14	LDAH	0.57	1.64E-04	3.18E-03
1421	ENSG00000164120.13	HPGD	-1.61	1.65E-04	3.19E-03
1422	ENSG00000100129.17	EIF3L	0.44	1.65E-04	3.19E-03
1423	ENSG00000270672.1	MTRNR2L6	1.25	1.66E-04	3.22E-03
1424	ENSG00000186106.11	ANKRD46	0.48	1.66E-04	3.22E-03
1425	ENSG00000230882.1	AC005077.4	3.01	1.67E-04	3.22E-03
1426	ENSG00000198736.11	MSRB1	0.81	1.67E-04	3.24E-03
1427	ENSG00000071967.11	CYBRD1	-0.41	1.69E-04	3.27E-03
1428	ENSG00000125304.9	TM9SF2	0.35	1.70E-04	3.29E-03
1429	ENSG00000152102.17	FAM168B	0.34	1.70E-04	3.29E-03
1430	ENSG00000218336.8	TENM3	-0.47	1.71E-04	3.30E-03
1431	ENSG00000182326.14	C1S	-0.56	1.71E-04	3.30E-03
1432	ENSG00000108821.13	COL1A1	-1.25	1.71E-04	3.30E-03
1433	ENSG00000124827.6	GCM2	-2.29	1.72E-04	3.30E-03
1434	ENSG00000278619.4	MRM1	0.99	1.72E-04	3.31E-03
1435	ENSG00000257337.6	AC068888.1	-0.59	1.73E-04	3.31E-03
1436	ENSG00000121753.12	ADGRB2	-1.57	1.73E-04	3.31E-03
1437	ENSG00000167165.18	UGT1A6	4.73	1.73E-04	3.32E-03
1438	ENSG00000206190.11	ATP10A	-1.34	1.75E-04	3.36E-03
1439	ENSG00000107864.14	CPEB3	0.78	1.76E-04	3.38E-03
1440	ENSG00000187994.13	RINL	0.84	1.80E-04	3.45E-03
1441	ENSG00000175806.14	MSRA	0.45	1.82E-04	3.48E-03
1442	ENSG00000013297.11	CLDN11	-1.22	1.86E-04	3.55E-03
1443	ENSG00000174917.8	C19orf70	0.67	1.86E-04	3.55E-03
1444	ENSG00000250742.2	LINC02381	-1.01	1.87E-04	3.57E-03
1445	ENSG00000143158.10	MPC2	0.90	1.89E-04	3.61E-03
1446	ENSG00000157111.12	TMEM171	3.61	1.92E-04	3.66E-03
1447	ENSG00000274173.1	AL035661.1	1.89	1.93E-04	3.67E-03

1448	ENSG00000138411.12	HECW2	0.76	1.93E-04	3.67E-03
1449	ENSG00000182379.9	NXPH4	1.96	1.93E-04	3.67E-03
1450	ENSG00000139842.14	CUL4A	-0.27	1.95E-04	3.71E-03
1451	ENSG00000128274.16	A4GALT	-0.72	1.95E-04	3.71E-03
1452	ENSG00000163378.13	EOGT	-0.52	1.96E-04	3.72E-03
1453	ENSG00000133424.20	LARGE1	-0.55	1.97E-04	3.74E-03
1454	ENSG00000188783.5	PRELP	-0.81	1.97E-04	3.74E-03
1455	ENSG00000267714.1	AC008738.4	-1.54	1.97E-04	3.74E-03
1456	ENSG00000170919.15	TPT1-AS1	-0.46	2.01E-04	3.80E-03
1457	ENSG00000279339.1	AC100788.2	-0.83	2.04E-04	3.86E-03
1458	ENSG00000111716.12	LDHB	0.60	2.04E-04	3.87E-03
1459	ENSG00000128595.16	CALU	-0.34	2.06E-04	3.89E-03
1460	ENSG00000182168.14	UNC5C	-1.07	2.09E-04	3.94E-03
1461	ENSG00000184708.17	EIF4ENIF1	-0.36	2.11E-04	3.97E-03
1462	ENSG00000216817.1	R3HDM2P2	-3.36	2.11E-04	3.97E-03
1463	ENSG00000117013.15	KCNQ4	1.65	2.11E-04	3.98E-03
1464	ENSG00000171408.13	PDE7B	0.74	2.11E-04	3.98E-03
1465	ENSG00000175084.11	DES	2.42	2.12E-04	3.99E-03
1466	ENSG00000158555.14	GDPD5	0.81	2.13E-04	4.01E-03
1467	ENSG00000256364.1	AC069234.2	0.71	2.13E-04	4.01E-03
1468	ENSG00000105976.14	MET	-0.70	2.13E-04	4.01E-03
1469	ENSG00000150457.8	LATS2	-0.48	2.15E-04	4.04E-03
1470	ENSG00000250266.1	LINC01612	2.02	2.16E-04	4.05E-03
1471	ENSG00000100242.15	SUN2	-0.46	2.18E-04	4.09E-03
1472	ENSG00000130957.4	FBP2	6.07	2.19E-04	4.10E-03
1473	ENSG00000133706.17	LARS	0.44	2.19E-04	4.10E-03
1474	ENSG00000066629.17	EML1	0.39	2.19E-04	4.10E-03
1475	ENSG00000090432.6	MUL1	0.51	2.20E-04	4.11E-03
1476	ENSG00000166401.14	SERPINB8	-0.87	2.24E-04	4.18E-03
1477	ENSG00000175518.6	UBQLNL	3.09	2.26E-04	4.22E-03
1478	ENSG00000124593.16	AL365205.1	-0.61	2.27E-04	4.24E-03
1479	ENSG00000203799.12	CCDC162P	-1.22	2.28E-04	4.24E-03
1480	ENSG00000075336.11	TIMM21	0.62	2.30E-04	4.28E-03
1481	ENSG00000116260.16	QSOX1	-0.50	2.30E-04	4.28E-03
1482	ENSG00000244062.1	AC080128.1	1.90	2.30E-04	4.28E-03
1483	ENSG00000147123.10	NDUFB11	0.76	2.31E-04	4.29E-03
1484	ENSG00000171132.13	PRKCE	0.54	2.31E-04	4.30E-03
1485	ENSG00000259571.1	BLID	-1.73	2.31E-04	4.30E-03
1486	ENSG00000197971.14	MBP	-0.63	2.33E-04	4.32E-03
1487	ENSG00000036672.15	USP2	1.25	2.33E-04	4.33E-03
1488	ENSG00000118849.9	RARRES1	0.93	2.35E-04	4.35E-03
1489	ENSG00000160218.12	TRAPPC10	0.47	2.38E-04	4.41E-03
1490	ENSG00000132326.11	PER2	-1.02	2.38E-04	4.41E-03
1491	ENSG00000185008.17	ROBO2	-1.08	2.39E-04	4.42E-03
1492	ENSG00000149131.15	SERPING1	-0.67	2.39E-04	4.42E-03
1493	ENSG00000226124.7	FTCDNL1	0.93	2.41E-04	4.45E-03
1494	ENSG00000090013.10	BLVRB	0.64	2.42E-04	4.47E-03
1495	ENSG00000171819.4	ANGPTL7	1.02	2.44E-04	4.50E-03
1496	ENSG00000062582.13	MRPS24	0.72	2.44E-04	4.50E-03
1497	ENSG00000111843.13	TMEM14C	0.55	2.44E-04	4.50E-03

1498	ENSG00000152689.17	RASGRP3	0.66	2.46E-04	4.53E-03
1499	ENSG00000136861.17	CDK5RAP2	-0.32	2.48E-04	4.56E-03
1500	ENSG00000178404.9	CEP295NL	-0.83	2.48E-04	4.56E-03
1501	ENSG00000167136.6	ENDOG	1.67	2.49E-04	4.57E-03
1502	ENSG00000106351.12	AGFG2	-0.78	2.49E-04	4.57E-03
1503	ENSG00000196914.8	ARHGEF12	0.32	2.49E-04	4.57E-03
1504	ENSG00000164010.14	ERMAP	-0.60	2.49E-04	4.57E-03
1505	ENSG00000130956.13	HABP4	-0.50	2.51E-04	4.60E-03
1506	ENSG00000168237.17	GLYCTK	1.08	2.52E-04	4.61E-03
1507	ENSG00000115170.13	ACVR1	-0.67	2.53E-04	4.63E-03
1508	ENSG00000166902.4	MRPL16	0.63	2.54E-04	4.64E-03
1509	ENSG00000072110.13	ACTN1	-0.46	2.54E-04	4.65E-03
1510	ENSG00000109339.21	MAPK10	0.42	2.55E-04	4.66E-03
1511	ENSG00000072071.16	ADGRL1	-0.51	2.57E-04	4.68E-03
1512	ENSG00000269107.1	AC092329.1	-2.82	2.57E-04	4.68E-03
1513	ENSG00000211955.2	IGHV3-33	5.20	2.58E-04	4.70E-03
1514	ENSG00000175471.19	MCTP1	-0.45	2.58E-04	4.70E-03
1515	ENSG00000135074.15	ADAM19	0.64	2.58E-04	4.70E-03
1516	ENSG00000182199.10	SHMT2	0.61	2.62E-04	4.77E-03
1517	ENSG00000155906.17	RMND1	0.47	2.62E-04	4.77E-03
1518	ENSG00000198929.12	NOS1AP	-0.91	2.65E-04	4.81E-03
1519	ENSG00000241860.6	AL627309.5	0.71	2.66E-04	4.83E-03
1520	ENSG00000257964.1	AC020612.3	-0.88	2.67E-04	4.84E-03
1521	ENSG00000105402.7	NAPA	0.40	2.67E-04	4.85E-03
1522	ENSG00000130734.9	ATG4D	0.93	2.68E-04	4.85E-03
1523	ENSG00000266962.2	AC067852.2	-0.94	2.69E-04	4.86E-03
1524	ENSG00000119185.12	ITGB1BP1	-0.51	2.70E-04	4.89E-03
1525	ENSG00000159433.11	STARD9	-0.34	2.71E-04	4.90E-03
1526	ENSG00000064218.4	DMRT3	1.58	2.72E-04	4.91E-03
1527	ENSG00000260877.2	AP005233.2	-5.76	2.76E-04	4.99E-03
1528	ENSG00000152402.10	GUCY1A2	0.44	2.77E-04	5.00E-03
1529	ENSG00000164776.9	PHKG1	-1.39	2.77E-04	5.00E-03
1530	ENSG00000165841.10	CYP2C19	1.67	2.79E-04	5.03E-03
1531	ENSG00000257267.3	ZNF271P	0.47	2.80E-04	5.04E-03
1532	ENSG00000180787.5	ZFP3	0.55	2.81E-04	5.06E-03
1533	ENSG00000153823.18	PID1	-0.64	2.83E-04	5.09E-03
1534	ENSG00000213398.7	LCAT	-0.51	2.86E-04	5.14E-03
1535	ENSG00000111726.12	CMAS	-0.43	2.86E-04	5.14E-03
1536	ENSG00000204843.12	DCTN1	-0.32	2.88E-04	5.16E-03
1537	ENSG00000099822.2	HCN2	2.74	2.91E-04	5.22E-03
1538	ENSG00000163909.7	HEYL	1.01	2.92E-04	5.23E-03
1539	ENSG00000176194.17	CIDEA	0.75	2.92E-04	5.23E-03
1540	ENSG00000091704.9	CPA1	1.48	2.93E-04	5.24E-03
1541	ENSG00000237263.1	MAPK6P3	1.08	2.95E-04	5.28E-03
1542	ENSG00000238755.3	LINC02006	2.24	2.95E-04	5.28E-03
1543	ENSG00000145476.15	CYP4V2	0.66	2.96E-04	5.29E-03
1544	ENSG00000167600.13	CYP2S1	2.06	2.97E-04	5.31E-03
1545	ENSG00000186104.10	CYP2R1	0.67	3.00E-04	5.35E-03
1546	ENSG00000275601.1	AC011330.2	1.35	3.00E-04	5.35E-03
1547	ENSG00000206120.11	EGFEM1P	0.80	3.01E-04	5.36E-03

1548	ENSG00000101182.14	PSMA7	0.36	3.01E-04	5.36E-03
1549	ENSG00000185875.12	THNSL1	0.62	3.03E-04	5.40E-03
1550	ENSG00000154229.11	PRKCA	0.55	3.04E-04	5.41E-03
1551	ENSG00000144619.14	CNTN4	1.73	3.05E-04	5.42E-03
1552	ENSG00000170876.7	TMEM43	-0.50	3.06E-04	5.43E-03
1553	ENSG00000177600.8	RPLP2	0.53	3.07E-04	5.46E-03
1554	ENSG00000077942.18	FBLN1	-0.74	3.08E-04	5.46E-03
1555	ENSG00000253424.1	AC025437.2	-0.92	3.08E-04	5.46E-03
1556	ENSG00000167232.13	ZNF91	0.35	3.11E-04	5.51E-03
1557	ENSG00000179542.15	SLITRK4	-1.08	3.12E-04	5.53E-03
1558	ENSG00000107874.10	CUEDC2	0.52	3.13E-04	5.53E-03
1559	ENSG00000177666.16	PNPLA2	0.54	3.14E-04	5.56E-03
1560	ENSG00000095627.9	TDRD1	1.47	3.16E-04	5.58E-03
1561	ENSG00000187942.11	LDLRAD2	-0.69	3.16E-04	5.58E-03
1562	ENSG00000184992.10	BRI3BP	0.68	3.18E-04	5.61E-03
1563	ENSG00000196465.10	MYL6B	0.62	3.20E-04	5.64E-03
1564	ENSG00000176171.11	BNIP3	0.43	3.20E-04	5.65E-03
1565	ENSG00000041353.9	RAB27B	-2.60	3.22E-04	5.68E-03
1566	ENSG00000279026.1	AC005225.4	0.55	3.24E-04	5.70E-03
1567	ENSG00000085733.15	CTTN	-0.36	3.24E-04	5.70E-03
1568	ENSG00000100284.20	TOM1	-0.46	3.27E-04	5.76E-03
1569	ENSG00000121552.3	CSTA	0.89	3.30E-04	5.79E-03
1570	ENSG00000198793.12	MTOR	0.33	3.34E-04	5.87E-03
1571	ENSG00000205277.9	MUC12	-1.63	3.38E-04	5.94E-03
1572	ENSG00000008083.13	JARID2	0.43	3.41E-04	5.98E-03
1573	ENSG00000128652.11	HOXD3	-0.90	3.42E-04	5.99E-03
1574	ENSG00000145675.14	PIK3R1	-0.53	3.43E-04	6.00E-03
1575	ENSG00000257270.1	AL928654.2	-0.45	3.44E-04	6.03E-03
1576	ENSG00000124788.18	ATXN1	-0.46	3.45E-04	6.03E-03
1577	ENSG00000133641.17	C12orf29	0.62	3.47E-04	6.08E-03
1578	ENSG00000213903.8	LTB4R	-0.57	3.49E-04	6.09E-03
1579	ENSG00000164099.3	PRSS12	-1.27	3.49E-04	6.10E-03
1580	ENSG00000122359.17	ANXA11	-0.38	3.52E-04	6.14E-03
1581	ENSG00000083544.14	TDRD3	0.48	3.53E-04	6.15E-03
1582	ENSG00000213088.10	ACKR1	-0.98	3.53E-04	6.15E-03
1583	ENSG00000166046.10	TCP11L2	-0.54	3.55E-04	6.18E-03
1584	ENSG00000154153.13	RETREG1	0.83	3.55E-04	6.18E-03
1585	ENSG00000126561.16	STAT5A	-0.45	3.57E-04	6.20E-03
1586	ENSG00000188997.7	KCTD21	0.64	3.57E-04	6.20E-03
1587	ENSG00000164896.19	FASTK	0.48	3.57E-04	6.21E-03
1588	ENSG00000038295.7	TLL1	-0.71	3.58E-04	6.21E-03
1589	ENSG00000138449.10	SLC40A1	0.55	3.59E-04	6.23E-03
1590	ENSG00000223813.2	AC007255.1	-3.07	3.60E-04	6.24E-03
1591	ENSG00000196549.10	MME	0.77	3.62E-04	6.28E-03
1592	ENSG00000162377.5	COA7	0.59	3.65E-04	6.32E-03
1593	ENSG00000100234.11	TIMP3	-0.58	3.65E-04	6.32E-03
1594	ENSG00000164442.9	CITED2	-0.85	3.65E-04	6.32E-03
1595	ENSG00000134853.11	PDGFRA	-0.57	3.67E-04	6.35E-03
1596	ENSG00000139116.18	KIF21A	0.57	3.68E-04	6.36E-03
1597	ENSG00000134871.18	COL4A2	-0.36	3.69E-04	6.37E-03

1598	ENSG00000137726.16	FXYD6	0.62	3.75E-04	6.47E-03
1599	ENSG00000166025.17	AMOTL1	0.38	3.75E-04	6.47E-03
1600	ENSG00000180155.19	LYNX1	0.74	3.77E-04	6.48E-03
1601	ENSG00000257365.7	FNTB	0.55	3.77E-04	6.48E-03
1602	ENSG00000134899.19	ERCC5	-0.34	3.76E-04	6.48E-03
1603	ENSG00000198001.13	IRAK4	-0.48	3.76E-04	6.48E-03
1604	ENSG00000163701.18	IL17RE	-0.71	3.77E-04	6.48E-03
1605	ENSG00000174307.6	PHLDA3	-0.65	3.78E-04	6.49E-03
1606	ENSG00000109971.13	HSPA8	0.62	3.78E-04	6.49E-03
1607	ENSG00000225313.5	AL513327.1	0.60	3.78E-04	6.49E-03
1608	ENSG00000169925.16	BRD3	-0.40	3.81E-04	6.53E-03
1609	ENSG00000185565.11	LSAMP	-0.90	3.83E-04	6.56E-03
1610	ENSG00000138735.15	PDE5A	0.55	3.84E-04	6.57E-03
1611	ENSG00000196975.15	ANXA4	-0.39	3.84E-04	6.57E-03
1612	ENSG00000274523.4	RCC1L	0.37	3.85E-04	6.59E-03
1613	ENSG00000178761.14	FAM219B	0.44	3.86E-04	6.61E-03
1614	ENSG00000175470.19	PPP2R2D	0.37	3.89E-04	6.64E-03
1615	ENSG00000132824.13	SERINC3	-0.31	3.89E-04	6.64E-03
1616	ENSG00000284968.1	AC093827.4	0.56	3.91E-04	6.67E-03
1617	ENSG00000183695.2	MRGPRX2	-3.60	3.91E-04	6.67E-03
1618	ENSG00000114771.13	AADAC	-3.97	3.91E-04	6.67E-03
1619	ENSG00000198931.10	APRT	0.67	3.93E-04	6.69E-03
1620	ENSG00000113070.7	HBEGF	0.97	3.95E-04	6.72E-03
1621	ENSG00000185122.10	HSF1	0.61	3.97E-04	6.75E-03
1622	ENSG00000065457.10	ADAT1	0.64	4.01E-04	6.82E-03
1623	ENSG00000062282.14	DGAT2	1.07	4.01E-04	6.82E-03
1624	ENSG00000144366.15	GULP1	0.49	4.02E-04	6.83E-03
1625	ENSG00000132846.5	ZBED3	0.59	4.02E-04	6.83E-03
1626	ENSG00000184470.20	TXNRD2	0.56	4.05E-04	6.87E-03
1627	ENSG00000183723.12	CMTM4	0.55	4.05E-04	6.87E-03
1628	ENSG00000184232.8	OAF	0.64	4.06E-04	6.88E-03
1629	ENSG00000281332.1	LINC00997	-0.60	4.08E-04	6.90E-03
1630	ENSG00000137699.16	TRIM29	-4.06	4.08E-04	6.90E-03
1631	ENSG00000164638.10	SLC29A4	0.63	4.11E-04	6.96E-03
1632	ENSG00000100311.16	PDGFB	0.61	4.14E-04	6.99E-03
1633	ENSG00000160712.12	IL6R	-0.59	4.14E-04	6.99E-03
1634	ENSG00000073792.15	IGF2BP2	-0.54	4.14E-04	6.99E-03
1635	ENSG00000172840.6	PDP2	0.47	4.15E-04	7.00E-03
1636	ENSG00000142657.20	PGD	0.46	4.16E-04	7.01E-03
1637	ENSG00000167705.11	RILP	0.63	4.17E-04	7.03E-03
1638	ENSG00000203837.4	PNLIPRP3	-7.35	4.17E-04	7.03E-03
1639	ENSG00000111879.19	FAM184A	0.77	4.19E-04	7.05E-03
1640	ENSG00000100439.10	ABHD4	0.69	4.19E-04	7.05E-03
1641	ENSG00000130159.13	ECSIT	0.72	4.21E-04	7.08E-03
1642	ENSG00000164211.12	STARD4	1.22	4.22E-04	7.08E-03
1643	ENSG00000140525.17	FANCI	-0.71	4.23E-04	7.09E-03
1644	ENSG00000182718.16	ANXA2	-0.44	4.25E-04	7.14E-03
1645	ENSG00000197496.5	SLC2A10	-0.70	4.28E-04	7.17E-03
1646	ENSG00000099968.17	BCL2L13	0.32	4.28E-04	7.18E-03
1647	ENSG00000140287.10	HDC	-2.51	4.30E-04	7.19E-03

1648	ENSG00000251191.7	LINC00589	4.31	4.34E-04	7.27E-03
1649	ENSG00000118816.9	CCNI	0.36	4.36E-04	7.30E-03
1650	ENSG00000142227.10	EMP3	-0.62	4.37E-04	7.31E-03
1651	ENSG00000279588.2	AC087762.2	-1.08	4.41E-04	7.36E-03
1652	ENSG00000269713.7	NBPF9	-0.46	4.44E-04	7.41E-03
1653	ENSG00000125510.15	OPRL1	-1.45	4.45E-04	7.42E-03
1654	ENSG00000102317.17	RBM3	-0.48	4.45E-04	7.43E-03
1655	ENSG00000135052.16	GOLM1	-0.49	4.46E-04	7.43E-03
1656	ENSG00000179603.17	GRM8	3.31	4.48E-04	7.46E-03
1657	ENSG00000179241.12	LDLRAD3	0.60	4.50E-04	7.49E-03
1658	ENSG00000162627.16	SNX7	-0.68	4.50E-04	7.49E-03
1659	ENSG00000034971.16	MYOC	1.49	4.51E-04	7.49E-03
1660	ENSG00000172724.11	CCL19	-2.06	4.52E-04	7.52E-03
1661	ENSG00000119514.6	GALNT12	-0.81	4.54E-04	7.53E-03
1662	ENSG00000164114.18	MAP9	0.68	4.56E-04	7.57E-03
1663	ENSG00000151789.11	ZNF385D	-0.68	4.57E-04	7.58E-03
1664	ENSG00000240184.6	PCDHGC3	-0.67	4.57E-04	7.58E-03
1665	ENSG00000099308.10	MAST3	-0.73	4.65E-04	7.70E-03
1666	ENSG00000175348.10	TMEM9B	0.45	4.65E-04	7.70E-03
1667	ENSG00000280224.1	AL008636.1	-1.03	4.66E-04	7.71E-03
1668	ENSG00000070526.14	ST6GALNAC1	-1.58	4.68E-04	7.73E-03
1669	ENSG00000119801.12	YPEL5	-0.45	4.68E-04	7.73E-03
1670	ENSG00000143869.6	GDF7	1.50	4.69E-04	7.74E-03
1671	ENSG00000138433.15	CIR1	-0.44	4.70E-04	7.75E-03
1672	ENSG00000162616.8	DNAJB4	0.51	4.72E-04	7.78E-03
1673	ENSG00000213722.8	DDAH2	-0.54	4.73E-04	7.78E-03
1674	ENSG00000179899.8	PHC1P1	-0.70	4.72E-04	7.78E-03
1675	ENSG00000153303.17	FRMD1	-1.09	4.72E-04	7.78E-03
1676	ENSG00000184838.14	PRR16	-1.77	4.77E-04	7.84E-03
1677	ENSG00000104936.17	DMPK	-0.58	4.79E-04	7.87E-03
1678	ENSG00000170291.14	ELP5	0.61	4.83E-04	7.94E-03
1679	ENSG00000159445.12	THEM4	0.68	4.86E-04	7.99E-03
1680	ENSG00000235590.7	GNAS-AS1	-1.19	4.86E-04	7.99E-03
1681	ENSG00000153933.9	DGKE	0.43	4.87E-04	7.99E-03
1682	ENSG00000140105.17	WARS	0.75	4.88E-04	8.01E-03
1683	ENSG00000169446.5	MMGT1	0.34	4.89E-04	8.01E-03
1684	ENSG00000179562.2	GCC1	-0.52	4.89E-04	8.01E-03
1685	ENSG00000125851.9	PCSK2	1.11	4.91E-04	8.04E-03
1686	ENSG00000143919.14	CAMKMT	0.57	4.93E-04	8.07E-03
1687	ENSG00000161509.13	GRIN2C	1.44	4.94E-04	8.08E-03
1688	ENSG00000184117.11	NIPSNAP1	0.65	4.95E-04	8.09E-03
1689	ENSG00000152784.15	PRDM8	-1.24	4.97E-04	8.12E-03
1690	ENSG00000196922.10	ZNF252P	0.42	4.99E-04	8.14E-03
1691	ENSG00000156253.6	RWDD2B	0.45	5.00E-04	8.15E-03
1692	ENSG00000167862.9	MRPL58	0.65	5.05E-04	8.24E-03
1693	ENSG00000160113.5	NR2F6	0.70	5.07E-04	8.25E-03
1694	ENSG00000167522.15	ANKRD11	-0.31	5.07E-04	8.25E-03
1695	ENSG00000233622.3	CYP2T1P	-1.08	5.07E-04	8.25E-03
1696	ENSG00000078687.17	TNRC6C	-0.27	5.08E-04	8.25E-03
1697	ENSG00000122482.20	ZNF644	0.33	5.11E-04	8.31E-03

1698	ENSG00000237238.3	BMS1P10	0.71	5.12E-04	8.31E-03
1699	ENSG00000237781.3	AL356356.1	-1.67	5.15E-04	8.37E-03
1700	ENSG00000166311.9	SMPD1	0.47	5.17E-04	8.39E-03
1701	ENSG00000119787.13	ATL2	0.61	5.18E-04	8.40E-03
1702	ENSG00000165548.10	TMEM63C	2.52	5.23E-04	8.47E-03
1703	ENSG00000142530.10	FAM71E1	3.13	5.23E-04	8.47E-03
1704	ENSG00000185825.16	BCAP31	0.46	5.24E-04	8.48E-03
1705	ENSG00000106610.15	STAG3L4	0.59	5.25E-04	8.50E-03
1706	ENSG00000129250.11	KIF1C	-0.47	5.25E-04	8.50E-03
1707	ENSG00000135900.3	MRPL44	0.71	5.28E-04	8.53E-03
1708	ENSG00000248187.1	AC078850.1	1.94	5.28E-04	8.53E-03
1709	ENSG00000254772.9	EEF1G	0.39	5.29E-04	8.54E-03
1710	ENSG00000119760.15	SUPT7L	0.36	5.32E-04	8.58E-03
1711	ENSG00000182463.15	TSHZ2	0.58	5.33E-04	8.59E-03
1712	ENSG00000111906.17	HDDC2	0.56	5.33E-04	8.59E-03
1713	ENSG00000182389.19	CACNB4	-0.86	5.33E-04	8.59E-03
1714	ENSG00000006042.11	TMEM98	-0.84	5.35E-04	8.62E-03
1715	ENSG00000254470.2	AP5B1	0.91	5.36E-04	8.62E-03
1716	ENSG00000174173.6	TRMT10C	0.51	5.36E-04	8.62E-03
1717	ENSG00000204822.6	MRPL53	0.50	5.42E-04	8.70E-03
1718	ENSG00000196569.12	LAMA2	-0.47	5.41E-04	8.70E-03
1719	ENSG00000046889.18	PREX2	-0.43	5.43E-04	8.71E-03
1720	ENSG00000271200.1	AC099791.2	-2.02	5.45E-04	8.74E-03
1721	ENSG00000100106.20	TRIOBP	-0.48	5.46E-04	8.75E-03
1722	ENSG00000181788.3	SIAH2	0.78	5.46E-04	8.75E-03
1723	ENSG00000225190.10	PLEKHM1	-0.51	5.50E-04	8.80E-03
1724	ENSG00000049246.14	PER3	-1.54	5.52E-04	8.83E-03
1725	ENSG00000153904.19	DDAH1	-0.83	5.54E-04	8.86E-03
1726	ENSG00000165476.13	REEP3	0.36	5.58E-04	8.92E-03
1727	ENSG00000267361.1	SEC24AP1	-1.07	5.59E-04	8.92E-03
1728	ENSG00000102287.18	GABRE	-0.54	5.60E-04	8.94E-03
1729	ENSG00000139517.8	LNX2	0.63	5.62E-04	8.97E-03
1730	ENSG00000154122.13	ANKH	0.36	5.63E-04	8.97E-03
1731	ENSG00000133477.16	FAM83F	-0.86	5.64E-04	8.99E-03
1732	ENSG00000197766.7	CFD	-0.61	5.68E-04	9.04E-03
1733	ENSG00000138741.10	TRPC3	1.61	5.70E-04	9.07E-03
1734	ENSG00000188517.15	COL25A1	-0.60	5.70E-04	9.07E-03
1735	ENSG00000077238.13	IL4R	-0.71	5.71E-04	9.08E-03
1736	ENSG00000165868.14	HSPA12A	0.80	5.72E-04	9.09E-03
1737	ENSG00000134987.11	WDR36	0.41	5.73E-04	9.10E-03
1738	ENSG00000093167.17	LRRFIP2	-0.32	5.74E-04	9.11E-03
1739	ENSG00000272645.3	GTF2IP20	0.66	5.74E-04	9.11E-03
1740	ENSG00000262406.2	MMP12	-5.34	5.78E-04	9.16E-03
1741	ENSG00000260097.2	SPDYE6	-1.19	5.79E-04	9.18E-03
1742	ENSG00000176532.3	PRR15	-3.90	5.85E-04	9.27E-03
1743	ENSG00000147586.9	MRPS28	0.45	5.94E-04	9.39E-03
1744	ENSG00000187753.13	C9orf153	1.57	5.96E-04	9.42E-03
1745	ENSG00000162600.11	OMA1	0.42	5.97E-04	9.44E-03
1746	ENSG00000048471.13	SNX29	-0.35	5.99E-04	9.46E-03
1747	ENSG00000236762.1	RPL19P16	-5.24	5.99E-04	9.46E-03

1748	ENSG00000126953.6	TIMM8A	0.67	6.00E-04	9.47E-03
1749	ENSG00000285184.2	AC244033.2	0.80	6.05E-04	9.54E-03
1750	ENSG00000144749.13	LRIG1	-0.67	6.06E-04	9.55E-03
1751	ENSG00000183853.17	KIRREL1	-0.34	6.08E-04	9.57E-03
1752	ENSG00000113532.12	ST8SIA4	0.72	6.10E-04	9.60E-03
1753	ENSG00000143013.12	LMO4	-0.60	6.10E-04	9.60E-03
1754	ENSG00000215450.2	AL022342.1	3.25	6.10E-04	9.60E-03
1755	ENSG00000258730.1	ITPK1-AS1	1.32	6.12E-04	9.61E-03
1756	ENSG00000169313.9	P2RY12	-1.48	6.12E-04	9.61E-03
1757	ENSG00000236077.2	BX119904.3	-3.02	6.12E-04	9.61E-03
1758	ENSG00000048707.15	VPS13D	0.30	6.13E-04	9.61E-03
1759	ENSG00000125755.18	SYMPK	-0.37	6.13E-04	9.61E-03
1760	ENSG00000187554.12	TLR5	0.61	6.13E-04	9.61E-03
1761	ENSG00000090060.17	PAPOLA	0.26	6.14E-04	9.61E-03
1762	ENSG00000087299.11	L2HGDH	0.38	6.14E-04	9.62E-03
1763	ENSG00000106733.20	NMRK1	-0.42	6.15E-04	9.62E-03
1764	ENSG00000174718.11	KIAA1551	0.37	6.18E-04	9.66E-03
1765	ENSG00000142173.14	COL6A2	-0.59	6.19E-04	9.67E-03
1766	ENSG00000243193.4	AC006387.1	0.67	6.20E-04	9.68E-03
1767	ENSG00000183023.18	SLC8A1	0.58	6.23E-04	9.73E-03
1768	ENSG00000198517.9	MAFK	-0.62	6.24E-04	9.74E-03
1769	ENSG00000280245.1	AC079336.7	-2.09	6.29E-04	9.80E-03
1770	ENSG00000128872.9	TMOD2	0.43	6.31E-04	9.83E-03
1771	ENSG00000147592.8	LACTB2	0.96	6.35E-04	9.87E-03
1772	ENSG00000115520.8	COQ10B	0.51	6.35E-04	9.87E-03
1773	ENSG00000129128.12	SPCS3	0.34	6.35E-04	9.87E-03
1774	ENSG00000240755.1	ERLEC1P1	-4.09	6.35E-04	9.87E-03
1775	ENSG00000106049.8	HIBADH	0.55	6.41E-04	9.96E-03
1776	ENSG00000149970.15	CNKSR2	-0.50	6.41E-04	9.96E-03
1777	ENSG00000172893.15	DHCR7	0.88	6.43E-04	9.98E-03
1778	ENSG00000154265.15	ABCA5	-0.35	6.44E-04	9.99E-03
1779	ENSG00000129055.12	ANAPC13	0.60	6.46E-04	1.00E-02
1780	ENSG00000141030.12	COPS3	0.55	6.48E-04	1.00E-02
1781	ENSG00000180769.8	WDFY3-AS2	0.62	6.51E-04	1.01E-02
1782	ENSG00000138796.16	HADH	0.48	6.55E-04	1.01E-02
1783	ENSG00000235961.5	PNMA6A	-1.29	6.56E-04	1.02E-02
1784	ENSG00000169302.15	STK32A	-1.26	6.57E-04	1.02E-02
1785	ENSG00000107223.12	EDF1	0.46	6.58E-04	1.02E-02
1786	ENSG00000268104.2	SLC6A14	-1.85	6.59E-04	1.02E-02
1787	ENSG00000135976.18	ANKRD36	-0.50	6.62E-04	1.02E-02
1788	ENSG00000103495.13	MAZ	-0.40	6.72E-04	1.04E-02
1789	ENSG00000070950.9	RAD18	-0.53	6.71E-04	1.04E-02
1790	ENSG00000112319.18	EYA4	-0.59	6.73E-04	1.04E-02
1791	ENSG00000224272.2	AC131097.3	-2.47	6.73E-04	1.04E-02
1792	ENSG00000139263.11	LRIG3	-0.55	6.74E-04	1.04E-02
1793	ENSG00000140526.17	ABHD2	-0.29	6.76E-04	1.04E-02
1794	ENSG00000184205.14	TSPYL2	-0.51	6.80E-04	1.05E-02
1795	ENSG00000181195.10	PENK	-2.28	6.81E-04	1.05E-02
1796	ENSG00000179918.18	SEPHS2	0.41	6.81E-04	1.05E-02
1797	ENSG00000100592.15	DAAM1	-0.61	6.81E-04	1.05E-02

1798	ENSG00000145604.15	SKP2	-0.43	6.84E-04	1.05E-02
1799	ENSG00000121671.11	CRY2	-0.80	6.85E-04	1.05E-02
1800	ENSG00000183791.4	ELOA3	1.62	6.86E-04	1.05E-02
1801	ENSG00000147010.17	SH3KBP1	0.36	6.85E-04	1.05E-02
1802	ENSG00000107829.13	FBXW4	-0.46	6.89E-04	1.05E-02
1803	ENSG00000186160.4	CYP4Z1	-1.16	6.90E-04	1.06E-02
1804	ENSG00000006757.11	PNPLA4	0.65	6.92E-04	1.06E-02
1805	ENSG00000177283.7	FZD8	0.92	6.92E-04	1.06E-02
1806	ENSG00000175701.10	MTLN	0.98	6.96E-04	1.06E-02
1807	ENSG00000281383.1	FP671120.4	-0.62	6.96E-04	1.06E-02
1808	ENSG00000125878.6	TCF15	1.08	6.97E-04	1.06E-02
1809	ENSG00000168818.9	STX18	-0.37	6.97E-04	1.06E-02
1810	ENSG00000259916.1	AL845331.2	1.17	7.00E-04	1.07E-02
1811	ENSG00000068308.13	OTUD5	-0.40	7.02E-04	1.07E-02
1812	ENSG00000184900.15	SUMO3	-0.33	7.03E-04	1.07E-02
1813	ENSG00000261340.1	LINC01616	-3.29	7.04E-04	1.07E-02
1814	ENSG00000120049.19	KCNIP2	0.52	7.14E-04	1.09E-02
1815	ENSG00000229056.2	AC020571.1	1.36	7.22E-04	1.10E-02
1816	ENSG00000114166.7	KAT2B	0.38	7.22E-04	1.10E-02
1817	ENSG00000079102.16	RUNX1T1	-0.39	7.24E-04	1.10E-02
1818	ENSG00000142798.19	HSPG2	-0.48	7.26E-04	1.10E-02
1819	ENSG00000134030.13	CTIF	-0.47	7.28E-04	1.10E-02
1820	ENSG00000103150.5	MLYCD	0.39	7.33E-04	1.11E-02
1821	ENSG00000123560.13	PLP1	0.91	7.35E-04	1.11E-02
1822	ENSG00000130347.12	RTN4IP1	0.86	7.35E-04	1.11E-02
1823	ENSG00000109016.17	DHRS7B	0.47	7.35E-04	1.11E-02
1824	ENSG00000170801.9	HTRA3	-0.97	7.38E-04	1.12E-02
1825	ENSG00000105887.10	MTPN	0.38	7.40E-04	1.12E-02
1826	ENSG00000109919.9	MTCH2	0.56	7.42E-04	1.12E-02
1827	ENSG00000167186.10	COQ7	0.49	7.43E-04	1.12E-02
1828	ENSG00000177576.11	C18orf32	0.69	7.43E-04	1.12E-02
1829	ENSG00000116815.15	CD58	0.58	7.44E-04	1.12E-02
1830	ENSG00000106991.13	ENG	0.50	7.45E-04	1.12E-02
1831	ENSG00000116885.18	OSCP1	-0.75	7.44E-04	1.12E-02
1832	ENSG00000153179.12	RASSF3	-0.43	7.47E-04	1.12E-02
1833	ENSG00000148200.16	NR6A1	1.00	7.48E-04	1.13E-02
1834	ENSG00000069667.15	RORA	-0.44	7.51E-04	1.13E-02
1835	ENSG00000112531.16	QKI	0.30	7.53E-04	1.13E-02
1836	ENSG00000170322.14	NFRKB	-0.36	7.53E-04	1.13E-02
1837	ENSG00000122203.14	KIAA1191	0.38	7.54E-04	1.13E-02
1838	ENSG00000134765.9	DSC1	-4.12	7.54E-04	1.13E-02
1839	ENSG00000145708.10	CRHBP	0.98	7.58E-04	1.14E-02
1840	ENSG00000156256.14	USP16	0.36	7.61E-04	1.14E-02
1841	ENSG00000187446.11	CHP1	0.52	7.62E-04	1.14E-02
1842	ENSG00000113580.14	NR3C1	-0.42	7.62E-04	1.14E-02
1843	ENSG00000267724.1	AC012254.3	0.99	7.70E-04	1.15E-02
1844	ENSG00000119711.12	ALDH6A1	0.55	7.74E-04	1.16E-02
1845	ENSG00000164967.9	RPP25L	0.96	7.78E-04	1.16E-02
1846	ENSG00000117308.14	GALE	0.62	7.78E-04	1.16E-02
1847	ENSG00000100351.16	GRAP2	-0.91	7.80E-04	1.16E-02

1848	ENSG00000242456.1	MTCO3P38	-4.48	7.80E-04	1.16E-02
1849	ENSG00000184922.13	FMNL1	-0.66	7.83E-04	1.17E-02
1850	ENSG00000115138.10	POMC	1.25	7.85E-04	1.17E-02
1851	ENSG00000112796.9	ENPP5	-1.26	7.86E-04	1.17E-02
1852	ENSG00000137960.5	GIPC2	0.66	7.90E-04	1.18E-02
1853	ENSG00000166340.16	TPP1	0.41	7.90E-04	1.18E-02
1854	ENSG00000169291.9	SHE	0.35	7.97E-04	1.19E-02
1855	ENSG00000113396.12	SLC27A6	2.09	8.02E-04	1.19E-02
1856	ENSG00000167112.9	TRUB2	0.46	8.04E-04	1.20E-02
1857	ENSG00000154262.12	ABCA6	-0.60	8.05E-04	1.20E-02
1858	ENSG00000175591.11	P2RY2	1.65	8.07E-04	1.20E-02
1859	ENSG00000145850.8	TIMD4	3.56	8.08E-04	1.20E-02
1860	ENSG00000260643.2	AC092718.3	0.98	8.13E-04	1.21E-02
1861	ENSG00000170776.21	AKAP13	-0.32	8.13E-04	1.21E-02
1862	ENSG00000124120.10	TTPAL	-0.47	8.15E-04	1.21E-02
1863	ENSG00000155761.13	SPAG17	-1.27	8.18E-04	1.21E-02
1864	ENSG00000120742.10	SERP1	0.30	8.26E-04	1.22E-02
1865	ENSG00000270681.1	AC095055.1	-1.12	8.25E-04	1.22E-02
1866	ENSG00000101856.9	PGRMC1	-0.40	8.28E-04	1.22E-02
1867	ENSG00000240216.7	CPHL1P	-5.55	8.31E-04	1.23E-02
1868	ENSG00000135624.15	CCT7	0.33	8.32E-04	1.23E-02
1869	ENSG00000186652.9	PRG2	-4.15	8.35E-04	1.23E-02
1870	ENSG00000162706.12	CADM3	-0.90	8.37E-04	1.23E-02
1871	ENSG00000140264.19	SERF2	0.40	8.40E-04	1.24E-02
1872	ENSG00000285508.1	AL034430.1	1.63	8.41E-04	1.24E-02
1873	ENSG00000173511.9	VEGFB	0.31	8.43E-04	1.24E-02
1874	ENSG00000117791.15	MARC2	0.61	8.49E-04	1.25E-02
1875	ENSG00000253284.2	AC092828.1	-0.63	8.50E-04	1.25E-02
1876	ENSG00000101266.18	CSNK2A1	0.32	8.52E-04	1.25E-02
1877	ENSG00000138674.16	SEC31A	-0.32	8.53E-04	1.25E-02
1878	ENSG00000110723.11	EXPH5	-1.02	8.52E-04	1.25E-02
1879	ENSG00000091831.23	ESR1	-0.67	8.54E-04	1.25E-02
1880	ENSG00000146674.14	IGFBP3	-0.75	8.53E-04	1.25E-02
1881	ENSG00000163626.16	COX18	0.61	8.55E-04	1.25E-02
1882	ENSG00000059769.19	DNAJC25	0.59	8.58E-04	1.26E-02
1883	ENSG00000284237.1	AL356275.1	0.75	8.61E-04	1.26E-02
1884	ENSG00000123146.19	ADGRE5	-0.53	8.62E-04	1.26E-02
1885	ENSG00000156711.16	MAPK13	-0.96	8.65E-04	1.27E-02
1886	ENSG00000144061.12	NPHP1	-0.60	8.73E-04	1.28E-02
1887	ENSG00000026652.13	AGPAT4	-0.53	8.75E-04	1.28E-02
1888	ENSG00000137135.17	ARHGEF39	-0.99	8.75E-04	1.28E-02
1889	ENSG00000181192.11	DHTKD1	0.56	8.79E-04	1.28E-02
1890	ENSG00000126545.13	CSN1S1	-4.51	8.82E-04	1.29E-02
1891	ENSG00000105088.8	OLFM2	-1.65	8.83E-04	1.29E-02
1892	ENSG00000146859.6	TMEM140	0.46	8.84E-04	1.29E-02
1893	ENSG00000196576.14	PLXNB2	-0.50	8.86E-04	1.29E-02
1894	ENSG00000166573.5	GALR1	-0.75	8.86E-04	1.29E-02
1895	ENSG00000169550.13	MUC15	-1.49	8.87E-04	1.29E-02
1896	ENSG00000129675.15	ARHGEF6	-0.73	8.87E-04	1.29E-02
1897	ENSG00000080503.23	SMARCA2	-0.38	8.93E-04	1.30E-02

1898	ENSG00000175155.8	YPEL2	-0.49	8.93E-04	1.30E-02
1899	ENSG00000170954.11	ZNF415	0.92	8.94E-04	1.30E-02
1900	ENSG00000148498.15	PARD3	-0.33	9.00E-04	1.31E-02
1901	ENSG00000113597.17	TRAPPC13	0.39	9.04E-04	1.31E-02
1902	ENSG00000143437.20	ARNT	-0.37	9.04E-04	1.31E-02
1903	ENSG00000174326.11	SLC16A11	1.97	9.08E-04	1.32E-02
1904	ENSG00000162636.15	FAM102B	-0.38	9.14E-04	1.32E-02
1905	ENSG00000260102.1	LINC01070	-1.43	9.17E-04	1.33E-02
1906	ENSG00000126858.17	RHOT1	0.44	9.21E-04	1.33E-02
1907	ENSG00000137806.8	NDUFAF1	0.68	9.23E-04	1.34E-02
1908	ENSG00000168275.14	COA6	0.67	9.28E-04	1.34E-02
1909	ENSG00000197702.12	PARVA	-0.34	9.38E-04	1.36E-02
1910	ENSG00000102781.13	KATNAL1	-0.36	9.39E-04	1.36E-02
1911	ENSG00000139908.14	TSSK4	-0.63	9.43E-04	1.36E-02
1912	ENSG00000064607.16	SUGP2	0.29	9.46E-04	1.36E-02
1913	ENSG00000119446.13	RBM18	0.37	9.52E-04	1.37E-02
1914	ENSG00000088035.16	ALG6	0.52	9.57E-04	1.38E-02
1915	ENSG00000175265.17	GOLGA8A	-0.34	9.59E-04	1.38E-02
1916	ENSG00000232434.2	AJM1	1.78	9.61E-04	1.38E-02
1917	ENSG00000128185.9	DGCR6L	0.86	9.64E-04	1.39E-02
1918	ENSG00000205517.12	RGL3	0.81	9.66E-04	1.39E-02
1919	ENSG00000130348.11	QRSL1	0.54	9.69E-04	1.39E-02
1920	ENSG00000153485.5	TMEM251	0.62	9.70E-04	1.39E-02
1921	ENSG00000188712.4	OR13D3P	3.04	9.71E-04	1.39E-02
1922	ENSG00000162599.15	NFIA	0.24	9.72E-04	1.40E-02
1923	ENSG00000188811.13	NHLRC3	-0.33	9.75E-04	1.40E-02
1924	ENSG00000111880.15	RNGTT	0.47	9.78E-04	1.40E-02
1925	ENSG00000113758.13	DBN1	-0.52	9.79E-04	1.40E-02
1926	ENSG00000123575.8	FAM199X	0.39	9.82E-04	1.41E-02
1927	ENSG00000164093.16	PITX2	2.40	9.88E-04	1.41E-02
1928	ENSG00000119596.17	YLPM1	-0.26	9.92E-04	1.42E-02
1929	ENSG00000155592.15	ZKSCAN2	-0.48	1.00E-03	1.43E-02
1930	ENSG00000170961.6	HAS2	-0.72	1.00E-03	1.43E-02
1931	ENSG00000152229.18	PSTPIP2	-0.81	1.00E-03	1.43E-02
1932	ENSG00000279209.1	AP001002.1	-2.59	1.01E-03	1.44E-02
1933	ENSG00000249839.1	AC011330.1	1.82	1.01E-03	1.44E-02
1934	ENSG00000126261.12	UBA2	0.40	1.01E-03	1.44E-02
1935	ENSG00000174446.12	SNAPC5	0.44	1.01E-03	1.44E-02
1936	ENSG00000162624.14	LHX8	4.25	1.02E-03	1.45E-02
1937	ENSG00000188677.14	PARVB	0.44	1.02E-03	1.45E-02
1938	ENSG00000161647.18	MPP3	-0.74	1.02E-03	1.45E-02
1939	ENSG00000167680.15	SEMA6B	-0.85	1.02E-03	1.45E-02
1940	ENSG00000009780.15	FAM76A	-0.50	1.02E-03	1.45E-02
1941	ENSG00000110321.16	EIF4G2	0.23	1.02E-03	1.45E-02
1942	ENSG00000099797.14	TECR	0.55	1.02E-03	1.45E-02
1943	ENSG00000269190.5	FBXO17	-0.49	1.02E-03	1.45E-02
1944	ENSG00000082515.17	MRPL22	0.46	1.02E-03	1.45E-02
1945	ENSG00000155008.13	APOOL	0.51	1.03E-03	1.46E-02
1946	ENSG00000105559.11	PLEKHA4	-0.54	1.03E-03	1.46E-02
1947	ENSG00000221983.7	UBA52	0.45	1.03E-03	1.47E-02

1948	ENSG00000205309.13	NT5M	1.52	1.04E-03	1.48E-02
1949	ENSG00000148180.19	GSN	-0.60	1.04E-03	1.48E-02
1950	ENSG00000137801.10	THBS1	-1.87	1.04E-03	1.48E-02
1951	ENSG00000185043.11	CIB1	0.42	1.05E-03	1.48E-02
1952	ENSG00000061794.12	MRPS35	0.73	1.05E-03	1.49E-02
1953	ENSG00000148943.11	LIN7C	0.39	1.05E-03	1.49E-02
1954	ENSG00000132405.18	TBC1D14	0.43	1.05E-03	1.49E-02
1955	ENSG00000272894.5	AC004982.2	-0.57	1.06E-03	1.49E-02
1956	ENSG00000177943.13	MAMDC4	1.05	1.06E-03	1.50E-02
1957	ENSG00000270885.1	RASL10B	-1.04	1.06E-03	1.50E-02
1958	ENSG00000107404.19	DVL1	0.48	1.07E-03	1.50E-02
1959	ENSG00000104687.13	GSR	0.46	1.07E-03	1.50E-02
1960	ENSG00000240775.1	AC021205.1	-3.12	1.07E-03	1.51E-02
1961	ENSG00000183172.8	SMDT1	0.51	1.07E-03	1.51E-02
1962	ENSG00000100442.10	FKBP3	0.48	1.07E-03	1.51E-02
1963	ENSG00000159128.14	IFNGR2	-0.58	1.07E-03	1.51E-02
1964	ENSG00000112782.16	CLIC5	0.66	1.07E-03	1.51E-02
1965	ENSG00000165124.17	SVEP1	-0.79	1.07E-03	1.51E-02
1966	ENSG00000136279.20	DBNL	-0.34	1.08E-03	1.51E-02
1967	ENSG00000010379.15	SLC6A13	3.97	1.08E-03	1.52E-02
1968	ENSG00000271687.1	MTND5P10	1.78	1.09E-03	1.52E-02
1969	ENSG00000125810.9	CD93	0.48	1.09E-03	1.53E-02
1970	ENSG00000276409.4	CCL14	-0.80	1.09E-03	1.53E-02
1971	ENSG00000116857.16	TMEM9	-0.35	1.09E-03	1.53E-02
1972	ENSG00000131097.6	HIGD1B	0.66	1.10E-03	1.53E-02
1973	ENSG00000124313.14	IQSEC2	-0.51	1.10E-03	1.54E-02
1974	ENSG00000148450.12	MSRB2	0.48	1.11E-03	1.55E-02
1975	ENSG00000185379.20	RAD51D	-0.42	1.11E-03	1.55E-02
1976	ENSG00000100473.16	COCH	-0.93	1.11E-03	1.55E-02
1977	ENSG00000172987.12	HPSE2	-1.22	1.11E-03	1.55E-02
1978	ENSG00000150471.16	ADGRL3	0.60	1.11E-03	1.55E-02
1979	ENSG00000205562.2	AL049775.1	-2.52	1.11E-03	1.55E-02
1980	ENSG00000253123.3	AC091182.1	1.37	1.12E-03	1.56E-02
1981	ENSG00000159842.15	ABR	0.49	1.12E-03	1.56E-02
1982	ENSG00000170390.15	DCLK2	0.54	1.12E-03	1.56E-02
1983	ENSG00000185070.10	FLRT2	-0.64	1.12E-03	1.56E-02
1984	ENSG00000102882.11	MAPK3	-0.44	1.13E-03	1.57E-02
1985	ENSG00000254582.1	PSMA2P1	1.29	1.13E-03	1.58E-02
1986	ENSG00000197006.13	METTL9	0.42	1.13E-03	1.58E-02
1987	ENSG00000152700.13	SAR1B	0.34	1.14E-03	1.58E-02
1988	ENSG00000153815.16	CMIP	-0.48	1.14E-03	1.58E-02
1989	ENSG00000100448.3	CTSG	-2.24	1.14E-03	1.58E-02
1990	ENSG00000076770.14	MBNL3	-0.68	1.14E-03	1.58E-02
1991	ENSG00000135480.15	KRT7	-4.84	1.15E-03	1.59E-02
1992	ENSG00000164182.10	NDUFAF2	0.55	1.15E-03	1.59E-02
1993	ENSG00000141401.11	IMPA2	0.61	1.15E-03	1.60E-02
1994	ENSG00000236576.1	AC241520.1	1.38	1.16E-03	1.60E-02
1995	ENSG00000197724.10	PHF2	-0.32	1.17E-03	1.61E-02
1996	ENSG00000241599.1	AL627309.4	1.19	1.17E-03	1.62E-02
1997	ENSG00000133835.15	HSD17B4	0.29	1.18E-03	1.62E-02

1998	ENSG00000229989.3	MIR181A1HG	0.94	1.18E-03	1.63E-02
1999	ENSG00000167601.11	AXL	-0.47	1.19E-03	1.65E-02
2000	ENSG00000176273.14	SLC35G1	-0.67	1.19E-03	1.65E-02
2001	ENSG00000120694.19	HSPH1	0.56	1.20E-03	1.65E-02
2002	ENSG00000135926.14	TMBIM1	0.39	1.20E-03	1.65E-02
2003	ENSG00000162735.18	PEX19	0.39	1.21E-03	1.66E-02
2004	ENSG00000172053.17	QARS	0.34	1.21E-03	1.66E-02
2005	ENSG00000131831.17	RAI2	-0.70	1.21E-03	1.66E-02
2006	ENSG00000280219.1	AC093908.1	-0.72	1.21E-03	1.66E-02
2007	ENSG00000101199.12	ARFGAP1	-0.52	1.21E-03	1.67E-02
2008	ENSG00000162576.16	MXRA8	-0.42	1.22E-03	1.67E-02
2009	ENSG00000176697.18	BDNF	0.96	1.22E-03	1.68E-02
2010	ENSG00000108244.16	KRT23	-5.51	1.22E-03	1.68E-02
2011	ENSG00000185100.10	ADSSL1	0.61	1.22E-03	1.68E-02
2012	ENSG00000050628.20	PTGER3	-0.59	1.23E-03	1.69E-02
2013	ENSG00000166265.11	CYYR1	0.38	1.23E-03	1.69E-02
2014	ENSG00000182175.14	RGMA	-0.57	1.23E-03	1.69E-02
2015	ENSG00000110042.7	DTX4	-0.53	1.24E-03	1.69E-02
2016	ENSG00000137210.13	TMEM14B	0.55	1.24E-03	1.70E-02
2017	ENSG00000164056.10	SPRY1	-0.62	1.24E-03	1.70E-02
2018	ENSG00000184719.11	RNLS	0.55	1.24E-03	1.70E-02
2019	ENSG00000165521.15	EML5	-1.47	1.24E-03	1.70E-02
2020	ENSG00000105676.13	ARMC6	1.04	1.25E-03	1.70E-02
2021	ENSG00000176971.3	FIBIN	-0.99	1.25E-03	1.71E-02
2022	ENSG00000149743.13	TRPT1	0.74	1.26E-03	1.72E-02
2023	ENSG00000143995.19	MEIS1	-0.40	1.27E-03	1.73E-02
2024	ENSG00000008405.11	CRY1	0.61	1.27E-03	1.73E-02
2025	ENSG00000174233.11	ADCY6	-0.39	1.28E-03	1.75E-02
2026	ENSG00000108826.15	MRPL27	0.53	1.28E-03	1.75E-02
2027	ENSG00000120318.15	ARAP3	0.42	1.29E-03	1.75E-02
2028	ENSG00000165152.8	TMEM246	-0.99	1.29E-03	1.76E-02
2029	ENSG00000105835.11	NAMPT	0.49	1.29E-03	1.76E-02
2030	ENSG00000228340.5	MIR646HG	1.18	1.29E-03	1.76E-02
2031	ENSG00000105321.13	CCDC9	-0.38	1.30E-03	1.76E-02
2032	ENSG00000176641.10	RNF152	1.14	1.30E-03	1.76E-02
2033	ENSG00000054523.17	KIF1B	-0.34	1.30E-03	1.76E-02
2034	ENSG00000204316.12	MRPL38	0.63	1.30E-03	1.76E-02
2035	ENSG00000283201.1	AC092329.3	-2.42	1.30E-03	1.76E-02
2036	ENSG00000084734.8	GCKR	1.66	1.30E-03	1.77E-02
2037	ENSG00000152932.7	RAB3C	-0.98	1.31E-03	1.77E-02
2038	ENSG00000070778.12	PTPN21	-0.49	1.31E-03	1.77E-02
2039	ENSG00000159348.12	CYB5R1	0.46	1.31E-03	1.77E-02
2040	ENSG00000164338.9	UTP15	0.55	1.31E-03	1.78E-02
2041	ENSG00000117500.12	TMED5	0.43	1.31E-03	1.78E-02
2042	ENSG00000120837.7	NFYB	-0.38	1.32E-03	1.78E-02
2043	ENSG00000234492.4	RPL34-AS1	-0.83	1.32E-03	1.78E-02
2044	ENSG00000134201.11	GSTM5	-1.05	1.33E-03	1.79E-02
2045	ENSG00000166348.18	USP54	0.50	1.33E-03	1.80E-02
2046	ENSG00000229308.1	AC010737.1	3.03	1.34E-03	1.80E-02
2047	ENSG00000187866.8	FAM122A	0.38	1.35E-03	1.81E-02

2048	ENSG00000196189.12	SEMA4A	-1.34	1.35E-03	1.81E-02
2049	ENSG00000169604.19	ANTXR1	-0.45	1.35E-03	1.82E-02
2050	ENSG00000185610.6	DBX2	2.30	1.35E-03	1.82E-02
2051	ENSG00000140284.10	SLC27A2	1.92	1.35E-03	1.82E-02
2052	ENSG00000126653.17	NSRP1	-0.32	1.35E-03	1.82E-02
2053	ENSG00000181234.9	TMEM132C	-0.60	1.35E-03	1.82E-02
2054	ENSG00000258698.1	AL139317.1	-2.45	1.35E-03	1.82E-02
2055	ENSG00000261578.1	AP003119.3	2.00	1.36E-03	1.82E-02
2056	ENSG00000247828.7	TMEM161B-AS1	0.59	1.37E-03	1.83E-02
2057	ENSG00000168038.10	ULK4	-0.50	1.37E-03	1.84E-02
2058	ENSG00000179399.14	GPC5	3.83	1.37E-03	1.84E-02
2059	ENSG00000134480.14	CCNH	0.39	1.37E-03	1.84E-02
2060	ENSG00000211899.10	IGHM	2.48	1.38E-03	1.84E-02
2061	ENSG00000166855.9	CLPX	0.37	1.38E-03	1.84E-02
2062	ENSG00000134970.13	TMED7	0.30	1.38E-03	1.84E-02
2063	ENSG00000137364.4	TPMT	0.53	1.38E-03	1.85E-02
2064	ENSG00000196155.12	PLEKHG4	-0.79	1.38E-03	1.85E-02
2065	ENSG00000118777.11	ABCG2	-1.18	1.39E-03	1.86E-02
2066	ENSG00000035141.7	FAM136A	0.42	1.40E-03	1.87E-02
2067	ENSG00000110200.8	ANAPC15	0.51	1.40E-03	1.87E-02
2068	ENSG00000243345.1	AC093714.2	-1.02	1.41E-03	1.88E-02
2069	ENSG00000144730.17	IL17RD	-0.73	1.41E-03	1.88E-02
2070	ENSG00000165832.5	TRUB1	0.65	1.41E-03	1.88E-02
2071	ENSG00000173548.8	SNX33	-0.47	1.41E-03	1.88E-02
2072	ENSG00000248774.1	AC097534.1	-3.73	1.41E-03	1.88E-02
2073	ENSG00000171720.9	HDAC3	-0.36	1.41E-03	1.88E-02
2074	ENSG00000235205.1	TATDN2P3	-1.17	1.41E-03	1.88E-02
2075	ENSG00000127191.17	TRAF2	0.72	1.41E-03	1.88E-02
2076	ENSG00000168079.16	SCARA5	-0.71	1.42E-03	1.88E-02
2077	ENSG00000153814.12	JAZF1	-0.44	1.42E-03	1.89E-02
2078	ENSG00000198753.11	PLXNB3	-1.44	1.42E-03	1.89E-02
2079	ENSG00000179776.18	CDH5	0.40	1.42E-03	1.89E-02
2080	ENSG00000147027.3	TMEM47	0.34	1.43E-03	1.89E-02
2081	ENSG00000165272.15	AQP3	-1.14	1.43E-03	1.89E-02
2082	ENSG00000102910.13	LONP2	0.30	1.43E-03	1.89E-02
2083	ENSG00000237844.1	AC016766.1	-1.31	1.43E-03	1.89E-02
2084	ENSG00000158859.9	ADAMTS4	0.64	1.43E-03	1.90E-02
2085	ENSG00000237094.12	AL732372.2	0.51	1.43E-03	1.90E-02
2086	ENSG00000108406.9	DHX40	-0.36	1.43E-03	1.90E-02
2087	ENSG00000148459.15	PDSS1	1.06	1.43E-03	1.90E-02
2088	ENSG00000235505.7	CASP17P	-0.60	1.43E-03	1.90E-02
2089	ENSG00000075420.12	FNDC3B	-0.42	1.44E-03	1.90E-02
2090	ENSG00000279168.2	AC105052.4	-1.04	1.44E-03	1.90E-02
2091	ENSG00000090020.10	SLC9A1	0.49	1.44E-03	1.90E-02
2092	ENSG00000163435.15	ELF3	-1.83	1.44E-03	1.91E-02
2093	ENSG00000111639.7	MRPL51	0.42	1.45E-03	1.91E-02
2094	ENSG00000050405.13	LIMA1	-0.35	1.46E-03	1.92E-02
2095	ENSG00000082438.15	COBLL1	-0.53	1.46E-03	1.92E-02
2096	ENSG00000146147.14	MLIP	1.26	1.46E-03	1.92E-02
2097	ENSG00000134352.19	IL6ST	-0.34	1.46E-03	1.92E-02

2098	ENSG00000112893.9	MAN2A1	-0.39	1.46E-03	1.92E-02
2099	ENSG00000120051.14	CFAP58	1.14	1.47E-03	1.93E-02
2100	ENSG00000183715.13	OPCML	2.34	1.47E-03	1.93E-02
2101	ENSG00000167671.11	UBXN6	0.39	1.47E-03	1.93E-02
2102	ENSG00000168016.14	TRANK1	-0.50	1.47E-03	1.93E-02
2103	ENSG00000260947.1	AL356489.2	-0.75	1.47E-03	1.93E-02
2104	ENSG00000243696.4	AC006254.1	1.18	1.47E-03	1.93E-02
2105	ENSG00000120438.11	TCP1	0.38	1.48E-03	1.94E-02
2106	ENSG00000136813.14	ECPAS	0.30	1.48E-03	1.94E-02
2107	ENSG00000152953.12	STK32B	-0.85	1.49E-03	1.95E-02
2108	ENSG00000181826.9	RELL1	-0.54	1.49E-03	1.96E-02
2109	ENSG00000122970.15	IFT81	-0.41	1.50E-03	1.96E-02
2110	ENSG00000143164.15	DCAF6	0.35	1.50E-03	1.96E-02
2111	ENSG00000211829.9	TRDC	-1.66	1.50E-03	1.96E-02
2112	ENSG00000105649.9	RAB3A	1.53	1.50E-03	1.96E-02
2113	ENSG00000171174.14	RBKS	0.68	1.51E-03	1.97E-02
2114	ENSG00000117519.15	CNN3	-0.39	1.51E-03	1.97E-02
2115	ENSG00000121068.13	TBX2	0.68	1.51E-03	1.97E-02
2116	ENSG00000217930.7	PAM16	0.67	1.52E-03	1.98E-02
2117	ENSG00000144560.14	VGLL4	0.45	1.53E-03	1.99E-02
2118	ENSG00000111652.9	COPS7A	0.36	1.53E-03	2.00E-02
2119	ENSG00000084731.14	KIF3C	-0.67	1.53E-03	2.00E-02
2120	ENSG00000230445.4	LRRC37A6P	1.18	1.55E-03	2.02E-02
2121	ENSG00000164402.13	SEPT8	-0.41	1.56E-03	2.02E-02
2122	ENSG00000007392.16	LUC7L	-0.35	1.56E-03	2.03E-02
2123	ENSG00000147257.13	GPC3	-0.69	1.56E-03	2.03E-02
2124	ENSG00000114019.14	AMOTL2	-0.61	1.56E-03	2.03E-02
2125	ENSG00000107242.17	PIP5K1B	1.71	1.56E-03	2.03E-02
2126	ENSG00000084093.16	REST	0.51	1.57E-03	2.03E-02
2127	ENSG00000161526.14	SAP30BP	-0.33	1.57E-03	2.03E-02
2128	ENSG00000258545.5	RHOXF1-AS1	0.65	1.57E-03	2.03E-02
2129	ENSG00000114770.16	ABCC5	0.35	1.57E-03	2.04E-02
2130	ENSG00000174109.4	C16orf91	0.71	1.58E-03	2.04E-02
2131	ENSG00000159216.18	RUNX1	-0.73	1.58E-03	2.05E-02
2132	ENSG00000090863.11	GLG1	-0.33	1.59E-03	2.05E-02
2133	ENSG00000076706.16	MCAM	-0.37	1.59E-03	2.05E-02
2134	ENSG00000219314.1	AL034372.1	3.06	1.59E-03	2.05E-02
2135	ENSG00000173918.14	C1QTNF1	-0.59	1.60E-03	2.06E-02
2136	ENSG00000166681.13	BEX3	-0.42	1.60E-03	2.06E-02
2137	ENSG00000034693.14	PEX3	0.50	1.60E-03	2.07E-02
2138	ENSG00000107566.13	ERLIN1	-0.44	1.60E-03	2.07E-02
2139	ENSG00000101282.8	RSPO4	-2.90	1.61E-03	2.07E-02
2140	ENSG00000110697.12	PITPNM1	1.07	1.61E-03	2.08E-02
2141	ENSG00000230551.4	AC021078.1	0.28	1.61E-03	2.08E-02
2142	ENSG00000107771.16	CCSER2	0.30	1.62E-03	2.08E-02
2143	ENSG00000223572.9	CKMT1A	5.04	1.62E-03	2.08E-02
2144	ENSG00000198400.11	NTRK1	0.97	1.62E-03	2.08E-02
2145	ENSG00000261240.1	AC009065.6	-0.71	1.62E-03	2.08E-02
2146	ENSG00000100312.10	ACR	-1.75	1.62E-03	2.08E-02
2147	ENSG00000138771.15	SHROOM3	-1.24	1.62E-03	2.09E-02

2148	ENSG00000110171.19	TRIM3	-0.43	1.63E-03	2.10E-02
2149	ENSG00000064545.14	TMEM161A	0.71	1.64E-03	2.10E-02
2150	ENSG00000156735.10	BAG4	-0.45	1.63E-03	2.10E-02
2151	ENSG00000077616.10	NAALAD2	-0.68	1.64E-03	2.10E-02
2152	ENSG00000160683.4	CXCR5	-0.93	1.64E-03	2.10E-02
2153	ENSG00000104324.15	CPQ	-0.37	1.65E-03	2.12E-02
2154	ENSG00000009335.17	UBE3C	0.30	1.66E-03	2.12E-02
2155	ENSG00000138073.13	PREB	0.46	1.67E-03	2.14E-02
2156	ENSG00000144867.11	SRPRB	0.48	1.68E-03	2.14E-02
2157	ENSG00000139329.4	LUM	-0.54	1.68E-03	2.15E-02
2158	ENSG00000237580.1	GCSHP3	1.50	1.69E-03	2.15E-02
2159	ENSG00000142207.6	URB1	0.43	1.69E-03	2.16E-02
2160	ENSG00000270504.1	AL391422.4	-0.61	1.69E-03	2.16E-02
2161	ENSG00000185189.17	NRBP2	-0.38	1.70E-03	2.17E-02
2162	ENSG00000136718.9	IMP4	0.35	1.70E-03	2.17E-02
2163	ENSG00000279378.1	AC009159.4	-1.64	1.71E-03	2.18E-02
2164	ENSG00000196656.7	AC004057.1	0.79	1.71E-03	2.18E-02
2165	ENSG00000247556.6	OIP5-AS1	0.31	1.71E-03	2.18E-02
2166	ENSG00000185561.9	TLCD2	-0.63	1.71E-03	2.18E-02
2167	ENSG00000124225.15	PMEPA1	-0.67	1.71E-03	2.18E-02
2168	ENSG00000108946.14	PRKAR1A	0.36	1.72E-03	2.18E-02
2169	ENSG00000102996.4	MMP15	0.59	1.72E-03	2.19E-02
2170	ENSG00000262112.1	AC015912.1	-1.28	1.74E-03	2.21E-02
2171	ENSG00000049239.12	H6PD	-0.32	1.75E-03	2.22E-02
2172	ENSG00000204539.3	CDSN	-3.10	1.75E-03	2.22E-02
2173	ENSG00000120915.13	EPHX2	0.47	1.77E-03	2.24E-02
2174	ENSG00000139687.15	RB1	0.33	1.76E-03	2.24E-02
2175	ENSG00000170745.11	KCNS3	-0.90	1.76E-03	2.24E-02
2176	ENSG00000203783.4	PRR9	-3.80	1.76E-03	2.24E-02
2177	ENSG00000253741.1	LNCOC1	1.28	1.77E-03	2.24E-02
2178	ENSG00000265727.2	RN7SL648P	-0.55	1.77E-03	2.24E-02
2179	ENSG00000237984.3	PTENP1	-1.43	1.78E-03	2.25E-02
2180	ENSG00000211456.10	SACM1L	0.33	1.79E-03	2.26E-02
2181	ENSG00000164683.16	HEY1	0.52	1.79E-03	2.27E-02
2182	ENSG00000135931.17	ARMC9	-0.54	1.79E-03	2.27E-02
2183	ENSG00000166450.12	PRTG	0.63	1.81E-03	2.28E-02
2184	ENSG00000275832.4	ARHGAP23	-0.45	1.81E-03	2.28E-02
2185	ENSG00000132613.14	MTSS1L	0.55	1.81E-03	2.29E-02
2186	ENSG00000177885.14	GRB2	0.32	1.81E-03	2.29E-02
2187	ENSG00000141985.9	SH3GL1	-0.46	1.81E-03	2.29E-02
2188	ENSG00000230937.11	MIR205HG	-3.22	1.82E-03	2.29E-02
2189	ENSG00000164163.10	ABCE1	0.41	1.83E-03	2.30E-02
2190	ENSG00000204219.10	TCEA3	0.56	1.83E-03	2.30E-02
2191	ENSG00000214253.8	FIS1	0.37	1.83E-03	2.31E-02
2192	ENSG00000112183.14	RBM24	1.05	1.84E-03	2.31E-02
2193	ENSG00000196482.16	ESRRG	2.06	1.84E-03	2.31E-02
2194	ENSG00000047849.21	MAP4	-0.40	1.84E-03	2.31E-02
2195	ENSG00000104381.12	GDAP1	-0.70	1.84E-03	2.31E-02
2196	ENSG00000146066.2	HIGD2A	0.71	1.86E-03	2.34E-02
2197	ENSG00000166750.9	SLFN5	-0.44	1.86E-03	2.34E-02

2198	ENSG00000137509.10	PRCP	0.37	1.87E-03	2.34E-02
2199	ENSG00000061273.17	HDAC7	-0.43	1.87E-03	2.34E-02
2200	ENSG00000180357.9	ZNF609	-0.30	1.87E-03	2.34E-02
2201	ENSG00000020922.12	MRE11	-0.38	1.89E-03	2.37E-02
2202	ENSG00000235609.7	AF127577.4	-0.74	1.89E-03	2.37E-02
2203	ENSG00000228536.2	AL513283.1	-1.13	1.90E-03	2.38E-02
2204	ENSG00000273148.1	AL035563.1	-1.00	1.91E-03	2.39E-02
2205	ENSG00000237596.6	AL138828.1	0.82	1.92E-03	2.40E-02
2206	ENSG00000138834.12	MAPK8IP3	-0.37	1.92E-03	2.41E-02
2207	ENSG00000140044.12	JDP2	-0.72	1.93E-03	2.41E-02
2208	ENSG00000113249.12	HAVCR1	1.66	1.94E-03	2.43E-02
2209	ENSG00000267528.1	AC008991.1	2.08	1.95E-03	2.43E-02
2210	ENSG00000126603.8	GLIS2	-0.77	1.95E-03	2.43E-02
2211	ENSG00000176919.12	C8G	3.36	1.96E-03	2.45E-02
2212	ENSG00000138835.22	RGS3	0.59	1.96E-03	2.45E-02
2213	ENSG00000135929.8	CYP27A1	-0.57	1.96E-03	2.45E-02
2214	ENSG00000136950.13	ARPC5L	0.56	1.98E-03	2.46E-02
2215	ENSG00000142279.12	WTIP	-0.35	1.98E-03	2.46E-02
2216	ENSG00000133193.12	FAM104A	-0.42	1.98E-03	2.46E-02
2217	ENSG00000165886.4	UBTD1	-0.76	1.98E-03	2.46E-02
2218	ENSG00000138134.11	STAMBPL1	-0.78	1.98E-03	2.46E-02
2219	ENSG00000113805.8	CNTN3	-0.84	1.98E-03	2.46E-02
2220	ENSG00000267121.5	AC008105.3	-0.85	1.98E-03	2.46E-02
2221	ENSG00000048828.16	FAM120A	0.33	1.99E-03	2.47E-02
2222	ENSG00000243319.7	FGF14-IT1	-1.83	1.99E-03	2.47E-02
2223	ENSG00000149633.11	KIAA1755	1.08	1.99E-03	2.47E-02
2224	ENSG00000163689.20	C3orf67	-1.28	1.99E-03	2.47E-02
2225	ENSG00000250295.6	RDH10-AS1	1.05	2.01E-03	2.49E-02
2226	ENSG00000272150.5	NBPF25P	-0.52	2.01E-03	2.49E-02
2227	ENSG00000257429.1	AC074031.1	-3.54	2.01E-03	2.49E-02
2228	ENSG00000178074.5	C2orf69	0.41	2.02E-03	2.50E-02
2229	ENSG00000007171.17	NOS2	2.26	2.02E-03	2.50E-02
2230	ENSG00000251194.2	AL133330.1	-0.94	2.03E-03	2.51E-02
2231	ENSG00000134278.15	SPIRE1	0.53	2.03E-03	2.51E-02
2232	ENSG00000160194.17	NDUFV3	0.36	2.04E-03	2.52E-02
2233	ENSG00000111186.12	WNT5B	-0.82	2.04E-03	2.52E-02
2234	ENSG00000270713.1	AL132994.2	2.04	2.04E-03	2.52E-02
2235	ENSG00000236065.2	AL020995.1	1.27	2.04E-03	2.52E-02
2236	ENSG00000124615.19	MOCS1	0.75	2.05E-03	2.53E-02
2237	ENSG00000186458.4	DEFB132	-2.26	2.05E-03	2.53E-02
2238	ENSG00000008130.15	NADK	0.39	2.06E-03	2.53E-02
2239	ENSG00000197345.12	MRPL21	0.78	2.06E-03	2.54E-02
2240	ENSG00000135972.8	MRPS9	0.60	2.08E-03	2.56E-02
2241	ENSG00000123739.10	PLA2G12A	0.37	2.08E-03	2.56E-02
2242	ENSG00000173950.15	XXYLT1	-0.71	2.08E-03	2.56E-02
2243	ENSG00000125675.17	GRIA3	-0.99	2.08E-03	2.56E-02
2244	ENSG00000189283.9	FHIT	0.89	2.08E-03	2.56E-02
2245	ENSG00000205707.10	ETFRF1	0.58	2.09E-03	2.57E-02
2246	ENSG00000049323.15	LTBP1	0.30	2.09E-03	2.57E-02
2247	ENSG00000226853.2	AC010894.2	2.02	2.10E-03	2.58E-02

2248	ENSG00000279419.1	AC004925.1	1.77	2.10E-03	2.58E-02
2249	ENSG00000011295.15	TTC19	0.38	2.11E-03	2.58E-02
2250	ENSG00000213085.9	CFAP45	-1.94	2.11E-03	2.59E-02
2251	ENSG00000117122.13	MFAP2	-1.51	2.12E-03	2.60E-02
2252	ENSG00000090565.15	RAB11FIP3	-0.43	2.13E-03	2.61E-02
2253	ENSG00000229593.1	SUCLA2P3	4.11	2.14E-03	2.62E-02
2254	ENSG00000214367.7	HAUS3	-0.32	2.14E-03	2.62E-02
2255	ENSG00000147533.16	GOLGA7	0.32	2.14E-03	2.62E-02
2256	ENSG00000157680.15	DGKI	-0.84	2.14E-03	2.62E-02
2257	ENSG00000128989.10	ARPP19	0.38	2.14E-03	2.62E-02
2258	ENSG00000170577.7	SIX2	1.07	2.15E-03	2.63E-02
2259	ENSG00000203797.9	DDO	1.01	2.15E-03	2.63E-02
2260	ENSG00000109861.15	CTSC	-0.41	2.15E-03	2.63E-02
2261	ENSG00000196368.4	NUDT11	-2.00	2.15E-03	2.63E-02
2262	ENSG00000269110.1	AC010636.2	-3.88	2.15E-03	2.63E-02
2263	ENSG00000132819.16	RBM38	0.92	2.16E-03	2.63E-02
2264	ENSG00000167614.13	TTYH1	-0.88	2.16E-03	2.63E-02
2265	ENSG00000166575.16	TMEM135	0.44	2.17E-03	2.64E-02
2266	ENSG00000176435.6	CLEC14A	0.36	2.17E-03	2.64E-02
2267	ENSG00000125166.12	GOT2	0.56	2.18E-03	2.65E-02
2268	ENSG00000187079.16	TEAD1	-0.43	2.18E-03	2.66E-02
2269	ENSG00000150593.17	PDCD4	-0.58	2.19E-03	2.66E-02
2270	ENSG00000224892.6	RPS4XP16	1.54	2.19E-03	2.66E-02
2271	ENSG00000275897.1	AC021491.4	1.32	2.19E-03	2.66E-02
2272	ENSG00000274312.1	AC015722.2	-3.05	2.19E-03	2.67E-02
2273	ENSG00000197157.10	SND1	-0.33	2.20E-03	2.67E-02
2274	ENSG00000102760.12	RGCC	0.55	2.20E-03	2.67E-02
2275	ENSG00000187164.19	SHTN1	-0.56	2.22E-03	2.70E-02
2276	ENSG00000147471.11	PLPBP	0.37	2.23E-03	2.70E-02
2277	ENSG00000156831.7	NSMCE2	-0.43	2.24E-03	2.71E-02
2278	ENSG00000256087.6	ZNF432	-0.50	2.24E-03	2.72E-02
2279	ENSG00000087111.20	PIGS	-0.46	2.25E-03	2.72E-02
2280	ENSG00000179820.15	MYADM	-0.49	2.25E-03	2.72E-02
2281	ENSG00000260369.2	AC120024.1	-0.98	2.26E-03	2.73E-02
2282	ENSG00000230102.7	LINC02028	1.38	2.26E-03	2.73E-02
2283	ENSG00000261220.2	AC103706.1	1.45	2.27E-03	2.74E-02
2284	ENSG00000064225.12	ST3GAL6	-0.45	2.27E-03	2.74E-02
2285	ENSG00000198728.10	LDB1	-0.47	2.27E-03	2.74E-02
2286	ENSG00000223482.7	NUTM2A-AS1	0.40	2.28E-03	2.76E-02
2287	ENSG00000224531.5	SMIM13	0.44	2.29E-03	2.76E-02
2288	ENSG00000181322.13	NME9	-0.54	2.29E-03	2.77E-02
2289	ENSG00000285815.1	WRB-SH3BGR	3.39	2.30E-03	2.77E-02
2290	ENSG00000147475.15	ERLIN2	0.31	2.30E-03	2.78E-02
2291	ENSG00000103449.11	SALL1	-2.24	2.31E-03	2.78E-02
2292	ENSG00000285977.1	AC022068.1	2.29	2.31E-03	2.78E-02
2293	ENSG00000163930.9	BAP1	0.28	2.32E-03	2.80E-02
2294	ENSG00000166924.8	NYAP1	-1.53	2.33E-03	2.80E-02
2295	ENSG00000070814.19	TCOF1	-0.36	2.33E-03	2.81E-02
2296	ENSG00000138629.15	UBL7	0.54	2.34E-03	2.81E-02
2297	ENSG00000138823.13	MTTP	1.41	2.34E-03	2.81E-02

2298	ENSG00000165487.13	MICU2	0.47	2.34E-03	2.81E-02
2299	ENSG00000243649.8	CFB	-0.74	2.38E-03	2.86E-02
2300	ENSG00000063660.8	GPC1	0.48	2.38E-03	2.86E-02
2301	ENSG00000169223.14	LMAN2	0.31	2.39E-03	2.86E-02
2302	ENSG00000102409.9	BEX4	-0.48	2.40E-03	2.88E-02
2303	ENSG00000182224.11	CYB5D1	0.47	2.41E-03	2.88E-02
2304	ENSG00000124164.15	VAPB	0.24	2.41E-03	2.88E-02
2305	ENSG00000163393.12	SLC22A15	1.20	2.41E-03	2.88E-02
2306	ENSG00000131148.8	EMC8	0.48	2.41E-03	2.89E-02
2307	ENSG00000154198.14	CYP4Z2P	-1.00	2.42E-03	2.89E-02
2308	ENSG00000088179.8	PTPN4	-0.29	2.43E-03	2.90E-02
2309	ENSG00000259343.6	TMC3-AS1	-1.75	2.43E-03	2.90E-02
2310	ENSG00000150768.15	DLAT	1.04	2.43E-03	2.90E-02
2311	ENSG00000166927.12	MS4A7	0.84	2.44E-03	2.91E-02
2312	ENSG00000143977.13	SNRPG	0.47	2.45E-03	2.92E-02
2313	ENSG00000253326.2	AL606534.4	3.25	2.46E-03	2.93E-02
2314	ENSG00000075223.13	SEMA3C	-0.66	2.47E-03	2.95E-02
2315	ENSG00000165996.13	HACD1	0.68	2.47E-03	2.95E-02
2316	ENSG00000111907.20	TPD52L1	0.54	2.48E-03	2.96E-02
2317	ENSG00000168542.14	COL3A1	-0.67	2.49E-03	2.96E-02
2318	ENSG00000271425.7	NBPF10	-0.41	2.50E-03	2.97E-02
2319	ENSG00000048392.11	RRM2B	0.39	2.50E-03	2.97E-02
2320	ENSG00000161203.13	AP2M1	-0.29	2.50E-03	2.97E-02
2321	ENSG00000163611.11	SPICE1	-0.44	2.50E-03	2.98E-02
2322	ENSG00000067064.11	IDI1	1.05	2.51E-03	2.98E-02
2323	ENSG00000233002.2	AC005324.1	-4.97	2.51E-03	2.99E-02
2324	ENSG00000077782.20	FGFR1	-0.33	2.52E-03	2.99E-02
2325	ENSG00000102034.16	ELF4	-0.51	2.52E-03	2.99E-02
2326	ENSG00000006756.15	ARSD	0.57	2.53E-03	2.99E-02
2327	ENSG00000167395.10	ZNF646	-0.35	2.53E-03	2.99E-02
2328	ENSG00000147485.12	PXDNL	1.24	2.53E-03	3.00E-02
2329	ENSG00000109686.17	SH3D19	-0.45	2.53E-03	3.00E-02
2330	ENSG00000197859.9	ADAMTSL2	1.46	2.54E-03	3.01E-02
2331	ENSG00000088836.13	SLC4A11	-2.84	2.56E-03	3.03E-02
2332	ENSG00000227372.12	TP73-AS1	-0.30	2.58E-03	3.05E-02
2333	ENSG00000124126.13	PREX1	0.54	2.58E-03	3.05E-02
2334	ENSG00000127948.15	POR	0.49	2.60E-03	3.07E-02
2335	ENSG00000253857.1	AC022679.1	3.53	2.61E-03	3.08E-02
2336	ENSG00000101251.12	SEL1L2	1.39	2.60E-03	3.08E-02
2337	ENSG00000178033.5	CALHM5	0.69	2.61E-03	3.08E-02
2338	ENSG00000124067.16	SLC12A4	-0.38	2.61E-03	3.08E-02
2339	ENSG00000119714.10	GPR68	-0.93	2.61E-03	3.08E-02
2340	ENSG00000137076.20	TLN1	-0.43	2.62E-03	3.09E-02
2341	ENSG00000119280.16	C1orf198	-0.71	2.63E-03	3.10E-02
2342	ENSG00000064601.18	CTSA	0.40	2.63E-03	3.10E-02
2343	ENSG00000100897.17	DCAF11	0.41	2.64E-03	3.11E-02
2344	ENSG00000013588.8	GPRC5A	-0.69	2.64E-03	3.11E-02
2345	ENSG00000211592.8	IGKC	3.40	2.65E-03	3.11E-02
2346	ENSG00000198589.11	LRBA	0.31	2.65E-03	3.11E-02
2347	ENSG00000143816.7	WNT9A	-1.05	2.65E-03	3.11E-02

2348	ENSG00000148290.9	SURF1	0.74	2.65E-03	3.11E-02
2349	ENSG00000153339.13	TRAPPC8	0.28	2.66E-03	3.12E-02
2350	ENSG00000072657.8	TRHDE	-0.56	2.66E-03	3.12E-02
2351	ENSG00000156103.15	MMP16	-0.73	2.66E-03	3.12E-02
2352	ENSG00000147654.14	EBAG9	0.46	2.67E-03	3.13E-02
2353	ENSG00000137288.9	UQCC2	0.55	2.67E-03	3.13E-02
2354	ENSG00000114779.19	ABHD14B	0.49	2.68E-03	3.14E-02
2355	ENSG00000156170.12	NDUFAF6	0.47	2.68E-03	3.14E-02
2356	ENSG00000109670.14	FBXW7	-0.37	2.68E-03	3.14E-02
2357	ENSG00000237125.9	HAND2-AS1	-0.43	2.69E-03	3.15E-02
2358	ENSG00000182575.7	NXPH3	-0.81	2.70E-03	3.16E-02
2359	ENSG00000277972.1	CISD3	0.62	2.71E-03	3.17E-02
2360	ENSG00000141905.18	NFIC	-0.31	2.73E-03	3.19E-02
2361	ENSG00000122870.11	BICC1	-0.68	2.73E-03	3.19E-02
2362	ENSG00000169230.9	PRELID1	0.34	2.73E-03	3.19E-02
2363	ENSG00000166226.12	CCT2	0.38	2.75E-03	3.21E-02
2364	ENSG00000174808.11	BTC	-0.87	2.76E-03	3.22E-02
2365	ENSG00000110237.4	ARHGEF17	-0.40	2.76E-03	3.22E-02
2366	ENSG00000256654.3	AC005906.2	-1.66	2.76E-03	3.22E-02
2367	ENSG00000145979.17	TBC1D7	0.50	2.77E-03	3.23E-02
2368	ENSG00000243075.3	RN7SL519P	-1.33	2.77E-03	3.23E-02
2369	ENSG00000173253.15	DMRT2	-1.32	2.79E-03	3.25E-02
2370	ENSG00000187678.9	SPRY4	0.54	2.80E-03	3.26E-02
2371	ENSG00000075035.9	WSCD2	-0.78	2.80E-03	3.26E-02
2372	ENSG00000182534.13	MXRA7	-0.56	2.80E-03	3.26E-02
2373	ENSG00000169026.12	SLC49A3	0.93	2.82E-03	3.27E-02
2374	ENSG00000168350.7	DEGS2	1.47	2.83E-03	3.29E-02
2375	ENSG00000119913.5	TECTB	3.29	2.84E-03	3.29E-02
2376	ENSG00000100335.14	MIEF1	0.43	2.84E-03	3.29E-02
2377	ENSG00000132507.17	EIF5A	0.28	2.84E-03	3.29E-02
2378	ENSG00000161638.10	ITGA5	-0.45	2.84E-03	3.29E-02
2379	ENSG00000139946.9	PELI2	-0.45	2.84E-03	3.29E-02
2380	ENSG00000162869.15	PPP1R21	-0.37	2.85E-03	3.30E-02
2381	ENSG00000151150.21	ANK3	0.82	2.86E-03	3.31E-02
2382	ENSG00000110074.10	FOXRED1	0.53	2.88E-03	3.33E-02
2383	ENSG00000113583.7	C5orf15	0.48	2.88E-03	3.33E-02
2384	ENSG00000250548.6	LINC01303	1.72	2.88E-03	3.33E-02
2385	ENSG00000153707.16	PTPRD	0.84	2.88E-03	3.34E-02
2386	ENSG00000092847.11	AGO1	-0.31	2.89E-03	3.34E-02
2387	ENSG00000242268.2	LINC02082	1.39	2.89E-03	3.34E-02
2388	ENSG00000198920.9	KIAA0753	-0.42	2.90E-03	3.35E-02
2389	ENSG00000279518.1	AC083843.3	0.87	2.90E-03	3.35E-02
2390	ENSG00000151461.19	UPF2	-0.29	2.90E-03	3.35E-02
2391	ENSG00000157881.13	PANK4	0.44	2.91E-03	3.36E-02
2392	ENSG00000138061.11	CYP1B1	0.66	2.92E-03	3.36E-02
2393	ENSG00000129009.12	ISLR	-0.43	2.92E-03	3.36E-02
2394	ENSG00000123892.11	RAB38	-2.48	2.92E-03	3.37E-02
2395	ENSG00000019995.6	ZRANB1	0.39	2.92E-03	3.37E-02
2396	ENSG00000163659.12	TIPARP	0.68	2.93E-03	3.37E-02
2397	ENSG00000173409.13	ARV1	0.60	2.93E-03	3.37E-02

2398	ENSG00000156599.10	ZDHHC5	0.25	2.93E-03	3.37E-02
2399	ENSG00000215251.3	FASTKD5	0.74	2.94E-03	3.38E-02
2400	ENSG00000161558.10	TMEM143	0.70	2.95E-03	3.39E-02
2401	ENSG00000179023.8	KLHDC7A	3.09	2.95E-03	3.39E-02
2402	ENSG00000140577.15	CRTC3	-0.42	2.95E-03	3.39E-02
2403	ENSG00000121207.11	LRAT	1.08	2.98E-03	3.42E-02
2404	ENSG00000086061.15	DNAJA1	0.48	2.98E-03	3.42E-02
2405	ENSG00000214671.4	RPL6P12	-2.24	2.98E-03	3.42E-02
2406	ENSG00000173598.13	NUDT4	-0.39	3.00E-03	3.44E-02
2407	ENSG00000068400.13	GRIPAP1	-0.29	3.02E-03	3.46E-02
2408	ENSG00000089123.15	TASP1	-0.45	3.02E-03	3.46E-02
2409	ENSG00000151834.15	GABRA2	-0.56	3.02E-03	3.46E-02
2410	ENSG00000224328.1	MDC1-AS1	-0.79	3.02E-03	3.46E-02
2411	ENSG00000167700.8	MFSD3	1.04	3.05E-03	3.49E-02
2412	ENSG00000198740.8	ZNF652	-0.32	3.06E-03	3.50E-02
2413	ENSG00000172236.16	TPSAB1	-2.11	3.07E-03	3.51E-02
2414	ENSG00000270673.1	YTHDF3-AS1	-3.55	3.08E-03	3.51E-02
2415	ENSG00000171346.15	KRT15	-3.72	3.08E-03	3.51E-02
2416	ENSG00000110756.17	HPS5	0.39	3.08E-03	3.52E-02
2417	ENSG00000180537.12	RNF182	1.58	3.09E-03	3.52E-02
2418	ENSG00000163617.10	CCDC191	-0.61	3.08E-03	3.52E-02
2419	ENSG00000103174.12	NAGPA	-0.69	3.09E-03	3.52E-02
2420	ENSG00000005961.18	ITGA2B	-1.03	3.09E-03	3.52E-02
2421	ENSG00000068781.21	STON1-GTF2A1L	-1.84	3.08E-03	3.52E-02
2422	ENSG00000142168.14	SOD1	0.31	3.09E-03	3.52E-02
2423	ENSG00000112685.13	EXOC2	0.39	3.09E-03	3.52E-02
2424	ENSG00000116209.11	TMEM59	0.28	3.10E-03	3.53E-02
2425	ENSG00000141504.11	SAT2	0.41	3.11E-03	3.53E-02
2426	ENSG00000109756.9	RAPGEF2	0.40	3.11E-03	3.54E-02
2427	ENSG00000117114.19	ADGRL2	-0.38	3.12E-03	3.54E-02
2428	ENSG00000076003.4	MCM6	-0.43	3.12E-03	3.54E-02
2429	ENSG00000115415.18	STAT1	-0.49	3.12E-03	3.54E-02
2430	ENSG00000177990.11	DPY19L2	-0.63	3.12E-03	3.54E-02
2431	ENSG00000196208.13	GREB1	-1.25	3.12E-03	3.54E-02
2432	ENSG00000154127.9	UBASH3B	1.01	3.12E-03	3.54E-02
2433	ENSG00000148343.18	MIGA2	0.48	3.12E-03	3.54E-02
2434	ENSG00000157214.13	STEAP2	0.53	3.13E-03	3.55E-02
2435	ENSG00000266010.2	GATA6-AS1	1.97	3.14E-03	3.55E-02
2436	ENSG00000188760.10	TMEM198	-1.62	3.14E-03	3.56E-02
2437	ENSG00000198547.8	C20orf203	-1.46	3.14E-03	3.56E-02
2438	ENSG00000253304.1	TMEM200B	-0.58	3.15E-03	3.57E-02
2439	ENSG00000136026.13	CKAP4	-0.33	3.16E-03	3.57E-02
2440	ENSG00000130402.11	ACTN4	-0.33	3.16E-03	3.57E-02
2441	ENSG00000237161.4	AC068446.1	-0.93	3.16E-03	3.57E-02
2442	ENSG00000112245.11	PTP4A1	0.53	3.17E-03	3.58E-02
2443	ENSG00000122642.10	FKBP9	-0.33	3.19E-03	3.60E-02
2444	ENSG00000135469.13	COQ10A	0.63	3.19E-03	3.61E-02
2445	ENSG00000227225.1	MTND1P14	-3.10	3.20E-03	3.61E-02
2446	ENSG00000143799.12	PARP1	0.31	3.21E-03	3.62E-02
2447	ENSG00000148737.16	TCF7L2	-0.56	3.23E-03	3.64E-02

2448	ENSG00000197989.14	SNHG12	-0.49	3.24E-03	3.65E-02
2449	ENSG00000279885.1	AP005060.1	-0.56	3.24E-03	3.65E-02
2450	ENSG00000061455.10	PRDM6	1.10	3.25E-03	3.66E-02
2451	ENSG00000100591.7	AHSA1	0.37	3.26E-03	3.67E-02
2452	ENSG00000106479.10	ZNF862	-0.38	3.26E-03	3.67E-02
2453	ENSG00000175334.7	BANF1	0.41	3.27E-03	3.68E-02
2454	ENSG00000213918.10	DNASE1	-0.40	3.27E-03	3.68E-02
2455	ENSG00000136628.17	EPRS	0.27	3.28E-03	3.68E-02
2456	ENSG00000284337.1	LIMS3-LOC440895	1.81	3.28E-03	3.69E-02
2457	ENSG00000145632.14	PLK2	-0.50	3.29E-03	3.69E-02
2458	ENSG00000156928.4	MALSU1	0.41	3.30E-03	3.70E-02
2459	ENSG00000283196.2	AC006453.2	-0.71	3.31E-03	3.72E-02
2460	ENSG00000171811.13	CFAP46	2.24	3.31E-03	3.72E-02
2461	ENSG00000236283.4	AC019197.1	-0.78	3.32E-03	3.72E-02
2462	ENSG00000125968.8	ID1	0.53	3.32E-03	3.72E-02
2463	ENSG00000261446.3	LINC00559	-2.19	3.32E-03	3.72E-02
2464	ENSG00000087076.8	HSD17B14	-1.18	3.32E-03	3.72E-02
2465	ENSG00000166033.12	HTRA1	-0.43	3.33E-03	3.73E-02
2466	ENSG00000167693.16	NXN	0.63	3.34E-03	3.74E-02
2467	ENSG00000149212.11	SESN3	0.49	3.34E-03	3.74E-02
2468	ENSG00000175606.10	TMEM70	0.68	3.35E-03	3.74E-02
2469	ENSG00000138119.16	MYOF	-0.49	3.35E-03	3.74E-02
2470	ENSG00000162409.10	PRKAA2	0.57	3.36E-03	3.75E-02
2471	ENSG00000230063.1	AL360091.2	0.75	3.37E-03	3.77E-02
2472	ENSG00000106688.11	SLC1A1	0.68	3.39E-03	3.78E-02
2473	ENSG00000128487.16	SPECC1	-0.45	3.39E-03	3.78E-02
2474	ENSG00000257940.1	AC079598.1	3.82	3.40E-03	3.79E-02
2475	ENSG00000277277.3	FAM243B	-1.01	3.40E-03	3.79E-02
2476	ENSG00000241679.2	AC018450.1	-1.91	3.41E-03	3.80E-02
2477	ENSG00000100014.19	SPECC1L	-0.29	3.42E-03	3.81E-02
2478	ENSG00000183048.11	SLC25A10	0.60	3.42E-03	3.81E-02
2479	ENSG00000104343.19	UBE2W	0.43	3.42E-03	3.81E-02
2480	ENSG00000182107.6	TMEM30B	-0.93	3.43E-03	3.82E-02
2481	ENSG00000186417.13	GLDN	-0.55	3.45E-03	3.84E-02
2482	ENSG00000226091.7	LINC00937	-0.71	3.45E-03	3.84E-02
2483	ENSG00000273090.1	AC007378.1	-1.98	3.47E-03	3.85E-02
2484	ENSG00000122435.9	TRMT13	0.38	3.47E-03	3.85E-02
2485	ENSG00000106305.9	AIMP2	0.88	3.48E-03	3.86E-02
2486	ENSG00000172292.14	CERS6	-0.43	3.48E-03	3.86E-02
2487	ENSG00000092295.11	TGM1	-1.58	3.48E-03	3.86E-02
2488	ENSG00000116675.15	DNAJC6	0.88	3.48E-03	3.86E-02
2489	ENSG00000228513.1	AC023271.1	1.36	3.48E-03	3.86E-02
2490	ENSG00000090382.6	LYZ	0.86	3.49E-03	3.86E-02
2491	ENSG00000133657.15	ATP13A3	0.40	3.49E-03	3.86E-02
2492	ENSG00000261888.1	AC144831.1	-1.04	3.49E-03	3.86E-02
2493	ENSG00000182580.2	EPHB3	-1.39	3.52E-03	3.89E-02
2494	ENSG00000134470.20	IL15RA	-0.87	3.52E-03	3.89E-02
2495	ENSG00000085185.15	BCORL1	-0.47	3.53E-03	3.91E-02
2496	ENSG00000136897.7	MRPL50	0.45	3.54E-03	3.91E-02
2497	ENSG00000106588.10	PSMA2	0.43	3.54E-03	3.91E-02

2498	ENSG00000250624.1	MTCYBP43	-2.92	3.54E-03	3.91E-02
2499	ENSG00000185046.18	ANKS1B	-0.48	3.55E-03	3.92E-02
2500	ENSG00000172005.10	MAL	0.99	3.56E-03	3.93E-02
2501	ENSG00000167193.7	CRK	0.33	3.58E-03	3.95E-02
2502	ENSG00000142082.14	SIRT3	0.32	3.59E-03	3.96E-02
2503	ENSG00000198825.13	INPP5F	-0.37	3.59E-03	3.96E-02
2504	ENSG00000181626.11	ANKRD62	1.44	3.60E-03	3.96E-02
2505	ENSG00000172830.12	SSH3	-0.64	3.60E-03	3.97E-02
2506	ENSG00000259866.1	AC007599.1	-0.96	3.62E-03	3.98E-02
2507	ENSG00000131100.12	ATP6V1E1	0.41	3.62E-03	3.99E-02
2508	ENSG00000264343.5	NOTCH2NLA	-0.45	3.62E-03	3.99E-02
2509	ENSG00000258920.1	FOXN3-AS1	0.90	3.64E-03	4.00E-02
2510	ENSG00000155115.6	GTF3C6	0.42	3.64E-03	4.00E-02
2511	ENSG00000279669.1	AC079801.1	1.53	3.64E-03	4.00E-02
2512	ENSG00000154134.14	ROBO3	-0.50	3.64E-03	4.00E-02
2513	ENSG00000143226.13	FCGR2A	0.62	3.65E-03	4.01E-02
2514	ENSG00000149218.4	ENDOD1	0.42	3.66E-03	4.01E-02
2515	ENSG00000223953.5	C1QTNF5	-0.56	3.67E-03	4.02E-02
2516	ENSG00000154102.10	C16orf74	-1.61	3.67E-03	4.02E-02
2517	ENSG00000167642.12	SPINT2	-1.22	3.67E-03	4.02E-02
2518	ENSG00000101280.7	ANGPT4	-1.08	3.67E-03	4.03E-02
2519	ENSG00000087266.15	SH3BP2	-0.45	3.70E-03	4.06E-02
2520	ENSG00000196811.11	CHRNG	-1.42	3.71E-03	4.06E-02
2521	ENSG00000137265.14	IRF4	2.45	3.71E-03	4.06E-02
2522	ENSG00000162755.13	KLHDC9	1.14	3.72E-03	4.07E-02
2523	ENSG00000159713.10	TPPP3	-0.65	3.72E-03	4.07E-02
2524	ENSG00000115307.16	AUP1	0.48	3.72E-03	4.07E-02
2525	ENSG00000104205.13	SGK3	0.47	3.72E-03	4.07E-02
2526	ENSG00000143376.13	SNX27	0.26	3.73E-03	4.07E-02
2527	ENSG00000113407.13	TARS	0.39	3.75E-03	4.09E-02
2528	ENSG00000148187.17	MRRF	0.36	3.75E-03	4.09E-02
2529	ENSG00000103051.18	COG4	0.30	3.75E-03	4.09E-02
2530	ENSG00000185453.12	ZSWIM9	-0.56	3.75E-03	4.09E-02
2531	ENSG00000128283.6	CDC42EP1	-0.70	3.75E-03	4.09E-02
2532	ENSG00000214049.7	UCA1	-0.41	3.76E-03	4.10E-02
2533	ENSG00000185306.12	C12orf56	1.25	3.76E-03	4.10E-02
2534	ENSG00000232623.1	AP000266.1	1.21	3.78E-03	4.11E-02
2535	ENSG00000237689.1	AC007064.2	0.92	3.78E-03	4.12E-02
2536	ENSG00000124159.15	MATN4	-3.24	3.78E-03	4.12E-02
2537	ENSG00000241912.1	AC078788.2	3.70	3.80E-03	4.13E-02
2538	ENSG00000093100.13	AC016026.1	-0.54	3.80E-03	4.13E-02
2539	ENSG00000172461.10	FUT9	-0.97	3.80E-03	4.13E-02
2540	ENSG00000105793.15	GTPBP10	0.37	3.80E-03	4.13E-02
2541	ENSG00000196235.13	SUPT5H	-0.28	3.81E-03	4.14E-02
2542	ENSG00000137166.14	FOXP4	0.63	3.82E-03	4.14E-02
2543	ENSG00000219926.11	OR7E104P	-1.36	3.82E-03	4.15E-02
2544	ENSG00000126070.19	AGO3	-0.33	3.85E-03	4.17E-02
2545	ENSG00000231035.1	RPL7L1P9	-0.92	3.85E-03	4.17E-02
2546	ENSG00000152137.6	HSPB8	0.98	3.86E-03	4.18E-02
2547	ENSG00000104973.17	MED25	-0.42	3.86E-03	4.18E-02

2548	ENSG00000262119.1	AL079343.1	-1.08	3.88E-03	4.20E-02
2549	ENSG00000106701.11	FSD1L	-0.55	3.89E-03	4.21E-02
2550	ENSG00000163812.13	ZDHHC3	0.33	3.90E-03	4.22E-02
2551	ENSG00000140807.6	NKD1	-0.56	3.90E-03	4.22E-02
2552	ENSG00000102189.16	EEA1	-0.38	3.91E-03	4.22E-02
2553	ENSG00000113048.16	MRPS27	0.41	3.91E-03	4.22E-02
2554	ENSG00000094963.13	FMO2	-0.62	3.91E-03	4.22E-02
2555	ENSG00000225265.1	TAF1A-AS1	1.13	3.92E-03	4.23E-02
2556	ENSG00000166793.10	YPEL4	-0.80	3.92E-03	4.23E-02
2557	ENSG00000149480.6	MTA2	-0.30	3.94E-03	4.25E-02
2558	ENSG00000143353.11	LYPLAL1	0.51	3.94E-03	4.25E-02
2559	ENSG00000100612.13	DHRS7	0.36	3.95E-03	4.26E-02
2560	ENSG00000144021.2	CIAO1	0.33	3.95E-03	4.26E-02
2561	ENSG00000077549.17	CAPZB	-0.33	3.95E-03	4.26E-02
2562	ENSG00000138801.8	PAPSS1	-0.53	3.95E-03	4.26E-02
2563	ENSG00000198771.10	RCSD1	0.42	3.96E-03	4.26E-02
2564	ENSG00000227700.1	AC239809.2	3.24	3.99E-03	4.29E-02
2565	ENSG00000136383.6	ALPK3	0.93	3.99E-03	4.29E-02
2566	ENSG00000198734.10	F5	-1.45	4.01E-03	4.31E-02
2567	ENSG00000230606.10	AC092683.1	-0.32	4.02E-03	4.32E-02
2568	ENSG00000141736.13	ERBB2	-0.57	4.03E-03	4.33E-02
2569	ENSG00000106211.8	HSPB1	0.66	4.04E-03	4.34E-02
2570	ENSG00000186174.12	BCL9L	-0.43	4.04E-03	4.34E-02
2571	ENSG00000137106.17	GRHPR	0.42	4.05E-03	4.34E-02
2572	ENSG00000108298.11	RPL19	0.36	4.05E-03	4.34E-02
2573	ENSG00000179152.19	TCAIM	0.33	4.05E-03	4.34E-02
2574	ENSG00000000460.16	C1orf112	-0.50	4.05E-03	4.34E-02
2575	ENSG00000138764.14	CCNG2	-0.62	4.05E-03	4.34E-02
2576	ENSG00000119242.8	CCDC92	-0.59	4.06E-03	4.34E-02
2577	ENSG00000182095.14	TNRC18	-0.34	4.08E-03	4.37E-02
2578	ENSG00000014216.15	CAPN1	-0.36	4.10E-03	4.39E-02
2579	ENSG00000265784.1	AC006441.3	-2.90	4.13E-03	4.41E-02
2580	ENSG00000257327.1	AC012555.1	1.74	4.13E-03	4.42E-02
2581	ENSG00000168802.12	CHTF8	-0.34	4.15E-03	4.43E-02
2582	ENSG00000160799.11	CCDC12	-0.39	4.16E-03	4.44E-02
2583	ENSG00000048544.5	MRPS10	0.34	4.18E-03	4.46E-02
2584	ENSG00000025423.11	HSD17B6	2.97	4.18E-03	4.47E-02
2585	ENSG00000270170.1	NCBP2-AS2	0.66	4.19E-03	4.48E-02
2586	ENSG00000276980.1	AC008760.2	-0.98	4.20E-03	4.49E-02
2587	ENSG00000277476.1	AC005332.5	-0.63	4.21E-03	4.49E-02
2588	ENSG00000100890.15	KIAA0391	0.34	4.22E-03	4.50E-02
2589	ENSG00000279281.1	AC015883.1	0.86	4.24E-03	4.52E-02
2590	ENSG00000155959.10	VBP1	0.38	4.24E-03	4.52E-02
2591	ENSG00000103199.13	ZNF500	-0.49	4.24E-03	4.52E-02
2592	ENSG00000241886.1	AC112496.1	3.03	4.25E-03	4.52E-02
2593	ENSG00000229534.1	HNRNPA1P53	-1.84	4.25E-03	4.53E-02
2594	ENSG00000154856.12	APCDD1	-0.77	4.26E-03	4.53E-02
2595	ENSG00000184465.16	WDR27	-0.34	4.26E-03	4.53E-02
2596	ENSG00000186654.20	PRR5	-0.75	4.26E-03	4.53E-02
2597	ENSG00000090316.15	MAEA	0.31	4.28E-03	4.54E-02

2598	ENSG00000177383.4	MAGEF1	-0.57	4.28E-03	4.55E-02
2599	ENSG00000223495.2	AC239859.2	-2.33	4.29E-03	4.56E-02
2600	ENSG00000116574.5	RHOU	-0.68	4.31E-03	4.57E-02
2601	ENSG00000168234.12	TTC39C	0.49	4.31E-03	4.57E-02
2602	ENSG00000005882.11	PDK2	0.50	4.34E-03	4.60E-02
2603	ENSG00000177469.12	CAVIN1	-0.46	4.34E-03	4.60E-02
2604	ENSG00000062096.14	ARSF	3.00	4.35E-03	4.61E-02
2605	ENSG00000269446.2	AC006967.3	2.27	4.35E-03	4.61E-02
2606	ENSG00000171208.9	NETO2	1.31	4.36E-03	4.61E-02
2607	ENSG00000129048.6	ACKR4	-0.69	4.37E-03	4.62E-02
2608	ENSG00000169067.3	ACTBL2	-1.46	4.37E-03	4.62E-02
2609	ENSG00000171241.8	SHCBP1	-0.88	4.37E-03	4.63E-02
2610	ENSG00000125398.6	SOX9	-1.88	4.38E-03	4.63E-02
2611	ENSG00000256812.1	CAPNS2	-2.34	4.39E-03	4.64E-02
2612	ENSG00000258529.5	AP001781.2	3.04	4.40E-03	4.64E-02
2613	ENSG00000204271.12	SPIN3	-0.28	4.40E-03	4.64E-02
2614	ENSG00000086504.16	MRPL28	0.47	4.40E-03	4.65E-02
2615	ENSG00000232081.1	LARGE-IT1	-1.68	4.40E-03	4.65E-02
2616	ENSG00000100983.10	GSS	0.33	4.41E-03	4.65E-02
2617	ENSG00000251136.8	AF117829.1	-0.42	4.42E-03	4.66E-02
2618	ENSG00000143409.15	MINDY1	-0.49	4.42E-03	4.66E-02
2619	ENSG00000181619.11	GPR135	-0.63	4.42E-03	4.66E-02
2620	ENSG00000198010.12	DLGAP2	1.58	4.44E-03	4.67E-02
2621	ENSG00000253632.1	AC084026.2	-3.13	4.44E-03	4.67E-02
2622	ENSG00000244411.3	KRTAP5-7	-1.88	4.44E-03	4.68E-02
2623	ENSG00000234553.1	AC022431.1	2.73	4.46E-03	4.69E-02
2624	ENSG00000146250.6	PRSS35	1.86	4.47E-03	4.70E-02
2625	ENSG00000170037.13	CNTROB	-0.39	4.47E-03	4.70E-02
2626	ENSG00000160886.13	LY6K	0.89	4.47E-03	4.70E-02
2627	ENSG00000013503.9	POLR3B	0.48	4.48E-03	4.70E-02
2628	ENSG00000188778.5	ADRB3	0.57	4.49E-03	4.71E-02
2629	ENSG00000123612.15	ACVR1C	-0.54	4.50E-03	4.72E-02
2630	ENSG00000138448.11	ITGAV	0.34	4.51E-03	4.73E-02
2631	ENSG00000277791.4	PSMB3	0.75	4.52E-03	4.74E-02
2632	ENSG00000186918.13	ZNF395	-0.32	4.52E-03	4.74E-02
2633	ENSG00000136059.14	VILL	-0.66	4.52E-03	4.74E-02
2634	ENSG00000187699.10	C2orf88	0.50	4.53E-03	4.74E-02
2635	ENSG00000117425.13	PTCH2	-0.73	4.53E-03	4.74E-02
2636	ENSG00000122026.10	RPL21	0.31	4.55E-03	4.76E-02
2637	ENSG00000185973.10	TMLHE	0.32	4.55E-03	4.77E-02
2638	ENSG00000147459.17	DOCK5	0.50	4.57E-03	4.78E-02
2639	ENSG00000078081.7	LAMP3	1.29	4.58E-03	4.79E-02
2640	ENSG00000021645.18	NRXN3	0.64	4.58E-03	4.79E-02
2641	ENSG00000163251.3	FZD5	-0.61	4.59E-03	4.80E-02
2642	ENSG00000232536.1	AL365436.2	-2.72	4.60E-03	4.80E-02
2643	ENSG00000102893.15	PHKB	0.30	4.61E-03	4.81E-02
2644	ENSG00000079691.17	CARMIL1	-0.71	4.61E-03	4.81E-02
2645	ENSG00000144659.12	SLC25A38	0.34	4.62E-03	4.82E-02
2646	ENSG00000070444.14	MNT	-0.38	4.62E-03	4.82E-02
2647	ENSG00000153071.14	DAB2	-0.36	4.63E-03	4.82E-02

2648	ENSG00000128645.14	HOXD1	-2.68	4.66E-03	4.85E-02
2649	ENSG00000187151.7	ANGPTL5	-1.09	4.66E-03	4.85E-02
2650	ENSG00000245812.2	LINC02202	-0.67	4.67E-03	4.86E-02
2651	ENSG00000188641.13	DPYD	0.36	4.67E-03	4.86E-02
2652	ENSG00000126016.15	AMOT	-0.73	4.67E-03	4.86E-02
2653	ENSG00000222460.1	RN7SKP271	2.44	4.69E-03	4.87E-02
2654	ENSG00000125618.16	PAX8	-1.15	4.70E-03	4.89E-02
2655	ENSG00000264324.1	AC006030.1	-1.60	4.70E-03	4.89E-02
2656	ENSG00000188706.12	ZDHHC9	-0.43	4.71E-03	4.89E-02
2657	ENSG00000137563.11	GGH	-0.64	4.71E-03	4.89E-02
2658	ENSG00000123562.16	MORF4L2	-0.23	4.72E-03	4.89E-02
2659	ENSG00000103363.14	ELOB	0.45	4.72E-03	4.90E-02
2660	ENSG00000103507.13	BCKDK	0.53	4.72E-03	4.90E-02
2661	ENSG00000165194.15	PCDH19	0.61	4.74E-03	4.91E-02
2662	ENSG00000237187.8	NR2F1-AS1	-0.58	4.74E-03	4.91E-02
2663	ENSG00000141298.18	SSH2	-0.34	4.76E-03	4.93E-02
2664	ENSG00000146112.11	PPP1R18	-0.48	4.77E-03	4.94E-02
2665	ENSG00000135144.7	DTX1	-0.53	4.77E-03	4.94E-02
2666	ENSG00000177082.12	WDR73	-0.44	4.78E-03	4.94E-02
2667	ENSG00000163468.14	CCT3	0.36	4.78E-03	4.95E-02
2668	ENSG00000186451.1	SPATA12	-1.49	4.79E-03	4.95E-02
2669	ENSG00000261216.1	AC007216.2	3.86	4.81E-03	4.96E-02
2670	ENSG00000240654.6	C1QTNF9	0.90	4.80E-03	4.96E-02
2671	ENSG00000162889.10	MAPKAPK2	0.35	4.81E-03	4.96E-02
2672	ENSG00000107554.16	DNMBP	-0.35	4.81E-03	4.96E-02
2673	ENSG00000146918.19	NCAPG2	-0.36	4.81E-03	4.96E-02
2674	ENSG00000149328.14	GLB1L2	-1.29	4.82E-03	4.97E-02
2675	ENSG00000184489.11	PTP4A3	0.67	4.83E-03	4.97E-02
2676	ENSG00000115556.13	PLCD4	0.65	4.83E-03	4.97E-02
2677	ENSG00000119771.14	KLHL29	0.31	4.82E-03	4.97E-02
2678	ENSG00000197982.13	C1orf122	0.45	4.83E-03	4.98E-02
2679	ENSG00000102098.17	SCML2	0.71	4.84E-03	4.99E-02
2680	ENSG00000164344.15	KLKB1	1.30	4.85E-03	5.00E-02

Supplementary Table 2. List of genes differentially expressed between brown and white hMADS cells identified using two-tailed DESeq2 analysis (n=3; FDR<0.05).

number	ID	Gene symbol	log2FoldChange	P value	FDR adjusted P
1	ENSG00000135114.12	OASL	6.72	0.00E+00	0.00E+00
2	ENSG00000109424.3	UCP1	5.78	0.00E+00	0.00E+00
3	ENSG00000198589.11	LRBA	2.85	6.40E-180	4.24E-176
4	ENSG00000165409.17	TSHR	5.03	2.43E-168	1.21E-164
5	ENSG00000144481.16	TRPM8	4.10	4.11E-166	1.63E-162
6	ENSG00000153283.12	CD96	4.39	1.69E-130	5.59E-127
7	ENSG00000162877.12	PM20D1	7.00	4.16E-128	1.18E-124
8	ENSG00000157150.4	TIMP4	-3.12	9.27E-124	2.30E-120
9	ENSG00000169239.12	CA5B	2.60	3.56E-120	7.87E-117
10	ENSG00000004799.7	PDK4	2.77	7.87E-117	1.56E-113
11	ENSG00000137642.12	SORL1	3.66	2.10E-108	3.79E-105
12	ENSG00000277363.4	SRCIN1	3.63	1.25E-107	2.07E-104
13	ENSG00000167315.17	ACAA2	2.17	1.38E-106	2.11E-103
14	ENSG00000181092.9	ADIPOQ	2.39	5.99E-105	8.51E-102
15	ENSG00000121057.12	AKAP1	2.40	5.59E-103	7.40E-100
16	ENSG00000147251.15	DOCK11	2.25	5.27E-102	6.54E-99
17	ENSG00000170262.12	MRAP	2.40	4.02E-99	4.70E-96
18	ENSG00000237289.9	CKMT1B	2.93	1.15E-98	1.27E-95
19	ENSG00000133401.15	PDZD2	2.49	3.08E-94	3.22E-91
20	ENSG00000143554.13	SLC27A3	2.61	4.68E-94	4.64E-91
21	ENSG00000126709.14	IFI6	2.68	7.96E-94	7.53E-91
22	ENSG00000124003.12	MOGAT1	3.43	2.53E-93	2.28E-90
23	ENSG00000131943.17	C19orf12	2.00	1.32E-88	1.14E-85
24	ENSG00000133027.17	PEMT	2.94	1.97E-87	1.63E-84
25	ENSG00000198814.12	GK	2.71	1.66E-85	1.32E-82
26	ENSG00000114757.18	PEX5L	-4.35	3.86E-82	2.95E-79
27	ENSG00000099260.10	PALMD	2.71	1.11E-80	8.15E-78
28	ENSG00000223572.9	CKMT1A	2.79	2.55E-80	1.81E-77
29	ENSG00000198346.10	ZNF813	2.48	1.92E-79	1.32E-76
30	ENSG00000172179.11	PRL	-5.48	7.35E-79	4.87E-76
31	ENSG00000154127.9	UBASH3B	2.70	2.26E-78	1.45E-75
32	ENSG00000104763.18	ASAH1	2.07	9.24E-77	5.74E-74
33	ENSG00000205560.12	CPT1B	3.12	1.04E-76	6.28E-74
34	ENSG00000138029.13	HADHB	1.51	6.75E-75	3.94E-72
35	ENSG00000198223.16	CSF2RA	3.46	2.05E-74	1.13E-71
36	ENSG00000226482.1	ADIPOQ-AS1	2.92	6.41E-74	3.44E-71
37	ENSG00000119673.14	ACOT2	2.00	3.13E-73	1.64E-70
38	ENSG00000082074.16	FYB1	3.17	2.09E-72	1.06E-69
39	ENSG00000135447.16	PPP1R1A	1.87	6.71E-72	3.33E-69
40	ENSG00000057294.14	PKP2	2.55	2.53E-71	1.23E-68
41	ENSG00000125538.11	IL1B	3.27	6.75E-70	3.20E-67
42	ENSG00000140374.15	ETFA	1.61	1.00E-69	4.62E-67
43	ENSG00000103723.13	AP3B2	2.04	1.43E-69	6.47E-67
44	ENSG00000118113.11	MMP8	-5.68	9.33E-68	4.12E-65
45	ENSG00000279964.1	AC009949.1	4.12	2.37E-67	1.00E-64
46	ENSG00000139631.18	CSAD	1.79	2.34E-67	1.00E-64
47	ENSG00000164318.17	EGFLAM	2.12	1.46E-66	6.04E-64

48	ENSG00000128268.11	MGAT3	2.13	2.58E-66	1.04E-63
49	ENSG00000164309.14	CMYA5	2.19	9.60E-66	3.81E-63
50	ENSG00000134242.15	PTPN22	-3.41	1.70E-65	6.64E-63
51	ENSG00000107104.18	KANK1	1.52	4.93E-65	1.89E-62
52	ENSG00000144749.13	LRIG1	-1.60	1.30E-64	4.89E-62
53	ENSG00000108960.8	MMD	2.22	8.54E-64	3.14E-61
54	ENSG00000166821.8	PEX11A	1.89	1.97E-63	7.11E-61
55	ENSG00000123360.11	PDE1B	2.20	6.22E-61	2.21E-58
56	ENSG00000184227.7	ACOT1	1.57	9.39E-61	3.27E-58
57	ENSG00000158445.9	KCNB1	1.87	1.73E-60	5.93E-58
58	ENSG00000103150.5	MLYCD	2.00	2.52E-60	8.49E-58
59	ENSG00000022267.16	FHL1	1.59	7.64E-60	2.53E-57
60	ENSG00000056972.18	TRAF3IP2	2.22	1.99E-59	6.47E-57
61	ENSG00000221963.5	APOL6	2.09	2.07E-59	6.62E-57
62	ENSG00000110042.7	DTX4	1.50	4.57E-59	1.44E-56
63	ENSG00000000005.5	TNMD	4.20	1.61E-58	4.97E-56
64	ENSG00000147383.10	NSDHL	1.35	1.62E-58	4.97E-56
65	ENSG00000171488.14	LRRC8C	-1.98	7.92E-58	2.38E-55
66	ENSG00000079739.16	PGM1	1.67	1.01E-57	2.99E-55
67	ENSG00000183655.12	KLHL25	2.08	1.09E-57	3.18E-55
68	ENSG00000185640.5	KRT79	5.01	2.78E-57	7.99E-55
69	ENSG00000101856.9	PGRMC1	1.81	7.47E-57	2.12E-54
70	ENSG00000151498.11	ACAD8	1.59	1.29E-56	3.62E-54
71	ENSG00000105135.15	ILVBL	1.95	2.29E-56	6.31E-54
72	ENSG00000270765.5	GAS2L2	3.53	3.81E-56	1.04E-53
73	ENSG00000178718.6	RPP25	2.20	6.17E-56	1.66E-53
74	ENSG00000130940.14	CASZ1	3.99	2.48E-55	6.57E-53
75	ENSG00000084754.11	HADHA	1.29	2.53E-54	6.63E-52
76	ENSG00000134369.15	NAV1	2.16	2.79E-54	7.19E-52
77	ENSG00000115425.13	PECR	1.42	8.07E-54	2.06E-51
78	ENSG00000165731.18	RET	-2.18	1.72E-53	4.33E-51
79	ENSG00000175445.15	LPL	2.12	5.22E-53	1.30E-50
80	ENSG00000166123.13	GPT2	1.42	3.29E-52	8.08E-50
81	ENSG00000178537.9	SLC25A20	1.58	3.40E-52	8.25E-50
82	ENSG00000064763.10	FAR2	2.10	5.97E-52	1.43E-49
83	ENSG00000179023.8	KLHDC7A	2.17	2.11E-51	4.99E-49
84	ENSG00000117500.12	TMED5	1.57	6.07E-51	1.42E-48
85	ENSG00000005187.11	ACSM3	3.30	8.20E-51	1.90E-48
86	ENSG00000104325.6	DECR1	1.30	1.87E-50	4.27E-48
87	ENSG00000176597.11	B3GNT5	2.58	2.46E-50	5.54E-48
88	ENSG00000254859.1	AC067930.4	4.54	5.41E-50	1.21E-47
89	ENSG00000117054.13	ACADM	1.69	6.87E-50	1.52E-47
90	ENSG00000138796.16	HADH	1.38	1.11E-49	2.43E-47
91	ENSG00000173801.16	JUP	-2.57	3.28E-49	7.07E-47
92	ENSG00000162433.14	AK4	1.21	1.12E-48	2.39E-46
93	ENSG00000148730.6	EIF4EBP2	1.45	5.09E-48	1.08E-45
94	ENSG00000278535.4	DHRS11	1.85	6.23E-48	1.30E-45
95	ENSG00000129675.15	ARHGEF6	2.12	1.13E-47	2.34E-45
96	ENSG00000123243.14	ITIH5	2.35	3.67E-47	7.53E-45
97	ENSG00000042445.13	RETSAT	1.22	5.10E-47	1.03E-44

98	ENSG00000101049.15	SGK2	1.62	9.92E-47	1.99E-44
99	ENSG00000099797.14	TECR	1.93	1.03E-46	2.06E-44
100	ENSG00000166104.15	AC126323.1	5.52	1.06E-46	2.08E-44
101	ENSG00000115226.9	FNDC4	1.63	1.15E-46	2.25E-44
102	ENSG00000135917.14	SLC19A3	2.02	1.59E-46	3.07E-44
103	ENSG00000124743.5	KLHL31	2.61	6.22E-46	1.19E-43
104	ENSG00000151376.16	ME3	1.61	8.16E-46	1.54E-43
105	ENSG00000213853.9	EMP2	1.72	1.13E-45	2.12E-43
106	ENSG00000197375.12	SLC22A5	2.55	1.30E-45	2.41E-43
107	ENSG00000187240.14	DYNC2H1	-1.69	1.56E-45	2.87E-43
108	ENSG00000164604.12	GPR85	-2.50	1.91E-45	3.48E-43
109	ENSG00000121879.4	PIK3CA	1.29	4.39E-45	7.93E-43
110	ENSG00000073737.16	DHRS9	3.75	4.47E-45	8.01E-43
111	ENSG00000186532.11	SMYD4	1.60	6.87E-45	1.22E-42
112	ENSG00000237515.8	SHISA9	2.65	1.64E-44	2.89E-42
113	ENSG00000147202.17	DIAPH2	-1.78	1.81E-44	3.16E-42
114	ENSG00000007314.12	SCN4A	5.05	2.26E-44	3.90E-42
115	ENSG00000259916.1	AL845331.2	3.14	3.66E-44	6.27E-42
116	ENSG00000138678.10	GPAT3	3.17	3.71E-44	6.30E-42
117	ENSG00000119686.9	FLVCR2	2.30	4.08E-44	6.87E-42
118	ENSG00000146477.5	SLC22A3	2.88	5.97E-44	9.97E-42
119	ENSG00000144908.13	ALDH1L1	1.27	3.89E-43	6.43E-41
120	ENSG00000086848.14	ALG9	2.14	5.77E-43	9.47E-41
121	ENSG00000164117.13	FBXO8	1.59	7.65E-43	1.25E-40
122	ENSG00000198959.11	TGM2	2.01	7.75E-43	1.25E-40
123	ENSG00000147465.11	STAR	3.65	8.75E-43	1.40E-40
124	ENSG00000137274.12	BPHL	1.47	9.83E-43	1.56E-40
125	ENSG00000143171.12	RXRG	4.17	1.12E-42	1.77E-40
126	ENSG00000147894.15	C9orf72	1.84	2.24E-42	3.51E-40
127	ENSG00000120049.19	KCNIP2	1.55	2.37E-42	3.67E-40
128	ENSG00000165269.12	AQP7	1.45	2.67E-42	4.12E-40
129	ENSG00000137124.7	ALDH1B1	1.30	1.15E-41	1.76E-39
130	ENSG00000115255.10	REEP6	2.06	1.54E-41	2.34E-39
131	ENSG00000089127.12	OAS1	3.03	1.64E-41	2.47E-39
132	ENSG00000147592.8	LACTB2	1.67	1.73E-41	2.58E-39
133	ENSG00000185052.11	SLC24A3	-3.45	4.47E-41	6.63E-39
134	ENSG00000171659.14	GPR34	2.95	6.07E-41	8.94E-39
135	ENSG00000182704.7	TSKU	1.37	7.12E-41	1.04E-38
136	ENSG00000108551.4	RASD1	2.06	4.21E-40	6.10E-38
137	ENSG00000168000.14	BSCL2	2.10	4.25E-40	6.12E-38
138	ENSG00000278195.1	SSTR3	2.79	6.54E-40	9.35E-38
139	ENSG00000188613.6	NANOS1	1.89	1.18E-39	1.68E-37
140	ENSG00000168496.3	FEN1	-1.51	1.53E-39	2.16E-37
141	ENSG00000157152.16	SYN2	2.18	1.68E-39	2.35E-37
142	ENSG00000060971.17	ACAA1	1.40	4.24E-39	5.89E-37
143	ENSG00000272414.5	FAM47E-STBD1	1.52	4.96E-39	6.84E-37
144	ENSG00000186417.13	GLDN	1.90	5.14E-39	7.03E-37
145	ENSG00000119471.14	HSDL2	1.32	5.17E-39	7.03E-37
146	ENSG00000170458.13	CD14	2.77	8.16E-39	1.10E-36
147	ENSG00000162735.18	PEX19	1.35	1.09E-38	1.46E-36

148	ENSG00000122859.4	NEUROG3	2.68	1.75E-38	2.33E-36
149	ENSG00000104490.17	NCALD	1.44	2.00E-38	2.63E-36
150	ENSG00000112246.9	SIM1	-1.71	2.00E-38	2.63E-36
151	ENSG00000183044.11	ABAT	-2.09	2.49E-38	3.25E-36
152	ENSG00000153064.11	BANK1	1.84	3.48E-38	4.52E-36
153	ENSG00000233574.1	AL020994.3	4.29	3.57E-38	4.60E-36
154	ENSG00000157617.16	C2CD2	1.28	3.70E-38	4.74E-36
155	ENSG00000261371.5	PECAM1	2.16	4.24E-38	5.40E-36
156	ENSG00000173085.14	COQ2	1.53	4.41E-38	5.58E-36
157	ENSG00000087299.11	L2HGDH	1.82	5.44E-38	6.85E-36
158	ENSG00000196781.14	TLE1	1.37	7.38E-38	9.23E-36
159	ENSG00000246705.4	H2AFJ	1.76	8.81E-38	1.09E-35
160	ENSG00000272941.1	AC083862.2	3.19	1.84E-37	2.27E-35
161	ENSG00000172831.11	CES2	1.62	2.33E-37	2.85E-35
162	ENSG00000072778.19	ACADVL	1.15	3.08E-37	3.75E-35
163	ENSG00000174307.6	PHLDA3	1.67	4.97E-37	6.02E-35
164	ENSG00000198848.12	CES1	-1.85	5.19E-37	6.25E-35
165	ENSG00000104331.8	IMPAD1	1.21	6.43E-37	7.70E-35
166	ENSG00000091409.14	ITGA6	1.61	9.72E-37	1.16E-34
167	ENSG00000189157.13	FAM47E	2.00	1.13E-36	1.34E-34
168	ENSG00000177119.15	ANO6	1.21	2.09E-36	2.46E-34
169	ENSG00000135604.9	STX11	2.44	2.17E-36	2.53E-34
170	ENSG00000069122.18	ADGRF5	5.08	4.19E-36	4.87E-34
171	ENSG00000139155.8	SLCO1C1	-2.95	1.03E-35	1.19E-33
172	ENSG00000167676.4	PLIN4	1.85	1.55E-35	1.78E-33
173	ENSG00000167588.12	GPD1	1.14	1.77E-35	2.02E-33
174	ENSG00000181418.7	DDN	5.66	3.39E-35	3.85E-33
175	ENSG00000171503.11	ETFDH	1.38	9.19E-35	1.04E-32
176	ENSG00000157184.6	CPT2	1.40	1.03E-34	1.15E-32
177	ENSG00000280587.1	LINC01348	2.60	1.71E-34	1.90E-32
178	ENSG00000250802.7	ZBED3-AS1	2.68	1.79E-34	1.98E-32
179	ENSG00000198794.11	SCAMP5	1.31	2.30E-34	2.53E-32
180	ENSG00000000938.12	FGR	1.52	2.87E-34	3.15E-32
181	ENSG00000112294.12	ALDH5A1	1.77	5.09E-34	5.55E-32
182	ENSG00000170525.20	PFKFB3	1.85	5.52E-34	6.00E-32
183	ENSG00000167191.11	GPRC5B	2.25	5.76E-34	6.22E-32
184	ENSG00000131979.18	GCH1	2.24	7.80E-34	8.38E-32
185	ENSG00000110080.18	ST3GAL4	1.47	7.88E-34	8.42E-32
186	ENSG00000166689.15	PLEKHA7	2.79	8.15E-34	8.66E-32
187	ENSG00000168924.14	LETM1	1.37	9.66E-34	1.02E-31
188	ENSG00000008323.15	PLEKHG6	1.92	1.13E-33	1.19E-31
189	ENSG00000139354.10	GAS2L3	1.82	1.28E-33	1.34E-31
190	ENSG00000086300.15	SNX10	1.89	1.80E-33	1.87E-31
191	ENSG00000234688.1	AL049749.1	5.10	1.95E-33	2.02E-31
192	ENSG00000095203.14	EPB41L4B	2.41	3.11E-33	3.20E-31
193	ENSG00000197891.11	SLC22A12	3.76	3.94E-33	4.03E-31
194	ENSG00000130707.17	ASS1	1.07	4.44E-33	4.53E-31
195	ENSG00000144909.7	OSBPL11	1.47	7.02E-33	7.11E-31
196	ENSG00000163932.13	PRKCD	1.59	1.65E-32	1.66E-30
197	ENSG00000076555.15	ACACB	1.10	1.70E-32	1.70E-30

198	ENSG00000224614.1	TNK2-AS1	4.16	2.39E-32	2.38E-30
199	ENSG00000175426.10	PCSK1	2.17	4.36E-32	4.33E-30
200	ENSG00000197296.5	FITM2	1.16	4.75E-32	4.69E-30
201	ENSG00000133048.12	CHI3L1	-3.71	7.14E-32	7.03E-30
202	ENSG00000259494.1	MRPL46	1.51	1.06E-31	1.04E-29
203	ENSG00000143198.12	MGST3	1.58	1.52E-31	1.48E-29
204	ENSG00000173638.18	SLC19A1	1.28	2.03E-31	1.97E-29
205	ENSG00000204839.8	MROH6	3.29	2.38E-31	2.29E-29
206	ENSG00000249751.3	ECSCR	3.71	2.53E-31	2.42E-29
207	ENSG00000205414.1	AC007608.1	5.13	2.66E-31	2.54E-29
208	ENSG00000186469.8	GNG2	-1.42	3.29E-31	3.13E-29
209	ENSG00000196396.9	PTPN1	1.07	7.29E-31	6.90E-29
210	ENSG00000109846.7	CRYAB	-2.00	9.76E-31	9.20E-29
211	ENSG00000166398.12	KIAA0355	1.31	1.14E-30	1.07E-28
212	ENSG00000176194.17	CIDEA	6.68	1.48E-30	1.38E-28
213	ENSG00000161249.20	DMKN	1.59	1.68E-30	1.56E-28
214	ENSG00000171791.12	BCL2	1.36	2.04E-30	1.89E-28
215	ENSG00000008086.11	CDKL5	1.71	3.10E-30	2.85E-28
216	ENSG00000147813.15	NAPRT	2.58	3.26E-30	2.99E-28
217	ENSG00000068366.19	ACSL4	1.32	3.74E-30	3.41E-28
218	ENSG00000023171.17	GRAMD1B	1.74	4.26E-30	3.87E-28
219	ENSG00000108179.13	PPIF	1.15	6.60E-30	5.96E-28
220	ENSG00000196850.5	PPTC7	1.23	7.18E-30	6.45E-28
221	ENSG00000130475.14	FCHO1	2.73	9.35E-30	8.37E-28
222	ENSG00000072657.8	TRHDE	1.05	1.23E-29	1.10E-27
223	ENSG00000117593.10	DARS2	1.19	1.27E-29	1.12E-27
224	ENSG00000086712.12	TXLNG	1.60	1.32E-29	1.17E-27
225	ENSG00000162645.12	GBP2	1.10	1.53E-29	1.34E-27
226	ENSG00000282692.1	AC129915.3	2.55	1.57E-29	1.37E-27
227	ENSG00000123739.10	PLA2G12A	1.41	1.63E-29	1.42E-27
228	ENSG00000146859.6	TMEM140	1.61	1.76E-29	1.53E-27
229	ENSG00000260102.1	LINC01070	2.81	2.14E-29	1.85E-27
230	ENSG00000188001.9	TPRG1	-2.56	2.38E-29	2.05E-27
231	ENSG00000088766.11	CRLS1	1.50	3.91E-29	3.35E-27
232	ENSG00000154016.13	GRAP	2.93	3.95E-29	3.37E-27
233	ENSG00000134508.12	CABLES1	1.53	4.77E-29	4.05E-27
234	ENSG00000172840.6	PDP2	1.08	4.96E-29	4.19E-27
235	ENSG00000197943.9	PLCG2	3.69	5.03E-29	4.23E-27
236	ENSG00000226742.3	HSBP1L1	1.73	7.29E-29	6.11E-27
237	ENSG00000250479.8	CHCHD10	1.31	1.09E-28	9.08E-27
238	ENSG00000031081.10	ARHGAP31	1.32	1.32E-28	1.10E-26
239	ENSG00000100418.7	DESI1	1.37	1.64E-28	1.36E-26
240	ENSG00000065325.12	GLP2R	3.01	1.85E-28	1.52E-26
241	ENSG00000198142.4	SOWAHC	1.25	2.53E-28	2.08E-26
242	ENSG00000162191.13	UBXN1	1.23	4.00E-28	3.27E-26
243	ENSG00000140968.10	IRF8	4.26	4.15E-28	3.38E-26
244	ENSG00000186335.8	SLC36A2	2.79	4.38E-28	3.55E-26
245	ENSG00000139131.12	YARS2	1.17	7.43E-28	6.00E-26
246	ENSG00000167995.15	BEST1	1.54	9.32E-28	7.50E-26
247	ENSG00000127884.4	ECHS1	1.01	9.36E-28	7.50E-26

248	ENSG00000149547.14	EI24	1.13	9.52E-28	7.60E-26
249	ENSG00000250722.5	SELENOP	-1.47	1.13E-27	9.01E-26
250	ENSG00000164619.9	BMPER	-1.33	1.60E-27	1.26E-25
251	ENSG00000135953.10	MFSD9	1.37	2.23E-27	1.76E-25
252	ENSG00000260898.5	ADPGK-AS1	3.12	3.21E-27	2.52E-25
253	ENSG00000243244.6	STON1	3.61	4.41E-27	3.45E-25
254	ENSG00000099282.9	TSPAN15	1.76	5.47E-27	4.26E-25
255	ENSG00000025434.18	NR1H3	1.18	5.64E-27	4.38E-25
256	ENSG00000214513.3	NOTO	2.33	6.14E-27	4.75E-25
257	ENSG00000010278.13	CD9	-1.45	6.73E-27	5.18E-25
258	ENSG00000167772.11	ANGPTL4	3.55	7.39E-27	5.67E-25
259	ENSG00000161533.11	ACOX1	1.14	8.48E-27	6.48E-25
260	ENSG00000137878.17	GCOM1	1.47	9.09E-27	6.92E-25
261	ENSG00000143653.9	SCCPDH	1.23	9.98E-27	7.57E-25
262	ENSG00000110876.9	SELPLG	1.97	1.07E-26	8.09E-25
263	ENSG00000177363.4	LRRN4CL	1.75	1.22E-26	9.20E-25
264	ENSG00000165806.19	CASP7	1.43	1.23E-26	9.20E-25
265	ENSG00000133835.15	HSD17B4	0.97	1.23E-26	9.22E-25
266	ENSG00000170540.14	ARL6IP1	1.16	1.32E-26	9.84E-25
267	ENSG00000176134.5	AL445665.1	2.19	1.93E-26	1.43E-24
268	ENSG00000171612.6	SLC25A33	1.24	2.26E-26	1.67E-24
269	ENSG00000069535.13	MAOB	-1.57	3.36E-26	2.47E-24
270	ENSG00000125931.10	CITED1	2.65	4.06E-26	2.97E-24
271	ENSG00000253304.1	TMEM200B	-1.80	7.49E-26	5.47E-24
272	ENSG00000137133.10	HINT2	1.47	7.82E-26	5.69E-24
273	ENSG00000130208.9	APOC1	1.14	8.23E-26	5.97E-24
274	ENSG00000152332.15	UHMK1	1.01	8.41E-26	6.07E-24
275	ENSG00000168781.22	PPIP5K1	1.25	9.41E-26	6.78E-24
276	ENSG00000196821.9	C6orf106	0.98	9.54E-26	6.84E-24
277	ENSG00000104044.15	OCA2	3.13	1.12E-25	8.03E-24
278	ENSG00000100347.14	SAMM50	0.99	1.16E-25	8.25E-24
279	ENSG00000165272.15	AQP3	2.03	1.23E-25	8.71E-24
280	ENSG00000119986.6	AVPI1	1.64	1.32E-25	9.36E-24
281	ENSG00000170571.11	EMB	4.17	1.59E-25	1.12E-23
282	ENSG00000156804.7	FBXO32	-1.64	2.15E-25	1.51E-23
283	ENSG00000269293.2	ZSCAN16-AS1	1.69	2.61E-25	1.82E-23
284	ENSG00000128294.15	TPST2	1.22	3.08E-25	2.15E-23
285	ENSG00000124615.19	MOCS1	1.31	3.14E-25	2.18E-23
286	ENSG00000124570.19	SERPINB6	0.94	3.25E-25	2.25E-23
287	ENSG00000168944.15	CEP120	-1.26	3.53E-25	2.44E-23
288	ENSG00000085662.13	AKR1B1	1.52	4.40E-25	3.02E-23
289	ENSG00000155906.17	RMND1	1.06	4.90E-25	3.36E-23
290	ENSG00000173559.12	NABP1	-1.04	4.97E-25	3.40E-23
291	ENSG00000101695.8	RNF125	1.80	5.92E-25	4.03E-23
292	ENSG00000129009.12	ISLR	-1.54	9.19E-25	6.23E-23
293	ENSG00000283706.1	PRSS50	5.07	1.16E-24	7.83E-23
294	ENSG00000171714.11	ANO5	2.00	1.27E-24	8.57E-23
295	ENSG00000197147.13	LRRC8B	1.26	1.31E-24	8.82E-23
296	ENSG00000158859.9	ADAMTS4	1.48	1.35E-24	9.06E-23
297	ENSG00000134909.18	ARHGAP32	1.27	1.49E-24	9.94E-23

298	ENSG00000113578.17	FGF1	1.58	1.56E-24	1.04E-22
299	ENSG00000182118.7	FAM89A	1.31	2.23E-24	1.47E-22
300	ENSG00000196136.17	SERPINA3	2.22	2.66E-24	1.76E-22
301	ENSG00000228794.8	LINC01128	1.13	2.68E-24	1.77E-22
302	ENSG00000107957.16	SH3PXD2A	0.81	3.07E-24	2.01E-22
303	ENSG00000205517.12	RGL3	2.08	3.29E-24	2.15E-22
304	ENSG00000152137.6	HSPB8	1.28	3.56E-24	2.32E-22
305	ENSG00000166402.8	TUB	1.45	3.61E-24	2.35E-22
306	ENSG00000181991.15	MRPS11	1.05	4.08E-24	2.64E-22
307	ENSG00000143416.20	SELENBP1	-1.46	4.74E-24	3.06E-22
308	ENSG00000198736.11	MSRB1	1.19	5.82E-24	3.74E-22
309	ENSG00000160862.12	AZGP1	-1.34	5.95E-24	3.81E-22
310	ENSG00000100714.15	MTHFD1	1.07	6.65E-24	4.25E-22
311	ENSG00000205403.13	CFI	2.88	1.02E-23	6.48E-22
312	ENSG00000134107.4	BHLHE40	-1.38	1.25E-23	7.93E-22
313	ENSG00000104518.10	GSDMD	2.00	1.50E-23	9.49E-22
314	ENSG00000174640.13	SLCO2A1	3.84	1.62E-23	1.02E-21
315	ENSG00000165389.6	SPTSSA	-1.30	1.84E-23	1.16E-21
316	ENSG00000177076.5	ACER2	1.81	3.31E-23	2.07E-21
317	ENSG00000163536.12	SERPINI1	1.78	3.33E-23	2.08E-21
318	ENSG00000233070.1	ZFY-AS1	1.87	3.55E-23	2.21E-21
319	ENSG00000175054.14	ATR	1.03	3.71E-23	2.31E-21
320	ENSG00000153246.12	PLA2R1	1.30	3.96E-23	2.45E-21
321	ENSG00000158516.11	CPA2	5.22	4.45E-23	2.74E-21
322	ENSG00000162194.12	LBHD1	1.06	8.79E-23	5.41E-21
323	ENSG00000152078.9	TMEM56	1.22	9.97E-23	6.11E-21
324	ENSG00000116898.11	MRPS15	0.93	1.35E-22	8.23E-21
325	ENSG00000122483.17	CCDC18	1.50	1.71E-22	1.04E-20
326	ENSG00000126432.13	PRDX5	0.91	1.73E-22	1.05E-20
327	ENSG00000138207.13	RBP4	0.87	2.35E-22	1.43E-20
328	ENSG00000100335.14	MIEF1	0.98	2.75E-22	1.66E-20
329	ENSG00000152270.8	PDE3B	1.28	3.99E-22	2.41E-20
330	ENSG00000158292.6	GPR153	1.24	4.19E-22	2.51E-20
331	ENSG00000198468.7	FLVCR1-DT	1.86	4.98E-22	2.98E-20
332	ENSG00000152518.7	ZFP36L2	-1.10	5.03E-22	3.00E-20
333	ENSG00000034510.5	TMSB10	-1.05	5.66E-22	3.37E-20
334	ENSG00000164684.13	ZNF704	3.41	5.83E-22	3.46E-20
335	ENSG00000128422.16	KRT17	3.67	6.27E-22	3.71E-20
336	ENSG00000124588.19	NQO2	0.93	6.30E-22	3.71E-20
337	ENSG00000285330.1	AC126283.2	3.26	7.08E-22	4.16E-20
338	ENSG00000162769.12	FLVCR1	1.34	7.86E-22	4.60E-20
339	ENSG00000128606.12	LRRC17	-1.57	7.98E-22	4.66E-20
340	ENSG00000122378.13	PRXL2A	0.95	8.09E-22	4.71E-20
341	ENSG00000145423.4	SFRP2	-1.72	8.12E-22	4.71E-20
342	ENSG00000139211.6	AMIGO2	1.41	1.04E-21	6.05E-20
343	ENSG00000122729.18	ACO1	0.79	1.22E-21	7.08E-20
344	ENSG00000136156.13	ITM2B	0.78	1.41E-21	8.10E-20
345	ENSG00000151500.14	THYN1	1.25	1.58E-21	9.06E-20
346	ENSG00000198010.12	DLGAP2	2.03	1.61E-21	9.20E-20
347	ENSG00000169047.5	IRS1	-0.82	1.63E-21	9.31E-20

348	ENSG00000174136.11	RGMB	-1.09	1.90E-21	1.08E-19
349	ENSG00000189045.13	ANKDD1B	3.38	2.03E-21	1.15E-19
350	ENSG00000112531.16	QKI	0.85	2.20E-21	1.25E-19
351	ENSG00000158062.20	UBXN11	1.47	2.52E-21	1.42E-19
352	ENSG00000175691.8	ZNF77	1.69	2.72E-21	1.53E-19
353	ENSG00000118804.8	STBD1	1.57	3.01E-21	1.69E-19
354	ENSG00000152936.10	LMNTD1	4.29	3.86E-21	2.16E-19
355	ENSG00000178038.16	ALS2CL	-2.74	3.90E-21	2.18E-19
356	ENSG00000274021.1	AC024909.2	2.00	6.65E-21	3.70E-19
357	ENSG00000197077.13	KIAA1671	1.80	9.45E-21	5.24E-19
358	ENSG00000276023.4	DUSP14	-1.35	9.67E-21	5.35E-19
359	ENSG00000235109.7	ZSCAN31	-1.01	1.25E-20	6.90E-19
360	ENSG00000124766.6	SOX4	-0.97	1.46E-20	8.04E-19
361	ENSG00000034693.14	PEX3	1.17	1.52E-20	8.37E-19
362	ENSG00000169313.9	P2RY12	2.16	1.54E-20	8.45E-19
363	ENSG00000091513.15	TF	2.86	1.56E-20	8.51E-19
364	ENSG00000123095.5	BHLHE41	1.37	1.68E-20	9.14E-19
365	ENSG00000154262.12	ABCA6	-1.81	2.25E-20	1.22E-18
366	ENSG00000085265.10	FCN1	4.25	2.32E-20	1.25E-18
367	ENSG00000174749.5	FAM241A	1.03	2.37E-20	1.28E-18
368	ENSG00000106537.7	TSPAN13	0.98	2.73E-20	1.47E-18
369	ENSG00000186854.10	TRABD2A	1.31	3.41E-20	1.83E-18
370	ENSG00000106400.11	ZNHIT1	0.98	3.42E-20	1.83E-18
371	ENSG00000198380.12	GFPT1	1.30	5.44E-20	2.91E-18
372	ENSG00000100075.9	SLC25A1	1.18	7.22E-20	3.85E-18
373	ENSG00000113739.10	STC2	-1.05	7.44E-20	3.95E-18
374	ENSG00000163520.13	FBLN2	-1.35	7.90E-20	4.18E-18
375	ENSG00000131730.15	CKMT2	1.16	8.99E-20	4.75E-18
376	ENSG00000153551.13	CMTM7	1.54	9.08E-20	4.79E-18
377	ENSG00000137225.12	CAPN11	3.62	9.11E-20	4.79E-18
378	ENSG00000132846.5	ZBED3	1.13	9.59E-20	5.03E-18
379	ENSG00000172059.10	KLF11	1.14	1.10E-19	5.73E-18
380	ENSG00000148248.13	SURF4	0.83	1.47E-19	7.64E-18
381	ENSG00000116729.13	WLS	-0.97	1.49E-19	7.77E-18
382	ENSG00000069020.18	MAST4	1.41	1.55E-19	8.03E-18
383	ENSG00000281769.1	LINC01230	1.77	1.56E-19	8.08E-18
384	ENSG00000162378.12	ZYG11B	1.02	1.70E-19	8.78E-18
385	ENSG00000147257.13	GPC3	-1.91	1.82E-19	9.37E-18
386	ENSG00000164236.11	ANKRD33B	1.47	1.84E-19	9.47E-18
387	ENSG00000089091.16	DZANK1	2.28	1.99E-19	1.02E-17
388	ENSG00000151292.17	CSNK1G3	-0.96	2.07E-19	1.06E-17
389	ENSG00000160179.18	ABCG1	1.51	2.87E-19	1.46E-17
390	ENSG00000051620.10	HEBP2	0.94	3.04E-19	1.55E-17
391	ENSG00000121552.3	CSTA	-1.48	3.05E-19	1.55E-17
392	ENSG00000103064.14	SLC7A6	1.21	3.25E-19	1.64E-17
393	ENSG00000134030.13	CTIF	-0.97	3.82E-19	1.93E-17
394	ENSG00000184906.11	AMYH02020865.1	2.29	4.14E-19	2.08E-17
395	ENSG00000165478.6	HEPACAM	1.62	4.48E-19	2.25E-17
396	ENSG00000143162.8	CREG1	-0.96	5.20E-19	2.60E-17
397	ENSG00000014641.17	MDH1	0.83	5.27E-19	2.63E-17

398	ENSG00000278615.4	C11orf98	0.96	6.32E-19	3.15E-17
399	ENSG00000105976.14	MET	-1.19	7.37E-19	3.66E-17
400	ENSG00000204291.10	COL15A1	-1.21	7.58E-19	3.75E-17
401	ENSG00000267580.1	AC008738.3	0.98	8.39E-19	4.15E-17
402	ENSG00000146555.18	SDK1	2.25	9.75E-19	4.81E-17
403	ENSG00000157193.16	LRP8	-1.19	9.99E-19	4.92E-17
404	ENSG00000159164.9	SV2A	3.78	1.11E-18	5.45E-17
405	ENSG00000204257.14	HLA-DMA	1.49	1.13E-18	5.51E-17
406	ENSG00000223382.4	LINC01778	2.53	1.15E-18	5.60E-17
407	ENSG00000086289.11	EPDR1	0.95	1.21E-18	5.91E-17
408	ENSG00000131471.6	AOC3	0.93	1.28E-18	6.21E-17
409	ENSG00000214456.8	PLIN5	1.29	1.37E-18	6.63E-17
410	ENSG00000133065.10	SLC41A1	0.97	1.48E-18	7.18E-17
411	ENSG00000138119.16	MYOF	-0.95	1.54E-18	7.43E-17
412	ENSG00000165948.10	IFI27L1	1.03	1.59E-18	7.64E-17
413	ENSG00000255020.1	AF131216.3	3.33	1.59E-18	7.65E-17
414	ENSG00000122694.15	GLIPR2	1.23	1.62E-18	7.74E-17
415	ENSG00000070601.9	FRMPD1	2.08	1.68E-18	8.02E-17
416	ENSG00000143545.8	RAB13	1.01	1.86E-18	8.85E-17
417	ENSG00000105835.11	NAMPT	1.36	2.35E-18	1.12E-16
418	ENSG00000102024.17	PLS3	-0.95	2.44E-18	1.16E-16
419	ENSG00000110076.18	NRXN2	-2.76	2.71E-18	1.28E-16
420	ENSG00000170791.17	CHCHD7	1.07	2.72E-18	1.29E-16
421	ENSG00000236333.3	TRHDE-AS1	1.09	3.46E-18	1.63E-16
422	ENSG00000113140.10	SPARC	-0.79	3.82E-18	1.79E-16
423	ENSG00000189221.9	MAOA	-1.48	3.89E-18	1.82E-16
424	ENSG00000120885.21	CLU	-1.05	4.03E-18	1.88E-16
425	ENSG00000122008.15	POLK	1.32	4.26E-18	1.99E-16
426	ENSG00000211829.9	TRDC	1.49	4.30E-18	2.00E-16
427	ENSG00000169692.12	AGPAT2	1.25	4.48E-18	2.08E-16
428	ENSG00000136059.14	VILL	2.94	4.49E-18	2.08E-16
429	ENSG00000188690.13	UROS	0.79	4.73E-18	2.19E-16
430	ENSG00000134072.10	CAMK1	1.01	4.78E-18	2.20E-16
431	ENSG00000164048.13	ZNF589	1.29	5.08E-18	2.34E-16
432	ENSG00000105552.14	BCAT2	-1.19	5.12E-18	2.35E-16
433	ENSG00000132170.20	PPARG	-0.95	5.57E-18	2.55E-16
434	ENSG00000093144.18	ECHDC1	0.98	5.84E-18	2.67E-16
435	ENSG00000138356.13	AOX1	0.94	6.13E-18	2.79E-16
436	ENSG00000145358.6	DDIT4L	1.38	6.96E-18	3.17E-16
437	ENSG00000197381.15	ADARB1	-0.92	7.28E-18	3.30E-16
438	ENSG00000154217.14	PITPNC1	1.09	7.48E-18	3.39E-16
439	ENSG00000213930.11	GALT	1.07	8.29E-18	3.74E-16
440	ENSG00000137975.7	CLCA2	-1.27	8.78E-18	3.96E-16
441	ENSG00000124126.13	PREX1	1.00	9.13E-18	4.10E-16
442	ENSG00000132274.15	TRIM22	-0.96	1.26E-17	5.66E-16
443	ENSG00000165194.15	PCDH19	2.56	1.27E-17	5.69E-16
444	ENSG00000105379.9	ETFB	1.05	1.28E-17	5.69E-16
445	ENSG00000149485.18	FADS1	-1.11	1.40E-17	6.22E-16
446	ENSG00000245848.2	CEBPA	0.99	1.48E-17	6.58E-16
447	ENSG00000176171.11	BNIP3	0.86	1.50E-17	6.64E-16

448	ENSG00000171316.11	CHD7	1.97	1.50E-17	6.65E-16
449	ENSG00000163531.15	NFASC	-1.27	1.73E-17	7.64E-16
450	ENSG00000125945.14	ZNF436	1.17	1.74E-17	7.65E-16
451	ENSG00000042286.14	AIFM2	0.95	1.91E-17	8.37E-16
452	ENSG00000145687.16	SSBP2	-1.01	1.93E-17	8.47E-16
453	ENSG00000137745.11	MMP13	-2.26	1.95E-17	8.54E-16
454	ENSG00000163513.17	TGFBR2	-1.03	1.96E-17	8.54E-16
455	ENSG00000127948.15	POR	0.97	2.05E-17	8.91E-16
456	ENSG00000134824.13	FADS2	-1.02	2.49E-17	1.08E-15
457	ENSG00000148339.12	SLC25A25	1.07	2.60E-17	1.13E-15
458	ENSG00000065060.16	UHRF1BP1	0.81	2.83E-17	1.23E-15
459	ENSG00000279026.1	AC005225.4	1.60	3.01E-17	1.30E-15
460	ENSG00000168542.14	COL3A1	-0.84	3.06E-17	1.32E-15
461	ENSG00000106003.12	LFNG	-1.64	3.68E-17	1.58E-15
462	ENSG00000120899.17	PTK2B	0.89	3.92E-17	1.68E-15
463	ENSG00000112139.15	MDGA1	-1.50	4.05E-17	1.73E-15
464	ENSG00000134986.13	NREP	-0.77	4.24E-17	1.81E-15
465	ENSG00000070915.9	SLC12A3	4.33	4.77E-17	2.03E-15
466	ENSG00000169282.17	KCNAB1	1.22	5.41E-17	2.30E-15
467	ENSG00000164125.15	FAM198B	-1.13	7.66E-17	3.25E-15
468	ENSG00000214837.8	LINC01347	1.58	7.91E-17	3.35E-15
469	ENSG00000197965.11	MPZL1	-0.77	8.21E-17	3.47E-15
470	ENSG00000133121.20	STARD13	-0.90	9.32E-17	3.93E-15
471	ENSG00000203799.12	CCDC162P	1.69	9.49E-17	4.00E-15
472	ENSG00000076662.9	ICAM3	1.39	9.54E-17	4.01E-15
473	ENSG00000183779.6	ZNF703	-1.35	1.11E-16	4.65E-15
474	ENSG00000179104.8	TMTC2	0.90	1.14E-16	4.79E-15
475	ENSG00000145990.10	GFOD1	1.20	1.37E-16	5.71E-15
476	ENSG00000095209.11	TMEM38B	1.07	1.37E-16	5.72E-15
477	ENSG00000149084.12	HSD17B12	1.00	1.38E-16	5.72E-15
478	ENSG00000000003.14	TSPAN6	0.91	1.61E-16	6.66E-15
479	ENSG00000025039.14	RRAGD	1.78	1.62E-16	6.70E-15
480	ENSG00000155016.17	CYP2U1	-1.07	1.67E-16	6.90E-15
481	ENSG00000091436.16	MAP3K20	-0.72	1.69E-16	6.97E-15
482	ENSG00000169946.13	ZFPM2	-1.22	1.76E-16	7.23E-15
483	ENSG00000260912.1	AL158206.1	1.66	1.93E-16	7.94E-15
484	ENSG00000126602.10	TRAP1	0.82	1.98E-16	8.11E-15
485	ENSG00000131951.11	LRRC9	4.58	2.09E-16	8.56E-15
486	ENSG00000164825.3	DEFB1	3.48	2.15E-16	8.76E-15
487	ENSG00000263535.2	AK4P1	1.51	2.20E-16	8.96E-15
488	ENSG00000151150.21	ANK3	1.10	2.25E-16	9.15E-15
489	ENSG00000067113.16	PLPP1	-0.83	2.27E-16	9.22E-15
490	ENSG00000147852.15	VLDLR	0.88	2.54E-16	1.03E-14
491	ENSG00000035862.12	TIMP2	-0.75	2.90E-16	1.17E-14
492	ENSG00000196092.12	PAX5	1.87	3.24E-16	1.31E-14
493	ENSG00000185909.14	KLHDC8B	1.16	3.72E-16	1.50E-14
494	ENSG00000079435.9	LIPE	1.15	3.73E-16	1.50E-14
495	ENSG00000104450.12	SPAG1	1.47	3.82E-16	1.53E-14
496	ENSG00000213904.8	LIPE-AS1	2.12	3.84E-16	1.54E-14
497	ENSG00000164188.8	RANBP3L	2.52	3.85E-16	1.54E-14

498	ENSG00000140848.16	CPNE2	0.93	4.16E-16	1.66E-14
499	ENSG00000185880.12	TRIM69	0.92	4.22E-16	1.68E-14
500	ENSG00000148175.12	STOM	1.03	4.53E-16	1.80E-14
501	ENSG00000137965.10	IFI44	-1.60	4.73E-16	1.87E-14
502	ENSG00000215386.12	MIR99AHG	-1.32	5.56E-16	2.19E-14
503	ENSG00000148516.21	ZEB1	-0.87	5.79E-16	2.28E-14
504	ENSG00000172986.12	GXYLT2	0.84	5.82E-16	2.29E-14
505	ENSG00000225206.9	MIR137HG	-1.85	5.84E-16	2.29E-14
506	ENSG00000244062.1	AC080128.1	2.68	5.91E-16	2.32E-14
507	ENSG00000198216.11	CACNA1E	3.95	6.56E-16	2.57E-14
508	ENSG00000254990.5	AP001781.1	2.88	6.66E-16	2.60E-14
509	ENSG00000146955.10	RAB19	6.38	6.70E-16	2.61E-14
510	ENSG00000182718.16	ANXA2	-0.94	6.89E-16	2.68E-14
511	ENSG00000107242.17	PIP5K1B	2.02	7.21E-16	2.80E-14
512	ENSG00000122971.8	ACADS	1.41	7.62E-16	2.95E-14
513	ENSG00000148671.13	ADIRF	1.04	8.75E-16	3.38E-14
514	ENSG00000172893.15	DHCR7	1.26	9.42E-16	3.64E-14
515	ENSG00000144362.11	PHOSPHO2	1.81	1.00E-15	3.87E-14
516	ENSG00000166963.12	MAP1A	-0.86	1.18E-15	4.53E-14
517	ENSG00000152582.13	SPEF2	1.52	1.24E-15	4.76E-14
518	ENSG00000152256.13	PDK1	1.09	1.44E-15	5.53E-14
519	ENSG00000259407.1	AC021739.2	2.24	1.48E-15	5.67E-14
520	ENSG00000162733.17	DDR2	-0.86	1.71E-15	6.52E-14
521	ENSG00000050426.15	LETMD1	0.79	1.79E-15	6.80E-14
522	ENSG00000150995.19	ITPR1	-0.92	1.80E-15	6.84E-14
523	ENSG00000164484.11	TMEM200A	-1.19	2.07E-15	7.86E-14
524	ENSG00000107338.9	SHB	1.35	2.22E-15	8.41E-14
525	ENSG00000167264.17	DUS2	0.93	2.39E-15	9.04E-14
526	ENSG00000158769.17	F11R	0.95	2.89E-15	1.09E-13
527	ENSG00000138018.17	SELENOI	0.94	2.96E-15	1.12E-13
528	ENSG00000137834.14	SMAD6	1.14	3.38E-15	1.27E-13
529	ENSG00000095015.5	MAP3K1	1.21	3.52E-15	1.32E-13
530	ENSG00000131323.14	TRAF3	0.88	3.58E-15	1.34E-13
531	ENSG00000140853.15	NLRC5	0.96	3.89E-15	1.45E-13
532	ENSG00000122756.14	CNTFR	1.24	3.95E-15	1.47E-13
533	ENSG00000243978.8	RTL9	2.49	4.02E-15	1.50E-13
534	ENSG00000255398.2	HCAR3	1.37	4.09E-15	1.52E-13
535	ENSG00000174804.3	FZD4	1.07	4.10E-15	1.52E-13
536	ENSG00000115977.18	AAK1	0.83	4.12E-15	1.52E-13
537	ENSG00000003147.18	ICA1	1.08	4.34E-15	1.60E-13
538	ENSG00000091490.10	SEL1L3	-0.98	4.33E-15	1.60E-13
539	ENSG00000111817.17	DSE	-0.97	4.38E-15	1.61E-13
540	ENSG00000221932.6	HEPN1	1.52	4.67E-15	1.72E-13
541	ENSG00000059691.11	GATB	0.88	4.78E-15	1.75E-13
542	ENSG00000165030.3	NFIL3	-1.11	4.77E-15	1.75E-13
543	ENSG00000186479.4	RGS7BP	2.48	4.95E-15	1.81E-13
544	ENSG00000169021.5	UQCRFS1	0.79	5.08E-15	1.85E-13
545	ENSG00000167703.14	SLC43A2	-1.16	5.15E-15	1.87E-13
546	ENSG00000182533.6	CAV3	4.58	5.19E-15	1.88E-13
547	ENSG00000197142.10	ACSL5	2.50	5.24E-15	1.90E-13

548	ENSG00000132840.9	BHMT2	1.04	5.30E-15	1.92E-13
549	ENSG00000139832.4	RAB20	1.84	5.52E-15	1.99E-13
550	ENSG00000087303.17	NID2	-1.09	5.53E-15	2.00E-13
551	ENSG00000271447.5	MMP28	-3.58	5.58E-15	2.01E-13
552	ENSG00000165914.14	TTC7B	0.84	5.59E-15	2.01E-13
553	ENSG00000189420.8	ZFP92	1.92	5.71E-15	2.05E-13
554	ENSG00000166405.14	RIC3	2.94	5.73E-15	2.05E-13
555	ENSG00000158571.10	PFKFB1	0.85	5.85E-15	2.09E-13
556	ENSG00000120756.12	PLS1	1.76	5.90E-15	2.10E-13
557	ENSG00000166106.3	ADAMTS15	1.37	5.98E-15	2.13E-13
558	ENSG00000263155.5	MYZAP	1.16	6.25E-15	2.22E-13
559	ENSG00000197321.14	SVIL	1.22	6.58E-15	2.33E-13
560	ENSG00000053524.12	MCF2L2	2.32	6.66E-15	2.36E-13
561	ENSG00000110047.17	EHD1	0.92	6.77E-15	2.39E-13
562	ENSG00000163812.13	ZDHHC3	0.74	6.80E-15	2.40E-13
563	ENSG00000117791.15	MARC2	0.90	7.18E-15	2.53E-13
564	ENSG00000139641.12	ESYT1	0.77	7.77E-15	2.73E-13
565	ENSG00000146278.10	PNRC1	-0.99	8.00E-15	2.81E-13
566	ENSG00000011028.13	MRC2	-1.25	8.03E-15	2.81E-13
567	ENSG00000151365.2	THRSP	0.87	8.24E-15	2.88E-13
568	ENSG00000173267.13	SNCG	-1.73	8.46E-15	2.95E-13
569	ENSG00000134463.14	ECHDC3	1.14	8.52E-15	2.97E-13
570	ENSG00000167705.11	RILP	0.96	8.60E-15	2.99E-13
571	ENSG00000185924.6	RTN4RL1	1.61	8.62E-15	3.00E-13
572	ENSG00000147684.8	NDUFB9	0.71	9.28E-15	3.22E-13
573	ENSG00000186847.5	KRT14	1.55	9.34E-15	3.23E-13
574	ENSG00000136143.15	SUCLA2	0.89	9.82E-15	3.39E-13
575	ENSG00000169599.12	NFU1	1.31	1.08E-14	3.73E-13
576	ENSG00000188549.12	CCDC9B	-1.43	1.12E-14	3.85E-13
577	ENSG00000155368.16	DBI	0.67	1.15E-14	3.94E-13
578	ENSG00000143149.12	ALDH9A1	1.02	1.15E-14	3.95E-13
579	ENSG00000112972.14	HMGCS1	0.78	1.23E-14	4.21E-13
580	ENSG00000155886.11	SLC24A2	3.30	1.35E-14	4.61E-13
581	ENSG00000189077.10	TMEM120A	0.94	1.36E-14	4.62E-13
582	ENSG00000073849.14	ST6GAL1	-1.95	1.36E-14	4.62E-13
583	ENSG00000141622.13	RNF165	2.03	1.37E-14	4.67E-13
584	ENSG00000005059.15	MCUB	0.88	1.43E-14	4.86E-13
585	ENSG00000243989.8	ACY1	1.08	1.48E-14	5.01E-13
586	ENSG00000197386.12	HTT	0.87	1.59E-14	5.39E-13
587	ENSG00000254413.8	CHKB-CPT1B	4.15	1.62E-14	5.46E-13
588	ENSG00000126106.13	TMEM53	1.01	1.81E-14	6.10E-13
589	ENSG00000055957.10	ITIH1	2.95	1.84E-14	6.20E-13
590	ENSG00000120068.6	HOXB8	1.13	1.89E-14	6.35E-13
591	ENSG00000160049.11	DFFA	0.74	2.02E-14	6.79E-13
592	ENSG00000163083.5	INHBB	1.92	2.05E-14	6.86E-13
593	ENSG00000140009.18	ESR2	1.46	2.10E-14	7.01E-13
594	ENSG00000279278.1	AC245060.6	1.23	2.22E-14	7.43E-13
595	ENSG00000113645.14	WWC1	1.52	2.29E-14	7.64E-13
596	ENSG00000141298.18	SSH2	-1.00	2.31E-14	7.68E-13
597	ENSG00000251448.1	AC063919.1	3.34	2.32E-14	7.71E-13

598	ENSG00000143507.17	DUSP10	1.13	2.34E-14	7.75E-13
599	ENSG00000260062.3	GOLGA2P11	3.00	2.39E-14	7.90E-13
600	ENSG00000139117.13	CPNE8	-1.48	2.83E-14	9.34E-13
601	ENSG00000159479.16	MED8	0.83	3.03E-14	1.00E-12
602	ENSG00000169302.15	STK32A	-1.68	3.35E-14	1.10E-12
603	ENSG00000140961.12	OSGIN1	1.20	3.66E-14	1.20E-12
604	ENSG00000110917.7	MLEC	0.81	3.65E-14	1.20E-12
605	ENSG00000019995.6	ZRANB1	0.82	3.82E-14	1.25E-12
606	ENSG00000254965.1	C1DP5	3.31	3.87E-14	1.27E-12
607	ENSG00000144031.11	ANKRD53	3.62	3.92E-14	1.28E-12
608	ENSG00000104870.12	FCGRT	0.88	4.50E-14	1.47E-12
609	ENSG00000236824.2	BCYRN1	1.44	4.66E-14	1.52E-12
610	ENSG00000101134.11	DOK5	-0.90	4.83E-14	1.57E-12
611	ENSG00000137210.13	TMEM14B	0.80	5.38E-14	1.75E-12
612	ENSG00000132185.16	FCRLA	1.49	5.44E-14	1.76E-12
613	ENSG00000156928.4	MALSU1	0.92	5.71E-14	1.85E-12
614	ENSG00000158864.12	NDUFS2	0.68	5.83E-14	1.88E-12
615	ENSG00000127124.15	HIVEP3	1.15	5.86E-14	1.89E-12
616	ENSG00000147883.10	CDKN2B	-0.88	6.33E-14	2.04E-12
617	ENSG00000182810.6	DDX28	1.12	6.60E-14	2.12E-12
618	ENSG00000120658.13	ENOX1	1.48	6.63E-14	2.13E-12
619	ENSG00000162836.11	ACP6	1.10	7.03E-14	2.25E-12
620	ENSG00000108039.17	XPNPEP1	0.84	7.20E-14	2.30E-12
621	ENSG00000122783.16	CYREN	0.98	7.31E-14	2.34E-12
622	ENSG00000157693.14	TMEM268	0.96	7.46E-14	2.38E-12
623	ENSG00000092969.11	TGFB2	-1.67	7.82E-14	2.49E-12
624	ENSG00000131981.15	LGALS3	-1.01	8.25E-14	2.62E-12
625	ENSG00000234745.10	HLA-B	0.96	8.42E-14	2.67E-12
626	ENSG00000165197.4	VEGFD	-1.76	8.63E-14	2.74E-12
627	ENSG00000100280.16	AP1B1	0.78	9.19E-14	2.91E-12
628	ENSG00000274956.2	NKAIN3-IT1	2.32	9.23E-14	2.92E-12
629	ENSG00000173166.17	RAPH1	-0.79	9.53E-14	3.01E-12
630	ENSG00000233006.7	MIR3936HG	1.91	9.61E-14	3.03E-12
631	ENSG00000170989.9	S1PR1	-2.03	9.79E-14	3.08E-12
632	ENSG00000206337.10	HCP5	1.71	1.05E-13	3.30E-12
633	ENSG00000109519.12	GRPEL1	0.93	1.06E-13	3.31E-12
634	ENSG00000185215.8	TNFAIP2	-0.98	1.08E-13	3.38E-12
635	ENSG00000146250.6	PRSS35	-1.60	1.09E-13	3.39E-12
636	ENSG00000159335.15	PTMS	-1.22	1.11E-13	3.46E-12
637	ENSG00000111666.10	CHPT1	0.86	1.22E-13	3.80E-12
638	ENSG00000163686.14	ABHD6	1.03	1.22E-13	3.81E-12
639	ENSG00000134317.17	GRHL1	1.86	1.24E-13	3.84E-12
640	ENSG00000066468.22	FGFR2	2.45	1.25E-13	3.87E-12
641	ENSG00000047634.14	SCML1	0.77	1.25E-13	3.87E-12
642	ENSG00000267383.6	AC011447.3	2.11	1.26E-13	3.89E-12
643	ENSG00000119915.4	ELOVL3	1.10	1.26E-13	3.90E-12
644	ENSG00000152749.7	GPR180	0.83	1.29E-13	3.98E-12
645	ENSG00000214189.9	ZNF788P	1.17	1.30E-13	4.00E-12
646	ENSG00000067704.9	IARS2	0.66	1.30E-13	4.00E-12
647	ENSG00000082996.19	RNF13	0.83	1.35E-13	4.13E-12

648	ENSG00000134243.11	SORT1	0.73	1.35E-13	4.13E-12
649	ENSG00000073417.14	PDE8A	1.01	1.36E-13	4.15E-12
650	ENSG00000006756.15	ARSD	-1.03	1.42E-13	4.34E-12
651	ENSG00000075618.17	FSCN1	-1.32	1.45E-13	4.41E-12
652	ENSG00000110435.11	PDHX	0.69	1.47E-13	4.46E-12
653	ENSG00000182902.13	SLC25A18	1.24	1.49E-13	4.53E-12
654	ENSG00000175556.16	LONRF3	1.78	1.50E-13	4.56E-12
655	ENSG00000161267.11	BDH1	0.94	1.62E-13	4.89E-12
656	ENSG00000227848.1	SUCLA2-AS1	3.92	1.62E-13	4.89E-12
657	ENSG00000066583.11	ISOC1	1.04	1.63E-13	4.91E-12
658	ENSG00000162129.13	CLPB	0.86	1.70E-13	5.13E-12
659	ENSG00000131969.14	ABHD12B	2.27	1.79E-13	5.39E-12
660	ENSG00000111775.2	COX6A1	0.73	1.80E-13	5.42E-12
661	ENSG00000152556.16	PFKM	-0.90	1.81E-13	5.43E-12
662	ENSG00000164405.10	UQCRQ	0.69	1.82E-13	5.47E-12
663	ENSG00000109929.9	SC5D	0.82	1.84E-13	5.50E-12
664	ENSG00000150764.13	DIXDC1	0.79	1.84E-13	5.50E-12
665	ENSG00000114054.13	PCCB	0.68	2.41E-13	7.18E-12
666	ENSG00000163399.15	ATP1A1	-0.90	2.48E-13	7.39E-12
667	ENSG00000181019.12	NQO1	-1.20	2.50E-13	7.43E-12
668	ENSG00000161091.12	MFSD12	0.88	2.62E-13	7.79E-12
669	ENSG00000007350.16	TKTL1	5.14	2.64E-13	7.83E-12
670	ENSG00000224389.9	C4B	1.25	2.66E-13	7.89E-12
671	ENSG00000165474.6	GJB2	-1.47	2.81E-13	8.31E-12
672	ENSG00000111684.10	LPCAT3	0.90	2.94E-13	8.67E-12
673	ENSG00000110497.14	AMBRA1	0.93	2.98E-13	8.79E-12
674	ENSG00000213949.8	ITGA1	1.09	3.09E-13	9.09E-12
675	ENSG00000163344.5	PMVK	0.80	3.18E-13	9.35E-12
676	ENSG00000122176.11	FMOD	-1.69	3.24E-13	9.52E-12
677	ENSG00000163840.9	DTX3L	1.19	3.26E-13	9.55E-12
678	ENSG00000162461.7	SLC25A34	2.31	3.55E-13	1.04E-11
679	ENSG00000151474.22	FRMD4A	0.71	3.68E-13	1.07E-11
680	ENSG00000284428.1	IPO5P1	1.32	3.97E-13	1.16E-11
681	ENSG00000078124.11	ACER3	1.02	3.99E-13	1.16E-11
682	ENSG00000137752.23	CASP1	1.51	4.08E-13	1.19E-11
683	ENSG00000067225.17	PKM	-1.05	4.42E-13	1.29E-11
684	ENSG00000129151.8	BBOX1	1.14	4.64E-13	1.34E-11
685	ENSG00000182263.13	FIGN	1.07	4.64E-13	1.34E-11
686	ENSG00000162817.6	C1orf115	-1.17	4.71E-13	1.36E-11
687	ENSG00000171992.12	SYNPO	1.16	4.73E-13	1.36E-11
688	ENSG00000187105.8	HEATR4	2.31	4.85E-13	1.40E-11
689	ENSG00000142920.16	AZIN2	1.37	5.02E-13	1.45E-11
690	ENSG00000249173.5	LINC01093	2.15	5.23E-13	1.50E-11
691	ENSG00000124214.19	STAU1	0.69	6.72E-13	1.93E-11
692	ENSG00000168528.11	SERINC2	1.03	6.73E-13	1.93E-11
693	ENSG00000163075.12	CFAP221	1.80	6.87E-13	1.96E-11
694	ENSG00000112149.9	CD83	1.19	6.86E-13	1.96E-11
695	ENSG00000182782.7	HCAR2	0.77	7.40E-13	2.11E-11
696	ENSG00000147160.9	AWAT2	3.31	7.44E-13	2.12E-11
697	ENSG00000026508.18	CD44	-1.12	7.74E-13	2.20E-11

698	ENSG00000285283.1	AL035078.4	1.06	7.78E-13	2.21E-11
699	ENSG00000182253.14	SYNM	0.64	7.79E-13	2.21E-11
700	ENSG00000148513.17	ANKRD30A	2.04	7.85E-13	2.22E-11
701	ENSG00000162402.13	USP24	0.74	7.86E-13	2.22E-11
702	ENSG00000154545.16	MAGED4	1.38	7.89E-13	2.23E-11
703	ENSG00000024422.11	EHD2	-0.79	7.93E-13	2.24E-11
704	ENSG00000245293.2	AC096564.1	2.50	8.35E-13	2.35E-11
705	ENSG00000113790.10	EHHADH	0.96	8.54E-13	2.40E-11
706	ENSG00000170921.15	TANC2	0.82	8.70E-13	2.45E-11
707	ENSG00000137266.14	SLC22A23	0.72	1.01E-12	2.83E-11
708	ENSG00000139263.11	LRIG3	-0.88	1.03E-12	2.87E-11
709	ENSG00000176014.12	TUBB6	0.90	1.03E-12	2.89E-11
710	ENSG00000104419.14	NDRG1	-0.88	1.04E-12	2.91E-11
711	ENSG00000162849.15	KIF26B	1.18	1.09E-12	3.05E-11
712	ENSG00000085721.12	RRN3	0.79	1.11E-12	3.09E-11
713	ENSG00000130876.11	SLC7A10	2.26	1.12E-12	3.12E-11
714	ENSG00000244731.7	C4A	1.16	1.12E-12	3.12E-11
715	ENSG00000106780.8	MEGF9	0.84	1.15E-12	3.19E-11
716	ENSG00000182326.14	C1S	-0.76	1.16E-12	3.21E-11
717	ENSG00000171914.16	TLN2	0.68	1.17E-12	3.25E-11
718	ENSG00000162391.11	FAM151A	2.38	1.21E-12	3.35E-11
719	ENSG00000167107.12	ACSF2	0.86	1.29E-12	3.56E-11
720	ENSG00000138073.13	PREB	0.76	1.47E-12	4.05E-11
721	ENSG00000072062.13	PRKACA	0.68	1.48E-12	4.07E-11
722	ENSG00000073910.21	FRY	-0.77	1.49E-12	4.09E-11
723	ENSG00000183098.10	GPC6	-0.94	1.55E-12	4.26E-11
724	ENSG00000176422.13	SPRYD4	0.91	1.58E-12	4.34E-11
725	ENSG00000145832.14	SLC25A48	3.41	1.60E-12	4.38E-11
726	ENSG00000172115.8	CYCS	0.79	1.68E-12	4.59E-11
727	ENSG00000163629.12	PTPN13	0.74	1.76E-12	4.81E-11
728	ENSG00000158816.15	VWA5B1	3.88	1.80E-12	4.90E-11
729	ENSG00000153071.14	DAB2	-0.75	1.85E-12	5.04E-11
730	ENSG00000057704.12	TMCC3	3.41	1.93E-12	5.24E-11
731	ENSG00000138496.16	PARP9	1.23	1.97E-12	5.35E-11
732	ENSG00000164182.10	NDUFAF2	1.00	1.99E-12	5.38E-11
733	ENSG00000132386.10	SERPINF1	0.65	2.04E-12	5.52E-11
734	ENSG00000148672.8	GLUD1	0.59	2.10E-12	5.68E-11
735	ENSG00000047648.21	ARHGAP6	-1.65	2.12E-12	5.71E-11
736	ENSG00000106034.17	CPED1	1.12	2.20E-12	5.93E-11
737	ENSG00000258227.6	CLEC5A	3.34	2.25E-12	6.07E-11
738	ENSG00000173918.14	C1QTNF1	-0.97	2.30E-12	6.18E-11
739	ENSG00000277737.3	FP325317.1	2.07	2.36E-12	6.33E-11
740	ENSG00000071073.12	MGAT4A	1.30	2.42E-12	6.49E-11
741	ENSG00000152049.6	KCNE4	-0.87	2.50E-12	6.70E-11
742	ENSG00000100092.22	SH3BP1	2.17	2.79E-12	7.46E-11
743	ENSG00000244026.6	FAM86DP	0.82	3.04E-12	8.12E-11
744	ENSG00000121316.10	PLBD1	1.75	3.31E-12	8.84E-11
745	ENSG00000274173.1	AL035661.1	1.98	3.37E-12	8.97E-11
746	ENSG00000138162.18	TACC2	0.93	3.40E-12	9.04E-11
747	ENSG00000203797.9	DDO	2.07	3.43E-12	9.12E-11

748	ENSG00000081377.16	CDC14B	-0.72	3.69E-12	9.79E-11
749	ENSG00000186918.13	ZNF395	-1.01	3.79E-12	1.01E-10
750	ENSG00000132932.16	ATP8A2	2.89	3.89E-12	1.03E-10
751	ENSG00000162520.14	SYNC	-0.76	4.00E-12	1.06E-10
752	ENSG00000112964.13	GHR	0.83	4.09E-12	1.08E-10
753	ENSG00000014164.6	ZC3H3	1.31	4.24E-12	1.12E-10
754	ENSG00000180817.11	PPA1	0.98	5.22E-12	1.37E-10
755	ENSG00000225855.6	RUSC1-AS1	-0.95	5.27E-12	1.38E-10
756	ENSG00000285508.1	AL034430.1	1.63	5.49E-12	1.44E-10
757	ENSG00000196465.10	MYL6B	0.91	5.67E-12	1.49E-10
758	ENSG00000198670.11	LPA	4.72	5.79E-12	1.52E-10
759	ENSG00000134247.9	PTGFRN	0.89	5.87E-12	1.53E-10
760	ENSG00000215012.8	RTL10	0.80	5.88E-12	1.53E-10
761	ENSG00000223678.1	AL356218.1	2.34	6.45E-12	1.68E-10
762	ENSG00000114771.13	AADAC	2.56	6.49E-12	1.69E-10
763	ENSG00000168827.14	GFM1	0.67	6.48E-12	1.69E-10
764	ENSG00000170634.12	ACYP2	0.91	6.52E-12	1.69E-10
765	ENSG00000129682.15	FGF13	1.47	6.58E-12	1.71E-10
766	ENSG00000044115.20	CTNNA1	-0.56	6.84E-12	1.77E-10
767	ENSG00000113209.8	PCDHB5	-1.23	6.84E-12	1.77E-10
768	ENSG00000121691.5	CAT	0.65	7.32E-12	1.89E-10
769	ENSG00000177666.16	PNPLA2	0.89	7.44E-12	1.92E-10
770	ENSG00000168291.12	PDHB	0.70	7.56E-12	1.95E-10
771	ENSG00000088367.21	EPB41L1	-0.95	7.58E-12	1.95E-10
772	ENSG00000159388.5	BTG2	-1.02	8.00E-12	2.06E-10
773	ENSG00000109861.15	CTSC	1.03	8.75E-12	2.25E-10
774	ENSG00000182575.7	NXPH3	-2.81	8.83E-12	2.27E-10
775	ENSG00000255248.8	MIR100HG	-0.84	9.08E-12	2.32E-10
776	ENSG00000182463.15	TSHZ2	-1.01	9.07E-12	2.32E-10
777	ENSG00000125089.16	SH3TC1	1.48	9.16E-12	2.34E-10
778	ENSG00000213533.11	STIMATE	0.72	9.27E-12	2.37E-10
779	ENSG00000180773.14	SLC36A4	0.97	9.30E-12	2.37E-10
780	ENSG00000186654.20	PRR5	1.20	9.46E-12	2.41E-10
781	ENSG00000184601.10	C14orf180	1.04	9.52E-12	2.42E-10
782	ENSG00000104447.12	TRPS1	-0.76	9.54E-12	2.42E-10
783	ENSG00000183579.15	ZNRF3	-0.92	9.68E-12	2.45E-10
784	ENSG00000269926.1	DDIT4-AS1	1.22	9.96E-12	2.52E-10
785	ENSG00000158186.12	MRAS	0.67	9.99E-12	2.53E-10
786	ENSG00000139428.11	MMAB	0.78	1.01E-11	2.56E-10
787	ENSG00000119138.4	KLF9	-0.86	1.02E-11	2.57E-10
788	ENSG00000023330.14	ALAS1	0.68	1.06E-11	2.67E-10
789	ENSG00000125772.12	GPCPD1	-0.75	1.08E-11	2.72E-10
790	ENSG00000071054.16	MAP4K4	-0.62	1.10E-11	2.77E-10
791	ENSG00000135362.13	PRR5L	1.76	1.12E-11	2.82E-10
792	ENSG00000101019.21	UQCC1	0.62	1.13E-11	2.83E-10
793	ENSG00000091986.15	CCDC80	-0.71	1.17E-11	2.92E-10
794	ENSG00000057252.12	SOAT1	0.81	1.22E-11	3.04E-10
795	ENSG00000186466.5	AQP7P1	3.70	1.29E-11	3.23E-10
796	ENSG00000140873.15	ADAMTS18	1.16	1.32E-11	3.29E-10
797	ENSG00000167470.12	MIDN	-1.08	1.37E-11	3.40E-10

798	ENSG00000176971.3	FIBIN	-1.38	1.37E-11	3.40E-10
799	ENSG00000257017.8	HP	2.90	1.43E-11	3.56E-10
800	ENSG00000204525.16	HLA-C	0.78	1.44E-11	3.57E-10
801	ENSG00000171724.2	VAT1L	-1.40	1.46E-11	3.62E-10
802	ENSG00000162434.11	JAK1	0.61	1.47E-11	3.65E-10
803	ENSG00000023734.10	STRAP	0.61	1.51E-11	3.74E-10
804	ENSG00000168807.16	SNTB2	-0.68	1.54E-11	3.80E-10
805	ENSG00000116353.15	MECR	0.83	1.59E-11	3.91E-10
806	ENSG00000121104.7	FAM117A	1.14	1.61E-11	3.96E-10
807	ENSG00000091140.13	DLD	0.62	1.61E-11	3.97E-10
808	ENSG00000183506.17	PI4KAP2	0.92	1.69E-11	4.14E-10
809	ENSG00000010704.18	HFE	1.06	1.70E-11	4.17E-10
810	ENSG00000244998.1	AC100803.1	2.04	1.76E-11	4.31E-10
811	ENSG00000131828.13	PDHA1	0.74	1.82E-11	4.44E-10
812	ENSG00000092964.17	DPYSL2	-0.62	1.85E-11	4.53E-10
813	ENSG00000186298.11	PPP1CC	0.73	1.90E-11	4.64E-10
814	ENSG00000004399.12	PLXND1	-0.90	1.94E-11	4.72E-10
815	ENSG00000116221.15	MRPL37	0.72	1.95E-11	4.76E-10
816	ENSG00000115306.15	SPTBN1	0.67	2.04E-11	4.96E-10
817	ENSG00000159231.5	CBR3	-1.53	2.09E-11	5.07E-10
818	ENSG00000245060.6	LINC00847	1.11	2.15E-11	5.22E-10
819	ENSG00000154493.18	C10orf90	1.65	2.16E-11	5.24E-10
820	ENSG00000082212.12	ME2	0.68	2.23E-11	5.39E-10
821	ENSG00000012232.8	EXTL3	0.65	2.24E-11	5.41E-10
822	ENSG00000187288.10	CIDEC	0.63	2.28E-11	5.50E-10
823	ENSG00000134297.6	PLEKHA8P1	1.14	2.30E-11	5.55E-10
824	ENSG00000117676.13	RPS6KA1	2.20	2.37E-11	5.71E-10
825	ENSG00000006432.15	MAP3K9	1.99	2.47E-11	5.93E-10
826	ENSG00000139874.5	SSTR1	-0.88	2.56E-11	6.15E-10
827	ENSG00000134352.19	IL6ST	-0.70	2.65E-11	6.35E-10
828	ENSG00000100154.14	TTC28	-0.74	2.71E-11	6.49E-10
829	ENSG00000261522.5	AL845331.3	4.17	2.72E-11	6.51E-10
830	ENSG00000143368.9	SF3B4	0.82	2.83E-11	6.76E-10
831	ENSG00000078070.12	MCCC1	0.77	2.88E-11	6.88E-10
832	ENSG00000236404.9	VLDLR-AS1	1.36	2.94E-11	7.01E-10
833	ENSG00000183978.7	COA3	0.77	2.96E-11	7.05E-10
834	ENSG00000163346.16	PBXIP1	-0.74	2.96E-11	7.05E-10
835	ENSG00000148848.14	ADAM12	0.92	3.00E-11	7.14E-10
836	ENSG00000138593.8	SECISBP2L	0.72	3.04E-11	7.23E-10
837	ENSG00000132361.16	CLUH	0.83	3.25E-11	7.72E-10
838	ENSG00000163683.11	SMIM14	-0.64	3.31E-11	7.85E-10
839	ENSG00000153823.18	PID1	-1.19	3.57E-11	8.44E-10
840	ENSG00000099875.14	MKNK2	0.93	3.63E-11	8.58E-10
841	ENSG00000169814.13	BTD	0.69	3.74E-11	8.81E-10
842	ENSG00000187837.3	HIST1H1C	0.70	3.87E-11	9.12E-10
843	ENSG00000006042.11	TMEM98	-0.81	3.92E-11	9.23E-10
844	ENSG00000185825.16	BCAP31	0.59	3.94E-11	9.26E-10
845	ENSG00000168461.12	RAB31	-0.74	3.97E-11	9.32E-10
846	ENSG00000139178.10	C1RL	-0.91	4.02E-11	9.43E-10
847	ENSG00000173660.11	UQCRH	0.67	4.55E-11	1.07E-09

848	ENSG00000270412.1	AL136084.3	-2.22	4.57E-11	1.07E-09
849	ENSG00000108639.7	SYNGR2	0.72	4.58E-11	1.07E-09
850	ENSG00000023572.9	GLRX2	0.96	4.73E-11	1.10E-09
851	ENSG00000168939.11	SPRY3	0.84	4.73E-11	1.10E-09
852	ENSG00000138613.13	APH1B	-0.84	4.78E-11	1.11E-09
853	ENSG00000133028.11	SCO1	0.63	5.02E-11	1.17E-09
854	ENSG00000121236.20	TRIM6	1.24	5.05E-11	1.17E-09
855	ENSG00000196739.14	COL27A1	1.12	5.10E-11	1.18E-09
856	ENSG00000154079.5	SDHAF4	0.87	5.15E-11	1.19E-09
857	ENSG00000166575.16	TMEM135	0.78	5.26E-11	1.22E-09
858	ENSG00000100078.3	PLA2G3	-2.62	5.58E-11	1.29E-09
859	ENSG00000136425.13	CIB2	1.41	5.59E-11	1.29E-09
860	ENSG00000108861.8	DUSP3	0.72	5.59E-11	1.29E-09
861	ENSG00000198689.11	SLC9A6	0.72	5.61E-11	1.29E-09
862	ENSG00000102606.18	ARHGEF7	0.70	6.03E-11	1.39E-09
863	ENSG00000197757.7	HOXC6	-0.74	6.10E-11	1.40E-09
864	ENSG00000151883.17	PARP8	1.20	6.22E-11	1.43E-09
865	ENSG00000267751.5	AC009005.1	1.12	6.23E-11	1.43E-09
866	ENSG00000167552.13	TUBA1A	-0.77	6.27E-11	1.44E-09
867	ENSG00000198624.12	CCDC69	0.94	6.68E-11	1.53E-09
868	ENSG00000105974.11	CAV1	-0.87	6.70E-11	1.53E-09
869	ENSG00000147065.16	MSN	-0.57	6.87E-11	1.57E-09
870	ENSG00000145362.17	ANK2	0.80	6.97E-11	1.59E-09
871	ENSG00000136870.10	ZNF189	0.77	7.13E-11	1.62E-09
872	ENSG00000198574.5	SH2D1B	6.84	7.25E-11	1.65E-09
873	ENSG00000161813.21	LARP4	0.67	7.43E-11	1.69E-09
874	ENSG00000118922.17	KLF12	-0.86	7.47E-11	1.69E-09
875	ENSG00000096080.11	MRPS18A	0.72	7.54E-11	1.71E-09
876	ENSG00000224531.5	SMIM13	0.68	7.56E-11	1.71E-09
877	ENSG00000196365.11	LONP1	0.79	7.90E-11	1.79E-09
878	ENSG00000050555.17	LAMC3	3.57	7.99E-11	1.80E-09
879	ENSG00000273489.1	AC008264.2	-2.36	8.15E-11	1.84E-09
880	ENSG00000146416.18	AIG1	0.82	8.20E-11	1.85E-09
881	ENSG00000186204.14	CYP4F12	0.99	8.30E-11	1.87E-09
882	ENSG00000149257.14	SERPINH1	-0.90	8.30E-11	1.87E-09
883	ENSG00000154734.14	ADAMTS1	-0.92	8.34E-11	1.87E-09
884	ENSG00000136830.11	FAM129B	-0.92	8.43E-11	1.89E-09
885	ENSG00000167815.11	PRDX2	0.68	8.52E-11	1.91E-09
886	ENSG00000198157.10	HMGN5	1.15	8.79E-11	1.97E-09
887	ENSG00000066322.14	ELOVL1	0.70	8.81E-11	1.97E-09
888	ENSG00000067798.15	NAV3	-0.61	8.93E-11	1.99E-09
889	ENSG00000274180.1	NATD1	0.73	9.07E-11	2.02E-09
890	ENSG00000176871.8	WSB2	0.64	9.19E-11	2.05E-09
891	ENSG00000067445.20	TRO	0.75	9.30E-11	2.07E-09
892	ENSG00000082641.15	NFE2L1	-0.77	9.30E-11	2.07E-09
893	ENSG00000196878.14	LAMB3	-1.35	9.36E-11	2.08E-09
894	ENSG00000135916.15	ITM2C	0.69	9.49E-11	2.10E-09
895	ENSG00000169718.17	DUS1L	0.80	1.02E-10	2.27E-09
896	ENSG00000260300.5	AC009119.2	1.36	1.06E-10	2.34E-09
897	ENSG00000236618.2	PITPNA-AS1	1.50	1.06E-10	2.35E-09

898	ENSG00000168209.4	DDIT4	1.20	1.10E-10	2.42E-09
899	ENSG00000184156.16	KCNQ3	3.76	1.10E-10	2.43E-09
900	ENSG00000106991.13	ENG	-0.88	1.13E-10	2.49E-09
901	ENSG00000203280.4	AL022323.1	3.26	1.13E-10	2.50E-09
902	ENSG00000240184.6	PCDHGC3	-0.72	1.25E-10	2.75E-09
903	ENSG00000110442.11	COMMD9	0.73	1.26E-10	2.76E-09
904	ENSG00000214313.8	AZGP1P1	-1.88	1.28E-10	2.81E-09
905	ENSG00000196502.11	SULT1A1	-1.19	1.35E-10	2.96E-09
906	ENSG00000065183.15	WDR3	0.88	1.36E-10	2.99E-09
907	ENSG00000279312.1	AL136164.4	-1.42	1.37E-10	2.99E-09
908	ENSG00000114013.15	CD86	7.59	1.43E-10	3.13E-09
909	ENSG00000139625.12	MAP3K12	-1.01	1.48E-10	3.23E-09
910	ENSG00000111696.11	NT5DC3	1.07	1.56E-10	3.41E-09
911	ENSG00000261578.1	AP003119.3	1.14	1.63E-10	3.55E-09
912	ENSG00000196569.12	LAMA2	-0.80	1.68E-10	3.64E-09
913	ENSG00000266412.5	NCOA4	0.54	1.69E-10	3.67E-09
914	ENSG00000119684.15	MLH3	-0.81	1.70E-10	3.69E-09
915	ENSG00000280071.3	GATD3B	0.64	1.72E-10	3.74E-09
916	ENSG00000143537.13	ADAM15	-0.97	1.73E-10	3.75E-09
917	ENSG00000151893.14	CACUL1	0.63	1.76E-10	3.81E-09
918	ENSG00000113231.13	PDE8B	1.41	1.77E-10	3.81E-09
919	ENSG00000180616.8	SSTR2	1.30	1.78E-10	3.83E-09
920	ENSG00000158793.13	NIT1	0.80	1.78E-10	3.84E-09
921	ENSG00000137824.15	RMDN3	0.63	1.79E-10	3.85E-09
922	ENSG00000132716.18	DCAF8	0.54	1.79E-10	3.85E-09
923	ENSG00000149212.11	SESN3	-0.80	1.79E-10	3.85E-09
924	ENSG00000005108.15	THSD7A	1.77	1.83E-10	3.93E-09
925	ENSG00000135862.5	LAMC1	-0.63	1.83E-10	3.93E-09
926	ENSG00000026652.13	AGPAT4	0.87	1.85E-10	3.96E-09
927	ENSG00000148053.15	NTRK2	1.49	1.87E-10	4.00E-09
928	ENSG00000107960.10	STN1	0.83	1.91E-10	4.07E-09
929	ENSG00000256053.7	APOPT1	0.84	2.02E-10	4.31E-09
930	ENSG00000166033.12	HTRA1	-0.65	2.19E-10	4.67E-09
931	ENSG00000127220.5	ABHD8	1.25	2.21E-10	4.71E-09
932	ENSG00000114423.20	CBLB	-0.77	2.25E-10	4.79E-09
933	ENSG00000177409.11	SAMD9L	-0.77	2.27E-10	4.81E-09
934	ENSG00000159792.9	PSKH1	0.68	2.37E-10	5.03E-09
935	ENSG00000143067.4	ZNF697	0.86	2.54E-10	5.39E-09
936	ENSG00000124249.6	KCNK15	1.34	2.77E-10	5.87E-09
937	ENSG00000145214.13	DGKQ	0.91	2.79E-10	5.90E-09
938	ENSG00000125648.14	SLC25A23	-0.76	2.91E-10	6.16E-09
939	ENSG00000198130.15	HIBCH	0.90	2.95E-10	6.23E-09
940	ENSG00000115364.13	MRPL19	0.67	2.95E-10	6.23E-09
941	ENSG00000079102.16	RUNX1T1	-0.85	3.13E-10	6.60E-09
942	ENSG00000178761.14	FAM219B	0.66	3.16E-10	6.64E-09
943	ENSG00000103222.18	ABCC1	-0.70	3.27E-10	6.87E-09
944	ENSG00000106823.12	ECM2	-0.79	3.44E-10	7.22E-09
945	ENSG00000054793.13	ATP9A	0.64	3.45E-10	7.23E-09
946	ENSG00000089220.4	PEBP1	0.71	3.52E-10	7.37E-09
947	ENSG00000262179.2	MYMX	1.25	3.53E-10	7.39E-09

948	ENSG00000091542.8	ALKBH5	0.59	3.71E-10	7.77E-09
949	ENSG00000141293.15	SKAP1	2.97	3.73E-10	7.79E-09
950	ENSG00000172270.19	BSG	0.79	3.74E-10	7.81E-09
951	ENSG00000271880.1	AGAP11	1.59	3.80E-10	7.92E-09
952	ENSG00000115828.16	QPCT	1.32	3.82E-10	7.96E-09
953	ENSG00000066926.10	FECH	0.61	3.83E-10	7.97E-09
954	ENSG00000198363.17	ASPH	-0.53	3.87E-10	8.05E-09
955	ENSG00000074219.13	TEAD2	-0.71	3.97E-10	8.23E-09
956	ENSG00000053438.9	NNAT	1.39	3.97E-10	8.24E-09
957	ENSG00000167323.11	STIM1	0.60	4.02E-10	8.32E-09
958	ENSG00000121769.7	FABP3	4.07	4.06E-10	8.39E-09
959	ENSG00000134954.14	ETS1	-0.95	4.07E-10	8.41E-09
960	ENSG00000141385.9	AFG3L2	0.58	4.08E-10	8.43E-09
961	ENSG00000273079.5	GRIN2B	-1.19	4.17E-10	8.61E-09
962	ENSG00000127920.5	GNG11	-0.94	4.24E-10	8.75E-09
963	ENSG00000173124.14	ACSM6	2.60	4.29E-10	8.83E-09
964	ENSG00000008394.12	MGST1	0.55	4.42E-10	9.09E-09
965	ENSG00000226145.7	KRT16P6	2.30	4.62E-10	9.49E-09
966	ENSG00000189184.11	PCDH18	-0.74	4.86E-10	9.97E-09
967	ENSG00000172572.6	PDE3A	-1.04	5.01E-10	1.03E-08
968	ENSG00000135472.8	FAIM2	-2.16	5.02E-10	1.03E-08
969	ENSG00000241360.1	PDXP	0.84	5.28E-10	1.08E-08
970	ENSG00000164776.9	PHKG1	-1.87	5.56E-10	1.14E-08
971	ENSG00000248429.5	FAM198B-AS1	-1.40	5.63E-10	1.15E-08
972	ENSG00000122140.10	MRPS2	0.82	5.82E-10	1.19E-08
973	ENSG00000181826.9	RELL1	-1.57	5.99E-10	1.22E-08
974	ENSG00000177542.10	SLC25A22	1.15	6.06E-10	1.23E-08
975	ENSG00000131507.10	NDFIP1	-0.55	6.08E-10	1.24E-08
976	ENSG00000185920.15	PTCH1	-1.01	6.54E-10	1.33E-08
977	ENSG00000215769.8	AP27P1-BPTFP1-KPN	-1.08	6.55E-10	1.33E-08
978	ENSG00000080573.6	COL5A3	0.72	6.73E-10	1.36E-08
979	ENSG00000160200.17	CBS	-0.99	6.74E-10	1.37E-08
980	ENSG00000134333.13	LDHA	0.62	6.76E-10	1.37E-08
981	ENSG00000241399.6	CD302	0.69	6.94E-10	1.40E-08
982	ENSG00000237813.3	AC002066.1	1.57	7.06E-10	1.43E-08
983	ENSG00000246523.7	AP001528.1	1.23	7.26E-10	1.46E-08
984	ENSG00000186458.4	DEFB132	-0.99	7.55E-10	1.52E-08
985	ENSG00000213965.3	NUDT19	0.78	7.63E-10	1.54E-08
986	ENSG00000138134.11	STAMBPL1	1.15	7.76E-10	1.56E-08
987	ENSG00000224424.7	PRKAR2A-AS1	1.13	7.85E-10	1.58E-08
988	ENSG00000180483.6	DEFB119	7.90	8.09E-10	1.62E-08
989	ENSG00000160336.14	ZNF761	0.72	8.29E-10	1.66E-08
990	ENSG00000065833.8	ME1	0.68	8.46E-10	1.69E-08
991	ENSG00000176903.4	PNMA1	-0.72	8.48E-10	1.70E-08
992	ENSG00000043143.20	JADE2	-0.87	8.70E-10	1.74E-08
993	ENSG00000169926.10	KLF13	-0.84	8.74E-10	1.75E-08
994	ENSG00000160691.18	SHC1	-0.78	8.85E-10	1.77E-08
995	ENSG00000115317.11	HTRA2	0.85	9.28E-10	1.85E-08
996	ENSG00000132819.16	RBM38	0.95	9.90E-10	1.97E-08
997	ENSG00000111341.9	MGP	-0.85	9.91E-10	1.97E-08

998	ENSG00000136960.12	ENPP2	-0.62	9.93E-10	1.97E-08
999	ENSG00000145354.11	CISD2	0.83	9.95E-10	1.98E-08
1000	ENSG00000165507.8	DEPP1	-0.89	1.02E-09	2.03E-08
1001	ENSG00000121281.12	ADCY7	-1.21	1.03E-09	2.03E-08
1002	ENSG00000269837.1	IPO5P1	0.94	1.03E-09	2.04E-08
1003	ENSG00000117394.21	SLC2A1	-0.92	1.03E-09	2.04E-08
1004	ENSG00000155313.15	USP25	0.66	1.06E-09	2.10E-08
1005	ENSG00000083123.14	BCKDHB	0.80	1.08E-09	2.13E-08
1006	ENSG00000137801.10	THBS1	-0.64	1.09E-09	2.15E-08
1007	ENSG00000204228.3	HSD17B8	1.04	1.13E-09	2.22E-08
1008	ENSG00000154188.9	ANGPT1	-0.67	1.16E-09	2.29E-08
1009	ENSG00000261737.1	AL049597.2	1.30	1.18E-09	2.33E-08
1010	ENSG00000140406.3	TLNRD1	1.19	1.20E-09	2.35E-08
1011	ENSG00000097033.14	SH3GLB1	0.59	1.21E-09	2.38E-08
1012	ENSG00000240602.7	AADACP1	4.82	1.23E-09	2.40E-08
1013	ENSG00000278970.1	HEIH	0.80	1.23E-09	2.41E-08
1014	ENSG00000062582.13	MRPS24	0.70	1.23E-09	2.41E-08
1015	ENSG00000235263.1	AL928921.1	2.16	1.27E-09	2.48E-08
1016	ENSG00000113758.13	DBN1	-0.71	1.27E-09	2.48E-08
1017	ENSG00000079482.12	OPHN1	0.74	1.28E-09	2.48E-08
1018	ENSG00000147459.17	DOCK5	-0.79	1.27E-09	2.48E-08
1019	ENSG00000072786.12	STK10	0.90	1.30E-09	2.52E-08
1020	ENSG00000077514.8	POLD3	0.92	1.33E-09	2.58E-08
1021	ENSG00000149256.15	TENM4	0.79	1.39E-09	2.69E-08
1022	ENSG00000101236.16	RNF24	-0.65	1.39E-09	2.69E-08
1023	ENSG00000169122.11	FAM110B	-0.87	1.42E-09	2.76E-08
1024	ENSG00000135776.4	ABCB10	0.71	1.56E-09	3.02E-08
1025	ENSG00000169174.10	PCSK9	-0.97	1.58E-09	3.06E-08
1026	ENSG00000135185.11	TMEM243	0.79	1.59E-09	3.06E-08
1027	ENSG00000092377.13	TBL1Y	1.58	1.60E-09	3.09E-08
1028	ENSG00000180871.7	CXCR2	1.64	1.66E-09	3.20E-08
1029	ENSG00000162909.17	CAPN2	-0.70	1.67E-09	3.21E-08
1030	ENSG00000131711.14	MAP1B	-0.52	1.69E-09	3.25E-08
1031	ENSG00000234509.1	AP000253.1	2.43	1.73E-09	3.32E-08
1032	ENSG00000140391.14	TSPAN3	0.51	1.75E-09	3.36E-08
1033	ENSG00000168792.4	ABHD15	0.64	1.78E-09	3.43E-08
1034	ENSG00000143878.9	RHOB	0.53	1.85E-09	3.54E-08
1035	ENSG00000120333.4	MRPS14	0.72	1.87E-09	3.59E-08
1036	ENSG00000277443.2	MARCKS	-0.75	1.87E-09	3.59E-08
1037	ENSG00000008083.13	JARID2	0.76	1.90E-09	3.63E-08
1038	ENSG00000285043.1	AC093512.2	1.25	1.92E-09	3.66E-08
1039	ENSG00000011198.9	ABHD5	0.71	1.92E-09	3.66E-08
1040	ENSG00000173281.4	PPP1R3B	-0.82	1.95E-09	3.71E-08
1041	ENSG00000106554.12	CHCHD3	0.66	1.96E-09	3.73E-08
1042	ENSG00000181444.12	ZNF467	-2.12	1.99E-09	3.79E-08
1043	ENSG00000062485.18	CS	0.56	2.15E-09	4.08E-08
1044	ENSG00000128591.15	FLNC	-0.73	2.21E-09	4.19E-08
1045	ENSG00000141338.13	ABCA8	-1.03	2.21E-09	4.19E-08
1046	ENSG00000162576.16	MXRA8	-0.80	2.29E-09	4.35E-08
1047	ENSG00000100629.16	CEP128	-1.18	2.47E-09	4.67E-08

1048	ENSG00000124225.15	PMEPA1	0.94	2.51E-09	4.76E-08
1049	ENSG00000145780.7	FEM1C	0.63	2.52E-09	4.77E-08
1050	ENSG00000162772.16	ATF3	1.12	2.55E-09	4.82E-08
1051	ENSG00000156709.13	AIFM1	0.56	2.61E-09	4.93E-08
1052	ENSG00000233725.7	LINC00284	3.63	2.67E-09	5.03E-08
1053	ENSG00000267385.1	AC011498.4	1.28	2.67E-09	5.03E-08
1054	ENSG00000100077.14	GRK3	0.91	2.73E-09	5.14E-08
1055	ENSG00000128311.13	TST	0.99	2.77E-09	5.21E-08
1056	ENSG00000079819.18	EPB41L2	-0.60	2.82E-09	5.30E-08
1057	ENSG00000097007.17	ABL1	-0.66	2.92E-09	5.48E-08
1058	ENSG00000196935.8	SRGAP1	-0.80	3.00E-09	5.62E-08
1059	ENSG00000240303.7	ACAD11	0.70	3.02E-09	5.66E-08
1060	ENSG00000137944.17	KYAT3	0.89	3.11E-09	5.82E-08
1061	ENSG00000211455.7	STK38L	0.65	3.26E-09	6.09E-08
1062	ENSG00000124613.8	ZNF391	0.92	3.34E-09	6.24E-08
1063	ENSG00000163430.11	FSTL1	-0.54	3.36E-09	6.26E-08
1064	ENSG00000106392.10	C1GALT1	-0.70	3.38E-09	6.29E-08
1065	ENSG00000260398.1	AC068700.1	2.32	3.41E-09	6.35E-08
1066	ENSG00000170323.8	FABP4	2.24	3.42E-09	6.35E-08
1067	ENSG00000146674.14	IGFBP3	-0.83	3.41E-09	6.35E-08
1068	ENSG00000164442.9	CITED2	-0.71	3.44E-09	6.39E-08
1069	ENSG00000232233.1	LINC02043	2.01	3.48E-09	6.46E-08
1070	ENSG00000261159.1	AC112484.3	0.91	3.75E-09	6.94E-08
1071	ENSG00000106462.10	EZH2	1.12	3.78E-09	6.99E-08
1072	ENSG00000182534.13	MXRA7	-0.60	3.83E-09	7.08E-08
1073	ENSG00000092841.18	MYL6	0.53	3.91E-09	7.23E-08
1074	ENSG00000233695.2	GAS6-AS1	-1.93	3.97E-09	7.34E-08
1075	ENSG00000013503.9	POLR3B	0.79	4.02E-09	7.42E-08
1076	ENSG00000075945.12	KIFAP3	-0.69	4.04E-09	7.45E-08
1077	ENSG00000166949.15	SMAD3	-0.71	4.08E-09	7.50E-08
1078	ENSG00000146112.11	PPP1R18	-0.72	4.08E-09	7.50E-08
1079	ENSG00000179715.12	PCED1B	1.59	4.21E-09	7.74E-08
1080	ENSG00000188525.3	AC010969.1	5.87	4.22E-09	7.75E-08
1081	ENSG00000197852.10	INKA2	-1.15	4.24E-09	7.77E-08
1082	ENSG00000230630.5	DNM3OS	-0.97	4.28E-09	7.85E-08
1083	ENSG00000085998.13	POMGNT1	0.61	4.51E-09	8.26E-08
1084	ENSG00000120158.11	RCL1	0.81	4.63E-09	8.46E-08
1085	ENSG00000140939.14	NOL3	-0.86	4.68E-09	8.55E-08
1086	ENSG00000258545.5	RHOXF1-AS1	1.23	4.69E-09	8.55E-08
1087	ENSG00000107372.12	ZFAND5	-0.54	4.68E-09	8.55E-08
1088	ENSG00000079385.21	CEACAM1	2.04	4.76E-09	8.69E-08
1089	ENSG00000113361.12	CDH6	-0.88	4.99E-09	9.09E-08
1090	ENSG00000162746.14	FCRLB	1.03	5.08E-09	9.25E-08
1091	ENSG00000156011.16	PSD3	0.61	5.09E-09	9.26E-08
1092	ENSG00000107719.8	PALD1	1.95	5.10E-09	9.27E-08
1093	ENSG00000002586.19	CD99	-0.67	5.12E-09	9.28E-08
1094	ENSG00000138303.17	ASCC1	0.65	5.24E-09	9.48E-08
1095	ENSG00000010256.10	UQCRC1	0.56	5.47E-09	9.89E-08
1096	ENSG00000087460.24	GNAS	0.52	5.48E-09	9.91E-08
1097	ENSG00000010319.6	SEMA3G	0.53	5.63E-09	1.02E-07

1098	ENSG00000284876.1	AC015908.6	4.64	5.74E-09	1.04E-07
1099	ENSG00000167862.9	MRPL58	0.71	5.76E-09	1.04E-07
1100	ENSG00000159423.16	ALDH4A1	0.80	5.81E-09	1.05E-07
1101	ENSG00000113083.13	LOX	-0.93	6.00E-09	1.08E-07
1102	ENSG00000133657.15	ATP13A3	0.54	6.01E-09	1.08E-07
1103	ENSG00000185189.17	NRBP2	-0.72	6.01E-09	1.08E-07
1104	ENSG00000147400.8	CETN2	0.59	6.03E-09	1.08E-07
1105	ENSG00000101182.14	PSMA7	0.63	6.07E-09	1.09E-07
1106	ENSG00000118276.11	B4GALT6	0.72	6.08E-09	1.09E-07
1107	ENSG00000132854.18	KANK4	-1.81	6.10E-09	1.09E-07
1108	ENSG00000026103.21	FAS	-0.85	6.21E-09	1.11E-07
1109	ENSG00000196549.10	MME	0.53	6.24E-09	1.11E-07
1110	ENSG00000184451.5	CCR10	-2.92	6.34E-09	1.13E-07
1111	ENSG00000001630.16	CYP51A1	0.53	6.38E-09	1.14E-07
1112	ENSG00000166170.9	BAG5	0.61	6.43E-09	1.15E-07
1113	ENSG00000166411.13	IDH3A	0.65	6.52E-09	1.16E-07
1114	ENSG00000275025.1	AC002401.3	2.44	6.69E-09	1.19E-07
1115	ENSG00000177565.16	TBL1XR1	-0.57	6.69E-09	1.19E-07
1116	ENSG00000174705.12	SH3PXD2B	-0.75	6.74E-09	1.20E-07
1117	ENSG00000124253.10	PCK1	2.69	6.76E-09	1.20E-07
1118	ENSG00000162804.13	SNED1	-0.88	6.81E-09	1.21E-07
1119	ENSG00000198053.11	SIRPA	-0.62	7.02E-09	1.24E-07
1120	ENSG00000170004.16	CHD3	-0.60	7.18E-09	1.27E-07
1121	ENSG00000285898.1	AC007317.1	1.77	7.26E-09	1.28E-07
1122	ENSG00000152133.14	GPATCH11	0.81	7.31E-09	1.29E-07
1123	ENSG00000134955.11	SLC37A2	1.83	7.42E-09	1.31E-07
1124	ENSG00000159399.9	HK2	-0.57	7.44E-09	1.31E-07
1125	ENSG00000170276.5	HSPB2	-1.34	7.51E-09	1.32E-07
1126	ENSG00000156920.10	ADGRG4	2.74	7.69E-09	1.35E-07
1127	ENSG00000173597.8	SULT1B1	1.15	7.74E-09	1.36E-07
1128	ENSG00000109016.17	DHRS7B	0.59	7.77E-09	1.37E-07
1129	ENSG00000233608.3	TWIST2	0.83	7.93E-09	1.39E-07
1130	ENSG00000235875.3	ARHGEF7-AS2	2.86	8.37E-09	1.47E-07
1131	ENSG00000118729.11	CASQ2	1.09	8.38E-09	1.47E-07
1132	ENSG00000138495.6	COX17	0.70	8.51E-09	1.49E-07
1133	ENSG00000151093.7	OXSM	0.78	8.63E-09	1.51E-07
1134	ENSG00000173372.16	C1QA	3.84	8.71E-09	1.52E-07
1135	ENSG00000216490.3	IFI30	1.92	8.85E-09	1.55E-07
1136	ENSG00000146197.8	SCUBE3	-0.92	8.95E-09	1.56E-07
1137	ENSG00000183828.14	NUDT14	1.00	9.01E-09	1.57E-07
1138	ENSG00000152818.18	UTRN	0.57	9.02E-09	1.57E-07
1139	ENSG00000105514.7	RAB3D	-0.97	9.03E-09	1.57E-07
1140	ENSG00000100320.22	RBFOX2	-0.60	9.13E-09	1.59E-07
1141	ENSG00000147853.16	AK3	0.52	9.15E-09	1.59E-07
1142	ENSG00000147676.13	MAL2	-1.73	9.35E-09	1.62E-07
1143	ENSG00000171033.12	PKIA	0.77	9.38E-09	1.63E-07
1144	ENSG00000146373.16	RNF217	-0.62	9.60E-09	1.66E-07
1145	ENSG00000129691.15	ASH2L	0.70	9.61E-09	1.66E-07
1146	ENSG00000102755.11	FLT1	-1.11	9.87E-09	1.71E-07
1147	ENSG00000126214.21	KLC1	-0.57	9.91E-09	1.71E-07

1148	ENSG00000134531.9	EMP1	-0.98	1.02E-08	1.76E-07
1149	ENSG00000112695.11	COX7A2	0.54	1.04E-08	1.80E-07
1150	ENSG00000283154.2	IQCJ-SCHIP1	-0.87	1.04E-08	1.80E-07
1151	ENSG00000113369.8	ARRDC3	-0.74	1.06E-08	1.83E-07
1152	ENSG00000179918.18	SEPHS2	0.50	1.07E-08	1.85E-07
1153	ENSG00000090776.5	EFNB1	0.76	1.09E-08	1.87E-07
1154	ENSG00000103356.15	EARS2	0.65	1.12E-08	1.92E-07
1155	ENSG00000134240.11	HMGCS2	4.10	1.13E-08	1.94E-07
1156	ENSG00000255508.7	AP002990.1	1.13	1.13E-08	1.94E-07
1157	ENSG00000186470.13	BTN3A2	0.59	1.13E-08	1.94E-07
1158	ENSG00000140836.16	ZFHX3	-0.64	1.15E-08	1.96E-07
1159	ENSG00000118523.5	CTGF	-0.61	1.16E-08	1.98E-07
1160	ENSG00000090013.10	BLVRB	0.76	1.16E-08	1.98E-07
1161	ENSG00000065809.13	FAM107B	-0.73	1.19E-08	2.03E-07
1162	ENSG00000072518.20	MARK2	0.65	1.19E-08	2.04E-07
1163	ENSG00000166473.17	PKD1L2	-2.42	1.21E-08	2.05E-07
1164	ENSG00000135900.3	MRPL44	0.58	1.21E-08	2.05E-07
1165	ENSG00000279605.1	AC067930.6	2.84	1.23E-08	2.10E-07
1166	ENSG00000137814.10	HAUS2	0.78	1.25E-08	2.12E-07
1167	ENSG00000138074.14	SLC5A6	0.75	1.27E-08	2.15E-07
1168	ENSG00000250282.1	AC002401.2	2.83	1.27E-08	2.16E-07
1169	ENSG00000168619.15	ADAM18	3.87	1.29E-08	2.18E-07
1170	ENSG00000174173.6	TRMT10C	0.61	1.29E-08	2.19E-07
1171	ENSG00000106771.12	TMEM245	0.49	1.29E-08	2.19E-07
1172	ENSG00000033627.16	ATP6V0A1	0.58	1.31E-08	2.21E-07
1173	ENSG00000100697.14	DICER1	0.66	1.33E-08	2.24E-07
1174	ENSG00000206538.8	VGLL3	-0.59	1.36E-08	2.30E-07
1175	ENSG00000183087.14	GAS6	-0.89	1.37E-08	2.31E-07
1176	ENSG00000122642.10	FKBP9	-0.55	1.38E-08	2.32E-07
1177	ENSG00000151366.12	NDUFC2	0.54	1.38E-08	2.32E-07
1178	ENSG00000182179.12	UBA7	0.73	1.41E-08	2.37E-07
1179	ENSG00000215845.10	TSTD1	1.07	1.47E-08	2.47E-07
1180	ENSG00000181467.4	RAP2B	-0.72	1.48E-08	2.48E-07
1181	ENSG00000106610.15	STAG3L4	0.92	1.48E-08	2.49E-07
1182	ENSG00000108950.11	FAM20A	-1.13	1.50E-08	2.51E-07
1183	ENSG00000136859.9	ANGPTL2	0.56	1.57E-08	2.64E-07
1184	ENSG00000130150.11	MOSPD2	0.73	1.59E-08	2.65E-07
1185	ENSG00000103653.16	CSK	0.71	1.61E-08	2.69E-07
1186	ENSG00000114098.17	ARMC8	0.64	1.67E-08	2.79E-07
1187	ENSG00000171954.12	CYP4F22	-2.65	1.69E-08	2.83E-07
1188	ENSG00000206527.9	HACD2	0.74	1.72E-08	2.88E-07
1189	ENSG00000175220.11	ARHGAP1	-0.63	1.73E-08	2.88E-07
1190	ENSG00000116141.15	MARK1	2.34	1.73E-08	2.89E-07
1191	ENSG00000117410.13	ATP6V0B	0.62	1.75E-08	2.91E-07
1192	ENSG00000226268.3	AC135977.1	1.23	1.76E-08	2.92E-07
1193	ENSG00000228522.2	AL845321.1	1.85	1.80E-08	2.99E-07
1194	ENSG00000089159.16	PXN	-0.65	1.81E-08	3.00E-07
1195	ENSG00000134548.10	SPX	-2.24	1.83E-08	3.03E-07
1196	ENSG00000171206.14	TRIM8	-0.62	1.87E-08	3.09E-07
1197	ENSG00000153944.10	MSI2	-0.67	1.87E-08	3.10E-07

1198	ENSG00000116977.18	LGALS8	0.62	1.88E-08	3.11E-07
1199	ENSG00000150347.15	ARID5B	-0.55	1.92E-08	3.17E-07
1200	ENSG00000177200.17	CHD9	-0.54	1.93E-08	3.20E-07
1201	ENSG00000259037.1	BX927359.1	1.23	1.98E-08	3.26E-07
1202	ENSG00000107738.19	VSIR	0.94	2.00E-08	3.30E-07
1203	ENSG00000269473.1	AC012313.8	1.56	2.05E-08	3.37E-07
1204	ENSG00000166888.11	STAT6	-0.62	2.05E-08	3.38E-07
1205	ENSG00000125812.15	GZF1	0.65	2.10E-08	3.46E-07
1206	ENSG00000204842.15	ATXN2	0.57	2.12E-08	3.49E-07
1207	ENSG00000077254.14	USP33	0.61	2.13E-08	3.49E-07
1208	ENSG00000165821.11	SALL2	-1.76	2.18E-08	3.58E-07
1209	ENSG00000006530.16	AGK	0.65	2.19E-08	3.58E-07
1210	ENSG00000157514.16	TSC22D3	-0.75	2.22E-08	3.63E-07
1211	ENSG00000161243.8	FBXO27	1.01	2.23E-08	3.65E-07
1212	ENSG00000184470.20	TXNRD2	0.82	2.23E-08	3.65E-07
1213	ENSG00000268043.7	NBPF12	0.60	2.26E-08	3.70E-07
1214	ENSG00000135454.13	B4GALNT1	-1.55	2.27E-08	3.71E-07
1215	ENSG00000144712.12	CAND2	1.70	2.38E-08	3.89E-07
1216	ENSG00000185033.14	SEMA4B	-1.07	2.39E-08	3.89E-07
1217	ENSG00000188917.14	TRMT2B	0.80	2.42E-08	3.94E-07
1218	ENSG00000272078.1	AL139423.1	3.29	2.42E-08	3.94E-07
1219	ENSG00000230590.9	FTX	-0.56	2.47E-08	4.02E-07
1220	ENSG00000151693.10	ASAP2	-0.76	2.51E-08	4.08E-07
1221	ENSG00000135111.15	TBX3	0.65	2.52E-08	4.10E-07
1222	ENSG00000197043.13	ANXA6	0.47	2.52E-08	4.10E-07
1223	ENSG00000114019.14	AMOTL2	-0.59	2.57E-08	4.16E-07
1224	ENSG00000158747.14	NBL1	-0.94	2.60E-08	4.21E-07
1225	ENSG00000255471.1	AP001528.2	1.03	2.74E-08	4.44E-07
1226	ENSG00000127989.13	MTERF1	0.70	2.75E-08	4.45E-07
1227	ENSG00000001084.12	GCLC	0.62	2.87E-08	4.64E-07
1228	ENSG00000119242.8	CCDC92	-0.58	2.95E-08	4.76E-07
1229	ENSG00000140285.9	FGF7	-0.81	2.97E-08	4.80E-07
1230	ENSG00000131771.13	PPP1R1B	0.71	3.05E-08	4.91E-07
1231	ENSG00000183617.4	MRPL54	0.80	3.09E-08	4.98E-07
1232	ENSG00000129988.5	LBP	3.48	3.10E-08	4.99E-07
1233	ENSG00000137145.20	DENND4C	0.58	3.14E-08	5.05E-07
1234	ENSG00000142871.16	CYR61	-0.69	3.15E-08	5.06E-07
1235	ENSG00000278891.1	AC105101.1	2.10	3.19E-08	5.12E-07
1236	ENSG00000132669.13	RIN2	-0.69	3.20E-08	5.13E-07
1237	ENSG00000163069.12	SGCB	-0.73	3.20E-08	5.13E-07
1238	ENSG00000198732.10	SMOC1	1.22	3.21E-08	5.14E-07
1239	ENSG00000121895.7	TMEM156	1.91	3.24E-08	5.19E-07
1240	ENSG00000118515.11	SGK1	-0.79	3.26E-08	5.22E-07
1241	ENSG00000110074.10	FOXRED1	0.61	3.31E-08	5.28E-07
1242	ENSG00000100890.15	KIAA0391	0.57	3.31E-08	5.28E-07
1243	ENSG00000167460.15	TPM4	-0.54	3.33E-08	5.31E-07
1244	ENSG00000100906.10	NFKBIA	-0.75	3.34E-08	5.32E-07
1245	ENSG00000008513.15	ST3GAL1	0.52	3.38E-08	5.39E-07
1246	ENSG00000095370.19	SH2D3C	1.41	3.39E-08	5.39E-07
1247	ENSG00000237689.1	AC007064.2	2.77	3.41E-08	5.42E-07

1248	ENSG00000124343.13	XG	0.83	3.42E-08	5.44E-07
1249	ENSG00000147872.9	PLIN2	0.64	3.53E-08	5.59E-07
1250	ENSG00000048740.18	CELF2	-0.75	3.54E-08	5.62E-07
1251	ENSG00000259416.2	AC021739.3	2.20	3.62E-08	5.73E-07
1252	ENSG00000118407.14	FILIP1	-1.34	3.65E-08	5.77E-07
1253	ENSG00000171903.16	CYP4F11	-2.00	3.72E-08	5.88E-07
1254	ENSG00000106853.19	PTGR1	0.67	3.85E-08	6.08E-07
1255	ENSG00000125445.10	MRPS7	0.54	3.88E-08	6.13E-07
1256	ENSG00000182021.10	AL591379.1	1.93	3.88E-08	6.13E-07
1257	ENSG00000088836.13	SLC4A11	-1.97	3.90E-08	6.15E-07
1258	ENSG00000109084.13	TMEM97	0.72	3.91E-08	6.17E-07
1259	ENSG00000166762.17	CATSPER2	1.11	3.97E-08	6.25E-07
1260	ENSG00000260192.2	LINC02240	1.84	3.98E-08	6.26E-07
1261	ENSG00000137869.14	CYP19A1	-2.05	3.98E-08	6.26E-07
1262	ENSG00000178741.11	COX5A	0.59	4.01E-08	6.29E-07
1263	ENSG00000267855.5	NDUFA7	0.66	4.08E-08	6.41E-07
1264	ENSG00000135617.3	PRADC1	0.61	4.09E-08	6.42E-07
1265	ENSG00000145715.14	RASA1	-0.54	4.13E-08	6.47E-07
1266	ENSG00000101098.12	RIMS4	0.97	4.17E-08	6.53E-07
1267	ENSG00000238018.2	AC093110.1	1.21	4.21E-08	6.59E-07
1268	ENSG00000169131.12	ZNF354A	0.74	4.24E-08	6.62E-07
1269	ENSG00000164284.14	GRPEL2	0.60	4.24E-08	6.62E-07
1270	ENSG00000197448.13	GSTK1	0.49	4.27E-08	6.67E-07
1271	ENSG00000175768.12	TOMM5	0.67	4.30E-08	6.70E-07
1272	ENSG00000277203.1	F8A1	0.56	4.30E-08	6.70E-07
1273	ENSG00000160712.12	IL6R	-1.48	4.41E-08	6.87E-07
1274	ENSG00000112079.8	STK38	0.60	4.43E-08	6.89E-07
1275	ENSG00000167080.8	B4GALNT2	0.85	4.46E-08	6.93E-07
1276	ENSG00000204568.11	MRPS18B	0.55	4.46E-08	6.94E-07
1277	ENSG00000111885.6	MAN1A1	-0.54	4.47E-08	6.94E-07
1278	ENSG00000116793.15	PHTF1	0.59	4.61E-08	7.16E-07
1279	ENSG00000083857.13	FAT1	-0.50	4.74E-08	7.35E-07
1280	ENSG00000166896.8	ATP23	0.78	4.77E-08	7.39E-07
1281	ENSG00000154518.9	ATP5MC3	0.48	4.81E-08	7.44E-07
1282	ENSG00000196233.13	LCOR	0.69	4.81E-08	7.44E-07
1283	ENSG00000166391.14	MOGAT2	1.18	4.83E-08	7.46E-07
1284	ENSG00000075426.11	FOSL2	-0.58	4.85E-08	7.48E-07
1285	ENSG00000104823.8	ECH1	0.76	4.89E-08	7.55E-07
1286	ENSG00000269955.2	C7orf55-LUC7L2	0.80	4.95E-08	7.63E-07
1287	ENSG00000133246.11	PRAM1	2.15	5.01E-08	7.71E-07
1288	ENSG00000153310.19	FAM49B	0.81	5.06E-08	7.79E-07
1289	ENSG00000170906.15	NDUFA3	0.69	5.28E-08	8.12E-07
1290	ENSG00000179604.9	CDC42EP4	0.66	5.31E-08	8.15E-07
1291	ENSG00000141012.12	GALNS	-0.76	5.30E-08	8.15E-07
1292	ENSG00000166025.17	AMOTL1	0.58	5.50E-08	8.43E-07
1293	ENSG00000122870.11	BICC1	-0.78	5.51E-08	8.44E-07
1294	ENSG00000164056.10	SPRY1	0.70	5.52E-08	8.46E-07
1295	ENSG00000143256.4	PFDN2	0.69	5.53E-08	8.47E-07
1296	ENSG00000125398.6	SOX9	0.60	5.63E-08	8.60E-07
1297	ENSG00000196139.13	AKR1C3	-0.65	5.63E-08	8.60E-07

1298	ENSG00000172432.18	GTPBP2	0.51	5.79E-08	8.84E-07
1299	ENSG00000227051.6	C14orf132	-0.66	5.87E-08	8.96E-07
1300	ENSG00000166619.13	BLCAP	0.53	6.00E-08	9.15E-07
1301	ENSG00000135678.11	CPM	-0.78	6.08E-08	9.26E-07
1302	ENSG00000071967.11	CYBRD1	-0.79	6.14E-08	9.36E-07
1303	ENSG00000124942.13	AHNAK	-0.67	6.16E-08	9.37E-07
1304	ENSG00000187243.16	MAGED4B	1.12	6.22E-08	9.46E-07
1305	ENSG00000261200.1	AC136944.2	1.33	6.28E-08	9.54E-07
1306	ENSG00000125266.7	EFNB2	1.51	6.42E-08	9.75E-07
1307	ENSG00000104043.14	ATP8B4	-0.89	6.42E-08	9.75E-07
1308	ENSG00000203857.9	HSD3B1	4.81	6.43E-08	9.75E-07
1309	ENSG00000165983.14	PTER	0.66	6.46E-08	9.78E-07
1310	ENSG00000167085.11	PHB	0.55	6.53E-08	9.88E-07
1311	ENSG00000134324.11	LPIN1	-0.62	6.58E-08	9.96E-07
1312	ENSG00000157216.15	SSBP3	-0.62	6.72E-08	1.01E-06
1313	ENSG00000142687.17	KIAA0319L	0.52	6.78E-08	1.02E-06
1314	ENSG00000170876.7	TMEM43	-0.58	6.87E-08	1.04E-06
1315	ENSG00000156052.10	GNAQ	-0.47	6.93E-08	1.04E-06
1316	ENSG00000137942.16	FNBP1L	-0.64	6.94E-08	1.05E-06
1317	ENSG00000100731.15	PCNX1	0.59	6.95E-08	1.05E-06
1318	ENSG00000147955.16	SIGMAR1	0.53	6.97E-08	1.05E-06
1319	ENSG00000184743.12	ATL3	-0.51	7.01E-08	1.05E-06
1320	ENSG00000131080.14	EDA2R	-0.80	7.10E-08	1.07E-06
1321	ENSG00000142655.12	PEX14	0.78	7.15E-08	1.07E-06
1322	ENSG00000186106.11	ANKRD46	0.65	7.23E-08	1.08E-06
1323	ENSG00000130193.7	THEM6	0.79	7.28E-08	1.09E-06
1324	ENSG00000147642.16	SYBU	-0.88	7.33E-08	1.10E-06
1325	ENSG00000074590.13	NUAK1	-0.66	7.39E-08	1.11E-06
1326	ENSG00000204681.10	GABBR1	-0.79	7.40E-08	1.11E-06
1327	ENSG00000164603.11	BMT2	-0.69	7.44E-08	1.11E-06
1328	ENSG00000166682.11	TMPRSS5	1.52	7.45E-08	1.11E-06
1329	ENSG00000175482.8	POLD4	-0.92	7.45E-08	1.11E-06
1330	ENSG00000145685.13	LHFPL2	0.57	7.49E-08	1.12E-06
1331	ENSG00000251257.2	AC010457.1	3.25	7.76E-08	1.15E-06
1332	ENSG00000142089.15	IFITM3	-0.71	7.75E-08	1.15E-06
1333	ENSG00000168874.12	ATOH8	0.99	7.77E-08	1.16E-06
1334	ENSG00000171574.17	ZNF584	0.77	8.08E-08	1.20E-06
1335	ENSG00000101391.20	CDK5RAP1	0.61	8.11E-08	1.20E-06
1336	ENSG00000154274.14	C4orf19	1.15	8.15E-08	1.21E-06
1337	ENSG00000089356.18	FXYD3	2.29	8.34E-08	1.24E-06
1338	ENSG00000163738.18	MTHFD2L	0.91	8.41E-08	1.25E-06
1339	ENSG00000120437.8	ACAT2	0.56	8.41E-08	1.25E-06
1340	ENSG00000167969.12	ECI1	0.78	8.62E-08	1.28E-06
1341	ENSG00000273729.1	AC007686.3	1.05	8.65E-08	1.28E-06
1342	ENSG00000285108.1	AC103718.1	-2.67	8.67E-08	1.28E-06
1343	ENSG00000082781.11	ITGB5	-0.62	8.87E-08	1.31E-06
1344	ENSG00000137941.16	TTLL7	0.55	9.06E-08	1.34E-06
1345	ENSG00000107897.18	ACBD5	0.63	9.15E-08	1.35E-06
1346	ENSG00000084734.8	GCKR	1.77	9.20E-08	1.35E-06
1347	ENSG00000136153.19	LMO7	-0.68	9.25E-08	1.36E-06

1348	ENSG00000128274.16	A4GALT	-1.23	9.25E-08	1.36E-06
1349	ENSG00000113441.15	LNPEP	0.63	9.28E-08	1.36E-06
1350	ENSG00000110536.13	PTPMT1	0.67	9.46E-08	1.39E-06
1351	ENSG00000105926.15	MPP6	0.72	9.54E-08	1.40E-06
1352	ENSG00000110651.11	CD81	-0.56	9.56E-08	1.40E-06
1353	ENSG00000179862.6	CITED4	1.42	9.58E-08	1.40E-06
1354	ENSG00000040531.14	CTNS	0.62	9.58E-08	1.40E-06
1355	ENSG00000127824.13	TUBA4A	-1.02	9.66E-08	1.41E-06
1356	ENSG00000213190.3	MLLT11	-0.87	9.85E-08	1.44E-06
1357	ENSG00000229619.3	MBNL1-AS1	-0.77	9.89E-08	1.44E-06
1358	ENSG00000120992.17	LYPLA1	0.71	1.00E-07	1.47E-06
1359	ENSG00000115459.17	ELMOD3	0.58	1.02E-07	1.48E-06
1360	ENSG00000153956.15	CACNA2D1	-0.49	1.03E-07	1.50E-06
1361	ENSG00000138621.11	PPCDC	0.83	1.04E-07	1.52E-06
1362	ENSG00000183111.11	ARHGEF37	-1.20	1.04E-07	1.52E-06
1363	ENSG00000172366.19	MCRIP2	0.84	1.06E-07	1.54E-06
1364	ENSG00000080503.23	SMARCA2	-0.51	1.06E-07	1.54E-06
1365	ENSG00000103423.13	DNAJA3	0.53	1.09E-07	1.58E-06
1366	ENSG00000167613.15	LAIR1	0.70	1.10E-07	1.59E-06
1367	ENSG00000239704.10	CDRT4	-1.77	1.10E-07	1.59E-06
1368	ENSG00000172340.14	SUCLG2	0.48	1.11E-07	1.61E-06
1369	ENSG00000186716.20	BCR	0.62	1.12E-07	1.63E-06
1370	ENSG00000214708.4	AC116407.1	2.22	1.13E-07	1.63E-06
1371	ENSG00000196236.12	XPNPEP3	0.56	1.13E-07	1.64E-06
1372	ENSG00000270419.1	CAHM	1.87	1.13E-07	1.64E-06
1373	ENSG00000084693.15	AGBL5	0.66	1.14E-07	1.65E-06
1374	ENSG00000145743.15	FBXL17	0.52	1.15E-07	1.66E-06
1375	ENSG00000073921.17	PICALM	0.43	1.15E-07	1.66E-06
1376	ENSG00000251169.2	LINC01843	1.37	1.17E-07	1.69E-06
1377	ENSG00000075624.14	ACTB	-0.53	1.19E-07	1.71E-06
1378	ENSG00000100380.13	ST13	0.49	1.20E-07	1.72E-06
1379	ENSG00000135596.17	MICAL1	-0.81	1.20E-07	1.73E-06
1380	ENSG00000067064.11	IDI1	0.53	1.22E-07	1.75E-06
1381	ENSG00000099194.5	SCD	0.45	1.24E-07	1.79E-06
1382	ENSG00000170522.9	ELOVL6	0.90	1.25E-07	1.80E-06
1383	ENSG00000174099.11	MSRB3	-0.48	1.26E-07	1.80E-06
1384	ENSG00000138685.15	FGF2	-0.64	1.26E-07	1.80E-06
1385	ENSG00000182544.8	MFSD5	0.71	1.29E-07	1.84E-06
1386	ENSG00000186522.14	SEPT10	0.52	1.29E-07	1.84E-06
1387	ENSG00000248358.2	AC243972.1	3.05	1.29E-07	1.85E-06
1388	ENSG00000127184.12	COX7C	0.45	1.29E-07	1.85E-06
1389	ENSG00000144320.13	LNPK	0.56	1.32E-07	1.89E-06
1390	ENSG00000185507.20	IRF7	1.21	1.32E-07	1.89E-06
1391	ENSG00000010310.8	GIPR	0.77	1.34E-07	1.91E-06
1392	ENSG00000133318.13	RTN3	0.53	1.35E-07	1.92E-06
1393	ENSG00000129757.13	CDKN1C	-1.35	1.37E-07	1.95E-06
1394	ENSG00000166582.9	CENPV	0.94	1.38E-07	1.97E-06
1395	ENSG00000086061.15	DNAJA1	0.67	1.46E-07	2.08E-06
1396	ENSG00000138756.17	BMP2K	-0.64	1.49E-07	2.12E-06
1397	ENSG00000023228.13	NDUFS1	0.50	1.52E-07	2.15E-06

1398	ENSG00000108176.14	DNAJC12	-1.77	1.52E-07	2.15E-06
1399	ENSG00000076351.12	SLC46A1	0.70	1.52E-07	2.16E-06
1400	ENSG00000121753.12	ADGRB2	1.03	1.55E-07	2.19E-06
1401	ENSG00000179627.9	ZBTB42	0.99	1.54E-07	2.19E-06
1402	ENSG00000100979.14	PLTP	1.72	1.55E-07	2.19E-06
1403	ENSG00000257303.1	AC073896.2	1.16	1.56E-07	2.20E-06
1404	ENSG00000235434.1	AL391883.1	2.80	1.56E-07	2.21E-06
1405	ENSG00000010810.17	FYN	-0.74	1.57E-07	2.22E-06
1406	ENSG00000137936.17	BCAR3	0.52	1.58E-07	2.22E-06
1407	ENSG00000129353.14	SLC44A2	-0.64	1.58E-07	2.22E-06
1408	ENSG00000143995.19	MEIS1	-0.62	1.58E-07	2.23E-06
1409	ENSG00000197872.11	FAM49A	-1.01	1.59E-07	2.24E-06
1410	ENSG00000101605.12	MYOM1	1.15	1.60E-07	2.25E-06
1411	ENSG00000185608.8	MRPL40	0.56	1.61E-07	2.26E-06
1412	ENSG00000243056.1	EIF4EBP3	1.50	1.63E-07	2.29E-06
1413	ENSG00000135404.11	CD63	0.51	1.64E-07	2.30E-06
1414	ENSG00000147100.10	SLC16A2	-0.60	1.66E-07	2.33E-06
1415	ENSG00000198169.8	ZNF251	-0.71	1.66E-07	2.33E-06
1416	ENSG00000123297.17	TSFM	0.63	1.67E-07	2.34E-06
1417	ENSG00000138650.8	PCDH10	-0.81	1.69E-07	2.36E-06
1418	ENSG00000169213.6	RAB3B	-0.70	1.69E-07	2.36E-06
1419	ENSG00000109339.21	MAPK10	0.68	1.72E-07	2.40E-06
1420	ENSG00000121297.6	TSHZ3	-0.83	1.73E-07	2.42E-06
1421	ENSG00000114541.14	FRMD4B	1.89	1.76E-07	2.46E-06
1422	ENSG00000173517.10	PEAK1	-0.46	1.80E-07	2.51E-06
1423	ENSG00000123342.15	MMP19	0.61	1.85E-07	2.58E-06
1424	ENSG00000120708.16	TGFBI	-0.70	1.86E-07	2.59E-06
1425	ENSG00000119689.14	DLST	0.46	1.90E-07	2.64E-06
1426	ENSG00000149541.9	B3GAT3	0.75	1.92E-07	2.67E-06
1427	ENSG00000137054.15	POLR1E	0.64	1.92E-07	2.67E-06
1428	ENSG00000131095.12	GFAP	1.28	1.96E-07	2.73E-06
1429	ENSG00000163931.15	TKT	0.56	1.97E-07	2.74E-06
1430	ENSG00000164938.13	TP53INP1	-0.53	2.01E-07	2.78E-06
1431	ENSG00000116106.11	EPHA4	-0.83	2.02E-07	2.80E-06
1432	ENSG00000204577.11	LILRB3	2.24	2.05E-07	2.84E-06
1433	ENSG00000150630.3	VEGFC	-0.88	2.09E-07	2.89E-06
1434	ENSG00000164761.8	TNFRSF11B	-0.99	2.09E-07	2.90E-06
1435	ENSG00000159167.11	STC1	-1.28	2.20E-07	3.04E-06
1436	ENSG00000231982.1	AC112907.2	2.74	2.28E-07	3.15E-06
1437	ENSG00000112293.14	GPLD1	1.48	2.29E-07	3.17E-06
1438	ENSG00000134262.12	AP4B1	-0.58	2.31E-07	3.19E-06
1439	ENSG00000106052.13	TAX1BP1	-0.52	2.33E-07	3.22E-06
1440	ENSG00000037042.8	TUBG2	-0.69	2.34E-07	3.22E-06
1441	ENSG00000073578.16	SDHA	0.49	2.38E-07	3.28E-06
1442	ENSG00000162627.16	SNX7	-0.81	2.41E-07	3.31E-06
1443	ENSG00000180530.10	NRIP1	0.76	2.44E-07	3.35E-06
1444	ENSG00000071242.11	RPS6KA2	-0.54	2.52E-07	3.46E-06
1445	ENSG00000182199.10	SHMT2	0.57	2.53E-07	3.48E-06
1446	ENSG00000229847.8	EMX2OS	-0.55	2.55E-07	3.49E-06
1447	ENSG00000116260.16	QSOX1	-0.76	2.55E-07	3.49E-06

1448	ENSG00000127838.13	PNKD	0.65	2.62E-07	3.58E-06
1449	ENSG00000171388.11	APLN	-1.37	2.67E-07	3.66E-06
1450	ENSG00000164176.12	EDIL3	-0.64	2.69E-07	3.68E-06
1451	ENSG00000136603.13	SKIL	-0.57	2.70E-07	3.68E-06
1452	ENSG00000204822.6	MRPL53	0.67	2.71E-07	3.70E-06
1453	ENSG00000099800.7	TIMM13	0.65	2.74E-07	3.73E-06
1454	ENSG00000249087.6	ZNF436-AS1	0.76	2.78E-07	3.79E-06
1455	ENSG00000182606.14	TRAK1	-0.52	2.79E-07	3.80E-06
1456	ENSG00000184232.8	OAF	0.60	2.81E-07	3.82E-06
1457	ENSG00000108561.8	C1QBP	0.53	2.81E-07	3.83E-06
1458	ENSG00000075151.20	EIF4G3	-0.53	2.82E-07	3.83E-06
1459	ENSG00000256040.2	PAPPA-AS1	-1.53	2.81E-07	3.83E-06
1460	ENSG00000145979.17	TBC1D7	0.70	2.83E-07	3.84E-06
1461	ENSG00000120738.7	EGR1	-1.08	2.85E-07	3.87E-06
1462	ENSG00000196917.5	HCAR1	0.93	2.86E-07	3.88E-06
1463	ENSG00000165246.14	NLGN4Y	-0.70	2.86E-07	3.88E-06
1464	ENSG00000100106.20	TRIOBP	-0.72	2.86E-07	3.88E-06
1465	ENSG00000119655.10	NPC2	0.62	2.90E-07	3.92E-06
1466	ENSG00000237118.3	CYP2F2P	2.83	2.92E-07	3.95E-06
1467	ENSG00000112183.14	RBM24	0.84	2.93E-07	3.96E-06
1468	ENSG00000116711.9	PLA2G4A	-0.60	2.98E-07	4.02E-06
1469	ENSG00000186104.10	CYP2R1	0.77	3.02E-07	4.07E-06
1470	ENSG00000248323.6	LUCAT1	1.27	3.04E-07	4.10E-06
1471	ENSG00000005700.14	IBTK	0.48	3.04E-07	4.10E-06
1472	ENSG00000182134.15	TDRKH	0.83	3.06E-07	4.12E-06
1473	ENSG00000205413.7	SAMD9	-0.93	3.10E-07	4.17E-06
1474	ENSG00000172594.12	SMPDL3A	0.61	3.13E-07	4.21E-06
1475	ENSG00000204136.10	GGTA1P	-2.38	3.18E-07	4.27E-06
1476	ENSG00000125319.14	C17orf53	1.03	3.21E-07	4.32E-06
1477	ENSG00000114805.17	PLCH1	1.49	3.23E-07	4.34E-06
1478	ENSG00000141076.17	UTP4	0.63	3.23E-07	4.34E-06
1479	ENSG00000185386.14	MAPK11	0.69	3.30E-07	4.43E-06
1480	ENSG00000136732.15	GYPC	-0.49	3.30E-07	4.43E-06
1481	ENSG00000166086.12	JAM3	-0.60	3.33E-07	4.46E-06
1482	ENSG00000049449.9	RCN1	0.65	3.39E-07	4.53E-06
1483	ENSG00000104320.13	NBN	0.51	3.43E-07	4.59E-06
1484	ENSG00000039560.13	RAI14	-0.50	3.44E-07	4.60E-06
1485	ENSG00000179195.15	ZNF664	-0.47	3.48E-07	4.64E-06
1486	ENSG00000231948.2	HS1BP3-IT1	2.45	3.50E-07	4.67E-06
1487	ENSG00000106588.10	PSMA2	0.56	3.51E-07	4.69E-06
1488	ENSG00000158258.16	CLSTN2	-1.58	3.53E-07	4.71E-06
1489	ENSG00000111145.7	ELK3	-0.66	3.57E-07	4.75E-06
1490	ENSG00000111077.17	TNS2	-0.76	3.58E-07	4.76E-06
1491	ENSG00000229474.6	PATL2	1.35	3.58E-07	4.76E-06
1492	ENSG00000164494.11	PDSS2	0.56	3.58E-07	4.76E-06
1493	ENSG00000163297.16	ANTXR2	-0.55	3.72E-07	4.93E-06
1494	ENSG00000033867.16	SLC4A7	-0.70	3.80E-07	5.04E-06
1495	ENSG00000167306.19	MYO5B	1.28	3.82E-07	5.07E-06
1496	ENSG00000110880.10	CORO1C	0.47	3.86E-07	5.12E-06
1497	ENSG00000145022.4	TCTA	0.50	3.87E-07	5.12E-06

1498	ENSG00000060982.14	BCAT1	-0.69	3.88E-07	5.13E-06
1499	ENSG00000240045.1	DWORF	3.68	3.96E-07	5.23E-06
1500	ENSG00000163710.8	PCOLCE2	-0.64	3.98E-07	5.26E-06
1501	ENSG00000148290.9	SURF1	0.56	4.00E-07	5.28E-06
1502	ENSG00000175938.6	ORAI3	-0.84	4.00E-07	5.28E-06
1503	ENSG00000167674.14	HDGFL2	-0.76	4.02E-07	5.31E-06
1504	ENSG00000168056.15	LTBP3	-0.74	4.07E-07	5.36E-06
1505	ENSG00000197045.12	GMFB	-0.52	4.10E-07	5.40E-06
1506	ENSG00000250378.3	AC114296.1	3.84	4.17E-07	5.50E-06
1507	ENSG00000171604.11	CXXC5	-0.69	4.27E-07	5.62E-06
1508	ENSG00000091129.19	NRCAM	1.43	4.36E-07	5.74E-06
1509	ENSG00000112297.14	CRYBG1	0.49	4.41E-07	5.79E-06
1510	ENSG00000115993.12	TRAK2	-0.56	4.41E-07	5.79E-06
1511	ENSG00000176720.5	BOK	0.76	4.44E-07	5.82E-06
1512	ENSG00000229644.6	NAMPTP1	1.39	4.46E-07	5.85E-06
1513	ENSG00000198252.11	STYX	0.53	4.48E-07	5.87E-06
1514	ENSG00000113389.15	NPR3	-0.68	4.49E-07	5.89E-06
1515	ENSG00000172935.8	MRGPRF	-1.06	4.51E-07	5.91E-06
1516	ENSG00000164099.3	PRSS12	-0.53	4.72E-07	6.17E-06
1517	ENSG00000106004.4	HOXA5	0.71	4.77E-07	6.23E-06
1518	ENSG00000139651.10	ZNF740	0.57	4.82E-07	6.30E-06
1519	ENSG00000104973.17	MED25	0.82	4.83E-07	6.30E-06
1520	ENSG00000134343.12	ANO3	1.29	4.83E-07	6.31E-06
1521	ENSG00000145147.19	SLIT2	-0.55	4.88E-07	6.37E-06
1522	ENSG00000109919.9	MTCH2	0.48	4.89E-07	6.37E-06
1523	ENSG00000137675.4	MMP27	-3.80	4.97E-07	6.47E-06
1524	ENSG00000151458.11	ANKRD50	-0.49	5.01E-07	6.52E-06
1525	ENSG00000158615.8	PPP1R15B	0.52	5.05E-07	6.56E-06
1526	ENSG00000137845.14	ADAM10	-0.59	5.05E-07	6.56E-06
1527	ENSG00000112852.6	PCDHB2	-1.29	5.06E-07	6.57E-06
1528	ENSG00000176595.3	KBTBD11	0.91	5.10E-07	6.62E-06
1529	ENSG00000132825.6	PPP1R3D	0.66	5.14E-07	6.66E-06
1530	ENSG00000155506.16	LARP1	0.48	5.13E-07	6.66E-06
1531	ENSG00000261701.7	HPR	2.34	5.22E-07	6.77E-06
1532	ENSG00000145901.15	TNIP1	0.51	5.31E-07	6.87E-06
1533	ENSG00000131171.12	SH3BGRL	-0.46	5.36E-07	6.94E-06
1534	ENSG00000164663.14	USP49	0.68	5.48E-07	7.09E-06
1535	ENSG00000153048.10	CARHSP1	-0.65	5.53E-07	7.15E-06
1536	ENSG00000136842.13	TMOD1	1.71	5.66E-07	7.31E-06
1537	ENSG00000159023.21	EPB41	0.49	5.66E-07	7.31E-06
1538	ENSG00000134684.10	YARS	0.48	5.68E-07	7.32E-06
1539	ENSG00000089157.15	RPLP0	0.54	5.71E-07	7.36E-06
1540	ENSG00000171862.10	PTEN	-0.46	5.73E-07	7.38E-06
1541	ENSG00000244005.12	NFS1	0.50	5.75E-07	7.39E-06
1542	ENSG00000131844.15	MCCC2	0.47	5.80E-07	7.45E-06
1543	ENSG00000072041.16	SLC6A15	0.75	5.90E-07	7.58E-06
1544	ENSG00000125124.11	BBS2	0.51	5.92E-07	7.61E-06
1545	ENSG00000087263.16	OGFOD1	0.56	5.96E-07	7.65E-06
1546	ENSG00000239911.2	PRKAG2-AS1	2.24	6.03E-07	7.74E-06
1547	ENSG00000166147.13	FBN1	-0.57	6.03E-07	7.74E-06

1548	ENSG00000149809.14	TM7SF2	0.83	6.05E-07	7.75E-06
1549	ENSG00000173065.13	FAM222B	0.68	6.07E-07	7.78E-06
1550	ENSG00000168297.15	PXK	-0.60	6.28E-07	8.04E-06
1551	ENSG00000182180.13	MRPS16	0.46	6.38E-07	8.16E-06
1552	ENSG00000276550.4	HERC2P2	-0.61	6.45E-07	8.25E-06
1553	ENSG00000105856.13	HBP1	-0.52	6.47E-07	8.26E-06
1554	ENSG00000180992.6	MRPL14	0.63	6.56E-07	8.38E-06
1555	ENSG00000253159.2	PCDHGA12	-0.84	6.58E-07	8.40E-06
1556	ENSG00000048544.5	MRPS10	0.50	6.76E-07	8.61E-06
1557	ENSG00000140740.10	UQCRC2	0.42	6.79E-07	8.65E-06
1558	ENSG00000174718.11	KIAA1551	-0.55	6.79E-07	8.65E-06
1559	ENSG00000110013.12	SIAE	-0.58	6.85E-07	8.71E-06
1560	ENSG00000143367.15	TUFT1	-0.61	6.86E-07	8.73E-06
1561	ENSG00000160211.17	G6PD	0.61	6.94E-07	8.82E-06
1562	ENSG00000085788.13	DDHD2	0.51	7.07E-07	8.98E-06
1563	ENSG00000270696.1	AC005034.3	0.67	7.08E-07	8.98E-06
1564	ENSG00000139174.11	PRICKLE1	-0.71	7.08E-07	8.98E-06
1565	ENSG00000147697.8	GSDMC	-1.00	7.10E-07	9.00E-06
1566	ENSG00000084070.11	SMAP2	-0.60	7.11E-07	9.00E-06
1567	ENSG00000109654.14	TRIM2	-0.57	7.21E-07	9.12E-06
1568	ENSG00000144857.14	BOC	-0.92	7.21E-07	9.12E-06
1569	ENSG00000197119.12	SLC25A29	-0.82	7.29E-07	9.21E-06
1570	ENSG00000196743.8	GM2A	0.56	7.38E-07	9.31E-06
1571	ENSG00000091527.15	CDV3	0.44	7.37E-07	9.31E-06
1572	ENSG00000117118.9	SDHB	0.51	7.44E-07	9.39E-06
1573	ENSG00000123384.13	LRP1	-0.79	7.47E-07	9.42E-06
1574	ENSG00000004142.11	POLDIP2	0.45	7.62E-07	9.60E-06
1575	ENSG00000272888.6	LINC01578	-0.65	7.65E-07	9.64E-06
1576	ENSG00000185201.16	IFITM2	-0.62	7.66E-07	9.64E-06
1577	ENSG00000168004.9	HRASLS5	0.50	7.67E-07	9.64E-06
1578	ENSG00000076864.19	RAP1GAP	-1.49	7.71E-07	9.69E-06
1579	ENSG00000141753.6	IGFBP4	-0.55	7.72E-07	9.69E-06
1580	ENSG00000140859.15	KIFC3	0.72	7.75E-07	9.73E-06
1581	ENSG00000088298.12	EDEM2	0.64	7.79E-07	9.78E-06
1582	ENSG00000114248.9	LRRC31	4.37	7.87E-07	9.87E-06
1583	ENSG00000123213.22	NLN	-0.62	8.13E-07	1.02E-05
1584	ENSG00000117479.13	SLC19A2	0.63	8.27E-07	1.04E-05
1585	ENSG00000142192.20	APP	-0.46	8.37E-07	1.05E-05
1586	ENSG00000260822.1	AC004656.1	1.22	8.57E-07	1.07E-05
1587	ENSG00000161011.19	SQSTM1	0.42	8.60E-07	1.07E-05
1588	ENSG00000205186.2	FABP9	6.25	8.78E-07	1.10E-05
1589	ENSG00000237576.1	LINC01888	4.36	8.78E-07	1.10E-05
1590	ENSG00000115361.7	ACADL	0.95	8.81E-07	1.10E-05
1591	ENSG00000141458.12	NPC1	0.51	8.84E-07	1.10E-05
1592	ENSG00000135272.10	MDFIC	0.48	8.99E-07	1.12E-05
1593	ENSG00000181625.17	SLX1B	1.01	9.20E-07	1.15E-05
1594	ENSG00000139974.15	SLC38A6	-0.74	9.21E-07	1.15E-05
1595	ENSG00000132535.19	DLG4	-0.66	9.22E-07	1.15E-05
1596	ENSG00000198369.9	SPRED2	-0.81	9.28E-07	1.15E-05
1597	ENSG00000176410.7	DNAJC30	0.59	9.30E-07	1.16E-05

1598	ENSG00000134480.14	CCNH	0.54	9.34E-07	1.16E-05
1599	ENSG00000173068.17	BNC2	-0.55	9.46E-07	1.17E-05
1600	ENSG00000159842.15	ABR	-0.49	9.54E-07	1.18E-05
1601	ENSG00000173253.15	DMRT2	1.55	9.59E-07	1.19E-05
1602	ENSG00000088832.16	FKBP1A	0.52	9.64E-07	1.19E-05
1603	ENSG00000131269.16	ABCB7	0.57	9.84E-07	1.22E-05
1604	ENSG00000170581.13	STAT2	-0.47	9.85E-07	1.22E-05
1605	ENSG00000105088.8	OLFM2	-0.68	9.94E-07	1.23E-05
1606	ENSG00000181873.12	IBA57	0.73	1.01E-06	1.24E-05
1607	ENSG00000101846.6	STS	0.65	1.01E-06	1.24E-05
1608	ENSG00000143797.11	MBOAT2	-0.77	1.01E-06	1.24E-05
1609	ENSG00000171861.10	MRM3	0.73	1.01E-06	1.25E-05
1610	ENSG00000164294.13	GPX8	-0.63	1.02E-06	1.26E-05
1611	ENSG00000060491.16	OGFR	0.68	1.03E-06	1.26E-05
1612	ENSG00000221866.9	PLXNA4	0.92	1.03E-06	1.27E-05
1613	ENSG00000167601.11	AXL	-0.81	1.03E-06	1.27E-05
1614	ENSG00000177425.10	PAWR	-0.59	1.03E-06	1.27E-05
1615	ENSG00000101849.16	TBL1X	-0.63	1.04E-06	1.28E-05
1616	ENSG00000176788.8	BASP1	-0.78	1.04E-06	1.28E-05
1617	ENSG00000162929.13	KIAA1841	-0.77	1.04E-06	1.28E-05
1618	ENSG00000184304.14	PRKD1	-0.65	1.05E-06	1.28E-05
1619	ENSG00000173442.12	EHBP1L1	-0.66	1.07E-06	1.31E-05
1620	ENSG00000160209.18	PDXK	0.45	1.08E-06	1.32E-05
1621	ENSG00000170558.8	CDH2	-0.75	1.09E-06	1.33E-05
1622	ENSG00000090263.15	MRPS33	0.51	1.09E-06	1.34E-05
1623	ENSG00000235180.1	LINC00601	2.03	1.10E-06	1.34E-05
1624	ENSG00000137142.4	IGFBPL1	1.48	1.10E-06	1.34E-05
1625	ENSG00000126778.9	SIX1	-0.59	1.10E-06	1.35E-05
1626	ENSG00000136026.13	CKAP4	-0.56	1.11E-06	1.35E-05
1627	ENSG00000113013.13	HSPA9	0.41	1.13E-06	1.38E-05
1628	ENSG00000090565.15	RAB11FIP3	-0.68	1.14E-06	1.39E-05
1629	ENSG00000158528.11	PPP1R9A	0.80	1.14E-06	1.39E-05
1630	ENSG00000279208.1	CR381653.2	-1.17	1.15E-06	1.39E-05
1631	ENSG00000066032.18	CTNNA2	2.33	1.15E-06	1.40E-05
1632	ENSG00000070761.7	CFAP20	0.66	1.15E-06	1.40E-05
1633	ENSG00000138594.13	TMOD3	-0.54	1.16E-06	1.41E-05
1634	ENSG00000168003.16	SLC3A2	0.48	1.17E-06	1.42E-05
1635	ENSG00000109113.19	RAB34	-0.56	1.17E-06	1.42E-05
1636	ENSG00000198753.11	PLXNB3	-0.94	1.17E-06	1.42E-05
1637	ENSG00000144381.16	HSPD1	0.55	1.19E-06	1.45E-05
1638	ENSG00000228716.6	DHFR	0.57	1.20E-06	1.45E-05
1639	ENSG00000253374.5	AC023644.1	5.77	1.20E-06	1.45E-05
1640	ENSG00000130827.6	PLXNA3	-0.70	1.20E-06	1.45E-05
1641	ENSG00000157214.13	STEAP2	-0.51	1.21E-06	1.46E-05
1642	ENSG00000186340.15	THBS2	-0.64	1.21E-06	1.47E-05
1643	ENSG00000135951.14	TSGA10	-1.00	1.23E-06	1.49E-05
1644	ENSG00000129158.10	SERGEF	0.73	1.23E-06	1.49E-05
1645	ENSG00000103202.12	NME4	0.53	1.24E-06	1.49E-05
1646	ENSG00000171603.17	CLSTN1	-0.56	1.24E-06	1.49E-05
1647	ENSG00000154678.16	PDE1C	-0.83	1.25E-06	1.51E-05

1648	ENSG00000135018.13	UBQLN1	0.40	1.26E-06	1.52E-05
1649	ENSG00000125637.15	PSD4	1.43	1.26E-06	1.52E-05
1650	ENSG00000006468.13	ETV1	-1.18	1.28E-06	1.54E-05
1651	ENSG00000240024.5	LINC00888	1.06	1.28E-06	1.54E-05
1652	ENSG00000158856.18	DMTN	0.99	1.29E-06	1.55E-05
1653	ENSG00000265298.1	AC132812.1	-1.26	1.30E-06	1.55E-05
1654	ENSG00000033170.16	FUT8	-0.69	1.30E-06	1.56E-05
1655	ENSG00000167965.17	MLST8	0.68	1.31E-06	1.56E-05
1656	ENSG00000171444.17	MCC	-0.53	1.31E-06	1.57E-05
1657	ENSG00000108528.13	SLC25A11	0.53	1.32E-06	1.57E-05
1658	ENSG00000253953.2	PCDHGB4	-0.65	1.32E-06	1.58E-05
1659	ENSG00000160293.16	VAV2	-0.83	1.33E-06	1.59E-05
1660	ENSG00000115594.11	IL1R1	-0.82	1.35E-06	1.61E-05
1661	ENSG00000130312.6	MRPL34	0.68	1.36E-06	1.63E-05
1662	ENSG00000148737.16	TCF7L2	-0.46	1.39E-06	1.66E-05
1663	ENSG00000215424.9	MCM3AP-AS1	-0.88	1.40E-06	1.66E-05
1664	ENSG00000164898.12	FMC1	0.86	1.40E-06	1.67E-05
1665	ENSG00000165678.20	GHITM	0.40	1.41E-06	1.68E-05
1666	ENSG00000226091.7	LINC00937	-1.85	1.41E-06	1.68E-05
1667	ENSG00000204176.13	SYT15	-0.81	1.41E-06	1.68E-05
1668	ENSG00000143514.16	TP53BP2	-0.59	1.42E-06	1.69E-05
1669	ENSG00000152601.17	MBNL1	-0.42	1.42E-06	1.69E-05
1670	ENSG00000169221.13	TBC1D10B	0.59	1.43E-06	1.69E-05
1671	ENSG00000012504.14	NR1H4	3.15	1.44E-06	1.71E-05
1672	ENSG00000285000.1	AC008581.2	1.06	1.44E-06	1.71E-05
1673	ENSG00000115310.17	RTN4	0.45	1.46E-06	1.73E-05
1674	ENSG00000139180.10	NDUFA9	0.51	1.47E-06	1.74E-05
1675	ENSG00000021762.19	OSBPL5	-0.85	1.47E-06	1.74E-05
1676	ENSG00000117020.16	AKT3	-0.52	1.48E-06	1.76E-05
1677	ENSG00000048162.20	NOP16	0.56	1.49E-06	1.76E-05
1678	ENSG00000136986.9	DERL1	0.51	1.50E-06	1.77E-05
1679	ENSG00000067082.14	KLF6	-0.58	1.51E-06	1.79E-05
1680	ENSG00000163132.6	MSX1	-0.79	1.53E-06	1.81E-05
1681	ENSG00000169627.7	BOLA2B	0.91	1.55E-06	1.83E-05
1682	ENSG00000160606.10	TLCD1	0.85	1.55E-06	1.83E-05
1683	ENSG00000197558.11	SSPO	-2.05	1.58E-06	1.86E-05
1684	ENSG00000173930.8	SLCO4C1	0.78	1.58E-06	1.86E-05
1685	ENSG00000107819.13	SFXN3	-0.64	1.59E-06	1.88E-05
1686	ENSG00000198353.7	HOXC4	-1.20	1.62E-06	1.90E-05
1687	ENSG00000248969.1	AC137810.1	3.36	1.62E-06	1.91E-05
1688	ENSG00000188542.9	DUSP28	0.90	1.65E-06	1.93E-05
1689	ENSG00000267469.1	AC005944.1	-1.07	1.65E-06	1.94E-05
1690	ENSG00000143158.10	MPC2	0.45	1.66E-06	1.95E-05
1691	ENSG00000206503.12	HLA-A	0.54	1.69E-06	1.99E-05
1692	ENSG00000205220.11	PSMB10	0.84	1.70E-06	2.00E-05
1693	ENSG00000167325.14	RRM1	-0.59	1.71E-06	2.00E-05
1694	ENSG00000114841.17	DNAH1	-0.87	1.73E-06	2.03E-05
1695	ENSG00000121005.8	CRISPLD1	2.67	1.74E-06	2.03E-05
1696	ENSG00000130304.16	SLC27A1	0.79	1.74E-06	2.03E-05
1697	ENSG00000109787.12	KLF3	0.44	1.74E-06	2.03E-05

1698	ENSG00000136295.14	TTYH3	-0.78	1.74E-06	2.03E-05
1699	ENSG00000175110.11	MRPS22	0.52	1.75E-06	2.05E-05
1700	ENSG00000064393.15	HIPK2	0.49	1.76E-06	2.05E-05
1701	ENSG00000169188.4	APEX2	0.59	1.78E-06	2.08E-05
1702	ENSG00000138095.18	LRPPRC	0.41	1.79E-06	2.09E-05
1703	ENSG00000242153.7	OFD1P6Y	1.61	1.82E-06	2.12E-05
1704	ENSG00000276223.1	AL118522.1	1.65	1.82E-06	2.12E-05
1705	ENSG00000114353.16	GNAI2	-0.51	1.85E-06	2.15E-05
1706	ENSG00000156113.22	KCNMA1	-0.78	1.85E-06	2.15E-05
1707	ENSG00000160221.17	GATD3A	0.69	1.85E-06	2.16E-05
1708	ENSG00000198146.4	ZNF770	-0.47	1.86E-06	2.16E-05
1709	ENSG00000141905.18	NFIC	-0.60	1.86E-06	2.16E-05
1710	ENSG00000163171.7	CDC42EP3	-0.71	1.88E-06	2.18E-05
1711	ENSG00000145908.12	ZNF300	-0.99	1.88E-06	2.18E-05
1712	ENSG00000120262.9	CCDC170	-1.12	1.89E-06	2.19E-05
1713	ENSG00000184557.4	SOCS3	-1.31	1.94E-06	2.25E-05
1714	ENSG00000124212.5	PTGIS	-0.53	1.96E-06	2.27E-05
1715	ENSG00000281655.1	AP000851.2	-2.83	1.99E-06	2.31E-05
1716	ENSG00000204922.4	UQCC3	0.95	2.01E-06	2.32E-05
1717	ENSG00000104611.11	SH2D4A	0.92	2.02E-06	2.33E-05
1718	ENSG00000169756.16	LIMS1	0.66	2.03E-06	2.35E-05
1719	ENSG00000020181.17	ADGRA2	-0.63	2.04E-06	2.35E-05
1720	ENSG00000174145.7	NWD2	5.68	2.06E-06	2.38E-05
1721	ENSG00000273259.3	AL049839.2	6.86	2.08E-06	2.40E-05
1722	ENSG00000279288.1	AC073346.2	-6.29	2.12E-06	2.44E-05
1723	ENSG00000159322.17	ADPGK	0.48	2.17E-06	2.49E-05
1724	ENSG00000172939.8	OXSR1	0.52	2.19E-06	2.52E-05
1725	ENSG00000246859.2	STARD4-AS1	-0.64	2.19E-06	2.52E-05
1726	ENSG00000215105.4	TTC3P1	-0.90	2.22E-06	2.55E-05
1727	ENSG00000143515.16	ATP8B2	-0.55	2.23E-06	2.56E-05
1728	ENSG00000160753.15	RUSC1	-0.65	2.27E-06	2.60E-05
1729	ENSG00000114626.17	ABTB1	-0.83	2.29E-06	2.63E-05
1730	ENSG00000166947.13	EPB42	4.66	2.31E-06	2.65E-05
1731	ENSG00000140545.14	MFGE8	-0.50	2.31E-06	2.65E-05
1732	ENSG00000163041.9	H3F3A	-0.40	2.33E-06	2.66E-05
1733	ENSG00000035403.17	VCL	-0.42	2.33E-06	2.67E-05
1734	ENSG00000081189.15	MEF2C	0.62	2.34E-06	2.68E-05
1735	ENSG00000137267.5	TUBB2A	0.71	2.35E-06	2.68E-05
1736	ENSG00000109436.7	TBC1D9	0.55	2.35E-06	2.68E-05
1737	ENSG00000165355.7	FBXO33	0.52	2.36E-06	2.70E-05
1738	ENSG00000146707.14	POMZP3	0.66	2.37E-06	2.70E-05
1739	ENSG00000132912.12	DCTN4	0.46	2.37E-06	2.71E-05
1740	ENSG00000229180.7	AC006001.3	0.58	2.38E-06	2.71E-05
1741	ENSG00000260923.6	LINC02193	0.78	2.38E-06	2.71E-05
1742	ENSG00000185513.16	L3MBTL1	0.76	2.39E-06	2.72E-05
1743	ENSG00000108797.11	CNTNAP1	-0.67	2.39E-06	2.72E-05
1744	ENSG00000247746.4	USP51	0.76	2.45E-06	2.78E-05
1745	ENSG00000214756.7	CSKMT	0.94	2.49E-06	2.83E-05
1746	ENSG00000198964.13	SGMS1	-0.55	2.49E-06	2.83E-05
1747	ENSG00000103642.11	LACTB	0.49	2.50E-06	2.84E-05

1748	ENSG00000139083.10	ETV6	-0.59	2.52E-06	2.86E-05
1749	ENSG00000242110.7	AMACR	0.77	2.53E-06	2.86E-05
1750	ENSG00000272921.1	AC005832.4	0.72	2.53E-06	2.86E-05
1751	ENSG00000186517.13	ARHGAP30	2.06	2.53E-06	2.87E-05
1752	ENSG00000155542.11	SETD9	0.70	2.57E-06	2.91E-05
1753	ENSG00000114786.16	ABHD14A-ACY1	0.92	2.58E-06	2.92E-05
1754	ENSG00000164932.12	CTHRC1	-0.57	2.58E-06	2.92E-05
1755	ENSG00000205542.10	TMSB4X	-0.62	2.60E-06	2.94E-05
1756	ENSG00000099308.10	MAST3	-0.98	2.63E-06	2.97E-05
1757	ENSG00000162407.8	PLPP3	-0.71	2.63E-06	2.97E-05
1758	ENSG00000152463.14	OLAH	1.72	2.66E-06	3.00E-05
1759	ENSG00000106591.3	MRPL32	0.50	2.67E-06	3.02E-05
1760	ENSG00000235687.9	LINC00993	2.71	2.72E-06	3.06E-05
1761	ENSG00000173706.13	HEG1	-0.59	2.74E-06	3.09E-05
1762	ENSG00000050748.17	MAPK9	0.47	2.78E-06	3.12E-05
1763	ENSG00000072310.16	SREBF1	-0.76	2.78E-06	3.12E-05
1764	ENSG00000203531.3	AC104772.1	2.48	2.81E-06	3.16E-05
1765	ENSG00000214548.16	MEG3	-0.62	2.81E-06	3.16E-05
1766	ENSG00000132837.14	DMGDH	0.79	2.82E-06	3.17E-05
1767	ENSG00000084234.17	APLP2	-0.43	2.85E-06	3.19E-05
1768	ENSG00000152642.10	GPD1L	0.57	2.96E-06	3.32E-05
1769	ENSG00000176974.19	SHMT1	0.54	2.96E-06	3.32E-05
1770	ENSG00000122417.15	ODF2L	-0.58	2.96E-06	3.32E-05
1771	ENSG00000164434.11	FABP7	1.75	2.98E-06	3.34E-05
1772	ENSG00000272667.1	AC012306.2	1.09	2.99E-06	3.34E-05
1773	ENSG00000237159.5	CNTFR-AS1	1.88	3.06E-06	3.42E-05
1774	ENSG00000109794.13	FAM149A	0.87	3.08E-06	3.44E-05
1775	ENSG00000117139.17	KDM5B	-0.44	3.08E-06	3.44E-05
1776	ENSG00000126785.12	RHOJ	-0.76	3.09E-06	3.45E-05
1777	ENSG00000087338.4	GMCL1	0.58	3.10E-06	3.46E-05
1778	ENSG00000147416.10	ATP6V1B2	0.45	3.12E-06	3.48E-05
1779	ENSG00000162231.13	NXF1	-0.46	3.14E-06	3.50E-05
1780	ENSG00000113163.16	COL4A3BP	0.45	3.16E-06	3.52E-05
1781	ENSG00000138032.20	PPM1B	0.47	3.17E-06	3.53E-05
1782	ENSG00000155256.17	ZFYVE27	0.52	3.20E-06	3.56E-05
1783	ENSG00000122034.14	GTF3A	0.45	3.23E-06	3.60E-05
1784	ENSG00000162618.13	ADGRL4	-1.03	3.24E-06	3.60E-05
1785	ENSG00000151806.13	GUF1	0.45	3.25E-06	3.61E-05
1786	ENSG00000177706.8	FAM20C	-0.77	3.26E-06	3.62E-05
1787	ENSG00000166483.10	WEE1	-0.73	3.29E-06	3.66E-05
1788	ENSG00000215251.3	FASTKD5	0.55	3.32E-06	3.69E-05
1789	ENSG00000142494.13	SLC47A1	1.03	3.35E-06	3.71E-05
1790	ENSG00000204131.9	NHSL2	-0.65	3.35E-06	3.72E-05
1791	ENSG00000203685.9	STUM	-1.76	3.36E-06	3.72E-05
1792	ENSG00000107863.17	ARHGAP21	-0.45	3.38E-06	3.74E-05
1793	ENSG00000204642.13	HLA-F	1.64	3.42E-06	3.78E-05
1794	ENSG00000214300.7	SPDYE3	0.87	3.42E-06	3.78E-05
1795	ENSG00000180155.19	LYNX1	-0.79	3.42E-06	3.78E-05
1796	ENSG00000109066.13	TMEM104	0.66	3.43E-06	3.79E-05
1797	ENSG00000133935.6	ERG28	0.54	3.45E-06	3.81E-05

1798	ENSG00000138434.16	ITPRID2	0.42	3.45E-06	3.81E-05
1799	ENSG00000111328.6	CDK2AP1	-0.53	3.45E-06	3.81E-05
1800	ENSG00000134160.13	TRPM1	-2.02	3.47E-06	3.83E-05
1801	ENSG00000122545.19	SEPT7	-0.47	3.49E-06	3.84E-05
1802	ENSG00000263089.1	AC007114.2	2.03	3.52E-06	3.87E-05
1803	ENSG00000167775.10	CD320	0.73	3.56E-06	3.92E-05
1804	ENSG00000178597.6	PSAPL1	6.76	3.57E-06	3.93E-05
1805	ENSG00000150051.13	MKX	-0.73	3.63E-06	3.99E-05
1806	ENSG00000164970.14	FAM219A	0.52	3.65E-06	4.01E-05
1807	ENSG00000146676.8	PURB	0.43	3.65E-06	4.01E-05
1808	ENSG00000088280.18	ASAP3	-0.51	3.68E-06	4.04E-05
1809	ENSG00000173846.12	PLK3	-0.97	3.69E-06	4.04E-05
1810	ENSG00000261296.1	AC126323.6	1.47	3.71E-06	4.07E-05
1811	ENSG00000134574.11	DDB2	-0.62	3.72E-06	4.07E-05
1812	ENSG00000102996.4	MMP15	0.69	3.75E-06	4.11E-05
1813	ENSG00000116285.12	ERRFI1	-0.62	3.75E-06	4.11E-05
1814	ENSG00000283361.2	CFAP97D2	1.98	3.83E-06	4.19E-05
1815	ENSG00000079150.17	FKBP7	-0.83	3.83E-06	4.19E-05
1816	ENSG00000151617.16	EDNRA	-0.77	3.85E-06	4.20E-05
1817	ENSG00000159111.12	MRPL10	0.47	3.86E-06	4.21E-05
1818	ENSG00000188257.11	PLA2G2A	-0.61	3.86E-06	4.21E-05
1819	ENSG00000134308.13	YWHAQ	-0.52	3.88E-06	4.23E-05
1820	ENSG00000132589.15	FLOT2	0.66	3.98E-06	4.34E-05
1821	ENSG00000140044.12	JDP2	-0.62	3.98E-06	4.34E-05
1822	ENSG00000102802.9	MEDAG	-0.92	4.00E-06	4.35E-05
1823	ENSG00000154945.6	ANKRD40	0.52	4.00E-06	4.35E-05
1824	ENSG00000126950.7	TMEM35A	-1.32	4.00E-06	4.35E-05
1825	ENSG00000166831.8	RBPMS2	-0.96	4.08E-06	4.43E-05
1826	ENSG00000117625.13	RCOR3	-0.48	4.09E-06	4.44E-05
1827	ENSG00000157911.10	PEX10	0.79	4.10E-06	4.45E-05
1828	ENSG00000076248.10	UNG	0.55	4.11E-06	4.46E-05
1829	ENSG00000087088.19	BAX	-0.68	4.16E-06	4.51E-05
1830	ENSG00000173848.18	NET1	0.52	4.17E-06	4.52E-05
1831	ENSG00000131477.10	RAMP2	1.19	4.21E-06	4.57E-05
1832	ENSG00000173867.10	AC013489.1	1.17	4.27E-06	4.62E-05
1833	ENSG00000169750.8	RAC3	0.77	4.32E-06	4.67E-05
1834	ENSG00000164106.7	SCRG1	-0.84	4.34E-06	4.70E-05
1835	ENSG00000188706.12	ZDHHC9	0.44	4.39E-06	4.75E-05
1836	ENSG00000122707.11	RECK	-0.54	4.40E-06	4.75E-05
1837	ENSG00000159899.14	NPR2	-0.58	4.44E-06	4.79E-05
1838	ENSG00000113583.7	C5orf15	-0.49	4.47E-06	4.83E-05
1839	ENSG00000204160.11	ZDHHC18	0.67	4.55E-06	4.91E-05
1840	ENSG00000104728.15	ARHGEF10	-0.79	4.60E-06	4.96E-05
1841	ENSG00000140937.13	CDH11	-0.61	4.60E-06	4.96E-05
1842	ENSG00000070540.12	WIPI1	-0.50	4.60E-06	4.96E-05
1843	ENSG00000181513.14	ACBD4	0.91	4.76E-06	5.13E-05
1844	ENSG00000172296.12	SPTLC3	-0.83	4.78E-06	5.15E-05
1845	ENSG00000157227.12	MMP14	-0.50	4.82E-06	5.18E-05
1846	ENSG00000019549.11	SNAI2	-0.89	4.83E-06	5.19E-05
1847	ENSG00000160953.15	MUM1	-0.57	4.83E-06	5.19E-05

1848	ENSG00000119326.14	CTNNAL1	-0.51	4.84E-06	5.20E-05
1849	ENSG00000153485.5	TMEM251	0.69	4.86E-06	5.22E-05
1850	ENSG00000176136.6	MC5R	3.30	4.88E-06	5.23E-05
1851	ENSG00000111181.12	SLC6A12	2.33	4.88E-06	5.23E-05
1852	ENSG00000143850.14	PLEKHA6	1.65	4.88E-06	5.23E-05
1853	ENSG00000135723.13	FHOD1	-0.71	4.88E-06	5.23E-05
1854	ENSG00000143376.13	SNX27	0.53	4.89E-06	5.23E-05
1855	ENSG00000259976.3	AC093010.3	-0.57	4.99E-06	5.34E-05
1856	ENSG00000143839.14	REN	2.12	5.04E-06	5.38E-05
1857	ENSG00000114021.11	NIT2	0.43	5.07E-06	5.42E-05
1858	ENSG00000102468.10	HTR2A	-0.81	5.07E-06	5.42E-05
1859	ENSG00000123836.14	PFKFB2	0.63	5.10E-06	5.44E-05
1860	ENSG00000109133.12	TMEM33	0.47	5.11E-06	5.45E-05
1861	ENSG00000137076.20	TLN1	-0.47	5.18E-06	5.52E-05
1862	ENSG00000118689.14	FOXO3	-0.59	5.21E-06	5.55E-05
1863	ENSG00000102878.16	HSF4	-1.36	5.22E-06	5.56E-05
1864	ENSG00000139278.9	GLIPR1	-0.51	5.23E-06	5.56E-05
1865	ENSG00000105953.14	OGDH	0.46	5.27E-06	5.61E-05
1866	ENSG00000184206.11	GOLGA6L4	-0.74	5.31E-06	5.65E-05
1867	ENSG00000185585.19	OLFML2A	-0.90	5.35E-06	5.69E-05
1868	ENSG00000214078.12	CPNE1	-0.49	5.36E-06	5.69E-05
1869	ENSG00000134287.9	ARF3	0.43	5.39E-06	5.72E-05
1870	ENSG00000128655.17	PDE11A	-0.74	5.40E-06	5.72E-05
1871	ENSG00000183648.9	NDUFB1	0.49	5.43E-06	5.75E-05
1872	ENSG00000126351.12	THRA	-0.51	5.43E-06	5.76E-05
1873	ENSG00000154719.13	MRPL39	0.52	5.46E-06	5.78E-05
1874	ENSG00000169410.9	PTPN9	-0.63	5.47E-06	5.79E-05
1875	ENSG00000256235.2	SMIM3	0.40	5.59E-06	5.91E-05
1876	ENSG00000049323.15	LTBP1	-0.54	5.63E-06	5.95E-05
1877	ENSG00000106266.10	SNX8	0.66	5.63E-06	5.96E-05
1878	ENSG00000267882.2	AL031666.2	2.25	5.64E-06	5.96E-05
1879	ENSG00000141447.17	OSBPL1A	0.49	5.64E-06	5.96E-05
1880	ENSG00000187051.8	RPS19BP1	0.55	5.70E-06	6.01E-05
1881	ENSG00000144357.16	UBR3	0.43	5.71E-06	6.02E-05
1882	ENSG00000165494.11	PCF11	0.50	5.76E-06	6.07E-05
1883	ENSG00000047644.18	WWC3	-0.60	5.78E-06	6.09E-05
1884	ENSG00000213064.9	SFT2D2	-0.45	5.88E-06	6.19E-05
1885	ENSG00000116459.10	ATP5PB	0.45	5.95E-06	6.26E-05
1886	ENSG00000141664.9	ZCCHC2	-0.58	5.98E-06	6.29E-05
1887	ENSG00000162415.6	ZSWIM5	1.55	6.09E-06	6.40E-05
1888	ENSG00000143079.14	CTTNBP2NL	-0.51	6.15E-06	6.47E-05
1889	ENSG00000168701.18	TMEM208	0.55	6.16E-06	6.47E-05
1890	ENSG00000267436.1	AC005786.3	1.46	6.18E-06	6.49E-05
1891	ENSG00000187097.12	ENTPD5	0.50	6.21E-06	6.51E-05
1892	ENSG00000178057.14	NDUFAF3	0.51	6.24E-06	6.54E-05
1893	ENSG00000095321.16	CRAT	0.53	6.29E-06	6.59E-05
1894	ENSG00000115107.19	STEAP3	-0.76	6.36E-06	6.66E-05
1895	ENSG00000081923.13	ATP8B1	-0.73	6.39E-06	6.69E-05
1896	ENSG00000135245.9	HILPDA	0.59	6.44E-06	6.74E-05
1897	ENSG00000145332.13	KLHL8	0.52	6.47E-06	6.77E-05

1898	ENSG00000231535.5	LINC00278	0.84	6.53E-06	6.83E-05
1899	ENSG00000144810.15	COL8A1	-0.58	6.59E-06	6.88E-05
1900	ENSG00000163516.13	ANKZF1	-0.64	6.64E-06	6.93E-05
1901	ENSG00000152767.16	FARP1	-0.42	6.66E-06	6.95E-05
1902	ENSG00000171421.12	MRPL36	0.57	6.67E-06	6.95E-05
1903	ENSG00000172667.10	ZMAT3	-0.45	6.70E-06	6.98E-05
1904	ENSG00000120324.8	PCDHB10	-1.39	6.70E-06	6.98E-05
1905	ENSG00000146242.8	TPBG	-0.71	6.82E-06	7.11E-05
1906	ENSG00000169635.9	HIC2	1.19	6.83E-06	7.11E-05
1907	ENSG00000205707.10	ETFRF1	0.51	6.89E-06	7.17E-05
1908	ENSG00000119041.10	GTF3C3	0.56	6.95E-06	7.22E-05
1909	ENSG00000177732.8	SOX12	-0.72	6.98E-06	7.26E-05
1910	ENSG00000073464.11	CLCN4	0.88	6.99E-06	7.27E-05
1911	ENSG00000064886.13	CHI3L2	0.48	7.06E-06	7.33E-05
1912	ENSG00000185070.10	FLRT2	-0.80	7.13E-06	7.40E-05
1913	ENSG00000285427.1	SOD2-OT1	1.85	7.15E-06	7.41E-05
1914	ENSG00000127955.16	GNAI1	-0.42	7.15E-06	7.41E-05
1915	ENSG00000198692.9	EIF1AY	0.57	7.21E-06	7.47E-05
1916	ENSG00000110237.4	ARHGEF17	-0.63	7.22E-06	7.47E-05
1917	ENSG00000183960.8	KCNH8	3.05	7.25E-06	7.51E-05
1918	ENSG00000011376.10	LARS2	0.48	7.26E-06	7.51E-05
1919	ENSG00000117528.12	ABCD3	0.44	7.30E-06	7.55E-05
1920	ENSG00000080561.13	MID2	0.61	7.31E-06	7.55E-05
1921	ENSG00000152104.11	PTPN14	-0.46	7.36E-06	7.61E-05
1922	ENSG00000082701.15	GSK3B	-0.41	7.37E-06	7.61E-05
1923	ENSG00000038427.15	VCAN	-0.65	7.42E-06	7.65E-05
1924	ENSG00000163319.10	MRPS18C	0.54	7.43E-06	7.66E-05
1925	ENSG00000134532.16	SOX5	0.95	7.44E-06	7.66E-05
1926	ENSG00000100325.14	ASCC2	0.46	7.44E-06	7.67E-05
1927	ENSG00000126749.15	EMG1	0.45	7.48E-06	7.70E-05
1928	ENSG00000136897.7	MRPL50	0.47	7.65E-06	7.87E-05
1929	ENSG00000005810.17	MYCBP2	-0.53	7.68E-06	7.90E-05
1930	ENSG00000156639.11	ZFAND3	-0.43	7.73E-06	7.95E-05
1931	ENSG00000111057.10	KRT18	-1.10	7.77E-06	7.98E-05
1932	ENSG00000172260.14	NEGR1	-0.57	7.79E-06	7.99E-05
1933	ENSG00000142227.10	EMP3	-0.68	7.78E-06	7.99E-05
1934	ENSG00000178127.12	NDUFV2	0.42	7.81E-06	8.01E-05
1935	ENSG00000168904.14	LRRC28	0.57	7.84E-06	8.04E-05
1936	ENSG00000254814.1	AP003031.1	-1.39	7.84E-06	8.04E-05
1937	ENSG00000070961.15	ATP2B1	-0.56	7.92E-06	8.11E-05
1938	ENSG00000147155.10	EBP	0.67	7.96E-06	8.15E-05
1939	ENSG00000261740.6	BOLA2-SMG1P6	0.57	8.00E-06	8.18E-05
1940	ENSG00000154174.7	TOMM70	0.43	8.09E-06	8.27E-05
1941	ENSG00000180914.10	OXTR	-0.66	8.23E-06	8.41E-05
1942	ENSG00000079308.18	TNS1	-0.59	8.24E-06	8.41E-05
1943	ENSG00000169604.19	ANTXR1	-0.46	8.28E-06	8.46E-05
1944	ENSG00000233237.6	LINC00472	-1.04	8.29E-06	8.46E-05
1945	ENSG00000162620.15	LRRIQ3	-1.27	8.29E-06	8.46E-05
1946	ENSG00000141971.12	MVB12A	0.68	8.38E-06	8.54E-05
1947	ENSG00000165526.8	RPUSD4	0.52	8.38E-06	8.54E-05

1948	ENSG00000137720.7	C11orf1	0.70	8.39E-06	8.54E-05
1949	ENSG00000243317.7	STMP1	-0.45	8.39E-06	8.54E-05
1950	ENSG00000180425.11	C11orf71	0.73	8.39E-06	8.54E-05
1951	ENSG00000107537.13	PHYH	0.45	8.50E-06	8.64E-05
1952	ENSG00000178695.5	KCTD12	-0.60	8.54E-06	8.68E-05
1953	ENSG00000151176.7	PLBD2	-0.47	8.61E-06	8.75E-05
1954	ENSG00000141441.15	GAREM1	0.58	8.63E-06	8.76E-05
1955	ENSG00000124257.6	NEURL2	1.32	8.67E-06	8.80E-05
1956	ENSG00000121210.15	TMEM131L	0.54	8.78E-06	8.91E-05
1957	ENSG00000105245.9	NUMBL	-0.72	8.79E-06	8.91E-05
1958	ENSG00000137872.16	SEMA6D	0.77	8.87E-06	8.99E-05
1959	ENSG00000181588.16	MEX3D	-0.79	8.92E-06	9.03E-05
1960	ENSG00000125912.10	NCLN	0.69	8.93E-06	9.03E-05
1961	ENSG00000065923.9	SLC9A7	-0.80	8.93E-06	9.04E-05
1962	ENSG00000159915.12	ZNF233	0.96	8.98E-06	9.08E-05
1963	ENSG00000107317.12	PTGDS	-0.80	9.01E-06	9.11E-05
1964	ENSG00000221813.4	OR6B1	2.83	9.11E-06	9.21E-05
1965	ENSG00000133639.4	BTG1	-0.61	9.14E-06	9.22E-05
1966	ENSG00000226137.5	BAIAP2-DT	-0.71	9.27E-06	9.35E-05
1967	ENSG00000279232.2	AC008522.1	-0.83	9.28E-06	9.36E-05
1968	ENSG00000008311.14	AASS	-0.61	9.30E-06	9.37E-05
1969	ENSG00000178860.8	MSC	-0.82	9.33E-06	9.40E-05
1970	ENSG00000101951.16	PAGE4	5.86	9.44E-06	9.50E-05
1971	ENSG00000174348.13	PODN	-0.53	9.48E-06	9.54E-05
1972	ENSG00000226031.5	FGF13-AS1	3.58	9.56E-06	9.62E-05
1973	ENSG00000196576.14	PLXNB2	-0.63	9.59E-06	9.64E-05
1974	ENSG00000107984.9	DKK1	-0.87	9.59E-06	9.64E-05
1975	ENSG00000010295.19	IFFO1	-0.64	9.62E-06	9.67E-05
1976	ENSG00000165626.17	BEND7	0.57	9.69E-06	9.72E-05
1977	ENSG00000101751.10	POLI	-0.52	9.69E-06	9.73E-05
1978	ENSG00000160194.17	NDUFV3	0.45	9.91E-06	9.94E-05
1979	ENSG00000111186.12	WNT5B	-0.83	9.92E-06	9.94E-05
1980	ENSG00000113119.12	TMCO6	0.75	9.93E-06	9.94E-05
1981	ENSG00000114450.9	GNB4	-0.53	9.92E-06	9.94E-05
1982	ENSG00000259158.4	ADAM20P1	1.97	9.94E-06	9.95E-05
1983	ENSG00000176842.14	IRX5	-0.90	9.98E-06	9.98E-05
1984	ENSG00000163541.11	SUCLG1	0.46	9.99E-06	9.99E-05
1985	ENSG00000272622.1	AC010735.2	-0.97	9.99E-06	9.99E-05
1986	ENSG00000116161.17	CACYBP	0.49	1.00E-05	1.00E-04
1987	ENSG00000235531.9	MSC-AS1	-0.55	1.01E-05	1.01E-04
1988	ENSG00000005073.5	HOXA11	-0.69	1.03E-05	1.03E-04
1989	ENSG00000061273.17	HDAC7	-0.49	1.04E-05	1.03E-04
1990	ENSG00000232098.3	AC012313.1	-0.93	1.04E-05	1.03E-04
1991	ENSG00000088179.8	PTPN4	0.49	1.04E-05	1.04E-04
1992	ENSG00000186312.10	CA5BP1	0.90	1.05E-05	1.05E-04
1993	ENSG00000213160.9	KLHL23	1.15	1.05E-05	1.05E-04
1994	ENSG00000105819.13	PMPCB	0.41	1.08E-05	1.07E-04
1995	ENSG00000172765.17	TMCC1	0.44	1.08E-05	1.08E-04
1996	ENSG00000178980.14	SELENOW	-0.55	1.08E-05	1.08E-04
1997	ENSG00000162692.11	VCAM1	-0.96	1.09E-05	1.08E-04

1998	ENSG00000138101.18	DTNB	0.91	1.09E-05	1.08E-04
1999	ENSG00000184076.13	UQCR10	0.48	1.10E-05	1.09E-04
2000	ENSG00000198796.6	ALPK2	-0.60	1.11E-05	1.11E-04
2001	ENSG00000099725.14	PRKY	-0.91	1.13E-05	1.12E-04
2002	ENSG00000270959.1	LPP-AS2	-0.89	1.13E-05	1.12E-04
2003	ENSG00000269985.1	AL021328.1	3.37	1.14E-05	1.13E-04
2004	ENSG00000272695.1	GAS6-DT	-1.02	1.14E-05	1.13E-04
2005	ENSG00000240857.1	RDH14	0.53	1.15E-05	1.14E-04
2006	ENSG00000069966.18	GNB5	-0.66	1.16E-05	1.15E-04
2007	ENSG00000100364.18	KIAA0930	-0.64	1.16E-05	1.15E-04
2008	ENSG00000160345.12	C9orf116	1.30	1.17E-05	1.15E-04
2009	ENSG00000185088.13	RPS27L	-0.54	1.17E-05	1.15E-04
2010	ENSG00000162851.7	TFB2M	0.53	1.18E-05	1.17E-04
2011	ENSG00000148700.14	ADD3	0.47	1.18E-05	1.17E-04
2012	ENSG00000132591.11	ERAL1	0.45	1.20E-05	1.18E-04
2013	ENSG00000111450.13	STX2	-0.61	1.21E-05	1.20E-04
2014	ENSG00000112039.4	FANCE	-0.64	1.22E-05	1.21E-04
2015	ENSG00000237505.7	PKN2-AS1	1.05	1.24E-05	1.22E-04
2016	ENSG00000135926.14	TMBIM1	0.43	1.24E-05	1.22E-04
2017	ENSG00000198542.13	ITGBL1	-0.66	1.24E-05	1.22E-04
2018	ENSG00000071794.15	HLTF	0.52	1.26E-05	1.23E-04
2019	ENSG00000215483.10	LINC00598	0.94	1.26E-05	1.23E-04
2020	ENSG00000279330.1	AJ003147.3	1.02	1.29E-05	1.26E-04
2021	ENSG00000164967.9	RPP25L	0.79	1.29E-05	1.27E-04
2022	ENSG00000121964.14	GTDC1	0.52	1.30E-05	1.28E-04
2023	ENSG00000244720.1	AC055748.1	5.18	1.31E-05	1.28E-04
2024	ENSG00000120832.9	MTERF2	0.50	1.31E-05	1.28E-04
2025	ENSG00000151892.14	GFRA1	-1.00	1.31E-05	1.28E-04
2026	ENSG00000018510.14	AGPS	0.44	1.31E-05	1.29E-04
2027	ENSG00000108061.11	SHOC2	-0.50	1.32E-05	1.29E-04
2028	ENSG00000230368.2	FAM41C	1.02	1.32E-05	1.29E-04
2029	ENSG00000004700.15	RECQL	-0.56	1.32E-05	1.29E-04
2030	ENSG00000005022.5	SLC25A5	0.51	1.35E-05	1.32E-04
2031	ENSG00000006007.11	GDE1	0.50	1.35E-05	1.32E-04
2032	ENSG00000166562.8	SEC11C	0.64	1.36E-05	1.33E-04
2033	ENSG00000149187.18	CELF1	0.37	1.37E-05	1.33E-04
2034	ENSG00000118655.5	DCLRE1B	-0.69	1.37E-05	1.33E-04
2035	ENSG00000165376.10	CLDN2	2.11	1.38E-05	1.34E-04
2036	ENSG00000253326.2	AL606534.4	1.89	1.38E-05	1.34E-04
2037	ENSG00000128309.16	MPST	0.58	1.38E-05	1.34E-04
2038	ENSG00000182752.9	PAPPA	-0.52	1.39E-05	1.35E-04
2039	ENSG00000224881.1	AC068279.1	2.11	1.39E-05	1.35E-04
2040	ENSG00000166228.8	PCBD1	0.58	1.40E-05	1.36E-04
2041	ENSG00000183060.15	LYSMD4	0.57	1.40E-05	1.36E-04
2042	ENSG00000141295.13	SCRN2	0.55	1.41E-05	1.37E-04
2043	ENSG00000149311.18	ATM	-0.44	1.41E-05	1.37E-04
2044	ENSG00000130429.14	ARPC1B	-0.63	1.42E-05	1.38E-04
2045	ENSG00000135414.9	GDF11	0.67	1.42E-05	1.38E-04
2046	ENSG00000261071.1	AL441883.1	4.00	1.43E-05	1.38E-04
2047	ENSG00000256269.8	HMBS	0.54	1.43E-05	1.38E-04

2048	ENSG00000197312.11	DDI2	0.50	1.43E-05	1.39E-04
2049	ENSG00000115325.13	DOK1	-0.71	1.44E-05	1.40E-04
2050	ENSG00000213619.9	NDUFS3	0.43	1.45E-05	1.40E-04
2051	ENSG00000214706.10	IFRD2	0.54	1.45E-05	1.41E-04
2052	ENSG00000169020.9	ATP5ME	0.45	1.46E-05	1.42E-04
2053	ENSG00000144827.8	ABHD10	0.45	1.46E-05	1.42E-04
2054	ENSG00000151117.8	TMEM86A	1.24	1.47E-05	1.42E-04
2055	ENSG00000197496.5	SLC2A10	-0.50	1.48E-05	1.43E-04
2056	ENSG00000179406.7	LINC00174	0.78	1.49E-05	1.43E-04
2057	ENSG00000140092.14	FBLN5	-0.51	1.49E-05	1.43E-04
2058	ENSG00000099139.13	PCSK5	0.70	1.49E-05	1.43E-04
2059	ENSG00000144233.9	AMMECR1L	0.44	1.49E-05	1.44E-04
2060	ENSG00000131174.5	COX7B	0.39	1.50E-05	1.45E-04
2061	ENSG00000159063.12	ALG8	0.46	1.51E-05	1.45E-04
2062	ENSG00000131368.7	MRPS25	0.44	1.51E-05	1.45E-04
2063	ENSG00000263429.3	TMEM238L	6.52	1.51E-05	1.45E-04
2064	ENSG00000179912.20	R3HDM2	-0.48	1.51E-05	1.45E-04
2065	ENSG00000204316.12	MRPL38	0.61	1.54E-05	1.48E-04
2066	ENSG00000135919.12	SERPINE2	-0.69	1.54E-05	1.48E-04
2067	ENSG00000179399.14	GPC5	6.38	1.54E-05	1.48E-04
2068	ENSG00000013288.8	MAN2B2	-0.46	1.54E-05	1.48E-04
2069	ENSG00000276547.1	PCDHGB5	-0.55	1.54E-05	1.48E-04
2070	ENSG00000156521.13	TYSND1	0.49	1.55E-05	1.48E-04
2071	ENSG00000124216.3	SNAI1	-1.13	1.55E-05	1.48E-04
2072	ENSG00000140459.17	CYP11A1	1.72	1.55E-05	1.48E-04
2073	ENSG00000137806.8	NDUFAF1	0.52	1.55E-05	1.49E-04
2074	ENSG00000039523.19	RIPOR1	-0.52	1.56E-05	1.49E-04
2075	ENSG00000124198.8	ARFGEF2	0.39	1.56E-05	1.49E-04
2076	ENSG00000115840.13	SLC25A12	-0.58	1.57E-05	1.50E-04
2077	ENSG00000112941.13	TENT4A	0.44	1.58E-05	1.51E-04
2078	ENSG00000145391.13	SETD7	-0.40	1.60E-05	1.53E-04
2079	ENSG00000257354.2	AC048341.1	-0.73	1.61E-05	1.54E-04
2080	ENSG00000285982.1	AC012213.5	-8.28	1.62E-05	1.54E-04
2081	ENSG00000159228.12	CBR1	-0.52	1.64E-05	1.57E-04
2082	ENSG00000150403.17	TMCO3	-0.43	1.64E-05	1.57E-04
2083	ENSG00000137831.14	UACA	-0.62	1.67E-05	1.59E-04
2084	ENSG00000198399.14	ITSN2	0.44	1.68E-05	1.60E-04
2085	ENSG00000157240.3	FZD1	-0.65	1.68E-05	1.60E-04
2086	ENSG00000137275.13	RIPK1	0.48	1.69E-05	1.61E-04
2087	ENSG00000169230.9	PRELID1	0.46	1.69E-05	1.61E-04
2088	ENSG00000120690.15	ELF1	-0.50	1.69E-05	1.61E-04
2089	ENSG00000096433.10	ITPR3	-0.66	1.69E-05	1.61E-04
2090	ENSG00000055950.16	MRPL43	0.53	1.70E-05	1.62E-04
2091	ENSG00000124172.9	ATP5F1E	0.40	1.70E-05	1.62E-04
2092	ENSG00000147454.13	SLC25A37	-0.58	1.72E-05	1.63E-04
2093	ENSG00000162413.16	KLHL21	0.44	1.73E-05	1.64E-04
2094	ENSG00000145779.7	TNFAIP8	-0.50	1.74E-05	1.65E-04
2095	ENSG00000182551.13	ADI1	-0.44	1.77E-05	1.68E-04
2096	ENSG00000106333.12	PCOLCE	-0.54	1.77E-05	1.68E-04
2097	ENSG00000122884.12	P4HA1	-0.58	1.77E-05	1.68E-04

2098	ENSG00000167074.14	TEF	0.56	1.77E-05	1.68E-04
2099	ENSG00000128039.10	SRD5A3	0.53	1.77E-05	1.68E-04
2100	ENSG00000164574.15	GALNT10	-0.53	1.78E-05	1.68E-04
2101	ENSG00000088899.15	LZTS3	0.68	1.79E-05	1.70E-04
2102	ENSG00000214415.3	GNAT3	3.60	1.83E-05	1.72E-04
2103	ENSG00000119283.15	TRIM67	1.65	1.83E-05	1.72E-04
2104	ENSG00000010404.17	IDS	-0.51	1.83E-05	1.73E-04
2105	ENSG00000104267.9	CA2	-1.66	1.86E-05	1.75E-04
2106	ENSG00000091157.13	WDR7	0.47	1.86E-05	1.76E-04
2107	ENSG00000116717.12	GADD45A	0.63	1.88E-05	1.77E-04
2108	ENSG00000214367.7	HAUS3	0.66	1.89E-05	1.78E-04
2109	ENSG00000112146.16	FBXO9	0.45	1.90E-05	1.78E-04
2110	ENSG00000123080.10	CDKN2C	-0.39	1.95E-05	1.83E-04
2111	ENSG00000108559.11	NUP88	0.50	1.96E-05	1.84E-04
2112	ENSG00000274602.4	PI4KAP1	0.80	1.97E-05	1.85E-04
2113	ENSG00000080200.9	CRYBG3	-0.51	1.98E-05	1.86E-04
2114	ENSG00000147010.17	SH3KBP1	-0.53	1.98E-05	1.86E-04
2115	ENSG00000196535.16	MYO18A	-0.64	1.99E-05	1.86E-04
2116	ENSG00000245556.2	SCAMP1-AS1	0.82	1.99E-05	1.86E-04
2117	ENSG00000078319.9	PMS2P1	0.72	1.99E-05	1.86E-04
2118	ENSG00000106628.10	POLD2	0.52	2.00E-05	1.88E-04
2119	ENSG00000154640.14	BTG3	0.63	2.02E-05	1.90E-04
2120	ENSG00000154723.12	ATP5PF	0.45	2.03E-05	1.90E-04
2121	ENSG00000222009.8	BTBD19	-0.67	2.04E-05	1.91E-04
2122	ENSG00000168502.17	MTCL1	-0.57	2.06E-05	1.92E-04
2123	ENSG00000156017.12	CARNMT1	0.64	2.08E-05	1.94E-04
2124	ENSG00000138653.9	NDST4	2.50	2.09E-05	1.95E-04
2125	ENSG00000110274.15	CEP164	-0.65	2.09E-05	1.95E-04
2126	ENSG00000010803.16	SCMH1	0.46	2.09E-05	1.95E-04
2127	ENSG00000006534.15	ALDH3B1	-0.92	2.10E-05	1.95E-04
2128	ENSG00000067992.13	PDK3	1.48	2.10E-05	1.96E-04
2129	ENSG00000165219.21	GAPVD1	0.38	2.15E-05	2.00E-04
2130	ENSG00000169083.16	AR	-0.49	2.15E-05	2.00E-04
2131	ENSG00000145506.13	NKD2	-1.63	2.15E-05	2.00E-04
2132	ENSG00000168077.13	SCARA3	-0.84	2.15E-05	2.01E-04
2133	ENSG00000178764.7	ZHX2	-0.61	2.16E-05	2.01E-04
2134	ENSG00000285768.1	AC232323.2	2.34	2.17E-05	2.02E-04
2135	ENSG00000115896.15	PLCL1	-0.78	2.17E-05	2.02E-04
2136	ENSG00000219665.8	ZNF433-AS1	0.69	2.18E-05	2.02E-04
2137	ENSG00000101463.5	SYNDIG1	-1.20	2.18E-05	2.03E-04
2138	ENSG00000167186.10	COQ7	0.56	2.22E-05	2.06E-04
2139	ENSG00000166902.4	MRPL16	0.49	2.22E-05	2.06E-04
2140	ENSG00000240438.2	OFD1P5Y	2.00	2.27E-05	2.11E-04
2141	ENSG00000087116.15	ADAMTS2	-0.45	2.28E-05	2.11E-04
2142	ENSG00000014216.15	CAPN1	-0.52	2.28E-05	2.11E-04
2143	ENSG00000183426.16	NPIPA1	-0.44	2.29E-05	2.12E-04
2144	ENSG00000243279.3	PRAF2	-0.61	2.31E-05	2.14E-04
2145	ENSG00000136270.13	TBRG4	0.50	2.34E-05	2.16E-04
2146	ENSG00000213859.5	KCTD11	-0.52	2.34E-05	2.17E-04
2147	ENSG00000153132.12	CLGN	0.65	2.35E-05	2.17E-04

2148	ENSG00000141736.13	ERBB2	-0.51	2.38E-05	2.20E-04
2149	ENSG00000196814.14	MVB12B	0.65	2.40E-05	2.21E-04
2150	ENSG00000154258.16	ABCA9	-0.96	2.42E-05	2.23E-04
2151	ENSG00000116774.11	OLFML3	-0.65	2.42E-05	2.23E-04
2152	ENSG00000096063.15	SRPK1	0.58	2.43E-05	2.24E-04
2153	ENSG00000114770.16	ABCC5	-0.55	2.44E-05	2.25E-04
2154	ENSG00000019991.16	HGF	-1.03	2.45E-05	2.25E-04
2155	ENSG00000232395.1	AL138830.2	6.49	2.46E-05	2.26E-04
2156	ENSG00000243789.10	JMJD7	0.86	2.46E-05	2.26E-04
2157	ENSG00000067646.11	ZFY	-0.53	2.53E-05	2.33E-04
2158	ENSG00000244405.7	ETV5	-0.97	2.53E-05	2.33E-04
2159	ENSG00000100568.10	VTI1B	0.39	2.54E-05	2.33E-04
2160	ENSG00000278935.1	AC087386.2	1.68	2.54E-05	2.33E-04
2161	ENSG00000198133.8	TMEM229B	-1.53	2.56E-05	2.35E-04
2162	ENSG00000145246.13	ATP10D	-0.50	2.57E-05	2.36E-04
2163	ENSG00000196628.16	TCF4	-0.43	2.58E-05	2.36E-04
2164	ENSG00000232388.4	SMIM26	0.51	2.58E-05	2.37E-04
2165	ENSG00000101670.11	LIPG	-0.96	2.60E-05	2.39E-04
2166	ENSG00000156298.12	TSPAN7	2.10	2.62E-05	2.40E-04
2167	ENSG00000213199.7	ASIC3	-1.35	2.62E-05	2.40E-04
2168	ENSG00000078053.16	AMPH	0.67	2.65E-05	2.42E-04
2169	ENSG00000115904.12	SOS1	-0.43	2.68E-05	2.45E-04
2170	ENSG00000130529.15	TRPM4	-0.72	2.69E-05	2.46E-04
2171	ENSG00000136930.12	PSMB7	0.38	2.69E-05	2.46E-04
2172	ENSG00000170315.13	UBB	0.37	2.69E-05	2.46E-04
2173	ENSG00000198712.1	MT-CO2	0.35	2.70E-05	2.46E-04
2174	ENSG00000153560.11	UBP1	0.40	2.70E-05	2.47E-04
2175	ENSG00000257337.6	AC068888.1	-1.03	2.73E-05	2.49E-04
2176	ENSG00000117305.14	HMGCL	0.46	2.73E-05	2.49E-04
2177	ENSG00000099377.13	HSD3B7	-0.74	2.74E-05	2.49E-04
2178	ENSG00000180346.3	TIGD2	0.59	2.74E-05	2.50E-04
2179	ENSG00000176209.11	SMIM19	0.48	2.75E-05	2.50E-04
2180	ENSG00000163412.12	EIF4E3	0.53	2.76E-05	2.51E-04
2181	ENSG00000246763.6	RGMB-AS1	-0.95	2.76E-05	2.51E-04
2182	ENSG00000131370.15	SH3BP5	0.48	2.76E-05	2.51E-04
2183	ENSG00000092607.14	TBX15	0.48	2.77E-05	2.52E-04
2184	ENSG00000178425.13	NT5DC1	0.49	2.77E-05	2.52E-04
2185	ENSG00000100242.15	SUN2	-0.53	2.77E-05	2.52E-04
2186	ENSG00000129255.15	MPDU1	0.40	2.79E-05	2.53E-04
2187	ENSG00000005249.12	PRKAR2B	0.59	2.81E-05	2.55E-04
2188	ENSG00000169976.6	SF3B5	0.53	2.82E-05	2.55E-04
2189	ENSG00000163428.3	LRRC58	0.46	2.83E-05	2.57E-04
2190	ENSG00000174808.11	BTC	1.32	2.85E-05	2.58E-04
2191	ENSG00000176485.11	PLA2G16	0.36	2.85E-05	2.58E-04
2192	ENSG00000178301.3	AQP11	0.75	2.86E-05	2.59E-04
2193	ENSG00000114302.15	PRKAR2A	0.43	2.86E-05	2.59E-04
2194	ENSG00000141026.5	MED9	0.56	2.89E-05	2.61E-04
2195	ENSG00000166073.10	GPR176	-0.60	2.89E-05	2.61E-04
2196	ENSG00000164300.16	SERINC5	0.52	2.90E-05	2.62E-04
2197	ENSG00000177465.4	ACOT4	1.06	2.92E-05	2.64E-04

2198	ENSG00000139971.15	ARMH4	-0.78	2.92E-05	2.64E-04
2199	ENSG00000136908.17	DPM2	0.49	2.93E-05	2.64E-04
2200	ENSG00000176406.22	RIMS2	-1.17	2.93E-05	2.65E-04
2201	ENSG00000010072.15	SPRTN	0.53	2.94E-05	2.65E-04
2202	ENSG00000104723.20	TUSC3	-0.60	2.94E-05	2.65E-04
2203	ENSG00000156265.15	MAP3K7CL	-0.86	2.94E-05	2.65E-04
2204	ENSG00000123992.19	DNPEP	0.44	2.94E-05	2.65E-04
2205	ENSG00000179091.4	CYC1	0.46	2.95E-05	2.65E-04
2206	ENSG00000196975.15	ANXA4	-0.59	2.96E-05	2.66E-04
2207	ENSG00000155254.12	MARVELD1	-0.46	2.97E-05	2.67E-04
2208	ENSG00000143624.13	INTS3	-0.45	2.97E-05	2.67E-04
2209	ENSG00000205544.3	TMEM256	0.56	2.98E-05	2.67E-04
2210	ENSG00000140416.20	TPM1	-0.58	3.00E-05	2.69E-04
2211	ENSG00000184349.12	EFNA5	-0.58	3.01E-05	2.70E-04
2212	ENSG00000102763.17	VWA8	0.40	3.02E-05	2.71E-04
2213	ENSG00000166741.7	NNMT	-0.74	3.03E-05	2.72E-04
2214	ENSG00000092621.12	PHGDH	-0.48	3.04E-05	2.73E-04
2215	ENSG00000154856.12	APCDD1	-1.06	3.06E-05	2.74E-04
2216	ENSG00000241015.2	TPM3P9	0.87	3.07E-05	2.75E-04
2217	ENSG00000052795.12	FNIP2	0.42	3.07E-05	2.75E-04
2218	ENSG00000166477.12	LEO1	-0.45	3.07E-05	2.75E-04
2219	ENSG00000234840.1	LINC01239	-0.90	3.08E-05	2.75E-04
2220	ENSG00000162989.4	KCNJ3	-1.50	3.10E-05	2.77E-04
2221	ENSG00000160145.15	KALRN	0.64	3.11E-05	2.77E-04
2222	ENSG00000059804.15	SLC2A3	-0.74	3.11E-05	2.78E-04
2223	ENSG00000140990.14	NDUFB10	0.41	3.11E-05	2.78E-04
2224	ENSG00000154146.12	NRGN	-2.81	3.12E-05	2.78E-04
2225	ENSG00000117569.18	PTBP2	-0.61	3.17E-05	2.83E-04
2226	ENSG00000170873.18	MTSS1	-0.68	3.20E-05	2.85E-04
2227	ENSG00000250021.7	C15orf38-AP3S2	0.45	3.22E-05	2.87E-04
2228	ENSG00000085491.16	SLC25A24	-0.53	3.23E-05	2.88E-04
2229	ENSG00000186832.8	KRT16	0.86	3.23E-05	2.88E-04
2230	ENSG00000001561.6	ENPP4	1.70	3.24E-05	2.88E-04
2231	ENSG00000269976.1	AC012065.3	2.10	3.30E-05	2.94E-04
2232	ENSG00000122862.4	SRGN	-1.35	3.32E-05	2.95E-04
2233	ENSG00000172845.14	SP3	-0.42	3.32E-05	2.95E-04
2234	ENSG00000117602.11	RCAN3	-0.84	3.33E-05	2.95E-04
2235	ENSG00000156463.17	SH3RF2	-1.30	3.35E-05	2.97E-04
2236	ENSG00000169435.13	RASSF6	1.41	3.39E-05	3.00E-04
2237	ENSG00000260537.2	AC012184.2	0.86	3.39E-05	3.01E-04
2238	ENSG00000147123.10	NDUFB11	0.45	3.39E-05	3.01E-04
2239	ENSG00000152465.17	NMT2	0.42	3.39E-05	3.01E-04
2240	ENSG00000172037.13	LAMB2	-0.52	3.40E-05	3.01E-04
2241	ENSG00000131378.13	RFTN1	-0.38	3.43E-05	3.04E-04
2242	ENSG00000110200.8	ANAPC15	0.51	3.48E-05	3.08E-04
2243	ENSG00000115170.13	ACVR1	-0.57	3.48E-05	3.08E-04
2244	ENSG00000102897.9	LYRM1	0.62	3.48E-05	3.08E-04
2245	ENSG00000145730.20	PAM	-0.51	3.48E-05	3.08E-04
2246	ENSG00000256977.12	LIMS3	0.76	3.50E-05	3.09E-04
2247	ENSG00000168101.14	NUDT16L1	0.59	3.50E-05	3.10E-04

2248	ENSG00000118402.5	ELOVL4	0.96	3.51E-05	3.10E-04
2249	ENSG00000165283.15	STOML2	0.42	3.53E-05	3.11E-04
2250	ENSG00000115641.18	FHL2	-0.70	3.55E-05	3.13E-04
2251	ENSG00000171988.18	JMJD1C	-0.43	3.57E-05	3.15E-04
2252	ENSG00000204217.13	BMPR2	-0.39	3.58E-05	3.15E-04
2253	ENSG00000253873.5	PCDHGA11	-0.58	3.58E-05	3.15E-04
2254	ENSG00000249695.6	AC026369.1	1.86	3.59E-05	3.16E-04
2255	ENSG00000138395.14	CDK15	0.80	3.59E-05	3.16E-04
2256	ENSG00000234535.1	AL161719.1	3.16	3.63E-05	3.19E-04
2257	ENSG00000259883.1	EHD4-AS1	2.59	3.68E-05	3.24E-04
2258	ENSG00000235081.1	AC245052.3	2.92	3.70E-05	3.25E-04
2259	ENSG00000198467.14	TPM2	-0.51	3.70E-05	3.25E-04
2260	ENSG00000177150.12	FAM210A	0.53	3.71E-05	3.26E-04
2261	ENSG00000167600.13	CYP2S1	3.65	3.73E-05	3.27E-04
2262	ENSG00000105270.14	CLIP3	0.58	3.74E-05	3.28E-04
2263	ENSG00000273680.1	AC009318.2	1.30	3.75E-05	3.28E-04
2264	ENSG00000248583.1	AC119751.3	-2.34	3.74E-05	3.28E-04
2265	ENSG00000119280.16	C1orf198	-0.47	3.75E-05	3.28E-04
2266	ENSG00000095794.19	CREM	0.57	3.75E-05	3.29E-04
2267	ENSG00000136161.12	RCBTB2	0.46	3.75E-05	3.29E-04
2268	ENSG00000183741.11	CBX6	-0.41	3.77E-05	3.30E-04
2269	ENSG00000144668.11	ITGA9	-0.87	3.79E-05	3.32E-04
2270	ENSG00000196268.11	ZNF493	0.59	3.80E-05	3.32E-04
2271	ENSG00000197696.9	NMB	0.61	3.82E-05	3.33E-04
2272	ENSG00000185477.4	GPRIN3	0.94	3.83E-05	3.34E-04
2273	ENSG00000166295.8	ANAPC16	0.43	3.86E-05	3.37E-04
2274	ENSG00000250731.1	TPM3P6	3.33	3.87E-05	3.38E-04
2275	ENSG00000233429.9	HOTAIRM1	1.03	3.87E-05	3.38E-04
2276	ENSG00000175567.8	UCP2	0.69	3.87E-05	3.38E-04
2277	ENSG00000104853.15	CLPTM1	0.41	3.88E-05	3.38E-04
2278	ENSG00000013619.13	MAMLD1	-0.54	3.88E-05	3.38E-04
2279	ENSG00000114251.14	WNT5A	-0.51	3.90E-05	3.39E-04
2280	ENSG00000100523.14	DDHD1	0.46	3.91E-05	3.40E-04
2281	ENSG00000188739.14	RBM34	0.57	3.92E-05	3.41E-04
2282	ENSG00000173905.8	GOLIM4	-0.50	3.92E-05	3.41E-04
2283	ENSG00000133789.14	SWAP70	-0.55	3.93E-05	3.41E-04
2284	ENSG00000122741.15	DCAF10	0.41	3.94E-05	3.42E-04
2285	ENSG00000123609.10	NMI	-0.87	3.96E-05	3.44E-04
2286	ENSG00000137413.15	TAF8	0.54	3.96E-05	3.44E-04
2287	ENSG00000184009.11	ACTG1	-0.48	3.98E-05	3.45E-04
2288	ENSG00000119772.16	DNMT3A	-0.55	3.98E-05	3.45E-04
2289	ENSG00000129480.12	DTD2	0.50	3.99E-05	3.46E-04
2290	ENSG00000003509.15	NDUFAF7	0.47	3.99E-05	3.46E-04
2291	ENSG00000223749.9	MIR503HG	-0.88	4.03E-05	3.49E-04
2292	ENSG00000102385.12	DRP2	1.00	4.04E-05	3.50E-04
2293	ENSG00000089472.16	HEPH	-0.49	4.04E-05	3.50E-04
2294	ENSG00000119681.11	LTBP2	-0.73	4.04E-05	3.50E-04
2295	ENSG00000070214.15	SLC44A1	-0.62	4.06E-05	3.51E-04
2296	ENSG00000025772.7	TOMM34	0.49	4.08E-05	3.53E-04
2297	ENSG00000079459.12	FDFT1	0.39	4.09E-05	3.54E-04

2298	ENSG00000181751.9	C5orf30	-0.58	4.09E-05	3.54E-04
2299	ENSG00000253276.2	CCDC71L	-0.50	4.11E-05	3.55E-04
2300	ENSG00000244198.6	AC004889.1	0.88	4.12E-05	3.55E-04
2301	ENSG00000187605.15	TET3	-0.56	4.13E-05	3.56E-04
2302	ENSG00000196704.11	AMZ2	0.43	4.14E-05	3.57E-04
2303	ENSG00000141756.18	FKBP10	-0.65	4.17E-05	3.59E-04
2304	ENSG00000129226.13	CD68	1.42	4.17E-05	3.59E-04
2305	ENSG00000166801.15	FAM111A	-0.72	4.18E-05	3.60E-04
2306	ENSG00000205208.4	C4orf46	0.58	4.18E-05	3.60E-04
2307	ENSG00000108187.15	PBLD	0.58	4.21E-05	3.62E-04
2308	ENSG00000164694.16	FNDC1	-0.77	4.21E-05	3.62E-04
2309	ENSG00000006128.11	TAC1	4.78	4.25E-05	3.65E-04
2310	ENSG00000118564.14	FBXL5	0.37	4.26E-05	3.66E-04
2311	ENSG00000105607.12	GCDH	0.52	4.29E-05	3.69E-04
2312	ENSG00000251632.1	LINC02172	-2.07	4.33E-05	3.72E-04
2313	ENSG00000131007.9	TTTY9B	1.27	4.36E-05	3.74E-04
2314	ENSG00000171943.11	SRGAP2C	-0.47	4.37E-05	3.75E-04
2315	ENSG00000198734.10	F5	2.02	4.38E-05	3.75E-04
2316	ENSG00000225889.7	AC012368.1	1.27	4.40E-05	3.77E-04
2317	ENSG00000162976.12	PQLC3	-0.52	4.43E-05	3.79E-04
2318	ENSG00000109501.13	WFS1	0.54	4.47E-05	3.83E-04
2319	ENSG00000105989.9	WNT2	-0.62	4.49E-05	3.85E-04
2320	ENSG00000170542.5	SERPINB9	-0.69	4.50E-05	3.85E-04
2321	ENSG00000166136.15	NDUFB8	0.42	4.52E-05	3.87E-04
2322	ENSG00000101911.12	PRPS2	0.65	4.54E-05	3.88E-04
2323	ENSG00000234362.5	LINC01914	1.75	4.54E-05	3.88E-04
2324	ENSG00000213516.9	RBMXL1	0.70	4.57E-05	3.90E-04
2325	ENSG00000091831.23	ESR1	1.16	4.62E-05	3.94E-04
2326	ENSG00000114857.17	NKTR	-0.38	4.61E-05	3.94E-04
2327	ENSG00000228526.7	MIR34AHG	-0.52	4.62E-05	3.94E-04
2328	ENSG00000247993.2	FOXD1-AS1	0.82	4.64E-05	3.95E-04
2329	ENSG00000119711.12	ALDH6A1	-0.51	4.64E-05	3.95E-04
2330	ENSG00000167549.18	CORO6	-0.91	4.64E-05	3.95E-04
2331	ENSG00000197860.9	SGTB	-0.69	4.66E-05	3.96E-04
2332	ENSG00000283189.2	AC104452.1	-1.08	4.68E-05	3.98E-04
2333	ENSG00000273906.1	AC011297.1	0.85	4.73E-05	4.02E-04
2334	ENSG00000162437.14	RAVER2	0.52	4.74E-05	4.03E-04
2335	ENSG00000163071.10	SPATA18	-0.66	4.74E-05	4.03E-04
2336	ENSG00000111676.14	ATN1	-0.45	4.76E-05	4.04E-04
2337	ENSG00000189058.8	APOD	-0.86	4.76E-05	4.04E-04
2338	ENSG00000107438.8	PDLIM1	-0.47	4.77E-05	4.05E-04
2339	ENSG00000123104.11	ITPR2	0.41	4.80E-05	4.07E-04
2340	ENSG00000237238.3	BMS1P10	0.92	4.80E-05	4.07E-04
2341	ENSG00000107281.9	NPDC1	-0.84	4.83E-05	4.09E-04
2342	ENSG00000166435.15	XRRA1	0.64	4.83E-05	4.10E-04
2343	ENSG00000198478.7	SH3BGRL2	0.84	4.85E-05	4.10E-04
2344	ENSG00000166250.11	CLMP	0.39	4.86E-05	4.11E-04
2345	ENSG00000116871.15	MAP7D1	-0.54	4.87E-05	4.12E-04
2346	ENSG00000184384.13	MAML2	-0.42	4.89E-05	4.13E-04
2347	ENSG00000123610.4	TNFAIP6	-1.00	4.91E-05	4.16E-04

2348	ENSG00000050165.17	DKK3	-0.61	4.95E-05	4.19E-04
2349	ENSG00000106799.12	TGFBR1	-0.38	4.96E-05	4.19E-04
2350	ENSG00000127990.17	SGCE	-0.45	4.97E-05	4.20E-04
2351	ENSG00000167112.9	TRUB2	0.43	4.99E-05	4.21E-04
2352	ENSG00000106236.3	NPTX2	2.38	5.01E-05	4.23E-04
2353	ENSG00000179520.10	SLC17A8	3.05	5.03E-05	4.24E-04
2354	ENSG00000163933.9	RFT1	0.52	5.03E-05	4.24E-04
2355	ENSG00000184898.6	RBM43	-0.63	5.04E-05	4.25E-04
2356	ENSG00000118898.15	PPL	-1.54	5.05E-05	4.26E-04
2357	ENSG00000135144.7	DTX1	0.66	5.07E-05	4.26E-04
2358	ENSG00000120156.20	TEK	-0.80	5.07E-05	4.26E-04
2359	ENSG00000159216.18	RUNX1	-0.57	5.07E-05	4.27E-04
2360	ENSG00000063854.12	HAGH	0.45	5.09E-05	4.28E-04
2361	ENSG00000138604.9	GLCE	-0.43	5.14E-05	4.32E-04
2362	ENSG00000109814.11	UGDH	-0.51	5.15E-05	4.33E-04
2363	ENSG00000257501.6	AC007424.1	1.90	5.18E-05	4.35E-04
2364	ENSG00000112977.15	DAP	-0.46	5.19E-05	4.36E-04
2365	ENSG00000236345.1	AL354719.2	-1.86	5.20E-05	4.36E-04
2366	ENSG00000187079.16	TEAD1	-0.35	5.22E-05	4.38E-04
2367	ENSG00000147535.16	PLPP5	-0.56	5.27E-05	4.42E-04
2368	ENSG00000171132.13	PRKCE	0.58	5.32E-05	4.46E-04
2369	ENSG00000111886.10	GABRR2	1.76	5.36E-05	4.49E-04
2370	ENSG00000150938.9	CRIM1	-0.46	5.37E-05	4.50E-04
2371	ENSG00000182195.7	LDOC1	-0.58	5.38E-05	4.50E-04
2372	ENSG00000138829.11	FBN2	-0.53	5.41E-05	4.53E-04
2373	ENSG00000088682.13	COQ9	0.40	5.41E-05	4.53E-04
2374	ENSG00000161281.10	COX7A1	-0.62	5.42E-05	4.53E-04
2375	ENSG00000262655.3	SPON1	1.88	5.43E-05	4.53E-04
2376	ENSG00000142156.14	COL6A1	-0.47	5.44E-05	4.55E-04
2377	ENSG00000214253.8	FIS1	0.41	5.46E-05	4.56E-04
2378	ENSG00000103365.15	GGA2	0.42	5.47E-05	4.57E-04
2379	ENSG00000104522.15	TSTA3	0.59	5.50E-05	4.59E-04
2380	ENSG00000106804.7	C5	-1.00	5.51E-05	4.59E-04
2381	ENSG00000168394.11	TAP1	0.49	5.53E-05	4.61E-04
2382	ENSG00000146066.2	HIGD2A	0.46	5.55E-05	4.62E-04
2383	ENSG00000130489.14	SCO2	0.61	5.61E-05	4.67E-04
2384	ENSG00000064225.12	ST3GAL6	0.60	5.61E-05	4.67E-04
2385	ENSG00000143437.20	ARNT	-0.43	5.61E-05	4.67E-04
2386	ENSG00000206549.13	PRSS50	3.66	5.62E-05	4.67E-04
2387	ENSG00000116001.15	TIA1	-0.42	5.63E-05	4.68E-04
2388	ENSG00000119685.19	TTLL5	0.52	5.69E-05	4.73E-04
2389	ENSG00000144029.11	MRPS5	0.41	5.73E-05	4.76E-04
2390	ENSG00000276600.4	RAB7B	-0.68	5.73E-05	4.76E-04
2391	ENSG00000076344.15	RGS11	-1.48	5.74E-05	4.76E-04
2392	ENSG00000130821.15	SLC6A8	0.48	5.74E-05	4.76E-04
2393	ENSG00000185278.15	ZBTB37	0.42	5.74E-05	4.76E-04
2394	ENSG00000211445.11	GPX3	0.81	5.76E-05	4.77E-04
2395	ENSG00000175662.17	TOM1L2	0.49	5.77E-05	4.78E-04
2396	ENSG00000164347.17	GFM2	0.43	5.80E-05	4.81E-04
2397	ENSG00000272734.1	ADIRF-AS1	0.92	5.83E-05	4.83E-04

2398	ENSG00000107736.20	CDH23	-0.97	5.86E-05	4.85E-04
2399	ENSG00000151640.12	DPYSL4	0.57	5.87E-05	4.85E-04
2400	ENSG00000143549.19	TPM3	-0.50	5.87E-05	4.85E-04
2401	ENSG00000149968.11	MMP3	-3.82	5.87E-05	4.85E-04
2402	ENSG00000189283.9	FHIT	0.90	5.89E-05	4.87E-04
2403	ENSG00000137038.7	DMAC1	0.45	5.90E-05	4.87E-04
2404	ENSG00000177508.11	IRX3	-0.88	5.90E-05	4.87E-04
2405	ENSG00000100412.15	ACO2	0.45	5.90E-05	4.87E-04
2406	ENSG00000189182.9	KRT77	1.53	5.91E-05	4.88E-04
2407	ENSG00000114270.17	COL7A1	-0.84	5.93E-05	4.89E-04
2408	ENSG00000259431.5	THTPA	0.51	5.93E-05	4.89E-04
2409	ENSG00000167702.12	KIFC2	-0.93	5.95E-05	4.90E-04
2410	ENSG00000196639.6	HRH1	-1.10	5.99E-05	4.93E-04
2411	ENSG00000173681.16	BCLAF3	-0.61	5.99E-05	4.93E-04
2412	ENSG00000169432.15	SCN9A	-0.60	6.00E-05	4.93E-04
2413	ENSG00000123612.15	ACVR1C	-0.58	6.02E-05	4.95E-04
2414	ENSG00000168172.8	HOOK3	0.45	6.04E-05	4.96E-04
2415	ENSG00000138175.8	ARL3	-0.59	6.04E-05	4.96E-04
2416	ENSG00000105928.14	GSDME	-0.53	6.09E-05	5.00E-04
2417	ENSG00000182481.8	KPNA2	-0.46	6.12E-05	5.02E-04
2418	ENSG00000125877.12	ITPA	0.45	6.12E-05	5.02E-04
2419	ENSG00000143196.4	DPT	-0.43	6.14E-05	5.04E-04
2420	ENSG00000143344.15	RGL1	-0.46	6.17E-05	5.06E-04
2421	ENSG00000143106.12	PSMA5	0.49	6.19E-05	5.08E-04
2422	ENSG00000122786.19	CALD1	-0.40	6.28E-05	5.15E-04
2423	ENSG00000184500.15	PROS1	-0.56	6.30E-05	5.16E-04
2424	ENSG00000139926.15	FRMD6	-0.59	6.32E-05	5.17E-04
2425	ENSG00000174483.19	BBS1	-0.67	6.38E-05	5.22E-04
2426	ENSG00000135269.17	TES	-0.52	6.43E-05	5.26E-04
2427	ENSG00000149260.16	CAPN5	0.56	6.46E-05	5.28E-04
2428	ENSG00000071564.14	TCF3	-0.57	6.46E-05	5.28E-04
2429	ENSG00000151388.10	ADAMTS12	0.37	6.47E-05	5.29E-04
2430	ENSG00000158850.14	B4GALT3	0.51	6.50E-05	5.31E-04
2431	ENSG00000123352.17	SPATS2	-0.58	6.52E-05	5.32E-04
2432	ENSG00000263956.6	NBPF11	0.48	6.54E-05	5.34E-04
2433	ENSG00000169231.13	THBS3	-0.66	6.55E-05	5.34E-04
2434	ENSG00000130997.16	POLN	0.83	6.56E-05	5.34E-04
2435	ENSG00000171492.14	LRRC8D	-0.48	6.60E-05	5.38E-04
2436	ENSG00000135318.11	NT5E	-0.68	6.61E-05	5.39E-04
2437	ENSG00000196313.11	POM121	0.41	6.62E-05	5.39E-04
2438	ENSG00000082512.14	TRAF5	-0.51	6.65E-05	5.41E-04
2439	ENSG00000197756.9	RPL37A	0.40	6.66E-05	5.42E-04
2440	ENSG00000183258.11	DDX41	0.42	6.66E-05	5.42E-04
2441	ENSG00000101407.12	TTI1	0.48	6.68E-05	5.43E-04
2442	ENSG00000196715.6	VKORC1L1	0.38	6.69E-05	5.44E-04
2443	ENSG00000213626.12	LBH	-0.49	6.72E-05	5.46E-04
2444	ENSG00000065150.18	IPO5	-0.39	6.72E-05	5.46E-04
2445	ENSG00000041357.15	PSMA4	0.43	6.73E-05	5.46E-04
2446	ENSG00000109685.17	NSD2	0.41	6.81E-05	5.53E-04
2447	ENSG00000159314.11	ARHGAP27	-1.25	6.82E-05	5.53E-04

2448	ENSG00000144366.15	GULP1	-0.58	6.83E-05	5.54E-04
2449	ENSG00000139946.9	PELI2	-0.84	6.85E-05	5.55E-04
2450	ENSG00000166923.10	GREM1	-0.38	6.89E-05	5.58E-04
2451	ENSG00000071282.11	LMCD1	-0.41	6.91E-05	5.60E-04
2452	ENSG00000149792.8	MRPL49	0.41	6.92E-05	5.60E-04
2453	ENSG00000132541.10	RIDA	0.50	6.93E-05	5.60E-04
2454	ENSG00000166797.10	CIAO2A	0.60	6.95E-05	5.62E-04
2455	ENSG00000143776.18	CDC42BPA	-0.38	7.00E-05	5.65E-04
2456	ENSG00000178033.5	CALHM5	-0.45	6.99E-05	5.65E-04
2457	ENSG00000007944.14	MYLIP	0.47	7.00E-05	5.66E-04
2458	ENSG00000187838.16	PLSCR3	-0.58	7.02E-05	5.67E-04
2459	ENSG00000258465.7	AL139011.2	3.37	7.03E-05	5.68E-04
2460	ENSG00000082556.10	OPRK1	0.81	7.04E-05	5.68E-04
2461	ENSG00000151690.14	MFSD6	-0.56	7.09E-05	5.72E-04
2462	ENSG00000165487.13	MICU2	0.43	7.11E-05	5.73E-04
2463	ENSG00000166750.9	SLFN5	-0.41	7.11E-05	5.73E-04
2464	ENSG00000164949.7	GEM	-0.52	7.12E-05	5.74E-04
2465	ENSG00000189007.15	ADAT2	0.70	7.14E-05	5.75E-04
2466	ENSG00000127418.14	FGFRL1	0.52	7.16E-05	5.76E-04
2467	ENSG00000077684.15	JADE1	-0.43	7.16E-05	5.76E-04
2468	ENSG00000051523.10	CYBA	-0.79	7.19E-05	5.78E-04
2469	ENSG00000099804.8	CDC34	0.55	7.26E-05	5.84E-04
2470	ENSG00000164050.12	PLXNB1	-0.68	7.27E-05	5.84E-04
2471	ENSG00000197256.10	KANK2	-0.38	7.29E-05	5.85E-04
2472	ENSG00000145012.13	LPP	-0.34	7.30E-05	5.86E-04
2473	ENSG00000240063.1	AC069431.1	1.91	7.30E-05	5.86E-04
2474	ENSG00000248487.8	ABHD14A	0.62	7.33E-05	5.88E-04
2475	ENSG00000103528.16	SYT17	0.76	7.40E-05	5.93E-04
2476	ENSG00000005884.17	ITGA3	-0.53	7.48E-05	5.99E-04
2477	ENSG00000148180.19	GSN	-0.45	7.55E-05	6.05E-04
2478	ENSG00000185345.20	PRKN	0.76	7.56E-05	6.06E-04
2479	ENSG00000188483.7	IER5L	-1.26	7.56E-05	6.06E-04
2480	ENSG00000235043.3	TECRP1	2.66	7.62E-05	6.10E-04
2481	ENSG00000165655.16	ZNF503	-0.65	7.64E-05	6.11E-04
2482	ENSG00000164116.16	GUCY1A1	2.32	7.65E-05	6.11E-04
2483	ENSG00000167397.14	VKORC1	-0.52	7.65E-05	6.11E-04
2484	ENSG00000188603.19	CLN3	0.51	7.66E-05	6.12E-04
2485	ENSG00000125375.14	ATP5S	0.45	7.66E-05	6.12E-04
2486	ENSG00000176046.8	NUPR1	0.55	7.68E-05	6.13E-04
2487	ENSG00000176340.3	COX8A	0.50	7.71E-05	6.15E-04
2488	ENSG00000177885.14	GRB2	0.33	7.81E-05	6.23E-04
2489	ENSG00000237797.1	AL161935.3	-5.48	7.84E-05	6.25E-04
2490	ENSG00000172264.17	MACROD2	0.64	7.91E-05	6.30E-04
2491	ENSG00000132635.16	PCED1A	0.57	7.91E-05	6.30E-04
2492	ENSG00000183671.12	GPR1	-0.93	7.92E-05	6.31E-04
2493	ENSG00000271425.7	NBPF10	-0.43	7.92E-05	6.31E-04
2494	ENSG00000225470.7	JPX	0.57	7.94E-05	6.32E-04
2495	ENSG00000133317.14	LGALS12	0.34	7.96E-05	6.33E-04
2496	ENSG00000164039.14	BDH2	-0.47	8.00E-05	6.36E-04
2497	ENSG00000162734.12	PEA15	-0.39	8.08E-05	6.42E-04

2498	ENSG00000100997.18	ABHD12	0.41	8.10E-05	6.43E-04
2499	ENSG00000176890.15	TYMS	-1.40	8.10E-05	6.43E-04
2500	ENSG00000109680.10	TBC1D19	-0.68	8.15E-05	6.47E-04
2501	ENSG00000095066.11	HOOK2	0.65	8.16E-05	6.48E-04
2502	ENSG00000150768.15	DLAT	0.49	8.17E-05	6.48E-04
2503	ENSG00000110906.12	KCTD10	-0.39	8.18E-05	6.48E-04
2504	ENSG00000137513.9	NARS2	0.48	8.21E-05	6.51E-04
2505	ENSG00000169242.11	EFNA1	1.33	8.23E-05	6.52E-04
2506	ENSG00000255100.1	AP003119.2	2.62	8.34E-05	6.60E-04
2507	ENSG00000175215.10	CTDSP2	-0.37	8.35E-05	6.61E-04
2508	ENSG00000126458.3	RRAS	-0.57	8.36E-05	6.61E-04
2509	ENSG00000099864.17	PALM	-0.67	8.36E-05	6.61E-04
2510	ENSG00000166326.6	TRIM44	0.34	8.38E-05	6.63E-04
2511	ENSG00000251493.4	FOXD1	0.56	8.39E-05	6.63E-04
2512	ENSG00000101181.17	MTG2	0.53	8.44E-05	6.67E-04
2513	ENSG00000131473.16	ACLY	0.58	8.45E-05	6.67E-04
2514	ENSG00000085832.16	EPS15	-0.40	8.52E-05	6.72E-04
2515	ENSG00000189337.16	KAZN	-0.54	8.55E-05	6.75E-04
2516	ENSG00000095303.15	PTGS1	-0.99	8.56E-05	6.75E-04
2517	ENSG00000153814.12	JAZF1	-0.46	8.59E-05	6.77E-04
2518	ENSG00000172987.12	HPSE2	2.20	8.61E-05	6.79E-04
2519	ENSG00000101365.20	IDH3B	0.39	8.68E-05	6.84E-04
2520	ENSG00000120327.6	PCDHB14	-0.84	8.69E-05	6.85E-04
2521	ENSG00000177426.20	TGIF1	-0.64	8.83E-05	6.95E-04
2522	ENSG00000089063.14	TMEM230	0.36	8.92E-05	7.02E-04
2523	ENSG00000197712.11	FAM114A1	-0.41	8.93E-05	7.02E-04
2524	ENSG00000278175.3	GLIDR	0.95	8.98E-05	7.06E-04
2525	ENSG00000217416.4	ISCA1P1	2.02	9.00E-05	7.07E-04
2526	ENSG00000177106.15	EPS8L2	-0.83	9.02E-05	7.09E-04
2527	ENSG00000178202.12	KDELC2	-0.52	9.04E-05	7.10E-04
2528	ENSG00000187997.11	C17orf99	1.41	9.06E-05	7.11E-04
2529	ENSG00000070718.11	AP3M2	-0.51	9.13E-05	7.16E-04
2530	ENSG00000100167.20	SEPT3	-2.30	9.19E-05	7.21E-04
2531	ENSG00000072682.18	P4HA2	-0.53	9.20E-05	7.21E-04
2532	ENSG00000256262.1	USP30-AS1	2.62	9.21E-05	7.22E-04
2533	ENSG00000072201.13	LNX1	-1.65	9.35E-05	7.32E-04
2534	ENSG00000136811.16	ODF2	-0.52	9.37E-05	7.34E-04
2535	ENSG00000083642.18	PDS5B	-0.38	9.40E-05	7.36E-04
2536	ENSG00000169375.15	SIN3A	-0.45	9.45E-05	7.39E-04
2537	ENSG00000182670.13	TTC3	-0.34	9.54E-05	7.47E-04
2538	ENSG00000112249.13	ASCC3	-0.46	9.57E-05	7.48E-04
2539	ENSG00000164692.17	COL1A2	-0.45	9.57E-05	7.48E-04
2540	ENSG00000219200.11	RNASEK	0.44	9.63E-05	7.52E-04
2541	ENSG00000154380.17	ENAH	-0.36	9.64E-05	7.53E-04
2542	ENSG00000185561.9	TLCD2	0.38	9.75E-05	7.61E-04
2543	ENSG00000271984.1	AL008726.1	1.42	9.76E-05	7.62E-04
2544	ENSG00000267519.6	AC020916.1	-0.66	9.81E-05	7.65E-04
2545	ENSG00000126603.8	GLIS2	-0.69	9.89E-05	7.72E-04
2546	ENSG00000175538.10	KCNE3	-0.80	9.97E-05	7.77E-04
2547	ENSG00000197646.7	PDCD1LG2	-1.03	9.98E-05	7.78E-04

2548	ENSG00000247626.4	MARS2	0.68	9.99E-05	7.78E-04
2549	ENSG00000186205.12	MARC1	0.42	1.01E-04	7.86E-04
2550	ENSG00000136883.14	KIF12	4.87	1.02E-04	7.90E-04
2551	ENSG00000113328.18	CCNG1	0.35	1.02E-04	7.92E-04
2552	ENSG00000143575.14	HAX1	0.39	1.03E-04	7.98E-04
2553	ENSG00000099256.18	PRTFDC1	-0.98	1.03E-04	8.02E-04
2554	ENSG00000259929.5	AC010601.1	2.27	1.03E-04	8.04E-04
2555	ENSG00000065357.19	DGKA	-0.58	1.04E-04	8.07E-04
2556	ENSG00000151503.12	NCAPD3	0.46	1.05E-04	8.16E-04
2557	ENSG00000147586.9	MRPS28	0.55	1.06E-04	8.23E-04
2558	ENSG00000137714.2	FDX1	0.50	1.07E-04	8.26E-04
2559	ENSG00000156030.13	ELMSAN1	-0.47	1.07E-04	8.27E-04
2560	ENSG00000076043.9	REXO2	-0.53	1.07E-04	8.28E-04
2561	ENSG00000203666.12	EFCAB2	0.58	1.07E-04	8.29E-04
2562	ENSG00000087253.12	LPCAT2	-0.69	1.07E-04	8.29E-04
2563	ENSG00000149577.15	SIDT2	-0.62	1.08E-04	8.38E-04
2564	ENSG00000203879.11	GDI1	-0.41	1.09E-04	8.41E-04
2565	ENSG00000285201.1	AC104454.2	3.13	1.09E-04	8.45E-04
2566	ENSG00000169116.11	PARM1	0.63	1.10E-04	8.52E-04
2567	ENSG00000132305.20	IMMT	0.33	1.10E-04	8.52E-04
2568	ENSG00000131143.8	COX4I1	0.32	1.12E-04	8.63E-04
2569	ENSG00000110871.14	COQ5	0.46	1.12E-04	8.64E-04
2570	ENSG00000114480.12	GBE1	0.37	1.12E-04	8.66E-04
2571	ENSG00000011465.16	DCN	-0.52	1.12E-04	8.67E-04
2572	ENSG00000149634.4	SPATA25	2.23	1.13E-04	8.72E-04
2573	ENSG00000168090.9	COPS6	0.42	1.13E-04	8.73E-04
2574	ENSG00000251201.8	TMED7-TICAM2	-1.31	1.13E-04	8.73E-04
2575	ENSG00000125148.6	MT2A	1.09	1.13E-04	8.74E-04
2576	ENSG00000283321.1	AC019117.2	-3.97	1.14E-04	8.77E-04
2577	ENSG00000102384.13	CENPI	0.73	1.14E-04	8.78E-04
2578	ENSG00000160789.19	LMNA	-0.48	1.14E-04	8.78E-04
2579	ENSG00000198668.10	CALM1	-0.37	1.14E-04	8.78E-04
2580	ENSG00000129038.15	LOXL1	-0.60	1.14E-04	8.78E-04
2581	ENSG00000139323.13	POC1B	0.59	1.15E-04	8.83E-04
2582	ENSG00000185483.11	ROR1	-0.58	1.15E-04	8.84E-04
2583	ENSG00000154589.6	LY96	-1.12	1.15E-04	8.85E-04
2584	ENSG00000233427.1	AL009181.1	2.44	1.16E-04	8.89E-04
2585	ENSG00000163393.12	SLC22A15	-0.87	1.16E-04	8.94E-04
2586	ENSG00000131016.16	AKAP12	-0.53	1.17E-04	8.95E-04
2587	ENSG00000124145.6	SDC4	-0.55	1.17E-04	8.96E-04
2588	ENSG00000064666.14	CNN2	-0.50	1.17E-04	8.96E-04
2589	ENSG00000153363.12	LINC00467	0.59	1.17E-04	8.98E-04
2590	ENSG00000184838.14	PRR16	-0.94	1.17E-04	8.99E-04
2591	ENSG00000138463.8	DIRC2	0.49	1.17E-04	8.99E-04
2592	ENSG00000174485.15	DENND4A	0.46	1.18E-04	9.03E-04
2593	ENSG00000127980.15	PEX1	0.46	1.18E-04	9.03E-04
2594	ENSG00000185305.10	ARL15	0.70	1.18E-04	9.04E-04
2595	ENSG00000162972.10	MAIP1	0.50	1.19E-04	9.07E-04
2596	ENSG00000130158.13	DOCK6	0.45	1.19E-04	9.07E-04
2597	ENSG00000131873.6	CHSY1	-0.45	1.19E-04	9.07E-04

2598	ENSG00000204381.11	LAYN	-0.55	1.19E-04	9.13E-04
2599	ENSG00000103472.10	RRN3P2	1.54	1.20E-04	9.15E-04
2600	ENSG00000152778.8	IFIT5	-0.50	1.20E-04	9.18E-04
2601	ENSG00000070785.16	EIF2B3	0.45	1.21E-04	9.22E-04
2602	ENSG00000162244.11	RPL29	0.45	1.22E-04	9.31E-04
2603	ENSG00000165704.14	HPRT1	0.48	1.22E-04	9.32E-04
2604	ENSG00000180818.4	HOXC10	-0.47	1.22E-04	9.32E-04
2605	ENSG00000084674.14	APOB	-0.64	1.22E-04	9.32E-04
2606	ENSG00000151414.14	NEK7	-0.53	1.22E-04	9.32E-04
2607	ENSG00000090376.10	IRAK3	-0.51	1.23E-04	9.34E-04
2608	ENSG00000102738.7	MRPS31	0.52	1.23E-04	9.38E-04
2609	ENSG00000186908.14	ZDHHC17	-0.37	1.24E-04	9.43E-04
2610	ENSG00000106927.11	AMBP	2.23	1.25E-04	9.49E-04
2611	ENSG00000119314.15	PTBP3	0.51	1.25E-04	9.49E-04
2612	ENSG00000165502.6	RPL36AL	0.40	1.25E-04	9.49E-04
2613	ENSG00000160218.12	TRAPPC10	0.39	1.25E-04	9.49E-04
2614	ENSG00000183621.15	ZNF438	-0.61	1.25E-04	9.49E-04
2615	ENSG00000069399.14	BCL3	-0.85	1.26E-04	9.55E-04
2616	ENSG00000110955.8	ATP5F1B	0.32	1.26E-04	9.58E-04
2617	ENSG00000176809.10	LRRC37A3	-0.54	1.26E-04	9.58E-04
2618	ENSG00000239697.10	TNFSF12	-0.78	1.26E-04	9.58E-04
2619	ENSG00000106733.20	NMRK1	0.66	1.27E-04	9.59E-04
2620	ENSG00000130684.13	ZNF337	-0.57	1.28E-04	9.66E-04
2621	ENSG00000204599.14	TRIM39	0.47	1.28E-04	9.67E-04
2622	ENSG00000274944.4	GJA9-MYCBP	1.56	1.28E-04	9.71E-04
2623	ENSG00000165023.6	DIRAS2	-0.66	1.29E-04	9.75E-04
2624	ENSG00000275004.3	ZNF280B	0.80	1.30E-04	9.85E-04
2625	ENSG00000258498.8	DIO3OS	1.79	1.30E-04	9.86E-04
2626	ENSG00000237732.9	AC010980.1	1.80	1.31E-04	9.90E-04
2627	ENSG00000141314.12	RHBDL3	2.06	1.31E-04	9.91E-04
2628	ENSG00000119862.12	LGALSL	-0.72	1.31E-04	9.93E-04
2629	ENSG00000179921.14	GPBAR1	1.43	1.32E-04	9.97E-04
2630	ENSG00000178209.15	PLEC	-0.65	1.33E-04	1.01E-03
2631	ENSG00000135835.11	KIAA1614	-0.69	1.34E-04	1.01E-03
2632	ENSG00000082482.13	KCNK2	-0.75	1.34E-04	1.01E-03
2633	ENSG00000049192.14	ADAMTS6	-0.84	1.35E-04	1.01E-03
2634	ENSG00000205221.12	VIT	-0.93	1.35E-04	1.02E-03
2635	ENSG00000048392.11	RRM2B	-0.43	1.35E-04	1.02E-03
2636	ENSG00000122591.11	FAM126A	-0.42	1.37E-04	1.03E-03
2637	ENSG00000257365.7	FNTB	0.46	1.37E-04	1.03E-03
2638	ENSG00000164211.12	STARD4	0.41	1.38E-04	1.04E-03
2639	ENSG00000231185.6	SPRY4-AS1	0.96	1.38E-04	1.04E-03
2640	ENSG00000122779.17	TRIM24	0.42	1.38E-04	1.04E-03
2641	ENSG00000148677.6	ANKRD1	-0.97	1.39E-04	1.04E-03
2642	ENSG00000170153.10	RNF150	-0.55	1.39E-04	1.04E-03
2643	ENSG00000185973.10	TMLHE	0.43	1.39E-04	1.05E-03
2644	ENSG00000164638.10	SLC29A4	0.57	1.40E-04	1.05E-03
2645	ENSG00000154122.13	ANKH	-0.37	1.40E-04	1.05E-03
2646	ENSG00000169504.14	CLIC4	-0.40	1.40E-04	1.05E-03
2647	ENSG00000166780.10	C16orf45	-0.62	1.40E-04	1.05E-03

2648	ENSG00000117682.16	DHDDS	0.33	1.41E-04	1.05E-03
2649	ENSG00000103187.7	COTL1	-0.62	1.41E-04	1.06E-03
2650	ENSG00000174175.16	SELP	1.63	1.41E-04	1.06E-03
2651	ENSG00000187955.11	COL14A1	-0.54	1.41E-04	1.06E-03
2652	ENSG00000092445.11	TYRO3	-0.57	1.42E-04	1.06E-03
2653	ENSG00000160602.13	NEK8	0.68	1.42E-04	1.06E-03
2654	ENSG00000237441.9	RGL2	-0.68	1.42E-04	1.06E-03
2655	ENSG00000132031.12	MATN3	-0.63	1.43E-04	1.07E-03
2656	ENSG00000185989.10	RASA3	0.39	1.44E-04	1.07E-03
2657	ENSG00000112186.11	CAP2	-0.49	1.44E-04	1.08E-03
2658	ENSG00000226741.1	LINC02554	1.74	1.45E-04	1.08E-03
2659	ENSG00000134020.7	PEBP4	-4.23	1.45E-04	1.08E-03
2660	ENSG00000113716.12	HMGXB3	0.39	1.46E-04	1.09E-03
2661	ENSG00000159596.6	TMEM69	0.41	1.46E-04	1.09E-03
2662	ENSG00000118985.15	ELL2	-0.44	1.46E-04	1.09E-03
2663	ENSG00000250241.5	AC105383.1	-1.71	1.46E-04	1.09E-03
2664	ENSG00000179152.19	TCAIM	0.45	1.47E-04	1.09E-03
2665	ENSG00000148123.14	PLPPR1	2.31	1.47E-04	1.10E-03
2666	ENSG00000189362.11	NEMP2	0.74	1.47E-04	1.10E-03
2667	ENSG00000253859.2	AC018616.1	3.32	1.48E-04	1.10E-03
2668	ENSG00000122912.14	SLC25A16	0.41	1.48E-04	1.10E-03
2669	ENSG00000134775.15	FHOD3	-0.56	1.49E-04	1.11E-03
2670	ENSG00000185090.14	MANEAL	0.91	1.49E-04	1.11E-03
2671	ENSG00000167468.16	GPX4	0.54	1.49E-04	1.11E-03
2672	ENSG00000054654.16	SYNE2	0.60	1.51E-04	1.12E-03
2673	ENSG00000174106.2	LEMD3	0.43	1.53E-04	1.13E-03
2674	ENSG00000107099.15	DOCK8	1.60	1.53E-04	1.13E-03
2675	ENSG00000263165.1	AC087292.1	-1.19	1.53E-04	1.13E-03
2676	ENSG00000267069.1	AP005264.1	3.02	1.53E-04	1.14E-03
2677	ENSG00000113248.5	PCDHB15	-1.25	1.53E-04	1.14E-03
2678	ENSG00000162630.5	B3GALT2	-0.90	1.54E-04	1.14E-03
2679	ENSG00000164885.12	CDK5	0.54	1.54E-04	1.14E-03
2680	ENSG00000108001.13	EBF3	-0.51	1.54E-04	1.14E-03
2681	ENSG00000188158.15	NHS	0.61	1.55E-04	1.15E-03
2682	ENSG00000110911.15	SLC11A2	-0.48	1.55E-04	1.15E-03
2683	ENSG00000108242.12	CYP2C18	2.77	1.56E-04	1.15E-03
2684	ENSG00000237846.1	AL773545.3	-1.08	1.56E-04	1.15E-03
2685	ENSG00000115392.11	FANCL	-0.53	1.57E-04	1.16E-03
2686	ENSG00000141696.12	P3H4	-0.64	1.57E-04	1.16E-03
2687	ENSG00000115762.16	PLEKHB2	0.46	1.57E-04	1.16E-03
2688	ENSG00000230487.7	PSMG3-AS1	-0.55	1.57E-04	1.16E-03
2689	ENSG00000111554.14	MDM1	0.49	1.58E-04	1.17E-03
2690	ENSG00000268205.1	AC005261.1	-0.54	1.58E-04	1.17E-03
2691	ENSG00000140945.16	CDH13	-0.71	1.59E-04	1.17E-03
2692	ENSG00000131495.8	NDUFA2	0.43	1.60E-04	1.18E-03
2693	ENSG00000163453.11	IGFBP7	-0.40	1.62E-04	1.20E-03
2694	ENSG00000090863.11	GLG1	-0.39	1.62E-04	1.20E-03
2695	ENSG00000107949.16	BCCIP	0.46	1.62E-04	1.20E-03
2696	ENSG00000138039.14	LHCGR	1.07	1.63E-04	1.20E-03
2697	ENSG00000249437.7	NAIP	-0.78	1.63E-04	1.20E-03

2698	ENSG00000169851.15	PCDH7	-0.46	1.63E-04	1.20E-03
2699	ENSG00000187792.4	ZNF70	0.67	1.64E-04	1.20E-03
2700	ENSG00000130702.15	LAMA5	-0.90	1.65E-04	1.21E-03
2701	ENSG00000159674.11	SPON2	-1.32	1.66E-04	1.22E-03
2702	ENSG00000145041.15	DCAF1	0.39	1.67E-04	1.22E-03
2703	ENSG00000196372.12	ASB13	0.62	1.68E-04	1.24E-03
2704	ENSG00000105968.18	H2AFV	0.39	1.69E-04	1.24E-03
2705	ENSG00000135218.18	CD36	0.42	1.70E-04	1.25E-03
2706	ENSG00000187800.13	PEAR1	-0.61	1.71E-04	1.25E-03
2707	ENSG00000187134.13	AKR1C1	-0.49	1.71E-04	1.26E-03
2708	ENSG00000165804.15	ZNF219	-0.67	1.71E-04	1.26E-03
2709	ENSG00000060237.16	WNK1	-0.34	1.72E-04	1.26E-03
2710	ENSG00000149925.18	ALDOA	0.56	1.72E-04	1.26E-03
2711	ENSG00000168300.13	PCMTD1	-0.40	1.75E-04	1.28E-03
2712	ENSG00000121897.14	LIAS	0.48	1.76E-04	1.29E-03
2713	ENSG00000056558.10	TRAF1	-0.82	1.76E-04	1.29E-03
2714	ENSG00000179119.14	SPTY2D1	0.40	1.77E-04	1.29E-03
2715	ENSG00000113594.9	LIFR	0.59	1.77E-04	1.29E-03
2716	ENSG00000249839.1	AC011330.1	1.34	1.77E-04	1.30E-03
2717	ENSG00000118849.9	RARRES1	2.46	1.78E-04	1.30E-03
2718	ENSG00000100100.12	PIK3IP1	-0.51	1.81E-04	1.32E-03
2719	ENSG00000168010.10	ATG16L2	-0.86	1.82E-04	1.33E-03
2720	ENSG00000165156.14	ZHX1	0.36	1.82E-04	1.33E-03
2721	ENSG00000099341.11	PSMD8	0.33	1.82E-04	1.33E-03
2722	ENSG00000124787.13	RPP40	0.70	1.83E-04	1.33E-03
2723	ENSG00000176641.10	RNF152	0.64	1.85E-04	1.35E-03
2724	ENSG00000196230.12	TUBB	-0.47	1.85E-04	1.35E-03
2725	ENSG00000100196.10	KDELR3	-0.47	1.85E-04	1.35E-03
2726	ENSG00000011405.13	PIK3C2A	0.35	1.86E-04	1.35E-03
2727	ENSG00000135365.15	PHF21A	-0.48	1.86E-04	1.36E-03
2728	ENSG00000116273.5	PHF13	0.44	1.87E-04	1.36E-03
2729	ENSG00000182986.13	ZNF320	-1.15	1.87E-04	1.36E-03
2730	ENSG00000162599.15	NFIA	-0.42	1.87E-04	1.36E-03
2731	ENSG00000112394.16	SLC16A10	1.64	1.87E-04	1.36E-03
2732	ENSG00000111275.12	ALDH2	-0.45	1.87E-04	1.36E-03
2733	ENSG00000147526.19	TACC1	-0.39	1.87E-04	1.36E-03
2734	ENSG00000183580.9	FBXL7	-0.56	1.88E-04	1.36E-03
2735	ENSG00000263731.1	AC145207.5	1.18	1.90E-04	1.38E-03
2736	ENSG00000137809.16	ITGA11	-0.44	1.90E-04	1.38E-03
2737	ENSG00000115155.17	OTOF	1.70	1.90E-04	1.38E-03
2738	ENSG00000155111.14	CDK19	-0.47	1.90E-04	1.38E-03
2739	ENSG00000196159.11	FAT4	-0.40	1.93E-04	1.40E-03
2740	ENSG00000120837.7	NFYB	-0.44	1.93E-04	1.40E-03
2741	ENSG00000227188.1	MGAT3-AS1	2.38	1.93E-04	1.40E-03
2742	ENSG00000118961.14	LDAH	0.42	1.93E-04	1.40E-03
2743	ENSG00000166333.13	ILK	-0.34	1.94E-04	1.40E-03
2744	ENSG00000105197.10	TIMM50	0.42	1.94E-04	1.40E-03
2745	ENSG00000068903.19	SIRT2	0.40	1.95E-04	1.41E-03
2746	ENSG00000136982.5	DSCC1	-1.24	1.95E-04	1.41E-03
2747	ENSG00000162783.10	IER5	-0.56	1.96E-04	1.42E-03

2748	ENSG00000162704.15	ARPC5	-0.42	1.97E-04	1.42E-03
2749	ENSG00000157456.7	CCNB2	-1.80	1.97E-04	1.42E-03
2750	ENSG00000112335.14	SNX3	0.35	1.98E-04	1.43E-03
2751	ENSG00000134769.21	DTNA	-1.20	1.98E-04	1.43E-03
2752	ENSG00000066279.17	ASPM	-1.55	1.98E-04	1.43E-03
2753	ENSG00000164008.15	C1orf50	0.54	1.99E-04	1.43E-03
2754	ENSG00000164342.12	TLR3	0.52	1.99E-04	1.43E-03
2755	ENSG00000143315.7	PIGM	0.42	1.99E-04	1.43E-03
2756	ENSG00000204262.12	COL5A2	-0.39	2.00E-04	1.44E-03
2757	ENSG00000178952.10	TUFM	0.37	2.00E-04	1.44E-03
2758	ENSG00000241886.1	AC112496.1	3.91	2.00E-04	1.44E-03
2759	ENSG00000100991.11	TRPC4AP	0.38	2.02E-04	1.45E-03
2760	ENSG00000228157.4	AC007952.2	2.00	2.02E-04	1.45E-03
2761	ENSG00000175040.5	CHST2	-0.73	2.03E-04	1.46E-03
2762	ENSG00000198315.10	ZKSCAN8	0.40	2.03E-04	1.46E-03
2763	ENSG00000204264.9	PSMB8	0.57	2.05E-04	1.47E-03
2764	ENSG00000108604.15	SMARCD2	-0.43	2.06E-04	1.48E-03
2765	ENSG00000235173.6	HGH1	0.50	2.06E-04	1.48E-03
2766	ENSG00000170412.16	GPRC5C	-1.52	2.06E-04	1.48E-03
2767	ENSG00000102096.9	PIM2	1.01	2.06E-04	1.48E-03
2768	ENSG00000121064.12	SCPEP1	-0.41	2.09E-04	1.49E-03
2769	ENSG00000196611.4	MMP1	-2.33	2.09E-04	1.49E-03
2770	ENSG00000092203.13	TOX4	0.34	2.10E-04	1.50E-03
2771	ENSG00000165688.11	PMPCA	0.41	2.10E-04	1.50E-03
2772	ENSG00000111653.19	ING4	-0.49	2.11E-04	1.51E-03
2773	ENSG00000137073.21	UBAP2	0.49	2.11E-04	1.51E-03
2774	ENSG00000165609.12	NUDT5	-0.38	2.11E-04	1.51E-03
2775	ENSG00000148153.13	INIP	-0.45	2.11E-04	1.51E-03
2776	ENSG00000075391.16	RASAL2	-0.41	2.11E-04	1.51E-03
2777	ENSG00000176381.5	PRR18	1.19	2.12E-04	1.51E-03
2778	ENSG00000112378.11	PERP	-0.56	2.12E-04	1.51E-03
2779	ENSG00000130956.13	HABP4	-0.49	2.15E-04	1.54E-03
2780	ENSG00000172757.12	CFL1	-0.36	2.16E-04	1.54E-03
2781	ENSG00000169925.16	BRD3	-0.44	2.16E-04	1.54E-03
2782	ENSG00000135940.6	COX5B	0.34	2.17E-04	1.55E-03
2783	ENSG00000103657.13	HERC1	0.36	2.17E-04	1.55E-03
2784	ENSG00000138413.13	IDH1	0.41	2.18E-04	1.55E-03
2785	ENSG00000255639.3	AC005833.1	0.90	2.18E-04	1.56E-03
2786	ENSG00000135441.7	BLOC1S1	0.44	2.19E-04	1.56E-03
2787	ENSG00000260121.1	AC138028.4	0.60	2.19E-04	1.56E-03
2788	ENSG00000132549.18	VPS13B	-0.36	2.19E-04	1.56E-03
2789	ENSG00000204947.8	ZNF425	-0.84	2.19E-04	1.56E-03
2790	ENSG00000129596.4	CDO1	0.35	2.20E-04	1.56E-03
2791	ENSG00000176490.4	DIRAS1	-0.76	2.20E-04	1.57E-03
2792	ENSG00000138778.11	CENPE	-1.37	2.21E-04	1.57E-03
2793	ENSG00000110925.6	CSRNP2	-0.34	2.21E-04	1.57E-03
2794	ENSG00000125863.19	MKKS	-0.52	2.21E-04	1.57E-03
2795	ENSG00000113580.14	NR3C1	-0.38	2.22E-04	1.57E-03
2796	ENSG00000158122.11	PRXL2C	-0.59	2.23E-04	1.58E-03
2797	ENSG00000132510.10	KDM6B	-0.58	2.23E-04	1.58E-03

2798	ENSG00000186815.12	TPCN1	-0.59	2.23E-04	1.58E-03
2799	ENSG00000138768.14	USO1	0.36	2.24E-04	1.59E-03
2800	ENSG00000186806.5	VSIG10L	0.80	2.24E-04	1.59E-03
2801	ENSG00000066056.13	TIE1	1.51	2.24E-04	1.59E-03
2802	ENSG00000064651.13	SLC12A2	-0.41	2.24E-04	1.59E-03
2803	ENSG00000144815.15	NXPE3	-0.61	2.25E-04	1.59E-03
2804	ENSG00000224251.6	AL391427.1	-0.88	2.25E-04	1.59E-03
2805	ENSG00000188878.19	FBF1	-0.79	2.25E-04	1.60E-03
2806	ENSG00000066135.12	KDM4A	-0.34	2.26E-04	1.60E-03
2807	ENSG00000146411.5	SLC2A12	-1.33	2.26E-04	1.60E-03
2808	ENSG00000146729.9	NIPSNAP2	0.38	2.27E-04	1.61E-03
2809	ENSG00000135093.12	USP30	0.45	2.29E-04	1.62E-03
2810	ENSG00000142583.17	SLC2A5	-0.69	2.30E-04	1.62E-03
2811	ENSG00000278845.4	MRPL45	0.38	2.30E-04	1.62E-03
2812	ENSG00000141510.17	TP53	-0.52	2.30E-04	1.62E-03
2813	ENSG00000145384.3	FABP2	2.05	2.30E-04	1.62E-03
2814	ENSG00000115556.13	PLCD4	-0.69	2.30E-04	1.62E-03
2815	ENSG00000170027.6	YWHAG	0.34	2.31E-04	1.63E-03
2816	ENSG00000112367.10	FIG4	0.48	2.32E-04	1.63E-03
2817	ENSG00000216863.9	LY86-AS1	-0.82	2.32E-04	1.64E-03
2818	ENSG00000185222.9	TCEAL9	-0.50	2.33E-04	1.64E-03
2819	ENSG00000178951.8	ZBTB7A	-0.47	2.33E-04	1.64E-03
2820	ENSG00000147854.16	UHRF2	-0.47	2.33E-04	1.64E-03
2821	ENSG00000170417.15	TMEM182	0.65	2.33E-04	1.64E-03
2822	ENSG00000144645.13	OSBPL10	-0.58	2.33E-04	1.64E-03
2823	ENSG00000189043.9	NDUFA4	0.32	2.34E-04	1.65E-03
2824	ENSG00000070756.15	PABPC1	0.37	2.35E-04	1.65E-03
2825	ENSG00000125430.8	HS3ST3B1	-0.66	2.35E-04	1.65E-03
2826	ENSG00000109944.10	JHY	-0.77	2.35E-04	1.65E-03
2827	ENSG00000224194.1	AC008278.1	4.69	2.36E-04	1.65E-03
2828	ENSG00000214021.15	TTLL3	-0.52	2.36E-04	1.66E-03
2829	ENSG00000214595.11	EML6	0.84	2.38E-04	1.67E-03
2830	ENSG00000106571.13	GLI3	-0.53	2.38E-04	1.67E-03
2831	ENSG00000231074.8	HCG18	0.44	2.39E-04	1.67E-03
2832	ENSG00000259985.1	AC017100.1	1.16	2.39E-04	1.67E-03
2833	ENSG00000170919.15	TPT1-AS1	-0.59	2.39E-04	1.68E-03
2834	ENSG00000109452.12	INPP4B	-0.54	2.39E-04	1.68E-03
2835	ENSG00000163872.15	YEATS2	-0.39	2.43E-04	1.70E-03
2836	ENSG00000266964.5	FXYD1	0.74	2.44E-04	1.71E-03
2837	ENSG00000143374.16	TARS2	0.47	2.44E-04	1.71E-03
2838	ENSG00000049239.12	H6PD	-0.43	2.44E-04	1.71E-03
2839	ENSG00000087245.12	MMP2	-0.50	2.44E-04	1.71E-03
2840	ENSG00000142657.20	PGD	0.33	2.48E-04	1.73E-03
2841	ENSG00000256654.3	AC005906.2	1.35	2.48E-04	1.74E-03
2842	ENSG00000142327.12	RNPEPL1	0.54	2.48E-04	1.74E-03
2843	ENSG00000242114.5	MTFP1	0.83	2.49E-04	1.74E-03
2844	ENSG00000169992.9	NLGN2	-0.42	2.49E-04	1.74E-03
2845	ENSG00000150433.9	TMEM218	0.47	2.51E-04	1.75E-03
2846	ENSG00000135912.10	TTLL4	0.53	2.51E-04	1.75E-03
2847	ENSG00000110987.8	BCL7A	0.54	2.52E-04	1.75E-03

2848	ENSG00000159199.13	ATP5MC1	0.46	2.52E-04	1.75E-03
2849	ENSG00000182512.4	GLRX5	0.41	2.52E-04	1.75E-03
2850	ENSG00000173114.12	LRRN3	-0.76	2.55E-04	1.78E-03
2851	ENSG00000128791.11	TWSG1	-0.37	2.57E-04	1.79E-03
2852	ENSG00000167615.16	LENG8	-0.42	2.57E-04	1.79E-03
2853	ENSG00000110315.6	RNF141	0.40	2.57E-04	1.79E-03
2854	ENSG00000086504.16	MRPL28	0.58	2.58E-04	1.79E-03
2855	ENSG00000186010.18	NDUFA13	0.39	2.60E-04	1.80E-03
2856	ENSG00000164530.14	PI16	-0.75	2.62E-04	1.82E-03
2857	ENSG00000136718.9	IMP4	0.43	2.62E-04	1.82E-03
2858	ENSG00000112078.13	KCTD20	0.34	2.63E-04	1.83E-03
2859	ENSG00000139624.12	CERS5	-0.43	2.63E-04	1.83E-03
2860	ENSG00000255302.4	EID1	-0.38	2.64E-04	1.83E-03
2861	ENSG00000115738.9	ID2	-0.45	2.65E-04	1.84E-03
2862	ENSG00000260597.1	AC012531.1	-0.77	2.65E-04	1.84E-03
2863	ENSG00000175309.14	PHYKPL	0.43	2.66E-04	1.85E-03
2864	ENSG00000127863.15	TNFRSF19	-0.99	2.66E-04	1.85E-03
2865	ENSG00000101474.11	APMAP	0.49	2.67E-04	1.85E-03
2866	ENSG00000146122.16	DAAM2	-0.44	2.67E-04	1.85E-03
2867	ENSG00000118007.12	STAG1	-0.38	2.69E-04	1.86E-03
2868	ENSG00000037965.5	HOXC8	-0.50	2.70E-04	1.87E-03
2869	ENSG00000154783.11	FGD5	1.89	2.73E-04	1.89E-03
2870	ENSG00000158079.15	PTPDC1	0.54	2.73E-04	1.89E-03
2871	ENSG00000118246.13	FASTKD2	0.37	2.73E-04	1.89E-03
2872	ENSG00000166396.12	SERPINB7	-0.79	2.74E-04	1.89E-03
2873	ENSG00000101972.18	STAG2	-0.36	2.74E-04	1.89E-03
2874	ENSG00000278685.4	IQCA1L	2.12	2.75E-04	1.90E-03
2875	ENSG00000148411.7	NACC2	-0.40	2.75E-04	1.90E-03
2876	ENSG00000231672.6	DIRC3	-1.32	2.75E-04	1.90E-03
2877	ENSG00000185010.14	F8	0.49	2.75E-04	1.90E-03
2878	ENSG00000154447.14	SH3RF1	-0.57	2.76E-04	1.90E-03
2879	ENSG00000254687.1	AC009646.2	4.21	2.76E-04	1.90E-03
2880	ENSG00000178162.8	FAR2P2	1.48	2.77E-04	1.91E-03
2881	ENSG00000151746.13	BICD1	-0.53	2.78E-04	1.91E-03
2882	ENSG00000080511.3	RDH8	2.96	2.79E-04	1.92E-03
2883	ENSG00000132429.9	POPDC3	0.65	2.79E-04	1.92E-03
2884	ENSG00000146963.17	LUC7L2	-0.44	2.80E-04	1.93E-03
2885	ENSG00000274523.4	RCC1L	0.44	2.82E-04	1.94E-03
2886	ENSG00000145545.11	SRD5A1	0.38	2.82E-04	1.94E-03
2887	ENSG00000260274.1	AC068338.2	0.91	2.85E-04	1.96E-03
2888	ENSG00000174899.10	PQLC2L	0.46	2.85E-04	1.96E-03
2889	ENSG00000140403.12	DNAJA4	0.62	2.85E-04	1.96E-03
2890	ENSG00000107140.15	TESK1	0.44	2.87E-04	1.97E-03
2891	ENSG00000019169.10	MARCO	-0.52	2.87E-04	1.97E-03
2892	ENSG00000122592.7	HOXA7	0.54	2.88E-04	1.97E-03
2893	ENSG00000118518.15	RNF146	-0.36	2.88E-04	1.98E-03
2894	ENSG00000167701.13	GPT	-0.97	2.89E-04	1.98E-03
2895	ENSG00000111300.9	NAA25	0.47	2.89E-04	1.98E-03
2896	ENSG00000137700.18	SLC37A4	0.59	2.90E-04	1.99E-03
2897	ENSG00000179841.8	AKAP5	1.37	2.90E-04	1.99E-03

2898	ENSG00000131475.6	VPS25	0.36	2.91E-04	1.99E-03
2899	ENSG00000160752.14	FDPS	-0.46	2.91E-04	1.99E-03
2900	ENSG00000120458.11	MSANTD2	-0.60	2.91E-04	1.99E-03
2901	ENSG00000159720.11	ATP6V0D1	0.37	2.91E-04	1.99E-03
2902	ENSG00000137968.16	SLC44A5	2.17	2.92E-04	2.00E-03
2903	ENSG00000153179.12	RASSF3	0.45	2.92E-04	2.00E-03
2904	ENSG00000226629.1	LINC00974	1.88	2.93E-04	2.00E-03
2905	ENSG00000149124.10	GLYAT	-1.35	2.93E-04	2.00E-03
2906	ENSG00000095627.9	TDRD1	3.31	2.93E-04	2.00E-03
2907	ENSG00000150961.14	SEC24D	-0.44	2.94E-04	2.01E-03
2908	ENSG00000160285.14	LSS	-0.53	2.95E-04	2.01E-03
2909	ENSG00000144935.14	TRPC1	-0.54	2.95E-04	2.01E-03
2910	ENSG00000132561.13	MATN2	-0.52	2.96E-04	2.02E-03
2911	ENSG00000168405.17	CMAHP	-0.57	2.96E-04	2.02E-03
2912	ENSG00000253301.5	LINC01606	3.56	2.97E-04	2.02E-03
2913	ENSG00000107611.15	CUBN	0.70	2.97E-04	2.02E-03
2914	ENSG00000111906.17	HDDC2	0.38	2.97E-04	2.03E-03
2915	ENSG00000232818.2	RPS2P32	1.37	2.98E-04	2.03E-03
2916	ENSG00000247596.8	TWF2	-1.59	2.99E-04	2.03E-03
2917	ENSG00000204843.12	DCTN1	-0.35	2.99E-04	2.03E-03
2918	ENSG00000198663.16	C6orf89	-0.35	2.99E-04	2.03E-03
2919	ENSG00000145632.14	PLK2	-0.61	3.02E-04	2.05E-03
2920	ENSG00000240053.8	LY6G5B	-0.73	3.02E-04	2.05E-03
2921	ENSG00000100934.14	SEC23A	0.35	3.02E-04	2.05E-03
2922	ENSG00000241860.6	AL627309.5	0.55	3.03E-04	2.06E-03
2923	ENSG00000102466.15	FGF14	-0.66	3.03E-04	2.06E-03
2924	ENSG00000101079.20	NDRG3	-0.50	3.09E-04	2.10E-03
2925	ENSG00000116455.13	WDR77	0.42	3.09E-04	2.10E-03
2926	ENSG00000260456.6	C16orf95	0.93	3.11E-04	2.11E-03
2927	ENSG00000116815.15	CD58	-0.66	3.11E-04	2.11E-03
2928	ENSG00000155099.7	PIP4P2	-0.46	3.13E-04	2.12E-03
2929	ENSG00000104177.17	MYEF2	0.48	3.14E-04	2.13E-03
2930	ENSG00000170949.17	ZNF160	-0.45	3.16E-04	2.14E-03
2931	ENSG00000180229.12	HERC2P3	-0.57	3.18E-04	2.16E-03
2932	ENSG00000162444.11	RBP7	1.50	3.19E-04	2.16E-03
2933	ENSG00000148926.9	ADM	-0.49	3.19E-04	2.16E-03
2934	ENSG00000164307.12	ERAP1	0.34	3.19E-04	2.16E-03
2935	ENSG00000069974.15	RAB27A	-0.70	3.19E-04	2.16E-03
2936	ENSG00000100416.13	TRMU	0.44	3.20E-04	2.16E-03
2937	ENSG00000117461.14	PIK3R3	-0.64	3.20E-04	2.16E-03
2938	ENSG00000184887.13	BTBD6	0.38	3.22E-04	2.17E-03
2939	ENSG00000182158.14	CREB3L2	-0.42	3.25E-04	2.19E-03
2940	ENSG00000269609.5	RPARP-AS1	0.74	3.26E-04	2.20E-03
2941	ENSG00000173821.19	RNF213	-0.39	3.28E-04	2.21E-03
2942	ENSG00000114650.19	SCAP	0.41	3.29E-04	2.22E-03
2943	ENSG00000263004.1	AC007114.1	1.52	3.32E-04	2.24E-03
2944	ENSG00000125656.9	CLPP	0.51	3.32E-04	2.24E-03
2945	ENSG00000141179.13	PCTP	0.55	3.32E-04	2.24E-03
2946	ENSG00000162065.13	TBC1D24	0.46	3.32E-04	2.24E-03
2947	ENSG00000248187.1	AC078850.1	-1.42	3.33E-04	2.24E-03

2948	ENSG00000086159.12	AQP6	2.18	3.35E-04	2.26E-03
2949	ENSG00000185532.16	PRKG1	-0.44	3.37E-04	2.27E-03
2950	ENSG00000250934.1	AC016924.1	0.63	3.37E-04	2.27E-03
2951	ENSG00000102359.6	SRPX2	0.69	3.38E-04	2.27E-03
2952	ENSG00000165323.15	FAT3	-0.82	3.39E-04	2.28E-03
2953	ENSG00000050327.14	ARHGEF5	0.83	3.39E-04	2.28E-03
2954	ENSG00000169855.19	ROBO1	0.38	3.40E-04	2.29E-03
2955	ENSG00000166886.12	NAB2	-0.55	3.41E-04	2.29E-03
2956	ENSG00000103740.9	ACSBG1	1.18	3.42E-04	2.29E-03
2957	ENSG00000188163.7	FAM166A	1.87	3.42E-04	2.30E-03
2958	ENSG00000119522.16	DENND1A	0.52	3.43E-04	2.30E-03
2959	ENSG00000228253.1	MT-ATP8	0.36	3.43E-04	2.30E-03
2960	ENSG00000283473.2	FAM240A	-1.58	3.43E-04	2.30E-03
2961	ENSG00000124496.12	TRERF1	-0.65	3.44E-04	2.31E-03
2962	ENSG00000268895.5	A1BG-AS1	-0.80	3.45E-04	2.31E-03
2963	ENSG00000165124.17	SVEP1	-0.52	3.45E-04	2.31E-03
2964	ENSG00000168899.4	VAMP5	-0.73	3.45E-04	2.31E-03
2965	ENSG00000204248.10	COL11A2	-1.68	3.45E-04	2.31E-03
2966	ENSG00000092068.19	SLC7A8	0.58	3.46E-04	2.31E-03
2967	ENSG00000170296.9	GABARAP	-0.31	3.46E-04	2.31E-03
2968	ENSG00000164828.17	SUN1	-0.33	3.48E-04	2.33E-03
2969	ENSG00000005243.9	COPZ2	-0.40	3.49E-04	2.33E-03
2970	ENSG00000145982.12	FARS2	0.54	3.50E-04	2.34E-03
2971	ENSG00000144339.11	TMEFF2	-0.71	3.54E-04	2.36E-03
2972	ENSG00000163638.13	ADAMTS9	0.45	3.54E-04	2.37E-03
2973	ENSG00000140450.8	ARRDC4	-0.48	3.54E-04	2.37E-03
2974	ENSG00000083099.10	LYRM2	0.39	3.56E-04	2.37E-03
2975	ENSG00000163590.13	PPM1L	-0.73	3.56E-04	2.38E-03
2976	ENSG00000180185.11	FAHD1	0.44	3.59E-04	2.40E-03
2977	ENSG00000120802.13	TMPO	-0.48	3.60E-04	2.40E-03
2978	ENSG00000103335.22	PIEZO1	0.53	3.60E-04	2.40E-03
2979	ENSG00000184007.20	PTP4A2	-0.31	3.61E-04	2.41E-03
2980	ENSG00000112096.17	SOD2	-0.41	3.64E-04	2.42E-03
2981	ENSG00000150556.16	LYPD6B	-1.04	3.64E-04	2.42E-03
2982	ENSG00000125868.15	DSTN	-0.34	3.65E-04	2.43E-03
2983	ENSG00000085733.15	CTTN	-0.34	3.65E-04	2.43E-03
2984	ENSG00000143952.19	VPS54	0.41	3.66E-04	2.44E-03
2985	ENSG00000176749.8	CDK5R1	0.93	3.67E-04	2.44E-03
2986	ENSG00000090924.14	PLEKHG2	-0.47	3.68E-04	2.45E-03
2987	ENSG00000184545.10	DUSP8	1.01	3.68E-04	2.45E-03
2988	ENSG00000127125.8	PPCS	0.39	3.69E-04	2.45E-03
2989	ENSG00000173276.13	ZBTB21	0.47	3.72E-04	2.47E-03
2990	ENSG00000154529.14	CNTNAP3B	0.60	3.72E-04	2.47E-03
2991	ENSG00000092531.9	SNAP23	-0.39	3.73E-04	2.47E-03
2992	ENSG00000013016.15	EHD3	-0.81	3.73E-04	2.48E-03
2993	ENSG00000119771.14	KLHL29	-0.52	3.73E-04	2.48E-03
2994	ENSG00000121716.20	PILRB	0.51	3.74E-04	2.48E-03
2995	ENSG00000271774.1	AL109930.1	-2.07	3.75E-04	2.49E-03
2996	ENSG00000021645.18	NRXN3	-0.60	3.77E-04	2.50E-03
2997	ENSG00000135632.11	SMYD5	0.44	3.78E-04	2.50E-03

2998	ENSG00000196460.13	RFX8	-2.38	3.79E-04	2.51E-03
2999	ENSG00000198018.6	ENTPD7	-0.53	3.80E-04	2.52E-03
3000	ENSG00000265799.1	AC090844.3	0.99	3.80E-04	2.52E-03
3001	ENSG00000184939.15	ZFP90	-0.40	3.81E-04	2.52E-03
3002	ENSG00000169570.9	DTWD2	0.50	3.83E-04	2.53E-03
3003	ENSG00000254445.1	HSPB2-C11orf52	-1.45	3.83E-04	2.53E-03
3004	ENSG00000105254.11	TBCB	-0.47	3.84E-04	2.54E-03
3005	ENSG00000185100.10	ADSSL1	0.91	3.84E-04	2.54E-03
3006	ENSG00000142173.14	COL6A2	-0.53	3.84E-04	2.54E-03
3007	ENSG00000187824.8	TMEM220	0.64	3.86E-04	2.54E-03
3008	ENSG00000198721.12	ECI2	0.37	3.86E-04	2.54E-03
3009	ENSG00000214940.8	NPIPA8	-0.57	3.86E-04	2.54E-03
3010	ENSG00000144583.4	MARCH4	-0.69	3.86E-04	2.54E-03
3011	ENSG00000122085.16	MTERF4	0.39	3.88E-04	2.56E-03
3012	ENSG00000104980.7	TIMM44	0.40	3.90E-04	2.57E-03
3013	ENSG00000173376.13	NDNF	-1.07	3.90E-04	2.57E-03
3014	ENSG00000259705.1	AC084757.3	-2.79	3.91E-04	2.58E-03
3015	ENSG00000228971.2	AL356479.1	0.88	3.92E-04	2.58E-03
3016	ENSG00000160307.9	S100B	-0.72	3.92E-04	2.58E-03
3017	ENSG00000008382.15	MPND	0.70	3.93E-04	2.58E-03
3018	ENSG00000157796.17	WDR19	-0.38	3.95E-04	2.60E-03
3019	ENSG00000260409.1	AC012414.5	2.37	3.97E-04	2.61E-03
3020	ENSG00000272831.1	AC027644.3	0.96	4.01E-04	2.64E-03
3021	ENSG00000149091.15	DGKZ	0.46	4.01E-04	2.64E-03
3022	ENSG00000177156.10	TALDO1	0.32	4.05E-04	2.66E-03
3023	ENSG00000143153.12	ATP1B1	-0.46	4.05E-04	2.66E-03
3024	ENSG00000170266.15	GLB1	0.37	4.07E-04	2.67E-03
3025	ENSG00000155096.13	AZIN1	0.34	4.11E-04	2.70E-03
3026	ENSG00000212766.9	EWSAT1	-2.77	4.12E-04	2.70E-03
3027	ENSG00000136518.16	ACTL6A	-0.44	4.14E-04	2.71E-03
3028	ENSG00000187546.13	AGMO	1.11	4.15E-04	2.72E-03
3029	ENSG00000141552.17	ANAPC11	0.48	4.17E-04	2.73E-03
3030	ENSG00000105290.11	APLP1	-0.72	4.17E-04	2.73E-03
3031	ENSG00000150967.17	ABCB9	-0.87	4.18E-04	2.74E-03
3032	ENSG00000101166.15	PRELID3B	0.43	4.19E-04	2.75E-03
3033	ENSG00000154001.13	PPP2R5E	-0.40	4.19E-04	2.75E-03
3034	ENSG00000163466.15	ARPC2	-0.38	4.23E-04	2.77E-03
3035	ENSG00000134851.12	TMEM165	-0.35	4.23E-04	2.77E-03
3036	ENSG00000167700.8	MFSD3	0.67	4.24E-04	2.77E-03
3037	ENSG00000260836.2	AC245033.1	2.45	4.25E-04	2.78E-03
3038	ENSG00000104368.17	PLAT	-0.77	4.25E-04	2.78E-03
3039	ENSG00000123575.8	FAM199X	0.37	4.26E-04	2.78E-03
3040	ENSG00000198911.11	SREBF2	-0.38	4.28E-04	2.80E-03
3041	ENSG00000258657.5	AL136018.1	1.63	4.30E-04	2.81E-03
3042	ENSG00000165113.12	GKAP1	0.59	4.30E-04	2.81E-03
3043	ENSG00000153162.8	BMP6	-0.57	4.35E-04	2.84E-03
3044	ENSG00000072210.18	ALDH3A2	0.39	4.35E-04	2.84E-03
3045	ENSG00000137877.9	SPTBN5	2.21	4.36E-04	2.84E-03
3046	ENSG00000052802.12	MSMO1	0.39	4.36E-04	2.84E-03
3047	ENSG00000108821.13	COL1A1	-0.49	4.37E-04	2.85E-03

3048	ENSG00000237125.9	HAND2-AS1	-0.52	4.38E-04	2.85E-03
3049	ENSG00000138378.18	STAT4	0.97	4.38E-04	2.85E-03
3050	ENSG00000242485.5	MRPL20	0.37	4.40E-04	2.86E-03
3051	ENSG00000119729.11	RHOQ	0.38	4.40E-04	2.87E-03
3052	ENSG00000260400.1	AL513534.1	-0.68	4.44E-04	2.88E-03
3053	ENSG00000184347.14	SLIT3	-0.40	4.45E-04	2.89E-03
3054	ENSG00000165312.6	OTUD1	-0.64	4.46E-04	2.90E-03
3055	ENSG00000116117.17	PARD3B	-0.46	4.46E-04	2.90E-03
3056	ENSG00000027847.13	B4GALT7	0.49	4.47E-04	2.90E-03
3057	ENSG00000125675.17	GRIA3	-0.75	4.47E-04	2.90E-03
3058	ENSG00000275066.4	SYNRG	0.38	4.47E-04	2.90E-03
3059	ENSG00000188783.5	PRELP	-1.29	4.47E-04	2.90E-03
3060	ENSG00000107742.12	SPOCK2	1.27	4.49E-04	2.92E-03
3061	ENSG00000153989.7	NUS1	0.38	4.51E-04	2.93E-03
3062	ENSG00000130414.11	NDUFA10	0.31	4.52E-04	2.93E-03
3063	ENSG00000126705.14	AHDC1	-0.64	4.52E-04	2.93E-03
3064	ENSG00000107959.15	PITRM1	0.30	4.53E-04	2.93E-03
3065	ENSG00000154642.10	C21orf91	-0.59	4.53E-04	2.93E-03
3066	ENSG00000060718.21	COL11A1	-0.42	4.54E-04	2.94E-03
3067	ENSG00000110841.13	PPFIBP1	-0.40	4.59E-04	2.97E-03
3068	ENSG00000115042.9	FAHD2A	0.41	4.61E-04	2.98E-03
3069	ENSG00000142794.18	NBPF3	0.48	4.64E-04	3.00E-03
3070	ENSG00000066427.22	ATXN3	-0.49	4.64E-04	3.00E-03
3071	ENSG00000187607.15	ZNF286A	-0.50	4.64E-04	3.00E-03
3072	ENSG00000163626.16	COX18	0.44	4.65E-04	3.00E-03
3073	ENSG00000136451.8	VEZF1	-0.37	4.65E-04	3.00E-03
3074	ENSG00000164051.13	CCDC51	0.47	4.67E-04	3.01E-03
3075	ENSG00000124782.19	RREB1	0.41	4.67E-04	3.02E-03
3076	ENSG00000111371.15	SLC38A1	-0.30	4.68E-04	3.02E-03
3077	ENSG00000168264.10	IRF2BP2	-0.44	4.70E-04	3.03E-03
3078	ENSG00000274422.1	AC245060.5	0.95	4.71E-04	3.03E-03
3079	ENSG00000272129.1	AL359715.3	0.75	4.71E-04	3.03E-03
3080	ENSG00000135540.11	NHSL1	0.61	4.71E-04	3.04E-03
3081	ENSG00000157613.10	CREB3L1	-0.53	4.74E-04	3.05E-03
3082	ENSG00000224968.1	LINC01645	3.55	4.74E-04	3.06E-03
3083	ENSG00000184224.3	C11orf72	1.08	4.75E-04	3.06E-03
3084	ENSG00000169764.15	UGP2	0.39	4.76E-04	3.06E-03
3085	ENSG00000170899.10	GSTA4	-0.38	4.80E-04	3.09E-03
3086	ENSG00000163251.3	FZD5	0.52	4.82E-04	3.10E-03
3087	ENSG00000073111.13	MCM2	0.74	4.83E-04	3.10E-03
3088	ENSG00000108424.9	KPNB1	-0.33	4.83E-04	3.10E-03
3089	ENSG00000133687.15	TMTC1	-0.60	4.83E-04	3.10E-03
3090	ENSG00000070759.16	TESK2	0.93	4.86E-04	3.12E-03
3091	ENSG00000270069.1	MIR222HG	-1.29	4.88E-04	3.13E-03
3092	ENSG00000087301.8	TXNDC16	0.54	4.89E-04	3.14E-03
3093	ENSG00000039537.13	C6	3.13	4.90E-04	3.14E-03
3094	ENSG00000178467.17	P4HTM	-0.61	4.90E-04	3.14E-03
3095	ENSG00000198774.4	RASSF9	-0.73	4.93E-04	3.16E-03
3096	ENSG00000072042.12	RDH11	0.34	4.96E-04	3.18E-03
3097	ENSG00000117308.14	GALE	0.60	5.01E-04	3.21E-03

3098	ENSG00000166263.13	STXBP4	-0.56	5.06E-04	3.24E-03
3099	ENSG00000273841.4	TAF9	0.40	5.07E-04	3.25E-03
3100	ENSG00000141391.13	PRELID3A	0.74	5.10E-04	3.27E-03
3101	ENSG00000110104.12	CCDC86	0.51	5.15E-04	3.29E-03
3102	ENSG00000160767.20	FAM189B	-0.60	5.15E-04	3.29E-03
3103	ENSG00000158966.14	CACHD1	-0.54	5.16E-04	3.30E-03
3104	ENSG00000113657.12	DPYSL3	-0.63	5.20E-04	3.32E-03
3105	ENSG00000030110.12	BAK1	-0.47	5.20E-04	3.32E-03
3106	ENSG00000159307.18	SCUBE1	-1.73	5.21E-04	3.33E-03
3107	ENSG00000102699.5	PARP4	-0.40	5.21E-04	3.33E-03
3108	ENSG00000145555.14	MYO10	-0.45	5.24E-04	3.34E-03
3109	ENSG00000166595.11	CIAO2B	0.43	5.25E-04	3.35E-03
3110	ENSG00000105516.10	DBP	-0.86	5.26E-04	3.36E-03
3111	ENSG00000064199.6	SPA17	-0.81	5.28E-04	3.37E-03
3112	ENSG00000183023.18	SLC8A1	-0.62	5.28E-04	3.37E-03
3113	ENSG00000075790.10	BCAP29	-0.40	5.28E-04	3.37E-03
3114	ENSG00000168234.12	TTC39C	-0.47	5.29E-04	3.37E-03
3115	ENSG00000228133.2	AC099684.1	3.65	5.31E-04	3.38E-03
3116	ENSG00000183775.10	KCTD16	-0.71	5.31E-04	3.38E-03
3117	ENSG00000115902.10	SLC1A4	-0.43	5.32E-04	3.39E-03
3118	ENSG00000272447.1	AL135925.1	0.69	5.33E-04	3.39E-03
3119	ENSG00000025708.13	TYMP	-0.93	5.33E-04	3.39E-03
3120	ENSG00000157823.16	AP3S2	0.37	5.34E-04	3.40E-03
3121	ENSG00000079156.16	OSBPL6	-0.56	5.36E-04	3.41E-03
3122	ENSG00000111669.14	TPI1	-0.37	5.37E-04	3.41E-03
3123	ENSG00000171236.9	LRG1	2.51	5.37E-04	3.41E-03
3124	ENSG00000261757.1	AC005592.1	-1.65	5.40E-04	3.43E-03
3125	ENSG00000184261.4	KCNK12	1.14	5.40E-04	3.43E-03
3126	ENSG00000270055.1	AC127502.2	0.40	5.41E-04	3.43E-03
3127	ENSG00000134545.13	KLRC1	-3.05	5.44E-04	3.45E-03
3128	ENSG00000160685.13	ZBTB7B	-0.46	5.44E-04	3.45E-03
3129	ENSG00000142599.17	RERE	0.35	5.49E-04	3.48E-03
3130	ENSG00000167693.16	NXN	-0.49	5.49E-04	3.48E-03
3131	ENSG00000136542.8	GALNT5	-0.59	5.52E-04	3.50E-03
3132	ENSG00000160050.14	CCDC28B	-0.92	5.52E-04	3.50E-03
3133	ENSG00000179833.4	SERTAD2	-0.37	5.54E-04	3.51E-03
3134	ENSG00000181264.8	TMEM136	-0.59	5.55E-04	3.52E-03
3135	ENSG00000150540.13	HNMT	-0.42	5.56E-04	3.52E-03
3136	ENSG00000100504.16	PYGL	-0.43	5.57E-04	3.53E-03
3137	ENSG00000223442.1	TH2LCRR	2.67	5.58E-04	3.53E-03
3138	ENSG00000149269.9	PAK1	-0.41	5.59E-04	3.54E-03
3139	ENSG00000135966.12	TGFBRAP1	0.37	5.61E-04	3.55E-03
3140	ENSG00000054983.16	GALC	0.39	5.62E-04	3.56E-03
3141	ENSG00000011677.12	GABRA3	3.46	5.63E-04	3.56E-03
3142	ENSG00000109917.10	ZPR1	0.39	5.63E-04	3.56E-03
3143	ENSG00000179163.11	FUCA1	0.53	5.64E-04	3.57E-03
3144	ENSG00000150907.7	FOXO1	0.39	5.66E-04	3.57E-03
3145	ENSG00000168938.5	PPIC	-0.39	5.70E-04	3.60E-03
3146	ENSG00000153107.12	ANAPC1	0.34	5.72E-04	3.61E-03
3147	ENSG00000214717.11	ZBED1	-0.42	5.72E-04	3.61E-03

3148	ENSG00000196924.15	FLNA	-0.53	5.73E-04	3.61E-03
3149	ENSG00000145349.16	CAMK2D	-0.37	5.73E-04	3.61E-03
3150	ENSG00000099721.13	AMELY	1.98	5.76E-04	3.63E-03
3151	ENSG00000250337.5	PURPL	-0.72	5.77E-04	3.64E-03
3152	ENSG00000125755.18	SYMPK	-0.42	5.79E-04	3.65E-03
3153	ENSG00000183889.12	PKD1P1	-0.50	5.80E-04	3.65E-03
3154	ENSG00000112237.12	CCNC	0.39	5.84E-04	3.68E-03
3155	ENSG00000204442.3	FAM155A	-0.99	5.88E-04	3.70E-03
3156	ENSG00000129566.12	TEP1	-0.48	5.88E-04	3.70E-03
3157	ENSG00000168994.13	PXDC1	-0.36	5.92E-04	3.72E-03
3158	ENSG00000213928.8	IRF9	-0.54	5.92E-04	3.72E-03
3159	ENSG00000114923.16	SLC4A3	-0.72	5.92E-04	3.72E-03
3160	ENSG00000163597.14	SNHG16	0.38	5.93E-04	3.72E-03
3161	ENSG00000115419.12	GLS	-0.42	5.99E-04	3.76E-03
3162	ENSG00000004660.14	CAMKK1	-1.04	6.01E-04	3.77E-03
3163	ENSG00000213215.5	OR2F1	1.71	6.07E-04	3.80E-03
3164	ENSG00000278611.1	ZNF426-DT	1.04	6.07E-04	3.80E-03
3165	ENSG00000154814.13	OXNAD1	0.47	6.07E-04	3.80E-03
3166	ENSG00000175029.16	CTBP2	-0.52	6.07E-04	3.80E-03
3167	ENSG00000109738.10	GLRB	0.64	6.09E-04	3.81E-03
3168	ENSG00000154310.16	TNIK	-0.68	6.10E-04	3.82E-03
3169	ENSG00000125454.11	SLC25A19	0.66	6.11E-04	3.83E-03
3170	ENSG00000067191.15	CACNB1	-0.71	6.11E-04	3.83E-03
3171	ENSG00000028116.17	VRK2	-0.47	6.18E-04	3.87E-03
3172	ENSG00000101311.15	FERMT1	0.73	6.20E-04	3.88E-03
3173	ENSG00000163697.16	APBB2	-0.37	6.22E-04	3.89E-03
3174	ENSG00000108854.15	SMURF2	-0.52	6.22E-04	3.89E-03
3175	ENSG00000092345.13	DAZL	0.94	6.23E-04	3.89E-03
3176	ENSG00000175550.7	DRAP1	0.41	6.23E-04	3.89E-03
3177	ENSG00000185710.9	SMG1P4	-1.00	6.24E-04	3.90E-03
3178	ENSG00000168246.5	UBTD2	-0.36	6.25E-04	3.90E-03
3179	ENSG00000165072.9	MAMDC2	-0.56	6.28E-04	3.92E-03
3180	ENSG00000272779.1	AC245060.4	0.47	6.31E-04	3.94E-03
3181	ENSG00000155850.7	SLC26A2	-0.41	6.33E-04	3.95E-03
3182	ENSG00000182197.11	EXT1	-0.44	6.34E-04	3.95E-03
3183	ENSG00000144550.12	CPNE9	2.69	6.36E-04	3.97E-03
3184	ENSG00000165730.15	STOX1	0.95	6.37E-04	3.97E-03
3185	ENSG00000107036.11	RIC1	-0.31	6.37E-04	3.97E-03
3186	ENSG00000130147.15	SH3BP4	-0.36	6.39E-04	3.98E-03
3187	ENSG00000198121.13	LPAR1	-0.54	6.39E-04	3.98E-03
3188	ENSG00000101224.17	CDC25B	-0.55	6.40E-04	3.98E-03
3189	ENSG00000246273.7	SBF2-AS1	-0.51	6.43E-04	4.00E-03
3190	ENSG00000262576.2	PCDHGA4	-0.57	6.45E-04	4.01E-03
3191	ENSG00000249592.5	AC139887.2	-1.21	6.46E-04	4.02E-03
3192	ENSG00000104856.13	RELB	0.67	6.55E-04	4.07E-03
3193	ENSG00000165672.6	PRDX3	0.31	6.56E-04	4.07E-03
3194	ENSG00000168785.7	TSPAN5	-0.55	6.58E-04	4.09E-03
3195	ENSG00000131759.17	RARA	-0.50	6.60E-04	4.10E-03
3196	ENSG00000134452.19	FBH1	-0.35	6.63E-04	4.12E-03
3197	ENSG00000107551.20	RASSF4	-0.47	6.64E-04	4.12E-03

3198	ENSG00000213676.12	ATF6B	-0.39	6.64E-04	4.12E-03
3199	ENSG00000122696.13	SLC25A51	0.37	6.65E-04	4.12E-03
3200	ENSG00000135052.16	GOLM1	-0.51	6.65E-04	4.12E-03
3201	ENSG00000087266.15	SH3BP2	0.56	6.68E-04	4.14E-03
3202	ENSG00000251230.5	MIR3945HG	1.80	6.71E-04	4.16E-03
3203	ENSG00000140262.17	TCF12	-0.36	6.72E-04	4.17E-03
3204	ENSG00000118257.16	NRP2	-0.49	6.74E-04	4.18E-03
3205	ENSG00000165752.16	STK32C	0.54	6.75E-04	4.18E-03
3206	ENSG00000109320.11	NFKB1	-0.41	6.79E-04	4.20E-03
3207	ENSG00000164733.20	CTSB	-0.44	6.79E-04	4.20E-03
3208	ENSG00000255282.6	WTAPP1	-2.52	6.79E-04	4.20E-03
3209	ENSG00000108582.11	CPD	-0.30	6.81E-04	4.21E-03
3210	ENSG00000143933.16	CALM2	-0.35	6.82E-04	4.21E-03
3211	ENSG00000132792.18	CTNNBL1	0.50	6.83E-04	4.22E-03
3212	ENSG00000169018.5	FEM1B	-0.30	6.84E-04	4.22E-03
3213	ENSG00000122966.15	CIT	-1.77	6.84E-04	4.23E-03
3214	ENSG00000188895.11	MSL1	-0.31	6.85E-04	4.23E-03
3215	ENSG00000144283.21	PKP4	-0.37	6.87E-04	4.24E-03
3216	ENSG00000121989.14	ACVR2A	-0.48	6.87E-04	4.24E-03
3217	ENSG00000247516.6	MIR4458HG	0.70	6.87E-04	4.24E-03
3218	ENSG00000111961.17	SASH1	-0.44	6.89E-04	4.25E-03
3219	ENSG00000139977.13	NAA30	0.39	6.90E-04	4.25E-03
3220	ENSG00000105327.17	BBC3	-0.94	6.91E-04	4.26E-03
3221	ENSG00000139531.12	SUOX	-0.37	6.95E-04	4.28E-03
3222	ENSG00000171150.8	SOCS5	-0.45	6.97E-04	4.30E-03
3223	ENSG00000005844.17	ITGAL	1.34	7.00E-04	4.31E-03
3224	ENSG00000165282.13	PIGO	0.47	7.00E-04	4.31E-03
3225	ENSG00000156411.9	ATP5MPL	0.36	7.05E-04	4.34E-03
3226	ENSG00000163827.12	LRRC2	-0.73	7.08E-04	4.35E-03
3227	ENSG00000248643.5	RBM14-RBM4	1.32	7.08E-04	4.36E-03
3228	ENSG00000142798.19	HSPG2	-0.56	7.09E-04	4.36E-03
3229	ENSG00000177398.18	UMODL1	2.72	7.10E-04	4.36E-03
3230	ENSG00000161958.10	FGF11	-1.35	7.12E-04	4.37E-03
3231	ENSG00000131748.15	STARD3	0.46	7.13E-04	4.38E-03
3232	ENSG00000279373.1	AC027228.3	2.54	7.13E-04	4.38E-03
3233	ENSG00000236714.1	LINC01844	-1.01	7.17E-04	4.40E-03
3234	ENSG00000256514.1	AP003419.1	-0.54	7.17E-04	4.40E-03
3235	ENSG00000159685.10	CHCHD6	0.69	7.19E-04	4.41E-03
3236	ENSG00000181222.15	POLR2A	-0.39	7.20E-04	4.41E-03
3237	ENSG00000163788.13	SNRK	0.39	7.20E-04	4.42E-03
3238	ENSG00000091483.6	FH	0.30	7.21E-04	4.42E-03
3239	ENSG00000275185.1	AC130324.3	0.98	7.24E-04	4.43E-03
3240	ENSG00000138760.9	SCARB2	0.30	7.28E-04	4.46E-03
3241	ENSG00000074047.21	GLI2	-0.67	7.28E-04	4.46E-03
3242	ENSG00000151327.12	FAM177A1	-0.40	7.28E-04	4.46E-03
3243	ENSG00000095951.16	HIVEP1	-0.45	7.29E-04	4.46E-03
3244	ENSG00000146826.16	C7orf43	0.49	7.31E-04	4.47E-03
3245	ENSG00000013306.15	SLC25A39	0.44	7.31E-04	4.47E-03
3246	ENSG00000100784.11	RPS6KA5	0.90	7.34E-04	4.49E-03
3247	ENSG00000127946.16	HIP1	-0.53	7.36E-04	4.50E-03

3248	ENSG00000181381.13	DDX60L	-0.58	7.36E-04	4.50E-03
3249	ENSG00000110092.3	CCND1	-1.01	7.37E-04	4.50E-03
3250	ENSG00000163864.16	NMNAT3	0.53	7.39E-04	4.51E-03
3251	ENSG00000221869.4	CEBPD	-0.71	7.41E-04	4.52E-03
3252	ENSG00000140443.14	IGF1R	-0.46	7.44E-04	4.54E-03
3253	ENSG00000116132.11	PRRX1	-0.48	7.46E-04	4.55E-03
3254	ENSG00000186063.12	AIDA	0.38	7.46E-04	4.55E-03
3255	ENSG00000124440.15	HIF3A	-1.35	7.46E-04	4.55E-03
3256	ENSG00000155792.9	DEPTOR	-0.40	7.47E-04	4.55E-03
3257	ENSG00000132196.14	HSD17B7	0.54	7.50E-04	4.57E-03
3258	ENSG00000197586.12	ENTPD6	0.39	7.51E-04	4.57E-03
3259	ENSG00000167996.15	FTH1	-0.41	7.54E-04	4.59E-03
3260	ENSG00000151718.15	WWC2	-0.37	7.58E-04	4.61E-03
3261	ENSG00000213977.7	TAX1BP3	-0.41	7.59E-04	4.62E-03
3262	ENSG00000260565.6	ERVK13-1	0.39	7.61E-04	4.62E-03
3263	ENSG00000136010.13	ALDH1L2	-0.39	7.61E-04	4.62E-03
3264	ENSG00000250510.7	GPR162	-0.95	7.61E-04	4.62E-03
3265	ENSG00000158604.14	TMED4	0.34	7.61E-04	4.63E-03
3266	ENSG00000137404.14	NRM	-0.91	7.62E-04	4.63E-03
3267	ENSG00000244479.7	OR2A1-AS1	1.92	7.63E-04	4.63E-03
3268	ENSG00000005483.20	KMT2E	-0.34	7.63E-04	4.63E-03
3269	ENSG00000267576.1	AC011472.3	-1.18	7.66E-04	4.65E-03
3270	ENSG00000196154.11	S100A4	-1.24	7.67E-04	4.65E-03
3271	ENSG00000182103.4	FAM181B	2.18	7.68E-04	4.66E-03
3272	ENSG00000071205.11	ARHGAP10	-0.48	7.70E-04	4.67E-03
3273	ENSG00000078369.17	GNB1	-0.28	7.73E-04	4.69E-03
3274	ENSG00000171016.12	PYGO1	-0.44	7.76E-04	4.70E-03
3275	ENSG00000181220.16	ZNF746	-0.49	7.80E-04	4.72E-03
3276	ENSG00000068885.14	IFT80	-0.48	7.81E-04	4.73E-03
3277	ENSG00000166446.14	CDYL2	0.44	7.84E-04	4.75E-03
3278	ENSG00000121440.14	PDZRN3	-0.43	7.84E-04	4.75E-03
3279	ENSG00000164077.14	MON1A	0.52	7.85E-04	4.75E-03
3280	ENSG00000117984.13	CTSD	0.48	7.85E-04	4.75E-03
3281	ENSG00000100441.9	KHNYN	0.35	7.86E-04	4.75E-03
3282	ENSG00000170759.10	KIF5B	0.31	7.88E-04	4.77E-03
3283	ENSG00000161920.9	MED11	0.50	7.90E-04	4.78E-03
3284	ENSG00000181619.11	GPR135	-0.83	7.92E-04	4.79E-03
3285	ENSG00000187398.11	LUZP2	-0.66	7.93E-04	4.79E-03
3286	ENSG00000125170.10	DOK4	-0.55	7.95E-04	4.80E-03
3287	ENSG00000136280.16	CCM2	0.40	8.00E-04	4.83E-03
3288	ENSG00000130382.8	MLLT1	-0.41	8.04E-04	4.86E-03
3289	ENSG00000262454.3	MIR193BHG	-0.56	8.05E-04	4.86E-03
3290	ENSG00000114374.12	USP9Y	-0.35	8.08E-04	4.87E-03
3291	ENSG00000109743.10	BST1	-0.69	8.10E-04	4.88E-03
3292	ENSG00000279200.1	AC020558.3	1.60	8.11E-04	4.89E-03
3293	ENSG00000241158.6	ADAMTS9-AS1	-0.61	8.15E-04	4.91E-03
3294	ENSG00000198795.10	ZNF521	-0.67	8.17E-04	4.92E-03
3295	ENSG00000189339.11	SLC35E2B	-0.30	8.17E-04	4.92E-03
3296	ENSG00000103226.17	NOMO3	0.34	8.19E-04	4.93E-03
3297	ENSG00000176531.10	PHLDB3	0.92	8.21E-04	4.94E-03

3298	ENSG00000136522.13	MRPL47	0.41	8.21E-04	4.94E-03
3299	ENSG00000198742.9	SMURF1	0.36	8.31E-04	5.00E-03
3300	ENSG00000011007.12	ELOA	0.31	8.32E-04	5.00E-03
3301	ENSG00000196199.13	MPHOSPH8	-0.43	8.42E-04	5.06E-03
3302	ENSG00000203965.12	EFCAB7	-0.77	8.45E-04	5.08E-03
3303	ENSG00000122550.17	KLHL7	-0.33	8.46E-04	5.08E-03
3304	ENSG00000186283.13	TOR3A	-0.45	8.50E-04	5.11E-03
3305	ENSG00000100207.18	TCF20	0.35	8.51E-04	5.11E-03
3306	ENSG00000259319.1	AF111167.2	-1.12	8.51E-04	5.11E-03
3307	ENSG00000155380.11	SLC16A1	0.33	8.52E-04	5.11E-03
3308	ENSG00000279289.1	AL136164.3	-1.45	8.52E-04	5.11E-03
3309	ENSG00000125817.7	CENPB	-0.37	8.54E-04	5.12E-03
3310	ENSG00000128656.13	CHN1	-0.61	8.54E-04	5.12E-03
3311	ENSG00000164045.11	CDC25A	0.97	8.58E-04	5.15E-03
3312	ENSG00000040487.12	PQLC2	0.50	8.61E-04	5.16E-03
3313	ENSG00000187720.14	THSD4	-0.74	8.61E-04	5.16E-03
3314	ENSG00000102174.8	PHEX	-1.37	8.64E-04	5.17E-03
3315	ENSG00000165617.14	DACT1	-0.42	8.67E-04	5.19E-03
3316	ENSG00000176697.18	BDNF	-0.69	8.67E-04	5.19E-03
3317	ENSG00000178802.17	MPI	0.42	8.71E-04	5.21E-03
3318	ENSG00000198768.10	APCDD1L	-1.10	8.76E-04	5.24E-03
3319	ENSG00000112624.12	BICRAL	-0.47	8.77E-04	5.24E-03
3320	ENSG00000196262.13	PPIA	0.32	8.81E-04	5.27E-03
3321	ENSG00000198937.8	CCDC167	0.48	8.85E-04	5.29E-03
3322	ENSG00000126768.12	TIMM17B	0.48	8.92E-04	5.33E-03
3323	ENSG00000106299.7	WASL	0.31	8.92E-04	5.33E-03
3324	ENSG00000172716.16	SLFN11	-0.57	8.93E-04	5.33E-03
3325	ENSG00000155966.13	AFF2	-0.63	8.92E-04	5.33E-03
3326	ENSG00000131196.17	NFATC1	-0.86	8.93E-04	5.33E-03
3327	ENSG00000123159.15	GIPC1	-0.47	8.98E-04	5.35E-03
3328	ENSG00000165006.13	UBAP1	0.32	9.05E-04	5.40E-03
3329	ENSG00000198682.12	PAPSS2	-0.49	9.06E-04	5.40E-03
3330	ENSG00000273000.5	AP000347.2	1.05	9.09E-04	5.41E-03
3331	ENSG00000049759.17	NEDD4L	0.44	9.09E-04	5.41E-03
3332	ENSG00000158458.19	NRG2	-1.35	9.09E-04	5.41E-03
3333	ENSG00000139718.10	SETD1B	-0.56	9.10E-04	5.42E-03
3334	ENSG00000229214.2	LINC00242	-1.46	9.11E-04	5.43E-03
3335	ENSG00000125611.15	CHCHD5	0.70	9.13E-04	5.43E-03
3336	ENSG00000196547.14	MAN2A2	0.33	9.13E-04	5.43E-03
3337	ENSG00000234420.7	ZNF37BP	-0.54	9.14E-04	5.44E-03
3338	ENSG00000128191.15	DGCR8	-0.38	9.15E-04	5.44E-03
3339	ENSG00000124406.16	ATP8A1	0.78	9.16E-04	5.45E-03
3340	ENSG00000108984.14	MAP2K6	-1.10	9.16E-04	5.45E-03
3341	ENSG00000111880.15	RNGTT	0.36	9.20E-04	5.47E-03
3342	ENSG00000128595.16	CALU	-0.42	9.20E-04	5.47E-03
3343	ENSG00000248527.1	MTATP6P1	0.30	9.25E-04	5.49E-03
3344	ENSG00000225345.3	SNX18P3	-0.86	9.28E-04	5.51E-03
3345	ENSG00000260549.1	MT1L	3.79	9.30E-04	5.52E-03
3346	ENSG00000127022.14	CANX	0.38	9.33E-04	5.53E-03
3347	ENSG00000122126.16	OCRL	0.32	9.33E-04	5.53E-03

3348	ENSG00000163960.11	UBXN7	-0.34	9.36E-04	5.55E-03
3349	ENSG00000146592.16	CREB5	-0.87	9.37E-04	5.55E-03
3350	ENSG00000215252.11	GOLGA8B	-0.41	9.39E-04	5.56E-03
3351	ENSG00000279453.1	Z99129.4	-0.70	9.42E-04	5.58E-03
3352	ENSG00000133056.13	PIK3C2B	-0.46	9.42E-04	5.58E-03
3353	ENSG00000164172.18	MOCS2	0.38	9.43E-04	5.58E-03
3354	ENSG00000148143.12	ZNF462	0.50	9.44E-04	5.59E-03
3355	ENSG00000162430.16	SELENON	-0.39	9.47E-04	5.60E-03
3356	ENSG00000118495.19	PLAGL1	0.35	9.47E-04	5.60E-03
3357	ENSG00000148600.14	CDHR1	0.77	9.49E-04	5.61E-03
3358	ENSG00000169991.10	IFFO2	0.48	9.50E-04	5.61E-03
3359	ENSG00000117481.10	NSUN4	0.36	9.50E-04	5.61E-03
3360	ENSG00000279019.1	AC009090.4	1.65	9.53E-04	5.63E-03
3361	ENSG00000196505.10	GDAP2	0.41	9.56E-04	5.64E-03
3362	ENSG00000092148.12	HECTD1	0.28	9.61E-04	5.67E-03
3363	ENSG00000165185.14	KIAA1958	0.44	9.62E-04	5.68E-03
3364	ENSG00000169246.16	NPIPB3	-0.45	9.63E-04	5.68E-03
3365	ENSG00000167065.13	DUSP18	-0.79	9.65E-04	5.69E-03
3366	ENSG00000131149.18	GSE1	0.39	9.67E-04	5.70E-03
3367	ENSG00000135070.14	ISCA1	0.38	9.67E-04	5.70E-03
3368	ENSG00000129472.14	RAB2B	0.39	9.67E-04	5.70E-03
3369	ENSG00000101825.7	MXRA5	-0.63	9.69E-04	5.71E-03
3370	ENSG00000166974.12	MAPRE2	0.49	9.71E-04	5.72E-03
3371	ENSG00000111110.11	PPM1H	0.68	9.74E-04	5.73E-03
3372	ENSG00000176868.2	AL358781.1	-0.62	9.75E-04	5.74E-03
3373	ENSG00000151413.16	NUBPL	0.41	9.78E-04	5.75E-03
3374	ENSG00000244115.1	DNAJC25-GNG10	0.43	9.82E-04	5.77E-03
3375	ENSG00000173621.8	LRFN4	-1.03	9.82E-04	5.77E-03
3376	ENSG00000148484.17	RSU1	0.30	9.84E-04	5.78E-03
3377	ENSG00000250988.7	SNHG21	1.03	9.86E-04	5.80E-03
3378	ENSG00000185684.14	EP400P1	0.48	9.90E-04	5.82E-03
3379	ENSG00000077238.13	IL4R	0.73	9.97E-04	5.85E-03
3380	ENSG00000169062.14	UPF3A	-0.34	9.97E-04	5.85E-03
3381	ENSG00000167136.6	ENDOG	0.77	9.97E-04	5.86E-03
3382	ENSG00000074319.12	TSG101	0.35	1.00E-03	5.87E-03
3383	ENSG00000143612.20	C1orf43	0.32	1.01E-03	5.92E-03
3384	ENSG00000139269.2	INHBE	0.95	1.01E-03	5.93E-03
3385	ENSG00000136048.13	DRAM1	-0.59	1.01E-03	5.93E-03
3386	ENSG00000082438.15	COBLL1	-0.45	1.01E-03	5.93E-03
3387	ENSG00000166889.13	PATL1	0.36	1.01E-03	5.94E-03
3388	ENSG00000104415.14	WISP1	-0.83	1.02E-03	5.96E-03
3389	ENSG00000090674.15	MCOLN1	0.41	1.02E-03	5.97E-03
3390	ENSG00000151208.16	DLG5	-0.34	1.02E-03	6.00E-03
3391	ENSG00000184564.9	SLITRK6	-0.92	1.02E-03	6.00E-03
3392	ENSG00000242732.4	RTL5	0.38	1.03E-03	6.00E-03
3393	ENSG00000020577.13	SAMD4A	-0.41	1.03E-03	6.01E-03
3394	ENSG00000171385.9	KCND3	-0.47	1.03E-03	6.04E-03
3395	ENSG00000120889.12	TNFRSF10B	-0.39	1.03E-03	6.04E-03
3396	ENSG00000152700.13	SAR1B	0.33	1.03E-03	6.04E-03
3397	ENSG00000135828.11	RNASEL	-0.43	1.04E-03	6.07E-03

3398	ENSG00000138942.15	RNF185	0.32	1.04E-03	6.08E-03
3399	ENSG00000272886.5	DCP1A	0.35	1.04E-03	6.10E-03
3400	ENSG00000165633.12	VSTM4	-0.40	1.04E-03	6.10E-03
3401	ENSG00000214063.10	TSPAN4	-0.57	1.04E-03	6.10E-03
3402	ENSG00000166997.7	CNPY4	-0.52	1.05E-03	6.12E-03
3403	ENSG00000116396.14	KCNC4	-0.89	1.05E-03	6.14E-03
3404	ENSG00000112561.17	TFEB	-0.66	1.05E-03	6.14E-03
3405	ENSG00000064419.13	TNPO3	0.31	1.06E-03	6.15E-03
3406	ENSG00000197329.11	PELI1	0.46	1.06E-03	6.16E-03
3407	ENSG00000126856.14	PRDM7	2.21	1.06E-03	6.20E-03
3408	ENSG00000159840.15	ZYX	-0.41	1.07E-03	6.20E-03
3409	ENSG00000067829.18	IDH3G	0.40	1.07E-03	6.21E-03
3410	ENSG00000182389.19	CACNB4	-0.71	1.07E-03	6.22E-03
3411	ENSG00000182175.14	RGMA	-0.72	1.07E-03	6.22E-03
3412	ENSG00000249125.1	AC093821.1	2.69	1.07E-03	6.24E-03
3413	ENSG00000103569.9	AQP9	-1.79	1.08E-03	6.26E-03
3414	ENSG00000178522.14	AMBN	2.34	1.08E-03	6.27E-03
3415	ENSG00000081760.16	AACS	-0.36	1.08E-03	6.28E-03
3416	ENSG00000122863.5	CHST3	0.42	1.08E-03	6.29E-03
3417	ENSG00000139668.8	WDFY2	-0.52	1.08E-03	6.29E-03
3418	ENSG00000138031.14	ADCY3	-0.60	1.09E-03	6.34E-03
3419	ENSG00000141380.13	SS18	-0.32	1.09E-03	6.34E-03
3420	ENSG00000244675.2	AC108676.1	0.90	1.09E-03	6.35E-03
3421	ENSG00000104047.14	DTWD1	-0.42	1.10E-03	6.36E-03
3422	ENSG00000113721.13	PDGFRB	-0.41	1.10E-03	6.39E-03
3423	ENSG00000198951.11	NAGA	-0.50	1.10E-03	6.40E-03
3424	ENSG00000175536.6	LIPT2	1.36	1.11E-03	6.41E-03
3425	ENSG00000188157.14	AGRN	-0.68	1.11E-03	6.41E-03
3426	ENSG00000136378.14	ADAMTS7	-0.47	1.11E-03	6.43E-03
3427	ENSG00000116663.10	FBXO6	0.61	1.11E-03	6.43E-03
3428	ENSG00000053254.15	FOXN3	0.32	1.12E-03	6.47E-03
3429	ENSG00000170962.12	PDGFD	-0.55	1.12E-03	6.47E-03
3430	ENSG00000100483.13	VCPKMT	-0.63	1.12E-03	6.49E-03
3431	ENSG00000149506.11	ZP1	3.49	1.12E-03	6.49E-03
3432	ENSG00000203778.7	FAM229B	-0.55	1.13E-03	6.52E-03
3433	ENSG00000003137.8	CYP26B1	0.74	1.13E-03	6.54E-03
3434	ENSG00000133398.3	MED10	0.40	1.13E-03	6.55E-03
3435	ENSG00000101425.13	BPI	3.60	1.13E-03	6.56E-03
3436	ENSG00000105011.8	ASF1B	1.20	1.14E-03	6.56E-03
3437	ENSG00000119632.3	IFI27L2	-0.58	1.14E-03	6.57E-03
3438	ENSG00000214194.8	SMIM30	-0.44	1.14E-03	6.57E-03
3439	ENSG00000230091.6	TMEM254-AS1	0.78	1.14E-03	6.58E-03
3440	ENSG00000125864.13	BFSP1	1.00	1.14E-03	6.59E-03
3441	ENSG00000197857.13	ZNF44	0.44	1.14E-03	6.59E-03
3442	ENSG00000008710.19	PKD1	-0.55	1.14E-03	6.60E-03
3443	ENSG00000255443.1	CD44-AS1	1.30	1.15E-03	6.61E-03
3444	ENSG00000132300.18	PTCD3	0.33	1.15E-03	6.62E-03
3445	ENSG00000236740.6	AL033384.1	3.08	1.15E-03	6.62E-03
3446	ENSG00000070444.14	MNT	-0.47	1.16E-03	6.66E-03
3447	ENSG00000065978.18	YBX1	0.32	1.16E-03	6.67E-03

3448	ENSG00000103966.10	EHD4	0.41	1.16E-03	6.67E-03
3449	ENSG00000120471.15	TP53AIP1	3.71	1.16E-03	6.68E-03
3450	ENSG00000198513.11	ATL1	-0.65	1.16E-03	6.69E-03
3451	ENSG00000101255.10	TRIB3	-0.49	1.17E-03	6.71E-03
3452	ENSG00000100612.13	DHRS7	0.35	1.17E-03	6.74E-03
3453	ENSG00000085433.15	WDR47	-0.42	1.18E-03	6.76E-03
3454	ENSG00000243147.7	MRPL33	0.51	1.18E-03	6.76E-03
3455	ENSG00000172020.12	GAP43	-0.80	1.18E-03	6.76E-03
3456	ENSG00000159131.16	GART	0.33	1.18E-03	6.77E-03
3457	ENSG00000120725.12	SIL1	0.39	1.19E-03	6.81E-03
3458	ENSG00000124831.18	LRRFIP1	-0.37	1.19E-03	6.82E-03
3459	ENSG00000167658.15	EEF2	0.36	1.20E-03	6.87E-03
3460	ENSG00000277559.1	AC018553.1	-1.55	1.20E-03	6.87E-03
3461	ENSG00000164930.11	FZD6	-0.37	1.20E-03	6.87E-03
3462	ENSG00000186462.8	NAP1L2	-2.04	1.20E-03	6.89E-03
3463	ENSG00000228495.1	LINC01013	-0.70	1.21E-03	6.92E-03
3464	ENSG00000060642.10	PIGV	0.42	1.21E-03	6.94E-03
3465	ENSG00000103257.8	SLC7A5	-0.49	1.21E-03	6.94E-03
3466	ENSG00000279955.3	AC129778.1	2.12	1.21E-03	6.95E-03
3467	ENSG00000125386.15	FAM193A	-0.39	1.21E-03	6.95E-03
3468	ENSG00000118762.7	PKD2	-0.40	1.22E-03	6.96E-03
3469	ENSG00000113048.16	MRPS27	0.33	1.22E-03	6.97E-03
3470	ENSG00000143889.15	HNRNPLL	-0.40	1.22E-03	6.98E-03
3471	ENSG00000105137.12	SYDE1	-0.47	1.22E-03	6.98E-03
3472	ENSG00000130584.10	ZBTB46	0.76	1.23E-03	7.01E-03
3473	ENSG00000012660.13	ELOVL5	-0.30	1.23E-03	7.02E-03
3474	ENSG00000112837.16	TBX18	-0.42	1.23E-03	7.02E-03
3475	ENSG00000105677.11	TMEM147	0.34	1.23E-03	7.05E-03
3476	ENSG00000270212.1	AC008738.6	3.26	1.24E-03	7.07E-03
3477	ENSG00000260792.1	LINC02280	1.64	1.24E-03	7.08E-03
3478	ENSG00000148843.14	PDCD11	0.39	1.24E-03	7.08E-03
3479	ENSG00000149131.15	SERPING1	-0.39	1.25E-03	7.12E-03
3480	ENSG00000166734.19	CASC4	-0.41	1.25E-03	7.12E-03
3481	ENSG00000106809.10	OGN	-0.46	1.25E-03	7.15E-03
3482	ENSG00000196950.13	SLC39A10	-0.42	1.26E-03	7.16E-03
3483	ENSG00000183955.12	KMT5A	-0.34	1.26E-03	7.16E-03
3484	ENSG00000090097.21	PCBP4	-0.48	1.26E-03	7.16E-03
3485	ENSG00000136247.14	ZDHHC4	0.33	1.26E-03	7.16E-03
3486	ENSG00000140332.15	TLE3	-0.53	1.26E-03	7.17E-03
3487	ENSG00000064932.15	SBNO2	-0.55	1.26E-03	7.17E-03
3488	ENSG00000180998.11	GPR137C	0.59	1.26E-03	7.18E-03
3489	ENSG00000100813.14	ACIN1	-0.30	1.26E-03	7.18E-03
3490	ENSG00000181026.14	AEN	-0.42	1.26E-03	7.18E-03
3491	ENSG00000064309.14	CDON	-0.41	1.27E-03	7.23E-03
3492	ENSG00000224259.6	LINC01133	-0.49	1.28E-03	7.26E-03
3493	ENSG00000237961.4	AC118282.1	-2.68	1.28E-03	7.26E-03
3494	ENSG00000137094.14	DNAJB5	-0.49	1.28E-03	7.27E-03
3495	ENSG00000079689.13	SCGN	2.60	1.28E-03	7.27E-03
3496	ENSG00000267216.1	AC020915.1	-1.62	1.28E-03	7.27E-03
3497	ENSG00000196498.13	NCOR2	-0.54	1.28E-03	7.27E-03

3498	ENSG00000104231.10	ZFAND1	0.48	1.29E-03	7.30E-03
3499	ENSG00000100139.13	MICALL1	0.37	1.29E-03	7.31E-03
3500	ENSG00000148356.13	LRSAM1	-0.52	1.29E-03	7.32E-03
3501	ENSG00000214026.10	MRPL23	0.52	1.29E-03	7.33E-03
3502	ENSG00000277287.1	AL109976.1	-1.17	1.30E-03	7.36E-03
3503	ENSG00000198001.13	IRAK4	-0.49	1.30E-03	7.37E-03
3504	ENSG00000179295.17	PTPN11	0.31	1.30E-03	7.37E-03
3505	ENSG00000101444.12	AHCY	0.37	1.31E-03	7.39E-03
3506	ENSG00000142149.8	HUNK	0.87	1.31E-03	7.40E-03
3507	ENSG00000162144.9	CYB561A3	-0.43	1.31E-03	7.40E-03
3508	ENSG00000169045.17	HNRNPH1	-0.33	1.31E-03	7.41E-03
3509	ENSG00000164587.12	RPS14	0.33	1.31E-03	7.41E-03
3510	ENSG00000104687.13	GSR	0.33	1.31E-03	7.41E-03
3511	ENSG00000083544.14	TDRD3	-0.45	1.31E-03	7.41E-03
3512	ENSG00000079332.14	SAR1A	0.36	1.31E-03	7.42E-03
3513	ENSG00000276266.1	FP325318.1	1.69	1.32E-03	7.43E-03
3514	ENSG00000151422.12	FER	-0.35	1.32E-03	7.45E-03
3515	ENSG00000104691.14	UBXN8	0.51	1.32E-03	7.45E-03
3516	ENSG00000070269.13	TMEM260	-0.42	1.32E-03	7.46E-03
3517	ENSG00000162104.9	ADCY9	-0.46	1.32E-03	7.46E-03
3518	ENSG00000171763.18	SPATA5L1	0.61	1.32E-03	7.47E-03
3519	ENSG00000273179.1	AC092535.4	-1.90	1.33E-03	7.48E-03
3520	ENSG00000138777.19	PPA2	0.33	1.33E-03	7.48E-03
3521	ENSG00000138190.16	EXOC6	0.57	1.33E-03	7.50E-03
3522	ENSG00000108813.10	DLX4	3.05	1.33E-03	7.50E-03
3523	ENSG00000240764.3	PCDHGC5	-1.53	1.34E-03	7.53E-03
3524	ENSG00000183935.5	HTR7P1	-0.55	1.34E-03	7.55E-03
3525	ENSG00000122970.15	IFT81	-0.47	1.34E-03	7.56E-03
3526	ENSG00000166153.16	DEPDC4	1.27	1.35E-03	7.62E-03
3527	ENSG00000174915.11	PTDSS2	0.43	1.35E-03	7.62E-03
3528	ENSG00000243137.7	PSG4	-0.74	1.37E-03	7.68E-03
3529	ENSG00000228782.7	MRPL45P2	0.74	1.37E-03	7.68E-03
3530	ENSG00000247121.6	AC009126.1	1.00	1.37E-03	7.70E-03
3531	ENSG00000152620.12	NADK2	0.32	1.37E-03	7.70E-03
3532	ENSG00000008988.9	RPS20	0.32	1.37E-03	7.71E-03
3533	ENSG00000267924.1	AC139769.2	2.30	1.38E-03	7.73E-03
3534	ENSG00000234585.6	CCT6P3	0.45	1.38E-03	7.76E-03
3535	ENSG00000169371.13	SNUPN	-0.39	1.38E-03	7.78E-03
3536	ENSG00000245685.6	FRG1-DT	1.48	1.39E-03	7.78E-03
3537	ENSG00000138336.8	TET1	-0.59	1.39E-03	7.79E-03
3538	ENSG00000130396.20	AFDN	0.37	1.39E-03	7.81E-03
3539	ENSG00000036054.12	TBC1D23	-0.38	1.40E-03	7.85E-03
3540	ENSG00000101333.16	PLCB4	-0.78	1.41E-03	7.88E-03
3541	ENSG00000256013.1	AC027277.1	3.43	1.41E-03	7.89E-03
3542	ENSG00000139112.10	GABARAPL1	-0.37	1.41E-03	7.89E-03
3543	ENSG00000168386.18	FILIP1L	-0.52	1.42E-03	7.94E-03
3544	ENSG00000255050.1	AC067930.5	2.41	1.42E-03	7.97E-03
3545	ENSG00000164010.14	ERMAP	-0.38	1.43E-03	8.00E-03
3546	ENSG00000187109.13	NAP1L1	-0.43	1.43E-03	8.00E-03
3547	ENSG00000228492.2	RAB11FIP1P1	-0.67	1.43E-03	8.01E-03

3548	ENSG00000243156.7	MICAL3	0.47	1.44E-03	8.03E-03
3549	ENSG00000142453.11	CARM1	-0.37	1.44E-03	8.08E-03
3550	ENSG00000251562.8	MALAT1	-0.38	1.45E-03	8.08E-03
3551	ENSG00000205213.13	LGR4	-0.29	1.45E-03	8.08E-03
3552	ENSG00000254109.5	RBPMS-AS1	0.96	1.45E-03	8.08E-03
3553	ENSG00000171786.5	NHLH1	0.51	1.45E-03	8.09E-03
3554	ENSG00000138172.10	CALHM2	-0.62	1.45E-03	8.09E-03
3555	ENSG00000120075.5	HOXB5	-0.58	1.46E-03	8.17E-03
3556	ENSG00000229642.1	AC087857.1	3.34	1.46E-03	8.17E-03
3557	ENSG00000264006.8	AKR1C8P	-2.17	1.46E-03	8.17E-03
3558	ENSG00000054267.21	ARID4B	-0.33	1.47E-03	8.19E-03
3559	ENSG00000162980.16	ARL5A	0.34	1.47E-03	8.21E-03
3560	ENSG00000076685.18	NT5C2	-0.35	1.48E-03	8.23E-03
3561	ENSG00000165060.12	FXN	0.47	1.49E-03	8.29E-03
3562	ENSG00000085871.8	MGST2	0.45	1.49E-03	8.31E-03
3563	ENSG00000084636.17	COL16A1	-0.52	1.49E-03	8.31E-03
3564	ENSG00000262814.7	MRPL12	0.43	1.50E-03	8.33E-03
3565	ENSG00000158169.13	FANCC	-0.57	1.50E-03	8.33E-03
3566	ENSG00000271601.3	LIX1L	-0.34	1.51E-03	8.39E-03
3567	ENSG00000073969.18	NSF	-0.32	1.51E-03	8.41E-03
3568	ENSG00000215717.5	TMEM167B	-0.30	1.51E-03	8.42E-03
3569	ENSG00000122641.10	INHBA	-0.36	1.51E-03	8.42E-03
3570	ENSG00000146414.15	SHPRH	0.37	1.52E-03	8.47E-03
3571	ENSG00000174021.10	GNG5	0.36	1.52E-03	8.47E-03
3572	ENSG00000221968.8	FADS3	-0.41	1.53E-03	8.47E-03
3573	ENSG00000102007.10	PLP2	-0.73	1.53E-03	8.47E-03
3574	ENSG00000143033.17	MTF2	-0.46	1.53E-03	8.49E-03
3575	ENSG00000178295.14	GEN1	0.36	1.53E-03	8.50E-03
3576	ENSG00000278122.1	AQP7P5	4.87	1.54E-03	8.54E-03
3577	ENSG00000238083.7	LRRC37A2	-0.39	1.54E-03	8.54E-03
3578	ENSG00000177283.7	FZD8	-0.61	1.54E-03	8.55E-03
3579	ENSG00000166546.13	BEAN1	-1.46	1.54E-03	8.55E-03
3580	ENSG00000168961.16	LGALS9	-1.48	1.54E-03	8.55E-03
3581	ENSG00000110811.19	P3H3	-0.50	1.54E-03	8.56E-03
3582	ENSG00000173950.15	XXYLT1	-0.36	1.54E-03	8.56E-03
3583	ENSG00000162377.5	COA7	0.41	1.55E-03	8.56E-03
3584	ENSG00000172493.20	AFF1	0.31	1.55E-03	8.58E-03
3585	ENSG00000176771.16	NCKAP5	-0.85	1.55E-03	8.59E-03
3586	ENSG00000164463.12	CREBRF	-0.37	1.55E-03	8.59E-03
3587	ENSG00000134250.19	NOTCH2	-0.36	1.56E-03	8.61E-03
3588	ENSG00000204852.15	TCTN1	-0.41	1.56E-03	8.62E-03
3589	ENSG00000178385.14	PLEKHM3	0.33	1.56E-03	8.63E-03
3590	ENSG00000204923.3	FBXO48	0.73	1.56E-03	8.64E-03
3591	ENSG00000142082.14	SIRT3	0.43	1.57E-03	8.66E-03
3592	ENSG00000107249.22	GLIS3	-0.59	1.57E-03	8.67E-03
3593	ENSG00000205423.11	CNEP1R1	-0.45	1.58E-03	8.73E-03
3594	ENSG00000126787.12	DLGAP5	-2.57	1.58E-03	8.74E-03
3595	ENSG00000234944.1	AL365199.1	1.13	1.58E-03	8.74E-03
3596	ENSG00000081791.8	DELE1	0.32	1.59E-03	8.76E-03
3597	ENSG00000251442.5	LINC01094	-1.90	1.59E-03	8.77E-03

3598	ENSG00000116874.11	WARS2	0.40	1.59E-03	8.78E-03
3599	ENSG00000173421.16	CCDC36	0.49	1.60E-03	8.80E-03
3600	ENSG00000177733.6	HNRNPA0	-0.28	1.60E-03	8.80E-03
3601	ENSG00000125484.11	GTF3C4	-0.35	1.60E-03	8.81E-03
3602	ENSG00000169105.7	CHST14	-0.54	1.60E-03	8.83E-03
3603	ENSG00000148948.7	LRRC4C	0.56	1.61E-03	8.87E-03
3604	ENSG00000171680.21	PLEKHG5	-0.76	1.61E-03	8.88E-03
3605	ENSG00000049167.14	ERCC8	0.44	1.62E-03	8.90E-03
3606	ENSG00000197982.13	C1orf122	-0.40	1.62E-03	8.91E-03
3607	ENSG00000198829.6	SUCNR1	1.57	1.62E-03	8.92E-03
3608	ENSG00000249790.2	AC092490.1	1.03	1.63E-03	8.97E-03
3609	ENSG00000164867.10	NOS3	-1.02	1.64E-03	9.01E-03
3610	ENSG00000205726.14	ITSN1	0.31	1.64E-03	9.03E-03
3611	ENSG00000131002.11	TXLNGY	-0.38	1.64E-03	9.04E-03
3612	ENSG00000101040.19	ZMYND8	-0.43	1.65E-03	9.06E-03
3613	ENSG00000106772.17	PRUNE2	-0.42	1.66E-03	9.13E-03
3614	ENSG00000285839.1	AL445685.3	-0.51	1.66E-03	9.14E-03
3615	ENSG00000166855.9	CLPX	0.29	1.67E-03	9.15E-03
3616	ENSG00000206341.7	HLA-H	0.85	1.67E-03	9.15E-03
3617	ENSG00000206561.12	COLQ	-0.76	1.67E-03	9.17E-03
3618	ENSG00000213719.8	CLIC1	-0.38	1.68E-03	9.23E-03
3619	ENSG00000184719.11	RNLS	-0.51	1.68E-03	9.23E-03
3620	ENSG00000037897.16	METTL1	0.54	1.69E-03	9.24E-03
3621	ENSG00000064989.12	CALCRL	0.39	1.69E-03	9.28E-03
3622	ENSG00000204311.13	PJVK	-0.71	1.69E-03	9.28E-03
3623	ENSG00000164054.15	SHISA5	-0.37	1.70E-03	9.29E-03
3624	ENSG00000165801.9	ARHGEF40	0.35	1.70E-03	9.30E-03
3625	ENSG00000164035.9	EMCN	-0.86	1.70E-03	9.32E-03
3626	ENSG00000240758.2	AC010655.2	0.51	1.71E-03	9.34E-03
3627	ENSG00000265511.1	AC020558.2	2.12	1.71E-03	9.35E-03
3628	ENSG00000206129.4	AC006305.1	1.24	1.73E-03	9.44E-03
3629	ENSG00000119231.10	SENP5	0.32	1.73E-03	9.45E-03
3630	ENSG00000110931.18	CAMKK2	0.30	1.73E-03	9.45E-03
3631	ENSG00000179941.7	BBS10	-0.44	1.73E-03	9.45E-03
3632	ENSG00000121671.11	CRY2	0.38	1.73E-03	9.46E-03
3633	ENSG00000146072.6	TNFRSF21	0.34	1.74E-03	9.50E-03
3634	ENSG00000151729.10	SLC25A4	0.36	1.74E-03	9.52E-03
3635	ENSG00000239305.6	RNF103	-0.35	1.75E-03	9.54E-03
3636	ENSG00000143314.12	MRPL24	0.36	1.75E-03	9.56E-03
3637	ENSG00000225032.5	AL162586.1	-1.12	1.76E-03	9.60E-03
3638	ENSG00000136504.11	KAT7	-0.35	1.76E-03	9.60E-03
3639	ENSG00000205002.3	AARD	-0.65	1.76E-03	9.60E-03
3640	ENSG00000177225.16	GATD1	-0.36	1.76E-03	9.61E-03
3641	ENSG00000128185.9	DGCR6L	0.46	1.77E-03	9.63E-03
3642	ENSG00000173153.13	ESRRA	0.37	1.77E-03	9.63E-03
3643	ENSG00000280064.1	AC130304.1	-1.68	1.78E-03	9.67E-03
3644	ENSG00000108312.14	UBTF	-0.34	1.78E-03	9.68E-03
3645	ENSG00000153002.11	CPB1	-0.64	1.78E-03	9.69E-03
3646	ENSG00000167216.16	KATNAL2	-0.65	1.78E-03	9.71E-03
3647	ENSG00000166484.19	MAPK7	-0.53	1.79E-03	9.72E-03

3648	ENSG00000131067.16	GGT7	-0.53	1.79E-03	9.76E-03
3649	ENSG00000204149.10	AGAP6	-0.40	1.80E-03	9.77E-03
3650	ENSG00000162341.16	TPCN2	-0.44	1.80E-03	9.78E-03
3651	ENSG00000131236.16	CAP1	-0.31	1.80E-03	9.78E-03
3652	ENSG00000105711.11	SCN1B	-0.90	1.80E-03	9.78E-03
3653	ENSG00000277701.4	AC159540.2	-0.65	1.80E-03	9.81E-03
3654	ENSG00000233334.3	FAM53B-AS1	1.56	1.81E-03	9.82E-03
3655	ENSG00000171812.12	COL8A2	-0.57	1.81E-03	9.82E-03
3656	ENSG00000117155.16	SSX2IP	-0.46	1.81E-03	9.82E-03
3657	ENSG00000235750.9	KIAA0040	-1.14	1.81E-03	9.84E-03
3658	ENSG00000115380.19	EFEMP1	-0.96	1.82E-03	9.87E-03
3659	ENSG00000155463.12	OXA1L	0.29	1.82E-03	9.87E-03
3660	ENSG00000167291.15	TBC1D16	0.38	1.85E-03	1.00E-02
3661	ENSG00000188554.13	NBR1	-0.30	1.85E-03	1.00E-02
3662	ENSG00000151789.11	ZNF385D	-0.85	1.85E-03	1.00E-02
3663	ENSG00000138867.16	GUCD1	0.32	1.85E-03	1.00E-02
3664	ENSG00000067182.7	TNFRSF1A	-0.36	1.86E-03	1.01E-02
3665	ENSG00000119900.8	OGFRL1	0.35	1.86E-03	1.01E-02
3666	ENSG00000187372.11	PCDHB13	-0.69	1.86E-03	1.01E-02
3667	ENSG00000172159.15	FRMD3	-0.96	1.87E-03	1.01E-02
3668	ENSG00000235374.2	SSR4P1	-1.19	1.88E-03	1.01E-02
3669	ENSG00000183696.13	UPP1	-0.73	1.88E-03	1.02E-02
3670	ENSG00000101353.14	MROH8	0.67	1.88E-03	1.02E-02
3671	ENSG00000120253.13	NUP43	-0.38	1.88E-03	1.02E-02
3672	ENSG00000144746.6	ARL6IP5	0.28	1.88E-03	1.02E-02
3673	ENSG00000165084.15	C8orf34	-1.21	1.88E-03	1.02E-02
3674	ENSG00000158467.16	AHCYL2	0.39	1.89E-03	1.02E-02
3675	ENSG00000110048.11	OSBP	0.28	1.89E-03	1.02E-02
3676	ENSG00000151465.13	CDC123	-0.36	1.90E-03	1.03E-02
3677	ENSG00000239779.6	WBP1	0.34	1.90E-03	1.03E-02
3678	ENSG00000035141.7	FAM136A	0.36	1.91E-03	1.03E-02
3679	ENSG00000106948.16	AKNA	0.41	1.91E-03	1.03E-02
3680	ENSG00000084073.8	ZMPSTE24	-0.31	1.91E-03	1.03E-02
3681	ENSG00000163577.7	EIF5A2	-0.48	1.91E-03	1.03E-02
3682	ENSG00000151445.15	VIPAS39	-0.36	1.91E-03	1.03E-02
3683	ENSG00000259291.2	ZNF710-AS1	-0.62	1.91E-03	1.03E-02
3684	ENSG00000079257.7	LXN	-0.69	1.91E-03	1.03E-02
3685	ENSG00000245281.7	AC124242.1	0.98	1.92E-03	1.03E-02
3686	ENSG00000119723.16	COQ6	0.37	1.92E-03	1.03E-02
3687	ENSG00000065308.4	TRAM2	-0.42	1.92E-03	1.04E-02
3688	ENSG00000254912.2	AC135983.3	2.19	1.93E-03	1.04E-02
3689	ENSG00000007384.15	RHBDF1	-0.46	1.93E-03	1.04E-02
3690	ENSG00000251301.6	LINC02384	1.57	1.93E-03	1.04E-02
3691	ENSG00000259969.1	AL049838.1	-1.36	1.94E-03	1.04E-02
3692	ENSG00000261799.1	AC007406.5	-0.54	1.94E-03	1.04E-02
3693	ENSG00000253537.2	PCDHGA7	-0.58	1.95E-03	1.05E-02
3694	ENSG00000187840.4	EIF4EBP1	0.47	1.95E-03	1.05E-02
3695	ENSG00000283341.1	AC068205.2	0.90	1.95E-03	1.05E-02
3696	ENSG00000184014.7	DENND5A	0.28	1.95E-03	1.05E-02
3697	ENSG00000184867.13	ARMCX2	-0.46	1.96E-03	1.05E-02

3698	ENSG00000180801.13	ARSJ	-0.59	1.97E-03	1.06E-02
3699	ENSG00000088986.10	DYNLL1	0.33	1.98E-03	1.06E-02
3700	ENSG00000197894.10	ADH5	-0.33	1.98E-03	1.06E-02
3701	ENSG00000146830.10	GIGYF1	-0.36	1.98E-03	1.06E-02
3702	ENSG00000063046.17	EIF4B	0.29	1.99E-03	1.07E-02
3703	ENSG00000243716.10	NPIPB5	-0.38	2.00E-03	1.07E-02
3704	ENSG00000158716.8	DUSP23	0.52	2.00E-03	1.07E-02
3705	ENSG00000267322.2	SNHG22	1.33	2.01E-03	1.07E-02
3706	ENSG00000143753.12	DEGS1	-0.29	2.01E-03	1.08E-02
3707	ENSG00000179603.17	GRM8	1.93	2.02E-03	1.08E-02
3708	ENSG00000203497.2	PDCD4-AS1	1.10	2.02E-03	1.08E-02
3709	ENSG00000134874.17	DZIP1	-0.40	2.02E-03	1.08E-02
3710	ENSG00000181104.6	F2R	-0.47	2.02E-03	1.08E-02
3711	ENSG00000004864.13	SLC25A13	0.37	2.03E-03	1.08E-02
3712	ENSG00000249994.1	AC025187.1	1.99	2.03E-03	1.08E-02
3713	ENSG00000138821.12	SLC39A8	0.60	2.03E-03	1.09E-02
3714	ENSG00000006831.9	ADIPOR2	0.33	2.05E-03	1.09E-02
3715	ENSG00000134046.11	MBD2	-0.30	2.05E-03	1.10E-02
3716	ENSG00000144597.13	EAF1	0.36	2.05E-03	1.10E-02
3717	ENSG00000114395.10	CYB561D2	0.51	2.06E-03	1.10E-02
3718	ENSG00000167123.18	CERCAM	-0.47	2.07E-03	1.11E-02
3719	ENSG00000150712.10	MTMR12	0.40	2.08E-03	1.11E-02
3720	ENSG00000254505.9	CHMP4A	0.36	2.08E-03	1.11E-02
3721	ENSG00000100580.7	TMED8	0.40	2.09E-03	1.11E-02
3722	ENSG00000158406.4	HIST1H4H	-0.63	2.09E-03	1.11E-02
3723	ENSG00000164209.16	SLC25A46	0.29	2.09E-03	1.12E-02
3724	ENSG00000127528.5	KLF2	-0.77	2.09E-03	1.12E-02
3725	ENSG00000265158.1	LRRC37A7P	0.83	2.10E-03	1.12E-02
3726	ENSG00000097096.8	SYDE2	0.40	2.10E-03	1.12E-02
3727	ENSG00000107130.9	NCS1	-0.52	2.10E-03	1.12E-02
3728	ENSG00000064042.17	LIMCH1	-0.30	2.11E-03	1.12E-02
3729	ENSG00000114115.9	RBP1	0.74	2.12E-03	1.13E-02
3730	ENSG00000166912.16	MTMR10	0.35	2.12E-03	1.13E-02
3731	ENSG00000141542.10	RAB40B	-0.52	2.12E-03	1.13E-02
3732	ENSG00000144040.12	SFXN5	0.43	2.13E-03	1.13E-02
3733	ENSG00000188582.8	PAQR9	1.66	2.13E-03	1.13E-02
3734	ENSG00000104529.17	EEF1D	0.40	2.13E-03	1.13E-02
3735	ENSG00000186868.15	MAPT	0.44	2.14E-03	1.14E-02
3736	ENSG00000183793.13	NPIPA5	-0.71	2.14E-03	1.14E-02
3737	ENSG00000168016.14	TRANK1	-0.47	2.14E-03	1.14E-02
3738	ENSG00000073536.17	NLE1	0.54	2.15E-03	1.14E-02
3739	ENSG00000152782.16	PANK1	0.39	2.15E-03	1.14E-02
3740	ENSG00000096092.5	TMEM14A	-0.58	2.15E-03	1.14E-02
3741	ENSG00000159403.16	C1R	-0.43	2.15E-03	1.14E-02
3742	ENSG00000027075.14	PRKCH	0.87	2.16E-03	1.14E-02
3743	ENSG00000109062.11	SLC9A3R1	-0.74	2.16E-03	1.15E-02
3744	ENSG00000225733.5	FGD5-AS1	-0.30	2.16E-03	1.15E-02
3745	ENSG00000233048.1	LINC01722	1.08	2.17E-03	1.15E-02
3746	ENSG00000136244.11	IL6	-0.81	2.17E-03	1.15E-02
3747	ENSG00000237976.1	AL391069.3	1.42	2.17E-03	1.15E-02

3748	ENSG00000111799.20	COL12A1	-0.33	2.17E-03	1.15E-02
3749	ENSG00000106714.17	CNTNAP3	1.11	2.17E-03	1.15E-02
3750	ENSG00000154767.14	XPC	-0.38	2.17E-03	1.15E-02
3751	ENSG00000271367.1	AL034374.1	0.69	2.18E-03	1.15E-02
3752	ENSG00000148090.11	AUH	0.39	2.18E-03	1.15E-02
3753	ENSG00000160688.18	FLAD1	0.36	2.19E-03	1.16E-02
3754	ENSG00000172638.12	EFEMP2	-0.42	2.20E-03	1.16E-02
3755	ENSG00000196449.3	YRDC	0.46	2.20E-03	1.16E-02
3756	ENSG00000172301.10	COPRS	-0.34	2.20E-03	1.17E-02
3757	ENSG00000223509.8	AC135983.2	0.44	2.21E-03	1.17E-02
3758	ENSG00000241878.11	PISD	0.39	2.21E-03	1.17E-02
3759	ENSG00000268129.1	AC026304.1	1.07	2.21E-03	1.17E-02
3760	ENSG00000151364.16	KCTD14	-1.51	2.21E-03	1.17E-02
3761	ENSG00000164291.16	ARSK	-0.54	2.22E-03	1.17E-02
3762	ENSG00000103275.19	UBE2I	-0.32	2.22E-03	1.17E-02
3763	ENSG00000007392.16	LUC7L	-0.35	2.23E-03	1.17E-02
3764	ENSG00000230606.10	AC092683.1	-0.42	2.23E-03	1.17E-02
3765	ENSG00000161381.13	PLXDC1	-0.54	2.23E-03	1.17E-02
3766	ENSG00000168398.6	BDKRB2	-1.42	2.23E-03	1.17E-02
3767	ENSG00000147274.14	RBMX	-0.38	2.23E-03	1.18E-02
3768	ENSG00000068615.18	REEP1	2.08	2.24E-03	1.18E-02
3769	ENSG00000113845.9	TIMMDC1	0.32	2.24E-03	1.18E-02
3770	ENSG00000170144.20	HNRNPA3	-0.30	2.24E-03	1.18E-02
3771	ENSG00000100345.21	MYH9	-0.38	2.24E-03	1.18E-02
3772	ENSG00000170145.4	SIK2	0.29	2.25E-03	1.18E-02
3773	ENSG00000274605.1	AL355338.1	0.68	2.25E-03	1.18E-02
3774	ENSG00000108306.12	FBXL20	-0.35	2.25E-03	1.19E-02
3775	ENSG00000197183.14	NOL4L	-0.49	2.26E-03	1.19E-02
3776	ENSG00000129474.15	AJUBA	-0.40	2.26E-03	1.19E-02
3777	ENSG00000198099.8	ADH4	1.11	2.27E-03	1.19E-02
3778	ENSG00000197614.10	MFAP5	-0.37	2.28E-03	1.20E-02
3779	ENSG00000235806.1	AF241728.1	1.13	2.28E-03	1.20E-02
3780	ENSG00000135046.13	ANXA1	-0.49	2.28E-03	1.20E-02
3781	ENSG00000182541.17	LIMK2	0.34	2.28E-03	1.20E-02
3782	ENSG00000107731.12	UNC5B	-0.58	2.32E-03	1.22E-02
3783	ENSG00000182310.14	SPACA6	-0.81	2.32E-03	1.22E-02
3784	ENSG00000136490.8	LIMD2	-1.20	2.32E-03	1.22E-02
3785	ENSG00000261654.1	AL360270.2	0.59	2.32E-03	1.22E-02
3786	ENSG00000112799.8	LY86	-1.51	2.32E-03	1.22E-02
3787	ENSG00000130173.13	ANGPTL8	0.67	2.33E-03	1.22E-02
3788	ENSG00000158195.10	WASF2	-0.32	2.33E-03	1.22E-02
3789	ENSG00000130985.16	UBA1	-0.31	2.33E-03	1.22E-02
3790	ENSG00000196411.9	EPHB4	-0.50	2.34E-03	1.23E-02
3791	ENSG00000120093.11	HOXB3	-0.36	2.35E-03	1.23E-02
3792	ENSG00000203288.3	TDRKH-AS1	1.92	2.35E-03	1.23E-02
3793	ENSG00000136159.3	NUDT15	0.41	2.35E-03	1.23E-02
3794	ENSG00000100003.17	SEC14L2	-0.84	2.35E-03	1.23E-02
3795	ENSG00000163359.15	COL6A3	-0.52	2.36E-03	1.23E-02
3796	ENSG00000165209.18	STRBP	0.42	2.36E-03	1.23E-02
3797	ENSG00000144218.18	AFF3	-0.46	2.36E-03	1.23E-02

3798	ENSG00000127774.6	EMC6	0.48	2.36E-03	1.23E-02
3799	ENSG00000266553.2	RN7SL356P	3.04	2.37E-03	1.24E-02
3800	ENSG00000258441.1	LINC00641	-0.45	2.37E-03	1.24E-02
3801	ENSG00000138311.16	ZNF365	-1.08	2.38E-03	1.24E-02
3802	ENSG00000132718.8	SYT11	-0.46	2.38E-03	1.24E-02
3803	ENSG00000175602.3	CCDC85B	0.68	2.38E-03	1.24E-02
3804	ENSG00000090006.17	LTBP4	-0.56	2.38E-03	1.24E-02
3805	ENSG00000114861.20	FOXP1	-0.38	2.40E-03	1.25E-02
3806	ENSG00000149016.15	TUT1	0.57	2.41E-03	1.26E-02
3807	ENSG00000130635.15	COL5A1	-0.38	2.41E-03	1.26E-02
3808	ENSG00000182220.14	ATP6AP2	-0.34	2.41E-03	1.26E-02
3809	ENSG00000129250.11	KIF1C	-0.28	2.41E-03	1.26E-02
3810	ENSG00000167664.8	TMIGD2	1.58	2.42E-03	1.26E-02
3811	ENSG00000115750.16	TAF1B	0.50	2.42E-03	1.26E-02
3812	ENSG00000177839.6	PCDHB9	-1.02	2.42E-03	1.26E-02
3813	ENSG00000162757.4	C1orf74	0.70	2.43E-03	1.26E-02
3814	ENSG00000218739.9	CEBPZOS	0.30	2.43E-03	1.26E-02
3815	ENSG00000186350.10	RXRA	-0.36	2.45E-03	1.27E-02
3816	ENSG00000154269.14	ENPP3	1.26	2.46E-03	1.28E-02
3817	ENSG00000151276.23	MAGI1	-0.37	2.46E-03	1.28E-02
3818	ENSG00000008441.16	NFIX	-0.34	2.46E-03	1.28E-02
3819	ENSG00000135931.17	ARMC9	-0.52	2.47E-03	1.28E-02
3820	ENSG00000123415.15	SMUG1	0.37	2.47E-03	1.28E-02
3821	ENSG00000175265.17	GOLGA8A	-0.31	2.47E-03	1.28E-02
3822	ENSG00000224950.2	AL390066.1	-1.67	2.48E-03	1.29E-02
3823	ENSG00000133872.13	SARAF	0.26	2.48E-03	1.29E-02
3824	ENSG00000141425.17	RPRD1A	0.36	2.49E-03	1.29E-02
3825	ENSG00000149451.17	ADAM33	-0.51	2.49E-03	1.29E-02
3826	ENSG00000141030.12	COPS3	0.31	2.49E-03	1.29E-02
3827	ENSG00000143147.14	GPR161	-0.48	2.49E-03	1.29E-02
3828	ENSG00000111860.13	CEP85L	-0.48	2.50E-03	1.30E-02
3829	ENSG00000250786.1	SNHG18	0.48	2.51E-03	1.30E-02
3830	ENSG00000169241.18	SLC50A1	0.39	2.52E-03	1.31E-02
3831	ENSG00000243970.3	PPIEL	-0.58	2.52E-03	1.31E-02
3832	ENSG00000108679.12	LGALS3BP	0.37	2.52E-03	1.31E-02
3833	ENSG00000136754.17	ABI1	-0.37	2.52E-03	1.31E-02
3834	ENSG00000255277.3	ABCC6P2	1.16	2.53E-03	1.31E-02
3835	ENSG00000148187.17	MRRF	0.33	2.53E-03	1.31E-02
3836	ENSG00000136630.12	HLX	-0.72	2.53E-03	1.31E-02
3837	ENSG00000169594.13	BNC1	-0.56	2.54E-03	1.31E-02
3838	ENSG00000091039.16	OSBPL8	-0.28	2.54E-03	1.31E-02
3839	ENSG00000163754.17	GYG1	-0.37	2.55E-03	1.32E-02
3840	ENSG00000247595.4	SPTY2D1OS	1.15	2.55E-03	1.32E-02
3841	ENSG00000116096.5	SPR	0.46	2.56E-03	1.32E-02
3842	ENSG00000244187.7	TMEM141	0.44	2.56E-03	1.32E-02
3843	ENSG00000161654.9	LSM12	-0.32	2.56E-03	1.32E-02
3844	ENSG00000110719.9	TCIRG1	-0.55	2.56E-03	1.32E-02
3845	ENSG00000183255.11	PTTG1IP	-0.36	2.56E-03	1.32E-02
3846	ENSG00000161981.10	SNRNP25	0.39	2.57E-03	1.32E-02
3847	ENSG00000151067.21	CACNA1C	-0.47	2.57E-03	1.33E-02

3848	ENSG00000113522.13	RAD50	-0.31	2.57E-03	1.33E-02
3849	ENSG00000120868.13	APAF1	-0.48	2.57E-03	1.33E-02
3850	ENSG00000111424.10	VDR	-0.62	2.57E-03	1.33E-02
3851	ENSG00000156711.16	MAPK13	-0.81	2.57E-03	1.33E-02
3852	ENSG00000279595.1	AC126335.2	2.07	2.58E-03	1.33E-02
3853	ENSG00000185567.6	AHNAK2	-0.49	2.58E-03	1.33E-02
3854	ENSG00000113269.13	RNF130	-0.34	2.58E-03	1.33E-02
3855	ENSG00000225614.2	ZNF469	-0.58	2.59E-03	1.33E-02
3856	ENSG00000172171.10	TEFM	0.42	2.59E-03	1.33E-02
3857	ENSG00000115825.9	PRKD3	0.32	2.59E-03	1.33E-02
3858	ENSG00000186162.10	CIDECP	0.45	2.60E-03	1.34E-02
3859	ENSG00000176244.6	ACBD7	1.43	2.61E-03	1.34E-02
3860	ENSG00000237153.3	AL162725.2	2.36	2.61E-03	1.34E-02
3861	ENSG00000237491.8	AL669831.5	0.57	2.61E-03	1.34E-02
3862	ENSG00000177984.6	LCN15	1.48	2.62E-03	1.35E-02
3863	ENSG00000149557.13	FEZ1	-0.46	2.62E-03	1.35E-02
3864	ENSG00000171291.8	ZNF439	-1.06	2.62E-03	1.35E-02
3865	ENSG00000077549.17	CAPZB	-0.29	2.63E-03	1.35E-02
3866	ENSG00000120925.15	RNF170	0.33	2.63E-03	1.35E-02
3867	ENSG00000172728.15	FUT10	-0.49	2.63E-03	1.35E-02
3868	ENSG00000164919.10	COX6C	0.31	2.64E-03	1.35E-02
3869	ENSG00000090054.14	SPTLC1	-0.30	2.64E-03	1.35E-02
3870	ENSG00000151929.9	BAG3	-0.35	2.64E-03	1.35E-02
3871	ENSG00000008283.15	CYB561	-0.49	2.64E-03	1.35E-02
3872	ENSG00000137168.7	PPIL1	0.36	2.65E-03	1.36E-02
3873	ENSG00000048052.21	HDAC9	-0.44	2.65E-03	1.36E-02
3874	ENSG00000038002.8	AGA	-0.36	2.66E-03	1.36E-02
3875	ENSG00000157600.11	TMEM164	0.28	2.66E-03	1.36E-02
3876	ENSG00000053501.12	USE1	0.50	2.66E-03	1.36E-02
3877	ENSG00000100983.10	GSS	0.34	2.66E-03	1.36E-02
3878	ENSG00000142507.9	PSMB6	0.30	2.67E-03	1.37E-02
3879	ENSG00000131788.15	PIAS3	-0.32	2.67E-03	1.37E-02
3880	ENSG00000152763.16	WDR78	-0.79	2.67E-03	1.37E-02
3881	ENSG00000119328.11	FAM206A	0.39	2.67E-03	1.37E-02
3882	ENSG00000115159.15	GPD2	0.34	2.67E-03	1.37E-02
3883	ENSG00000119318.12	RAD23B	-0.30	2.67E-03	1.37E-02
3884	ENSG00000091592.15	NLRP1	-0.40	2.67E-03	1.37E-02
3885	ENSG00000272701.3	MESTIT1	1.89	2.69E-03	1.37E-02
3886	ENSG00000164715.5	LMTK2	0.37	2.69E-03	1.38E-02
3887	ENSG00000113810.15	SMC4	-0.40	2.69E-03	1.38E-02
3888	ENSG00000080608.9	PUM3	0.36	2.70E-03	1.38E-02
3889	ENSG00000278932.3	CR381653.1	-0.77	2.70E-03	1.38E-02
3890	ENSG00000170633.16	RNF34	-0.46	2.71E-03	1.38E-02
3891	ENSG00000175414.6	ARL10	0.36	2.71E-03	1.38E-02
3892	ENSG00000112210.11	RAB23	-0.30	2.71E-03	1.38E-02
3893	ENSG00000272086.1	AC025181.2	0.85	2.72E-03	1.38E-02
3894	ENSG00000179820.15	MYADM	-0.47	2.72E-03	1.39E-02
3895	ENSG00000137161.16	CNPY3	-0.50	2.74E-03	1.39E-02
3896	ENSG00000127241.16	MASP1	-0.55	2.74E-03	1.40E-02
3897	ENSG00000172244.8	C5orf34	-0.94	2.75E-03	1.40E-02

3898	ENSG00000136826.14	KLF4	-0.56	2.75E-03	1.40E-02
3899	ENSG00000163364.9	LINC01116	-0.85	2.76E-03	1.40E-02
3900	ENSG00000171533.11	MAP6	-0.47	2.76E-03	1.41E-02
3901	ENSG00000006016.10	CRLF1	-0.85	2.77E-03	1.41E-02
3902	ENSG00000168653.10	NDUFS5	0.29	2.79E-03	1.42E-02
3903	ENSG00000185883.11	ATP6V0C	0.33	2.79E-03	1.42E-02
3904	ENSG00000169288.17	MRPL1	0.41	2.80E-03	1.42E-02
3905	ENSG00000137285.9	TUBB2B	0.73	2.80E-03	1.42E-02
3906	ENSG00000119979.17	FAM45A	-0.34	2.80E-03	1.42E-02
3907	ENSG00000226025.9	LGALS17A	3.20	2.81E-03	1.43E-02
3908	ENSG00000188215.9	DCUN1D3	0.40	2.82E-03	1.43E-02
3909	ENSG00000254226.5	LINC01933	1.67	2.83E-03	1.44E-02
3910	ENSG00000130921.7	C12orf65	-0.43	2.83E-03	1.44E-02
3911	ENSG00000176148.15	TCP11L1	-0.51	2.84E-03	1.44E-02
3912	ENSG00000225663.7	MCRIP1	0.49	2.84E-03	1.44E-02
3913	ENSG00000237649.7	KIFC1	-2.22	2.85E-03	1.45E-02
3914	ENSG00000174125.7	TLR1	-0.89	2.87E-03	1.46E-02
3915	ENSG00000269242.1	AC010422.3	-2.45	2.87E-03	1.46E-02
3916	ENSG00000162267.12	ITIH3	0.96	2.88E-03	1.46E-02
3917	ENSG00000127084.18	FGD3	-1.21	2.88E-03	1.46E-02
3918	ENSG00000107331.16	ABCA2	-0.41	2.89E-03	1.47E-02
3919	ENSG00000198899.2	MT-ATP6	0.27	2.90E-03	1.47E-02
3920	ENSG00000008735.13	MAPK8IP2	-0.53	2.91E-03	1.47E-02
3921	ENSG00000149591.16	TAGLN	-0.39	2.91E-03	1.47E-02
3922	ENSG00000186812.12	ZNF397	-0.36	2.92E-03	1.48E-02
3923	ENSG00000238181.2	AHCYP2	2.24	2.92E-03	1.48E-02
3924	ENSG00000175564.12	UCP3	-1.34	2.93E-03	1.48E-02
3925	ENSG00000070404.9	FSTL3	-0.53	2.94E-03	1.49E-02
3926	ENSG00000154556.18	SORBS2	-0.54	2.95E-03	1.49E-02
3927	ENSG00000151014.5	NOCT	0.67	2.95E-03	1.49E-02
3928	ENSG00000084092.6	NOA1	0.35	2.95E-03	1.49E-02
3929	ENSG00000124380.10	SNRNP27	0.33	2.96E-03	1.49E-02
3930	ENSG00000121579.12	NAA50	0.33	2.96E-03	1.50E-02
3931	ENSG00000106258.14	CYP3A5	-0.86	2.97E-03	1.50E-02
3932	ENSG00000169903.6	TM4SF4	1.34	2.97E-03	1.50E-02
3933	ENSG00000173141.4	MRPL57	0.39	2.98E-03	1.51E-02
3934	ENSG00000145075.12	CCDC39	-0.52	2.99E-03	1.51E-02
3935	ENSG00000111911.6	HINT3	0.35	3.00E-03	1.51E-02
3936	ENSG00000148795.6	CYP17A1	3.37	3.00E-03	1.51E-02
3937	ENSG00000125753.13	VASP	-0.45	3.00E-03	1.51E-02
3938	ENSG00000197785.13	ATAD3A	0.47	3.00E-03	1.51E-02
3939	ENSG00000115307.16	AUP1	0.28	3.01E-03	1.52E-02
3940	ENSG00000154096.13	THY1	-0.46	3.01E-03	1.52E-02
3941	ENSG00000163492.15	CCDC141	1.20	3.02E-03	1.52E-02
3942	ENSG00000272040.1	AC010245.2	1.19	3.02E-03	1.52E-02
3943	ENSG00000012963.14	UBR7	-0.34	3.03E-03	1.53E-02
3944	ENSG00000171928.13	TVP23B	0.35	3.03E-03	1.53E-02
3945	ENSG00000256073.3	URB1-AS1	0.75	3.04E-03	1.53E-02
3946	ENSG00000155511.17	GRIA1	-0.88	3.05E-03	1.54E-02
3947	ENSG00000128829.11	EIF2AK4	-0.29	3.06E-03	1.54E-02

3948	ENSG00000126953.6	TIMM8A	0.53	3.06E-03	1.54E-02
3949	ENSG00000042317.16	SPATA7	0.53	3.07E-03	1.54E-02
3950	ENSG00000125735.10	TNFSF14	-1.78	3.07E-03	1.54E-02
3951	ENSG00000012817.15	KDM5D	-0.31	3.08E-03	1.55E-02
3952	ENSG00000153214.10	TMEM87B	-0.39	3.08E-03	1.55E-02
3953	ENSG00000224370.1	AC093392.2	1.66	3.09E-03	1.55E-02
3954	ENSG00000233521.5	LINC01638	1.36	3.09E-03	1.55E-02
3955	ENSG00000011638.10	TMEM159	-0.50	3.09E-03	1.55E-02
3956	ENSG00000114126.17	TFDP2	-0.33	3.10E-03	1.56E-02
3957	ENSG00000087111.20	PIGS	0.28	3.10E-03	1.56E-02
3958	ENSG00000128596.16	CCDC136	-0.64	3.10E-03	1.56E-02
3959	ENSG00000016864.18	GLT8D1	-0.38	3.11E-03	1.56E-02
3960	ENSG00000162695.11	SLC30A7	-0.38	3.11E-03	1.56E-02
3961	ENSG00000228474.5	OST4	0.27	3.12E-03	1.56E-02
3962	ENSG00000134504.13	KCTD1	-0.53	3.12E-03	1.56E-02
3963	ENSG00000158813.17	EDA	1.00	3.12E-03	1.56E-02
3964	ENSG00000204386.10	NEU1	0.37	3.12E-03	1.56E-02
3965	ENSG00000189171.14	S100A13	0.30	3.13E-03	1.56E-02
3966	ENSG00000104067.16	TJP1	-0.29	3.13E-03	1.56E-02
3967	ENSG00000115091.11	ACTR3	-0.33	3.13E-03	1.56E-02
3968	ENSG00000117228.9	GBP1	-0.38	3.13E-03	1.56E-02
3969	ENSG00000164850.14	GPER1	0.50	3.13E-03	1.57E-02
3970	ENSG00000128040.10	SPINK2	-2.74	3.16E-03	1.58E-02
3971	ENSG00000198792.12	TMEM184B	0.37	3.17E-03	1.58E-02
3972	ENSG00000113407.13	TARS	0.31	3.17E-03	1.58E-02
3973	ENSG00000142694.6	EVA1B	-0.86	3.19E-03	1.59E-02
3974	ENSG00000094841.13	UPRT	0.42	3.19E-03	1.59E-02
3975	ENSG00000174233.11	ADCY6	-0.33	3.19E-03	1.59E-02
3976	ENSG00000120910.14	PPP3CC	0.38	3.20E-03	1.60E-02
3977	ENSG00000161594.6	KLHL10	0.81	3.21E-03	1.60E-02
3978	ENSG00000268220.1	AC008040.5	-0.92	3.21E-03	1.60E-02
3979	ENSG00000072163.19	LIMS2	-0.58	3.21E-03	1.60E-02
3980	ENSG00000162944.10	RFTN2	-0.43	3.22E-03	1.60E-02
3981	ENSG00000164104.11	HMGB2	-0.47	3.23E-03	1.61E-02
3982	ENSG00000065882.15	TBC1D1	0.37	3.23E-03	1.61E-02
3983	ENSG00000150457.8	LATS2	-0.30	3.23E-03	1.61E-02
3984	ENSG00000233622.3	CYP2T1P	1.01	3.24E-03	1.61E-02
3985	ENSG00000284691.1	AC073111.5	0.51	3.24E-03	1.61E-02
3986	ENSG00000137807.15	KIF23	-0.97	3.24E-03	1.61E-02
3987	ENSG00000050405.13	LIMA1	0.29	3.26E-03	1.62E-02
3988	ENSG00000188002.10	AC026412.1	0.47	3.27E-03	1.63E-02
3989	ENSG00000115415.18	STAT1	-0.31	3.27E-03	1.63E-02
3990	ENSG00000175063.16	UBE2C	-2.77	3.28E-03	1.63E-02
3991	ENSG00000252690.3	AC105339.2	0.69	3.29E-03	1.63E-02
3992	ENSG00000117643.14	MAN1C1	-0.61	3.29E-03	1.63E-02
3993	ENSG00000077380.15	DYNC1I2	-0.30	3.29E-03	1.64E-02
3994	ENSG00000165782.10	PIP4P1	0.34	3.29E-03	1.64E-02
3995	ENSG00000188338.14	SLC38A3	1.09	3.29E-03	1.64E-02
3996	ENSG00000164120.13	HPGD	1.33	3.29E-03	1.64E-02
3997	ENSG00000133131.14	MORC4	0.34	3.30E-03	1.64E-02

3998	ENSG00000077097.15	TOP2B	-0.32	3.30E-03	1.64E-02
3999	ENSG00000124635.8	HIST1H2BJ	0.67	3.31E-03	1.64E-02
4000	ENSG00000166046.10	TCP11L2	-0.54	3.32E-03	1.65E-02
4001	ENSG00000102547.18	CAB39L	-0.49	3.32E-03	1.65E-02
4002	ENSG00000168497.4	CAVIN2	-0.44	3.33E-03	1.65E-02
4003	ENSG00000181924.6	COA4	0.36	3.33E-03	1.65E-02
4004	ENSG00000112651.11	MRPL2	0.39	3.34E-03	1.66E-02
4005	ENSG00000280798.1	LINC00294	-0.58	3.34E-03	1.66E-02
4006	ENSG00000172890.12	NADSYN1	-0.36	3.36E-03	1.66E-02
4007	ENSG00000013588.8	GPRC5A	-0.89	3.36E-03	1.67E-02
4008	ENSG00000083290.19	ULK2	-0.34	3.37E-03	1.67E-02
4009	ENSG00000167081.16	PBX3	-0.57	3.37E-03	1.67E-02
4010	ENSG00000278709.1	NKILA	0.88	3.39E-03	1.68E-02
4011	ENSG00000141448.8	GATA6	-0.62	3.39E-03	1.68E-02
4012	ENSG00000203485.12	INF2	0.42	3.40E-03	1.68E-02
4013	ENSG00000212747.4	RTL8B	-0.39	3.40E-03	1.68E-02
4014	ENSG00000105329.9	TGFB1	-0.52	3.40E-03	1.68E-02
4015	ENSG00000160299.16	PCNT	-0.34	3.41E-03	1.68E-02
4016	ENSG00000172985.10	SH3RF3	-0.79	3.41E-03	1.68E-02
4017	ENSG00000138835.22	RGS3	0.46	3.42E-03	1.69E-02
4018	ENSG00000110090.12	CPT1A	-0.67	3.43E-03	1.69E-02
4019	ENSG00000162688.16	AGL	-0.28	3.43E-03	1.69E-02
4020	ENSG00000166130.14	IKBIP	-0.55	3.43E-03	1.69E-02
4021	ENSG00000110628.14	SLC22A18	-0.78	3.44E-03	1.70E-02
4022	ENSG00000245694.9	CRNDE	-0.49	3.44E-03	1.70E-02
4023	ENSG00000172795.15	DCP2	0.34	3.45E-03	1.70E-02
4024	ENSG00000156273.15	BACH1	-0.47	3.45E-03	1.70E-02
4025	ENSG00000132646.10	PCNA	-0.48	3.46E-03	1.71E-02
4026	ENSG00000111801.15	BTN3A3	0.33	3.47E-03	1.71E-02
4027	ENSG00000002834.17	LASP1	-0.29	3.47E-03	1.71E-02
4028	ENSG00000235760.4	HCG2040054	3.74	3.47E-03	1.71E-02
4029	ENSG00000173338.12	KCNK7	1.45	3.48E-03	1.71E-02
4030	ENSG00000155085.15	AK9	0.53	3.49E-03	1.72E-02
4031	ENSG00000165140.10	FBP1	3.26	3.49E-03	1.72E-02
4032	ENSG00000153029.14	MR1	-0.33	3.50E-03	1.72E-02
4033	ENSG00000196083.9	IL1RAP	-0.45	3.50E-03	1.72E-02
4034	ENSG00000085185.15	BCORL1	-0.45	3.50E-03	1.72E-02
4035	ENSG00000107779.13	BMPR1A	-0.30	3.52E-03	1.73E-02
4036	ENSG00000164933.11	SLC25A32	0.35	3.52E-03	1.73E-02
4037	ENSG00000078808.16	SDF4	-0.37	3.54E-03	1.74E-02
4038	ENSG00000168778.11	TCTN2	-0.65	3.56E-03	1.75E-02
4039	ENSG00000229400.1	AL596330.1	3.31	3.57E-03	1.75E-02
4040	ENSG00000146828.17	SLC12A9	-0.56	3.57E-03	1.76E-02
4041	ENSG00000177822.7	AC098864.1	0.57	3.58E-03	1.76E-02
4042	ENSG00000182117.5	NOP10	0.34	3.58E-03	1.76E-02
4043	ENSG00000173320.11	STOX2	-0.80	3.58E-03	1.76E-02
4044	ENSG00000100221.10	JOSD1	-0.40	3.60E-03	1.76E-02
4045	ENSG00000106479.10	ZNF862	-0.44	3.60E-03	1.76E-02
4046	ENSG00000277782.1	AC068870.2	1.04	3.60E-03	1.77E-02
4047	ENSG00000137962.12	ARHGAP29	0.40	3.61E-03	1.77E-02

4048	ENSG00000213398.7	LCAT	-0.56	3.61E-03	1.77E-02
4049	ENSG00000186635.14	ARAP1	0.32	3.62E-03	1.77E-02
4050	ENSG00000112305.14	SMAP1	0.30	3.62E-03	1.78E-02
4051	ENSG00000122359.17	ANXA11	0.33	3.63E-03	1.78E-02
4052	ENSG00000140688.16	C16orf58	0.32	3.63E-03	1.78E-02
4053	ENSG00000116157.5	GPX7	0.38	3.64E-03	1.78E-02
4054	ENSG00000133107.14	TRPC4	-0.69	3.64E-03	1.78E-02
4055	ENSG00000263126.1	AC040162.3	-1.86	3.64E-03	1.78E-02
4056	ENSG00000163235.15	TGFA	1.80	3.65E-03	1.79E-02
4057	ENSG00000166482.11	MFAP4	-0.34	3.67E-03	1.80E-02
4058	ENSG00000253369.1	AC131902.1	2.92	3.68E-03	1.80E-02
4059	ENSG00000235162.8	C12orf75	-0.48	3.68E-03	1.80E-02
4060	ENSG00000145284.11	SCD5	-0.58	3.69E-03	1.80E-02
4061	ENSG00000197535.14	MYO5A	-0.32	3.70E-03	1.81E-02
4062	ENSG00000106415.12	GLCCI1	-0.40	3.70E-03	1.81E-02
4063	ENSG00000167642.12	SPINT2	-1.17	3.71E-03	1.81E-02
4064	ENSG00000137337.14	MDC1	-0.41	3.71E-03	1.81E-02
4065	ENSG00000142634.12	EFHD2	0.49	3.72E-03	1.82E-02
4066	ENSG00000100033.16	PRODH	-1.35	3.72E-03	1.82E-02
4067	ENSG00000100577.18	GSTZ1	0.40	3.72E-03	1.82E-02
4068	ENSG00000177692.11	DNAJC28	0.64	3.73E-03	1.82E-02
4069	ENSG00000156467.9	UQCRB	0.32	3.73E-03	1.82E-02
4070	ENSG00000005339.14	CREBBP	-0.30	3.75E-03	1.83E-02
4071	ENSG00000170165.5	CR848007.1	-2.84	3.76E-03	1.83E-02
4072	ENSG00000081177.18	EXD2	0.31	3.76E-03	1.83E-02
4073	ENSG00000276289.4	KCNE1B	-1.44	3.76E-03	1.83E-02
4074	ENSG00000128849.10	CGNL1	1.16	3.76E-03	1.83E-02
4075	ENSG00000263001.5	GTF2I	-0.24	3.76E-03	1.83E-02
4076	ENSG00000241553.12	ARPC4	-0.26	3.77E-03	1.84E-02
4077	ENSG00000101193.7	GID8	-0.32	3.79E-03	1.84E-02
4078	ENSG00000173020.10	GRK2	0.36	3.79E-03	1.85E-02
4079	ENSG00000116525.13	TRIM62	-0.78	3.79E-03	1.85E-02
4080	ENSG00000163517.14	HDAC11	-0.37	3.80E-03	1.85E-02
4081	ENSG00000071575.11	TRIB2	-0.55	3.81E-03	1.85E-02
4082	ENSG00000117616.17	RSRP1	-0.40	3.82E-03	1.86E-02
4083	ENSG00000214941.7	ZSWIM7	0.47	3.82E-03	1.86E-02
4084	ENSG00000179403.11	VWA1	-1.01	3.83E-03	1.86E-02
4085	ENSG00000155760.2	FZD7	-0.35	3.83E-03	1.86E-02
4086	ENSG00000164031.16	DNAJB14	-0.31	3.84E-03	1.87E-02
4087	ENSG00000196155.12	PLEKHG4	-0.51	3.85E-03	1.87E-02
4088	ENSG00000167004.12	PDIA3	-0.36	3.85E-03	1.87E-02
4089	ENSG00000149380.11	P4HA3	-0.59	3.87E-03	1.88E-02
4090	ENSG00000101343.14	CRNKL1	-0.37	3.87E-03	1.88E-02
4091	ENSG00000213742.6	ZNF337-AS1	0.66	3.88E-03	1.88E-02
4092	ENSG00000013364.18	MVP	-0.44	3.88E-03	1.88E-02
4093	ENSG00000164081.12	TEX264	0.33	3.88E-03	1.88E-02
4094	ENSG00000104427.11	ZC2HC1A	-0.45	3.89E-03	1.89E-02
4095	ENSG00000136160.15	EDNRB	-0.59	3.89E-03	1.89E-02
4096	ENSG00000167232.13	ZNF91	0.34	3.91E-03	1.90E-02
4097	ENSG00000272601.1	AC010655.3	0.74	3.92E-03	1.90E-02

4098	ENSG00000183765.21	CHEK2	-0.60	3.93E-03	1.90E-02
4099	ENSG00000040731.10	CDH10	-1.10	3.93E-03	1.90E-02
4100	ENSG00000260630.6	SNAI3-AS1	0.47	3.93E-03	1.90E-02
4101	ENSG00000091128.12	LAMB4	1.95	3.94E-03	1.91E-02
4102	ENSG00000115963.13	RND3	-0.37	3.94E-03	1.91E-02
4103	ENSG00000148908.14	RGS10	-0.59	3.94E-03	1.91E-02
4104	ENSG00000116350.16	SRSF4	-0.27	3.95E-03	1.91E-02
4105	ENSG00000171681.12	ATF7IP	-0.27	3.96E-03	1.91E-02
4106	ENSG00000244560.7	AC004890.2	-0.56	3.96E-03	1.91E-02
4107	ENSG00000137880.5	GCHFR	0.85	3.96E-03	1.91E-02
4108	ENSG00000100596.6	SPTLC2	-0.40	3.96E-03	1.91E-02
4109	ENSG00000032389.12	EIPR1	0.39	3.96E-03	1.91E-02
4110	ENSG00000128872.9	TMOD2	-0.43	3.96E-03	1.91E-02
4111	ENSG00000111897.6	SERINC1	-0.25	3.97E-03	1.92E-02
4112	ENSG00000102172.15	SMS	-0.39	3.97E-03	1.92E-02
4113	ENSG00000143420.18	ENSA	0.27	3.97E-03	1.92E-02
4114	ENSG00000140876.11	NUDT7	0.49	3.98E-03	1.92E-02
4115	ENSG00000083720.12	OXCT1	-0.31	4.00E-03	1.93E-02
4116	ENSG00000231721.7	LINC-PINT	0.63	4.00E-03	1.93E-02
4117	ENSG00000104969.9	SGTA	0.39	4.00E-03	1.93E-02
4118	ENSG00000104679.10	R3HCC1	0.39	4.01E-03	1.93E-02
4119	ENSG00000165182.11	CXorf58	1.50	4.01E-03	1.93E-02
4120	ENSG00000089041.16	P2RX7	1.29	4.01E-03	1.93E-02
4121	ENSG00000099937.10	SERPIND1	1.75	4.02E-03	1.94E-02
4122	ENSG00000147162.13	OGT	-0.24	4.02E-03	1.94E-02
4123	ENSG00000135749.18	PCNX2	-0.47	4.03E-03	1.94E-02
4124	ENSG00000153046.17	CDYL	-0.32	4.03E-03	1.94E-02
4125	ENSG00000259939.1	AC022167.1	-1.89	4.03E-03	1.94E-02
4126	ENSG00000100336.17	APOL4	0.96	4.03E-03	1.94E-02
4127	ENSG00000248144.5	ADH1C	1.61	4.04E-03	1.94E-02
4128	ENSG00000133794.17	ARNTL	-0.53	4.04E-03	1.94E-02
4129	ENSG00000221978.11	CCNL2	-0.32	4.05E-03	1.95E-02
4130	ENSG00000111716.12	LDHB	-0.27	4.05E-03	1.95E-02
4131	ENSG00000275512.1	AC007998.4	0.71	4.08E-03	1.96E-02
4132	ENSG00000197020.10	ZNF100	0.47	4.09E-03	1.97E-02
4133	ENSG00000054965.10	FAM168A	-0.33	4.10E-03	1.97E-02
4134	ENSG00000107021.15	TBC1D13	0.31	4.10E-03	1.97E-02
4135	ENSG00000179241.12	LDLRAD3	0.34	4.12E-03	1.98E-02
4136	ENSG00000142686.7	C1orf216	-0.31	4.13E-03	1.98E-02
4137	ENSG00000133433.10	GSTT2B	-0.54	4.14E-03	1.98E-02
4138	ENSG00000254319.5	AC246817.2	1.38	4.15E-03	1.99E-02
4139	ENSG00000157833.12	GAREM2	0.54	4.15E-03	1.99E-02
4140	ENSG00000231770.5	TMEM44-AS1	0.76	4.15E-03	1.99E-02
4141	ENSG00000104894.11	CD37	-1.08	4.15E-03	1.99E-02
4142	ENSG00000170469.10	SPATA24	1.06	4.16E-03	1.99E-02
4143	ENSG00000214022.11	REPIN1	0.29	4.16E-03	1.99E-02
4144	ENSG00000173285.4	OR10K1	1.24	4.18E-03	2.00E-02
4145	ENSG00000176826.15	FKBP9P1	-0.66	4.19E-03	2.01E-02
4146	ENSG00000183172.8	SMDT1	0.33	4.20E-03	2.01E-02
4147	ENSG00000162086.14	ZNF75A	-0.38	4.21E-03	2.02E-02

4148	ENSG00000196741.5	LINC01560	-0.89	4.21E-03	2.02E-02
4149	ENSG00000183718.5	TRIM52	0.36	4.22E-03	2.02E-02
4150	ENSG00000183578.7	TNFAIP8L3	0.59	4.24E-03	2.03E-02
4151	ENSG00000116329.10	OPRD1	0.86	4.25E-03	2.03E-02
4152	ENSG00000103494.13	RPGRIP1L	-0.45	4.26E-03	2.04E-02
4153	ENSG00000123600.19	METTL8	-0.35	4.28E-03	2.05E-02
4154	ENSG00000115935.17	WIPF1	-0.38	4.28E-03	2.05E-02
4155	ENSG00000168672.3	FAM84B	-0.65	4.28E-03	2.05E-02
4156	ENSG00000009954.10	BAZ1B	-0.28	4.29E-03	2.05E-02
4157	ENSG00000183010.16	PYCR1	-0.53	4.29E-03	2.05E-02
4158	ENSG00000155229.20	MMS19	-0.31	4.30E-03	2.05E-02
4159	ENSG00000128536.15	CDHR3	-0.49	4.30E-03	2.05E-02
4160	ENSG00000167193.7	CRK	0.25	4.30E-03	2.05E-02
4161	ENSG00000108256.8	NUFIP2	-0.28	4.30E-03	2.05E-02
4162	ENSG00000213903.8	LTB4R	-0.81	4.31E-03	2.06E-02
4163	ENSG00000205885.7	C1RL-AS1	-0.88	4.32E-03	2.06E-02
4164	ENSG00000102710.19	SUPT20H	-0.33	4.33E-03	2.07E-02
4165	ENSG00000168883.19	USP39	-0.32	4.34E-03	2.07E-02
4166	ENSG00000125970.11	RALY	-0.30	4.34E-03	2.07E-02
4167	ENSG00000036549.12	AC118549.1	-0.36	4.34E-03	2.07E-02
4168	ENSG00000115129.13	TP53I3	0.36	4.35E-03	2.07E-02
4169	ENSG00000174010.9	KLHL15	0.37	4.37E-03	2.08E-02
4170	ENSG00000197713.14	RPE	0.31	4.37E-03	2.08E-02
4171	ENSG00000070081.16	NUCB2	-0.36	4.37E-03	2.08E-02
4172	ENSG00000118785.13	SPP1	-0.97	4.37E-03	2.08E-02
4173	ENSG00000136114.16	THSD1	-0.80	4.37E-03	2.08E-02
4174	ENSG00000124194.16	GDAP1L1	2.17	4.38E-03	2.08E-02
4175	ENSG00000215695.1	RSC1A1	0.64	4.38E-03	2.08E-02
4176	ENSG00000102781.13	KATNAL1	-0.31	4.38E-03	2.08E-02
4177	ENSG00000166851.14	PLK1	-1.06	4.38E-03	2.08E-02
4178	ENSG00000143442.21	POGZ	-0.30	4.39E-03	2.09E-02
4179	ENSG00000119950.20	MXI1	-0.40	4.39E-03	2.09E-02
4180	ENSG00000253368.3	TRNP1	-0.50	4.40E-03	2.09E-02
4181	ENSG00000118680.13	MYL12B	-0.33	4.41E-03	2.10E-02
4182	ENSG00000080345.17	RIF1	-0.29	4.42E-03	2.10E-02
4183	ENSG00000152242.10	C18orf25	0.38	4.44E-03	2.11E-02
4184	ENSG00000277258.4	PCGF2	-0.48	4.45E-03	2.11E-02
4185	ENSG00000166928.10	MS4A14	-2.20	4.45E-03	2.11E-02
4186	ENSG00000164038.14	SLC9B2	-0.53	4.46E-03	2.11E-02
4187	ENSG00000166260.11	COX11	0.32	4.46E-03	2.11E-02
4188	ENSG00000261172.1	AC133919.2	0.94	4.46E-03	2.11E-02
4189	ENSG00000081692.12	JMJD4	0.44	4.48E-03	2.12E-02
4190	ENSG00000146648.17	EGFR	0.32	4.48E-03	2.12E-02
4191	ENSG00000239887.4	C1orf226	2.02	4.48E-03	2.12E-02
4192	ENSG00000133316.15	WDR74	0.41	4.49E-03	2.12E-02
4193	ENSG00000100321.14	SYNGR1	0.60	4.49E-03	2.13E-02
4194	ENSG00000112851.14	ERBIN	-0.28	4.50E-03	2.13E-02
4195	ENSG00000106443.16	PHF14	-0.43	4.51E-03	2.13E-02
4196	ENSG00000276805.2	AL133216.2	0.63	4.52E-03	2.14E-02
4197	ENSG00000118292.8	C1orf54	-0.74	4.52E-03	2.14E-02

4198	ENSG00000106688.11	SLC1A1	-0.51	4.53E-03	2.14E-02
4199	ENSG00000165795.23	NDRG2	-0.53	4.53E-03	2.14E-02
4200	ENSG00000183569.17	SERHL2	-0.79	4.53E-03	2.14E-02
4201	ENSG00000164741.14	DLC1	0.32	4.54E-03	2.14E-02
4202	ENSG00000110700.6	RPS13	0.33	4.57E-03	2.16E-02
4203	ENSG00000102098.17	SCML2	0.76	4.58E-03	2.16E-02
4204	ENSG00000161638.10	ITGA5	-0.42	4.59E-03	2.17E-02
4205	ENSG00000139329.4	LUM	-0.35	4.59E-03	2.17E-02
4206	ENSG00000250644.3	AC068580.4	0.64	4.59E-03	2.17E-02
4207	ENSG00000160766.14	GBAP1	-0.60	4.60E-03	2.17E-02
4208	ENSG00000143862.7	ARL8A	0.33	4.61E-03	2.17E-02
4209	ENSG00000074696.12	HACD3	0.28	4.61E-03	2.17E-02
4210	ENSG00000182013.17	PNMA8A	-0.71	4.62E-03	2.18E-02
4211	ENSG00000180596.7	HIST1H2BC	0.48	4.63E-03	2.18E-02
4212	ENSG00000121774.17	KHDRBS1	-0.32	4.63E-03	2.18E-02
4213	ENSG00000146143.17	PRIM2	-0.45	4.63E-03	2.18E-02
4214	ENSG00000182632.15	CCNYL2	0.99	4.65E-03	2.19E-02
4215	ENSG00000134363.11	FST	-0.94	4.66E-03	2.19E-02
4216	ENSG00000186566.12	GPATCH8	-0.30	4.66E-03	2.19E-02
4217	ENSG00000070770.8	CSNK2A2	0.29	4.66E-03	2.19E-02
4218	ENSG00000137343.17	ATAT1	-0.43	4.67E-03	2.20E-02
4219	ENSG00000074416.14	MGLL	0.38	4.67E-03	2.20E-02
4220	ENSG00000183520.11	UTP11	0.33	4.67E-03	2.20E-02
4221	ENSG00000144476.5	ACKR3	0.28	4.67E-03	2.20E-02
4222	ENSG00000111639.7	MRPL51	0.27	4.70E-03	2.21E-02
4223	ENSG00000203705.10	TATDN3	0.39	4.70E-03	2.21E-02
4224	ENSG00000128578.9	STRIP2	0.59	4.70E-03	2.21E-02
4225	ENSG00000144741.17	SLC25A26	0.38	4.70E-03	2.21E-02
4226	ENSG00000112419.14	PHACTR2	0.31	4.70E-03	2.21E-02
4227	ENSG00000139793.18	MBNL2	0.36	4.72E-03	2.21E-02
4228	ENSG00000110171.19	TRIM3	-0.47	4.72E-03	2.21E-02
4229	ENSG00000156970.12	BUB1B	-2.25	4.72E-03	2.21E-02
4230	ENSG00000196544.7	BORCS6	0.58	4.72E-03	2.21E-02
4231	ENSG00000134146.11	DPH6	0.60	4.73E-03	2.22E-02
4232	ENSG00000081154.11	PCNP	-0.35	4.73E-03	2.22E-02
4233	ENSG00000118503.14	TNFAIP3	0.46	4.73E-03	2.22E-02
4234	ENSG00000138623.10	SEMA7A	-0.57	4.74E-03	2.22E-02
4235	ENSG00000197291.8	RAMP2-AS1	0.79	4.76E-03	2.23E-02
4236	ENSG00000059377.16	TBXAS1	1.09	4.77E-03	2.24E-02
4237	ENSG00000100968.13	NFATC4	-0.40	4.78E-03	2.24E-02
4238	ENSG00000113971.19	NPHP3	-0.31	4.79E-03	2.24E-02
4239	ENSG00000154263.17	ABCA10	-0.59	4.79E-03	2.24E-02
4240	ENSG00000072195.14	SPEG	-0.47	4.79E-03	2.24E-02
4241	ENSG00000205632.3	LINC01310	1.79	4.79E-03	2.24E-02
4242	ENSG00000260342.2	AC138811.2	0.49	4.80E-03	2.25E-02
4243	ENSG00000166823.5	MESP1	0.57	4.83E-03	2.26E-02
4244	ENSG00000108465.14	CDK5RAP3	-0.31	4.87E-03	2.28E-02
4245	ENSG00000215218.3	UBE2QL1	2.07	4.87E-03	2.28E-02
4246	ENSG00000185787.14	MORF4L1	-0.25	4.88E-03	2.28E-02
4247	ENSG00000144224.16	UBXN4	-0.27	4.89E-03	2.29E-02

4248	ENSG00000172469.15	MANEA	-0.36	4.90E-03	2.29E-02
4249	ENSG00000102178.12	UBL4A	-0.39	4.90E-03	2.29E-02
4250	ENSG00000198551.9	ZNF627	-0.42	4.90E-03	2.29E-02
4251	ENSG00000269604.1	AC005523.2	0.32	4.91E-03	2.29E-02
4252	ENSG00000116754.13	SRSF11	-0.27	4.91E-03	2.29E-02
4253	ENSG00000241644.2	INMT	-0.51	4.92E-03	2.29E-02
4254	ENSG00000185722.17	ANKFY1	-0.26	4.93E-03	2.30E-02
4255	ENSG00000170017.12	ALCAM	-0.43	4.94E-03	2.30E-02
4256	ENSG00000062716.12	VMP1	-0.31	4.94E-03	2.30E-02
4257	ENSG00000141232.4	TOB1	0.34	4.95E-03	2.31E-02
4258	ENSG00000172830.12	SSH3	-0.54	4.95E-03	2.31E-02
4259	ENSG00000280120.1	AC073857.1	-0.79	4.97E-03	2.32E-02
4260	ENSG00000120332.15	TNN	1.26	4.98E-03	2.32E-02
4261	ENSG00000166532.15	RIMKLB	0.31	4.98E-03	2.32E-02
4262	ENSG00000176973.7	FAM89B	-0.43	4.99E-03	2.32E-02
4263	ENSG00000150779.11	TIMM8B	0.35	4.99E-03	2.32E-02
4264	ENSG00000276386.1	CNTNAP3P2	1.43	5.01E-03	2.33E-02
4265	ENSG00000158882.14	TOMM40L	0.33	5.01E-03	2.33E-02
4266	ENSG00000139597.17	N4BP2L1	-0.71	5.02E-03	2.34E-02
4267	ENSG00000155366.16	RHOC	-0.35	5.03E-03	2.34E-02
4268	ENSG00000233030.2	AC243772.2	-1.46	5.04E-03	2.34E-02
4269	ENSG00000180066.9	C10orf91	1.30	5.06E-03	2.35E-02
4270	ENSG00000106366.8	SERPINE1	-0.93	5.07E-03	2.36E-02
4271	ENSG00000130770.17	ATP5IF1	-0.33	5.07E-03	2.36E-02
4272	ENSG00000177640.15	CASC2	-0.86	5.08E-03	2.36E-02
4273	ENSG00000076928.17	ARHGEF1	-0.40	5.10E-03	2.37E-02
4274	ENSG00000163702.19	IL17RC	-0.48	5.11E-03	2.37E-02
4275	ENSG00000074211.13	PPP2R2C	-1.61	5.13E-03	2.38E-02
4276	ENSG00000059573.8	ALDH18A1	-0.27	5.13E-03	2.38E-02
4277	ENSG00000260077.1	AC104794.2	0.68	5.14E-03	2.39E-02
4278	ENSG00000136463.7	TACO1	0.35	5.15E-03	2.39E-02
4279	ENSG00000205133.11	TRIQK	-0.39	5.15E-03	2.39E-02
4280	ENSG00000176454.13	LPCAT4	-0.68	5.15E-03	2.39E-02
4281	ENSG00000099821.13	POLRMT	0.45	5.16E-03	2.39E-02
4282	ENSG00000228544.1	CCDC183-AS1	0.78	5.17E-03	2.40E-02
4283	ENSG00000154889.16	MPPE1	-0.35	5.18E-03	2.40E-02
4284	ENSG00000065802.11	ASB1	0.35	5.19E-03	2.41E-02
4285	ENSG00000168758.10	SEMA4C	-0.43	5.19E-03	2.41E-02
4286	ENSG00000174827.13	PDZK1	0.81	5.20E-03	2.41E-02
4287	ENSG00000140575.12	IQGAP1	-0.29	5.20E-03	2.41E-02
4288	ENSG00000249330.1	AC095060.1	2.84	5.21E-03	2.41E-02
4289	ENSG00000128524.4	ATP6V1F	0.31	5.22E-03	2.41E-02
4290	ENSG00000072506.12	HSD17B10	0.27	5.22E-03	2.41E-02
4291	ENSG00000117614.9	SYF2	-0.35	5.22E-03	2.42E-02
4292	ENSG00000245532.8	NEAT1	-0.43	5.24E-03	2.42E-02
4293	ENSG00000196405.12	EVL	0.37	5.24E-03	2.42E-02
4294	ENSG00000282508.1	LINC01002	0.51	5.24E-03	2.42E-02
4295	ENSG00000164414.17	SLC35A1	-0.38	5.25E-03	2.43E-02
4296	ENSG00000142552.7	RCN3	-0.55	5.28E-03	2.44E-02
4297	ENSG00000080823.22	MOK	0.47	5.29E-03	2.44E-02

4298	ENSG00000124813.21	RUNX2	-0.51	5.29E-03	2.44E-02
4299	ENSG00000172965.15	MIR4435-2HG	-0.58	5.32E-03	2.46E-02
4300	ENSG00000105355.8	PLIN3	-0.46	5.32E-03	2.46E-02
4301	ENSG00000273295.1	AP000350.6	-0.99	5.33E-03	2.46E-02
4302	ENSG00000205937.11	RNPS1	-0.29	5.34E-03	2.46E-02
4303	ENSG00000184677.17	ZBTB40	0.28	5.34E-03	2.46E-02
4304	ENSG00000244754.8	N4BP2L2	-0.26	5.34E-03	2.46E-02
4305	ENSG00000112701.17	SENP6	-0.26	5.36E-03	2.47E-02
4306	ENSG00000177685.16	CRACR2B	0.99	5.37E-03	2.48E-02
4307	ENSG00000171824.13	EXOSC10	-0.32	5.37E-03	2.48E-02
4308	ENSG00000140564.11	FURIN	0.38	5.38E-03	2.48E-02
4309	ENSG00000166173.10	LARP6	-0.30	5.38E-03	2.48E-02
4310	ENSG00000128567.16	PODXL	-0.58	5.38E-03	2.48E-02
4311	ENSG00000125967.16	NECAB3	0.44	5.38E-03	2.48E-02
4312	ENSG00000142279.12	WTIP	-0.44	5.38E-03	2.48E-02
4313	ENSG00000165512.4	ZNF22	-0.41	5.39E-03	2.48E-02
4314	ENSG00000160339.15	FCN2	-1.26	5.39E-03	2.48E-02
4315	ENSG00000101084.17	RAB5IF	0.36	5.40E-03	2.48E-02
4316	ENSG00000120322.3	PCDHB8	-1.37	5.40E-03	2.48E-02
4317	ENSG00000100644.16	HIF1A	-0.25	5.40E-03	2.48E-02
4318	ENSG00000102780.16	DGKH	-0.38	5.41E-03	2.49E-02
4319	ENSG00000229314.5	ORM1	1.17	5.41E-03	2.49E-02
4320	ENSG00000164896.19	FASTK	0.34	5.42E-03	2.49E-02
4321	ENSG00000146426.18	TIAM2	-0.58	5.44E-03	2.50E-02
4322	ENSG00000266074.8	BAHCC1	-0.51	5.46E-03	2.51E-02
4323	ENSG00000005007.12	UPF1	0.32	5.46E-03	2.51E-02
4324	ENSG00000177030.16	DEAF1	0.35	5.46E-03	2.51E-02
4325	ENSG00000144837.8	PLA1A	1.57	5.47E-03	2.51E-02
4326	ENSG00000247077.6	PGAM5	0.32	5.47E-03	2.51E-02
4327	ENSG00000173366.11	AC097637.1	-1.98	5.47E-03	2.51E-02
4328	ENSG00000205363.5	INSYN1	0.54	5.49E-03	2.52E-02
4329	ENSG00000137804.12	NUSAP1	-1.24	5.49E-03	2.52E-02
4330	ENSG00000100097.11	LGALS1	0.32	5.50E-03	2.52E-02
4331	ENSG00000196214.10	ZNF766	-0.43	5.50E-03	2.52E-02
4332	ENSG00000143393.16	PI4KB	0.26	5.50E-03	2.52E-02
4333	ENSG00000198885.9	ITPRIPL1	1.51	5.51E-03	2.52E-02
4334	ENSG00000163104.17	SMARCAD1	-0.32	5.51E-03	2.52E-02
4335	ENSG00000140199.11	SLC12A6	-0.39	5.52E-03	2.53E-02
4336	ENSG00000130159.13	ECSIT	0.41	5.53E-03	2.53E-02
4337	ENSG00000132297.11	HHLA1	1.50	5.53E-03	2.53E-02
4338	ENSG00000213186.7	TRIM59	-0.45	5.54E-03	2.53E-02
4339	ENSG00000099957.16	P2RX6	-0.50	5.54E-03	2.53E-02
4340	ENSG00000185896.10	LAMP1	-0.25	5.54E-03	2.54E-02
4341	ENSG00000026025.15	VIM	-0.39	5.55E-03	2.54E-02
4342	ENSG00000143811.18	PYCR2	0.31	5.55E-03	2.54E-02
4343	ENSG00000138135.6	CH25H	-1.03	5.57E-03	2.54E-02
4344	ENSG00000244094.1	SPRR2F	4.85	5.57E-03	2.55E-02
4345	ENSG00000197694.15	SPTAN1	0.26	5.57E-03	2.55E-02
4346	ENSG00000214814.7	FER1L6	-1.33	5.58E-03	2.55E-02
4347	ENSG00000117906.13	RCN2	-0.34	5.62E-03	2.57E-02

4348	ENSG00000105443.14	CYTH2	-0.28	5.62E-03	2.57E-02
4349	ENSG00000184985.16	SORCS2	-0.66	5.64E-03	2.57E-02
4350	ENSG00000183011.13	NAA38	0.47	5.64E-03	2.57E-02
4351	ENSG00000240038.6	AMY2B	-0.47	5.64E-03	2.57E-02
4352	ENSG00000169744.12	LDB2	-0.46	5.65E-03	2.58E-02
4353	ENSG00000072958.8	AP1M1	-0.34	5.65E-03	2.58E-02
4354	ENSG00000151131.10	C12orf45	0.35	5.66E-03	2.58E-02
4355	ENSG00000204130.13	RUFY2	-0.35	5.66E-03	2.58E-02
4356	ENSG00000085511.19	MAP3K4	0.32	5.66E-03	2.58E-02
4357	ENSG00000164304.15	CAGE1	1.79	5.66E-03	2.58E-02
4358	ENSG00000137203.11	TFAP2A	-1.03	5.67E-03	2.58E-02
4359	ENSG00000120820.12	GLT8D2	-0.56	5.67E-03	2.58E-02
4360	ENSG00000177096.8	PHETA2	-0.51	5.68E-03	2.58E-02
4361	ENSG00000168393.12	DTYMK	0.39	5.68E-03	2.59E-02
4362	ENSG00000196227.10	FAM217B	0.34	5.69E-03	2.59E-02
4363	ENSG00000099817.11	POLR2E	0.39	5.69E-03	2.59E-02
4364	ENSG00000110057.7	UNC93B1	-0.90	5.72E-03	2.60E-02
4365	ENSG00000007168.12	PAFAH1B1	-0.24	5.72E-03	2.60E-02
4366	ENSG00000055163.19	CYFIP2	0.64	5.73E-03	2.61E-02
4367	ENSG00000251298.1	AC093835.1	-1.15	5.75E-03	2.61E-02
4368	ENSG00000133884.9	DPF2	-0.30	5.75E-03	2.62E-02
4369	ENSG00000168421.12	RHOH	1.83	5.76E-03	2.62E-02
4370	ENSG00000150593.17	PDCD4	-0.26	5.77E-03	2.62E-02
4371	ENSG00000186174.12	BCL9L	-0.41	5.77E-03	2.62E-02
4372	ENSG00000223799.1	IL10RB-DT	1.04	5.77E-03	2.62E-02
4373	ENSG00000173214.5	MFSD4B	0.49	5.78E-03	2.62E-02
4374	ENSG00000138131.3	LOXL4	-0.97	5.78E-03	2.62E-02
4375	ENSG00000198242.13	RPL23A	0.29	5.79E-03	2.63E-02
4376	ENSG00000005448.16	WDR54	-1.20	5.79E-03	2.63E-02
4377	ENSG00000267318.1	AC005702.1	1.73	5.80E-03	2.63E-02
4378	ENSG00000086619.13	ERO1B	0.58	5.80E-03	2.63E-02
4379	ENSG00000232623.1	AP000266.1	1.23	5.82E-03	2.64E-02
4380	ENSG00000270170.1	NCBP2-AS2	0.44	5.82E-03	2.64E-02
4381	ENSG00000090339.8	ICAM1	-0.59	5.83E-03	2.64E-02
4382	ENSG00000129195.15	PIMREG	-2.73	5.84E-03	2.64E-02
4383	ENSG00000279821.1	AC145098.2	-0.97	5.85E-03	2.65E-02
4384	ENSG00000261762.1	AC027228.2	1.45	5.85E-03	2.65E-02
4385	ENSG00000215883.10	CYB5RL	0.42	5.86E-03	2.65E-02
4386	ENSG00000135842.16	FAM129A	-0.41	5.86E-03	2.65E-02
4387	ENSG00000257949.6	TEN1	0.50	5.87E-03	2.66E-02
4388	ENSG00000117143.13	UAP1	0.34	5.88E-03	2.66E-02
4389	ENSG00000153879.8	CEBPG	0.28	5.88E-03	2.66E-02
4390	ENSG00000163701.18	IL17RE	-0.76	5.89E-03	2.67E-02
4391	ENSG00000132964.11	CDK8	0.37	5.92E-03	2.68E-02
4392	ENSG00000017427.16	IGF1	-0.32	5.94E-03	2.68E-02
4393	ENSG00000265590.9	AP000275.2	-1.27	5.94E-03	2.68E-02
4394	ENSG00000141198.15	TOM1L1	-0.43	5.95E-03	2.69E-02
4395	ENSG00000243335.9	KCTD7	0.28	5.95E-03	2.69E-02
4396	ENSG00000179051.13	RCC2	0.29	5.95E-03	2.69E-02
4397	ENSG00000232442.1	MHENCR	0.86	5.96E-03	2.69E-02

4398	ENSG00000182685.7	BRICD5	-0.97	6.00E-03	2.71E-02
4399	ENSG00000263394.1	AC007952.7	2.28	6.01E-03	2.71E-02
4400	ENSG00000145777.14	TSLP	0.90	6.02E-03	2.72E-02
4401	ENSG00000138801.8	PAPSS1	-0.35	6.03E-03	2.72E-02
4402	ENSG00000203930.11	LINC00632	0.32	6.03E-03	2.72E-02
4403	ENSG00000170892.10	TSEN34	0.40	6.04E-03	2.72E-02
4404	ENSG00000176101.11	SSNA1	0.42	6.04E-03	2.72E-02
4405	ENSG00000122691.12	TWIST1	-0.42	6.05E-03	2.72E-02
4406	ENSG00000127540.11	UQCR11	0.29	6.06E-03	2.73E-02
4407	ENSG00000196455.7	PIK3R4	0.26	6.11E-03	2.75E-02
4408	ENSG00000112208.11	BAG2	-0.36	6.12E-03	2.76E-02
4409	ENSG00000170775.2	GPR37	-0.41	6.13E-03	2.76E-02
4410	ENSG00000250821.2	EXOC1L	1.83	6.14E-03	2.76E-02
4411	ENSG00000018280.16	SLC11A1	1.55	6.14E-03	2.76E-02
4412	ENSG00000250012.1	AC079848.1	1.42	6.15E-03	2.77E-02
4413	ENSG00000259330.2	INAFM2	-0.37	6.16E-03	2.77E-02
4414	ENSG00000169071.14	ROR2	-0.82	6.18E-03	2.78E-02
4415	ENSG00000155816.19	FMN2	-0.71	6.18E-03	2.78E-02
4416	ENSG00000047932.13	GOPC	-0.26	6.19E-03	2.78E-02
4417	ENSG00000125779.22	PANK2	-0.31	6.21E-03	2.79E-02
4418	ENSG00000101280.7	ANGPT4	-0.85	6.21E-03	2.79E-02
4419	ENSG00000225205.5	AC078883.1	0.97	6.21E-03	2.79E-02
4420	ENSG00000221988.12	PPT2	0.45	6.23E-03	2.80E-02
4421	ENSG00000196072.11	BLOC1S2	-0.37	6.23E-03	2.80E-02
4422	ENSG00000134809.8	TIMM10	0.40	6.23E-03	2.80E-02
4423	ENSG00000122557.9	HERPUD2	-0.35	6.26E-03	2.81E-02
4424	ENSG00000171819.4	ANGPTL7	-2.42	6.27E-03	2.81E-02
4425	ENSG00000114544.16	SLC41A3	0.34	6.27E-03	2.81E-02
4426	ENSG00000233821.1	ENOX1-AS1	3.03	6.28E-03	2.82E-02
4427	ENSG00000167771.5	RCOR2	-1.12	6.28E-03	2.82E-02
4428	ENSG00000104064.17	GABPB1	-0.38	6.29E-03	2.82E-02
4429	ENSG00000197479.6	PCDHB11	-0.90	6.30E-03	2.82E-02
4430	ENSG00000232931.5	LINC00342	-0.48	6.31E-03	2.83E-02
4431	ENSG00000173406.15	DAB1	-0.64	6.31E-03	2.83E-02
4432	ENSG00000197561.6	ELANE	-2.03	6.31E-03	2.83E-02
4433	ENSG00000221990.4	EXOC3-AS1	0.58	6.32E-03	2.83E-02
4434	ENSG00000171813.13	PWWP2B	0.48	6.33E-03	2.83E-02
4435	ENSG00000110422.11	HIPK3	0.25	6.34E-03	2.84E-02
4436	ENSG00000169902.14	TPST1	-0.35	6.34E-03	2.84E-02
4437	ENSG00000128283.6	CDC42EP1	-0.49	6.36E-03	2.84E-02
4438	ENSG00000178031.16	ADAMTSL1	-1.18	6.37E-03	2.85E-02
4439	ENSG00000184154.14	LRTOMT	-0.59	6.40E-03	2.86E-02
4440	ENSG00000100181.22	TPTEP1	0.65	6.41E-03	2.87E-02
4441	ENSG00000054967.12	RELT	0.97	6.41E-03	2.87E-02
4442	ENSG00000184110.14	EIF3C	0.24	6.44E-03	2.88E-02
4443	ENSG00000198040.10	ZNF84	-0.29	6.44E-03	2.88E-02
4444	ENSG00000168092.13	PAFAH1B2	-0.26	6.44E-03	2.88E-02
4445	ENSG00000164070.11	HSPA4L	-0.45	6.47E-03	2.89E-02
4446	ENSG00000101546.12	RBFA	0.34	6.47E-03	2.89E-02
4447	ENSG00000250182.3	EEF1A1P13	0.72	6.47E-03	2.89E-02

4448	ENSG00000105357.16	MYH14	0.45	6.48E-03	2.89E-02
4449	ENSG00000183864.4	TOB2	0.33	6.49E-03	2.89E-02
4450	ENSG00000163961.4	RNF168	-0.29	6.50E-03	2.90E-02
4451	ENSG00000139182.14	CLSTN3	-0.65	6.53E-03	2.91E-02
4452	ENSG00000196911.10	KPNA5	0.33	6.53E-03	2.91E-02
4453	ENSG00000104497.13	SNX16	0.40	6.54E-03	2.91E-02
4454	ENSG00000067715.13	SYT1	-0.97	6.54E-03	2.92E-02
4455	ENSG00000177879.15	AP3S1	-0.34	6.55E-03	2.92E-02
4456	ENSG00000125744.11	RTN2	-0.72	6.58E-03	2.93E-02
4457	ENSG00000122203.14	KIAA1191	0.25	6.59E-03	2.93E-02
4458	ENSG00000261625.1	AP003071.4	-1.92	6.59E-03	2.93E-02
4459	ENSG00000261487.1	AC135048.1	-0.78	6.59E-03	2.94E-02
4460	ENSG00000128342.4	LIF	-0.81	6.60E-03	2.94E-02
4461	ENSG00000008256.15	CYTH3	-0.32	6.63E-03	2.95E-02
4462	ENSG00000156531.16	PHF6	0.31	6.66E-03	2.96E-02
4463	ENSG00000181029.8	TRAPPC5	0.67	6.66E-03	2.96E-02
4464	ENSG00000160318.6	CLDND2	1.07	6.66E-03	2.96E-02
4465	ENSG00000166503.8	HDGFL3	-0.40	6.68E-03	2.97E-02
4466	ENSG00000062598.17	ELMO2	0.26	6.68E-03	2.97E-02
4467	ENSG00000009830.11	POMT2	0.36	6.69E-03	2.97E-02
4468	ENSG00000101247.17	NDUFAF5	0.34	6.70E-03	2.98E-02
4469	ENSG00000108439.10	PNPO	0.34	6.70E-03	2.98E-02
4470	ENSG00000169442.8	CD52	2.87	6.72E-03	2.98E-02
4471	ENSG00000246263.2	UBR5-AS1	0.51	6.72E-03	2.98E-02
4472	ENSG00000090266.12	NDUFB2	0.28	6.72E-03	2.98E-02
4473	ENSG00000279713.1	AC080038.3	-1.89	6.72E-03	2.98E-02
4474	ENSG00000241852.9	C8orf58	-0.50	6.73E-03	2.99E-02
4475	ENSG00000108515.17	ENO3	0.58	6.75E-03	2.99E-02
4476	ENSG00000137693.13	YAP1	-0.25	6.74E-03	2.99E-02
4477	ENSG00000143337.18	TOR1AIP1	-0.26	6.74E-03	2.99E-02
4478	ENSG00000182095.14	TNRC18	-0.42	6.76E-03	2.99E-02
4479	ENSG00000006118.14	TMEM132A	-0.64	6.76E-03	2.99E-02
4480	ENSG00000155961.4	RAB39B	-1.42	6.76E-03	2.99E-02
4481	ENSG00000104237.9	RP1	1.68	6.76E-03	3.00E-02
4482	ENSG00000083444.16	PLOD1	-0.32	6.77E-03	3.00E-02
4483	ENSG00000262209.2	PCDHGB3	-0.69	6.77E-03	3.00E-02
4484	ENSG00000145819.16	ARHGAP26	-0.36	6.77E-03	3.00E-02
4485	ENSG00000198546.14	ZNF511	0.48	6.79E-03	3.00E-02
4486	ENSG00000067221.13	STOML1	0.39	6.79E-03	3.00E-02
4487	ENSG00000114646.9	CSPG5	-0.75	6.79E-03	3.00E-02
4488	ENSG00000268575.1	AL031282.2	-0.53	6.80E-03	3.01E-02
4489	ENSG00000232940.5	HCG25	-0.72	6.81E-03	3.01E-02
4490	ENSG00000169684.13	CHRNA5	1.56	6.82E-03	3.02E-02
4491	ENSG00000175470.19	PPP2R2D	0.30	6.83E-03	3.02E-02
4492	ENSG00000151967.18	SCHIP1	-1.04	6.84E-03	3.02E-02
4493	ENSG00000122490.18	PQLC1	0.40	6.84E-03	3.02E-02
4494	ENSG00000005882.11	PDK2	0.29	6.85E-03	3.02E-02
4495	ENSG00000265263.1	AC124066.1	-0.76	6.87E-03	3.03E-02
4496	ENSG00000260804.3	LINC01963	-0.43	6.87E-03	3.03E-02
4497	ENSG00000058085.14	LAMC2	-0.83	6.88E-03	3.04E-02

4498	ENSG00000183155.4	RABIF	0.33	6.89E-03	3.04E-02
4499	ENSG00000038382.19	TRIO	-0.27	6.88E-03	3.04E-02
4500	ENSG00000196712.17	NF1	0.29	6.89E-03	3.04E-02
4501	ENSG00000182154.7	MRPL41	0.46	6.89E-03	3.04E-02
4502	ENSG00000164877.18	MICALL2	-0.61	6.91E-03	3.05E-02
4503	ENSG00000165915.13	SLC39A13	-0.41	6.92E-03	3.05E-02
4504	ENSG00000144677.14	CTDSPL	-0.31	6.93E-03	3.05E-02
4505	ENSG00000132763.14	MMACHC	0.35	6.96E-03	3.07E-02
4506	ENSG00000224559.2	LINC01087	-1.23	6.96E-03	3.07E-02
4507	ENSG00000145431.10	PDGFC	-0.34	6.96E-03	3.07E-02
4508	ENSG00000139679.15	LPAR6	-0.47	6.97E-03	3.07E-02
4509	ENSG00000143013.12	LMO4	-0.29	6.97E-03	3.07E-02
4510	ENSG00000130224.14	LRCH2	-0.61	6.98E-03	3.07E-02
4511	ENSG00000181004.9	BBS12	-0.55	6.99E-03	3.07E-02
4512	ENSG00000198947.15	DMD	-0.40	6.99E-03	3.08E-02
4513	ENSG00000007933.12	FMO3	1.75	7.00E-03	3.08E-02
4514	ENSG00000253293.4	HOXA10	-0.34	7.01E-03	3.08E-02
4515	ENSG00000163947.11	ARHGEF3	-0.36	7.01E-03	3.08E-02
4516	ENSG00000142303.13	ADAMTS10	-0.67	7.02E-03	3.09E-02
4517	ENSG00000125746.16	EML2	-0.48	7.05E-03	3.10E-02
4518	ENSG00000258643.5	BCL2L2-PABPN1	0.60	7.06E-03	3.10E-02
4519	ENSG00000047849.21	MAP4	-0.25	7.06E-03	3.10E-02
4520	ENSG00000135643.4	KCNMB4	-1.37	7.08E-03	3.11E-02
4521	ENSG00000114491.13	UMPS	0.37	7.08E-03	3.11E-02
4522	ENSG00000111962.7	UST	-0.34	7.10E-03	3.12E-02
4523	ENSG00000132530.16	XAF1	-0.35	7.12E-03	3.12E-02
4524	ENSG00000231991.4	ANXA2P2	-1.41	7.12E-03	3.13E-02
4525	ENSG00000275026.1	GXYLT1P4	2.78	7.13E-03	3.13E-02
4526	ENSG00000116044.15	NFE2L2	-0.26	7.15E-03	3.14E-02
4527	ENSG00000250067.11	YJEFN3	-0.90	7.19E-03	3.15E-02
4528	ENSG00000245552.6	AP000787.1	-0.98	7.19E-03	3.15E-02
4529	ENSG00000113068.9	PFDN1	0.27	7.20E-03	3.16E-02
4530	ENSG00000258232.2	AC125611.3	-0.74	7.21E-03	3.16E-02
4531	ENSG00000058600.15	POLR3E	0.32	7.21E-03	3.16E-02
4532	ENSG00000156374.15	PCGF6	0.39	7.22E-03	3.16E-02
4533	ENSG00000049130.15	KITLG	-0.58	7.22E-03	3.16E-02
4534	ENSG00000164506.14	STXBP5	-0.26	7.22E-03	3.16E-02
4535	ENSG00000166016.5	ABTB2	0.46	7.23E-03	3.17E-02
4536	ENSG00000273252.1	OR7E39P	1.83	7.26E-03	3.18E-02
4537	ENSG00000225265.1	TAF1A-AS1	0.66	7.26E-03	3.18E-02
4538	ENSG00000129083.12	COPB1	-0.32	7.26E-03	3.18E-02
4539	ENSG00000118939.17	UCHL3	0.41	7.27E-03	3.18E-02
4540	ENSG00000156515.23	HK1	-0.26	7.27E-03	3.18E-02
4541	ENSG00000168216.11	LMBRD1	-0.31	7.29E-03	3.19E-02
4542	ENSG00000135205.14	CCDC146	-0.69	7.31E-03	3.19E-02
4543	ENSG00000197181.11	PIWIL2	-0.85	7.32E-03	3.20E-02
4544	ENSG00000196323.13	ZBTB44	0.29	7.33E-03	3.20E-02
4545	ENSG00000132824.13	SERINC3	-0.23	7.33E-03	3.20E-02
4546	ENSG00000073756.11	PTGS2	-0.75	7.34E-03	3.20E-02
4547	ENSG00000169032.9	MAP2K1	-0.30	7.34E-03	3.21E-02

4548	ENSG00000105778.18	AVL9	0.34	7.34E-03	3.21E-02
4549	ENSG00000144043.11	TEX261	0.23	7.35E-03	3.21E-02
4550	ENSG00000120913.23	PDLIM2	-0.40	7.36E-03	3.21E-02
4551	ENSG00000229638.1	RPL4P4	1.10	7.38E-03	3.22E-02
4552	ENSG00000170214.4	ADRA1B	1.05	7.38E-03	3.22E-02
4553	ENSG00000143994.13	ABHD1	0.88	7.40E-03	3.22E-02
4554	ENSG00000130513.6	GDF15	-0.67	7.40E-03	3.22E-02
4555	ENSG00000167515.10	TRAPPC2L	0.34	7.40E-03	3.23E-02
4556	ENSG00000133104.13	SPART	-0.28	7.41E-03	3.23E-02
4557	ENSG00000278376.1	AP004609.3	1.05	7.42E-03	3.23E-02
4558	ENSG00000253392.2	AC119403.1	1.71	7.47E-03	3.25E-02
4559	ENSG00000223797.5	ENTPD3-AS1	0.61	7.47E-03	3.25E-02
4560	ENSG00000123416.15	TUBA1B	-0.43	7.47E-03	3.25E-02
4561	ENSG00000160310.17	PRMT2	-0.28	7.48E-03	3.25E-02
4562	ENSG00000267272.5	LINC01140	-0.47	7.50E-03	3.27E-02
4563	ENSG00000099203.6	TMED1	0.41	7.51E-03	3.27E-02
4564	ENSG00000149970.15	CNKSR2	-0.53	7.53E-03	3.27E-02
4565	ENSG00000155903.11	RASA2	-0.45	7.54E-03	3.28E-02
4566	ENSG00000082213.17	C5orf22	0.29	7.55E-03	3.28E-02
4567	ENSG00000094963.13	FMO2	1.32	7.57E-03	3.29E-02
4568	ENSG00000177054.13	ZDHHC13	0.46	7.57E-03	3.29E-02
4569	ENSG00000175806.14	MSRA	0.35	7.57E-03	3.29E-02
4570	ENSG00000114738.10	MAPKAPK3	0.33	7.57E-03	3.29E-02
4571	ENSG00000125691.12	RPL23	0.25	7.57E-03	3.29E-02
4572	ENSG00000114999.7	TTL	-0.27	7.57E-03	3.29E-02
4573	ENSG00000100605.16	ITPK1	0.36	7.58E-03	3.29E-02
4574	ENSG00000249487.6	LINC01586	1.82	7.61E-03	3.30E-02
4575	ENSG00000115947.13	ORC4	0.29	7.61E-03	3.30E-02
4576	ENSG00000146374.13	RSPO3	-0.45	7.62E-03	3.31E-02
4577	ENSG00000167566.16	NCKAP5L	-0.42	7.63E-03	3.31E-02
4578	ENSG00000189367.14	KIAA0408	-0.45	7.63E-03	3.31E-02
4579	ENSG00000253719.3	ATXN7L3B	-0.24	7.65E-03	3.32E-02
4580	ENSG00000148450.12	MSRB2	-0.32	7.65E-03	3.32E-02
4581	ENSG00000164241.13	C5orf63	0.48	7.66E-03	3.32E-02
4582	ENSG00000125249.6	RAP2A	-0.30	7.66E-03	3.32E-02
4583	ENSG00000147889.17	CDKN2A	-0.43	7.66E-03	3.32E-02
4584	ENSG00000121067.17	SPOP	-0.27	7.68E-03	3.33E-02
4585	ENSG00000126267.9	COX6B1	0.23	7.70E-03	3.33E-02
4586	ENSG00000070882.12	OSBPL3	-0.44	7.74E-03	3.35E-02
4587	ENSG00000100599.15	RIN3	-0.49	7.73E-03	3.35E-02
4588	ENSG00000116574.5	RHOU	-0.34	7.74E-03	3.35E-02
4589	ENSG00000186188.10	FFAR4	0.45	7.78E-03	3.37E-02
4590	ENSG00000107798.17	LIPA	0.31	7.79E-03	3.37E-02
4591	ENSG00000180611.6	MB21D2	-0.58	7.80E-03	3.37E-02
4592	ENSG00000242372.7	EIF6	0.28	7.81E-03	3.37E-02
4593	ENSG00000198691.12	ABCA4	0.70	7.81E-03	3.38E-02
4594	ENSG00000259132.1	AL132780.3	-0.96	7.83E-03	3.39E-02
4595	ENSG00000131018.22	SYNE1	-0.34	7.84E-03	3.39E-02
4596	ENSG00000099977.14	DDT	0.30	7.85E-03	3.39E-02
4597	ENSG00000183726.10	TMEM50A	-0.31	7.85E-03	3.39E-02

4598	ENSG00000112290.12	WASF1	-0.27	7.86E-03	3.39E-02
4599	ENSG00000181035.13	SLC25A42	0.33	7.87E-03	3.40E-02
4600	ENSG00000187266.13	EPOR	0.87	7.88E-03	3.40E-02
4601	ENSG00000246067.7	RAB30-AS1	0.57	7.89E-03	3.40E-02
4602	ENSG00000135063.19	FAM189A2	1.19	7.90E-03	3.41E-02
4603	ENSG00000078699.21	CBFA2T2	-0.33	7.90E-03	3.41E-02
4604	ENSG00000099968.17	BCL2L13	0.25	7.90E-03	3.41E-02
4605	ENSG00000142945.12	KIF2C	-1.79	7.90E-03	3.41E-02
4606	ENSG00000167635.11	ZNF146	-0.33	7.91E-03	3.41E-02
4607	ENSG00000122643.19	NT5C3A	-0.46	7.91E-03	3.41E-02
4608	ENSG00000171951.4	SCG2	-1.05	7.91E-03	3.41E-02
4609	ENSG00000119630.13	PGF	-1.17	7.92E-03	3.41E-02
4610	ENSG00000163655.15	GMPS	-0.28	7.93E-03	3.42E-02
4611	ENSG00000183570.16	PCBP3	-0.65	7.94E-03	3.42E-02
4612	ENSG00000132676.15	DAP3	0.25	7.95E-03	3.42E-02
4613	ENSG00000078018.19	MAP2	-0.62	7.95E-03	3.42E-02
4614	ENSG00000137478.14	FCHSD2	0.33	7.96E-03	3.42E-02
4615	ENSG00000151702.16	FLI1	-0.63	7.96E-03	3.42E-02
4616	ENSG00000256087.6	ZNF432	-0.40	7.98E-03	3.43E-02
4617	ENSG00000135338.13	LCA5	-0.55	7.98E-03	3.43E-02
4618	ENSG00000150054.18	MPP7	0.33	8.00E-03	3.44E-02
4619	ENSG00000285634.1	AL583827.1	0.62	8.01E-03	3.44E-02
4620	ENSG00000166526.16	ZNF3	-0.33	8.02E-03	3.44E-02
4621	ENSG00000151303.11	AL136982.1	0.78	8.03E-03	3.45E-02
4622	ENSG00000134259.3	NGF	-0.76	8.04E-03	3.45E-02
4623	ENSG00000187210.13	GCNT1	-0.56	8.04E-03	3.45E-02
4624	ENSG00000112893.9	MAN2A1	-0.30	8.04E-03	3.45E-02
4625	ENSG00000166862.6	CACNG2	2.38	8.05E-03	3.46E-02
4626	ENSG00000105767.2	CADM4	-0.62	8.05E-03	3.46E-02
4627	ENSG00000224078.13	SNHG14	-0.28	8.10E-03	3.48E-02
4628	ENSG00000102349.17	KLF8	-0.64	8.11E-03	3.48E-02
4629	ENSG00000261693.1	AC134682.1	1.52	8.14E-03	3.49E-02
4630	ENSG00000253846.2	PCDHGA10	-0.38	8.14E-03	3.49E-02
4631	ENSG00000254928.1	AP001372.3	-2.98	8.15E-03	3.50E-02
4632	ENSG00000106603.18	COA1	0.32	8.16E-03	3.50E-02
4633	ENSG00000138468.15	SENP7	-0.39	8.16E-03	3.50E-02
4634	ENSG00000143819.12	EPHX1	-0.34	8.17E-03	3.50E-02
4635	ENSG00000234036.4	TXNP6	0.80	8.18E-03	3.50E-02
4636	ENSG00000123892.11	RAB38	1.36	8.19E-03	3.51E-02
4637	ENSG00000114790.12	ARHGEF26	0.63	8.19E-03	3.51E-02
4638	ENSG00000012171.19	SEMA3B	-0.90	8.19E-03	3.51E-02
4639	ENSG00000117152.13	RGS4	-0.53	8.20E-03	3.51E-02
4640	ENSG00000196177.12	ACADSB	0.35	8.26E-03	3.53E-02
4641	ENSG00000255236.2	AP002992.1	1.77	8.26E-03	3.53E-02
4642	ENSG00000214447.4	FAM187A	1.13	8.26E-03	3.53E-02
4643	ENSG00000182359.14	KBTBD3	-0.70	8.28E-03	3.54E-02
4644	ENSG00000267023.5	LRRC37A16P	0.47	8.29E-03	3.54E-02
4645	ENSG00000154025.15	SLC5A10	2.11	8.31E-03	3.55E-02
4646	ENSG00000166165.12	CKB	-0.42	8.31E-03	3.55E-02
4647	ENSG00000116489.12	CAPZA1	-0.28	8.31E-03	3.55E-02

4648	ENSG00000174327.6	SLC16A13	1.07	8.33E-03	3.56E-02
4649	ENSG00000109689.15	STIM2	-0.37	8.35E-03	3.56E-02
4650	ENSG00000223547.9	ZNF844	-0.44	8.35E-03	3.56E-02
4651	ENSG00000255920.2	CCND2-AS1	-0.72	8.37E-03	3.57E-02
4652	ENSG00000068781.21	STON1-GTF2A1L	3.48	8.37E-03	3.57E-02
4653	ENSG00000147164.11	SNX12	-0.29	8.41E-03	3.59E-02
4654	ENSG00000164093.16	PITX2	-0.37	8.41E-03	3.59E-02
4655	ENSG00000235106.9	BRD3OS	-0.33	8.42E-03	3.59E-02
4656	ENSG00000284196.2	AL158064.1	0.57	8.42E-03	3.59E-02
4657	ENSG00000127922.9	SEM1	0.28	8.43E-03	3.59E-02
4658	ENSG00000175224.16	ATG13	-0.30	8.43E-03	3.59E-02
4659	ENSG00000107554.16	DNMBP	-0.34	8.43E-03	3.59E-02
4660	ENSG00000170734.11	POLH	-0.35	8.45E-03	3.60E-02
4661	ENSG00000106459.14	NRF1	-0.43	8.45E-03	3.60E-02
4662	ENSG00000082458.11	DLG3	-0.59	8.49E-03	3.62E-02
4663	ENSG00000196196.2	HRCT1	-0.84	8.49E-03	3.62E-02
4664	ENSG00000197332.8	AC008543.1	1.07	8.51E-03	3.62E-02
4665	ENSG00000106615.9	RHEB	0.28	8.53E-03	3.63E-02
4666	ENSG00000215790.7	SLC35E2A	-0.38	8.54E-03	3.63E-02
4667	ENSG00000090905.18	TNRC6A	-0.26	8.56E-03	3.64E-02
4668	ENSG00000167302.10	TEPSIN	-0.44	8.56E-03	3.64E-02
4669	ENSG00000156502.13	SUPV3L1	0.29	8.57E-03	3.64E-02
4670	ENSG00000166448.14	TMEM130	-0.63	8.57E-03	3.64E-02
4671	ENSG00000174527.9	MYO1H	-2.98	8.57E-03	3.64E-02
4672	ENSG00000248919.7	ATP5MF-PTCD1	0.51	8.57E-03	3.64E-02
4673	ENSG00000102893.15	PHKB	-0.30	8.58E-03	3.65E-02
4674	ENSG00000188000.4	OR7D2	1.11	8.59E-03	3.65E-02
4675	ENSG00000163017.13	ACTG2	0.71	8.62E-03	3.66E-02
4676	ENSG00000171858.17	RPS21	0.36	8.64E-03	3.67E-02
4677	ENSG00000101220.17	C20orf27	0.43	8.65E-03	3.67E-02
4678	ENSG00000174080.10	CTSF	-0.35	8.65E-03	3.67E-02
4679	ENSG00000069998.12	HDHD5	0.34	8.67E-03	3.68E-02
4680	ENSG00000167476.10	JSRP1	0.39	8.68E-03	3.68E-02
4681	ENSG00000285649.1	AL357079.2	1.53	8.70E-03	3.69E-02
4682	ENSG00000176407.17	KCMF1	0.28	8.71E-03	3.69E-02
4683	ENSG00000189079.15	ARID2	-0.28	8.71E-03	3.69E-02
4684	ENSG00000125901.5	MRPS26	0.36	8.73E-03	3.70E-02
4685	ENSG00000204628.11	RACK1	0.24	8.73E-03	3.70E-02
4686	ENSG00000023839.11	ABCC2	-0.96	8.74E-03	3.70E-02
4687	ENSG00000163435.15	ELF3	2.59	8.74E-03	3.70E-02
4688	ENSG00000176533.12	GNG7	-1.65	8.74E-03	3.70E-02
4689	ENSG00000274512.5	TBC1D3L	-0.51	8.77E-03	3.71E-02
4690	ENSG00000198873.11	GRK5	-0.52	8.80E-03	3.73E-02
4691	ENSG00000159921.15	GNE	0.27	8.82E-03	3.73E-02
4692	ENSG00000186481.16	ANKRD20A5P	-1.02	8.84E-03	3.74E-02
4693	ENSG00000111404.6	RERGL	1.80	8.85E-03	3.74E-02
4694	ENSG00000170430.9	MGMT	0.39	8.87E-03	3.75E-02
4695	ENSG00000162873.14	KLHDC8A	1.58	8.88E-03	3.76E-02
4696	ENSG00000123815.11	COQ8B	-0.43	8.90E-03	3.76E-02
4697	ENSG00000120875.8	DUSP4	-0.39	8.92E-03	3.77E-02

4698	ENSG00000213347.10	MXD3	-1.17	8.92E-03	3.77E-02
4699	ENSG00000161277.10	THAP8	0.50	8.93E-03	3.77E-02
4700	ENSG00000087157.18	PGS1	0.30	8.93E-03	3.77E-02
4701	ENSG00000146701.11	MDH2	0.23	8.93E-03	3.77E-02
4702	ENSG00000182872.15	RBM10	-0.37	8.93E-03	3.77E-02
4703	ENSG00000214517.9	PPME1	-0.27	8.94E-03	3.77E-02
4704	ENSG00000129003.17	VPS13C	-0.25	8.95E-03	3.78E-02
4705	ENSG00000107518.17	ATRNL1	-1.33	8.97E-03	3.78E-02
4706	ENSG00000100151.15	PICK1	0.52	8.97E-03	3.79E-02
4707	ENSG00000068323.16	TFE3	0.24	8.99E-03	3.79E-02
4708	ENSG00000148773.13	MKI67	-3.15	9.02E-03	3.80E-02
4709	ENSG00000166813.14	KIF7	-0.42	9.03E-03	3.81E-02
4710	ENSG00000173926.5	MARCH3	0.58	9.04E-03	3.81E-02
4711	ENSG00000124374.8	PAIP2B	0.55	9.04E-03	3.81E-02
4712	ENSG00000266918.1	AC091132.3	-0.63	9.04E-03	3.81E-02
4713	ENSG00000019144.18	PHLDB1	-0.36	9.04E-03	3.81E-02
4714	ENSG00000188766.12	SPRED3	-1.31	9.08E-03	3.82E-02
4715	ENSG00000058866.14	DGKG	1.47	9.09E-03	3.83E-02
4716	ENSG00000274290.2	HIST1H2BE	1.41	9.10E-03	3.83E-02
4717	ENSG00000120616.15	EPC1	0.29	9.10E-03	3.83E-02
4718	ENSG00000105220.15	GPI	0.25	9.11E-03	3.83E-02
4719	ENSG00000115414.18	FN1	-0.75	9.11E-03	3.83E-02
4720	ENSG00000074071.14	MRPS34	0.35	9.14E-03	3.84E-02
4721	ENSG00000173482.16	PTPRM	0.23	9.14E-03	3.84E-02
4722	ENSG00000146938.15	NLGN4X	0.91	9.14E-03	3.84E-02
4723	ENSG00000242294.6	STAG3L5P	0.52	9.16E-03	3.85E-02
4724	ENSG00000177469.12	CAVIN1	0.30	9.16E-03	3.85E-02
4725	ENSG00000168078.9	PBK	-1.72	9.16E-03	3.85E-02
4726	ENSG00000166167.17	BTRC	0.29	9.25E-03	3.89E-02
4727	ENSG00000267712.5	LINC01539	2.34	9.29E-03	3.90E-02
4728	ENSG00000111678.10	C12orf57	-0.52	9.30E-03	3.91E-02
4729	ENSG00000105889.15	STEAP1B	1.16	9.31E-03	3.91E-02
4730	ENSG00000227082.2	AC244021.1	0.85	9.31E-03	3.91E-02
4731	ENSG00000225648.5	SBDSP1	-0.44	9.31E-03	3.91E-02
4732	ENSG00000198898.13	CAPZA2	-0.24	9.34E-03	3.92E-02
4733	ENSG00000106868.16	SUSD1	-0.37	9.34E-03	3.92E-02
4734	ENSG00000135407.10	AVIL	-0.43	9.34E-03	3.92E-02
4735	ENSG00000230911.1	PPIHP1	1.25	9.35E-03	3.92E-02
4736	ENSG00000196776.15	CD47	-0.34	9.39E-03	3.94E-02
4737	ENSG00000259668.5	AC066613.1	1.18	9.40E-03	3.94E-02
4738	ENSG00000275832.4	ARHGAP23	-0.29	9.41E-03	3.94E-02
4739	ENSG00000234857.2	HNRNPUL2-BSCL2	0.29	9.41E-03	3.94E-02
4740	ENSG00000164920.9	OSR2	-0.43	9.42E-03	3.94E-02
4741	ENSG00000172379.20	ARNT2	-0.54	9.42E-03	3.94E-02
4742	ENSG00000107872.12	FBXL15	0.72	9.42E-03	3.94E-02
4743	ENSG00000144730.17	IL17RD	-0.64	9.42E-03	3.94E-02
4744	ENSG00000140396.12	NCOA2	-0.29	9.43E-03	3.94E-02
4745	ENSG00000105894.11	PTN	-0.83	9.43E-03	3.94E-02
4746	ENSG00000111785.19	RIC8B	0.36	9.43E-03	3.95E-02
4747	ENSG00000277631.4	PGM5P3-AS1	1.77	9.44E-03	3.95E-02

4748	ENSG00000087495.16	PHACTR3	-0.97	9.44E-03	3.95E-02
4749	ENSG00000144061.12	NPHP1	-0.74	9.45E-03	3.95E-02
4750	ENSG00000254221.2	PCDHGB1	-0.77	9.48E-03	3.96E-02
4751	ENSG00000259948.2	AC124068.1	0.94	9.48E-03	3.96E-02
4752	ENSG00000242247.10	ARFGAP3	-0.28	9.50E-03	3.97E-02
4753	ENSG00000176715.16	ACSF3	0.32	9.52E-03	3.98E-02
4754	ENSG00000259848.8	AC097374.1	0.32	9.52E-03	3.98E-02
4755	ENSG00000237172.3	B3GNT9	-0.42	9.53E-03	3.98E-02
4756	ENSG00000144120.12	TMEM177	0.48	9.54E-03	3.98E-02
4757	ENSG00000129696.12	TTI2	0.39	9.54E-03	3.98E-02
4758	ENSG00000101608.12	MYL12A	-0.28	9.55E-03	3.98E-02
4759	ENSG00000162616.8	DNAJB4	-0.38	9.55E-03	3.98E-02
4760	ENSG00000077713.18	SLC25A43	-0.41	9.56E-03	3.99E-02
4761	ENSG00000067167.7	TRAM1	-0.28	9.58E-03	4.00E-02
4762	ENSG00000273784.4	AL137058.2	-1.57	9.58E-03	4.00E-02
4763	ENSG00000258920.1	FOXN3-AS1	0.75	9.59E-03	4.00E-02
4764	ENSG00000131051.22	RBM39	-0.24	9.60E-03	4.00E-02
4765	ENSG00000166681.13	BEX3	-0.26	9.60E-03	4.00E-02
4766	ENSG00000198420.9	TCAF1	0.28	9.61E-03	4.00E-02
4767	ENSG00000100941.8	PNN	-0.25	9.61E-03	4.00E-02
4768	ENSG00000106460.18	TMEM106B	-0.25	9.63E-03	4.01E-02
4769	ENSG00000134256.12	CD101	1.89	9.64E-03	4.01E-02
4770	ENSG00000276043.4	UHRF1	-1.30	9.64E-03	4.01E-02
4771	ENSG00000016391.10	CHDH	0.89	9.65E-03	4.02E-02
4772	ENSG00000118046.14	STK11	0.31	9.66E-03	4.02E-02
4773	ENSG00000266340.1	AC138207.7	-0.60	9.67E-03	4.02E-02
4774	ENSG00000175701.10	MTLN	0.53	9.68E-03	4.03E-02
4775	ENSG00000170917.13	NUDT6	0.53	9.69E-03	4.03E-02
4776	ENSG00000042493.15	CAPG	-0.39	9.72E-03	4.04E-02
4777	ENSG00000115827.13	DCAF17	-0.33	9.73E-03	4.04E-02
4778	ENSG00000279419.1	AC004925.1	-1.01	9.73E-03	4.04E-02
4779	ENSG00000139154.14	AEBP2	0.29	9.74E-03	4.05E-02
4780	ENSG00000171148.13	TADA3	-0.29	9.74E-03	4.05E-02
4781	ENSG00000172482.4	AGXT	1.67	9.76E-03	4.05E-02
4782	ENSG00000254726.2	MEX3A	-0.69	9.76E-03	4.05E-02
4783	ENSG00000148690.11	FRA10AC1	-0.31	9.76E-03	4.05E-02
4784	ENSG00000139567.12	ACVRL1	-0.58	9.78E-03	4.06E-02
4785	ENSG00000198496.11	NBR2	0.45	9.79E-03	4.06E-02
4786	ENSG00000182749.5	PAQR7	-0.45	9.79E-03	4.06E-02
4787	ENSG00000280401.1	AC022532.1	1.93	9.80E-03	4.06E-02
4788	ENSG00000234617.2	SNRK-AS1	-0.97	9.80E-03	4.07E-02
4789	ENSG00000212694.8	LINC01089	-0.47	9.82E-03	4.07E-02
4790	ENSG00000236882.7	LINC01554	-1.07	9.82E-03	4.07E-02
4791	ENSG00000272674.3	PCDHB16	-0.61	9.83E-03	4.07E-02
4792	ENSG00000011347.9	SYT7	-1.08	9.83E-03	4.07E-02
4793	ENSG00000270629.5	NBPF14	-0.32	9.84E-03	4.07E-02
4794	ENSG00000124243.17	BCAS4	-0.70	9.85E-03	4.08E-02
4795	ENSG00000116783.14	TNNI3K	-2.27	9.88E-03	4.09E-02
4796	ENSG00000007866.20	TEAD3	-0.33	9.90E-03	4.10E-02
4797	ENSG00000235706.7	DICER1-AS1	0.76	9.94E-03	4.11E-02

4798	ENSG00000266877.1	AC007923.4	-2.03	9.94E-03	4.11E-02
4799	ENSG00000261707.1	AC092134.1	1.02	9.95E-03	4.12E-02
4800	ENSG00000029363.16	BCLAF1	-0.23	9.96E-03	4.12E-02
4801	ENSG00000153147.5	SMARCA5	-0.26	9.96E-03	4.12E-02
4802	ENSG00000065970.8	FOXJ2	-0.31	9.97E-03	4.12E-02
4803	ENSG00000137959.15	IFI44L	-0.98	9.98E-03	4.12E-02
4804	ENSG00000261379.1	AC010735.1	-0.92	9.99E-03	4.13E-02
4805	ENSG00000102218.5	RP2	-0.29	1.00E-02	4.13E-02
4806	ENSG00000105948.13	TTC26	-0.65	1.00E-02	4.13E-02
4807	ENSG00000145934.16	TENM2	-0.69	1.00E-02	4.15E-02
4808	ENSG00000198824.6	CHAMP1	-0.32	1.01E-02	4.15E-02
4809	ENSG00000257923.10	CUX1	-0.26	1.01E-02	4.15E-02
4810	ENSG00000224525.2	AL591686.1	0.87	1.01E-02	4.16E-02
4811	ENSG00000168517.10	HEXIM2	0.68	1.01E-02	4.17E-02
4812	ENSG00000149480.6	MTA2	-0.27	1.01E-02	4.18E-02
4813	ENSG00000177169.9	ULK1	-0.41	1.01E-02	4.18E-02
4814	ENSG00000241935.8	HOGA1	-0.87	1.01E-02	4.18E-02
4815	ENSG00000158234.12	FAIM	0.44	1.01E-02	4.18E-02
4816	ENSG00000189152.10	GRAPL	2.10	1.02E-02	4.19E-02
4817	ENSG00000124749.16	COL21A1	-0.52	1.02E-02	4.19E-02
4818	ENSG00000159176.13	CSRP1	-0.34	1.02E-02	4.19E-02
4819	ENSG00000057019.15	DCBLD2	-0.36	1.02E-02	4.20E-02
4820	ENSG00000154305.16	MIA3	0.30	1.02E-02	4.20E-02
4821	ENSG00000114686.8	MRPL3	0.27	1.02E-02	4.20E-02
4822	ENSG00000128607.13	KLHDC10	0.24	1.02E-02	4.20E-02
4823	ENSG00000005893.15	LAMP2	-0.27	1.02E-02	4.20E-02
4824	ENSG00000158042.8	MRPL17	0.32	1.03E-02	4.23E-02
4825	ENSG00000087008.15	ACOX3	0.42	1.03E-02	4.23E-02
4826	ENSG00000183979.7	NPB	-2.28	1.03E-02	4.23E-02
4827	ENSG00000228570.7	NUTM2E	0.99	1.03E-02	4.24E-02
4828	ENSG00000196312.13	MFSD14C	-0.31	1.03E-02	4.24E-02
4829	ENSG00000182010.10	RTKN2	-0.49	1.03E-02	4.25E-02
4830	ENSG00000138035.14	PNPT1	0.33	1.03E-02	4.25E-02
4831	ENSG00000100902.10	PSMA6	0.26	1.04E-02	4.26E-02
4832	ENSG00000173960.13	UBXN2A	0.29	1.04E-02	4.26E-02
4833	ENSG00000134901.12	KDELC1	-0.41	1.04E-02	4.26E-02
4834	ENSG00000112655.15	PTK7	-0.41	1.04E-02	4.26E-02
4835	ENSG00000113621.14	TXNDC15	-0.27	1.04E-02	4.27E-02
4836	ENSG00000077458.12	FAM76B	-0.36	1.04E-02	4.27E-02
4837	ENSG00000132970.12	WASF3	0.26	1.04E-02	4.27E-02
4838	ENSG00000114331.13	ACAP2	-0.23	1.04E-02	4.28E-02
4839	ENSG00000272505.1	AC104964.3	0.69	1.04E-02	4.28E-02
4840	ENSG00000089280.18	FUS	-0.29	1.05E-02	4.29E-02
4841	ENSG00000182261.3	NLRP10	-1.27	1.05E-02	4.29E-02
4842	ENSG00000137409.19	MTCH1	-0.28	1.05E-02	4.30E-02
4843	ENSG00000134690.10	CDCA8	-1.17	1.05E-02	4.30E-02
4844	ENSG00000128908.16	INO80	-0.28	1.05E-02	4.30E-02
4845	ENSG00000137819.13	PAQR5	-0.50	1.06E-02	4.33E-02
4846	ENSG00000163449.10	TMEM169	-0.45	1.06E-02	4.34E-02
4847	ENSG00000128590.4	DNAJB9	0.28	1.06E-02	4.34E-02

4848	ENSG00000214402.6	LCNL1	-1.49	1.06E-02	4.34E-02
4849	ENSG00000284624.1	AC092902.5	0.60	1.06E-02	4.35E-02
4850	ENSG00000197044.10	ZNF441	-0.40	1.06E-02	4.35E-02
4851	ENSG00000171497.4	PPID	0.33	1.07E-02	4.36E-02
4852	ENSG00000246250.2	AC087521.2	0.63	1.07E-02	4.37E-02
4853	ENSG00000186088.15	GSAP	0.39	1.07E-02	4.37E-02
4854	ENSG00000140553.17	UNC45A	-0.31	1.07E-02	4.38E-02
4855	ENSG00000104812.14	GYS1	-0.24	1.07E-02	4.38E-02
4856	ENSG00000092201.9	SUPT16H	-0.25	1.07E-02	4.38E-02
4857	ENSG00000232633.4	AC079465.1	-2.03	1.07E-02	4.38E-02
4858	ENSG00000253125.1	AC055854.1	0.97	1.07E-02	4.38E-02
4859	ENSG00000166987.14	MBD6	-0.39	1.07E-02	4.38E-02
4860	ENSG00000177697.18	CD151	-0.32	1.07E-02	4.39E-02
4861	ENSG00000188906.15	LRRK2	-0.39	1.07E-02	4.39E-02
4862	ENSG00000134996.11	OSTF1	0.40	1.08E-02	4.40E-02
4863	ENSG00000172339.9	ALG14	0.32	1.08E-02	4.40E-02
4864	ENSG00000072415.8	MPP5	-0.28	1.08E-02	4.41E-02
4865	ENSG00000183048.11	SLC25A10	-0.42	1.08E-02	4.41E-02
4866	ENSG00000229759.1	MRPS18AP1	1.40	1.08E-02	4.42E-02
4867	ENSG00000163170.11	BOLA3	0.40	1.08E-02	4.43E-02
4868	ENSG00000130958.12	SLC35D2	0.49	1.09E-02	4.43E-02
4869	ENSG00000153558.15	FBXL2	-0.50	1.09E-02	4.43E-02
4870	ENSG00000160360.12	GPSM1	-0.52	1.09E-02	4.44E-02
4871	ENSG00000141759.14	TXNL4A	0.27	1.09E-02	4.44E-02
4872	ENSG00000119402.16	FBXW2	-0.23	1.09E-02	4.44E-02
4873	ENSG00000183722.8	LHFPL6	-0.30	1.09E-02	4.45E-02
4874	ENSG00000167972.13	ABCA3	0.40	1.09E-02	4.45E-02
4875	ENSG00000187189.10	TSPYL4	-0.28	1.09E-02	4.45E-02
4876	ENSG00000237356.6	AL365295.1	1.26	1.10E-02	4.47E-02
4877	ENSG00000254901.7	BORCS8	0.38	1.10E-02	4.47E-02
4878	ENSG00000135390.18	ATP5MC2	0.27	1.10E-02	4.47E-02
4879	ENSG00000141378.14	PTRH2	0.32	1.10E-02	4.49E-02
4880	ENSG00000198680.4	TUSC1	0.43	1.10E-02	4.49E-02
4881	ENSG00000175387.15	SMAD2	0.23	1.10E-02	4.49E-02
4882	ENSG00000175899.14	A2M	-0.47	1.10E-02	4.49E-02
4883	ENSG00000198515.13	CNGA1	2.41	1.11E-02	4.49E-02
4884	ENSG00000118473.21	SGIP1	-0.69	1.11E-02	4.50E-02
4885	ENSG00000116906.12	GNPAT	0.24	1.11E-02	4.50E-02
4886	ENSG00000224940.8	PRRT4	-0.82	1.11E-02	4.50E-02
4887	ENSG00000184916.8	JAG2	0.59	1.11E-02	4.50E-02
4888	ENSG00000204261.8	PSMB8-AS1	1.00	1.11E-02	4.50E-02
4889	ENSG00000278934.1	AC117489.1	-0.73	1.11E-02	4.51E-02
4890	ENSG00000132849.20	PATJ	-0.49	1.11E-02	4.51E-02
4891	ENSG00000143365.17	RORC	-0.97	1.11E-02	4.52E-02
4892	ENSG00000244256.3	RN7SL130P	1.77	1.12E-02	4.53E-02
4893	ENSG00000132768.13	DPH2	0.41	1.12E-02	4.55E-02
4894	ENSG00000215190.9	LINC00680	-0.44	1.13E-02	4.56E-02
4895	ENSG00000131459.12	GFPT2	-0.65	1.13E-02	4.56E-02
4896	ENSG00000170677.5	SOCS6	-0.30	1.13E-02	4.57E-02
4897	ENSG00000137776.16	SLTM	0.28	1.13E-02	4.57E-02

4898	ENSG00000134825.15	TMEM258	-0.34	1.13E-02	4.58E-02
4899	ENSG00000255029.1	AC110058.1	1.04	1.13E-02	4.58E-02
4900	ENSG00000174891.12	RSRC1	-0.36	1.13E-02	4.58E-02
4901	ENSG00000059122.16	FLYWCH1	-0.38	1.13E-02	4.58E-02
4902	ENSG00000131233.9	GJA9	2.54	1.13E-02	4.59E-02
4903	ENSG00000198901.13	PRC1	-1.05	1.13E-02	4.59E-02
4904	ENSG00000116883.8	AL591845.1	-0.86	1.14E-02	4.60E-02
4905	ENSG00000167332.8	OR51E2	1.23	1.14E-02	4.61E-02
4906	ENSG00000198938.2	MT-CO3	0.23	1.14E-02	4.61E-02
4907	ENSG00000170382.11	LRRN2	0.83	1.14E-02	4.61E-02
4908	ENSG00000268350.7	FAM156A	-0.43	1.14E-02	4.61E-02
4909	ENSG00000100294.12	MCAT	0.40	1.14E-02	4.61E-02
4910	ENSG00000141522.11	ARHGDIA	-0.34	1.14E-02	4.62E-02
4911	ENSG00000083896.12	YTHDC1	-0.26	1.14E-02	4.62E-02
4912	ENSG00000088451.10	TGDS	0.40	1.14E-02	4.62E-02
4913	ENSG00000091656.16	ZFHX4	-0.27	1.15E-02	4.63E-02
4914	ENSG00000162063.12	CCNF	0.56	1.15E-02	4.64E-02
4915	ENSG00000118263.14	KLF7	-0.28	1.15E-02	4.64E-02
4916	ENSG00000109762.15	SNX25	-0.36	1.15E-02	4.64E-02
4917	ENSG00000138646.8	HERC5	0.89	1.15E-02	4.64E-02
4918	ENSG00000156469.8	MTERF3	0.34	1.15E-02	4.65E-02
4919	ENSG00000123472.12	ATPAF1	0.27	1.15E-02	4.65E-02
4920	ENSG00000198000.11	NOL8	-0.37	1.15E-02	4.65E-02
4921	ENSG00000137843.11	PAK6	1.85	1.15E-02	4.66E-02
4922	ENSG00000106609.16	TMEM248	-0.24	1.16E-02	4.66E-02
4923	ENSG00000165929.12	TC2N	-1.08	1.16E-02	4.66E-02
4924	ENSG00000111640.14	GAPDH	-0.22	1.16E-02	4.68E-02
4925	ENSG00000235893.5	BX284632.1	1.76	1.16E-02	4.68E-02
4926	ENSG00000156671.13	SAMD8	0.29	1.16E-02	4.68E-02
4927	ENSG00000084652.15	TXLNA	-0.26	1.16E-02	4.68E-02
4928	ENSG00000115216.13	NRBP1	-0.24	1.16E-02	4.69E-02
4929	ENSG00000136717.14	BIN1	0.31	1.16E-02	4.69E-02
4930	ENSG00000172421.9	EFCAB3	0.57	1.16E-02	4.69E-02
4931	ENSG00000068305.17	MEF2A	0.25	1.17E-02	4.69E-02
4932	ENSG00000185619.18	PCGF3	-0.24	1.17E-02	4.69E-02
4933	ENSG00000164244.20	PRRC1	-0.28	1.17E-02	4.69E-02
4934	ENSG00000272768.1	AC004854.2	0.77	1.17E-02	4.69E-02
4935	ENSG00000226167.1	AP4B1-AS1	-1.04	1.17E-02	4.69E-02
4936	ENSG00000154920.14	EME1	-1.55	1.17E-02	4.70E-02
4937	ENSG00000188859.6	FAM78B	1.56	1.17E-02	4.71E-02
4938	ENSG00000206535.7	LNP1	0.79	1.17E-02	4.71E-02
4939	ENSG00000182240.15	BACE2	-0.57	1.17E-02	4.71E-02
4940	ENSG00000162643.12	WDR63	-0.77	1.17E-02	4.71E-02
4941	ENSG00000170340.10	B3GNT2	-0.32	1.17E-02	4.71E-02
4942	ENSG00000197442.9	MAP3K5	-0.44	1.17E-02	4.72E-02
4943	ENSG00000108830.9	RND2	-0.97	1.17E-02	4.72E-02
4944	ENSG00000220161.4	LINC02076	0.69	1.18E-02	4.72E-02
4945	ENSG00000139318.7	DUSP6	0.39	1.18E-02	4.72E-02
4946	ENSG00000006453.13	BAIAP2L1	-0.65	1.18E-02	4.72E-02
4947	ENSG00000141034.9	GID4	0.30	1.18E-02	4.73E-02

4948	ENSG00000142676.14	RPL11	0.28	1.18E-02	4.73E-02
4949	ENSG00000085760.14	MTIF2	0.28	1.18E-02	4.75E-02
4950	ENSG00000121073.14	SLC35B1	0.28	1.19E-02	4.77E-02
4951	ENSG00000129197.14	RPAIN	0.27	1.19E-02	4.77E-02
4952	ENSG00000234616.8	JRK	0.29	1.19E-02	4.77E-02
4953	ENSG00000229431.1	AL139289.1	1.01	1.19E-02	4.77E-02
4954	ENSG00000274276.4	CBSL	-1.36	1.19E-02	4.77E-02
4955	ENSG00000143341.11	HMCN1	-0.29	1.19E-02	4.77E-02
4956	ENSG00000061938.17	TNK2	0.47	1.19E-02	4.78E-02
4957	ENSG00000176946.11	THAP4	0.33	1.19E-02	4.78E-02
4958	ENSG00000228594.3	FNDC10	-0.99	1.20E-02	4.79E-02
4959	ENSG00000171469.10	ZNF561	-0.28	1.20E-02	4.80E-02
4960	ENSG00000067369.13	TP53BP1	0.25	1.20E-02	4.80E-02
4961	ENSG00000127329.15	PTPRB	-1.36	1.20E-02	4.80E-02
4962	ENSG00000036530.8	CYP46A1	-0.59	1.20E-02	4.80E-02
4963	ENSG00000136810.12	TXN	-0.38	1.20E-02	4.80E-02
4964	ENSG00000251598.1	AC096711.3	2.45	1.20E-02	4.81E-02
4965	ENSG00000116954.7	RRAGC	0.27	1.20E-02	4.81E-02
4966	ENSG00000198416.9	ZNF658B	0.51	1.20E-02	4.81E-02
4967	ENSG00000275180.1	AC048341.2	-0.86	1.20E-02	4.81E-02
4968	ENSG00000176087.14	SLC35A4	0.26	1.20E-02	4.81E-02
4969	ENSG00000169826.7	CSGALNACT2	-0.38	1.21E-02	4.82E-02
4970	ENSG00000164105.3	SAP30	-0.55	1.21E-02	4.82E-02
4971	ENSG00000151532.13	VTI1A	0.28	1.21E-02	4.82E-02
4972	ENSG00000167618.9	LAIR2	1.04	1.21E-02	4.83E-02
4973	ENSG00000274315.1	AC009318.3	1.46	1.21E-02	4.83E-02
4974	ENSG00000164220.6	F2RL2	-0.46	1.21E-02	4.84E-02
4975	ENSG00000234494.7	SP2-AS1	0.86	1.22E-02	4.86E-02
4976	ENSG00000165629.19	ATP5F1C	0.25	1.22E-02	4.86E-02
4977	ENSG00000181610.12	MRPS23	0.30	1.22E-02	4.86E-02
4978	ENSG00000110881.11	ASIC1	-0.40	1.22E-02	4.87E-02
4979	ENSG00000265458.1	AC132938.3	0.88	1.22E-02	4.88E-02
4980	ENSG00000226711.6	FAM66C	-0.74	1.23E-02	4.88E-02
4981	ENSG00000221995.5	TIAF1	-0.91	1.23E-02	4.88E-02
4982	ENSG00000184903.9	IMMP2L	0.48	1.23E-02	4.91E-02
4983	ENSG00000124641.15	MED20	-0.30	1.23E-02	4.91E-02
4984	ENSG00000144468.16	RHBDD1	-0.24	1.24E-02	4.93E-02
4985	ENSG00000168140.4	VASN	-0.46	1.24E-02	4.93E-02
4986	ENSG00000115318.11	LOXL3	-0.46	1.24E-02	4.93E-02
4987	ENSG00000151835.15	SACS	-0.24	1.24E-02	4.93E-02
4988	ENSG00000158560.14	DYNC1I1	-0.61	1.24E-02	4.94E-02
4989	ENSG00000197798.8	FAM118B	0.34	1.24E-02	4.94E-02
4990	ENSG00000152520.13	PAN3	-0.29	1.24E-02	4.94E-02
4991	ENSG00000082515.17	MRPL22	0.31	1.25E-02	4.97E-02
4992	ENSG00000283088.1	AC010487.3	0.85	1.25E-02	4.97E-02
4993	ENSG00000248866.1	USP46-AS1	0.59	1.25E-02	4.97E-02
4994	ENSG00000064607.16	SUGP2	0.22	1.25E-02	4.98E-02
4995	ENSG00000001461.16	NIPAL3	-0.30	1.25E-02	4.98E-02
4996	ENSG00000133818.13	RRAS2	-0.35	1.25E-02	4.98E-02
4997	ENSG00000168273.7	SMIM4	0.46	1.26E-02	4.99E-02

4998	ENSG00000117122.13	MFAP2	-0.36	1.26E-02	4.99E-02
4999	ENSG00000135801.9	TAF5L	0.32	1.26E-02	4.99E-02
5000	ENSG00000130779.20	CLIP1	-0.21	1.26E-02	4.99E-02
5001	ENSG00000250266.1	LINC01612	1.67	1.26E-02	4.99E-02
5002	ENSG00000126261.12	UBA2	0.27	1.26E-02	4.99E-02
5003	ENSG00000170836.11	PPM1D	-0.32	1.26E-02	4.99E-02
5004	ENSG00000176532.3	PRR15	-0.80	1.26E-02	4.99E-02

Supplementary Table 3. List of genes differentially expressed between pure murine brown and white adipocytes identified using two-tailed DESeq2 analysis (n=5; FDR<0.05).

number	ID	Gene symbol	log2FoldChange	P value	FDR adjusted P
1	ENSMUSG00000034855	Cxcl10	-4.43	3.60E-25	2.21E-21
2	ENSMUSG00000025059	Gk	4.09	4.31E-25	2.21E-21
3	ENSMUSG00000023064	Sncg	-4.89	9.65E-25	3.29E-21
4	ENSMUSG00000031710	Ucp1	6.21	1.99E-24	5.10E-21
5	ENSMUSG00000038754	Elovl3	4.99	3.64E-24	6.54E-21
6	ENSMUSG00000050860	Phospho1	4.70	4.44E-24	6.54E-21
7	ENSMUSG00000053113	Socs3	-4.50	4.48E-24	6.54E-21
8	ENSMUSG00000037868	Egr2	-4.75	7.49E-24	9.58E-21
9	ENSMUSG00000044786	Zfp36	-3.94	1.04E-23	1.18E-20
10	ENSMUSG00000027359	Slc27a2	5.15	1.37E-23	1.37E-20
11	ENSMUSG00000050777	Tmem37	4.69	1.60E-23	1.37E-20
12	ENSMUSG00000024526	Cidea	4.77	1.61E-23	1.37E-20
13	ENSMUSG00000058022	Adtrp	4.06	1.95E-23	1.53E-20
14	ENSMUSG00000023913	Pla2g7	4.69	4.49E-23	3.28E-20
15	ENSMUSG00000037686	Aspg	4.19	5.00E-23	3.41E-20
16	ENSMUSG00000097458	Gm26697	4.38	6.51E-23	4.16E-20
17	ENSMUSG00000018899	Irf1	-3.82	7.27E-23	4.28E-20
18	ENSMUSG00000024981	Acsl5	3.80	7.53E-23	4.28E-20
19	ENSMUSG00000033610	Pank1	4.02	1.17E-22	6.28E-20
20	ENSMUSG00000026043	Col3a1	-4.02	1.52E-22	7.75E-20
21	ENSMUSG00000038418	Egr1	-3.55	1.85E-22	9.03E-20
22	ENSMUSG00000038521	C1s1	-3.77	2.01E-22	9.32E-20
23	ENSMUSG00000018381	Abi3	3.79	2.85E-22	1.27E-19
24	ENSMUSG00000036136	Fam110c	-3.91	3.71E-22	1.48E-19
25	ENSMUSG00000022617	Chkb	3.68	3.76E-22	1.48E-19
26	ENSMUSG00000031328	Flna	-3.72	3.77E-22	1.48E-19
27	ENSMUSG00000100291	2310069B03Rik	4.68	4.13E-22	1.56E-19
28	ENSMUSG00000074218	Cox7a1	6.05	4.53E-22	1.66E-19
29	ENSMUSG00000064354	mt-Co2	3.56	8.74E-22	3.08E-19
30	ENSMUSG00000064358	mt-Co3	4.21	9.12E-22	3.11E-19
31	ENSMUSG00000022893	Adamts1	-4.30	9.57E-22	3.16E-19
32	ENSMUSG00000064357	mt-Atp6	3.79	1.08E-21	3.42E-19
33	ENSMUSG00000029380	Cxcl1	-4.27	1.10E-21	3.42E-19
34	ENSMUSG00000024525	Impa2	4.05	1.17E-21	3.52E-19
35	ENSMUSG00000028773	Fabp3	5.97	1.29E-21	3.74E-19
36	ENSMUSG00000075595	Zfp652	3.48	1.33E-21	3.74E-19
37	ENSMUSG00000021250	Fos	-3.41	1.35E-21	3.74E-19
38	ENSMUSG00000071637	Cebpd	-3.52	1.83E-21	4.92E-19
39	ENSMUSG00000029368	Alb	-4.30	2.03E-21	5.31E-19
40	ENSMUSG00000078937	Cpt1b	3.98	2.08E-21	5.33E-19
41	ENSMUSG00000060459	Kng2	4.77	2.93E-21	7.30E-19
42	ENSMUSG00000110439	Gm21320	-4.49	3.04E-21	7.41E-19
43	ENSMUSG00000035356	Nfkbiz	-3.63	3.31E-21	7.88E-19
44	ENSMUSG00000022346	Myc	-4.72	3.57E-21	8.20E-19
45	ENSMUSG00000040152	Thbs1	-4.09	3.61E-21	8.20E-19
46	ENSMUSG00000026819	Slc25a25	-3.19	3.99E-21	8.88E-19
47	ENSMUSG00000030246	Ldhb	3.66	4.44E-21	9.67E-19

48	ENSMUSG00000027832	Ptx3	-4.44	5.38E-21	1.15E-18
49	ENSMUSG00000027068	Dhrs9	3.75	5.91E-21	1.23E-18
50	ENSMUSG00000078680	Mup10	-4.43	8.02E-21	1.64E-18
51	ENSMUSG00000020423	Btg2	-3.45	8.53E-21	1.70E-18
52	ENSMUSG00000037353	Letmd1	3.35	8.65E-21	1.70E-18
53	ENSMUSG00000023224	Serping1	-4.13	9.12E-21	1.76E-18
54	ENSMUSG00000053560	Ier2	-2.97	1.09E-20	2.06E-18
55	ENSMUSG00000074063	Osgin1	-3.37	1.36E-20	2.52E-18
56	ENSMUSG00000038526	Car14	4.13	1.56E-20	2.84E-18
57	ENSMUSG00000025488	Cox8b	4.58	2.25E-20	4.03E-18
58	ENSMUSG00000030108	Slc6a13	-2.88	2.33E-20	4.11E-18
59	ENSMUSG00000056708	Ier5	-3.68	2.38E-20	4.12E-18
60	ENSMUSG00000045312	Lhfpl2	-3.77	2.48E-20	4.23E-18
61	ENSMUSG00000064345	mt-Nd2	3.65	2.76E-20	4.63E-18
62	ENSMUSG00000007872	Id3	-3.03	3.24E-20	5.34E-18
63	ENSMUSG00000028195	Cyr61	-3.03	3.34E-20	5.43E-18
64	ENSMUSG00000034853	Acot11	3.78	4.11E-20	6.56E-18
65	ENSMUSG00000072949	Acot1	3.37	4.51E-20	7.09E-18
66	ENSMUSG00000051596	Otop1	2.83	4.77E-20	7.32E-18
67	ENSMUSG00000040339	Fam102b	-3.07	4.79E-20	7.32E-18
68	ENSMUSG00000032501	Trib1	-3.17	4.99E-20	7.50E-18
69	ENSMUSG00000011305	Plin5	2.77	5.65E-20	8.37E-18
70	ENSMUSG00000064367	mt-Nd5	3.08	6.11E-20	8.92E-18
71	ENSMUSG00000038543	BC028528	4.50	6.52E-20	9.40E-18
72	ENSMUSG00000063882	Uqcrh	3.62	6.75E-20	9.46E-18
73	ENSMUSG00000078713	Tomm5	3.44	6.76E-20	9.46E-18
74	ENSMUSG00000045287	Rtn4rl1	-3.43	7.44E-20	1.03E-17
75	ENSMUSG00000064368	mt-Nd6	3.38	7.64E-20	1.04E-17
76	ENSMUSG00000032531	Amotl2	-2.72	8.14E-20	1.10E-17
77	ENSMUSG00000049265	Kcnk3	3.13	8.32E-20	1.11E-17
78	ENSMUSG00000038522	Mfsd4b1	2.97	8.60E-20	1.13E-17
79	ENSMUSG00000078486	Perm1	3.76	9.10E-20	1.18E-17
80	ENSMUSG00000034871	Fam151a	3.65	9.27E-20	1.19E-17
81	ENSMUSG00000000753	Serpinf1	-2.96	9.49E-20	1.20E-17
82	ENSMUSG00000063694	Cycs	3.13	1.11E-19	1.38E-17
83	ENSMUSG00000032381	Fam96a	2.93	1.15E-19	1.41E-17
84	ENSMUSG00000001119	Col6a1	-3.84	1.25E-19	1.52E-17
85	ENSMUSG00000052837	Junb	-3.22	1.35E-19	1.61E-17
86	ENSMUSG00000001435	Col18a1	-2.94	1.37E-19	1.61E-17
87	ENSMUSG00000061474	Mrps36	3.52	1.37E-19	1.61E-17
88	ENSMUSG00000053214	Gm9899	2.82	1.42E-19	1.66E-17
89	ENSMUSG00000006784	Ttc25	3.45	1.44E-19	1.66E-17
90	ENSMUSG00000028655	Mfsd2a	3.89	1.48E-19	1.68E-17
91	ENSMUSG00000064363	mt-Nd4	3.44	1.67E-19	1.88E-17
92	ENSMUSG00000027673	Ndufb5	3.04	1.72E-19	1.91E-17
93	ENSMUSG00000096887	Gm20594	3.78	1.83E-19	2.01E-17
94	ENSMUSG00000045160	Bola3	2.89	1.87E-19	2.03E-17
95	ENSMUSG00000090307	1700071M16Rik	3.36	1.89E-19	2.03E-17
96	ENSMUSG00000000901	Mmp11	3.12	2.04E-19	2.17E-17
97	ENSMUSG00000045316	Fahd1	2.67	2.15E-19	2.27E-17

98	ENSMUSG00000071014	Ndufb6	3.19	2.23E-19	2.33E-17
99	ENSMUSG00000026574	Dpt	-3.32	2.28E-19	2.35E-17
100	ENSMUSG00000091471	Gm20538	3.25	2.35E-19	2.40E-17
101	ENSMUSG00000056427	Slit3	-3.37	2.40E-19	2.43E-17
102	ENSMUSG00000086324	Gm15564	3.70	2.57E-19	2.55E-17
103	ENSMUSG00000064341	mt-Nd1	3.41	2.57E-19	2.55E-17
104	ENSMUSG00000021456	Fbp2	4.01	2.71E-19	2.67E-17
105	ENSMUSG00000049422	Chchd10	3.16	2.82E-19	2.75E-17
106	ENSMUSG00000037060	Prkcdbp	-3.62	2.87E-19	2.76E-17
107	ENSMUSG00000031231	Cox7b	3.36	2.95E-19	2.82E-17
108	ENSMUSG00000025204	Ndufb8	3.21	3.11E-19	2.94E-17
109	ENSMUSG00000059248	Sep-09	2.90	3.30E-19	3.10E-17
110	ENSMUSG00000022450	Ndufa6	3.17	3.64E-19	3.39E-17
111	ENSMUSG00000060961	Slc4a4	3.15	3.89E-19	3.59E-17
112	ENSMUSG00000028407	Toporsos	3.03	3.96E-19	3.62E-17
113	ENSMUSG00000079508	Apoo	2.82	4.12E-19	3.73E-17
114	ENSMUSG00000002289	Angptl4	-2.51	4.23E-19	3.79E-17
115	ENSMUSG00000032602	Slc25a20	2.62	4.34E-19	3.85E-17
116	ENSMUSG00000019689	Fmc1	3.71	4.36E-19	3.85E-17
117	ENSMUSG00000024099	Ndufv2	2.90	4.54E-19	3.96E-17
118	ENSMUSG00000067786	Nnat	-5.47	4.92E-19	4.25E-17
119	ENSMUSG00000026042	Col5a2	-3.27	4.94E-19	4.25E-17
120	ENSMUSG00000021520	Uqcrb	3.01	5.07E-19	4.30E-17
121	ENSMUSG00000023089	Ndufa5	3.20	5.09E-19	4.30E-17
122	ENSMUSG00000003545	Fosb	-3.91	5.37E-19	4.50E-17
123	ENSMUSG00000053398	Phgdh	-3.89	5.59E-19	4.65E-17
124	ENSMUSG00000039611	Tmem246	3.34	5.85E-19	4.83E-17
125	ENSMUSG00000079343	C1s2	-3.02	6.21E-19	5.08E-17
126	ENSMUSG00000046516	Cox17	3.54	6.36E-19	5.16E-17
127	ENSMUSG00000022332	Khdrbs3	2.93	6.54E-19	5.26E-17
128	ENSMUSG00000064370	mt-Cytb	3.09	6.69E-19	5.33E-17
129	ENSMUSG00000022820	Ndufb4	3.14	6.76E-19	5.33E-17
130	ENSMUSG00000108573	Gm44986	2.92	6.77E-19	5.33E-17
131	ENSMUSG00000091191	Gm17334	-3.22	6.99E-19	5.46E-17
132	ENSMUSG00000050856	Atp5k	3.36	7.17E-19	5.54E-17
133	ENSMUSG00000095464	Gm21987	3.51	7.20E-19	5.54E-17
134	ENSMUSG00000032330	Cox7a2	3.27	7.28E-19	5.56E-17
135	ENSMUSG00000034566	Atp5h	3.10	7.66E-19	5.78E-17
136	ENSMUSG00000020241	Col6a2	-3.97	7.69E-19	5.78E-17
137	ENSMUSG00000028681	Ptch2	-3.07	7.81E-19	5.83E-17
138	ENSMUSG00000074826	Gm10767	2.77	8.08E-19	5.95E-17
139	ENSMUSG00000057913	Gm10032	3.79	8.09E-19	5.95E-17
140	ENSMUSG00000086582	Gm16272	3.20	8.18E-19	5.95E-17
141	ENSMUSG00000026478	Lamc1	-2.73	8.21E-19	5.95E-17
142	ENSMUSG00000034880	Mrpl34	2.80	9.08E-19	6.51E-17
143	ENSMUSG00000036751	Cox6b1	3.28	9.10E-19	6.51E-17
144	ENSMUSG00000045102	Poln	2.90	9.61E-19	6.82E-17
145	ENSMUSG00000059534	Uqcr10	3.40	9.67E-19	6.82E-17
146	ENSMUSG00000028766	Alpl	3.76	9.95E-19	6.97E-17
147	ENSMUSG00000113902	AC121965.1	3.13	1.02E-18	7.07E-17

148	ENSMUSG00000036880	Acaa2	2.85	1.04E-18	7.14E-17
149	ENSMUSG00000035373	Ccl7	-3.78	1.04E-18	7.14E-17
150	ENSMUSG00000033429	Mcee	3.21	1.11E-18	7.60E-17
151	ENSMUSG00000048126	Col6a3	-3.25	1.29E-18	8.77E-17
152	ENSMUSG00000105361	AY036118	4.30	1.31E-18	8.79E-17
153	ENSMUSG00000030869	Ndufab1	2.82	1.34E-18	8.96E-17
154	ENSMUSG00000044894	Uqcrq	3.56	1.35E-18	8.96E-17
155	ENSMUSG00000025092	Hspa12a	-2.78	1.36E-18	8.96E-17
156	ENSMUSG00000021606	Ndufs6	3.23	1.38E-18	8.96E-17
157	ENSMUSG00000035674	Ndufa3	3.40	1.38E-18	8.96E-17
158	ENSMUSG00000016252	Atp5e	3.95	1.38E-18	8.96E-17
159	ENSMUSG00000071528	Usmg5	3.39	1.43E-18	9.21E-17
160	ENSMUSG00000040740	Slc25a34	3.04	1.46E-18	9.31E-17
161	ENSMUSG00000020963	Tshr	-2.94	1.55E-18	9.83E-17
162	ENSMUSG00000037531	Mrpl47	2.62	1.57E-18	9.90E-17
163	ENSMUSG00000002416	Ndufb2	3.27	1.58E-18	9.90E-17
164	ENSMUSG00000035202	Lars2	3.68	1.60E-18	9.95E-17
165	ENSMUSG00000020386	Sar1b	2.47	1.63E-18	1.01E-16
166	ENSMUSG00000042793	Lgr6	3.95	1.66E-18	1.02E-16
167	ENSMUSG00000052738	Suclg1	2.94	1.68E-18	1.03E-16
168	ENSMUSG00000005397	Nid1	-3.92	1.68E-18	1.03E-16
169	ENSMUSG00000074743	Thbd	-2.64	1.82E-18	1.10E-16
170	ENSMUSG00000086868	Gm15883	-4.71	1.89E-18	1.14E-16
171	ENSMUSG00000080727	C920021L13Rik	4.16	1.92E-18	1.15E-16
172	ENSMUSG00000029810	Tmem176b	-3.70	2.07E-18	1.23E-16
173	ENSMUSG00000050157	Gm867	2.94	2.10E-18	1.24E-16
174	ENSMUSG00000023885	Thbs2	-3.37	2.12E-18	1.25E-16
175	ENSMUSG00000023175	Bsg	2.72	2.16E-18	1.26E-16
176	ENSMUSG00000033938	Ndufb7	3.02	2.17E-18	1.26E-16
177	ENSMUSG00000098178	Gm42418	4.19	2.23E-18	1.29E-16
178	ENSMUSG00000032579	Hemk1	2.48	2.32E-18	1.33E-16
179	ENSMUSG00000107071	Gm42420	3.20	2.34E-18	1.34E-16
180	ENSMUSG00000072235	Tuba1a	-2.63	2.54E-18	1.45E-16
181	ENSMUSG00000027875	Hmgcs2	-3.31	2.56E-18	1.45E-16
182	ENSMUSG00000087190	D430001F17Rik	2.72	2.58E-18	1.45E-16
183	ENSMUSG00000031059	Ndufb11	2.84	2.69E-18	1.50E-16
184	ENSMUSG00000052974	Cyp2f2	-5.05	2.81E-18	1.56E-16
185	ENSMUSG00000026895	Ndufa8	2.73	3.01E-18	1.66E-16
186	ENSMUSG00000039016	Timm8b	2.97	3.03E-18	1.67E-16
187	ENSMUSG00000109735	Gm45667	-3.26	3.17E-18	1.73E-16
188	ENSMUSG00000000171	Sdhd	2.49	3.27E-18	1.78E-16
189	ENSMUSG00000056091	St3gal5	3.87	3.32E-18	1.80E-16
190	ENSMUSG00000038690	Atp5j2	3.25	3.39E-18	1.82E-16
191	ENSMUSG00000061518	Cox5b	3.02	3.40E-18	1.82E-16
192	ENSMUSG00000021764	Ndufs4	2.89	3.70E-18	1.97E-16
193	ENSMUSG00000000078	Klf6	-3.02	3.84E-18	2.03E-16
194	ENSMUSG00000037904	Ankrd9	3.10	3.95E-18	2.08E-16
195	ENSMUSG00000030790	Adm	-3.15	4.00E-18	2.10E-16
196	ENSMUSG00000002379	Ndufa11	3.15	4.06E-18	2.12E-16
197	ENSMUSG00000073616	Cops9	3.11	4.24E-18	2.20E-16

198	ENSMUSG00000022354	Ndufb9	2.87	4.28E-18	2.21E-16
199	ENSMUSG00000050608	Minos1	3.16	4.50E-18	2.29E-16
200	ENSMUSG00000022587	Ly6e	-2.51	4.52E-18	2.29E-16
201	ENSMUSG00000022956	Atp5o	2.80	4.53E-18	2.29E-16
202	ENSMUSG00000059734	Ndufs8	2.98	4.53E-18	2.29E-16
203	ENSMUSG00000064351	mt-Co1	2.90	4.67E-18	2.35E-16
204	ENSMUSG00000024371	C2	-3.58	4.81E-18	2.41E-16
205	ENSMUSG00000020022	Ndufa12	2.72	5.25E-18	2.62E-16
206	ENSMUSG00000073676	Hspe1	2.91	5.43E-18	2.70E-16
207	ENSMUSG00000021978	Extl3	-2.44	5.52E-18	2.73E-16
208	ENSMUSG00000025348	Itga7	-2.97	5.57E-18	2.73E-16
209	ENSMUSG00000037152	Ndufc1	3.05	5.57E-18	2.73E-16
210	ENSMUSG00000024659	Anxa1	-2.76	5.77E-18	2.81E-16
211	ENSMUSG00000016257	Prelid3b	2.23	5.79E-18	2.81E-16
212	ENSMUSG00000026748	Plxdc2	-3.38	6.06E-18	2.92E-16
213	ENSMUSG00000022223	Sdr39u1	2.43	6.60E-18	3.17E-16
214	ENSMUSG00000055172	C1ra	-3.72	6.79E-18	3.25E-16
215	ENSMUSG00000062981	Mrpl42	2.43	7.12E-18	3.39E-16
216	ENSMUSG00000031502	Col4a1	-2.46	7.34E-18	3.46E-16
217	ENSMUSG00000029632	Ndufa4	3.08	7.35E-18	3.46E-16
218	ENSMUSG00000006315	Tmem147	2.36	7.38E-18	3.46E-16
219	ENSMUSG00000021361	Tmem14c	2.71	7.69E-18	3.59E-16
220	ENSMUSG00000036298	Slc2a13	-3.31	7.81E-18	3.63E-16
221	ENSMUSG00000017778	Cox7c	2.95	7.94E-18	3.67E-16
222	ENSMUSG00000040048	Ndufb10	2.50	8.39E-18	3.86E-16
223	ENSMUSG00000027452	Acss1	2.75	8.57E-18	3.93E-16
224	ENSMUSG00000039914	Coq10a	2.29	8.72E-18	3.98E-16
225	ENSMUSG00000040564	Apoc1	3.40	8.81E-18	3.99E-16
226	ENSMUSG00000025466	Fuom	3.05	8.82E-18	3.99E-16
227	ENSMUSG00000097445	Gm26631	2.98	9.07E-18	4.08E-16
228	ENSMUSG00000008153	Clstn3	2.35	9.09E-18	4.08E-16
229	ENSMUSG00000021290	2010107E04Rik	3.02	9.35E-18	4.18E-16
230	ENSMUSG00000026365	Cfh	-3.57	1.01E-17	4.47E-16
231	ENSMUSG00000035863	Palm	-2.59	1.03E-17	4.54E-16
232	ENSMUSG00000032135	Mcam	-3.48	1.05E-17	4.61E-16
233	ENSMUSG00000024869	Nudt8	2.49	1.09E-17	4.78E-16
234	ENSMUSG00000006717	Acot13	2.92	1.09E-17	4.78E-16
235	ENSMUSG00000110949	Nudt8	2.49	1.12E-17	4.88E-16
236	ENSMUSG00000001663	Gstt1	-2.37	1.18E-17	5.12E-16
237	ENSMUSG00000024781	Lipa	2.26	1.22E-17	5.25E-16
238	ENSMUSG00000036611	Eepd1	-3.38	1.25E-17	5.38E-16
239	ENSMUSG00000000088	Cox5a	2.93	1.30E-17	5.57E-16
240	ENSMUSG00000023034	Nr4a1	-2.66	1.41E-17	5.99E-16
241	ENSMUSG00000107689	Gm44386	3.01	1.45E-17	6.14E-16
242	ENSMUSG00000026087	Mrpl30	2.23	1.47E-17	6.19E-16
243	ENSMUSG00000029778	Adcyap1r1	-3.60	1.48E-17	6.21E-16
244	ENSMUSG00000016427	Ndufa1	2.95	1.49E-17	6.24E-16
245	ENSMUSG00000028121	Bcar3	-3.21	1.55E-17	6.45E-16
246	ENSMUSG00000087478	4930506C21Rik	2.64	1.57E-17	6.52E-16
247	ENSMUSG00000011752	Pgam1	2.27	1.59E-17	6.57E-16

248	ENSMUSG00000086938	4930481A15Rik	3.09	1.70E-17	6.99E-16
249	ENSMUSG00000025732	Fam195a	2.75	1.82E-17	7.49E-16
250	ENSMUSG00000037095	Lrg1	-2.83	1.83E-17	7.50E-16
251	ENSMUSG00000022892	App	-2.34	1.90E-17	7.72E-16
252	ENSMUSG00000113380	AC122465.1	-3.18	1.91E-17	7.73E-16
253	ENSMUSG00000015451	C4a	-5.04	1.97E-17	7.96E-16
254	ENSMUSG00000059201	Lep	-4.05	2.04E-17	8.21E-16
255	ENSMUSG00000053279	Aldh1a1	-3.74	2.08E-17	8.35E-16
256	ENSMUSG00000002346	Slc25a42	2.40	2.09E-17	8.36E-16
257	ENSMUSG00000049760	2410015M20Rik	2.69	2.18E-17	8.63E-16
258	ENSMUSG00000026568	Mpc2	2.88	2.18E-17	8.63E-16
259	ENSMUSG00000023861	Mpc1	2.63	2.19E-17	8.64E-16
260	ENSMUSG00000022894	Adamts5	-3.04	2.21E-17	8.70E-16
261	ENSMUSG00000030621	Me3	3.38	2.25E-17	8.82E-16
262	ENSMUSG00000019763	Rmnd1	2.12	2.46E-17	9.59E-16
263	ENSMUSG00000032279	Idh3a	2.24	2.49E-17	9.69E-16
264	ENSMUSG00000096449	Gm4076	2.65	2.53E-17	9.81E-16
265	ENSMUSG00000026623	Lpgat1	-2.23	2.73E-17	1.05E-15
266	ENSMUSG00000019853	Hebp2	2.54	2.86E-17	1.10E-15
267	ENSMUSG00000036427	Gpi1	2.57	2.93E-17	1.12E-15
268	ENSMUSG00000034842	Art3	-2.65	2.99E-17	1.14E-15
269	ENSMUSG00000059325	Hopx	2.99	3.04E-17	1.16E-15
270	ENSMUSG00000021868	Ppif	2.27	3.06E-17	1.16E-15
271	ENSMUSG00000004098	Col5a3	-2.70	3.31E-17	1.25E-15
272	ENSMUSG00000027076	Timm10	2.70	3.34E-17	1.26E-15
273	ENSMUSG00000060126	Tpt1	2.28	3.55E-17	1.33E-15
274	ENSMUSG00000089961	Gm16567	-3.13	3.62E-17	1.35E-15
275	ENSMUSG00000038462	Uqcrfs1	2.44	3.63E-17	1.35E-15
276	ENSMUSG00000021806	Nid2	-3.53	3.69E-17	1.37E-15
277	ENSMUSG00000024222	Fkbp5	-2.55	3.73E-17	1.37E-15
278	ENSMUSG00000000253	Gmpr	4.62	3.74E-17	1.37E-15
279	ENSMUSG00000052684	Jun	-2.32	3.90E-17	1.43E-15
280	ENSMUSG00000006715	Gmnn	2.88	3.96E-17	1.45E-15
281	ENSMUSG00000027357	Crls1	2.18	4.11E-17	1.50E-15
282	ENSMUSG00000049960	Mrps16	2.55	4.14E-17	1.50E-15
283	ENSMUSG00000020163	Uqcr11	3.02	4.28E-17	1.55E-15
284	ENSMUSG00000029335	Bmp3	-3.22	4.35E-17	1.56E-15
285	ENSMUSG00000064360	mt-Nd3	3.20	4.35E-17	1.56E-15
286	ENSMUSG00000027099	Mtx2	2.08	4.43E-17	1.58E-15
287	ENSMUSG00000038717	Atp5l	2.97	4.60E-17	1.64E-15
288	ENSMUSG00000001666	Ddt	2.64	4.70E-17	1.67E-15
289	ENSMUSG00000029096	Htra3	-2.97	4.81E-17	1.70E-15
290	ENSMUSG00000025885	Myo5b	3.25	4.87E-17	1.72E-15
291	ENSMUSG00000036199	Ndufa13	2.44	5.13E-17	1.80E-15
292	ENSMUSG00000025190	Got1	3.32	5.62E-17	1.97E-15
293	ENSMUSG00000026837	Col5a1	-2.86	5.64E-17	1.97E-15
294	ENSMUSG00000011148	Adssl1	2.85	5.89E-17	2.04E-15
295	ENSMUSG00000026208	Des	2.73	5.91E-17	2.04E-15
296	ENSMUSG00000009281	Rarres2	-2.85	5.92E-17	2.04E-15
297	ENSMUSG00000031448	Adprhl1	3.33	6.13E-17	2.11E-15

298	ENSMUSG00000020277	Pfkl	2.37	6.15E-17	2.11E-15
299	ENSMUSG00000097660	Gm26762	2.43	6.18E-17	2.11E-15
300	ENSMUSG00000031818	Cox4i1	2.79	6.30E-17	2.15E-15
301	ENSMUSG00000019806	Aig1	3.04	6.36E-17	2.16E-15
302	ENSMUSG00000029070	Mxra8	-3.39	6.37E-17	2.16E-15
303	ENSMUSG00000060152	Pop5	2.53	6.56E-17	2.21E-15
304	ENSMUSG00000020674	Pxdn	-3.34	6.76E-17	2.27E-15
305	ENSMUSG00000111082	AC061963.3	2.90	7.02E-17	2.35E-15
306	ENSMUSG00000028532	Cachd1	-3.06	7.27E-17	2.43E-15
307	ENSMUSG00000075706	Gpx4	2.53	7.74E-17	2.58E-15
308	ENSMUSG00000014301	Pam16	2.68	7.77E-17	2.58E-15
309	ENSMUSG00000041881	Ndufa7	2.53	7.79E-17	2.58E-15
310	ENSMUSG00000071847	Apcdd1	-3.76	7.87E-17	2.60E-15
311	ENSMUSG00000016256	Ctsz	2.47	8.38E-17	2.76E-15
312	ENSMUSG00000028229	Rmdn1	2.96	9.02E-17	2.96E-15
313	ENSMUSG00000023367	Tmem176a	-3.21	9.08E-17	2.97E-15
314	ENSMUSG00000024248	Cox7a2l	2.13	9.13E-17	2.97E-15
315	ENSMUSG00000014313	Cox6c	3.00	9.23E-17	3.00E-15
316	ENSMUSG00000004945	Tmem242	2.60	9.42E-17	3.05E-15
317	ENSMUSG00000001627	Ifrd1	-2.05	9.54E-17	3.08E-15
318	ENSMUSG00000002820	Atg4d	1.99	9.56E-17	3.08E-15
319	ENSMUSG00000029001	Fbxo44	2.17	1.03E-16	3.29E-15
320	ENSMUSG00000028772	Zcchc17	3.30	1.09E-16	3.49E-15
321	ENSMUSG00000023004	Tuba1b	-2.31	1.10E-16	3.49E-15
322	ENSMUSG00000062070	Pgk1	2.09	1.13E-16	3.59E-15
323	ENSMUSG00000002661	Alkbh7	3.14	1.14E-16	3.60E-15
324	ENSMUSG00000048731	Ggnbp1	2.68	1.16E-16	3.66E-15
325	ENSMUSG00000028648	Ndufs5	2.49	1.16E-16	3.66E-15
326	ENSMUSG00000087366	Junos	-2.25	1.23E-16	3.87E-15
327	ENSMUSG00000024955	Esrra	2.04	1.31E-16	4.11E-15
328	ENSMUSG00000033576	Apol6	-2.84	1.33E-16	4.16E-15
329	ENSMUSG00000021241	Isca2	2.15	1.36E-16	4.24E-15
330	ENSMUSG00000107002	0610012G03Rik	2.32	1.37E-16	4.24E-15
331	ENSMUSG00000022146	Osmr	-2.90	1.40E-16	4.32E-15
332	ENSMUSG00000022013	Dnajc15	2.47	1.44E-16	4.44E-15
333	ENSMUSG00000025486	Sirt3	2.03	1.47E-16	4.50E-15
334	ENSMUSG00000025722	Wdr73	2.21	1.53E-16	4.69E-15
335	ENSMUSG00000039959	Hip1	-2.78	1.62E-16	4.95E-15
336	ENSMUSG00000079941	Gm11273	2.90	1.66E-16	5.05E-15
337	ENSMUSG00000031781	Ciapin1	2.36	1.76E-16	5.35E-15
338	ENSMUSG00000033208	S100b	3.93	1.88E-16	5.69E-15
339	ENSMUSG00000009863	Sdhb	2.42	1.89E-16	5.69E-15
340	ENSMUSG00000033845	Mrpl15	2.32	1.90E-16	5.73E-15
341	ENSMUSG00000017144	Rnd3	-3.05	1.94E-16	5.83E-15
342	ENSMUSG00000058076	Sdhc	2.02	1.98E-16	5.93E-15
343	ENSMUSG00000056899	Immp2l	2.35	1.99E-16	5.94E-15
344	ENSMUSG00000020390	Ube2b	1.96	2.01E-16	5.99E-15
345	ENSMUSG00000025498	Irf7	-3.00	2.06E-16	6.11E-15
346	ENSMUSG00000032584	Mst1r	-2.97	2.08E-16	6.15E-15
347	ENSMUSG00000090258	Churc1	2.25	2.09E-16	6.15E-15

348	ENSMUSG00000029455	Aldh2	-2.00	2.16E-16	6.36E-15
349	ENSMUSG00000029198	Grpel1	2.05	2.22E-16	6.51E-15
350	ENSMUSG00000022415	Syngr1	2.71	2.26E-16	6.60E-15
351	ENSMUSG00000052911	Lamb2	-2.25	2.36E-16	6.88E-15
352	ENSMUSG00000023723	Mrps23	2.11	2.38E-16	6.91E-15
353	ENSMUSG00000063406	Tmed5	1.97	2.47E-16	7.17E-15
354	ENSMUSG00000092360	Gm20441	2.42	2.50E-16	7.23E-15
355	ENSMUSG00000003955	Fam162a	2.30	2.59E-16	7.45E-15
356	ENSMUSG00000024529	Lox	-2.14	2.60E-16	7.46E-15
357	ENSMUSG00000004285	Atp6v1f	2.30	2.68E-16	7.67E-15
358	ENSMUSG00000057388	Mrpl18	1.99	2.70E-16	7.72E-15
359	ENSMUSG00000022665	Ccdc80	-4.28	2.80E-16	7.97E-15
360	ENSMUSG00000014294	Ndufa2	2.70	3.08E-16	8.74E-15
361	ENSMUSG00000030208	Emp1	-3.46	3.12E-16	8.83E-15
362	ENSMUSG00000074364	Ehd2	-2.23	3.25E-16	9.17E-15
363	ENSMUSG00000098188	Sowahc	-2.45	3.26E-16	9.18E-15
364	ENSMUSG00000058927	Gm10053	2.50	3.28E-16	9.23E-15
365	ENSMUSG00000021094	Dhrs7	2.00	3.44E-16	9.63E-15
366	ENSMUSG00000040370	Etfrf1	1.99	3.59E-16	1.00E-14
367	ENSMUSG00000020153	Ndufs7	2.35	3.62E-16	1.01E-14
368	ENSMUSG00000020010	Vnn3	-2.72	3.92E-16	1.09E-14
369	ENSMUSG00000071654	Uqcc3	2.39	4.05E-16	1.12E-14
370	ENSMUSG00000090137	Uba52	2.34	4.05E-16	1.12E-14
371	ENSMUSG00000031253	Srpx2	-2.78	4.10E-16	1.13E-14
372	ENSMUSG00000111394	AC160637.1	2.35	4.15E-16	1.14E-14
373	ENSMUSG00000030037	Mrpl53	2.09	4.21E-16	1.15E-14
374	ENSMUSG00000043162	Pyurf	2.09	4.28E-16	1.17E-14
375	ENSMUSG00000004610	Etfb	2.44	4.30E-16	1.17E-14
376	ENSMUSG00000033361	Prrg3	-2.68	4.37E-16	1.19E-14
377	ENSMUSG00000107482	Gm45233	2.43	4.39E-16	1.19E-14
378	ENSMUSG00000022110	Sucla2	2.17	4.44E-16	1.20E-14
379	ENSMUSG00000025175	Fn3k	2.59	4.57E-16	1.23E-14
380	ENSMUSG00000054072	Iigp1	-2.99	4.62E-16	1.24E-14
381	ENSMUSG00000053768	Chchd3	2.00	4.98E-16	1.34E-14
382	ENSMUSG00000033295	Ptprf	-2.98	5.02E-16	1.35E-14
383	ENSMUSG00000038156	Spon1	-3.08	5.06E-16	1.35E-14
384	ENSMUSG00000021091	Serpina3n	-4.03	5.09E-16	1.36E-14
385	ENSMUSG00000036256	Igfbp7	-2.65	5.14E-16	1.37E-14
386	ENSMUSG00000038845	Phb	2.02	5.25E-16	1.39E-14
387	ENSMUSG00000005354	Txn2	1.96	5.32E-16	1.41E-14
388	ENSMUSG00000020101	Vsir	-3.02	5.36E-16	1.41E-14
389	ENSMUSG00000028998	Tomm7	2.29	5.37E-16	1.41E-14
390	ENSMUSG00000002831	Plin4	-3.01	5.56E-16	1.46E-14
391	ENSMUSG00000018821	Avpi1	1.99	5.66E-16	1.48E-14
392	ENSMUSG00000061461	Smim20	2.21	5.76E-16	1.50E-14
393	ENSMUSG00000028339	Col15a1	-2.96	5.79E-16	1.51E-14
394	ENSMUSG00000026817	Ak1	2.61	5.83E-16	1.51E-14
395	ENSMUSG00000073834	Mup11	-3.15	5.85E-16	1.52E-14
396	ENSMUSG00000027282	Mtch2	2.05	6.14E-16	1.59E-14
397	ENSMUSG00000049521	Cdc42ep1	-2.99	6.16E-16	1.59E-14

398	ENSMUSG00000038366	Lasp1	-2.49	6.29E-16	1.62E-14
399	ENSMUSG00000027636	Sla2	1.94	6.38E-16	1.63E-14
400	ENSMUSG00000028359	Orm3	2.13	6.39E-16	1.63E-14
401	ENSMUSG00000029066	Mrpl20	2.33	6.43E-16	1.64E-14
402	ENSMUSG00000106019	Gm43672	2.44	7.39E-16	1.88E-14
403	ENSMUSG00000055239	Kcmf1	1.91	7.40E-16	1.88E-14
404	ENSMUSG00000036545	Adamts2	-3.46	7.45E-16	1.89E-14
405	ENSMUSG00000023939	Mrpl14	2.22	7.54E-16	1.90E-14
406	ENSMUSG00000034203	Chchd4	1.94	7.55E-16	1.90E-14
407	ENSMUSG00000079511	Gm42688	2.05	7.59E-16	1.91E-14
408	ENSMUSG00000042670	Immp1l	2.11	7.62E-16	1.91E-14
409	ENSMUSG00000019139	Isyna1	2.29	7.64E-16	1.91E-14
410	ENSMUSG00000022615	Tymp	2.10	7.70E-16	1.92E-14
411	ENSMUSG00000018585	Atox1	2.46	7.82E-16	1.95E-14
412	ENSMUSG00000048490	Nrip1	-2.72	7.99E-16	1.98E-14
413	ENSMUSG00000056228	Cars2	2.42	8.01E-16	1.98E-14
414	ENSMUSG00000027133	Nop10	2.38	8.04E-16	1.99E-14
415	ENSMUSG00000041697	Cox6a1	2.55	8.12E-16	2.00E-14
416	ENSMUSG00000064215	Ifi27	2.36	8.60E-16	2.11E-14
417	ENSMUSG00000074794	Arrdc3	-2.34	8.84E-16	2.17E-14
418	ENSMUSG00000031503	Col4a2	-2.20	8.95E-16	2.19E-14
419	ENSMUSG00000079036	Alkbh1	2.16	9.32E-16	2.27E-14
420	ENSMUSG00000031834	Pik3r2	-1.88	9.54E-16	2.32E-14
421	ENSMUSG00000032959	Pebp1	1.95	9.56E-16	2.32E-14
422	ENSMUSG00000036860	Mrpl55	2.05	9.84E-16	2.38E-14
423	ENSMUSG00000086527	Gm15856	2.45	1.00E-15	2.42E-14
424	ENSMUSG00000065947	mt-Nd4l	3.37	1.04E-15	2.50E-14
425	ENSMUSG00000051234	Rnf7	2.10	1.06E-15	2.54E-14
426	ENSMUSG00000024664	Fads3	-3.33	1.09E-15	2.62E-14
427	ENSMUSG00000091780	Sco2	2.04	1.10E-15	2.63E-14
428	ENSMUSG00000110279	Gm45552	2.10	1.11E-15	2.66E-14
429	ENSMUSG00000022324	Matn2	-2.68	1.12E-15	2.66E-14
430	ENSMUSG00000067847	Romo1	2.32	1.17E-15	2.79E-14
431	ENSMUSG00000030335	Mrpl51	2.11	1.18E-15	2.79E-14
432	ENSMUSG00000028862	Map3k6	-2.12	1.22E-15	2.89E-14
433	ENSMUSG00000039497	Dse	-2.64	1.23E-15	2.89E-14
434	ENSMUSG00000067212	H2-T23	-2.22	1.23E-15	2.89E-14
435	ENSMUSG00000048537	Phldb1	-2.81	1.23E-15	2.89E-14
436	ENSMUSG00000021226	Acot2	2.24	1.27E-15	2.97E-14
437	ENSMUSG00000014633	Cmc2	2.56	1.33E-15	3.12E-14
438	ENSMUSG00000043155	Hpdl	2.65	1.35E-15	3.14E-14
439	ENSMUSG00000040972	Igsf21	2.57	1.36E-15	3.17E-14
440	ENSMUSG00000059022	Kcp	3.40	1.39E-15	3.22E-14
441	ENSMUSG00000044080	S100a1	2.29	1.40E-15	3.25E-14
442	ENSMUSG00000024208	Uqcc2	2.94	1.47E-15	3.39E-14
443	ENSMUSG00000035828	Pim3	-2.37	1.53E-15	3.53E-14
444	ENSMUSG00000027637	1110008F13Rik	1.92	1.54E-15	3.55E-14
445	ENSMUSG00000026154	Sdhaf4	2.47	1.57E-15	3.61E-14
446	ENSMUSG00000072772	Grcc10	2.08	1.61E-15	3.69E-14
447	ENSMUSG00000022551	Cyc1	2.10	1.62E-15	3.70E-14

448	ENSMUSG00000107368	Gm42574	1.96	1.62E-15	3.70E-14
449	ENSMUSG00000035105	Egln3	2.25	1.72E-15	3.91E-14
450	ENSMUSG00000045569	Mc2r	-2.56	1.77E-15	4.02E-14
451	ENSMUSG00000039518	Cdsn	-3.16	1.79E-15	4.05E-14
452	ENSMUSG00000026032	Ndufb3	2.20	1.80E-15	4.06E-14
453	ENSMUSG00000030647	Ndufc2	2.14	1.80E-15	4.06E-14
454	ENSMUSG00000003072	Atp5d	2.16	1.81E-15	4.08E-14
455	ENSMUSG00000017188	Coa3	2.24	1.82E-15	4.08E-14
456	ENSMUSG00000038894	Irs2	-2.90	1.82E-15	4.08E-14
457	ENSMUSG00000033849	B3galt2	-3.09	1.84E-15	4.11E-14
458	ENSMUSG00000040435	Ppp1r15a	-1.85	1.86E-15	4.15E-14
459	ENSMUSG00000019970	Sgk1	-2.13	1.88E-15	4.18E-14
460	ENSMUSG00000006818	Sod2	2.01	1.89E-15	4.21E-14
461	ENSMUSG00000026811	St6galnac6	2.30	1.91E-15	4.24E-14
462	ENSMUSG00000013629	Cad	2.88	1.93E-15	4.27E-14
463	ENSMUSG00000042688	Mapk6	-2.56	1.96E-15	4.33E-14
464	ENSMUSG00000036478	Btg1	-1.91	2.06E-15	4.54E-14
465	ENSMUSG00000042293	Gm5617	2.48	2.10E-15	4.62E-14
466	ENSMUSG00000021365	Nedd9	-2.41	2.22E-15	4.87E-14
467	ENSMUSG00000005510	Ndufs3	2.07	2.23E-15	4.88E-14
468	ENSMUSG00000037035	Inhbb	-2.84	2.27E-15	4.95E-14
469	ENSMUSG00000020219	Timm13	2.26	2.31E-15	5.04E-14
470	ENSMUSG00000000563	Atp5f1	1.94	2.33E-15	5.06E-14
471	ENSMUSG00000022383	Ppara	2.43	2.36E-15	5.13E-14
472	ENSMUSG00000021905	Dph3	2.19	2.47E-15	5.35E-14
473	ENSMUSG00000026489	Coq8a	2.08	2.48E-15	5.37E-14
474	ENSMUSG00000021906	Oxnad1	1.92	2.49E-15	5.37E-14
475	ENSMUSG00000003429	Rps11	2.11	2.57E-15	5.53E-14
476	ENSMUSG00000022043	Trim35	-1.81	2.60E-15	5.58E-14
477	ENSMUSG00000042312	S100a13	2.76	2.61E-15	5.58E-14
478	ENSMUSG00000027809	Etfdh	1.81	2.62E-15	5.58E-14
479	ENSMUSG00000001891	Ugp2	2.11	2.62E-15	5.58E-14
480	ENSMUSG00000032294	Pkm	2.31	2.62E-15	5.58E-14
481	ENSMUSG00000004655	Aqp1	-2.38	2.62E-15	5.58E-14
482	ENSMUSG00000021040	Slirp	2.03	2.63E-15	5.58E-14
483	ENSMUSG00000020467	Efemp1	-2.52	2.68E-15	5.66E-14
484	ENSMUSG00000036103	Colec12	-2.69	2.80E-15	5.92E-14
485	ENSMUSG00000019850	Tnfaip3	-3.03	2.82E-15	5.94E-14
486	ENSMUSG00000078772	Gm12353	2.51	2.88E-15	6.06E-14
487	ENSMUSG00000036395	Glb1l2	-3.70	2.90E-15	6.09E-14
488	ENSMUSG00000029428	Stx2	-2.18	3.07E-15	6.43E-14
489	ENSMUSG00000040713	Creg1	1.99	3.08E-15	6.44E-14
490	ENSMUSG00000022193	Psmb5	2.02	3.12E-15	6.52E-14
491	ENSMUSG00000030887	Pdzd9	2.01	3.33E-15	6.94E-14
492	ENSMUSG00000060981	Hist1h4h	2.12	3.48E-15	7.24E-14
493	ENSMUSG00000015092	Edf1	2.05	3.54E-15	7.34E-14
494	ENSMUSG00000037151	Lrrc20	3.15	3.63E-15	7.51E-14
495	ENSMUSG00000019577	Pdk4	1.85	3.64E-15	7.51E-14
496	ENSMUSG00000033379	Atp6v0b	1.93	3.65E-15	7.53E-14
497	ENSMUSG00000026519	Tmem63a	-2.15	3.68E-15	7.58E-14

498	ENSMUSG00000027327	1700037H04Rik	1.78	3.70E-15	7.59E-14
499	ENSMUSG00000062619	2310039H08Rik	2.11	3.84E-15	7.87E-14
500	ENSMUSG00000030588	Yif1b	1.96	3.95E-15	8.09E-14
501	ENSMUSG00000028270	Gbp2	-2.19	3.97E-15	8.10E-14
502	ENSMUSG00000038412	Higd1a	2.25	3.98E-15	8.11E-14
503	ENSMUSG00000098470	C1rb	-3.05	4.00E-15	8.13E-14
504	ENSMUSG00000031819	Emc8	2.16	4.29E-15	8.71E-14
505	ENSMUSG00000041351	Rap1gap	2.47	4.32E-15	8.76E-14
506	ENSMUSG00000017307	Acot8	2.07	4.37E-15	8.84E-14
507	ENSMUSG00000016024	Lbp	-3.41	4.44E-15	8.95E-14
508	ENSMUSG00000030137	Tuba8	2.36	4.51E-15	9.08E-14
509	ENSMUSG00000013701	Timm23	1.87	4.53E-15	9.09E-14
510	ENSMUSG00000073418	C4b	-5.41	4.60E-15	9.22E-14
511	ENSMUSG00000021079	Timm9	2.13	4.64E-15	9.29E-14
512	ENSMUSG00000070394	Tmem256	2.41	4.65E-15	9.29E-14
513	ENSMUSG00000005952	Trpv1	-2.80	4.72E-15	9.41E-14
514	ENSMUSG00000030214	Plbd1	1.79	4.74E-15	9.44E-14
515	ENSMUSG00000020267	Hint1	2.11	4.77E-15	9.46E-14
516	ENSMUSG00000059820	AU019823	2.05	4.79E-15	9.49E-14
517	ENSMUSG00000056749	Nfil3	-1.83	4.80E-15	9.49E-14
518	ENSMUSG00000014303	Glis2	2.29	4.94E-15	9.76E-14
519	ENSMUSG00000000399	Ndufa9	1.92	4.97E-15	9.80E-14
520	ENSMUSG00000086107	Gm16312	2.14	5.13E-15	1.01E-13
521	ENSMUSG00000043687	1190005I06Rik	2.07	5.14E-15	1.01E-13
522	ENSMUSG00000067279	Ppp1r3c	1.82	5.48E-15	1.07E-13
523	ENSMUSG00000091228	Gm20390	2.11	5.70E-15	1.12E-13
524	ENSMUSG00000018809	Smyd4	2.15	5.76E-15	1.12E-13
525	ENSMUSG00000055553	Kxd1	2.05	5.82E-15	1.13E-13
526	ENSMUSG00000019978	Epb41l2	-2.31	5.95E-15	1.16E-13
527	ENSMUSG00000020444	Guk1	1.82	6.00E-15	1.16E-13
528	ENSMUSG00000031283	Chrdl1	-3.11	6.01E-15	1.16E-13
529	ENSMUSG00000037960	Card19	2.07	6.01E-15	1.16E-13
530	ENSMUSG00000062580	Timm17a	1.91	6.03E-15	1.16E-13
531	ENSMUSG00000032092	Mpzl2	1.97	6.10E-15	1.18E-13
532	ENSMUSG00000017404	Rpl19	2.18	6.15E-15	1.18E-13
533	ENSMUSG00000085278	Gm12841	1.88	6.24E-15	1.20E-13
534	ENSMUSG00000034729	Mrps10	1.76	6.29E-15	1.20E-13
535	ENSMUSG00000039481	Nrtn	2.48	6.30E-15	1.20E-13
536	ENSMUSG00000024130	Abca3	-2.01	6.64E-15	1.27E-13
537	ENSMUSG00000023967	Mrps18a	1.95	6.71E-15	1.28E-13
538	ENSMUSG00000029135	Fosl2	-2.07	6.77E-15	1.29E-13
539	ENSMUSG00000020857	Nme2	2.12	6.95E-15	1.32E-13
540	ENSMUSG00000037447	Arid5a	-3.03	7.13E-15	1.35E-13
541	ENSMUSG00000051314	Ffar2	-2.78	7.23E-15	1.37E-13
542	ENSMUSG00000037493	Cib2	2.25	7.24E-15	1.37E-13
543	ENSMUSG00000013076	Amotl1	-2.16	7.33E-15	1.38E-13
544	ENSMUSG00000100548	Gm29585	-2.11	7.35E-15	1.38E-13
545	ENSMUSG00000020652	Cenpo	1.71	7.44E-15	1.40E-13
546	ENSMUSG00000073460	Pnldc1	2.90	7.55E-15	1.41E-13
547	ENSMUSG00000023909	Paqr4	-2.67	7.57E-15	1.42E-13

548	ENSMUSG00000002728	Naa20	1.95	7.67E-15	1.43E-13
549	ENSMUSG00000030884	Uqcrc2	1.89	7.71E-15	1.44E-13
550	ENSMUSG00000054312	Mrps21	2.06	8.25E-15	1.53E-13
551	ENSMUSG00000028261	Ndufaf4	1.99	8.26E-15	1.53E-13
552	ENSMUSG00000028600	Podn	-2.88	8.73E-15	1.62E-13
553	ENSMUSG00000038037	Socs1	-2.43	8.77E-15	1.62E-13
554	ENSMUSG00000060923	Acyp2	2.12	8.79E-15	1.62E-13
555	ENSMUSG00000020843	Timm22	1.81	8.99E-15	1.66E-13
556	ENSMUSG00000026879	Gsn	-4.16	9.31E-15	1.71E-13
557	ENSMUSG00000049112	Oxtr	-2.52	9.40E-15	1.73E-13
558	ENSMUSG00000060938	Rpl26	1.99	9.42E-15	1.73E-13
559	ENSMUSG00000002384	Bmp8b	2.59	9.48E-15	1.74E-13
560	ENSMUSG00000107478	Gm45234	1.94	9.52E-15	1.74E-13
561	ENSMUSG00000031137	Fgf13	-2.52	9.67E-15	1.76E-13
562	ENSMUSG00000057666	Gapdh	2.21	9.80E-15	1.78E-13
563	ENSMUSG00000032515	Csrnp1	-2.64	9.98E-15	1.81E-13
564	ENSMUSG00000037966	Ninj1	1.94	1.01E-14	1.82E-13
565	ENSMUSG00000061313	Ddhd2	1.71	1.01E-14	1.83E-13
566	ENSMUSG00000031209	Heph	-3.39	1.01E-14	1.83E-13
567	ENSMUSG00000019846	Lama4	-2.09	1.03E-14	1.85E-13
568	ENSMUSG00000059278	Naa38	2.12	1.03E-14	1.86E-13
569	ENSMUSG00000020644	Id2	2.31	1.03E-14	1.86E-13
570	ENSMUSG00000045502	Hcar2	-2.70	1.04E-14	1.87E-13
571	ENSMUSG00000033059	Pygb	-2.41	1.06E-14	1.90E-13
572	ENSMUSG00000032067	Pts	1.84	1.07E-14	1.91E-13
573	ENSMUSG00000034892	Rps29	2.15	1.10E-14	1.96E-13
574	ENSMUSG00000025745	Hadha	1.82	1.13E-14	2.02E-13
575	ENSMUSG00000024038	Ndufv3	2.16	1.16E-14	2.07E-13
576	ENSMUSG00000026872	Zeb2	-2.20	1.18E-14	2.10E-13
577	ENSMUSG00000110899	AC122305.1	2.02	1.26E-14	2.23E-13
578	ENSMUSG00000021967	Mrpl57	1.90	1.27E-14	2.24E-13
579	ENSMUSG00000073409	H2-Q6	-2.71	1.28E-14	2.26E-13
580	ENSMUSG00000005161	Prdx2	1.80	1.34E-14	2.36E-13
581	ENSMUSG00000046814	Gchfr	2.57	1.42E-14	2.50E-13
582	ENSMUSG00000002900	Lamb1	-1.79	1.43E-14	2.52E-13
583	ENSMUSG00000038319	Kcnh2	-2.69	1.44E-14	2.52E-13
584	ENSMUSG00000014856	Tmem208	1.86	1.45E-14	2.54E-13
585	ENSMUSG00000022817	Itgb5	-1.63	1.45E-14	2.54E-13
586	ENSMUSG00000020282	Rhbdf1	-2.32	1.52E-14	2.64E-13
587	ENSMUSG00000031075	Ano1	2.62	1.55E-14	2.70E-13
588	ENSMUSG00000068566	Myadm	-2.54	1.57E-14	2.72E-13
589	ENSMUSG00000024608	Rps14	2.19	1.57E-14	2.73E-13
590	ENSMUSG00000063524	Eno1	2.05	1.59E-14	2.76E-13
591	ENSMUSG00000043300	B3galnt1	-2.53	1.60E-14	2.76E-13
592	ENSMUSG00000028041	Adam15	-1.99	1.62E-14	2.79E-13
593	ENSMUSG00000035835	Plppr3	-2.18	1.63E-14	2.82E-13
594	ENSMUSG00000024132	Eci1	1.90	1.65E-14	2.83E-13
595	ENSMUSG00000037601	Nme1	1.96	1.65E-14	2.84E-13
596	ENSMUSG00000029918	Mrps33	2.15	1.67E-14	2.87E-13
597	ENSMUSG00000025366	Esyt1	-1.94	1.70E-14	2.91E-13

598	ENSMUSG00000046415	B430212C06Rik	1.98	1.70E-14	2.91E-13
599	ENSMUSG00000106918	Mrpl33	2.10	1.73E-14	2.95E-13
600	ENSMUSG00000019326	Aoc3	-2.15	1.79E-14	3.05E-13
601	ENSMUSG00000020654	Adcy3	1.61	1.85E-14	3.14E-13
602	ENSMUSG00000073096	Lrrc61	-1.97	1.87E-14	3.18E-13
603	ENSMUSG00000022427	Tomm22	2.00	1.91E-14	3.24E-13
604	ENSMUSG00000035885	Cox8a	1.96	1.91E-14	3.24E-13
605	ENSMUSG00000032026	Rexo2	1.71	1.92E-14	3.25E-13
606	ENSMUSG00000045438	Cox19	1.84	1.98E-14	3.34E-13
607	ENSMUSG00000035064	Eef2k	-2.28	2.10E-14	3.53E-13
608	ENSMUSG00000015850	Adamtsl4	-2.39	2.13E-14	3.59E-13
609	ENSMUSG00000006205	Htra1	-2.17	2.15E-14	3.61E-13
610	ENSMUSG00000035385	Ccl2	-2.60	2.16E-14	3.62E-13
611	ENSMUSG00000085687	Gm16153	1.97	2.16E-14	3.62E-13
612	ENSMUSG00000026628	Atf3	-2.23	2.20E-14	3.68E-13
613	ENSMUSG00000035275	Raver2	-2.33	2.25E-14	3.75E-13
614	ENSMUSG00000022219	Cideb	2.69	2.25E-14	3.75E-13
615	ENSMUSG00000025503	Taldo1	1.82	2.28E-14	3.79E-13
616	ENSMUSG00000009378	Slc16a12	-3.07	2.28E-14	3.79E-13
617	ENSMUSG00000041378	Cldn5	-2.66	2.30E-14	3.82E-13
618	ENSMUSG00000003746	Man1a	-1.99	2.32E-14	3.83E-13
619	ENSMUSG00000059447	Hadhb	1.81	2.32E-14	3.83E-13
620	ENSMUSG00000026500	Cox20	1.93	2.41E-14	3.97E-13
621	ENSMUSG00000069744	Psmb3	1.86	2.47E-14	4.07E-13
622	ENSMUSG00000001918	Slc1a5	-2.72	2.49E-14	4.09E-13
623	ENSMUSG00000078695	Cisd3	2.14	2.55E-14	4.18E-13
624	ENSMUSG00000090625	Gm20721	2.01	2.55E-14	4.18E-13
625	ENSMUSG00000015337	Endog	1.93	2.61E-14	4.27E-13
626	ENSMUSG00000027523	Gnas	2.01	2.62E-14	4.29E-13
627	ENSMUSG00000062300	Nectin2	2.17	2.67E-14	4.35E-13
628	ENSMUSG00000018411	Mapt	2.73	2.70E-14	4.39E-13
629	ENSMUSG00000036775	Decr2	1.81	2.78E-14	4.52E-13
630	ENSMUSG00000025735	Rhbdl1	-2.83	2.79E-14	4.53E-13
631	ENSMUSG00000028470	Hint2	2.08	2.82E-14	4.57E-13
632	ENSMUSG00000073877	Gm13306	2.54	2.84E-14	4.60E-13
633	ENSMUSG00000026475	Rgs16	-2.61	2.86E-14	4.62E-13
634	ENSMUSG00000033373	Fntb	1.89	2.87E-14	4.63E-13
635	ENSMUSG00000015575	Atp6v0e	1.87	2.90E-14	4.66E-13
636	ENSMUSG00000046879	Irgm1	-2.24	2.91E-14	4.68E-13
637	ENSMUSG00000020888	Dvl2	1.87	2.91E-14	4.68E-13
638	ENSMUSG00000057229	Atp5sl	1.94	2.93E-14	4.69E-13
639	ENSMUSG00000000058	Cav2	-1.98	2.93E-14	4.69E-13
640	ENSMUSG00000021024	Psma6	1.76	2.94E-14	4.69E-13
641	ENSMUSG00000037905	Bri3bp	2.14	2.96E-14	4.73E-13
642	ENSMUSG00000002395	Use1	1.87	2.97E-14	4.73E-13
643	ENSMUSG00000024640	Psat1	-2.47	2.97E-14	4.73E-13
644	ENSMUSG00000042148	Cox10	1.85	3.01E-14	4.78E-13
645	ENSMUSG00000078974	Sec61g	2.17	3.03E-14	4.80E-13
646	ENSMUSG00000020460	Rps27a	1.93	3.16E-14	5.00E-13
647	ENSMUSG00000036781	Rps27l	2.33	3.17E-14	5.01E-13

648	ENSMUSG00000048078	Tenm4	-2.16	3.17E-14	5.01E-13
649	ENSMUSG00000051748	Wfdc21	-3.09	3.19E-14	5.03E-13
650	ENSMUSG00000038302	Afg1l	1.95	3.20E-14	5.04E-13
651	ENSMUSG00000026827	Gpd2	2.01	3.26E-14	5.12E-13
652	ENSMUSG00000070493	Chchd2	1.91	3.34E-14	5.25E-13
653	ENSMUSG00000006218	Fam131c	3.18	3.36E-14	5.25E-13
654	ENSMUSG00000022437	Samm50	1.57	3.38E-14	5.28E-13
655	ENSMUSG00000045954	Sdpr	-1.96	3.39E-14	5.30E-13
656	ENSMUSG00000025290	Rps24	1.87	3.49E-14	5.44E-13
657	ENSMUSG00000019368	Sec14l4	2.05	3.57E-14	5.55E-13
658	ENSMUSG00000030122	Ptms	1.87	3.62E-14	5.63E-13
659	ENSMUSG00000004069	Dnaja3	1.59	3.72E-14	5.78E-13
660	ENSMUSG00000041515	Irf8	-2.45	3.73E-14	5.78E-13
661	ENSMUSG00000087541	Hopxos	2.40	3.80E-14	5.87E-13
662	ENSMUSG00000028223	Decr1	1.81	3.90E-14	6.03E-13
663	ENSMUSG00000028251	Tstd3	2.11	3.98E-14	6.13E-13
664	ENSMUSG00000037710	Cisd1	1.82	4.02E-14	6.19E-13
665	ENSMUSG00000062908	Acadm	1.74	4.07E-14	6.26E-13
666	ENSMUSG00000087687	Pet100	2.44	4.08E-14	6.26E-13
667	ENSMUSG00000057322	Rpl38	2.58	4.11E-14	6.30E-13
668	ENSMUSG00000098975	Gm27177	1.77	4.30E-14	6.59E-13
669	ENSMUSG00000093954	Gm16867	1.76	4.33E-14	6.61E-13
670	ENSMUSG00000029359	Tesc	2.64	4.42E-14	6.74E-13
671	ENSMUSG00000031375	Bgn	-2.47	4.43E-14	6.75E-13
672	ENSMUSG00000020176	Grb10	-2.53	4.50E-14	6.85E-13
673	ENSMUSG00000028402	Mpdz	-1.85	4.52E-14	6.87E-13
674	ENSMUSG00000018574	Acadvl	1.85	4.60E-14	6.98E-13
675	ENSMUSG00000058267	Mrps14	1.86	4.63E-14	7.02E-13
676	ENSMUSG00000042814	Mcts2	1.93	4.64E-14	7.02E-13
677	ENSMUSG00000018893	Mb	2.18	4.65E-14	7.02E-13
678	ENSMUSG00000031700	Gpt2	-3.04	4.72E-14	7.12E-13
679	ENSMUSG00000025868	Higd2a	1.69	4.73E-14	7.12E-13
680	ENSMUSG00000034932	Mrpl54	2.01	4.74E-14	7.14E-13
681	ENSMUSG00000026170	Cyp27a1	1.82	4.79E-14	7.20E-13
682	ENSMUSG00000030615	Tmem126a	1.84	4.86E-14	7.29E-13
683	ENSMUSG00000063787	Chchd1	2.08	4.88E-14	7.31E-13
684	ENSMUSG00000017765	Slc12a4	-2.08	5.00E-14	7.47E-13
685	ENSMUSG00000078572	Ndufaf8	2.13	5.08E-14	7.58E-13
686	ENSMUSG00000021732	Fgf10	-2.81	5.11E-14	7.62E-13
687	ENSMUSG00000022370	Mrpl13	2.02	5.18E-14	7.70E-13
688	ENSMUSG00000057497	Fam136a	1.57	5.18E-14	7.70E-13
689	ENSMUSG00000022066	Gm21685	1.69	5.19E-14	7.70E-13
690	ENSMUSG00000074064	Mlycd	1.77	5.20E-14	7.71E-13
691	ENSMUSG00000095463	Entpd4	1.69	5.26E-14	7.79E-13
692	ENSMUSG00000078861	Zfp931	1.93	5.37E-14	7.93E-13
693	ENSMUSG00000028517	Plpp3	-1.64	5.58E-14	8.24E-13
694	ENSMUSG00000030612	Mrpl46	2.00	5.70E-14	8.40E-13
695	ENSMUSG00000022452	Smdt1	1.85	5.74E-14	8.44E-13
696	ENSMUSG00000053475	Tnfaip6	-2.88	5.79E-14	8.51E-13
697	ENSMUSG00000074211	Sdhaf1	1.64	5.83E-14	8.55E-13

698	ENSMUSG00000025208	Mrpl43	1.72	5.84E-14	8.56E-13
699	ENSMUSG00000031093	Dock11	-3.02	5.88E-14	8.60E-13
700	ENSMUSG00000064120	Mocs1	-1.70	6.02E-14	8.79E-13
701	ENSMUSG00000005674	Tomm40l	1.60	6.05E-14	8.83E-13
702	ENSMUSG00000068706	Gm10250	2.36	6.19E-14	9.01E-13
703	ENSMUSG00000022111	Uchl3	2.11	6.20E-14	9.01E-13
704	ENSMUSG00000101859	Gm29233	-2.59	6.25E-14	9.08E-13
705	ENSMUSG00000037366	Pafah2	1.95	6.36E-14	9.22E-13
706	ENSMUSG00000041220	Elovl6	2.08	6.38E-14	9.24E-13
707	ENSMUSG00000024940	Ltbp3	-1.71	6.53E-14	9.45E-13
708	ENSMUSG00000062683	Atp5g2	1.62	6.83E-14	9.87E-13
709	ENSMUSG00000029642	Polr1d	1.70	6.89E-14	9.94E-13
710	ENSMUSG00000028780	Sema3c	-1.98	7.07E-14	1.02E-12
711	ENSMUSG00000043445	Pgp	1.65	7.18E-14	1.03E-12
712	ENSMUSG00000004748	Mtfp1	2.05	7.26E-14	1.04E-12
713	ENSMUSG00000029417	Cxcl9	-2.21	7.29E-14	1.05E-12
714	ENSMUSG00000021235	Coq6	1.64	7.43E-14	1.06E-12
715	ENSMUSG00000025495	Ptdss2	1.62	7.48E-14	1.07E-12
716	ENSMUSG00000019528	Gyg	1.55	7.51E-14	1.07E-12
717	ENSMUSG00000025651	Uqcrc1	1.93	7.58E-14	1.08E-12
718	ENSMUSG00000005677	Nr1i3	1.58	7.63E-14	1.09E-12
719	ENSMUSG00000045333	Zfp423	-1.82	7.64E-14	1.09E-12
720	ENSMUSG00000032314	Etfa	1.75	7.85E-14	1.12E-12
721	ENSMUSG00000030483	Cyp2b10	2.88	7.95E-14	1.13E-12
722	ENSMUSG00000017493	Igfbp4	-3.70	8.03E-14	1.14E-12
723	ENSMUSG00000031848	Lsm4	1.86	8.31E-14	1.18E-12
724	ENSMUSG00000052456	Asna1	1.61	8.53E-14	1.20E-12
725	ENSMUSG00000020334	Slc22a4	-2.77	8.55E-14	1.21E-12
726	ENSMUSG00000073888	Ccl27a	2.53	8.57E-14	1.21E-12
727	ENSMUSG00000031987	Egln1	1.66	9.28E-14	1.31E-12
728	ENSMUSG00000027983	Cyp2u1	1.71	9.31E-14	1.31E-12
729	ENSMUSG00000074210	E130208F15Rik	1.62	9.48E-14	1.33E-12
730	ENSMUSG00000037820	Tgm2	-1.67	9.62E-14	1.35E-12
731	ENSMUSG00000020100	Slc29a3	-1.95	9.69E-14	1.36E-12
732	ENSMUSG00000031449	Atp4b	2.43	9.84E-14	1.38E-12
733	ENSMUSG00000028719	Cmpk1	1.53	9.92E-14	1.38E-12
734	ENSMUSG00000097482	Gm17634	2.68	1.02E-13	1.43E-12
735	ENSMUSG00000020057	Dram1	-2.39	1.03E-13	1.43E-12
736	ENSMUSG00000016833	Mrps18c	1.82	1.06E-13	1.47E-12
737	ENSMUSG00000018217	Pmp22	-1.70	1.06E-13	1.47E-12
738	ENSMUSG00000022400	Rbx1	1.73	1.06E-13	1.47E-12
739	ENSMUSG00000024181	Mrpl28	1.73	1.06E-13	1.47E-12
740	ENSMUSG00000034285	Nipsnap1	2.21	1.07E-13	1.48E-12
741	ENSMUSG00000024165	Hn1l	-2.15	1.12E-13	1.55E-12
742	ENSMUSG00000020102	Slc16a7	-2.37	1.12E-13	1.55E-12
743	ENSMUSG00000058966	Fam57b	1.88	1.13E-13	1.56E-12
744	ENSMUSG00000027406	Idh3b	1.77	1.14E-13	1.57E-12
745	ENSMUSG00000021607	Mrpl36	1.88	1.14E-13	1.57E-12
746	ENSMUSG00000019791	Hint3	1.92	1.15E-13	1.58E-12
747	ENSMUSG00000018677	Slc25a39	1.63	1.15E-13	1.58E-12

748	ENSMUSG00000025034	Trim8	-1.80	1.16E-13	1.58E-12
749	ENSMUSG00000056602	Fry	-2.05	1.18E-13	1.61E-12
750	ENSMUSG00000059363	Fxn	2.08	1.27E-13	1.73E-12
751	ENSMUSG00000070867	Trabd2b	-2.80	1.27E-13	1.73E-12
752	ENSMUSG00000051154	Commd3	1.72	1.28E-13	1.73E-12
753	ENSMUSG00000047843	Bri3	2.85	1.29E-13	1.75E-12
754	ENSMUSG00000038253	Hoxa5	2.62	1.30E-13	1.76E-12
755	ENSMUSG00000035673	Sbno2	-1.75	1.32E-13	1.78E-12
756	ENSMUSG00000048351	Coa7	1.80	1.32E-13	1.78E-12
757	ENSMUSG00000042198	Chchd7	1.99	1.36E-13	1.84E-12
758	ENSMUSG00000024829	Mrpl21	2.04	1.38E-13	1.87E-12
759	ENSMUSG00000055782	Abcd2	-2.72	1.39E-13	1.87E-12
760	ENSMUSG00000005125	Ndrg1	-2.38	1.41E-13	1.90E-12
761	ENSMUSG00000087574	C030037D09Rik	2.35	1.41E-13	1.90E-12
762	ENSMUSG00000029207	Apbb2	-2.14	1.42E-13	1.91E-12
763	ENSMUSG00000015750	Aph1a	1.77	1.43E-13	1.91E-12
764	ENSMUSG00000062963	Ufc1	1.62	1.43E-13	1.91E-12
765	ENSMUSG00000020477	Mrps24	1.76	1.46E-13	1.95E-12
766	ENSMUSG00000038671	Arfrp1	1.64	1.47E-13	1.96E-12
767	ENSMUSG00000010406	Mrpl52	2.06	1.47E-13	1.96E-12
768	ENSMUSG00000025940	Tmem70	1.54	1.47E-13	1.96E-12
769	ENSMUSG00000062006	Rpl34	2.11	1.48E-13	1.96E-12
770	ENSMUSG00000091803	Cox16	1.75	1.48E-13	1.96E-12
771	ENSMUSG00000036046	5031439G07Rik	1.57	1.53E-13	2.03E-12
772	ENSMUSG00000031807	Pgls	1.64	1.53E-13	2.03E-12
773	ENSMUSG00000075486	Commd6	1.67	1.63E-13	2.16E-12
774	ENSMUSG00000052962	Mrpl35	1.64	1.66E-13	2.19E-12
775	ENSMUSG00000007655	Cav1	-1.76	1.66E-13	2.19E-12
776	ENSMUSG00000030652	Coq7	1.79	1.67E-13	2.20E-12
777	ENSMUSG00000039670	Oxld1	1.92	1.69E-13	2.22E-12
778	ENSMUSG00000028184	Adgrl2	-2.41	1.70E-13	2.23E-12
779	ENSMUSG00000055839	Elob	1.81	1.70E-13	2.24E-12
780	ENSMUSG00000040112	Mrps35	1.73	1.73E-13	2.26E-12
781	ENSMUSG00000021025	Nfkbia	-1.78	1.73E-13	2.27E-12
782	ENSMUSG00000008206	Cers4	-2.04	1.73E-13	2.27E-12
783	ENSMUSG00000030095	Tmem43	-2.61	1.74E-13	2.27E-12
784	ENSMUSG00000079316	Rab9	1.73	1.80E-13	2.35E-12
785	ENSMUSG00000022091	Sorbs3	2.06	1.84E-13	2.39E-12
786	ENSMUSG00000019832	Rab32	-2.34	1.84E-13	2.39E-12
787	ENSMUSG00000032462	Pik3cb	-1.85	1.85E-13	2.40E-12
788	ENSMUSG00000022037	Clu	-2.66	1.86E-13	2.41E-12
789	ENSMUSG00000024997	Prdx3	1.59	1.87E-13	2.42E-12
790	ENSMUSG00000071648	Rom1	2.03	1.87E-13	2.42E-12
791	ENSMUSG00000026833	Olfm1	-2.01	1.88E-13	2.44E-12
792	ENSMUSG00000028070	Apoa1bp	1.54	1.90E-13	2.46E-12
793	ENSMUSG00000045973	Slc25a51	1.52	1.91E-13	2.46E-12
794	ENSMUSG00000029622	Arpc1b	-1.93	1.96E-13	2.53E-12
795	ENSMUSG00000000739	Sult5a1	2.21	1.97E-13	2.53E-12
796	ENSMUSG00000079658	Eloc	1.85	1.97E-13	2.53E-12
797	ENSMUSG00000004264	Phb2	1.50	2.00E-13	2.57E-12

798	ENSMUSG00000001211	Agpat3	1.53	2.04E-13	2.62E-12
799	ENSMUSG00000050705	2310061I04Rik	1.75	2.09E-13	2.67E-12
800	ENSMUSG00000005299	Letm1	1.59	2.09E-13	2.67E-12
801	ENSMUSG00000064356	mt-Atp8	3.72	2.11E-13	2.70E-12
802	ENSMUSG00000001761	Smo	-2.18	2.13E-13	2.72E-12
803	ENSMUSG00000024841	Eif1ad	1.63	2.15E-13	2.74E-12
804	ENSMUSG00000008976	Gabpa	1.51	2.18E-13	2.77E-12
805	ENSMUSG00000001552	Jup	-1.60	2.23E-13	2.83E-12
806	ENSMUSG00000018858	Mrpl58	1.72	2.26E-13	2.87E-12
807	ENSMUSG00000061740	Cyp2d22	1.77	2.28E-13	2.89E-12
808	ENSMUSG00000035048	Anapc13	1.96	2.32E-13	2.93E-12
809	ENSMUSG00000036278	Macrod1	1.84	2.35E-13	2.96E-12
810	ENSMUSG00000034361	Cpne2	-2.35	2.35E-13	2.97E-12
811	ENSMUSG00000061787	Rps17	1.90	2.38E-13	3.00E-12
812	ENSMUSG00000096188	Cmtm4	2.13	2.41E-13	3.03E-12
813	ENSMUSG00000041598	Cdc42ep4	-1.98	2.43E-13	3.06E-12
814	ENSMUSG00000038587	Akap12	-2.07	2.44E-13	3.07E-12
815	ENSMUSG00000049791	Fzd4	-1.87	2.47E-13	3.09E-12
816	ENSMUSG00000026755	Arpc5l	1.82	2.48E-13	3.10E-12
817	ENSMUSG00000014599	Csf1	-2.05	2.48E-13	3.11E-12
818	ENSMUSG00000024446	Rpp21	1.94	2.55E-13	3.18E-12
819	ENSMUSG00000028701	Lurap1	1.72	2.56E-13	3.19E-12
820	ENSMUSG00000052605	Isoc2b	1.77	2.58E-13	3.22E-12
821	ENSMUSG00000020865	Abcc3	-2.31	2.59E-13	3.23E-12
822	ENSMUSG00000074896	Ifit3	-2.71	2.62E-13	3.25E-12
823	ENSMUSG00000032526	Ss18l2	1.89	2.63E-13	3.27E-12
824	ENSMUSG00000019817	Plagl1	-2.62	2.69E-13	3.34E-12
825	ENSMUSG00000003534	Ddr1	-1.89	2.72E-13	3.37E-12
826	ENSMUSG00000031767	Nudt7	1.49	2.75E-13	3.40E-12
827	ENSMUSG00000047260	Emc6	1.61	2.89E-13	3.57E-12
828	ENSMUSG00000031782	Coq9	1.51	2.90E-13	3.58E-12
829	ENSMUSG00000026821	Ralgds	-2.25	2.93E-13	3.61E-12
830	ENSMUSG00000062488	Ifit3b	-2.42	2.94E-13	3.63E-12
831	ENSMUSG00000044792	Isca1	1.45	2.97E-13	3.65E-12
832	ENSMUSG00000028763	Hspg2	-1.90	2.98E-13	3.66E-12
833	ENSMUSG00000109841	E330011O21Rik	2.47	3.03E-13	3.72E-12
834	ENSMUSG00000028568	Btf3l4	1.58	3.04E-13	3.73E-12
835	ENSMUSG00000017009	Sdc4	1.78	3.06E-13	3.74E-12
836	ENSMUSG00000102495	Gm26524	2.14	3.06E-13	3.74E-12
837	ENSMUSG00000057789	Bak1	2.39	3.08E-13	3.76E-12
838	ENSMUSG00000021773	Comtd1	1.91	3.08E-13	3.76E-12
839	ENSMUSG00000034810	Scn7a	-3.20	3.09E-13	3.76E-12
840	ENSMUSG00000000838	Fmr1	-1.92	3.17E-13	3.86E-12
841	ENSMUSG00000029860	Zyx	-2.05	3.22E-13	3.91E-12
842	ENSMUSG00000037022	Mmaa	1.72	3.24E-13	3.93E-12
843	ENSMUSG00000022404	Slc25a17	1.49	3.27E-13	3.96E-12
844	ENSMUSG00000030934	Oat	-1.46	3.32E-13	4.02E-12
845	ENSMUSG00000028992	Nmnat1	1.70	3.37E-13	4.07E-12
846	ENSMUSG00000044337	Ackr3	2.08	3.42E-13	4.13E-12
847	ENSMUSG00000003299	Mrpl4	1.61	3.43E-13	4.14E-12

848	ENSMUSG00000060073	Psma3	1.59	3.50E-13	4.23E-12
849	ENSMUSG00000036667	Tcaf1	-2.26	3.53E-13	4.25E-12
850	ENSMUSG00000006800	Sulf2	-2.46	3.58E-13	4.30E-12
851	ENSMUSG00000021823	Vcl	-1.87	3.60E-13	4.33E-12
852	ENSMUSG00000020122	Egfr	-2.24	3.62E-13	4.34E-12
853	ENSMUSG00000050732	Vamp8	1.72	3.71E-13	4.45E-12
854	ENSMUSG00000050989	Selenon	-2.19	3.77E-13	4.52E-12
855	ENSMUSG00000092283	Gm20412	-1.67	3.79E-13	4.53E-12
856	ENSMUSG00000090564	A430057M04Rik	1.92	3.86E-13	4.61E-12
857	ENSMUSG00000002043	Trappc6a	1.60	3.88E-13	4.63E-12
858	ENSMUSG00000042541	Sem1	1.87	3.94E-13	4.70E-12
859	ENSMUSG00000066361	Serpina3c	-3.47	4.02E-13	4.79E-12
860	ENSMUSG00000029050	Ski	-1.77	4.04E-13	4.80E-12
861	ENSMUSG00000015478	Rnf5	1.50	4.17E-13	4.95E-12
862	ENSMUSG00000032024	Clmp	-2.82	4.22E-13	5.00E-12
863	ENSMUSG00000070730	Rmdn3	1.49	4.27E-13	5.06E-12
864	ENSMUSG00000102918	Pcdhgc3	-1.92	4.32E-13	5.11E-12
865	ENSMUSG00000091537	Tma7	1.61	4.39E-13	5.19E-12
866	ENSMUSG00000057729	Prtn3	-3.16	4.51E-13	5.33E-12
867	ENSMUSG00000071866	Ppia	1.64	4.55E-13	5.37E-12
868	ENSMUSG00000021139	Gm20498	1.62	4.75E-13	5.60E-12
869	ENSMUSG00000020775	Mrpl38	1.53	4.78E-13	5.63E-12
870	ENSMUSG00000029287	Tgfbr3	-1.85	4.85E-13	5.69E-12
871	ENSMUSG00000024953	Prdx5	1.63	4.85E-13	5.69E-12
872	ENSMUSG00000045377	Tmem88	1.71	4.86E-13	5.70E-12
873	ENSMUSG00000060636	Rpl35a	2.12	4.96E-13	5.81E-12
874	ENSMUSG00000026179	Pnkd	1.65	4.98E-13	5.83E-12
875	ENSMUSG00000051989	Smim11	1.94	4.99E-13	5.83E-12
876	ENSMUSG00000022912	Pros1	-2.38	4.99E-13	5.83E-12
877	ENSMUSG00000019066	Rab3d	-1.76	5.08E-13	5.93E-12
878	ENSMUSG00000022206	Npr3	-2.04	5.15E-13	6.00E-12
879	ENSMUSG00000026185	Igfbp5	-2.00	5.24E-13	6.09E-12
880	ENSMUSG00000031616	Ednra	-2.75	5.24E-13	6.09E-12
881	ENSMUSG00000038121	Fam210a	1.47	5.25E-13	6.09E-12
882	ENSMUSG00000015846	Rxra	-1.90	5.32E-13	6.17E-12
883	ENSMUSG00000019797	1700021F05Rik	1.73	5.34E-13	6.18E-12
884	ENSMUSG00000087253	Gm12043	2.04	5.34E-13	6.18E-12
885	ENSMUSG00000033307	Mif	1.86	5.41E-13	6.25E-12
886	ENSMUSG00000049580	Tsku	1.90	5.47E-13	6.30E-12
887	ENSMUSG00000031351	Zfp185	-2.32	5.47E-13	6.30E-12
888	ENSMUSG00000030695	Aldoa	1.95	5.53E-13	6.37E-12
889	ENSMUSG00000090946	Ccdc71l	-2.02	5.56E-13	6.40E-12
890	ENSMUSG00000047604	Frat2	-2.49	5.59E-13	6.42E-12
891	ENSMUSG00000097059	Fam120aos	1.74	5.63E-13	6.46E-12
892	ENSMUSG00000022602	Arc	-2.43	5.65E-13	6.48E-12
893	ENSMUSG00000050323	Ndufaf6	1.84	5.68E-13	6.50E-12
894	ENSMUSG00000007682	Dio2	2.32	5.76E-13	6.59E-12
895	ENSMUSG00000029108	Pcdh7	-2.28	5.84E-13	6.68E-12
896	ENSMUSG00000031938	4931406C07Rik	1.79	5.96E-13	6.80E-12
897	ENSMUSG00000016344	Ppdpf	1.73	5.99E-13	6.83E-12

898	ENSMUSG00000040249	Lrp1	-1.83	6.14E-13	6.99E-12
899	ENSMUSG00000069266	Hist1h4b	1.92	6.31E-13	7.18E-12
900	ENSMUSG00000075229	Ccdc58	2.07	6.35E-13	7.22E-12
901	ENSMUSG00000021127	Zfp36l1	-1.79	6.40E-13	7.27E-12
902	ENSMUSG00000025525	Apool	1.55	6.42E-13	7.27E-12
903	ENSMUSG00000061315	Naca	1.71	6.45E-13	7.30E-12
904	ENSMUSG00000022425	Enpp2	-1.78	6.56E-13	7.42E-12
905	ENSMUSG00000008090	Fgfrl1	-1.57	6.63E-13	7.50E-12
906	ENSMUSG00000004789	Dlst	1.48	6.69E-13	7.55E-12
907	ENSMUSG00000022571	Pycrl	1.59	6.79E-13	7.65E-12
908	ENSMUSG00000039286	Fndc3b	-2.11	6.80E-13	7.65E-12
909	ENSMUSG00000032193	Ldlr	-1.57	6.87E-13	7.72E-12
910	ENSMUSG00000087327	Gm15884	1.42	6.91E-13	7.77E-12
911	ENSMUSG00000034259	Exosc4	1.53	6.96E-13	7.80E-12
912	ENSMUSG00000036622	Atp13a2	-1.80	6.96E-13	7.80E-12
913	ENSMUSG00000003444	Med29	1.71	7.03E-13	7.88E-12
914	ENSMUSG00000047735	Samd9l	-2.41	7.07E-13	7.91E-12
915	ENSMUSG00000032403	2300009A05Rik	1.63	7.12E-13	7.96E-12
916	ENSMUSG00000031565	Fgfr1	-1.87	7.47E-13	8.34E-12
917	ENSMUSG00000021565	Slc6a19	2.32	7.60E-13	8.47E-12
918	ENSMUSG00000046027	Stard5	1.53	7.62E-13	8.48E-12
919	ENSMUSG00000023908	Pkmyt1	-2.21	7.62E-13	8.48E-12
920	ENSMUSG00000024921	Smarca2	-1.80	7.66E-13	8.51E-12
921	ENSMUSG00000030317	Timp4	-1.69	7.68E-13	8.53E-12
922	ENSMUSG00000021719	Rgs7bp	-2.32	7.85E-13	8.70E-12
923	ENSMUSG00000031748	Gnao1	1.91	7.85E-13	8.70E-12
924	ENSMUSG00000023094	Msrb2	1.55	8.00E-13	8.85E-12
925	ENSMUSG00000026525	Opn3	-2.51	8.29E-13	9.17E-12
926	ENSMUSG00000042569	Dhrs7b	1.42	8.37E-13	9.24E-12
927	ENSMUSG00000026688	Mgst3	-1.45	8.47E-13	9.35E-12
928	ENSMUSG00000038663	Fsd2	-1.95	8.51E-13	9.37E-12
929	ENSMUSG00000021496	Pcbd2	1.74	8.70E-13	9.58E-12
930	ENSMUSG00000037971	1110032A03Rik	1.43	8.73E-13	9.59E-12
931	ENSMUSG00000046764	A530053G22Rik	1.74	8.74E-13	9.60E-12
932	ENSMUSG00000003068	Stk11	2.10	8.89E-13	9.76E-12
933	ENSMUSG00000022865	Cxadr	2.20	9.04E-13	9.90E-12
934	ENSMUSG00000042190	Cmklr1	-2.51	9.06E-13	9.92E-12
935	ENSMUSG00000028247	Coq3	1.48	9.62E-13	1.05E-11
936	ENSMUSG00000018286	Psmb6	1.60	9.88E-13	1.08E-11
937	ENSMUSG00000105617	Gm43809	1.86	9.91E-13	1.08E-11
938	ENSMUSG00000032702	Kank1	-1.86	1.01E-12	1.10E-11
939	ENSMUSG00000089847	Timm10b	1.82	1.02E-12	1.11E-11
940	ENSMUSG00000029338	Antxr2	-1.93	1.02E-12	1.11E-11
941	ENSMUSG00000063316	Rpl27	1.78	1.02E-12	1.11E-11
942	ENSMUSG00000105837	Gm35986	2.21	1.04E-12	1.13E-11
943	ENSMUSG00000022477	Aco2	1.58	1.05E-12	1.13E-11
944	ENSMUSG00000034254	Agpat1	1.45	1.05E-12	1.14E-11
945	ENSMUSG00000038181	Chpf2	-1.63	1.06E-12	1.14E-11
946	ENSMUSG00000076617	Ighm	-1.93	1.06E-12	1.14E-11
947	ENSMUSG00000002588	Pon1	-1.93	1.06E-12	1.14E-11

948	ENSMUSG00000039234	Sec24d	-1.83	1.06E-12	1.14E-11
949	ENSMUSG00000068523	Gng5	1.63	1.07E-12	1.16E-11
950	ENSMUSG00000027274	Mkks	1.70	1.08E-12	1.16E-11
951	ENSMUSG00000033020	Polr2f	1.76	1.10E-12	1.18E-11
952	ENSMUSG00000087505	Gm15241	2.98	1.10E-12	1.19E-11
953	ENSMUSG00000067288	Rps28	1.74	1.11E-12	1.20E-11
954	ENSMUSG00000037058	Paip2	1.39	1.12E-12	1.20E-11
955	ENSMUSG00000049892	Rasd1	-1.86	1.12E-12	1.20E-11
956	ENSMUSG00000086769	Gm15587	-2.17	1.15E-12	1.23E-11
957	ENSMUSG00000001134	Uxt	1.86	1.16E-12	1.24E-11
958	ENSMUSG00000030587	2200002D01Rik	1.68	1.16E-12	1.24E-11
959	ENSMUSG00000040269	Mrps28	1.77	1.18E-12	1.25E-11
960	ENSMUSG00000068874	Selenbp1	-1.49	1.19E-12	1.26E-11
961	ENSMUSG00000024190	Dusp1	-1.50	1.19E-12	1.26E-11
962	ENSMUSG00000025277	Abhd6	1.39	1.19E-12	1.27E-11
963	ENSMUSG00000025508	Rplp2	1.92	1.20E-12	1.27E-11
964	ENSMUSG00000029661	Col1a2	-2.16	1.20E-12	1.27E-11
965	ENSMUSG00000096826	Ccl27b	2.35	1.20E-12	1.28E-11
966	ENSMUSG00000103472	Pcdhga7	-1.77	1.21E-12	1.28E-11
967	ENSMUSG00000041115	Iqsec2	2.25	1.25E-12	1.33E-11
968	ENSMUSG00000028207	Asph	-2.30	1.26E-12	1.33E-11
969	ENSMUSG00000031451	Gas6	-1.92	1.26E-12	1.33E-11
970	ENSMUSG00000027762	Sucnr1	-2.12	1.27E-12	1.33E-11
971	ENSMUSG00000062997	Rpl35	1.84	1.27E-12	1.34E-11
972	ENSMUSG00000024146	Cript	1.48	1.29E-12	1.35E-11
973	ENSMUSG00000028959	Fastk	1.36	1.29E-12	1.35E-11
974	ENSMUSG00000028982	Slc25a33	1.55	1.31E-12	1.37E-11
975	ENSMUSG00000104761	Gm43511	1.76	1.32E-12	1.38E-11
976	ENSMUSG00000090553	Snrpe	1.89	1.36E-12	1.43E-11
977	ENSMUSG00000019362	D8Ertd738e	1.72	1.38E-12	1.44E-11
978	ENSMUSG00000035772	Mrps2	1.53	1.38E-12	1.44E-11
979	ENSMUSG00000025781	Atp5c1	1.52	1.39E-12	1.45E-11
980	ENSMUSG00000073411	H2-D1	-2.20	1.39E-12	1.45E-11
981	ENSMUSG00000041362	Shtn1	2.47	1.39E-12	1.45E-11
982	ENSMUSG00000086539	Gm16759	1.60	1.40E-12	1.46E-11
983	ENSMUSG00000092511	Gm20547	-3.72	1.40E-12	1.46E-11
984	ENSMUSG00000110234	Gm45799	1.76	1.43E-12	1.49E-11
985	ENSMUSG00000041229	Phf8	-2.00	1.44E-12	1.50E-11
986	ENSMUSG00000073433	Arhgdig	2.74	1.45E-12	1.50E-11
987	ENSMUSG00000002660	Clpp	1.54	1.47E-12	1.52E-11
988	ENSMUSG00000039395	Mreg	2.55	1.47E-12	1.52E-11
989	ENSMUSG00000034595	Ppp1r18	-2.18	1.51E-12	1.57E-11
990	ENSMUSG00000035764	Fbxo45	-2.09	1.53E-12	1.58E-11
991	ENSMUSG00000024197	Plin3	1.54	1.54E-12	1.59E-11
992	ENSMUSG00000055737	Ghr	-2.07	1.55E-12	1.60E-11
993	ENSMUSG00000103037	Pcdhgb1	-1.77	1.58E-12	1.63E-11
994	ENSMUSG00000014243	Zswim7	1.70	1.60E-12	1.64E-11
995	ENSMUSG00000013822	Elof1	1.51	1.60E-12	1.65E-11
996	ENSMUSG00000031950	Gabarapl2	1.52	1.63E-12	1.68E-11
997	ENSMUSG00000103897	Pcdhga8	-1.77	1.67E-12	1.71E-11

998	ENSMUSG00000038776	Ephx1	1.67	1.67E-12	1.71E-11
999	ENSMUSG00000028978	Nos3	-2.00	1.67E-12	1.71E-11
1000	ENSMUSG00000009927	Rps25	1.54	1.69E-12	1.73E-11
1001	ENSMUSG00000039640	Mrpl12	1.54	1.70E-12	1.73E-11
1002	ENSMUSG00000103567	Pcdhga5	-1.78	1.70E-12	1.74E-11
1003	ENSMUSG00000002688	Prkd1	-1.84	1.71E-12	1.74E-11
1004	ENSMUSG00000068742	Cry2	-1.51	1.73E-12	1.76E-11
1005	ENSMUSG00000057614	Gnai1	-1.74	1.73E-12	1.76E-11
1006	ENSMUSG00000015806	Qdpr	1.35	1.75E-12	1.78E-11
1007	ENSMUSG00000103749	Pcdhgb5	-1.75	1.76E-12	1.78E-11
1008	ENSMUSG00000102742	Pcdhga11	-1.76	1.76E-12	1.79E-11
1009	ENSMUSG00000102440	Pcdhga9	-1.76	1.78E-12	1.80E-11
1010	ENSMUSG00000104346	Pcdhga3	-1.76	1.82E-12	1.84E-11
1011	ENSMUSG00000097919	Gm27021	1.54	1.83E-12	1.85E-11
1012	ENSMUSG00000028494	Plin2	1.67	1.85E-12	1.87E-11
1013	ENSMUSG00000027322	Siglec1	-2.42	1.85E-12	1.87E-11
1014	ENSMUSG00000102748	Pcdhgb2	-1.77	1.85E-12	1.87E-11
1015	ENSMUSG00000052428	Tmco1	1.41	1.88E-12	1.89E-11
1016	ENSMUSG00000103081	Pcdhgb8	-1.76	1.89E-12	1.91E-11
1017	ENSMUSG00000103677	Pcdhga4	-1.77	1.92E-12	1.93E-11
1018	ENSMUSG00000053898	Ech1	1.70	1.93E-12	1.94E-11
1019	ENSMUSG00000052957	Gas1	2.48	1.93E-12	1.94E-11
1020	ENSMUSG00000103144	Pcdhga1	-1.76	1.93E-12	1.94E-11
1021	ENSMUSG00000112058	AC165164.1	2.09	1.94E-12	1.95E-11
1022	ENSMUSG00000102543	Pcdhgc5	-1.76	1.96E-12	1.96E-11
1023	ENSMUSG00000018143	Mafk	-1.75	2.00E-12	2.00E-11
1024	ENSMUSG00000103793	Pcdhga6	-1.77	2.01E-12	2.01E-11
1025	ENSMUSG00000089671	Gm16537	1.69	2.04E-12	2.03E-11
1026	ENSMUSG00000058743	Kcnj14	-2.41	2.06E-12	2.06E-11
1027	ENSMUSG00000037772	Mrpl23	1.69	2.08E-12	2.07E-11
1028	ENSMUSG00000039910	Cited2	-2.01	2.09E-12	2.08E-11
1029	ENSMUSG00000042737	Dpm3	1.71	2.10E-12	2.09E-11
1030	ENSMUSG00000042682	Selenok	1.56	2.10E-12	2.09E-11
1031	ENSMUSG00000097004	4731419I09Rik	-1.76	2.12E-12	2.10E-11
1032	ENSMUSG00000032757	Bet1	1.61	2.14E-12	2.12E-11
1033	ENSMUSG00000026939	Tmem141	1.67	2.15E-12	2.13E-11
1034	ENSMUSG00000045996	Polr2k	1.84	2.16E-12	2.14E-11
1035	ENSMUSG00000021509	Slc25a48	2.25	2.17E-12	2.14E-11
1036	ENSMUSG00000004846	Plod3	-1.69	2.17E-12	2.14E-11
1037	ENSMUSG00000103332	Pcdhga2	-1.76	2.17E-12	2.14E-11
1038	ENSMUSG00000102222	Pcdhga10	-1.75	2.20E-12	2.16E-11
1039	ENSMUSG00000102428	Pcdhga12	-1.75	2.20E-12	2.17E-11
1040	ENSMUSG00000061232	H2-K1	-1.96	2.25E-12	2.21E-11
1041	ENSMUSG00000029413	Naaa	-1.57	2.26E-12	2.22E-11
1042	ENSMUSG00000046312	AI464131	-2.00	2.26E-12	2.22E-11
1043	ENSMUSG00000058600	Rpl30	1.74	2.33E-12	2.28E-11
1044	ENSMUSG00000002580	Mien1	1.62	2.38E-12	2.33E-11
1045	ENSMUSG00000103088	Pcdhgb6	-1.76	2.39E-12	2.34E-11
1046	ENSMUSG00000037106	Fer1l6	1.93	2.39E-12	2.34E-11
1047	ENSMUSG00000087269	D330023K18Rik	1.80	2.42E-12	2.37E-11

1048	ENSMUSG00000084835	Gm12352	1.56	2.42E-12	2.37E-11
1049	ENSMUSG00000001802	Lrp3	-1.66	2.43E-12	2.37E-11
1050	ENSMUSG00000092395	Gm20463	1.48	2.46E-12	2.40E-11
1051	ENSMUSG00000002949	Timm44	1.45	2.47E-12	2.40E-11
1052	ENSMUSG00000034854	Mfsd12	-2.27	2.50E-12	2.43E-11
1053	ENSMUSG00000021748	Pdhb	1.61	2.52E-12	2.44E-11
1054	ENSMUSG00000031813	Mvb12a	1.51	2.55E-12	2.47E-11
1055	ENSMUSG00000015090	Ptgds	3.01	2.55E-12	2.47E-11
1056	ENSMUSG00000004040	Stat3	-1.44	2.58E-12	2.50E-11
1057	ENSMUSG00000034449	Dhrs11	1.72	2.60E-12	2.52E-11
1058	ENSMUSG00000103585	Pcdhgb4	-1.76	2.61E-12	2.52E-11
1059	ENSMUSG00000027035	Cers6	-2.37	2.69E-12	2.60E-11
1060	ENSMUSG00000044627	Swi5	1.54	2.73E-12	2.64E-11
1061	ENSMUSG00000022751	Nit2	1.50	2.74E-12	2.64E-11
1062	ENSMUSG00000066363	Serpina3f	-2.37	2.75E-12	2.65E-11
1063	ENSMUSG00000035517	Tdrd7	-1.73	2.78E-12	2.67E-11
1064	ENSMUSG00000097658	Gm16755	1.50	2.79E-12	2.68E-11
1065	ENSMUSG00000057863	Rpl36	2.00	2.79E-12	2.68E-11
1066	ENSMUSG00000023036	Pcdhgc4	-1.75	2.79E-12	2.68E-11
1067	ENSMUSG00000002984	Tomm40	1.40	2.80E-12	2.68E-11
1068	ENSMUSG00000061758	Akr1b10	1.56	2.81E-12	2.69E-11
1069	ENSMUSG00000028300	3110043O21Rik	-1.43	2.84E-12	2.72E-11
1070	ENSMUSG00000087382	Ctcflos	1.67	2.85E-12	2.73E-11
1071	ENSMUSG00000001056	Nhp2	1.50	2.87E-12	2.74E-11
1072	ENSMUSG00000006362	Cbfa2t3	2.08	2.89E-12	2.76E-11
1073	ENSMUSG00000029513	Prkab1	1.67	2.89E-12	2.76E-11
1074	ENSMUSG00000024066	Xdh	-1.57	2.90E-12	2.76E-11
1075	ENSMUSG00000078308	Gm7293	1.71	2.90E-12	2.76E-11
1076	ENSMUSG00000025137	Pcyt2	1.39	2.90E-12	2.76E-11
1077	ENSMUSG00000079037	Prnp	-1.79	2.95E-12	2.80E-11
1078	ENSMUSG00000056204	Pgpep1	1.51	2.98E-12	2.83E-11
1079	ENSMUSG00000001289	Pfdn5	1.67	3.01E-12	2.85E-11
1080	ENSMUSG00000026003	Acadl	1.46	3.04E-12	2.87E-11
1081	ENSMUSG00000004044	Ptrf	-2.21	3.05E-12	2.89E-11
1082	ENSMUSG00000104063	Pcdhgb7	-1.73	3.06E-12	2.89E-11
1083	ENSMUSG00000087249	Gm16062	1.76	3.08E-12	2.91E-11
1084	ENSMUSG00000063320	1190007I07Rik	2.06	3.08E-12	2.91E-11
1085	ENSMUSG00000098274	Rpl24	1.53	3.11E-12	2.94E-11
1086	ENSMUSG00000020205	Phlda1	-2.04	3.12E-12	2.94E-11
1087	ENSMUSG00000022244	Amacr	1.42	3.14E-12	2.96E-11
1088	ENSMUSG00000085403	Gm13068	1.55	3.15E-12	2.96E-11
1089	ENSMUSG00000098371	Gm28037	1.51	3.36E-12	3.16E-11
1090	ENSMUSG00000019464	Ptger1	1.61	3.38E-12	3.17E-11
1091	ENSMUSG00000038489	Polr2l	1.62	3.42E-12	3.20E-11
1092	ENSMUSG00000019158	Tmem160	1.77	3.45E-12	3.23E-11
1093	ENSMUSG00000047215	Rpl9	1.64	3.48E-12	3.25E-11
1094	ENSMUSG00000040274	Cdk6	1.88	3.49E-12	3.26E-11
1095	ENSMUSG00000050315	Synpo2	-2.76	3.49E-12	3.26E-11
1096	ENSMUSG00000074129	Rpl13a	1.77	3.52E-12	3.28E-11
1097	ENSMUSG00000029596	Sdsl	1.69	3.54E-12	3.30E-11

1098	ENSMUSG00000007892	Rplp1	1.70	3.62E-12	3.37E-11
1099	ENSMUSG00000084998	Gm16279	1.41	3.63E-12	3.38E-11
1100	ENSMUSG00000027679	Dnajc19	1.47	3.67E-12	3.41E-11
1101	ENSMUSG00000041841	Rpl37	1.78	3.71E-12	3.45E-11
1102	ENSMUSG00000071655	Ubxn1	1.53	3.73E-12	3.47E-11
1103	ENSMUSG00000023571	C1qtnf12	1.28	3.75E-12	3.47E-11
1104	ENSMUSG00000075700	Selenot	1.82	3.75E-12	3.47E-11
1105	ENSMUSG00000003617	Cp	-2.36	3.78E-12	3.50E-11
1106	ENSMUSG00000025823	Pdia4	-1.75	3.92E-12	3.62E-11
1107	ENSMUSG00000028691	Prdx1	1.47	3.98E-12	3.67E-11
1108	ENSMUSG00000049751	Rpl36al	1.68	4.02E-12	3.71E-11
1109	ENSMUSG00000034211	Mrps17	1.41	4.04E-12	3.72E-11
1110	ENSMUSG00000029152	Ociad1	1.41	4.06E-12	3.74E-11
1111	ENSMUSG00000029166	Mapre3	1.82	4.11E-12	3.79E-11
1112	ENSMUSG00000010911	Apip	1.44	4.14E-12	3.81E-11
1113	ENSMUSG00000042505	Sdhaf3	1.62	4.21E-12	3.86E-11
1114	ENSMUSG00000029911	Ssbp1	1.88	4.25E-12	3.90E-11
1115	ENSMUSG00000042041	2010003K11Rik	1.60	4.36E-12	4.00E-11
1116	ENSMUSG00000071001	Hrct1	-1.71	4.40E-12	4.03E-11
1117	ENSMUSG00000051041	Olfml1	-2.54	4.41E-12	4.04E-11
1118	ENSMUSG00000042401	Crtac1	-2.34	4.44E-12	4.06E-11
1119	ENSMUSG00000027984	Hadh	1.37	4.49E-12	4.10E-11
1120	ENSMUSG00000024661	Fth1	1.54	4.51E-12	4.12E-11
1121	ENSMUSG00000066154	Mup3	-2.12	4.52E-12	4.13E-11
1122	ENSMUSG00000060550	H2-Q7	-2.46	4.53E-12	4.13E-11
1123	ENSMUSG00000024309	Pfdn6	1.77	4.54E-12	4.13E-11
1124	ENSMUSG00000089989	Gm45713	1.76	4.55E-12	4.13E-11
1125	ENSMUSG00000036111	Lmo1	1.86	4.55E-12	4.13E-11
1126	ENSMUSG00000002227	Mov10	-2.32	4.59E-12	4.17E-11
1127	ENSMUSG00000020514	Mrpl22	1.64	4.61E-12	4.19E-11
1128	ENSMUSG00000049755	Zfp672	1.33	4.66E-12	4.23E-11
1129	ENSMUSG00000028849	Map7d1	-1.61	4.67E-12	4.23E-11
1130	ENSMUSG00000030737	Slco2b1	-2.29	4.67E-12	4.23E-11
1131	ENSMUSG00000015094	Npdc1	-1.31	4.68E-12	4.23E-11
1132	ENSMUSG00000062753	AI413582	2.14	4.68E-12	4.23E-11
1133	ENSMUSG00000000355	Mcts1	1.45	4.70E-12	4.24E-11
1134	ENSMUSG00000008668	Rps18	1.55	4.73E-12	4.26E-11
1135	ENSMUSG00000038780	Smurf1	-1.52	4.73E-12	4.26E-11
1136	ENSMUSG00000044338	Aplnr	-2.22	4.74E-12	4.26E-11
1137	ENSMUSG00000035242	Oaz1	1.42	4.75E-12	4.27E-11
1138	ENSMUSG00000055148	Klf2	-1.72	4.79E-12	4.30E-11
1139	ENSMUSG00000024277	Mapre2	-1.75	4.80E-12	4.31E-11
1140	ENSMUSG00000035295	Wdr38	1.75	4.82E-12	4.33E-11
1141	ENSMUSG00000032051	Fdx1	1.54	4.93E-12	4.42E-11
1142	ENSMUSG00000022419	Deptor	1.40	4.95E-12	4.43E-11
1143	ENSMUSG00000023019	Gpd1	1.68	4.97E-12	4.45E-11
1144	ENSMUSG00000071662	Polr2g	1.48	5.07E-12	4.53E-11
1145	ENSMUSG00000019864	Rtn4ip1	1.33	5.07E-12	4.53E-11
1146	ENSMUSG00000027829	Ccnl1	-1.59	5.16E-12	4.60E-11
1147	ENSMUSG00000096938	9530052E02Rik	-2.11	5.17E-12	4.61E-11

1148	ENSMUSG00000036106	Prr5	-1.89	5.24E-12	4.66E-11
1149	ENSMUSG00000020415	Pttg1	1.61	5.24E-12	4.66E-11
1150	ENSMUSG00000024899	Papss2	-2.75	5.25E-12	4.67E-11
1151	ENSMUSG00000007817	Zmiz1	-1.76	5.28E-12	4.69E-11
1152	ENSMUSG00000032109	Nlrx1	-1.53	5.30E-12	4.70E-11
1153	ENSMUSG00000027074	Slc43a3	-1.62	5.40E-12	4.79E-11
1154	ENSMUSG00000031605	Klhl2	-2.11	5.44E-12	4.82E-11
1155	ENSMUSG00000003033	Ap1m1	1.44	5.49E-12	4.86E-11
1156	ENSMUSG00000031641	Cbr4	1.48	5.49E-12	4.86E-11
1157	ENSMUSG00000067722	BC003965	1.55	5.53E-12	4.89E-11
1158	ENSMUSG00000051855	Mest	-3.28	5.57E-12	4.92E-11
1159	ENSMUSG00000024844	Banf1	1.53	5.60E-12	4.94E-11
1160	ENSMUSG00000024335	Brd2	-1.37	5.67E-12	5.00E-11
1161	ENSMUSG00000092550	Gm20496	-1.96	5.74E-12	5.06E-11
1162	ENSMUSG00000036309	Skp1a	1.39	5.80E-12	5.10E-11
1163	ENSMUSG00000022840	Adcy5	-3.67	5.85E-12	5.14E-11
1164	ENSMUSG00000027566	Psma7	1.45	5.91E-12	5.19E-11
1165	ENSMUSG00000030495	Slc7a10	-2.39	6.12E-12	5.37E-11
1166	ENSMUSG00000020156	Mum1	-1.68	6.32E-12	5.54E-11
1167	ENSMUSG00000038274	Fau	1.53	6.39E-12	5.60E-11
1168	ENSMUSG00000002014	Ssr4	1.44	6.43E-12	5.63E-11
1169	ENSMUSG00000029772	Ahcyl2	-1.91	6.53E-12	5.71E-11
1170	ENSMUSG00000038963	Slco4a1	2.21	6.61E-12	5.77E-11
1171	ENSMUSG00000023176	Cpn2	2.11	6.61E-12	5.77E-11
1172	ENSMUSG00000038058	Nod1	-1.81	6.68E-12	5.82E-11
1173	ENSMUSG00000027323	Rad51	1.40	6.70E-12	5.84E-11
1174	ENSMUSG00000038301	Snx10	1.30	6.71E-12	5.84E-11
1175	ENSMUSG00000022853	Ehhadh	1.43	6.84E-12	5.95E-11
1176	ENSMUSG00000026260	Ndufa10	1.47	6.85E-12	5.96E-11
1177	ENSMUSG00000016308	Ube2a	1.45	6.87E-12	5.96E-11
1178	ENSMUSG00000048772	Tmem53	1.49	6.87E-12	5.96E-11
1179	ENSMUSG00000014956	Ppp1cb	1.29	6.88E-12	5.96E-11
1180	ENSMUSG00000045246	Kcng4	2.12	6.89E-12	5.97E-11
1181	ENSMUSG00000044345	Marveld1	-1.48	6.90E-12	5.97E-11
1182	ENSMUSG00000045672	Col27a1	2.29	7.10E-12	6.14E-11
1183	ENSMUSG00000028062	Lamtor2	1.47	7.27E-12	6.29E-11
1184	ENSMUSG00000036390	Gadd45a	-1.86	7.41E-12	6.40E-11
1185	ENSMUSG00000024683	Mrpl16	1.51	7.44E-12	6.42E-11
1186	ENSMUSG00000090231	Cfb	-3.64	7.45E-12	6.42E-11
1187	ENSMUSG00000027332	Ivd	-1.36	7.46E-12	6.42E-11
1188	ENSMUSG00000040767	Snrnp25	1.97	7.47E-12	6.43E-11
1189	ENSMUSG00000063428	Ddo	1.55	7.53E-12	6.48E-11
1190	ENSMUSG00000050953	Gja1	1.80	7.68E-12	6.60E-11
1191	ENSMUSG00000021583	Erap1	-1.78	7.76E-12	6.66E-11
1192	ENSMUSG00000031453	Rasa3	-2.76	7.89E-12	6.76E-11
1193	ENSMUSG00000026610	Esrrg	2.09	7.99E-12	6.85E-11
1194	ENSMUSG00000026856	Dolpp1	-1.63	8.00E-12	6.85E-11
1195	ENSMUSG00000086877	A230072C01Rik	1.71	8.00E-12	6.85E-11
1196	ENSMUSG00000038967	Pdk2	1.32	8.12E-12	6.94E-11
1197	ENSMUSG00000045435	Tmem60	1.52	8.13E-12	6.94E-11

1198	ENSMUSG00000027195	Hsd17b12	1.33	8.18E-12	6.99E-11
1199	ENSMUSG00000075704	Txnrd2	1.36	8.22E-12	7.01E-11
1200	ENSMUSG00000051483	Cbr1	1.44	8.25E-12	7.03E-11
1201	ENSMUSG00000032261	Sh3bgrl2	2.00	8.29E-12	7.06E-11
1202	ENSMUSG00000026750	Psmb7	1.32	8.38E-12	7.13E-11
1203	ENSMUSG00000039607	Rbms3	-2.47	8.46E-12	7.19E-11
1204	ENSMUSG00000090000	Ier3ip1	1.64	8.54E-12	7.25E-11
1205	ENSMUSG00000051439	Cd14	-1.90	8.63E-12	7.33E-11
1206	ENSMUSG00000019820	Utrn	-1.64	8.66E-12	7.34E-11
1207	ENSMUSG00000034354	Mtmr3	-1.37	8.70E-12	7.37E-11
1208	ENSMUSG00000031714	Gab1	-1.78	8.79E-12	7.44E-11
1209	ENSMUSG00000028295	Smim8	1.55	8.83E-12	7.47E-11
1210	ENSMUSG00000035847	Ids	-1.82	8.87E-12	7.50E-11
1211	ENSMUSG00000028636	Ppcs	1.59	8.98E-12	7.59E-11
1212	ENSMUSG00000050552	Lamtor4	1.48	9.01E-12	7.60E-11
1213	ENSMUSG00000066839	Ecsit	1.35	9.02E-12	7.60E-11
1214	ENSMUSG00000056870	Gulp1	-2.27	9.03E-12	7.61E-11
1215	ENSMUSG00000041828	Abca8a	-2.25	9.04E-12	7.61E-11
1216	ENSMUSG00000029571	Tmem106b	1.33	9.08E-12	7.63E-11
1217	ENSMUSG00000110058	Gm45519	2.01	9.37E-12	7.87E-11
1218	ENSMUSG00000001665	Gstt3	-1.87	9.39E-12	7.88E-11
1219	ENSMUSG00000065990	Aurkaip1	1.34	9.52E-12	7.99E-11
1220	ENSMUSG00000080058	Gm11175	-1.43	9.57E-12	8.02E-11
1221	ENSMUSG00000055447	Cd47	-1.34	9.72E-12	8.14E-11
1222	ENSMUSG00000040253	Gbp7	-1.83	9.78E-12	8.18E-11
1223	ENSMUSG00000019373	Cops3	1.31	9.84E-12	8.22E-11
1224	ENSMUSG00000030560	Ctsc	-2.29	9.90E-12	8.27E-11
1225	ENSMUSG00000016487	Ppfibp1	-1.55	9.91E-12	8.27E-11
1226	ENSMUSG00000027384	Ndufaf5	1.70	9.92E-12	8.27E-11
1227	ENSMUSG00000048007	Timm8a1	1.64	9.99E-12	8.32E-11
1228	ENSMUSG00000030744	Rps3	1.37	1.01E-11	8.41E-11
1229	ENSMUSG00000071076	Jund	-1.41	1.03E-11	8.56E-11
1230	ENSMUSG00000021963	Sap18	1.32	1.04E-11	8.63E-11
1231	ENSMUSG00000034343	Ube2f	1.34	1.05E-11	8.68E-11
1232	ENSMUSG00000004105	Angptl2	-2.03	1.05E-11	8.72E-11
1233	ENSMUSG00000087380	2210408F21Rik	1.72	1.06E-11	8.75E-11
1234	ENSMUSG00000029659	Medag	-2.11	1.07E-11	8.88E-11
1235	ENSMUSG00000009633	G0s2	1.41	1.11E-11	9.15E-11
1236	ENSMUSG00000062691	Cebpzos	1.71	1.11E-11	9.22E-11
1237	ENSMUSG00000006344	Ggt5	-2.24	1.13E-11	9.34E-11
1238	ENSMUSG00000025421	Hdhd2	1.29	1.14E-11	9.39E-11
1239	ENSMUSG00000021186	Fbln5	-2.01	1.14E-11	9.39E-11
1240	ENSMUSG00000024436	Mrps18b	1.41	1.14E-11	9.39E-11
1241	ENSMUSG00000030287	Itpr2	2.10	1.14E-11	9.43E-11
1242	ENSMUSG00000111013	AC122326.1	2.18	1.15E-11	9.49E-11
1243	ENSMUSG00000093674	Rpl41	1.60	1.17E-11	9.59E-11
1244	ENSMUSG00000033170	Card10	-1.62	1.20E-11	9.83E-11
1245	ENSMUSG00000002985	Apoe	-2.37	1.22E-11	9.98E-11
1246	ENSMUSG00000015016	Acsf3	1.53	1.22E-11	9.99E-11
1247	ENSMUSG00000020544	Cox11	1.55	1.22E-11	1.00E-10

1248	ENSMUSG00000033880	Lgals3bp	-2.03	1.23E-11	1.01E-10
1249	ENSMUSG00000056612	Ppp1r14b	1.47	1.24E-11	1.01E-10
1250	ENSMUSG00000025511	Tspan4	-1.69	1.25E-11	1.02E-10
1251	ENSMUSG00000001270	Ckb	1.77	1.25E-11	1.02E-10
1252	ENSMUSG00000031725	Ces1f	-1.81	1.27E-11	1.03E-10
1253	ENSMUSG00000031441	Atp11a	-2.15	1.27E-11	1.04E-10
1254	ENSMUSG00000028019	Pdgfc	-1.92	1.29E-11	1.05E-10
1255	ENSMUSG00000017774	Myo1c	-1.51	1.29E-11	1.06E-10
1256	ENSMUSG00000051355	Commd1	1.38	1.31E-11	1.06E-10
1257	ENSMUSG00000073565	Prr16	-2.51	1.32E-11	1.07E-10
1258	ENSMUSG00000090862	Rps13	1.58	1.32E-11	1.08E-10
1259	ENSMUSG00000059741	Myl3	2.22	1.33E-11	1.08E-10
1260	ENSMUSG00000068877	Selenbp2	-1.48	1.34E-11	1.09E-10
1261	ENSMUSG00000018554	Ybx2	1.96	1.35E-11	1.10E-10
1262	ENSMUSG00000054452	Aes	1.32	1.36E-11	1.10E-10
1263	ENSMUSG00000063268	Parp10	-1.42	1.36E-11	1.10E-10
1264	ENSMUSG00000033355	Rtp4	-2.38	1.39E-11	1.12E-10
1265	ENSMUSG00000021243	Fcf1	1.61	1.41E-11	1.14E-10
1266	ENSMUSG00000032911	Cspg4	-1.88	1.41E-11	1.14E-10
1267	ENSMUSG00000025428	Atp5a1	1.42	1.42E-11	1.15E-10
1268	ENSMUSG00000039768	Dnajc11	1.33	1.44E-11	1.16E-10
1269	ENSMUSG00000027111	Itga6	-1.65	1.45E-11	1.17E-10
1270	ENSMUSG00000105255	Gm42413	1.25	1.45E-11	1.17E-10
1271	ENSMUSG00000057530	Ece1	-1.39	1.47E-11	1.18E-10
1272	ENSMUSG00000035877	Zhx3	-1.51	1.47E-11	1.18E-10
1273	ENSMUSG00000001995	Sipa1l2	-1.93	1.48E-11	1.19E-10
1274	ENSMUSG00000028461	Ccdc107	1.64	1.49E-11	1.19E-10
1275	ENSMUSG00000008892	Vdac3	1.25	1.49E-11	1.20E-10
1276	ENSMUSG00000031749	St3gal2	-2.26	1.50E-11	1.20E-10
1277	ENSMUSG00000049303	Syt12	-1.47	1.51E-11	1.21E-10
1278	ENSMUSG00000030688	Stard10	1.79	1.52E-11	1.22E-10
1279	ENSMUSG00000022484	Hoxc10	-2.17	1.57E-11	1.25E-10
1280	ENSMUSG00000023088	Abcc1	-1.39	1.57E-11	1.25E-10
1281	ENSMUSG00000032058	Ppp2r1b	-1.48	1.59E-11	1.27E-10
1282	ENSMUSG00000087232	Gm14764	2.11	1.59E-11	1.27E-10
1283	ENSMUSG00000025545	Clybl	1.51	1.60E-11	1.27E-10
1284	ENSMUSG00000024972	Lgals12	-1.73	1.62E-11	1.29E-10
1285	ENSMUSG00000058240	Cryzl1	1.45	1.63E-11	1.29E-10
1286	ENSMUSG00000032177	Pde4a	1.60	1.63E-11	1.30E-10
1287	ENSMUSG00000034220	Gpc1	-1.53	1.64E-11	1.30E-10
1288	ENSMUSG00000107314	Gm20488	-1.95	1.65E-11	1.31E-10
1289	ENSMUSG00000055401	Fbxo6	1.49	1.67E-11	1.32E-10
1290	ENSMUSG00000031633	Slc25a4	1.33	1.67E-11	1.32E-10
1291	ENSMUSG00000057672	Pkn1	1.43	1.67E-11	1.32E-10
1292	ENSMUSG00000040174	Alkbh3	1.35	1.68E-11	1.33E-10
1293	ENSMUSG00000074754	Smim26	1.63	1.68E-11	1.33E-10
1294	ENSMUSG00000024431	Nr3c1	-1.31	1.69E-11	1.34E-10
1295	ENSMUSG00000015013	Trappc2l	1.42	1.70E-11	1.34E-10
1296	ENSMUSG00000002279	Lmf1	1.35	1.70E-11	1.34E-10
1297	ENSMUSG00000030878	Cdr2	-2.04	1.71E-11	1.34E-10

1298	ENSMUSG00000036835	Psenen	1.39	1.72E-11	1.35E-10
1299	ENSMUSG00000024427	Spry4	-1.68	1.73E-11	1.36E-10
1300	ENSMUSG00000030861	Acadsb	-1.73	1.76E-11	1.38E-10
1301	ENSMUSG00000063457	Rps15	1.50	1.78E-11	1.40E-10
1302	ENSMUSG00000029781	Fkbp9	-1.50	1.82E-11	1.43E-10
1303	ENSMUSG00000033720	Sfxn5	1.50	1.83E-11	1.44E-10
1304	ENSMUSG00000029314	Gpat3	-1.94	1.85E-11	1.45E-10
1305	ENSMUSG00000052942	Glis3	-2.06	1.85E-11	1.45E-10
1306	ENSMUSG00000059070	Rpl18	1.36	1.88E-11	1.47E-10
1307	ENSMUSG00000041629	Fam104a	1.50	1.89E-11	1.48E-10
1308	ENSMUSG00000018339	Gpx3	1.33	1.90E-11	1.48E-10
1309	ENSMUSG00000087260	Lamtor5	1.48	1.90E-11	1.48E-10
1310	ENSMUSG00000027599	Armc1	1.38	1.95E-11	1.53E-10
1311	ENSMUSG00000030990	Pgap2	1.34	1.98E-11	1.54E-10
1312	ENSMUSG00000021771	Vdac2	1.26	2.00E-11	1.56E-10
1313	ENSMUSG00000031750	Il34	-2.22	2.00E-11	1.56E-10
1314	ENSMUSG00000026664	Phyh	1.32	2.01E-11	1.56E-10
1315	ENSMUSG00000099881	2810013P06Rik	1.45	2.04E-11	1.59E-10
1316	ENSMUSG00000026896	Ifih1	-1.93	2.06E-11	1.60E-10
1317	ENSMUSG00000020044	Timp3	-1.29	2.07E-11	1.61E-10
1318	ENSMUSG00000039105	Atp6v1g1	1.39	2.07E-11	1.61E-10
1319	ENSMUSG00000025151	Maged1	-1.42	2.07E-11	1.61E-10
1320	ENSMUSG00000021270	Hsp90aa1	1.36	2.09E-11	1.62E-10
1321	ENSMUSG00000028009	1700061I17Rik	1.85	2.11E-11	1.63E-10
1322	ENSMUSG00000039953	Clstn1	-1.59	2.12E-11	1.64E-10
1323	ENSMUSG00000070283	Ndufaf3	1.34	2.12E-11	1.64E-10
1324	ENSMUSG00000025453	Nnt	-2.23	2.13E-11	1.65E-10
1325	ENSMUSG00000113525	AC154550.1	-2.23	2.13E-11	1.65E-10
1326	ENSMUSG00000002265	Peg3	-2.33	2.15E-11	1.66E-10
1327	ENSMUSG00000000326	Comt	1.48	2.16E-11	1.66E-10
1328	ENSMUSG00000019710	Mrpl24	1.37	2.16E-11	1.66E-10
1329	ENSMUSG00000026399	Cd55	-2.00	2.16E-11	1.66E-10
1330	ENSMUSG00000030417	Pdcd5	1.48	2.16E-11	1.66E-10
1331	ENSMUSG00000031295	Phka2	-1.45	2.18E-11	1.68E-10
1332	ENSMUSG00000074178	Gm10638	2.13	2.21E-11	1.69E-10
1333	ENSMUSG00000024339	Tap2	-1.44	2.22E-11	1.71E-10
1334	ENSMUSG00000033707	Lrrc24	1.29	2.25E-11	1.73E-10
1335	ENSMUSG00000032216	Nedd4	-1.62	2.29E-11	1.75E-10
1336	ENSMUSG00000078185	Chml	-2.15	2.30E-11	1.76E-10
1337	ENSMUSG00000067924	Cxx1b	-1.38	2.31E-11	1.77E-10
1338	ENSMUSG00000018567	Gabarap	1.37	2.35E-11	1.79E-10
1339	ENSMUSG00000041842	Fhdc1	1.96	2.35E-11	1.79E-10
1340	ENSMUSG00000021760	Gpx8	1.43	2.35E-11	1.79E-10
1341	ENSMUSG00000028798	Eif3i	1.33	2.36E-11	1.80E-10
1342	ENSMUSG00000017692	Rhbdl3	-2.17	2.39E-11	1.82E-10
1343	ENSMUSG00000106847	Peg13	-1.86	2.40E-11	1.83E-10
1344	ENSMUSG00000000278	Scpep1	1.31	2.41E-11	1.84E-10
1345	ENSMUSG00000030979	Uros	1.40	2.42E-11	1.84E-10
1346	ENSMUSG00000024067	Dpy30	1.57	2.42E-11	1.84E-10
1347	ENSMUSG00000035283	Adrb1	1.80	2.43E-11	1.84E-10

1348	ENSMUSG00000113149	AC099934.3	1.53	2.44E-11	1.85E-10
1349	ENSMUSG00000026027	Stradb	1.40	2.47E-11	1.87E-10
1350	ENSMUSG00000100113	Gm28592	-1.96	2.49E-11	1.89E-10
1351	ENSMUSG00000036850	Mrpl41	1.39	2.54E-11	1.92E-10
1352	ENSMUSG00000025825	Iscu	1.36	2.57E-11	1.95E-10
1353	ENSMUSG00000020307	Cdc34	1.41	2.58E-11	1.95E-10
1354	ENSMUSG00000016382	Pls3	-1.32	2.63E-11	1.99E-10
1355	ENSMUSG00000030629	Zfand6	1.31	2.64E-11	1.99E-10
1356	ENSMUSG00000060519	Tor3a	-1.64	2.66E-11	2.01E-10
1357	ENSMUSG00000036372	Tmem258	1.53	2.66E-11	2.01E-10
1358	ENSMUSG00000019782	Rwdd1	1.38	2.66E-11	2.01E-10
1359	ENSMUSG00000035208	Slfn8	-1.89	2.67E-11	2.01E-10
1360	ENSMUSG00000036553	Sh3tc1	-1.41	2.72E-11	2.05E-10
1361	ENSMUSG00000033703	Fuk	-1.75	2.74E-11	2.06E-10
1362	ENSMUSG00000018565	Elp5	1.25	2.74E-11	2.06E-10
1363	ENSMUSG00000028465	Tln1	-1.37	2.79E-11	2.10E-10
1364	ENSMUSG00000087135	Gm16096	-2.27	2.80E-11	2.10E-10
1365	ENSMUSG00000085704	4921531C22Rik	1.62	2.80E-11	2.10E-10
1366	ENSMUSG00000068740	Celsr2	-2.25	2.83E-11	2.12E-10
1367	ENSMUSG00000025887	Casp12	-2.15	2.86E-11	2.14E-10
1368	ENSMUSG00000034875	Nudt19	1.36	2.87E-11	2.15E-10
1369	ENSMUSG00000023904	Hcfc1r1	1.42	2.90E-11	2.17E-10
1370	ENSMUSG00000005803	Sqrdl	-1.31	2.95E-11	2.21E-10
1371	ENSMUSG00000028369	Svep1	-2.25	2.96E-11	2.21E-10
1372	ENSMUSG00000024712	Rfk	1.26	3.00E-11	2.23E-10
1373	ENSMUSG00000042726	Trafd1	-1.59	3.02E-11	2.25E-10
1374	ENSMUSG00000005575	Ube2m	1.31	3.10E-11	2.31E-10
1375	ENSMUSG00000012428	Steap4	-3.24	3.12E-11	2.32E-10
1376	ENSMUSG00000103309	BC037039	-1.81	3.20E-11	2.38E-10
1377	ENSMUSG00000068391	Chrac1	1.36	3.20E-11	2.38E-10
1378	ENSMUSG00000026674	Ddr2	-2.36	3.20E-11	2.38E-10
1379	ENSMUSG00000023051	Tarbp2	1.44	3.22E-11	2.39E-10
1380	ENSMUSG00000029486	Mrpl1	1.44	3.24E-11	2.40E-10
1381	ENSMUSG00000035929	H2-Q4	-1.66	3.24E-11	2.40E-10
1382	ENSMUSG00000023020	Cox14	1.54	3.30E-11	2.44E-10
1383	ENSMUSG00000030045	Mrpl19	1.51	3.31E-11	2.45E-10
1384	ENSMUSG00000057531	Dtnbp1	1.32	3.32E-11	2.45E-10
1385	ENSMUSG00000030706	Mrpl48	1.39	3.38E-11	2.49E-10
1386	ENSMUSG00000034173	Zbed5	1.74	3.41E-11	2.51E-10
1387	ENSMUSG00000039713	Plekhg5	-2.00	3.42E-11	2.52E-10
1388	ENSMUSG00000035258	Abi3bp	-2.07	3.43E-11	2.53E-10
1389	ENSMUSG00000028367	Txn1	1.41	3.48E-11	2.56E-10
1390	ENSMUSG00000037936	Scarb1	-1.54	3.48E-11	2.56E-10
1391	ENSMUSG00000028919	Arhgef19	2.14	3.50E-11	2.58E-10
1392	ENSMUSG00000025068	Gsto1	1.22	3.52E-11	2.58E-10
1393	ENSMUSG00000022711	Pmm2	1.22	3.54E-11	2.60E-10
1394	ENSMUSG00000055128	Cgrrf1	1.23	3.55E-11	2.60E-10
1395	ENSMUSG00000046756	Mrps7	1.32	3.57E-11	2.62E-10
1396	ENSMUSG00000039831	Arhgap29	-1.73	3.60E-11	2.63E-10
1397	ENSMUSG00000048138	Dmrt2	-1.48	3.60E-11	2.63E-10

1398	ENSMUSG00000004951	Hspb1	1.50	3.60E-11	2.63E-10
1399	ENSMUSG00000035983	Gm7008	-1.75	3.60E-11	2.63E-10
1400	ENSMUSG00000064068	Mtx1	1.30	3.61E-11	2.64E-10
1401	ENSMUSG00000054200	Ffar4	1.68	3.61E-11	2.64E-10
1402	ENSMUSG00000058586	Serhl	1.25	3.62E-11	2.64E-10
1403	ENSMUSG00000025381	Cnpy2	1.39	3.66E-11	2.66E-10
1404	ENSMUSG00000097020	Gm26613	1.37	3.67E-11	2.67E-10
1405	ENSMUSG00000034108	Ccs	1.31	3.69E-11	2.69E-10
1406	ENSMUSG00000109378	U2af1l4	1.45	3.69E-11	2.69E-10
1407	ENSMUSG00000089665	Fcor	1.38	3.70E-11	2.69E-10
1408	ENSMUSG00000049517	Rps23	1.43	3.72E-11	2.70E-10
1409	ENSMUSG00000026421	Csrp1	-1.54	3.72E-11	2.70E-10
1410	ENSMUSG00000026511	Srp9	1.31	3.77E-11	2.74E-10
1411	ENSMUSG00000039693	Msantd3	1.66	3.88E-11	2.81E-10
1412	ENSMUSG00000028145	Them4	1.63	3.89E-11	2.82E-10
1413	ENSMUSG00000027893	Ahcyl1	1.29	3.90E-11	2.82E-10
1414	ENSMUSG00000031661	Nkd1	-2.14	3.94E-11	2.85E-10
1415	ENSMUSG00000027217	Tspan18	1.26	3.94E-11	2.85E-10
1416	ENSMUSG00000010663	Fads1	-2.68	3.97E-11	2.87E-10
1417	ENSMUSG00000008859	Rala	1.34	4.01E-11	2.89E-10
1418	ENSMUSG00000109482	Gm4756	1.65	4.08E-11	2.94E-10
1419	ENSMUSG00000092381	Gm20512	1.37	4.14E-11	2.98E-10
1420	ENSMUSG00000097417	Gm26669	-1.62	4.16E-11	3.00E-10
1421	ENSMUSG00000031432	Prps1	-1.92	4.17E-11	3.00E-10
1422	ENSMUSG00000067365	Tmem128	1.70	4.18E-11	3.00E-10
1423	ENSMUSG00000094447	9430069I07Rik	-2.00	4.20E-11	3.02E-10
1424	ENSMUSG00000003541	Ier3	-1.51	4.22E-11	3.03E-10
1425	ENSMUSG00000044221	Grsf1	1.17	4.31E-11	3.09E-10
1426	ENSMUSG00000024008	Cpne5	2.54	4.39E-11	3.15E-10
1427	ENSMUSG00000019804	Snx3	1.19	4.41E-11	3.16E-10
1428	ENSMUSG00000031119	Gpc4	-1.48	4.48E-11	3.21E-10
1429	ENSMUSG00000078570	1110065P20Rik	1.38	4.49E-11	3.21E-10
1430	ENSMUSG00000068184	Ndufaf2	1.69	4.52E-11	3.23E-10
1431	ENSMUSG00000032491	Nradd	1.96	4.54E-11	3.25E-10
1432	ENSMUSG00000052712	BC004004	1.24	4.57E-11	3.26E-10
1433	ENSMUSG00000047721	Bola2	1.75	4.61E-11	3.29E-10
1434	ENSMUSG00000015671	Psma2	1.30	4.72E-11	3.37E-10
1435	ENSMUSG00000034714	Ttyh2	-1.58	4.79E-11	3.41E-10
1436	ENSMUSG00000021708	Rasgrf2	-1.85	4.80E-11	3.42E-10
1437	ENSMUSG00000060240	Cend1	2.18	4.82E-11	3.43E-10
1438	ENSMUSG00000078765	U2af1l4	1.42	4.84E-11	3.44E-10
1439	ENSMUSG00000022024	Sugt1	1.59	4.86E-11	3.45E-10
1440	ENSMUSG00000030357	Fkbp4	1.22	4.90E-11	3.48E-10
1441	ENSMUSG00000067925	Cxx1a	-1.27	4.94E-11	3.50E-10
1442	ENSMUSG00000022594	Lynx1	1.82	4.95E-11	3.51E-10
1443	ENSMUSG00000003153	Slc2a3	-2.05	5.00E-11	3.54E-10
1444	ENSMUSG00000026463	Atp2b4	-2.23	5.07E-11	3.59E-10
1445	ENSMUSG00000027803	Wwtr1	-1.41	5.13E-11	3.63E-10
1446	ENSMUSG00000020053	Igf1	-3.01	5.15E-11	3.64E-10
1447	ENSMUSG00000026150	Mff	1.22	5.16E-11	3.65E-10

1448	ENSMUSG00000042709	Atpaf2	1.19	5.25E-11	3.71E-10
1449	ENSMUSG00000030591	Psmd8	1.25	5.28E-11	3.72E-10
1450	ENSMUSG00000057335	Cep170	-1.71	5.32E-11	3.75E-10
1451	ENSMUSG00000003348	Mob3a	-1.71	5.44E-11	3.83E-10
1452	ENSMUSG00000012519	Mlkl	-2.06	5.51E-11	3.88E-10
1453	ENSMUSG00000035783	Acta2	-2.43	5.51E-11	3.88E-10
1454	ENSMUSG00000031352	Hccs	1.26	5.54E-11	3.89E-10
1455	ENSMUSG00000026820	Ptges2	1.24	5.74E-11	4.03E-10
1456	ENSMUSG00000104574	Gm42836	-1.98	5.75E-11	4.04E-10
1457	ENSMUSG00000025647	Shisa5	-1.28	5.96E-11	4.18E-10
1458	ENSMUSG00000109857	Gm45563	1.95	6.02E-11	4.22E-10
1459	ENSMUSG00000026492	Tfb2m	1.22	6.02E-11	4.22E-10
1460	ENSMUSG00000110195	Pde2a	-1.60	6.02E-11	4.22E-10
1461	ENSMUSG00000027371	Fahd2a	1.27	6.03E-11	4.22E-10
1462	ENSMUSG00000081512	Gm15821	-1.45	6.05E-11	4.23E-10
1463	ENSMUSG00000061983	Rps12	1.65	6.11E-11	4.27E-10
1464	ENSMUSG00000023067	Cdkn1a	1.76	6.12E-11	4.27E-10
1465	ENSMUSG00000079013	Serpina3j	-1.93	6.17E-11	4.31E-10
1466	ENSMUSG00000078651	Aoc2	-1.92	6.19E-11	4.32E-10
1467	ENSMUSG00000002767	Mrpl2	1.32	6.20E-11	4.32E-10
1468	ENSMUSG00000025648	Pfkfb4	-2.04	6.20E-11	4.32E-10
1469	ENSMUSG00000038648	Creb3l2	-1.94	6.23E-11	4.34E-10
1470	ENSMUSG00000010097	Nxf1	1.32	6.24E-11	4.34E-10
1471	ENSMUSG00000017868	Sgk2	2.03	6.34E-11	4.40E-10
1472	ENSMUSG00000028036	Ptgfr	-1.95	6.39E-11	4.44E-10
1473	ENSMUSG00000058546	Rpl23a	1.43	6.40E-11	4.44E-10
1474	ENSMUSG00000056629	Fkbp2	1.27	6.40E-11	4.44E-10
1475	ENSMUSG00000061360	Phf5a	1.44	6.44E-11	4.46E-10
1476	ENSMUSG00000039501	Znfx1	-1.56	6.47E-11	4.48E-10
1477	ENSMUSG00000035107	Dcbld2	-1.86	6.56E-11	4.54E-10
1478	ENSMUSG00000014606	Slc25a11	1.16	6.56E-11	4.54E-10
1479	ENSMUSG00000053317	Sec61b	1.54	6.57E-11	4.54E-10
1480	ENSMUSG00000020621	Rdh14	1.30	6.59E-11	4.55E-10
1481	ENSMUSG00000051236	Msrb3	-1.49	6.68E-11	4.61E-10
1482	ENSMUSG00000022856	Tmem41a	1.49	6.72E-11	4.64E-10
1483	ENSMUSG00000059895	Ptp4a3	1.39	6.89E-11	4.75E-10
1484	ENSMUSG00000022261	Sdc2	-1.58	6.96E-11	4.80E-10
1485	ENSMUSG00000022210	Dhrs4	1.26	7.06E-11	4.86E-10
1486	ENSMUSG00000025499	Hras	1.29	7.10E-11	4.88E-10
1487	ENSMUSG00000110136	Gm45785	1.76	7.14E-11	4.91E-10
1488	ENSMUSG00000023944	Hsp90ab1	1.27	7.15E-11	4.92E-10
1489	ENSMUSG00000053819	Camk2d	-1.54	7.19E-11	4.94E-10
1490	ENSMUSG00000079444	Gm21981	1.29	7.30E-11	5.01E-10
1491	ENSMUSG00000033475	Tomm6	1.30	7.35E-11	5.04E-10
1492	ENSMUSG00000024235	Map3k8	-1.86	7.44E-11	5.10E-10
1493	ENSMUSG00000031903	Pla2g15	-1.52	7.46E-11	5.11E-10
1494	ENSMUSG00000032802	Srxn1	1.18	7.47E-11	5.11E-10
1495	ENSMUSG00000039163	Cmc1	1.35	7.54E-11	5.16E-10
1496	ENSMUSG00000038886	Man2a2	-1.30	7.66E-11	5.23E-10
1497	ENSMUSG00000061559	Wdr61	1.31	7.68E-11	5.24E-10

1498	ENSMUSG00000051851	Cxx1c	-2.22	7.84E-11	5.35E-10
1499	ENSMUSG00000027086	Fastkd1	1.48	7.86E-11	5.36E-10
1500	ENSMUSG00000027177	Hipk3	-1.55	8.11E-11	5.53E-10
1501	ENSMUSG00000025962	Fastkd2	1.34	8.33E-11	5.68E-10
1502	ENSMUSG00000001052	Sec24b	-1.85	8.48E-11	5.77E-10
1503	ENSMUSG00000071335	Mfsd4b3	1.86	8.52E-11	5.79E-10
1504	ENSMUSG00000038880	Mrps34	1.25	8.57E-11	5.83E-10
1505	ENSMUSG00000001983	Taco1	1.79	8.64E-11	5.87E-10
1506	ENSMUSG00000027634	Ndrg3	1.19	8.72E-11	5.92E-10
1507	ENSMUSG00000022203	Efs	-1.71	8.72E-11	5.92E-10
1508	ENSMUSG00000021102	Glrx5	1.23	8.74E-11	5.92E-10
1509	ENSMUSG00000024346	Pfdn1	1.41	8.82E-11	5.97E-10
1510	ENSMUSG00000013593	Ndufs2	1.29	8.83E-11	5.97E-10
1511	ENSMUSG00000027639	Samhd1	-1.63	8.83E-11	5.97E-10
1512	ENSMUSG00000073838	Tufm	1.30	8.84E-11	5.98E-10
1513	ENSMUSG00000066442	Mthfs	1.32	8.89E-11	6.01E-10
1514	ENSMUSG00000029722	Agfg2	-1.64	8.91E-11	6.02E-10
1515	ENSMUSG00000021559	Dapk1	-2.09	8.99E-11	6.07E-10
1516	ENSMUSG00000025220	Mgea5	-1.46	9.02E-11	6.08E-10
1517	ENSMUSG00000038900	Rpl12	1.48	9.06E-11	6.11E-10
1518	ENSMUSG00000021039	Snw1	1.30	9.11E-11	6.14E-10
1519	ENSMUSG00000111840	AC159809.2	-1.95	9.41E-11	6.33E-10
1520	ENSMUSG00000024338	Psmb8	-2.20	9.49E-11	6.39E-10
1521	ENSMUSG00000003316	Glg1	-1.46	9.52E-11	6.40E-10
1522	ENSMUSG00000027131	Emc4	1.23	9.53E-11	6.40E-10
1523	ENSMUSG00000028444	Cntfr	1.96	9.65E-11	6.48E-10
1524	ENSMUSG00000097889	Gm26541	-1.70	9.70E-11	6.51E-10
1525	ENSMUSG00000093904	Tomm20	1.14	9.75E-11	6.54E-10
1526	ENSMUSG00000047878	A4galt	-2.08	9.75E-11	6.54E-10
1527	ENSMUSG00000054404	Slfn5	-1.48	9.76E-11	6.54E-10
1528	ENSMUSG00000030088	Aldh1l1	-1.59	9.79E-11	6.55E-10
1529	ENSMUSG00000047497	Adamts12	-2.07	9.80E-11	6.56E-10
1530	ENSMUSG00000027221	Chst1	-2.75	9.97E-11	6.66E-10
1531	ENSMUSG00000059040	Eno1b	1.71	1.01E-10	6.76E-10
1532	ENSMUSG00000028015	Ctso	-1.91	1.01E-10	6.77E-10
1533	ENSMUSG00000041957	Pkp2	-1.75	1.02E-10	6.79E-10
1534	ENSMUSG00000073702	Rpl31	1.46	1.02E-10	6.79E-10
1535	ENSMUSG00000097318	1700007L15Rik	1.82	1.02E-10	6.79E-10
1536	ENSMUSG00000096727	Psmb9	-1.77	1.03E-10	6.85E-10
1537	ENSMUSG00000041481	Serpina3g	-2.04	1.05E-10	6.98E-10
1538	ENSMUSG00000036073	Galt	1.41	1.06E-10	7.02E-10
1539	ENSMUSG00000033732	Sf3b3	-1.54	1.06E-10	7.07E-10
1540	ENSMUSG00000004667	Polr2e	1.17	1.09E-10	7.22E-10
1541	ENSMUSG00000038539	Atf5	2.09	1.09E-10	7.22E-10
1542	ENSMUSG00000022338	Eny2	1.39	1.10E-10	7.29E-10
1543	ENSMUSG00000031158	Timm17b	1.22	1.10E-10	7.31E-10
1544	ENSMUSG00000028743	Akr7a5	1.16	1.11E-10	7.38E-10
1545	ENSMUSG00000032348	Gsta4	1.31	1.12E-10	7.39E-10
1546	ENSMUSG00000039853	Trim14	-1.82	1.12E-10	7.39E-10
1547	ENSMUSG00000005566	Trim28	-1.53	1.14E-10	7.52E-10

1548	ENSMUSG00000102828	Gm38182	-1.73	1.15E-10	7.58E-10
1549	ENSMUSG00000020234	4930404N11Rik	-2.11	1.15E-10	7.58E-10
1550	ENSMUSG00000035530	Eif1	1.25	1.16E-10	7.63E-10
1551	ENSMUSG00000018846	Pank3	-1.71	1.16E-10	7.67E-10
1552	ENSMUSG00000020268	Lyrm7	1.55	1.18E-10	7.74E-10
1553	ENSMUSG00000023791	Pigx	1.67	1.20E-10	7.87E-10
1554	ENSMUSG00000064210	Ano6	-1.54	1.20E-10	7.91E-10
1555	ENSMUSG00000030029	Lrig1	-1.73	1.21E-10	7.98E-10
1556	ENSMUSG00000024426	Atat1	1.31	1.22E-10	7.99E-10
1557	ENSMUSG00000069729	Arid1b	-1.36	1.22E-10	8.01E-10
1558	ENSMUSG00000064254	Ethe1	1.76	1.23E-10	8.06E-10
1559	ENSMUSG00000079012	Serpina3m	-2.31	1.25E-10	8.20E-10
1560	ENSMUSG00000111339	AC139579.1	1.34	1.26E-10	8.26E-10
1561	ENSMUSG00000046070	Igfals	-2.23	1.26E-10	8.26E-10
1562	ENSMUSG00000020538	Srebf1	-1.38	1.27E-10	8.33E-10
1563	ENSMUSG00000056492	Adgrf5	-1.32	1.29E-10	8.41E-10
1564	ENSMUSG00000090964	Gm17206	1.40	1.29E-10	8.41E-10
1565	ENSMUSG00000089675	Ugt1a8	-2.17	1.29E-10	8.42E-10
1566	ENSMUSG00000030653	Gm45837	-1.48	1.30E-10	8.51E-10
1567	ENSMUSG00000016933	Plcg1	-1.63	1.31E-10	8.53E-10
1568	ENSMUSG00000029716	Tfr2	1.60	1.32E-10	8.59E-10
1569	ENSMUSG00000105692	Gm43737	1.32	1.32E-10	8.59E-10
1570	ENSMUSG00000052146	Rps10	1.41	1.33E-10	8.67E-10
1571	ENSMUSG00000090175	Ugt1a9	-2.17	1.34E-10	8.69E-10
1572	ENSMUSG00000026796	Fam129b	-1.84	1.35E-10	8.76E-10
1573	ENSMUSG00000089943	Ugt1a5	-2.17	1.35E-10	8.80E-10
1574	ENSMUSG00000087132	A930001C03Rik	-1.43	1.36E-10	8.86E-10
1575	ENSMUSG00000037361	Sf3b6	1.31	1.37E-10	8.87E-10
1576	ENSMUSG00000027792	Bche	-1.66	1.37E-10	8.91E-10
1577	ENSMUSG00000090165	Ugt1a10	-2.17	1.38E-10	8.91E-10
1578	ENSMUSG00000090171	Ugt1a2	-2.17	1.38E-10	8.91E-10
1579	ENSMUSG00000089960	Ugt1a1	-2.17	1.38E-10	8.91E-10
1580	ENSMUSG00000008140	Emc10	1.10	1.38E-10	8.93E-10
1581	ENSMUSG00000031402	Mpp1	-1.53	1.39E-10	8.96E-10
1582	ENSMUSG00000087406	E130215H24Rik	1.79	1.42E-10	9.16E-10
1583	ENSMUSG00000026456	Cyb5r1	1.36	1.42E-10	9.17E-10
1584	ENSMUSG00000040356	Skiv2l	-1.14	1.42E-10	9.19E-10
1585	ENSMUSG00000042429	Adora1	-1.42	1.44E-10	9.28E-10
1586	ENSMUSG00000025934	Gsta3	-2.32	1.44E-10	9.30E-10
1587	ENSMUSG00000029551	Psmg3	1.60	1.47E-10	9.45E-10
1588	ENSMUSG00000064181	Rab3ip	1.45	1.47E-10	9.45E-10
1589	ENSMUSG00000084880	Tomm6os	1.27	1.48E-10	9.50E-10
1590	ENSMUSG00000068747	Sort1	-1.59	1.48E-10	9.52E-10
1591	ENSMUSG00000051671	Coa6	1.52	1.49E-10	9.57E-10
1592	ENSMUSG00000073910	Mob3b	-1.89	1.49E-10	9.57E-10
1593	ENSMUSG00000026034	Clk1	-1.39	1.50E-10	9.63E-10
1594	ENSMUSG00000025465	Echs1	1.24	1.51E-10	9.66E-10
1595	ENSMUSG00000030603	Psmc4	1.23	1.51E-10	9.66E-10
1596	ENSMUSG00000021619	Atg10	1.32	1.51E-10	9.69E-10
1597	ENSMUSG00000022519	Srl	1.50	1.52E-10	9.74E-10

1598	ENSMUSG00000019179	Mdh2	1.33	1.54E-10	9.88E-10
1599	ENSMUSG00000037916	Ndufv1	1.38	1.55E-10	9.91E-10
1600	ENSMUSG00000028165	Cisd2	1.24	1.55E-10	9.91E-10
1601	ENSMUSG00000069223	4930451E10Rik	1.56	1.58E-10	1.01E-09
1602	ENSMUSG00000039646	Vasn	-2.26	1.58E-10	1.01E-09
1603	ENSMUSG00000022197	Pdzd2	-1.67	1.61E-10	1.03E-09
1604	ENSMUSG00000038195	Rilp	1.53	1.62E-10	1.03E-09
1605	ENSMUSG00000061780	Cfd	-2.69	1.62E-10	1.03E-09
1606	ENSMUSG00000062169	Cnih4	1.30	1.62E-10	1.03E-09
1607	ENSMUSG00000022889	Mrpl39	1.34	1.63E-10	1.03E-09
1608	ENSMUSG00000091996	BC049352	1.80	1.64E-10	1.04E-09
1609	ENSMUSG00000092220	Gm20528	-2.08	1.64E-10	1.04E-09
1610	ENSMUSG00000025324	Atp10a	-2.04	1.64E-10	1.04E-09
1611	ENSMUSG00000020186	Csrp2	1.61	1.66E-10	1.05E-09
1612	ENSMUSG00000028751	Pla2g2e	1.71	1.66E-10	1.05E-09
1613	ENSMUSG00000022635	Zcrb1	1.28	1.67E-10	1.06E-09
1614	ENSMUSG00000045658	Pid1	-1.95	1.67E-10	1.06E-09
1615	ENSMUSG00000084783	Gm15419	-1.98	1.69E-10	1.07E-09
1616	ENSMUSG00000019210	Atp6v1e1	1.23	1.69E-10	1.07E-09
1617	ENSMUSG00000044533	Rps2	1.24	1.71E-10	1.08E-09
1618	ENSMUSG00000022500	Litaf	-1.28	1.73E-10	1.09E-09
1619	ENSMUSG00000020744	Slc25a19	1.31	1.73E-10	1.09E-09
1620	ENSMUSG00000018169	Mfng	-2.25	1.73E-10	1.09E-09
1621	ENSMUSG00000027559	Car3	-2.98	1.74E-10	1.10E-09
1622	ENSMUSG00000042380	Smim12	1.19	1.74E-10	1.10E-09
1623	ENSMUSG00000024414	Mrpl27	1.27	1.75E-10	1.10E-09
1624	ENSMUSG00000021124	Vti1b	1.15	1.75E-10	1.11E-09
1625	ENSMUSG00000018900	Slc22a5	1.27	1.77E-10	1.11E-09
1626	ENSMUSG00000020774	Aspa	1.25	1.78E-10	1.12E-09
1627	ENSMUSG00000073422	H2-Ke6	1.32	1.80E-10	1.13E-09
1628	ENSMUSG00000109237	Gm44642	1.73	1.82E-10	1.14E-09
1629	ENSMUSG00000020485	Supt4a	1.46	1.82E-10	1.14E-09
1630	ENSMUSG00000030842	Lamtor1	1.18	1.83E-10	1.15E-09
1631	ENSMUSG00000069833	Ahnak	-1.30	1.86E-10	1.16E-09
1632	ENSMUSG00000098754	Gm44637	-1.56	1.86E-10	1.17E-09
1633	ENSMUSG00000026471	Mr1	-2.01	1.86E-10	1.17E-09
1634	ENSMUSG00000041193	Pla2g5	-2.02	1.88E-10	1.18E-09
1635	ENSMUSG00000032633	Flcn	1.29	1.89E-10	1.18E-09
1636	ENSMUSG00000022906	Parp9	-1.49	1.90E-10	1.19E-09
1637	ENSMUSG00000108555	Gm18310	1.79	1.90E-10	1.19E-09
1638	ENSMUSG00000016520	Lnx2	-1.34	1.91E-10	1.19E-09
1639	ENSMUSG00000036398	Ppp1r11	1.48	1.91E-10	1.19E-09
1640	ENSMUSG00000076431	Sox4	-2.05	1.92E-10	1.20E-09
1641	ENSMUSG00000030711	Sult1a1	-2.13	1.95E-10	1.21E-09
1642	ENSMUSG00000078784	1810022K09Rik	1.37	1.96E-10	1.22E-09
1643	ENSMUSG00000084960	B430010I23Rik	1.37	1.96E-10	1.22E-09
1644	ENSMUSG00000050373	Snx21	1.27	1.99E-10	1.24E-09
1645	ENSMUSG00000028795	Ccdc28b	1.60	1.99E-10	1.24E-09
1646	ENSMUSG00000010609	Psen2	-1.52	2.03E-10	1.26E-09
1647	ENSMUSG00000037072	Selenof	1.18	2.04E-10	1.27E-09

1648	ENSMUSG00000005198	Polr2a	-1.34	2.07E-10	1.28E-09
1649	ENSMUSG00000045868	Gvin1	-2.15	2.13E-10	1.32E-09
1650	ENSMUSG00000028622	Mrpl37	1.10	2.13E-10	1.32E-09
1651	ENSMUSG00000027170	Eif3m	1.20	2.13E-10	1.32E-09
1652	ENSMUSG00000028684	Urod	1.20	2.15E-10	1.33E-09
1653	ENSMUSG00000000938	Hoxa10	-1.86	2.16E-10	1.33E-09
1654	ENSMUSG00000038227	Hoxa9	-1.91	2.16E-10	1.34E-09
1655	ENSMUSG00000056214	Pard6g	-1.86	2.18E-10	1.35E-09
1656	ENSMUSG00000002064	Sdf2	1.23	2.20E-10	1.36E-09
1657	ENSMUSG00000090958	Lrrc32	-1.97	2.25E-10	1.39E-09
1658	ENSMUSG00000079017	Ifi27l2a	-1.56	2.31E-10	1.43E-09
1659	ENSMUSG00000027624	Epb41l1	-2.04	2.34E-10	1.44E-09
1660	ENSMUSG00000094910	D430019H16Rik	-1.84	2.34E-10	1.44E-09
1661	ENSMUSG00000022544	Eef2kmt	1.26	2.35E-10	1.45E-09
1662	ENSMUSG00000030770	Parva	-1.32	2.35E-10	1.45E-09
1663	ENSMUSG00000039001	Rps21	1.71	2.35E-10	1.45E-09
1664	ENSMUSG00000106178	Gm42987	-1.78	2.43E-10	1.49E-09
1665	ENSMUSG00000025981	Coq10b	1.24	2.45E-10	1.51E-09
1666	ENSMUSG00000030291	Med21	1.31	2.49E-10	1.53E-09
1667	ENSMUSG00000005882	Uqcc1	1.16	2.51E-10	1.54E-09
1668	ENSMUSG00000022199	Slc22a17	-1.79	2.53E-10	1.55E-09
1669	ENSMUSG00000053565	Eif3k	1.17	2.55E-10	1.56E-09
1670	ENSMUSG00000062797	Hikeshi	1.42	2.55E-10	1.56E-09
1671	ENSMUSG00000030879	Mrpl17	1.15	2.58E-10	1.58E-09
1672	ENSMUSG00000025969	Nrp2	1.62	2.58E-10	1.58E-09
1673	ENSMUSG00000031431	Tsc22d3	-1.36	2.59E-10	1.58E-09
1674	ENSMUSG00000026860	Sh3glb2	-1.14	2.62E-10	1.60E-09
1675	ENSMUSG00000001506	Col1a1	-2.02	2.63E-10	1.60E-09
1676	ENSMUSG00000024201	Kdm4b	-1.30	2.63E-10	1.60E-09
1677	ENSMUSG00000010914	Pdhx	1.20	2.64E-10	1.61E-09
1678	ENSMUSG00000114036	AC124426.5	1.42	2.65E-10	1.61E-09
1679	ENSMUSG00000041733	Coq5	1.10	2.65E-10	1.61E-09
1680	ENSMUSG00000068686	Cd59b	1.33	2.67E-10	1.63E-09
1681	ENSMUSG00000003423	Pih1d1	1.37	2.72E-10	1.65E-09
1682	ENSMUSG00000034640	Tiparp	-1.15	2.76E-10	1.68E-09
1683	ENSMUSG00000027835	Pdcd10	1.34	2.78E-10	1.69E-09
1684	ENSMUSG00000109745	1700018G05Rik	1.87	2.78E-10	1.69E-09
1685	ENSMUSG00000022982	Sod1	1.22	2.79E-10	1.69E-09
1686	ENSMUSG00000008683	Rps15a	1.60	2.79E-10	1.69E-09
1687	ENSMUSG00000023021	Cers5	-1.43	2.79E-10	1.69E-09
1688	ENSMUSG00000018102	Hist1h2bc	1.32	2.79E-10	1.69E-09
1689	ENSMUSG00000004562	Arhgef40	-1.58	2.82E-10	1.70E-09
1690	ENSMUSG00000015672	Mrpl32	1.30	2.83E-10	1.71E-09
1691	ENSMUSG00000020321	Mdh1	1.20	2.86E-10	1.73E-09
1692	ENSMUSG00000090145	Ugt1a6b	-2.27	2.86E-10	1.73E-09
1693	ENSMUSG00000020520	Galnt10	-1.59	2.90E-10	1.75E-09
1694	ENSMUSG00000060678	Hist1h4c	1.84	2.93E-10	1.77E-09
1695	ENSMUSG00000027447	Cst3	1.29	2.97E-10	1.79E-09
1696	ENSMUSG00000021411	Pxdc1	-1.77	2.99E-10	1.81E-09
1697	ENSMUSG00000101438	Gm19412	-1.28	3.02E-10	1.82E-09

1698	ENSMUSG00000008822	Acyp1	1.71	3.02E-10	1.82E-09
1699	ENSMUSG00000040824	Snrpd2	1.38	3.06E-10	1.84E-09
1700	ENSMUSG00000060586	H2-Eb1	2.07	3.06E-10	1.84E-09
1701	ENSMUSG00000020303	Stc2	-2.03	3.09E-10	1.86E-09
1702	ENSMUSG00000056515	Rab31	-1.72	3.11E-10	1.87E-09
1703	ENSMUSG00000041632	Mrps27	1.14	3.14E-10	1.88E-09
1704	ENSMUSG00000079197	Psme2	1.20	3.16E-10	1.89E-09
1705	ENSMUSG00000026385	Dbi	1.39	3.22E-10	1.93E-09
1706	ENSMUSG00000020181	Nav3	-1.89	3.28E-10	1.97E-09
1707	ENSMUSG00000086432	B430119L08Rik	-1.74	3.33E-10	2.00E-09
1708	ENSMUSG00000002768	Mea1	1.19	3.37E-10	2.02E-09
1709	ENSMUSG00000042043	Tbca	1.37	3.37E-10	2.02E-09
1710	ENSMUSG00000027082	Tfpi	-2.05	3.39E-10	2.03E-09
1711	ENSMUSG00000024242	Map4k3	-1.48	3.40E-10	2.03E-09
1712	ENSMUSG00000034390	Cmip	-1.93	3.40E-10	2.03E-09
1713	ENSMUSG00000037321	Tap1	-1.44	3.40E-10	2.03E-09
1714	ENSMUSG00000024014	Pim1	2.14	3.45E-10	2.06E-09
1715	ENSMUSG00000044927	H1fx	-1.75	3.46E-10	2.06E-09
1716	ENSMUSG00000015243	Abca1	-1.62	3.46E-10	2.06E-09
1717	ENSMUSG00000021196	Pfkp	1.27	3.48E-10	2.07E-09
1718	ENSMUSG00000027823	Gmps	1.12	3.52E-10	2.10E-09
1719	ENSMUSG00000024902	Mrpl11	1.42	3.55E-10	2.11E-09
1720	ENSMUSG00000079505	Gm11131	-1.75	3.55E-10	2.11E-09
1721	ENSMUSG00000038991	Txndc5	-1.24	3.58E-10	2.13E-09
1722	ENSMUSG00000021756	Il6st	-1.70	3.58E-10	2.13E-09
1723	ENSMUSG00000002280	Narfl	1.23	3.61E-10	2.14E-09
1724	ENSMUSG00000022181	C6	-1.72	3.63E-10	2.15E-09
1725	ENSMUSG00000029826	Zc3hav1	-1.65	3.68E-10	2.18E-09
1726	ENSMUSG00000003526	Prodh	-1.36	3.71E-10	2.20E-09
1727	ENSMUSG00000000959	Oxa1l	1.18	3.71E-10	2.20E-09
1728	ENSMUSG00000021830	Txndc16	-1.83	3.71E-10	2.20E-09
1729	ENSMUSG00000004268	Emg1	1.17	3.73E-10	2.20E-09
1730	ENSMUSG00000018761	Mpdu1	1.12	3.82E-10	2.26E-09
1731	ENSMUSG00000038803	Ost4	1.20	3.83E-10	2.27E-09
1732	ENSMUSG00000031835	Mbtps1	-1.20	3.89E-10	2.29E-09
1733	ENSMUSG00000001334	Fndc5	1.75	3.97E-10	2.34E-09
1734	ENSMUSG00000005625	Psmd4	1.13	3.98E-10	2.35E-09
1735	ENSMUSG00000032534	Cep63	1.25	4.01E-10	2.37E-09
1736	ENSMUSG00000002804	Nudt14	1.64	4.02E-10	2.37E-09
1737	ENSMUSG00000045107	Saysd1	1.57	4.06E-10	2.39E-09
1738	ENSMUSG00000019768	Esr1	-2.06	4.06E-10	2.39E-09
1739	ENSMUSG00000022231	Sema5a	-2.18	4.09E-10	2.41E-09
1740	ENSMUSG00000094724	Rnaset2b	1.41	4.13E-10	2.43E-09
1741	ENSMUSG00000028469	Npr2	-1.47	4.15E-10	2.44E-09
1742	ENSMUSG00000024807	Syvn1	-1.15	4.18E-10	2.46E-09
1743	ENSMUSG00000017754	Pltp	-1.14	4.19E-10	2.46E-09
1744	ENSMUSG00000015085	Entpd2	-1.47	4.20E-10	2.46E-09
1745	ENSMUSG00000020150	Gamt	-1.62	4.21E-10	2.47E-09
1746	ENSMUSG00000031239	Itm2a	-1.95	4.30E-10	2.52E-09
1747	ENSMUSG00000027546	Atp9a	-1.24	4.32E-10	2.53E-09

1748	ENSMUSG00000031879	Fam96b	1.35	4.32E-10	2.53E-09
1749	ENSMUSG00000096199	Ptrhd1	1.44	4.35E-10	2.54E-09
1750	ENSMUSG00000027374	Mrps5	1.22	4.35E-10	2.54E-09
1751	ENSMUSG00000050737	Ptges	-2.08	4.40E-10	2.57E-09
1752	ENSMUSG00000030516	Tjp1	-1.63	4.43E-10	2.58E-09
1753	ENSMUSG00000036817	Sun1	-1.40	4.48E-10	2.61E-09
1754	ENSMUSG00000027709	Mccc1	-1.16	4.51E-10	2.63E-09
1755	ENSMUSG00000022971	Ifnar2	1.08	4.53E-10	2.64E-09
1756	ENSMUSG00000020949	Fkbp3	1.38	4.55E-10	2.65E-09
1757	ENSMUSG00000002885	Adgre5	-1.47	4.55E-10	2.65E-09
1758	ENSMUSG00000037563	Rps16	1.42	4.65E-10	2.71E-09
1759	ENSMUSG00000036782	Klhl13	-1.84	4.73E-10	2.75E-09
1760	ENSMUSG00000034903	Cobll1	-1.30	4.77E-10	2.77E-09
1761	ENSMUSG00000028837	Psmb2	1.13	4.80E-10	2.79E-09
1762	ENSMUSG00000003868	Ruvbl2	1.13	4.81E-10	2.79E-09
1763	ENSMUSG00000038683	Pak1ip1	1.27	4.82E-10	2.79E-09
1764	ENSMUSG00000039660	Spout1	1.29	4.87E-10	2.82E-09
1765	ENSMUSG00000034158	Lrrc58	-1.44	4.89E-10	2.83E-09
1766	ENSMUSG00000057177	Gsk3a	1.12	4.92E-10	2.85E-09
1767	ENSMUSG00000107838	Gm45769	1.41	4.94E-10	2.86E-09
1768	ENSMUSG00000033565	Rbfox2	-1.28	4.96E-10	2.87E-09
1769	ENSMUSG00000049106	Dcaf5	-1.35	4.99E-10	2.88E-09
1770	ENSMUSG00000006676	Usp19	-1.11	5.01E-10	2.90E-09
1771	ENSMUSG00000025815	Dhtkd1	-1.80	5.12E-10	2.96E-09
1772	ENSMUSG00000030611	Mrps11	1.39	5.13E-10	2.96E-09
1773	ENSMUSG00000014418	Hps5	-1.34	5.19E-10	2.99E-09
1774	ENSMUSG00000030432	Rpl28	1.36	5.25E-10	3.03E-09
1775	ENSMUSG00000014551	Mrps25	1.14	5.26E-10	3.03E-09
1776	ENSMUSG00000069094	Pde7a	-1.53	5.27E-10	3.03E-09
1777	ENSMUSG00000032280	Tle3	-1.41	5.29E-10	3.04E-09
1778	ENSMUSG00000036570	Fxyd1	1.20	5.30E-10	3.05E-09
1779	ENSMUSG00000043122	A530016L24Rik	-1.70	5.30E-10	3.05E-09
1780	ENSMUSG00000042961	Egflam	-1.85	5.31E-10	3.05E-09
1781	ENSMUSG00000090733	Rps27	1.62	5.32E-10	3.05E-09
1782	ENSMUSG00000078941	Ak6	1.23	5.35E-10	3.07E-09
1783	ENSMUSG00000043510	Hscb	1.85	5.35E-10	3.07E-09
1784	ENSMUSG00000037519	Ppfia1	-1.49	5.39E-10	3.09E-09
1785	ENSMUSG00000006333	Rps9	1.28	5.43E-10	3.11E-09
1786	ENSMUSG00000087165	2010001A14Rik	1.77	5.45E-10	3.12E-09
1787	ENSMUSG00000028430	Nol6	-1.36	5.50E-10	3.15E-09
1788	ENSMUSG00000066952	Myo1h	-1.24	5.57E-10	3.19E-09
1789	ENSMUSG00000058624	Gda	-1.77	5.58E-10	3.19E-09
1790	ENSMUSG00000021054	Sgpp1	-1.89	5.60E-10	3.20E-09
1791	ENSMUSG00000028479	Gne	-1.19	5.60E-10	3.20E-09
1792	ENSMUSG00000029313	Aff1	-1.44	5.65E-10	3.22E-09
1793	ENSMUSG00000103458	Gm42416	-1.62	5.67E-10	3.24E-09
1794	ENSMUSG00000039230	Tbcd	-1.33	5.68E-10	3.24E-09
1795	ENSMUSG00000020484	Xbp1	-1.64	5.68E-10	3.24E-09
1796	ENSMUSG00000032479	Map4	-1.34	5.71E-10	3.25E-09
1797	ENSMUSG00000019054	Fis1	1.27	5.71E-10	3.25E-09

1798	ENSMUSG00000015312	Gadd45b	-1.60	5.73E-10	3.26E-09
1799	ENSMUSG00000071649	B3gat3	1.34	5.81E-10	3.30E-09
1800	ENSMUSG00000104217	Gm37988	1.09	5.90E-10	3.35E-09
1801	ENSMUSG00000029993	Nfu1	1.15	5.97E-10	3.39E-09
1802	ENSMUSG00000086429	Gt(ROSA)26Sor	1.45	5.98E-10	3.39E-09
1803	ENSMUSG00000020003	Pex7	1.22	6.03E-10	3.42E-09
1804	ENSMUSG00000032786	Alas1	-1.19	6.05E-10	3.43E-09
1805	ENSMUSG00000055980	Irs1	-1.34	6.07E-10	3.44E-09
1806	ENSMUSG00000043673	Kcns3	-1.35	6.16E-10	3.49E-09
1807	ENSMUSG00000025103	Btbd1	1.08	6.23E-10	3.53E-09
1808	ENSMUSG00000026361	Cdc73	-1.32	6.28E-10	3.55E-09
1809	ENSMUSG00000025746	Il6	-1.62	6.35E-10	3.59E-09
1810	ENSMUSG00000020717	Pecam1	-1.50	6.40E-10	3.62E-09
1811	ENSMUSG00000087231	E230016M11Rik	-1.70	6.42E-10	3.62E-09
1812	ENSMUSG00000066800	Rnasel	-2.06	6.42E-10	3.62E-09
1813	ENSMUSG00000021136	Smoc1	-1.72	6.49E-10	3.66E-09
1814	ENSMUSG00000023084	Lrrc71	1.41	6.58E-10	3.71E-09
1815	ENSMUSG00000025971	Maip1	1.25	6.59E-10	3.71E-09
1816	ENSMUSG00000059714	Flot1	-1.11	6.69E-10	3.77E-09
1817	ENSMUSG00000027878	Notch2	-1.68	6.69E-10	3.77E-09
1818	ENSMUSG00000035642	Aamdc	1.15	6.76E-10	3.80E-09
1819	ENSMUSG00000027472	Pdrg1	1.28	6.78E-10	3.81E-09
1820	ENSMUSG00000031380	Vegfd	-1.68	6.78E-10	3.81E-09
1821	ENSMUSG00000020978	Klhdc2	1.13	6.81E-10	3.82E-09
1822	ENSMUSG00000031529	Tnks	-1.53	6.86E-10	3.85E-09
1823	ENSMUSG00000017412	Cacnb4	1.40	6.87E-10	3.85E-09
1824	ENSMUSG00000028563	Tm2d1	1.15	6.96E-10	3.90E-09
1825	ENSMUSG00000055296	Tmem245	-1.49	7.05E-10	3.95E-09
1826	ENSMUSG00000030104	Edem1	-1.17	7.05E-10	3.95E-09
1827	ENSMUSG00000021585	Cast	-1.21	7.14E-10	4.00E-09
1828	ENSMUSG00000007777	0610009B22Rik	1.37	7.17E-10	4.01E-09
1829	ENSMUSG00000098004	Gm27027	1.36	7.26E-10	4.06E-09
1830	ENSMUSG00000025362	Rps26	1.29	7.29E-10	4.07E-09
1831	ENSMUSG00000036139	Hoxc9	-1.79	7.29E-10	4.07E-09
1832	ENSMUSG00000036863	Syde2	-1.60	7.30E-10	4.08E-09
1833	ENSMUSG00000018822	Sfrp5	-2.27	7.33E-10	4.09E-09
1834	ENSMUSG00000047496	Rnf152	1.87	7.33E-10	4.09E-09
1835	ENSMUSG00000025876	Unc5a	-1.62	7.44E-10	4.14E-09
1836	ENSMUSG00000056666	Retsat	1.09	7.57E-10	4.22E-09
1837	ENSMUSG00000037440	Vnn1	-2.22	7.67E-10	4.27E-09
1838	ENSMUSG00000091479	Gm17035	-1.91	7.75E-10	4.31E-09
1839	ENSMUSG00000020580	Rock2	-1.27	7.78E-10	4.33E-09
1840	ENSMUSG00000001445	Mrpl10	1.04	7.80E-10	4.34E-09
1841	ENSMUSG00000018882	Mrpl45	1.11	7.81E-10	4.34E-09
1842	ENSMUSG00000061436	Hipk2	-1.18	7.82E-10	4.34E-09
1843	ENSMUSG00000031785	Adgrg1	-1.44	7.87E-10	4.37E-09
1844	ENSMUSG00000102329	Gm10851	1.30	7.88E-10	4.37E-09
1845	ENSMUSG00000042834	Nrep	-2.00	7.90E-10	4.38E-09
1846	ENSMUSG00000078606	Gm4070	-2.16	7.94E-10	4.40E-09
1847	ENSMUSG00000059343	Aldoart1	1.74	8.05E-10	4.45E-09

1848	ENSMUSG00000021114	Atp6v1d	1.12	8.05E-10	4.45E-09
1849	ENSMUSG00000023249	Parp3	-1.12	8.09E-10	4.48E-09
1850	ENSMUSG00000079435	Rpl36a	1.35	8.10E-10	4.48E-09
1851	ENSMUSG00000028127	Abcd3	1.12	8.16E-10	4.51E-09
1852	ENSMUSG00000039195	1110008P14Rik	1.24	8.22E-10	4.54E-09
1853	ENSMUSG00000089781	Gm15756	-1.36	8.31E-10	4.59E-09
1854	ENSMUSG00000032271	Nnmt	-1.26	8.32E-10	4.59E-09
1855	ENSMUSG00000029815	Malsu1	1.39	8.35E-10	4.60E-09
1856	ENSMUSG00000044881	Coa4	1.65	8.38E-10	4.62E-09
1857	ENSMUSG00000021951	Eef1akmt1	1.36	8.40E-10	4.62E-09
1858	ENSMUSG00000073490	Ifi207	-2.20	8.40E-10	4.63E-09
1859	ENSMUSG00000032596	Uba7	-1.65	8.42E-10	4.63E-09
1860	ENSMUSG00000045414	1190002N15Rik	-1.40	8.44E-10	4.64E-09
1861	ENSMUSG00000045968	Teddm2	1.06	8.47E-10	4.65E-09
1862	ENSMUSG00000052331	Ankrd44	-1.81	8.51E-10	4.67E-09
1863	ENSMUSG00000019278	Dpep1	1.51	8.57E-10	4.71E-09
1864	ENSMUSG00000092071	A230065N10Rik	1.82	8.58E-10	4.71E-09
1865	ENSMUSG00000020331	Hcn2	1.87	8.78E-10	4.81E-09
1866	ENSMUSG00000044405	Adig	1.16	8.91E-10	4.88E-09
1867	ENSMUSG00000078681	Tm2d3	1.21	8.91E-10	4.88E-09
1868	ENSMUSG00000022574	Naprt	1.07	8.91E-10	4.88E-09
1869	ENSMUSG00000098140	Gm26938	1.61	8.92E-10	4.88E-09
1870	ENSMUSG00000031078	Cttn	-1.04	8.93E-10	4.88E-09
1871	ENSMUSG00000027890	Gstm4	1.11	8.99E-10	4.91E-09
1872	ENSMUSG00000002846	Timmdc1	1.49	9.01E-10	4.92E-09
1873	ENSMUSG00000019738	Polr2i	1.22	9.09E-10	4.96E-09
1874	ENSMUSG00000020541	Tom1l1	1.21	9.11E-10	4.97E-09
1875	ENSMUSG00000018287	Spag7	1.09	9.19E-10	5.01E-09
1876	ENSMUSG00000032299	Commd4	1.15	9.23E-10	5.03E-09
1877	ENSMUSG00000026021	Sumo1	1.12	9.41E-10	5.13E-09
1878	ENSMUSG00000067194	Eif1ax	1.15	9.68E-10	5.27E-09
1879	ENSMUSG00000020841	Cpd	-1.49	9.72E-10	5.29E-09
1880	ENSMUSG00000049489	Fam58b	1.45	9.74E-10	5.30E-09
1881	ENSMUSG00000023186	Vwa5a	-1.28	9.75E-10	5.30E-09
1882	ENSMUSG00000042831	Alkbh6	1.30	9.76E-10	5.30E-09
1883	ENSMUSG00000032459	Mrps22	1.20	9.92E-10	5.39E-09
1884	ENSMUSG00000041319	Thoc6	1.36	1.02E-09	5.54E-09
1885	ENSMUSG00000035713	Usp35	-1.72	1.02E-09	5.54E-09
1886	ENSMUSG00000079491	H2-T10	-1.16	1.04E-09	5.62E-09
1887	ENSMUSG00000058799	Nap1l1	-1.15	1.04E-09	5.64E-09
1888	ENSMUSG00000025372	Baiap2	-1.73	1.05E-09	5.70E-09
1889	ENSMUSG00000073555	Gm4951	-1.95	1.06E-09	5.74E-09
1890	ENSMUSG00000029433	Diablo	1.14	1.06E-09	5.75E-09
1891	ENSMUSG00000025350	Rdh5	1.28	1.09E-09	5.87E-09
1892	ENSMUSG00000054843	Atrnl1	-1.75	1.09E-09	5.88E-09
1893	ENSMUSG00000047731	Wbp1l	-1.17	1.09E-09	5.89E-09
1894	ENSMUSG00000005054	Cstb	1.18	1.09E-09	5.89E-09
1895	ENSMUSG00000037216	Lipt1	1.37	1.09E-09	5.90E-09
1896	ENSMUSG00000001036	Epn2	-1.40	1.10E-09	5.93E-09
1897	ENSMUSG00000047409	Ctdspl	-1.49	1.10E-09	5.94E-09

1898	ENSMUSG00000035168	Tanc1	-1.44	1.11E-09	5.96E-09
1899	ENSMUSG00000049233	Apoo-ps	1.76	1.12E-09	6.05E-09
1900	ENSMUSG00000020225	Tmbim4	1.08	1.13E-09	6.05E-09
1901	ENSMUSG00000079641	Rpl39	1.22	1.13E-09	6.05E-09
1902	ENSMUSG00000033543	Gtf2a2	1.15	1.14E-09	6.10E-09
1903	ENSMUSG00000024892	Pcx	-1.79	1.14E-09	6.11E-09
1904	ENSMUSG00000028672	Hmgcl	1.04	1.14E-09	6.12E-09
1905	ENSMUSG00000039048	Foxred1	1.04	1.15E-09	6.15E-09
1906	ENSMUSG00000063235	Ptpmt1	1.14	1.15E-09	6.17E-09
1907	ENSMUSG00000027555	Car13	1.35	1.16E-09	6.22E-09
1908	ENSMUSG00000032575	Manf	1.29	1.16E-09	6.22E-09
1909	ENSMUSG00000110613	Lncbate1	1.33	1.16E-09	6.23E-09
1910	ENSMUSG00000084786	Ubl5	1.20	1.17E-09	6.24E-09
1911	ENSMUSG00000037405	Icam1	-2.02	1.17E-09	6.25E-09
1912	ENSMUSG00000026880	Stom	1.10	1.17E-09	6.26E-09
1913	ENSMUSG00000111059	AC160116.1	1.82	1.18E-09	6.30E-09
1914	ENSMUSG00000085315	A430018G15Rik	1.40	1.19E-09	6.36E-09
1915	ENSMUSG00000046727	Cystm1	1.32	1.19E-09	6.36E-09
1916	ENSMUSG00000056917	Sipa1	-1.73	1.20E-09	6.42E-09
1917	ENSMUSG00000016541	Atxn10	-1.10	1.21E-09	6.45E-09
1918	ENSMUSG00000060429	Sntb1	-2.15	1.22E-09	6.49E-09
1919	ENSMUSG00000019189	Rnf145	-1.33	1.23E-09	6.53E-09
1920	ENSMUSG00000045948	Mrps12	1.25	1.23E-09	6.53E-09
1921	ENSMUSG00000020664	Dld	1.04	1.23E-09	6.56E-09
1922	ENSMUSG00000021996	Esd	1.12	1.25E-09	6.64E-09
1923	ENSMUSG00000043190	Rfesd	1.48	1.25E-09	6.66E-09
1924	ENSMUSG00000016319	Slc25a5	1.11	1.26E-09	6.69E-09
1925	ENSMUSG00000035311	Gnptab	-1.34	1.26E-09	6.70E-09
1926	ENSMUSG00000025731	Mettl26	1.20	1.26E-09	6.71E-09
1927	ENSMUSG00000002393	Nr2f6	1.07	1.28E-09	6.79E-09
1928	ENSMUSG00000090247	Bloc1s1	1.28	1.31E-09	6.94E-09
1929	ENSMUSG00000040128	Pnrc1	-1.12	1.32E-09	6.98E-09
1930	ENSMUSG00000053581	Zfand2a	-1.40	1.33E-09	7.03E-09
1931	ENSMUSG00000032383	Ppib	1.09	1.33E-09	7.03E-09
1932	ENSMUSG00000038527	C1rl	1.11	1.33E-09	7.03E-09
1933	ENSMUSG00000025484	Bet1l	1.23	1.35E-09	7.13E-09
1934	ENSMUSG00000042032	Mat2b	1.09	1.37E-09	7.22E-09
1935	ENSMUSG00000048100	Taf13	1.20	1.37E-09	7.22E-09
1936	ENSMUSG00000053453	Thoc7	1.30	1.38E-09	7.26E-09
1937	ENSMUSG00000002010	Idh3g	1.20	1.38E-09	7.27E-09
1938	ENSMUSG00000043964	Orai3	1.20	1.38E-09	7.30E-09
1939	ENSMUSG00000028455	Stoml2	1.19	1.38E-09	7.30E-09
1940	ENSMUSG00000027276	Jag1	-1.90	1.40E-09	7.37E-09
1941	ENSMUSG00000001098	Kctd10	-1.17	1.40E-09	7.38E-09
1942	ENSMUSG00000029458	Brap	1.11	1.42E-09	7.45E-09
1943	ENSMUSG00000033940	Brk1	1.11	1.42E-09	7.48E-09
1944	ENSMUSG00000021950	Anxa8	-2.01	1.44E-09	7.56E-09
1945	ENSMUSG00000022895	Ets2	-1.41	1.45E-09	7.63E-09
1946	ENSMUSG00000022505	Emp2	-1.66	1.45E-09	7.64E-09
1947	ENSMUSG00000000384	Tbrg4	1.39	1.46E-09	7.64E-09

1948	ENSMUSG00000018909	Arrb1	-1.79	1.46E-09	7.65E-09
1949	ENSMUSG00000062328	Rpl17	1.54	1.46E-09	7.65E-09
1950	ENSMUSG00000026941	Mamdc4	1.26	1.46E-09	7.65E-09
1951	ENSMUSG00000049241	Hcar1	-2.40	1.47E-09	7.68E-09
1952	ENSMUSG00000051495	Irf2bp2	-1.56	1.47E-09	7.70E-09
1953	ENSMUSG00000074884	Serf2	1.22	1.48E-09	7.77E-09
1954	ENSMUSG00000105204	Gm43738	1.37	1.52E-09	7.93E-09
1955	ENSMUSG00000031207	Msn	-1.37	1.53E-09	7.98E-09
1956	ENSMUSG00000097383	1500026H17Rik	1.86	1.53E-09	8.00E-09
1957	ENSMUSG00000057738	Sptan1	-1.09	1.53E-09	8.01E-09
1958	ENSMUSG00000054545	Ugt1a6a	-2.15	1.54E-09	8.04E-09
1959	ENSMUSG00000038388	Mpp6	1.32	1.55E-09	8.07E-09
1960	ENSMUSG00000004896	Rrnad1	1.20	1.56E-09	8.14E-09
1961	ENSMUSG00000025792	Slc25a10	-2.36	1.57E-09	8.18E-09
1962	ENSMUSG00000000184	Ccnd2	-1.58	1.58E-09	8.21E-09
1963	ENSMUSG00000021193	Pitrm1	-1.15	1.58E-09	8.21E-09
1964	ENSMUSG00000049807	Arhgap23	-1.43	1.58E-09	8.21E-09
1965	ENSMUSG00000086929	Gm11788	-2.33	1.58E-09	8.21E-09
1966	ENSMUSG00000039452	Snx22	1.10	1.58E-09	8.24E-09
1967	ENSMUSG00000075029	4930558J22Rik	1.63	1.59E-09	8.25E-09
1968	ENSMUSG00000032402	Smad3	-1.55	1.59E-09	8.28E-09
1969	ENSMUSG00000024592	C330018D20Rik	1.21	1.60E-09	8.29E-09
1970	ENSMUSG00000041921	Metap1d	1.31	1.60E-09	8.30E-09
1971	ENSMUSG00000039357	Fut11	-1.87	1.60E-09	8.31E-09
1972	ENSMUSG00000063229	Ldha	1.23	1.61E-09	8.36E-09
1973	ENSMUSG00000014504	Srp19	1.20	1.61E-09	8.36E-09
1974	ENSMUSG00000059920	4930453N24Rik	1.05	1.63E-09	8.45E-09
1975	ENSMUSG00000018740	Slc25a35	1.18	1.65E-09	8.56E-09
1976	ENSMUSG00000029591	Ung	1.74	1.66E-09	8.57E-09
1977	ENSMUSG00000021650	Ptcd2	1.10	1.68E-09	8.66E-09
1978	ENSMUSG00000026278	Bok	-1.60	1.69E-09	8.73E-09
1979	ENSMUSG00000030801	Kat8	1.18	1.69E-09	8.75E-09
1980	ENSMUSG00000021097	Clmn	-1.62	1.72E-09	8.86E-09
1981	ENSMUSG00000085655	Gm15952	-1.19	1.72E-09	8.88E-09
1982	ENSMUSG00000026589	Sec16b	-2.21	1.73E-09	8.90E-09
1983	ENSMUSG00000036504	Phpt1	1.24	1.73E-09	8.94E-09
1984	ENSMUSG00000000131	Xpo6	-1.31	1.74E-09	8.95E-09
1985	ENSMUSG00000025283	Sat1	1.34	1.76E-09	9.04E-09
1986	ENSMUSG00000024194	Cuta	1.20	1.76E-09	9.07E-09
1987	ENSMUSG00000018841	Rad51d	-1.12	1.77E-09	9.13E-09
1988	ENSMUSG00000110834	CAAA01180111.2	1.75	1.78E-09	9.15E-09
1989	ENSMUSG00000031557	Plekha2	-1.65	1.78E-09	9.16E-09
1990	ENSMUSG00000090124	Ugt1a7c	-2.17	1.78E-09	9.16E-09
1991	ENSMUSG00000040462	Os9	-1.06	1.79E-09	9.18E-09
1992	ENSMUSG00000032549	Rab6b	1.60	1.79E-09	9.21E-09
1993	ENSMUSG00000026526	Fh1	1.11	1.80E-09	9.25E-09
1994	ENSMUSG00000023942	Slc29a1	1.85	1.82E-09	9.34E-09
1995	ENSMUSG00000022280	Rnf19a	-1.28	1.82E-09	9.34E-09
1996	ENSMUSG00000024789	Jak2	-1.55	1.83E-09	9.38E-09
1997	ENSMUSG00000100691	2010320M18Rik	1.44	1.84E-09	9.40E-09

1998	ENSMUSG00000075467	Dnlz	1.14	1.85E-09	9.44E-09
1999	ENSMUSG00000024236	Svil	-1.54	1.86E-09	9.49E-09
2000	ENSMUSG00000039167	Adgrl4	-1.35	1.89E-09	9.65E-09
2001	ENSMUSG00000041769	Ppp2r2d	1.08	1.90E-09	9.72E-09
2002	ENSMUSG00000024713	Pcsk5	-1.50	1.90E-09	9.72E-09
2003	ENSMUSG00000033735	Spr	1.17	1.90E-09	9.72E-09
2004	ENSMUSG00000108368	Gm45053	1.05	1.91E-09	9.73E-09
2005	ENSMUSG00000025510	Cd151	1.40	1.92E-09	9.77E-09
2006	ENSMUSG00000029321	Slc10a6	-2.05	1.92E-09	9.78E-09
2007	ENSMUSG00000054733	Msra	1.07	1.92E-09	9.80E-09
2008	ENSMUSG00000063077	Kif1b	-1.08	1.94E-09	9.87E-09
2009	ENSMUSG00000005142	Man2b1	-1.28	1.94E-09	9.87E-09
2010	ENSMUSG00000063439	B9d2	1.30	1.95E-09	9.91E-09
2011	ENSMUSG00000039629	Strip2	-1.75	2.01E-09	1.02E-08
2012	ENSMUSG00000024914	Drap1	1.15	2.07E-09	1.05E-08
2013	ENSMUSG00000004508	Gab2	-1.53	2.10E-09	1.07E-08
2014	ENSMUSG00000078816	Prkcg	-1.75	2.11E-09	1.07E-08
2015	ENSMUSG00000024725	Ostf1	1.25	2.12E-09	1.07E-08
2016	ENSMUSG00000035376	Hacd2	1.12	2.12E-09	1.08E-08
2017	ENSMUSG00000033701	Acbd6	1.13	2.13E-09	1.08E-08
2018	ENSMUSG00000037020	Wdr62	-1.67	2.13E-09	1.08E-08
2019	ENSMUSG00000006390	Elovl1	-1.43	2.15E-09	1.09E-08
2020	ENSMUSG00000096221	1500002C15Rik	1.14	2.15E-09	1.09E-08
2021	ENSMUSG00000031880	Rrad	-1.91	2.16E-09	1.09E-08
2022	ENSMUSG00000013858	Tmem259	1.25	2.16E-09	1.09E-08
2023	ENSMUSG00000059743	Fdps	1.36	2.17E-09	1.10E-08
2024	ENSMUSG00000050627	Gpd1l	-1.22	2.18E-09	1.10E-08
2025	ENSMUSG00000024875	Yif1a	1.01	2.18E-09	1.10E-08
2026	ENSMUSG00000038593	Tctn1	-1.61	2.20E-09	1.11E-08
2027	ENSMUSG00000025980	Hspd1	1.02	2.21E-09	1.12E-08
2028	ENSMUSG00000031198	Fundc2	1.07	2.23E-09	1.12E-08
2029	ENSMUSG00000034957	Cebpa	-1.09	2.24E-09	1.13E-08
2030	ENSMUSG00000020483	Dynll2	1.23	2.25E-09	1.13E-08
2031	ENSMUSG00000102997	Gm17244	1.62	2.28E-09	1.15E-08
2032	ENSMUSG00000097333	Zfp87	1.47	2.29E-09	1.15E-08
2033	ENSMUSG00000056501	Cebpb	1.13	2.35E-09	1.18E-08
2034	ENSMUSG00000010607	Pigyl	1.22	2.36E-09	1.18E-08
2035	ENSMUSG00000089783	Gm454	-1.55	2.38E-09	1.20E-08
2036	ENSMUSG00000038456	Dennd2a	-1.41	2.40E-09	1.20E-08
2037	ENSMUSG00000037306	Man1c1	-1.71	2.41E-09	1.21E-08
2038	ENSMUSG00000032892	Rangrf	1.36	2.45E-09	1.23E-08
2039	ENSMUSG00000021540	Smad5	-1.31	2.46E-09	1.23E-08
2040	ENSMUSG00000087338	Scpep1os	1.26	2.48E-09	1.25E-08
2041	ENSMUSG00000024866	Acy3	1.20	2.49E-09	1.25E-08
2042	ENSMUSG00000032452	Clstn2	-2.05	2.50E-09	1.25E-08
2043	ENSMUSG00000061477	Rps7	1.33	2.55E-09	1.27E-08
2044	ENSMUSG00000001473	Tubb6	-1.21	2.56E-09	1.28E-08
2045	ENSMUSG00000014769	Psmb1	1.06	2.59E-09	1.30E-08
2046	ENSMUSG00000012705	Retn	-2.44	2.59E-09	1.30E-08
2047	ENSMUSG00000041216	Clvs1	-1.65	2.60E-09	1.30E-08

2048	ENSMUSG00000078429	Ctdsp2	-1.21	2.61E-09	1.30E-08
2049	ENSMUSG00000002342	Tmem161a	-1.21	2.65E-09	1.33E-08
2050	ENSMUSG00000032855	Pkd1	-1.32	2.66E-09	1.33E-08
2051	ENSMUSG00000026611	Spata17	1.73	2.68E-09	1.34E-08
2052	ENSMUSG00000026189	Pecr	1.07	2.69E-09	1.34E-08
2053	ENSMUSG00000046792	Zfp787	1.29	2.76E-09	1.37E-08
2054	ENSMUSG00000035900	Gramd4	-1.52	2.77E-09	1.38E-08
2055	ENSMUSG00000068663	Clec16a	1.24	2.78E-09	1.38E-08
2056	ENSMUSG00000006526	Tmem110	1.05	2.79E-09	1.39E-08
2057	ENSMUSG00000041028	Ghitm	1.06	2.81E-09	1.40E-08
2058	ENSMUSG00000041241	Mul1	1.07	2.83E-09	1.41E-08
2059	ENSMUSG00000001657	Hoxc8	-1.34	2.84E-09	1.41E-08
2060	ENSMUSG00000035441	Myo1d	-1.70	2.85E-09	1.42E-08
2061	ENSMUSG00000041426	Hibch	1.01	2.87E-09	1.43E-08
2062	ENSMUSG00000037999	Arap2	-1.42	2.88E-09	1.43E-08
2063	ENSMUSG00000028383	Hsdl2	0.99	2.88E-09	1.43E-08
2064	ENSMUSG00000024663	Rab3il1	-1.81	2.92E-09	1.45E-08
2065	ENSMUSG00000015522	Arnt	-1.43	3.00E-09	1.49E-08
2066	ENSMUSG00000019494	Cops6	0.99	3.02E-09	1.50E-08
2067	ENSMUSG00000043140	Tmem186	1.08	3.03E-09	1.50E-08
2068	ENSMUSG00000034300	Fam53c	-1.16	3.03E-09	1.50E-08
2069	ENSMUSG00000026798	Coq4	1.14	3.07E-09	1.52E-08
2070	ENSMUSG00000032902	Slc16a1	1.22	3.11E-09	1.54E-08
2071	ENSMUSG00000027305	Ndufaf1	1.12	3.11E-09	1.54E-08
2072	ENSMUSG00000021763	BC067074	-1.62	3.12E-09	1.54E-08
2073	ENSMUSG00000020407	Upp1	1.90	3.14E-09	1.55E-08
2074	ENSMUSG00000028128	F3	-1.16	3.16E-09	1.56E-08
2075	ENSMUSG00000051319	1500011K16Rik	1.40	3.17E-09	1.56E-08
2076	ENSMUSG00000030760	Acer3	1.43	3.19E-09	1.57E-08
2077	ENSMUSG00000026923	Notch1	-1.53	3.23E-09	1.59E-08
2078	ENSMUSG00000011257	Pabpc4	1.49	3.23E-09	1.59E-08
2079	ENSMUSG00000029561	Oasl2	-1.68	3.24E-09	1.59E-08
2080	ENSMUSG00000030068	Gm20696	1.12	3.24E-09	1.59E-08
2081	ENSMUSG00000002396	Ocel1	1.04	3.26E-09	1.60E-08
2082	ENSMUSG00000097006	9530082P21Rik	-1.15	3.27E-09	1.61E-08
2083	ENSMUSG00000032112	Trappc4	1.05	3.30E-09	1.62E-08
2084	ENSMUSG00000059981	Taok2	-1.28	3.32E-09	1.63E-08
2085	ENSMUSG00000031537	Ikbkb	-1.27	3.39E-09	1.66E-08
2086	ENSMUSG00000052698	Tln2	-1.64	3.44E-09	1.68E-08
2087	ENSMUSG00000058833	2810428I15Rik	1.25	3.44E-09	1.68E-08
2088	ENSMUSG00000026867	Gapvd1	-1.14	3.47E-09	1.70E-08
2089	ENSMUSG00000027569	Mrgbp	1.58	3.55E-09	1.74E-08
2090	ENSMUSG00000056116	H2-T22	-1.03	3.55E-09	1.74E-08
2091	ENSMUSG00000036599	Chst12	-1.14	3.57E-09	1.75E-08
2092	ENSMUSG00000025558	Dock9	-1.83	3.58E-09	1.75E-08
2093	ENSMUSG00000028700	Pomgnt1	0.98	3.58E-09	1.75E-08
2094	ENSMUSG00000027642	Rpn2	-1.08	3.62E-09	1.77E-08
2095	ENSMUSG00000034422	Parp14	-1.47	3.66E-09	1.78E-08
2096	ENSMUSG00000034342	Cbl	-1.63	3.66E-09	1.78E-08
2097	ENSMUSG00000058351	Smim4	1.58	3.69E-09	1.80E-08

2098	ENSMUSG00000038811	Gngt2	1.62	3.71E-09	1.81E-08
2099	ENSMUSG00000030255	Sspn	-1.28	3.74E-09	1.82E-08
2100	ENSMUSG00000028995	Fam126a	-1.45	3.74E-09	1.82E-08
2101	ENSMUSG00000028268	Gbp3	-1.65	3.76E-09	1.83E-08
2102	ENSMUSG00000004032	Gstm5	1.08	3.81E-09	1.85E-08
2103	ENSMUSG00000001100	Poldip2	0.99	3.84E-09	1.87E-08
2104	ENSMUSG00000030401	Rtn2	-1.47	3.84E-09	1.87E-08
2105	ENSMUSG00000033871	Ppargc1b	1.15	3.85E-09	1.87E-08
2106	ENSMUSG00000054612	Mgmt	1.41	3.85E-09	1.87E-08
2107	ENSMUSG00000040296	Ddx58	-1.56	3.86E-09	1.88E-08
2108	ENSMUSG00000052512	Nav2	1.69	3.88E-09	1.88E-08
2109	ENSMUSG00000017446	C1qtnf1	-1.94	3.90E-09	1.89E-08
2110	ENSMUSG00000029869	Ephb6	-1.88	3.92E-09	1.90E-08
2111	ENSMUSG00000032571	Pik3r4	-1.34	3.93E-09	1.90E-08
2112	ENSMUSG00000032615	Nt5m	1.05	3.93E-09	1.91E-08
2113	ENSMUSG00000043733	Ptpn11	1.09	3.97E-09	1.92E-08
2114	ENSMUSG00000053329	D10Jhu81e	1.16	4.00E-09	1.93E-08
2115	ENSMUSG00000024308	Tapbp	-1.20	4.04E-09	1.96E-08
2116	ENSMUSG00000039209	Rpl39l	1.72	4.09E-09	1.98E-08
2117	ENSMUSG00000026739	Bmi1	1.10	4.09E-09	1.98E-08
2118	ENSMUSG00000058793	Cds2	-1.08	4.09E-09	1.98E-08
2119	ENSMUSG00000037206	Islr	-1.89	4.10E-09	1.98E-08
2120	ENSMUSG00000110344	Gm45716	-1.07	4.17E-09	2.01E-08
2121	ENSMUSG00000025069	Gsto2	1.51	4.21E-09	2.03E-08
2122	ENSMUSG00000057278	Snrpg	1.56	4.24E-09	2.05E-08
2123	ENSMUSG00000029165	Agbl5	1.12	4.25E-09	2.05E-08
2124	ENSMUSG00000020180	Snrpd3	1.41	4.26E-09	2.05E-08
2125	ENSMUSG00000030717	Nupr1	-1.11	4.32E-09	2.08E-08
2126	ENSMUSG00000037926	Ssh2	-1.35	4.39E-09	2.11E-08
2127	ENSMUSG00000074698	Csnk2a1	1.22	4.42E-09	2.12E-08
2128	ENSMUSG00000051118	Olfr77	1.69	4.43E-09	2.13E-08
2129	ENSMUSG00000038679	Trps1	-1.82	4.49E-09	2.16E-08
2130	ENSMUSG00000098650	Gm28048	1.04	4.49E-09	2.16E-08
2131	ENSMUSG00000001424	Snd1	-1.29	4.49E-09	2.16E-08
2132	ENSMUSG00000024012	Mtch1	0.99	4.51E-09	2.16E-08
2133	ENSMUSG00000020868	Xylt2	-1.29	4.52E-09	2.17E-08
2134	ENSMUSG00000044968	Napepld	-1.27	4.53E-09	2.17E-08
2135	ENSMUSG00000030826	Bcat2	-1.21	4.62E-09	2.21E-08
2136	ENSMUSG00000058446	Znrf2	-1.11	4.63E-09	2.22E-08
2137	ENSMUSG00000059291	Rpl11	1.21	4.66E-09	2.23E-08
2138	ENSMUSG00000055041	Commd5	1.25	4.69E-09	2.24E-08
2139	ENSMUSG00000028926	Cdk14	-1.25	4.75E-09	2.27E-08
2140	ENSMUSG00000038371	Sbf2	-1.21	4.77E-09	2.28E-08
2141	ENSMUSG00000020638	Cmpk2	-1.53	4.80E-09	2.29E-08
2142	ENSMUSG00000037013	Ss18	-1.05	4.80E-09	2.29E-08
2143	ENSMUSG00000013236	Ptprs	-1.50	4.81E-09	2.30E-08
2144	ENSMUSG00000001739	Cldn15	-1.59	4.82E-09	2.30E-08
2145	ENSMUSG00000006403	Adamts4	-1.81	4.83E-09	2.30E-08
2146	ENSMUSG00000003752	Itpkc	-1.48	4.83E-09	2.30E-08
2147	ENSMUSG00000020167	Tcf3	-1.36	4.84E-09	2.31E-08

2148	ENSMUSG00000037242	Clic4	-1.09	4.86E-09	2.31E-08
2149	ENSMUSG00000043885	Slc36a4	-1.48	4.87E-09	2.32E-08
2150	ENSMUSG00000026482	Rgl1	-1.10	4.88E-09	2.32E-08
2151	ENSMUSG00000036273	Lrrk2	-1.78	4.95E-09	2.35E-08
2152	ENSMUSG00000039771	Polr2j	1.25	4.98E-09	2.37E-08
2153	ENSMUSG00000029094	Afap1	-1.26	5.03E-09	2.39E-08
2154	ENSMUSG00000073968	Trim68	-1.46	5.04E-09	2.39E-08
2155	ENSMUSG00000027360	Hdc	1.78	5.05E-09	2.39E-08
2156	ENSMUSG00000097565	Gm26965	1.48	5.06E-09	2.40E-08
2157	ENSMUSG00000007656	Arpp19	1.09	5.09E-09	2.41E-08
2158	ENSMUSG00000032387	Rbpms2	1.00	5.10E-09	2.42E-08
2159	ENSMUSG00000018446	C1qbp	1.11	5.16E-09	2.44E-08
2160	ENSMUSG00000038065	Mturn	-1.16	5.19E-09	2.46E-08
2161	ENSMUSG00000040620	Dhx33	-1.24	5.28E-09	2.50E-08
2162	ENSMUSG00000059146	Ntrk3	1.55	5.28E-09	2.50E-08
2163	ENSMUSG00000027312	Atrn	-1.35	5.29E-09	2.50E-08
2164	ENSMUSG00000087611	4930458D05Rik	-1.68	5.31E-09	2.51E-08
2165	ENSMUSG00000085262	Gm11574	1.05	5.34E-09	2.52E-08
2166	ENSMUSG00000047617	BC029214	1.47	5.39E-09	2.54E-08
2167	ENSMUSG00000030780	BC017158	-1.24	5.41E-09	2.55E-08
2168	ENSMUSG00000109455	Gm44710	1.30	5.45E-09	2.57E-08
2169	ENSMUSG00000002769	Gnmt	1.81	5.58E-09	2.63E-08
2170	ENSMUSG00000020232	Hmg20b	1.39	5.77E-09	2.72E-08
2171	ENSMUSG00000022913	Psmg1	1.13	5.81E-09	2.74E-08
2172	ENSMUSG00000040521	Tsfm	1.20	5.84E-09	2.75E-08
2173	ENSMUSG00000078919	Dpm1	1.03	5.87E-09	2.76E-08
2174	ENSMUSG00000073842	Mup7	-1.65	5.89E-09	2.77E-08
2175	ENSMUSG00000004626	Stxbp2	1.01	5.91E-09	2.78E-08
2176	ENSMUSG00000027313	Chac1	-1.70	5.92E-09	2.78E-08
2177	ENSMUSG00000026223	Itm2c	-1.26	5.95E-09	2.79E-08
2178	ENSMUSG00000066900	Suds3	1.00	5.97E-09	2.80E-08
2179	ENSMUSG00000033751	Gadd45gip1	1.18	5.97E-09	2.80E-08
2180	ENSMUSG00000037894	H2afz	1.01	5.97E-09	2.80E-08
2181	ENSMUSG00000005779	Psmb4	1.03	6.00E-09	2.81E-08
2182	ENSMUSG00000030671	Pde3b	-1.16	6.08E-09	2.85E-08
2183	ENSMUSG00000018326	Ywhab	-0.99	6.09E-09	2.85E-08
2184	ENSMUSG00000024570	Rbfa	1.05	6.16E-09	2.88E-08
2185	ENSMUSG00000030751	Psma1	0.97	6.29E-09	2.94E-08
2186	ENSMUSG00000062382	Ftl1-ps1	1.12	6.37E-09	2.98E-08
2187	ENSMUSG00000064307	Lrrc51	1.20	6.45E-09	3.02E-08
2188	ENSMUSG00000028405	Aco1	-1.08	6.48E-09	3.03E-08
2189	ENSMUSG00000022436	Sh3bp1	-1.68	6.48E-09	3.03E-08
2190	ENSMUSG00000021957	Tkt	1.34	6.53E-09	3.05E-08
2191	ENSMUSG00000045128	Rpl18a	1.11	6.56E-09	3.06E-08
2192	ENSMUSG00000022031	Elp3	-1.22	6.60E-09	3.08E-08
2193	ENSMUSG00000025246	Tbl1x	-1.11	6.62E-09	3.09E-08
2194	ENSMUSG00000026227	2810459M11Rik	-1.92	6.66E-09	3.10E-08
2195	ENSMUSG00000036352	Ubac1	1.03	6.67E-09	3.11E-08
2196	ENSMUSG00000028541	B4galt2	-1.58	6.70E-09	3.12E-08
2197	ENSMUSG00000042298	Ttc19	1.03	6.71E-09	3.12E-08

2198	ENSMUSG00000032777	Gtf3c1	-1.24	6.88E-09	3.20E-08
2199	ENSMUSG00000032518	Rpsa	1.05	7.03E-09	3.27E-08
2200	ENSMUSG00000004931	Apba3	1.08	7.07E-09	3.29E-08
2201	ENSMUSG00000040488	Ltbp4	-1.07	7.14E-09	3.32E-08
2202	ENSMUSG00000024158	Hagh	1.00	7.15E-09	3.32E-08
2203	ENSMUSG00000056692	D17Wsu92e	0.95	7.16E-09	3.32E-08
2204	ENSMUSG00000039339	Mfsd4b2	1.53	7.20E-09	3.34E-08
2205	ENSMUSG00000041445	Mmrn2	-1.72	7.26E-09	3.37E-08
2206	ENSMUSG00000027993	Trim2	1.75	7.31E-09	3.39E-08
2207	ENSMUSG00000020964	Sel1l	-1.12	7.36E-09	3.41E-08
2208	ENSMUSG00000096981	Gm16845	1.13	7.40E-09	3.43E-08
2209	ENSMUSG00000057841	Rpl32	1.22	7.41E-09	3.43E-08
2210	ENSMUSG00000005374	Tbl2	-1.18	7.45E-09	3.45E-08
2211	ENSMUSG00000051730	Mettl5	1.28	7.50E-09	3.47E-08
2212	ENSMUSG00000038695	Josd2	1.39	7.55E-09	3.49E-08
2213	ENSMUSG00000021420	Fars2	0.98	7.57E-09	3.50E-08
2214	ENSMUSG00000021069	Pygl	1.18	7.57E-09	3.50E-08
2215	ENSMUSG00000068735	Trp53i11	-1.85	7.66E-09	3.54E-08
2216	ENSMUSG00000031505	Naxd	1.06	7.69E-09	3.55E-08
2217	ENSMUSG00000025967	Eef1b2	1.22	7.73E-09	3.57E-08
2218	ENSMUSG00000050891	Tatdn1	1.44	7.74E-09	3.57E-08
2219	ENSMUSG00000024163	Mapk8ip3	1.01	7.76E-09	3.58E-08
2220	ENSMUSG00000075254	Heg1	-1.01	7.77E-09	3.58E-08
2221	ENSMUSG00000005360	Slc1a3	-1.82	7.77E-09	3.58E-08
2222	ENSMUSG00000024594	Prrc1	-1.24	7.87E-09	3.62E-08
2223	ENSMUSG00000015289	Lage3	1.11	7.88E-09	3.62E-08
2224	ENSMUSG00000024750	Zfand5	-0.97	8.08E-09	3.71E-08
2225	ENSMUSG00000001158	Snrnp27	1.33	8.08E-09	3.71E-08
2226	ENSMUSG00000040532	Abhd11	1.11	8.18E-09	3.76E-08
2227	ENSMUSG00000022801	Lrch3	-1.35	8.22E-09	3.78E-08
2228	ENSMUSG00000019261	Map1s	-1.41	8.28E-09	3.80E-08
2229	ENSMUSG00000090330	9130221H12Rik	1.09	8.42E-09	3.86E-08
2230	ENSMUSG00000023106	Denr	1.07	8.43E-09	3.87E-08
2231	ENSMUSG00000097617	Gm10687	-1.87	8.46E-09	3.88E-08
2232	ENSMUSG00000020828	Pld2	-1.59	8.46E-09	3.88E-08
2233	ENSMUSG00000089647	Gm2245	1.49	8.47E-09	3.88E-08
2234	ENSMUSG00000020661	Dnmt3a	-1.22	8.49E-09	3.89E-08
2235	ENSMUSG00000021660	Btf3	1.07	8.56E-09	3.92E-08
2236	ENSMUSG00000093593	Gm20683	-0.96	8.58E-09	3.92E-08
2237	ENSMUSG00000021902	Phf7	1.45	8.60E-09	3.93E-08
2238	ENSMUSG00000111685	AC124577.4	-1.34	8.66E-09	3.96E-08
2239	ENSMUSG00000027001	Dusp19	1.41	8.72E-09	3.98E-08
2240	ENSMUSG00000046364	Rpl27a	1.39	8.73E-09	3.99E-08
2241	ENSMUSG00000021375	Kif13a	-1.27	8.80E-09	4.02E-08
2242	ENSMUSG00000022708	Zbtb20	-0.99	8.90E-09	4.06E-08
2243	ENSMUSG00000006958	Chrd	-2.15	9.04E-09	4.12E-08
2244	ENSMUSG00000057880	Abat	-1.92	9.11E-09	4.15E-08
2245	ENSMUSG00000060679	Mrps9	1.06	9.14E-09	4.16E-08
2246	ENSMUSG00000025231	Sufu	-1.31	9.15E-09	4.17E-08
2247	ENSMUSG00000020372	Rack1	1.07	9.19E-09	4.18E-08

2248	ENSMUSG00000049858	Suox	0.98	9.35E-09	4.25E-08
2249	ENSMUSG00000073468	Sft2d1	1.11	9.35E-09	4.25E-08
2250	ENSMUSG00000027651	Rprd1b	-1.06	9.46E-09	4.30E-08
2251	ENSMUSG00000050708	Ftl1	1.08	9.52E-09	4.32E-08
2252	ENSMUSG00000006412	Pfdn2	1.09	9.61E-09	4.36E-08
2253	ENSMUSG00000020459	Mtif2	1.19	9.65E-09	4.38E-08
2254	ENSMUSG00000063382	Bcl9l	-1.23	9.68E-09	4.39E-08
2255	ENSMUSG00000021417	Eci2	0.96	9.80E-09	4.44E-08
2256	ENSMUSG00000025479	Cyp2e1	-2.43	9.81E-09	4.45E-08
2257	ENSMUSG00000024424	Ttc39c	-1.42	9.87E-09	4.47E-08
2258	ENSMUSG00000000628	Hk2	0.98	9.89E-09	4.48E-08
2259	ENSMUSG00000051379	Flrt3	-1.66	1.02E-08	4.60E-08
2260	ENSMUSG00000024827	Gldc	-1.65	1.02E-08	4.60E-08
2261	ENSMUSG00000030161	Gabarapl1	1.03	1.02E-08	4.63E-08
2262	ENSMUSG00000025036	Sfxn2	-1.73	1.02E-08	4.63E-08
2263	ENSMUSG00000021190	Lgmn	1.17	1.04E-08	4.68E-08
2264	ENSMUSG00000027944	Hax1	0.99	1.05E-08	4.75E-08
2265	ENSMUSG00000024985	Tcf7l2	-1.57	1.06E-08	4.77E-08
2266	ENSMUSG00000031672	Got2	1.14	1.06E-08	4.78E-08
2267	ENSMUSG00000027078	Ube2l6	-1.34	1.07E-08	4.81E-08
2268	ENSMUSG00000024516	Sec11c	1.27	1.07E-08	4.83E-08
2269	ENSMUSG00000019947	Arid5b	-1.35	1.07E-08	4.83E-08
2270	ENSMUSG00000032562	Gnai2	-1.01	1.08E-08	4.86E-08
2271	ENSMUSG00000031555	Adam9	-1.03	1.08E-08	4.86E-08
2272	ENSMUSG00000028416	Bag1	0.96	1.09E-08	4.92E-08
2273	ENSMUSG00000025780	Itih5	-0.97	1.09E-08	4.92E-08
2274	ENSMUSG00000029715	Pop7	1.40	1.10E-08	4.94E-08
2275	ENSMUSG00000031681	Smad1	-1.25	1.11E-08	4.97E-08
2276	ENSMUSG00000020790	Ankfy1	-1.33	1.12E-08	5.01E-08
2277	ENSMUSG00000066149	Cdc26	1.31	1.12E-08	5.02E-08
2278	ENSMUSG00000028690	Mmachc	0.98	1.12E-08	5.04E-08
2279	ENSMUSG00000066571	4931406P16Rik	-1.43	1.12E-08	5.05E-08
2280	ENSMUSG00000030538	Cib1	1.21	1.13E-08	5.05E-08
2281	ENSMUSG00000093701	Gm21970	0.94	1.13E-08	5.06E-08
2282	ENSMUSG00000022559	Fbxl6	1.34	1.13E-08	5.07E-08
2283	ENSMUSG00000079555	Haus3	1.43	1.14E-08	5.11E-08
2284	ENSMUSG00000015133	Lrrk1	-1.74	1.14E-08	5.12E-08
2285	ENSMUSG00000036550	Cnot1	-1.31	1.18E-08	5.29E-08
2286	ENSMUSG00000037411	Serpine1	-1.93	1.18E-08	5.30E-08
2287	ENSMUSG00000037643	Prkci	-1.38	1.19E-08	5.30E-08
2288	ENSMUSG00000020346	Mgat1	-1.04	1.19E-08	5.33E-08
2289	ENSMUSG00000063558	Aox1	-1.07	1.19E-08	5.33E-08
2290	ENSMUSG00000005373	Mlxipl	0.97	1.20E-08	5.34E-08
2291	ENSMUSG00000074151	Nlrc5	-1.61	1.20E-08	5.34E-08
2292	ENSMUSG00000027806	Tsc22d2	-1.28	1.20E-08	5.36E-08
2293	ENSMUSG00000044667	Plppr4	-1.41	1.20E-08	5.36E-08
2294	ENSMUSG00000020380	Rad50	-1.74	1.21E-08	5.38E-08
2295	ENSMUSG00000014355	Anapc1	-1.08	1.21E-08	5.38E-08
2296	ENSMUSG00000043822	Adamtsl5	1.60	1.21E-08	5.39E-08
2297	ENSMUSG00000022361	Zhx1	-1.05	1.21E-08	5.40E-08

2298	ENSMUSG00000072612	Gm10382	-1.05	1.22E-08	5.42E-08
2299	ENSMUSG00000082815	Gm5678	1.31	1.22E-08	5.44E-08
2300	ENSMUSG00000032263	Bckdhb	0.93	1.22E-08	5.44E-08
2301	ENSMUSG00000073471	Rsph3a	1.04	1.23E-08	5.45E-08
2302	ENSMUSG00000024805	Pcgf5	1.10	1.23E-08	5.45E-08
2303	ENSMUSG00000026553	Copa	-1.17	1.24E-08	5.49E-08
2304	ENSMUSG00000033065	Pfkm	1.05	1.24E-08	5.49E-08
2305	ENSMUSG00000032081	Apoc3	1.54	1.25E-08	5.56E-08
2306	ENSMUSG00000091512	Lamtor3	1.14	1.25E-08	5.56E-08
2307	ENSMUSG00000008036	Ap2s1	1.00	1.26E-08	5.58E-08
2308	ENSMUSG00000075705	Msrb1	1.04	1.27E-08	5.62E-08
2309	ENSMUSG00000020456	Ogdh	1.00	1.28E-08	5.65E-08
2310	ENSMUSG00000085095	Gm15635	-1.59	1.29E-08	5.70E-08
2311	ENSMUSG00000071415	Rpl23	1.14	1.29E-08	5.70E-08
2312	ENSMUSG00000079677	Fdx1l	1.13	1.29E-08	5.72E-08
2313	ENSMUSG00000085962	Gm16984	0.97	1.29E-08	5.72E-08
2314	ENSMUSG00000002897	Il17ra	-1.64	1.30E-08	5.72E-08
2315	ENSMUSG00000003380	Rabac1	1.00	1.30E-08	5.73E-08
2316	ENSMUSG00000037266	Rsrp1	1.38	1.31E-08	5.76E-08
2317	ENSMUSG00000052889	Prkcb	-1.90	1.31E-08	5.77E-08
2318	ENSMUSG00000004070	Hmox2	1.03	1.31E-08	5.77E-08
2319	ENSMUSG00000020534	Shmt1	1.16	1.31E-08	5.77E-08
2320	ENSMUSG00000037972	Snn	-1.49	1.36E-08	5.98E-08
2321	ENSMUSG00000032504	Pdcd6ip	-1.01	1.39E-08	6.14E-08
2322	ENSMUSG00000041417	Pik3r1	-1.45	1.41E-08	6.21E-08
2323	ENSMUSG00000019173	Rab5c	1.27	1.42E-08	6.26E-08
2324	ENSMUSG00000050545	Fam228b	1.74	1.42E-08	6.26E-08
2325	ENSMUSG00000046909	Tefm	1.08	1.43E-08	6.29E-08
2326	ENSMUSG00000031639	Tlr3	-1.63	1.43E-08	6.31E-08
2327	ENSMUSG00000074886	Grk6	-1.22	1.45E-08	6.38E-08
2328	ENSMUSG00000022560	Slc52a2	1.04	1.46E-08	6.40E-08
2329	ENSMUSG00000004631	Sgce	-1.31	1.46E-08	6.40E-08
2330	ENSMUSG00000059970	Hspa2	-1.35	1.47E-08	6.43E-08
2331	ENSMUSG00000024726	Carnmt1	0.97	1.48E-08	6.48E-08
2332	ENSMUSG00000024645	Timm21	1.13	1.48E-08	6.51E-08
2333	ENSMUSG00000070699	Sars2	1.12	1.49E-08	6.55E-08
2334	ENSMUSG00000005981	Trap1	-0.93	1.51E-08	6.62E-08
2335	ENSMUSG00000026202	Tuba4a	1.17	1.52E-08	6.67E-08
2336	ENSMUSG00000047907	Tshz2	-1.30	1.53E-08	6.71E-08
2337	ENSMUSG00000057098	Ebf1	-0.99	1.55E-08	6.77E-08
2338	ENSMUSG00000016194	Hsd11b1	1.21	1.55E-08	6.80E-08
2339	ENSMUSG00000030782	Tgfb1i1	-1.56	1.57E-08	6.85E-08
2340	ENSMUSG00000028487	Bnc2	-1.72	1.57E-08	6.86E-08
2341	ENSMUSG00000024601	Isoc1	-0.93	1.58E-08	6.89E-08
2342	ENSMUSG00000109967	Gm45883	-1.39	1.58E-08	6.90E-08
2343	ENSMUSG00000031799	Tpm4	-1.18	1.59E-08	6.93E-08
2344	ENSMUSG00000091625	Lsm5	1.56	1.59E-08	6.93E-08
2345	ENSMUSG00000021716	Srek1ip1	1.41	1.60E-08	6.97E-08
2346	ENSMUSG00000027230	Creb3l1	-1.63	1.61E-08	7.00E-08
2347	ENSMUSG00000028140	Mrpl9	0.95	1.61E-08	7.02E-08

2348	ENSMUSG00000020105	Lrig3	-1.45	1.61E-08	7.03E-08
2349	ENSMUSG00000096972	Gm26883	-1.45	1.62E-08	7.04E-08
2350	ENSMUSG00000087684	1200007C13Rik	-1.96	1.62E-08	7.04E-08
2351	ENSMUSG00000029528	Pxn	-1.31	1.62E-08	7.07E-08
2352	ENSMUSG00000062762	Ei24	0.94	1.63E-08	7.07E-08
2353	ENSMUSG00000028962	Slc4a2	-1.10	1.64E-08	7.11E-08
2354	ENSMUSG00000034247	Plekhm1	-1.04	1.65E-08	7.15E-08
2355	ENSMUSG00000105504	Gbp5	-1.38	1.65E-08	7.17E-08
2356	ENSMUSG00000070738	Dgkd	-1.10	1.66E-08	7.21E-08
2357	ENSMUSG00000021408	Ripk1	-1.66	1.67E-08	7.24E-08
2358	ENSMUSG00000038612	Mcl1	-1.06	1.67E-08	7.26E-08
2359	ENSMUSG00000025135	Anapc11	1.24	1.69E-08	7.30E-08
2360	ENSMUSG00000014226	Cacybp	0.95	1.69E-08	7.33E-08
2361	ENSMUSG00000037010	Apln	-1.65	1.70E-08	7.36E-08
2362	ENSMUSG00000031429	Psmd10	1.12	1.71E-08	7.40E-08
2363	ENSMUSG00000068240	Gm11808	1.53	1.72E-08	7.43E-08
2364	ENSMUSG00000029038	Ssu72	1.00	1.75E-08	7.56E-08
2365	ENSMUSG00000013698	Pea15a	-1.10	1.75E-08	7.58E-08
2366	ENSMUSG00000021930	Spryd7	0.97	1.76E-08	7.59E-08
2367	ENSMUSG00000020087	Tysnd1	1.00	1.76E-08	7.60E-08
2368	ENSMUSG00000038712	Fam63a	-1.03	1.76E-08	7.60E-08
2369	ENSMUSG00000020536	Llgl1	-1.32	1.76E-08	7.60E-08
2370	ENSMUSG00000110350	Gm10252	1.01	1.76E-08	7.60E-08
2371	ENSMUSG00000037938	Chchd5	1.17	1.76E-08	7.60E-08
2372	ENSMUSG00000031536	Polb	1.13	1.77E-08	7.61E-08
2373	ENSMUSG00000040952	Rps19	0.99	1.77E-08	7.61E-08
2374	ENSMUSG00000058704	Memo1	0.96	1.77E-08	7.62E-08
2375	ENSMUSG00000024772	Ehd1	-0.94	1.78E-08	7.68E-08
2376	ENSMUSG00000111905	AC153962.1	1.21	1.78E-08	7.68E-08
2377	ENSMUSG00000018040	Rrp7a	0.96	1.79E-08	7.68E-08
2378	ENSMUSG00000030748	Il4ra	-1.45	1.79E-08	7.71E-08
2379	ENSMUSG00000079020	Slc45a4	-1.30	1.80E-08	7.72E-08
2380	ENSMUSG00000009549	Srp14	1.08	1.80E-08	7.72E-08
2381	ENSMUSG00000026887	Mrrf	0.99	1.80E-08	7.72E-08
2382	ENSMUSG00000003438	Timm50	0.92	1.80E-08	7.72E-08
2383	ENSMUSG00000026219	Trip12	-1.01	1.80E-08	7.74E-08
2384	ENSMUSG00000000942	Hoxa4	1.47	1.83E-08	7.84E-08
2385	ENSMUSG00000037316	Bag4	-1.14	1.83E-08	7.85E-08
2386	ENSMUSG00000050663	Trhde	-2.12	1.83E-08	7.85E-08
2387	ENSMUSG00000018559	Ctdnep1	1.28	1.85E-08	7.91E-08
2388	ENSMUSG00000027087	Itgav	-1.36	1.85E-08	7.94E-08
2389	ENSMUSG00000045257	Morn2	1.44	1.86E-08	7.96E-08
2390	ENSMUSG00000040562	Gstm2	-0.97	1.86E-08	7.97E-08
2391	ENSMUSG00000058407	Txndc9	1.02	1.86E-08	7.97E-08
2392	ENSMUSG00000086709	Gm16263	-1.54	1.87E-08	7.97E-08
2393	ENSMUSG00000102869	2900097C17Rik	-1.14	1.87E-08	7.99E-08
2394	ENSMUSG00000028044	Cks1b	-1.37	1.89E-08	8.07E-08
2395	ENSMUSG00000021288	Klc1	-1.27	1.90E-08	8.09E-08
2396	ENSMUSG00000042485	Mustn1	-1.67	1.92E-08	8.19E-08
2397	ENSMUSG00000010054	Tusc2	0.94	1.93E-08	8.25E-08

2398	ENSMUSG00000037992	Rara	-1.52	1.95E-08	8.31E-08
2399	ENSMUSG00000058444	Map2k5	1.00	1.96E-08	8.37E-08
2400	ENSMUSG00000026162	Nhej1	1.35	1.96E-08	8.37E-08
2401	ENSMUSG00000031246	Sh3bgrl	0.93	1.98E-08	8.43E-08
2402	ENSMUSG00000022607	Ptk2	-1.14	1.99E-08	8.47E-08
2403	ENSMUSG00000015970	Chdh	-1.01	2.00E-08	8.50E-08
2404	ENSMUSG00000016128	Stard13	-1.62	2.00E-08	8.50E-08
2405	ENSMUSG00000043284	Tmem11	0.93	2.00E-08	8.50E-08
2406	ENSMUSG00000063550	Nup98	-1.28	2.01E-08	8.55E-08
2407	ENSMUSG00000038178	Slc43a2	1.38	2.01E-08	8.55E-08
2408	ENSMUSG00000031808	Slc27a1	-1.52	2.04E-08	8.67E-08
2409	ENSMUSG00000028890	Mtf1	1.01	2.06E-08	8.73E-08
2410	ENSMUSG00000021959	Lats2	-1.12	2.06E-08	8.73E-08
2411	ENSMUSG00000044709	Gemin7	0.93	2.06E-08	8.73E-08
2412	ENSMUSG00000024855	Pacs1	-1.27	2.07E-08	8.78E-08
2413	ENSMUSG00000103041	Gm37305	-1.40	2.08E-08	8.80E-08
2414	ENSMUSG00000033444	Specc1l	-1.24	2.08E-08	8.80E-08
2415	ENSMUSG00000032413	Rasa2	-1.48	2.12E-08	8.97E-08
2416	ENSMUSG00000002058	Unc119	-1.23	2.13E-08	9.00E-08
2417	ENSMUSG00000033039	Micall1	-1.02	2.15E-08	9.11E-08
2418	ENSMUSG00000038722	Bud31	1.02	2.16E-08	9.11E-08
2419	ENSMUSG00000025817	Nudt5	0.93	2.16E-08	9.15E-08
2420	ENSMUSG00000038582	Pptc7	0.97	2.18E-08	9.19E-08
2421	ENSMUSG00000027889	Ampd2	-1.15	2.19E-08	9.25E-08
2422	ENSMUSG00000025903	Lypla1	0.95	2.21E-08	9.33E-08
2423	ENSMUSG00000020705	Ddx42	-1.22	2.22E-08	9.35E-08
2424	ENSMUSG00000022799	Arhgap31	-1.19	2.24E-08	9.46E-08
2425	ENSMUSG00000028789	Azin2	-1.34	2.24E-08	9.46E-08
2426	ENSMUSG00000001525	Tubb5	-1.24	2.25E-08	9.50E-08
2427	ENSMUSG00000031783	Polr2c	0.93	2.26E-08	9.53E-08
2428	ENSMUSG00000091811	Inafm1	1.16	2.28E-08	9.62E-08
2429	ENSMUSG00000015943	Bola1	1.15	2.30E-08	9.68E-08
2430	ENSMUSG00000006057	Atp5g1	-1.37	2.30E-08	9.70E-08
2431	ENSMUSG00000027610	Gss	1.06	2.31E-08	9.73E-08
2432	ENSMUSG00000033955	Tnks1bp1	-1.05	2.32E-08	9.74E-08
2433	ENSMUSG00000030605	Mfge8	-0.91	2.33E-08	9.77E-08
2434	ENSMUSG00000021208	Ifi27l2b	-1.73	2.33E-08	9.79E-08
2435	ENSMUSG00000008348	Ubc	-0.95	2.34E-08	9.81E-08
2436	ENSMUSG00000030110	Ret	-1.77	2.34E-08	9.82E-08
2437	ENSMUSG00000033004	Mycbp2	-1.25	2.35E-08	9.84E-08
2438	ENSMUSG00000026547	Tagln2	-1.52	2.35E-08	9.85E-08
2439	ENSMUSG00000038060	Dlec1	1.14	2.35E-08	9.85E-08
2440	ENSMUSG00000016559	H3f3b	-0.96	2.36E-08	9.90E-08
2441	ENSMUSG00000026965	Anapc2	-1.00	2.42E-08	1.02E-07
2442	ENSMUSG00000069874	Irgm2	-1.68	2.43E-08	1.02E-07
2443	ENSMUSG00000062647	Rpl7a	1.02	2.44E-08	1.02E-07
2444	ENSMUSG00000025130	P4hb	-1.25	2.45E-08	1.02E-07
2445	ENSMUSG00000030213	Atf7ip	-1.25	2.46E-08	1.03E-07
2446	ENSMUSG00000035559	Mpv17l2	0.96	2.46E-08	1.03E-07
2447	ENSMUSG00000019433	Gipc1	1.04	2.52E-08	1.05E-07

2448	ENSMUSG00000027007	Ssfa2	0.92	2.54E-08	1.06E-07
2449	ENSMUSG00000041879	Ipo9	-1.23	2.55E-08	1.06E-07
2450	ENSMUSG00000060143	Gm10076	1.14	2.55E-08	1.07E-07
2451	ENSMUSG00000022677	Fopnl	1.02	2.55E-08	1.07E-07
2452	ENSMUSG00000039318	Rab3gap2	-1.22	2.56E-08	1.07E-07
2453	ENSMUSG00000024851	Pitpnm1	-1.28	2.58E-08	1.08E-07
2454	ENSMUSG00000050248	Evc2	-1.68	2.59E-08	1.08E-07
2455	ENSMUSG00000020700	Map3k3	-1.08	2.59E-08	1.08E-07
2456	ENSMUSG00000005683	Cs	1.02	2.63E-08	1.10E-07
2457	ENSMUSG00000034487	Kdelc2	-1.47	2.65E-08	1.10E-07
2458	ENSMUSG00000003948	Mmd	-1.23	2.67E-08	1.11E-07
2459	ENSMUSG00000038422	Hdhd3	1.00	2.69E-08	1.12E-07
2460	ENSMUSG00000099568	Gm28513	-1.60	2.70E-08	1.12E-07
2461	ENSMUSG00000036639	Nudt1	1.33	2.72E-08	1.13E-07
2462	ENSMUSG00000029312	Klhl8	-1.41	2.74E-08	1.14E-07
2463	ENSMUSG00000025260	Hsd17b10	0.92	2.74E-08	1.14E-07
2464	ENSMUSG00000071713	Csf2rb	-1.55	2.77E-08	1.15E-07
2465	ENSMUSG00000005886	Ncoa2	-1.29	2.77E-08	1.15E-07
2466	ENSMUSG00000026112	Coa5	1.08	2.78E-08	1.15E-07
2467	ENSMUSG00000053559	Smagp	1.30	2.79E-08	1.16E-07
2468	ENSMUSG00000022205	Sub1	1.01	2.81E-08	1.16E-07
2469	ENSMUSG00000060019	Gm10073	1.37	2.81E-08	1.16E-07
2470	ENSMUSG00000062526	Mppe1	1.18	2.84E-08	1.17E-07
2471	ENSMUSG00000039031	Arhgap18	-1.74	2.86E-08	1.18E-07
2472	ENSMUSG00000041949	Tango6	-1.28	2.86E-08	1.19E-07
2473	ENSMUSG00000028944	Prkag2	-1.05	2.88E-08	1.19E-07
2474	ENSMUSG00000025733	Rhot2	1.02	2.88E-08	1.19E-07
2475	ENSMUSG00000014776	Nol3	1.17	2.90E-08	1.20E-07
2476	ENSMUSG00000104713	Gbp6	-1.10	2.93E-08	1.21E-07
2477	ENSMUSG00000044288	Cnr1	-1.58	2.96E-08	1.22E-07
2478	ENSMUSG00000098332	2310009A05Rik	1.36	2.96E-08	1.22E-07
2479	ENSMUSG00000016018	Skiv2l2	-1.10	2.96E-08	1.22E-07
2480	ENSMUSG00000004837	Grap	-1.40	2.98E-08	1.23E-07
2481	ENSMUSG00000005973	Rcn1	-1.37	2.98E-08	1.23E-07
2482	ENSMUSG00000027435	Cd93	-1.53	2.99E-08	1.23E-07
2483	ENSMUSG00000047067	Dusp28	1.05	3.02E-08	1.24E-07
2484	ENSMUSG00000029446	Psph	1.05	3.03E-08	1.25E-07
2485	ENSMUSG00000037410	Tbc1d2b	-1.75	3.03E-08	1.25E-07
2486	ENSMUSG00000045411	2410002F23Rik	-1.47	3.04E-08	1.25E-07
2487	ENSMUSG00000060803	Gstp1	0.95	3.05E-08	1.25E-07
2488	ENSMUSG00000029033	Acap3	-1.36	3.09E-08	1.27E-07
2489	ENSMUSG00000028420	Tmem38b	1.15	3.15E-08	1.30E-07
2490	ENSMUSG00000024527	Afg3l2	0.92	3.19E-08	1.31E-07
2491	ENSMUSG00000025616	Usp16	1.05	3.21E-08	1.32E-07
2492	ENSMUSG00000042549	Map2k3os	1.54	3.21E-08	1.32E-07
2493	ENSMUSG00000017314	Mpp2	-1.31	3.21E-08	1.32E-07
2494	ENSMUSG00000029675	Eln	-1.63	3.23E-08	1.32E-07
2495	ENSMUSG00000034022	Cpsf1	-1.02	3.24E-08	1.33E-07
2496	ENSMUSG00000028530	Jak1	-1.05	3.26E-08	1.33E-07
2497	ENSMUSG00000111692	AC163637.2	1.09	3.29E-08	1.35E-07

2498	ENSMUSG00000031365	Zfp275	-1.68	3.30E-08	1.35E-07
2499	ENSMUSG00000034424	Gcsh	0.91	3.32E-08	1.36E-07
2500	ENSMUSG00000001768	Rin2	-1.18	3.32E-08	1.36E-07
2501	ENSMUSG00000043391	2510009E07Rik	-1.22	3.33E-08	1.36E-07
2502	ENSMUSG00000016528	Mapkapk2	0.89	3.34E-08	1.36E-07
2503	ENSMUSG00000084863	Gm12523	-1.48	3.38E-08	1.38E-07
2504	ENSMUSG00000072946	Ptgr2	0.89	3.40E-08	1.39E-07
2505	ENSMUSG00000039037	St6galnac5	-2.07	3.43E-08	1.40E-07
2506	ENSMUSG00000000154	Slc22a18	-1.52	3.45E-08	1.41E-07
2507	ENSMUSG00000009614	Sardh	-0.99	3.47E-08	1.41E-07
2508	ENSMUSG00000010651	Acaa1b	0.99	3.53E-08	1.44E-07
2509	ENSMUSG00000033029	1700088E04Rik	-1.13	3.53E-08	1.44E-07
2510	ENSMUSG00000003038	Hmgn2	-1.02	3.55E-08	1.45E-07
2511	ENSMUSG00000031386	Hcfc1	-1.12	3.56E-08	1.45E-07
2512	ENSMUSG00000038155	Gstp2	0.92	3.56E-08	1.45E-07
2513	ENSMUSG00000085241	Snhg3	1.47	3.57E-08	1.45E-07
2514	ENSMUSG00000026853	Crat	0.93	3.58E-08	1.46E-07
2515	ENSMUSG00000021238	Aldh6a1	-1.18	3.63E-08	1.48E-07
2516	ENSMUSG00000049502	Dtx3l	-1.38	3.64E-08	1.48E-07
2517	ENSMUSG00000040822	1700123O20Rik	0.98	3.65E-08	1.48E-07
2518	ENSMUSG00000026172	Bcs1l	1.02	3.65E-08	1.48E-07
2519	ENSMUSG00000040033	Stat2	-1.59	3.67E-08	1.49E-07
2520	ENSMUSG00000025902	Sox17	-1.46	3.67E-08	1.49E-07
2521	ENSMUSG00000025226	Fbxl15	1.24	3.69E-08	1.50E-07
2522	ENSMUSG00000017679	Ttpal	-1.33	3.75E-08	1.52E-07
2523	ENSMUSG00000059602	Syn3	-1.09	3.78E-08	1.53E-07
2524	ENSMUSG00000046330	Rpl37a	1.27	3.80E-08	1.54E-07
2525	ENSMUSG00000057265	Bbof1	-1.12	3.84E-08	1.56E-07
2526	ENSMUSG00000041638	Gcn1l1	-1.10	3.88E-08	1.57E-07
2527	ENSMUSG00000052102	Gnpda1	1.14	3.91E-08	1.58E-07
2528	ENSMUSG00000038909	Kat7	-0.96	3.93E-08	1.59E-07
2529	ENSMUSG00000037470	Uggt1	-1.15	3.95E-08	1.60E-07
2530	ENSMUSG00000025869	Nop16	1.05	3.96E-08	1.60E-07
2531	ENSMUSG00000032086	Bace1	-1.05	3.96E-08	1.60E-07
2532	ENSMUSG00000032120	C2cd2l	-1.35	4.04E-08	1.63E-07
2533	ENSMUSG00000087679	C330006A16Rik	0.96	4.04E-08	1.63E-07
2534	ENSMUSG00000031963	Bmper	-1.51	4.13E-08	1.67E-07
2535	ENSMUSG00000020402	Vdac1	0.99	4.16E-08	1.68E-07
2536	ENSMUSG00000024420	Zfp521	-1.48	4.19E-08	1.69E-07
2537	ENSMUSG00000033335	Dnm2	-0.90	4.20E-08	1.69E-07
2538	ENSMUSG00000031820	Babam1	0.90	4.23E-08	1.70E-07
2539	ENSMUSG00000033157	Abhd10	1.21	4.23E-08	1.70E-07
2540	ENSMUSG00000028607	Cpt2	0.89	4.25E-08	1.71E-07
2541	ENSMUSG00000039005	Tlr4	-1.40	4.26E-08	1.72E-07
2542	ENSMUSG00000020806	Rhbdf2	-1.38	4.31E-08	1.73E-07
2543	ENSMUSG00000028398	Tmem261	1.21	4.31E-08	1.73E-07
2544	ENSMUSG00000038518	Jarid2	-1.47	4.31E-08	1.73E-07
2545	ENSMUSG00000097216	4932441J04Rik	-1.52	4.32E-08	1.74E-07
2546	ENSMUSG00000031996	Aplp2	-1.04	4.36E-08	1.75E-07
2547	ENSMUSG00000037119	Fam91a1	-1.24	4.37E-08	1.76E-07

2548	ENSMUSG00000031613	Hpgd	-1.31	4.41E-08	1.77E-07
2549	ENSMUSG00000023307	March5	0.90	4.56E-08	1.83E-07
2550	ENSMUSG00000020116	Pno1	1.01	4.57E-08	1.83E-07
2551	ENSMUSG00000030058	Copg1	-0.97	4.63E-08	1.85E-07
2552	ENSMUSG00000024187	Fam234a	0.90	4.66E-08	1.87E-07
2553	ENSMUSG00000001794	Capns1	0.97	4.67E-08	1.87E-07
2554	ENSMUSG00000032125	Robo4	-1.37	4.67E-08	1.87E-07
2555	ENSMUSG00000021917	Spcs1	0.95	4.69E-08	1.88E-07
2556	ENSMUSG00000048240	Gng7	-1.28	4.70E-08	1.88E-07
2557	ENSMUSG00000031485	Prosc	0.93	4.71E-08	1.88E-07
2558	ENSMUSG00000003346	Abhd17a	0.88	4.76E-08	1.90E-07
2559	ENSMUSG00000085829	Gm4285	1.43	4.76E-08	1.90E-07
2560	ENSMUSG00000030759	Far1	-1.34	4.79E-08	1.91E-07
2561	ENSMUSG00000079480	Pin4	1.30	4.80E-08	1.92E-07
2562	ENSMUSG00000028382	Ptbp3	-1.00	4.80E-08	1.92E-07
2563	ENSMUSG00000025920	Stau2	0.99	4.82E-08	1.92E-07
2564	ENSMUSG00000026773	Pfkfb3	-0.89	4.83E-08	1.93E-07
2565	ENSMUSG00000057963	Itpk1	1.06	4.87E-08	1.94E-07
2566	ENSMUSG00000046387	Pcdhb17	-1.45	4.87E-08	1.94E-07
2567	ENSMUSG00000034101	Ctnnd1	-1.06	4.88E-08	1.94E-07
2568	ENSMUSG00000059263	Usp47	-0.95	4.91E-08	1.95E-07
2569	ENSMUSG00000038046	Mrm3	1.04	4.91E-08	1.95E-07
2570	ENSMUSG00000063179	Pstk	1.08	4.91E-08	1.95E-07
2571	ENSMUSG00000018593	Sparc	-0.93	4.93E-08	1.96E-07
2572	ENSMUSG00000026924	Sec16a	-1.32	5.07E-08	2.02E-07
2573	ENSMUSG00000062198	2700097O09Rik	1.42	5.11E-08	2.03E-07
2574	ENSMUSG00000022781	Pak2	-1.01	5.11E-08	2.03E-07
2575	ENSMUSG00000087436	Gm16156	-1.52	5.12E-08	2.03E-07
2576	ENSMUSG00000068749	Psma5	0.95	5.13E-08	2.04E-07
2577	ENSMUSG00000002458	Rgs19	-1.11	5.13E-08	2.04E-07
2578	ENSMUSG00000090266	Mettl23	1.03	5.14E-08	2.04E-07
2579	ENSMUSG00000012848	Rps5	1.05	5.16E-08	2.05E-07
2580	ENSMUSG00000029426	Scarb2	0.92	5.20E-08	2.06E-07
2581	ENSMUSG00000028639	Ybx1	0.87	5.23E-08	2.07E-07
2582	ENSMUSG00000002147	Stat6	-0.89	5.24E-08	2.08E-07
2583	ENSMUSG00000001156	Mxd1	-1.37	5.25E-08	2.08E-07
2584	ENSMUSG00000042520	Ubap2l	-0.92	5.26E-08	2.08E-07
2585	ENSMUSG00000068036	Afdn	-1.18	5.29E-08	2.09E-07
2586	ENSMUSG00000024668	Sdhaf2	0.87	5.34E-08	2.11E-07
2587	ENSMUSG00000038507	Parp12	-1.22	5.34E-08	2.11E-07
2588	ENSMUSG00000026632	Tatdn3	1.41	5.37E-08	2.12E-07
2589	ENSMUSG00000030447	Cyfip1	-0.98	5.40E-08	2.13E-07
2590	ENSMUSG00000018340	Anxa6	-0.87	5.41E-08	2.14E-07
2591	ENSMUSG00000020009	Ifngr1	-1.06	5.42E-08	2.14E-07
2592	ENSMUSG00000005686	Ampd3	1.08	5.46E-08	2.15E-07
2593	ENSMUSG00000091021	Gm17300	1.47	5.48E-08	2.16E-07
2594	ENSMUSG00000105987	AI506816	-1.28	5.52E-08	2.17E-07
2595	ENSMUSG00000026753	Ppp6c	0.87	5.52E-08	2.17E-07
2596	ENSMUSG00000029502	Golga3	-1.19	5.52E-08	2.17E-07
2597	ENSMUSG00000113311	CT009486.1	1.29	5.59E-08	2.20E-07

2598	ENSMUSG00000037112	Sik2	-0.95	5.59E-08	2.20E-07
2599	ENSMUSG00000034723	Tmx4	1.08	5.65E-08	2.22E-07
2600	ENSMUSG00000031988	Vps26b	-1.13	5.75E-08	2.26E-07
2601	ENSMUSG00000031722	Hp	-0.98	5.75E-08	2.26E-07
2602	ENSMUSG00000049090	Zadh2	0.92	5.77E-08	2.27E-07
2603	ENSMUSG00000001946	Esam	-1.14	5.78E-08	2.27E-07
2604	ENSMUSG00000028525	Pde4b	-1.38	5.79E-08	2.27E-07
2605	ENSMUSG00000031534	Smim19	0.93	5.81E-08	2.28E-07
2606	ENSMUSG00000103380	Gm37756	-1.08	5.81E-08	2.28E-07
2607	ENSMUSG00000034908	Sidt2	-1.14	5.82E-08	2.28E-07
2608	ENSMUSG00000024580	Grpel2	1.08	5.90E-08	2.31E-07
2609	ENSMUSG00000021832	Psmc6	0.93	5.90E-08	2.31E-07
2610	ENSMUSG00000039384	Dusp10	-1.13	5.92E-08	2.32E-07
2611	ENSMUSG00000022474	Pmm1	0.97	5.93E-08	2.32E-07
2612	ENSMUSG00000043518	Rai2	-1.27	5.97E-08	2.34E-07
2613	ENSMUSG00000028551	Cdkn2c	-1.00	6.03E-08	2.36E-07
2614	ENSMUSG00000021037	Ahsa1	0.98	6.04E-08	2.36E-07
2615	ENSMUSG00000040037	Negr1	-1.09	6.09E-08	2.38E-07
2616	ENSMUSG00000021576	Pdcd6	0.91	6.11E-08	2.39E-07
2617	ENSMUSG00000025347	Mettl7b	-1.60	6.16E-08	2.41E-07
2618	ENSMUSG00000002781	Tmem143	0.89	6.17E-08	2.41E-07
2619	ENSMUSG00000029430	Ran	0.89	6.19E-08	2.42E-07
2620	ENSMUSG00000020440	Arf5	0.89	6.22E-08	2.43E-07
2621	ENSMUSG00000049354	Dcaf7	-1.06	6.26E-08	2.44E-07
2622	ENSMUSG00000027439	Gzf1	-1.10	6.28E-08	2.45E-07
2623	ENSMUSG00000019861	Gopc	-1.10	6.32E-08	2.46E-07
2624	ENSMUSG00000027088	Phospho2	1.12	6.36E-08	2.48E-07
2625	ENSMUSG00000032536	Trak1	-0.99	6.44E-08	2.51E-07
2626	ENSMUSG00000055322	Tns1	-0.91	6.45E-08	2.51E-07
2627	ENSMUSG00000079362	Gm43302	-1.04	6.52E-08	2.54E-07
2628	ENSMUSG00000003531	Dgcr6	0.90	6.54E-08	2.55E-07
2629	ENSMUSG00000047473	Zfp30	1.39	6.56E-08	2.55E-07
2630	ENSMUSG00000049299	Trappc1	0.99	6.56E-08	2.55E-07
2631	ENSMUSG00000020388	Pdlim4	-1.37	6.58E-08	2.56E-07
2632	ENSMUSG00000011884	Gltp	-0.92	6.59E-08	2.56E-07
2633	ENSMUSG00000028760	Eif4g3	-1.07	6.61E-08	2.57E-07
2634	ENSMUSG00000022309	Angpt1	-1.49	6.63E-08	2.57E-07
2635	ENSMUSG00000041734	Kirrel	-1.57	6.71E-08	2.60E-07
2636	ENSMUSG00000020190	Mknk2	-0.95	6.75E-08	2.62E-07
2637	ENSMUSG00000024081	Cebpz	1.09	6.85E-08	2.66E-07
2638	ENSMUSG00000042099	Kank3	-1.16	6.87E-08	2.66E-07
2639	ENSMUSG00000006058	Snf8	0.93	6.90E-08	2.67E-07
2640	ENSMUSG00000063060	Sox7	-1.26	6.93E-08	2.68E-07
2641	ENSMUSG00000028614	Ndc1	-1.32	6.96E-08	2.70E-07
2642	ENSMUSG00000022706	Mrpl40	1.01	6.98E-08	2.70E-07
2643	ENSMUSG00000017466	Timp2	-1.91	7.02E-08	2.72E-07
2644	ENSMUSG00000032673	Prorsd1	1.14	7.04E-08	2.72E-07
2645	ENSMUSG00000039656	Rxrb	0.93	7.04E-08	2.72E-07
2646	ENSMUSG00000064080	Fbln2	-1.50	7.05E-08	2.73E-07
2647	ENSMUSG00000022842	Ece2	1.23	7.06E-08	2.73E-07

2648	ENSMUSG00000015083	C8g	1.21	7.09E-08	2.74E-07
2649	ENSMUSG00000003923	Tfam	1.09	7.09E-08	2.74E-07
2650	ENSMUSG00000043716	Rpl7	1.01	7.10E-08	2.74E-07
2651	ENSMUSG00000027245	Hypk	1.05	7.14E-08	2.75E-07
2652	ENSMUSG00000032035	Ets1	-1.12	7.15E-08	2.76E-07
2653	ENSMUSG00000031972	Acta1	-1.55	7.16E-08	2.76E-07
2654	ENSMUSG00000038459	Abhd17c	0.99	7.19E-08	2.77E-07
2655	ENSMUSG00000032563	Mrpl3	0.94	7.19E-08	2.77E-07
2656	ENSMUSG00000036026	Tmem63b	0.92	7.25E-08	2.79E-07
2657	ENSMUSG00000037204	Atg101	1.01	7.29E-08	2.81E-07
2658	ENSMUSG00000027882	Stxbp3	-1.05	7.30E-08	2.81E-07
2659	ENSMUSG00000047557	Lxn	-1.49	7.32E-08	2.81E-07
2660	ENSMUSG00000020577	Tspan13	-1.19	7.33E-08	2.82E-07
2661	ENSMUSG00000020018	Snrpf	1.19	7.35E-08	2.82E-07
2662	ENSMUSG00000014791	Elmo3	-1.43	7.36E-08	2.83E-07
2663	ENSMUSG00000021710	Nln	-0.93	7.37E-08	2.83E-07
2664	ENSMUSG00000050002	Idnk	0.98	7.47E-08	2.87E-07
2665	ENSMUSG00000012483	Rpa3	1.60	7.57E-08	2.90E-07
2666	ENSMUSG00000053841	Txlna	-1.27	7.71E-08	2.96E-07
2667	ENSMUSG00000025436	Atp23	1.37	7.75E-08	2.97E-07
2668	ENSMUSG00000026176	Ctdsp1	1.25	7.83E-08	3.00E-07
2669	ENSMUSG00000064267	Hvcn1	-1.57	7.84E-08	3.01E-07
2670	ENSMUSG00000024533	Spire1	0.98	7.86E-08	3.01E-07
2671	ENSMUSG00000021807	2700060E02Rik	0.90	7.88E-08	3.02E-07
2672	ENSMUSG00000029843	Slc13a4	-1.61	7.88E-08	3.02E-07
2673	ENSMUSG00000030917	Tmem159	-1.09	8.03E-08	3.07E-07
2674	ENSMUSG00000046324	Ermp1	-1.41	8.06E-08	3.08E-07
2675	ENSMUSG00000029851	Tcaf2	-1.52	8.06E-08	3.08E-07
2676	ENSMUSG00000058672	Tubb2a	-1.39	8.10E-08	3.10E-07
2677	ENSMUSG00000029821	Dfna5	-1.39	8.11E-08	3.10E-07
2678	ENSMUSG00000087331	1810021B22Rik	1.17	8.12E-08	3.10E-07
2679	ENSMUSG00000026790	Odf2	-1.32	8.13E-08	3.10E-07
2680	ENSMUSG00000018401	Mtmr4	-1.33	8.15E-08	3.11E-07
2681	ENSMUSG00000020038	Cry1	-1.47	8.23E-08	3.14E-07
2682	ENSMUSG00000045875	Adra1a	1.10	8.24E-08	3.14E-07
2683	ENSMUSG00000011832	Evi5l	-1.02	8.32E-08	3.17E-07
2684	ENSMUSG00000055319	Sec23ip	-1.06	8.34E-08	3.18E-07
2685	ENSMUSG00000033453	Adamts15	-1.85	8.35E-08	3.18E-07
2686	ENSMUSG00000024259	Slc25a46	0.86	8.40E-08	3.20E-07
2687	ENSMUSG00000055762	Eef1d	1.07	8.53E-08	3.25E-07
2688	ENSMUSG00000042462	Dctpp1	1.26	8.65E-08	3.29E-07
2689	ENSMUSG00000050103	Agmo	-0.99	8.78E-08	3.34E-07
2690	ENSMUSG00000041633	Kctd12b	-1.31	8.80E-08	3.35E-07
2691	ENSMUSG00000085236	2610206C17Rik	1.03	8.81E-08	3.35E-07
2692	ENSMUSG00000034168	Irf2bpl	-1.40	8.81E-08	3.35E-07
2693	ENSMUSG00000022462	Slc38a2	-1.01	8.83E-08	3.35E-07
2694	ENSMUSG00000090035	Galnt4	-1.39	8.97E-08	3.40E-07
2695	ENSMUSG00000015291	Gdi1	-0.96	9.01E-08	3.42E-07
2696	ENSMUSG00000027573	Gid8	0.97	9.04E-08	3.43E-07
2697	ENSMUSG00000031980	Agt	-1.99	9.06E-08	3.44E-07

2698	ENSMUSG00000032763	Ilvbl	0.94	9.16E-08	3.47E-07
2699	ENSMUSG00000054808	Actn4	-0.84	9.20E-08	3.49E-07
2700	ENSMUSG00000097185	Gm26596	0.98	9.25E-08	3.50E-07
2701	ENSMUSG00000021910	Nisch	-0.85	9.27E-08	3.51E-07
2702	ENSMUSG00000029701	Rbm28	-1.17	9.30E-08	3.52E-07
2703	ENSMUSG00000004530	Coro1c	-1.07	9.39E-08	3.55E-07
2704	ENSMUSG00000095687	Rnaset2a	1.09	9.40E-08	3.55E-07
2705	ENSMUSG00000048787	Dcun1d3	-1.14	9.50E-08	3.59E-07
2706	ENSMUSG00000025224	Gbf1	-0.92	9.51E-08	3.60E-07
2707	ENSMUSG00000023951	Vegfa	-0.92	9.58E-08	3.62E-07
2708	ENSMUSG00000029465	Arpc3	0.90	9.60E-08	3.63E-07
2709	ENSMUSG00000033287	Kctd17	-0.95	9.64E-08	3.64E-07
2710	ENSMUSG00000035357	Pdzrn3	-1.37	9.64E-08	3.64E-07
2711	ENSMUSG00000071653	1810009A15Rik	1.04	9.67E-08	3.65E-07
2712	ENSMUSG00000031838	Ifi30	1.48	9.67E-08	3.65E-07
2713	ENSMUSG00000027940	Tpm3	-0.91	9.84E-08	3.71E-07
2714	ENSMUSG00000079588	Tmem182	-1.14	9.86E-08	3.72E-07
2715	ENSMUSG00000029185	Fam114a1	-1.43	9.87E-08	3.72E-07
2716	ENSMUSG00000001855	Nup214	-1.16	1.00E-07	3.77E-07
2717	ENSMUSG00000032437	Stt3b	-1.23	1.01E-07	3.80E-07
2718	ENSMUSG00000021794	Glud1	-0.87	1.01E-07	3.81E-07
2719	ENSMUSG00000021453	Gadd45g	-1.17	1.02E-07	3.82E-07
2720	ENSMUSG00000090639	Gm20425	-2.45	1.02E-07	3.83E-07
2721	ENSMUSG00000020733	Slc9a3r1	1.39	1.02E-07	3.84E-07
2722	ENSMUSG00000021598	Med10	1.09	1.03E-07	3.88E-07
2723	ENSMUSG00000097033	Gm26819	1.09	1.03E-07	3.88E-07
2724	ENSMUSG00000049076	Acap2	-1.46	1.03E-07	3.88E-07
2725	ENSMUSG00000017707	Serinc3	-0.96	1.03E-07	3.88E-07
2726	ENSMUSG00000023150	Ivns1abp	-1.02	1.04E-07	3.88E-07
2727	ENSMUSG00000019437	Tlcd1	0.90	1.04E-07	3.90E-07
2728	ENSMUSG00000037419	Endod1	-1.33	1.04E-07	3.91E-07
2729	ENSMUSG00000071451	Psmg4	1.27	1.05E-07	3.93E-07
2730	ENSMUSG00000027067	Ssrp1	-0.89	1.05E-07	3.94E-07
2731	ENSMUSG00000032867	Fbxw8	-0.99	1.05E-07	3.94E-07
2732	ENSMUSG00000069844	Sco1	0.98	1.06E-07	3.97E-07
2733	ENSMUSG00000059013	Sh2d3c	-1.38	1.06E-07	3.97E-07
2734	ENSMUSG00000006611	Hfe	-0.89	1.07E-07	4.01E-07
2735	ENSMUSG00000020955	Ap4s1	1.10	1.08E-07	4.04E-07
2736	ENSMUSG00000022312	Eif3h	0.92	1.09E-07	4.06E-07
2737	ENSMUSG00000020189	Osbpl8	-1.17	1.09E-07	4.06E-07
2738	ENSMUSG00000031458	Coprs	1.63	1.09E-07	4.06E-07
2739	ENSMUSG00000028961	Pgd	1.07	1.09E-07	4.07E-07
2740	ENSMUSG00000034345	Gtf2h5	0.98	1.09E-07	4.07E-07
2741	ENSMUSG00000033278	Ptprm	-1.00	1.10E-07	4.08E-07
2742	ENSMUSG00000027787	Nmd3	0.94	1.10E-07	4.09E-07
2743	ENSMUSG00000039929	Urb1	1.02	1.10E-07	4.11E-07
2744	ENSMUSG00000039982	Dtx4	-2.42	1.10E-07	4.11E-07
2745	ENSMUSG00000024958	Gpr137	0.92	1.11E-07	4.12E-07
2746	ENSMUSG00000050212	Eva1b	-1.35	1.11E-07	4.12E-07
2747	ENSMUSG00000054920	Klhl5	-0.98	1.11E-07	4.13E-07

2748	ENSMUSG00000020986	Sec23a	-1.17	1.11E-07	4.13E-07
2749	ENSMUSG00000022174	Dad1	0.87	1.11E-07	4.14E-07
2750	ENSMUSG00000022890	Atp5j	0.87	1.12E-07	4.17E-07
2751	ENSMUSG00000020023	Tmcc3	-1.04	1.12E-07	4.17E-07
2752	ENSMUSG00000054204	Fam150b	-1.43	1.12E-07	4.17E-07
2753	ENSMUSG00000029250	Polr2b	-0.99	1.12E-07	4.17E-07
2754	ENSMUSG00000042594	Sh2b3	-1.15	1.15E-07	4.25E-07
2755	ENSMUSG00000032554	Trf	-2.48	1.16E-07	4.30E-07
2756	ENSMUSG00000009376	Met	-1.18	1.16E-07	4.31E-07
2757	ENSMUSG00000059854	Hydin	-1.30	1.17E-07	4.35E-07
2758	ENSMUSG00000028063	Lmna	-0.83	1.19E-07	4.40E-07
2759	ENSMUSG00000041958	Pigs	-0.93	1.20E-07	4.43E-07
2760	ENSMUSG00000024121	Atp6v0c	0.86	1.20E-07	4.43E-07
2761	ENSMUSG00000053714	4732471J01Rik	-0.94	1.20E-07	4.45E-07
2762	ENSMUSG00000026701	Prdx6	0.94	1.21E-07	4.48E-07
2763	ENSMUSG00000020458	Rtn4	0.85	1.21E-07	4.48E-07
2764	ENSMUSG00000053477	Tcf4	-0.94	1.21E-07	4.48E-07
2765	ENSMUSG00000078153	Psme2b	1.15	1.22E-07	4.50E-07
2766	ENSMUSG00000037499	Nenf	1.07	1.22E-07	4.50E-07
2767	ENSMUSG00000022340	Sybu	-1.31	1.23E-07	4.53E-07
2768	ENSMUSG00000025809	Itgb1	-0.95	1.23E-07	4.53E-07
2769	ENSMUSG00000074781	Ube2n	0.91	1.23E-07	4.53E-07
2770	ENSMUSG00000063897	DHRSX	0.99	1.23E-07	4.55E-07
2771	ENSMUSG00000044018	Mrpl50	0.89	1.24E-07	4.56E-07
2772	ENSMUSG00000028910	Mecr	0.98	1.24E-07	4.57E-07
2773	ENSMUSG00000050896	Rtn4rl2	-1.59	1.24E-07	4.58E-07
2774	ENSMUSG00000100017	2410022M11Rik	1.25	1.25E-07	4.59E-07
2775	ENSMUSG00000107335	Gm43372	1.06	1.25E-07	4.59E-07
2776	ENSMUSG00000015027	Galns	-1.44	1.25E-07	4.61E-07
2777	ENSMUSG00000036299	BC031181	0.89	1.25E-07	4.61E-07
2778	ENSMUSG00000018697	Aatf	1.22	1.26E-07	4.65E-07
2779	ENSMUSG00000024077	Strn	-1.15	1.28E-07	4.70E-07
2780	ENSMUSG00000086359	9630013K17Rik	1.27	1.28E-07	4.70E-07
2781	ENSMUSG00000026473	Glul	0.87	1.29E-07	4.73E-07
2782	ENSMUSG00000032320	Rcn2	-0.92	1.29E-07	4.75E-07
2783	ENSMUSG00000072964	Bhlhb9	-1.26	1.30E-07	4.76E-07
2784	ENSMUSG00000026604	Ptpn14	-1.32	1.30E-07	4.76E-07
2785	ENSMUSG00000022885	St6gal1	1.57	1.30E-07	4.76E-07
2786	ENSMUSG00000063884	Ptcd3	0.89	1.30E-07	4.77E-07
2787	ENSMUSG00000061950	Ppp4r1	-1.01	1.30E-07	4.77E-07
2788	ENSMUSG00000033161	Atp1a1	1.28	1.30E-07	4.77E-07
2789	ENSMUSG00000089766	Gm16538	1.17	1.31E-07	4.79E-07
2790	ENSMUSG00000042451	Mybph	-1.28	1.31E-07	4.79E-07
2791	ENSMUSG00000093661	Eif4e3	0.92	1.31E-07	4.80E-07
2792	ENSMUSG00000020821	Kif1c	1.23	1.32E-07	4.82E-07
2793	ENSMUSG00000029600	Rita1	1.16	1.32E-07	4.83E-07
2794	ENSMUSG00000028793	Rnf19b	-1.16	1.32E-07	4.84E-07
2795	ENSMUSG00000047126	Cltc	-0.92	1.33E-07	4.86E-07
2796	ENSMUSG00000000295	Hddc2	1.07	1.33E-07	4.87E-07
2797	ENSMUSG00000032741	Tpcn1	1.11	1.34E-07	4.89E-07

2798	ENSMUSG00000078870	Gm14410	1.27	1.35E-07	4.91E-07
2799	ENSMUSG00000032997	Chpf	-0.93	1.35E-07	4.92E-07
2800	ENSMUSG00000039328	Rnf122	-1.44	1.35E-07	4.93E-07
2801	ENSMUSG00000045838	A430105I19Rik	-1.43	1.37E-07	5.01E-07
2802	ENSMUSG00000003660	Snrnp200	-1.08	1.38E-07	5.02E-07
2803	ENSMUSG00000031839	Hsbp1	0.86	1.38E-07	5.04E-07
2804	ENSMUSG00000034858	Fam214a	-1.08	1.39E-07	5.05E-07
2805	ENSMUSG00000020623	Map2k6	-1.61	1.39E-07	5.08E-07
2806	ENSMUSG00000010376	Nedd8	1.02	1.40E-07	5.08E-07
2807	ENSMUSG00000033209	Ttc28	-1.10	1.40E-07	5.09E-07
2808	ENSMUSG00000042747	Krtcap2	1.01	1.40E-07	5.10E-07
2809	ENSMUSG00000005505	Kbtbd4	0.88	1.40E-07	5.10E-07
2810	ENSMUSG00000045827	Serpinb9	-1.65	1.41E-07	5.13E-07
2811	ENSMUSG00000045983	Eif4g1	-0.90	1.41E-07	5.13E-07
2812	ENSMUSG00000008450	Nutf2	0.92	1.41E-07	5.14E-07
2813	ENSMUSG00000017485	Top2b	-0.98	1.42E-07	5.17E-07
2814	ENSMUSG00000093752	Gm20716	0.86	1.42E-07	5.17E-07
2815	ENSMUSG00000023074	Mospd1	0.87	1.42E-07	5.17E-07
2816	ENSMUSG00000037211	Spry1	-1.29	1.43E-07	5.19E-07
2817	ENSMUSG00000066456	Hmgn3	-1.41	1.43E-07	5.20E-07
2818	ENSMUSG00000032412	Atp1b3	0.86	1.46E-07	5.30E-07
2819	ENSMUSG00000040957	Cables1	0.99	1.46E-07	5.30E-07
2820	ENSMUSG00000000581	C1d	0.95	1.47E-07	5.31E-07
2821	ENSMUSG00000040560	Wdr7	-1.15	1.47E-07	5.33E-07
2822	ENSMUSG00000063652	Slc22a21	0.98	1.49E-07	5.40E-07
2823	ENSMUSG00000049516	Spty2d1	-1.16	1.50E-07	5.44E-07
2824	ENSMUSG00000004043	Stat5a	-0.96	1.53E-07	5.52E-07
2825	ENSMUSG00000039081	Zfp503	-1.43	1.53E-07	5.52E-07
2826	ENSMUSG00000063172	Hspb11	1.37	1.54E-07	5.56E-07
2827	ENSMUSG00000029090	Adgra3	-1.22	1.54E-07	5.57E-07
2828	ENSMUSG00000078517	Emc1	-0.89	1.54E-07	5.58E-07
2829	ENSMUSG00000035504	Reep6	1.10	1.55E-07	5.58E-07
2830	ENSMUSG00000036986	Pml	-1.45	1.56E-07	5.62E-07
2831	ENSMUSG00000054181	A930012O16Rik	1.26	1.56E-07	5.62E-07
2832	ENSMUSG00000024082	Ndufaf7	0.86	1.56E-07	5.64E-07
2833	ENSMUSG00000055943	Emc7	0.86	1.57E-07	5.66E-07
2834	ENSMUSG00000038982	Bloc1s5	0.89	1.58E-07	5.70E-07
2835	ENSMUSG00000032301	Psma4	0.84	1.60E-07	5.75E-07
2836	ENSMUSG00000028631	Kcnq4	-1.63	1.60E-07	5.76E-07
2837	ENSMUSG00000061731	Ext1	-1.19	1.60E-07	5.76E-07
2838	ENSMUSG00000030244	Gys2	1.06	1.60E-07	5.78E-07
2839	ENSMUSG00000078812	Eif5a	0.85	1.60E-07	5.78E-07
2840	ENSMUSG00000028647	Mycbp	0.95	1.61E-07	5.78E-07
2841	ENSMUSG00000032905	Atg12	0.84	1.62E-07	5.83E-07
2842	ENSMUSG00000028443	Nudt2	1.04	1.63E-07	5.85E-07
2843	ENSMUSG00000097101	1810034E14Rik	1.27	1.64E-07	5.88E-07
2844	ENSMUSG00000002325	Irf9	-1.24	1.65E-07	5.93E-07
2845	ENSMUSG00000026797	Stxbp1	-1.54	1.65E-07	5.93E-07
2846	ENSMUSG00000036845	Lin37	1.26	1.66E-07	5.95E-07
2847	ENSMUSG00000003731	Kpna6	-0.94	1.66E-07	5.95E-07

2848	ENSMUSG00000017309	Cd300lg	-1.02	1.66E-07	5.97E-07
2849	ENSMUSG00000025198	Erlin1	-1.39	1.66E-07	5.97E-07
2850	ENSMUSG00000026640	Plxna2	-1.02	1.67E-07	5.98E-07
2851	ENSMUSG00000028833	Ncdn	-1.12	1.67E-07	6.00E-07
2852	ENSMUSG00000045100	Slc25a26	1.04	1.68E-07	6.02E-07
2853	ENSMUSG00000026527	Rgs7	0.98	1.68E-07	6.02E-07
2854	ENSMUSG00000107169	Gm43796	0.84	1.68E-07	6.02E-07
2855	ENSMUSG00000039345	Mettl22	1.01	1.68E-07	6.02E-07
2856	ENSMUSG00000006301	Tmbim1	-0.97	1.68E-07	6.03E-07
2857	ENSMUSG00000017491	Rarb	1.60	1.69E-07	6.03E-07
2858	ENSMUSG00000046460	Sh2d7	1.38	1.69E-07	6.04E-07
2859	ENSMUSG00000021240	Abcd4	0.96	1.70E-07	6.08E-07
2860	ENSMUSG00000003199	Mpnd	0.87	1.70E-07	6.08E-07
2861	ENSMUSG00000026311	Asb1	-1.26	1.71E-07	6.11E-07
2862	ENSMUSG00000021737	Psmd6	0.82	1.71E-07	6.11E-07
2863	ENSMUSG00000085255	Taco1os	1.37	1.72E-07	6.15E-07
2864	ENSMUSG00000086753	Gm15751	0.96	1.75E-07	6.23E-07
2865	ENSMUSG00000018196	Glrx2	0.93	1.75E-07	6.24E-07
2866	ENSMUSG00000027075	Slc43a1	-1.71	1.76E-07	6.28E-07
2867	ENSMUSG00000036644	Tbc1d9b	-0.92	1.76E-07	6.29E-07
2868	ENSMUSG00000005481	Ddx39	-0.90	1.77E-07	6.32E-07
2869	ENSMUSG00000050043	Tmx2	0.83	1.78E-07	6.33E-07
2870	ENSMUSG00000014361	Mertk	-1.52	1.78E-07	6.36E-07
2871	ENSMUSG00000003762	Coq8b	-0.88	1.79E-07	6.36E-07
2872	ENSMUSG00000020589	Fam49a	-1.02	1.80E-07	6.42E-07
2873	ENSMUSG00000097761	4930534D22Rik	1.24	1.82E-07	6.47E-07
2874	ENSMUSG00000097675	1700101I11Rik	0.92	1.84E-07	6.56E-07
2875	ENSMUSG00000027200	Sema6d	-1.55	1.86E-07	6.60E-07
2876	ENSMUSG00000041571	Selenow	1.04	1.87E-07	6.63E-07
2877	ENSMUSG00000031154	Otud5	-1.27	1.89E-07	6.72E-07
2878	ENSMUSG00000057766	Ankrd29	-1.39	1.91E-07	6.78E-07
2879	ENSMUSG00000108282	Gm44317	-1.34	1.91E-07	6.78E-07
2880	ENSMUSG00000020056	Washc3	0.96	1.91E-07	6.79E-07
2881	ENSMUSG00000021840	Mapk1ip1l	-0.98	1.92E-07	6.82E-07
2882	ENSMUSG00000074643	Cpne1	-0.96	1.92E-07	6.82E-07
2883	ENSMUSG00000026567	Adcy10	1.47	1.93E-07	6.84E-07
2884	ENSMUSG00000016918	Sulf1	-1.44	2.00E-07	7.08E-07
2885	ENSMUSG00000022476	Polr3h	0.86	2.02E-07	7.16E-07
2886	ENSMUSG00000021036	Sptlc2	-1.08	2.02E-07	7.16E-07
2887	ENSMUSG00000025492	Ifitm3	-0.90	2.03E-07	7.20E-07
2888	ENSMUSG00000042215	Bag2	1.03	2.04E-07	7.21E-07
2889	ENSMUSG00000036955	Kif1bp	-0.89	2.04E-07	7.24E-07
2890	ENSMUSG00000005871	Apc	-1.13	2.05E-07	7.24E-07
2891	ENSMUSG00000012405	Rpl15	0.95	2.06E-07	7.30E-07
2892	ENSMUSG00000028969	Cdk5	0.91	2.07E-07	7.32E-07
2893	ENSMUSG00000000339	Rtca	0.93	2.07E-07	7.33E-07
2894	ENSMUSG00000056851	Pcbp2	0.92	2.07E-07	7.33E-07
2895	ENSMUSG00000018648	Dusp14	1.47	2.08E-07	7.34E-07
2896	ENSMUSG00000045410	Akr1e1	0.90	2.11E-07	7.47E-07
2897	ENSMUSG00000048371	Pdp2	0.94	2.12E-07	7.47E-07

2898	ENSMUSG00000036249	Rbm43	-1.30	2.12E-07	7.47E-07
2899	ENSMUSG00000032249	Anp32a	1.26	2.13E-07	7.51E-07
2900	ENSMUSG00000024232	Bambi	-1.41	2.14E-07	7.53E-07
2901	ENSMUSG00000031149	Praf2	1.16	2.15E-07	7.58E-07
2902	ENSMUSG00000085118	Gm15774	-1.37	2.15E-07	7.59E-07
2903	ENSMUSG00000033068	Entpd6	-1.07	2.16E-07	7.60E-07
2904	ENSMUSG00000046470	Sox18	-1.05	2.16E-07	7.61E-07
2905	ENSMUSG00000028088	Fmo5	-1.10	2.17E-07	7.63E-07
2906	ENSMUSG00000001150	Mcm3ap	-1.16	2.17E-07	7.64E-07
2907	ENSMUSG00000050195	Scd4	-1.44	2.17E-07	7.64E-07
2908	ENSMUSG00000044562	Rasip1	-1.08	2.19E-07	7.71E-07
2909	ENSMUSG00000038267	Slc22a23	-1.09	2.20E-07	7.72E-07
2910	ENSMUSG00000004558	Ndrg2	0.81	2.20E-07	7.74E-07
2911	ENSMUSG00000027775	Mfsd1	-0.86	2.22E-07	7.80E-07
2912	ENSMUSG00000027303	Ptpra	-0.88	2.23E-07	7.81E-07
2913	ENSMUSG00000027465	Tbc1d20	0.81	2.23E-07	7.81E-07
2914	ENSMUSG00000020634	Ubxn2a	0.85	2.23E-07	7.81E-07
2915	ENSMUSG00000089715	Cbx6	-1.48	2.24E-07	7.85E-07
2916	ENSMUSG00000096606	Tpbgl	1.06	2.24E-07	7.86E-07
2917	ENSMUSG00000062960	Kdr	-1.03	2.25E-07	7.90E-07
2918	ENSMUSG00000026155	Smap1	0.94	2.26E-07	7.92E-07
2919	ENSMUSG00000028173	Wls	-0.85	2.27E-07	7.96E-07
2920	ENSMUSG00000022687	Boc	-1.54	2.28E-07	8.00E-07
2921	ENSMUSG00000052560	Cpne8	-1.21	2.32E-07	8.11E-07
2922	ENSMUSG00000031539	Ap3m2	-1.24	2.34E-07	8.20E-07
2923	ENSMUSG00000032002	Dcun1d5	0.86	2.35E-07	8.22E-07
2924	ENSMUSG00000025144	Cenpx	0.93	2.38E-07	8.33E-07
2925	ENSMUSG00000017837	Nkiras2	0.82	2.39E-07	8.36E-07
2926	ENSMUSG00000019808	Adat2	1.10	2.41E-07	8.43E-07
2927	ENSMUSG00000047719	Ubiad1	0.91	2.44E-07	8.52E-07
2928	ENSMUSG00000042599	Kdm7a	-1.18	2.45E-07	8.57E-07
2929	ENSMUSG00000024070	Prkd3	-1.47	2.45E-07	8.57E-07
2930	ENSMUSG00000026181	Ppm1f	-0.97	2.46E-07	8.59E-07
2931	ENSMUSG00000026103	Gls	-1.13	2.47E-07	8.61E-07
2932	ENSMUSG00000031015	Swap70	-1.43	2.50E-07	8.72E-07
2933	ENSMUSG00000079593	Gm14597	-1.44	2.50E-07	8.72E-07
2934	ENSMUSG00000037977	6430571L13Rik	1.26	2.54E-07	8.84E-07
2935	ENSMUSG00000019951	Uhrf1bp1l	-0.97	2.54E-07	8.84E-07
2936	ENSMUSG00000028991	Mtor	0.81	2.54E-07	8.86E-07
2937	ENSMUSG00000028266	Lmo4	-0.99	2.55E-07	8.86E-07
2938	ENSMUSG00000038393	Txnip	-0.92	2.55E-07	8.86E-07
2939	ENSMUSG00000051256	Jagn1	0.82	2.58E-07	8.97E-07
2940	ENSMUSG00000096974	Gm26881	1.38	2.59E-07	9.00E-07
2941	ENSMUSG00000052085	Dock8	1.14	2.59E-07	9.00E-07
2942	ENSMUSG00000030849	Fgfr2	-1.30	2.59E-07	9.00E-07
2943	ENSMUSG00000021701	Plk2	-1.28	2.59E-07	9.01E-07
2944	ENSMUSG00000026544	Dusp23	1.03	2.59E-07	9.01E-07
2945	ENSMUSG00000036712	Cyld	-1.16	2.60E-07	9.01E-07
2946	ENSMUSG00000028156	Eif4e	0.82	2.61E-07	9.04E-07
2947	ENSMUSG00000074398	Gm15441	-1.00	2.61E-07	9.04E-07

2948	ENSMUSG00000034462	Pkd2	-1.21	2.61E-07	9.06E-07
2949	ENSMUSG00000037787	Apopt1	0.86	2.63E-07	9.12E-07
2950	ENSMUSG00000064326	Siva1	0.96	2.63E-07	9.12E-07
2951	ENSMUSG00000060802	B2m	-1.03	2.64E-07	9.13E-07
2952	ENSMUSG00000024695	Zfp91	0.88	2.67E-07	9.24E-07
2953	ENSMUSG00000004947	Dtx2	-0.97	2.67E-07	9.24E-07
2954	ENSMUSG00000033809	Alg3	0.86	2.69E-07	9.30E-07
2955	ENSMUSG00000023960	Enpp5	-1.02	2.69E-07	9.30E-07
2956	ENSMUSG00000052299	Ltn1	-0.97	2.69E-07	9.30E-07
2957	ENSMUSG00000047712	Ust	-1.19	2.70E-07	9.34E-07
2958	ENSMUSG00000035944	Ttc38	0.91	2.73E-07	9.42E-07
2959	ENSMUSG00000003382	Etv3	-1.13	2.74E-07	9.48E-07
2960	ENSMUSG00000027314	Dll4	-1.29	2.75E-07	9.49E-07
2961	ENSMUSG00000035342	Lzts2	-0.98	2.75E-07	9.49E-07
2962	ENSMUSG00000054708	Ankrd24	1.17	2.77E-07	9.57E-07
2963	ENSMUSG00000036748	Cuedc2	0.89	2.78E-07	9.59E-07
2964	ENSMUSG00000026029	Casp8	-1.06	2.78E-07	9.59E-07
2965	ENSMUSG00000030922	Lyrm1	1.23	2.78E-07	9.59E-07
2966	ENSMUSG00000024990	Rbp4	-1.05	2.80E-07	9.64E-07
2967	ENSMUSG00000020412	Ascc2	-1.05	2.80E-07	9.64E-07
2968	ENSMUSG00000054277	Arfgap3	-1.23	2.80E-07	9.64E-07
2969	ENSMUSG00000079283	2310009B15Rik	1.29	2.80E-07	9.65E-07
2970	ENSMUSG00000025202	Scd3	-1.53	2.81E-07	9.66E-07
2971	ENSMUSG00000024944	Arl2	0.96	2.81E-07	9.67E-07
2972	ENSMUSG00000037012	Hk1	1.29	2.82E-07	9.69E-07
2973	ENSMUSG00000097681	Gm26643	0.91	2.82E-07	9.69E-07
2974	ENSMUSG00000032841	Prr5l	-1.60	2.85E-07	9.78E-07
2975	ENSMUSG00000022323	Rida	0.90	2.85E-07	9.79E-07
2976	ENSMUSG00000071724	Smpd5	0.87	2.87E-07	9.86E-07
2977	ENSMUSG00000032497	Lrrfip2	0.92	2.87E-07	9.86E-07
2978	ENSMUSG00000029195	Klb	-1.27	2.87E-07	9.86E-07
2979	ENSMUSG00000039840	Epg5	-1.23	2.88E-07	9.89E-07
2980	ENSMUSG00000040820	Hlcs	-1.51	2.89E-07	9.90E-07
2981	ENSMUSG00000003458	Ncstn	-1.01	2.89E-07	9.91E-07
2982	ENSMUSG00000020553	Pctp	1.06	2.89E-07	9.92E-07
2983	ENSMUSG00000029106	Add1	-0.86	2.91E-07	9.98E-07
2984	ENSMUSG00000025076	Casp7	-1.43	2.91E-07	9.98E-07
2985	ENSMUSG00000017999	Ddx27	-1.02	2.91E-07	9.98E-07
2986	ENSMUSG00000055681	Cope	0.87	2.95E-07	1.01E-06
2987	ENSMUSG00000036932	Aifm1	0.85	2.95E-07	1.01E-06
2988	ENSMUSG00000031865	Dctn1	-0.90	2.96E-07	1.01E-06
2989	ENSMUSG00000070407	Hs3st3b1	-1.74	2.97E-07	1.02E-06
2990	ENSMUSG00000055413	H2-Q5	-1.07	2.99E-07	1.02E-06
2991	ENSMUSG00000022744	Cldnd1	0.88	3.00E-07	1.02E-06
2992	ENSMUSG00000023905	Tnfrsf12a	1.08	3.00E-07	1.03E-06
2993	ENSMUSG00000067389	Gm17080	1.46	3.02E-07	1.03E-06
2994	ENSMUSG00000020059	Sycp3	0.81	3.02E-07	1.03E-06
2995	ENSMUSG00000015961	Adss	0.91	3.03E-07	1.03E-06
2996	ENSMUSG00000031708	Tecr	0.86	3.04E-07	1.04E-06
2997	ENSMUSG00000026273	Mterf4	1.00	3.04E-07	1.04E-06

2998	ENSMUSG00000025937	Lactb2	0.87	3.05E-07	1.04E-06
2999	ENSMUSG00000009647	Mcu	1.08	3.05E-07	1.04E-06
3000	ENSMUSG00000041453	Rpl21	0.93	3.07E-07	1.05E-06
3001	ENSMUSG00000028066	Pmf1	0.90	3.07E-07	1.05E-06
3002	ENSMUSG00000037461	Ints7	-1.04	3.08E-07	1.05E-06
3003	ENSMUSG00000072582	Ptrh2	1.04	3.09E-07	1.05E-06
3004	ENSMUSG00000023830	Igf2r	-1.20	3.13E-07	1.07E-06
3005	ENSMUSG00000000555	Itga5	-1.43	3.13E-07	1.07E-06
3006	ENSMUSG00000040016	Ptger3	-1.27	3.13E-07	1.07E-06
3007	ENSMUSG00000063802	Hspbp1	0.94	3.14E-07	1.07E-06
3008	ENSMUSG00000022299	Slc25a32	1.00	3.14E-07	1.07E-06
3009	ENSMUSG00000025203	Scd2	-1.44	3.14E-07	1.07E-06
3010	ENSMUSG00000006498	Ptbp1	-0.94	3.17E-07	1.08E-06
3011	ENSMUSG00000027602	Map1lc3a	0.88	3.22E-07	1.09E-06
3012	ENSMUSG00000036168	Ccdc38	1.14	3.23E-07	1.10E-06
3013	ENSMUSG00000024294	Mib1	-1.26	3.23E-07	1.10E-06
3014	ENSMUSG00000019235	Rps6kl1	1.31	3.26E-07	1.10E-06
3015	ENSMUSG00000026389	Steap3	-0.95	3.27E-07	1.11E-06
3016	ENSMUSG00000090069	E430024P14Rik	0.95	3.27E-07	1.11E-06
3017	ENSMUSG00000030249	Abcc9	-1.44	3.30E-07	1.12E-06
3018	ENSMUSG00000032913	Lrig2	-1.28	3.31E-07	1.12E-06
3019	ENSMUSG00000001707	Eef1e1	1.04	3.32E-07	1.13E-06
3020	ENSMUSG00000016496	Cd274	-1.05	3.36E-07	1.14E-06
3021	ENSMUSG00000030094	Xpc	-1.23	3.40E-07	1.15E-06
3022	ENSMUSG00000025351	Cd63	-0.86	3.45E-07	1.17E-06
3023	ENSMUSG00000079562	Maea	0.81	3.46E-07	1.17E-06
3024	ENSMUSG00000001755	Coasy	0.78	3.46E-07	1.17E-06
3025	ENSMUSG00000071072	Ptges3	0.86	3.48E-07	1.18E-06
3026	ENSMUSG00000037300	Ttc13	-0.93	3.49E-07	1.18E-06
3027	ENSMUSG00000072640	Lyrm9	1.12	3.50E-07	1.18E-06
3028	ENSMUSG00000039262	Prrc2b	-1.04	3.52E-07	1.19E-06
3029	ENSMUSG00000029047	Pex10	1.02	3.53E-07	1.19E-06
3030	ENSMUSG00000037685	Atp8a1	1.11	3.57E-07	1.20E-06
3031	ENSMUSG00000029136	Rbks	1.17	3.57E-07	1.21E-06
3032	ENSMUSG00000053929	Cyhr1	0.80	3.62E-07	1.22E-06
3033	ENSMUSG00000045665	Mfsd5	-0.87	3.62E-07	1.22E-06
3034	ENSMUSG00000097125	Gm26885	1.42	3.65E-07	1.23E-06
3035	ENSMUSG00000024480	Ap3s1	0.78	3.66E-07	1.23E-06
3036	ENSMUSG00000034684	Sema3f	-1.55	3.68E-07	1.24E-06
3037	ENSMUSG00000056718	Gm13199	1.07	3.69E-07	1.24E-06
3038	ENSMUSG00000022295	Atp6v1c1	0.81	3.70E-07	1.25E-06
3039	ENSMUSG00000027499	Pkia	-1.38	3.72E-07	1.25E-06
3040	ENSMUSG00000019659	Ccdc12	1.02	3.72E-07	1.25E-06
3041	ENSMUSG00000034807	Colgalt1	-0.91	3.74E-07	1.26E-06
3042	ENSMUSG00000005621	Zfp592	-1.07	3.77E-07	1.27E-06
3043	ENSMUSG00000059173	Pde1a	-1.05	3.77E-07	1.27E-06
3044	ENSMUSG00000015461	Atf6b	-1.01	3.77E-07	1.27E-06
3045	ENSMUSG00000020697	Lig3	-1.27	3.80E-07	1.28E-06
3046	ENSMUSG00000003154	Foxj2	-1.06	3.81E-07	1.28E-06
3047	ENSMUSG00000026197	Zfand2b	0.92	3.81E-07	1.28E-06

3048	ENSMUSG00000026116	Tmem131	-0.95	3.83E-07	1.29E-06
3049	ENSMUSG00000025241	Fyco1	-0.94	3.84E-07	1.29E-06
3050	ENSMUSG00000041126	H2afv	0.85	3.86E-07	1.29E-06
3051	ENSMUSG00000026946	Nmi	-1.33	3.86E-07	1.29E-06
3052	ENSMUSG00000003123	Lipe	-0.90	3.86E-07	1.29E-06
3053	ENSMUSG00000022150	Dab2	-1.35	3.89E-07	1.30E-06
3054	ENSMUSG00000060012	Kif13b	-1.44	3.93E-07	1.32E-06
3055	ENSMUSG00000038080	Kdm1b	-1.08	3.96E-07	1.33E-06
3056	ENSMUSG00000044378	Slc15a5	1.51	4.03E-07	1.35E-06
3057	ENSMUSG00000032238	Rora	-0.97	4.04E-07	1.35E-06
3058	ENSMUSG00000040613	Apobec1	-1.40	4.07E-07	1.36E-06
3059	ENSMUSG00000030788	Rnf141	-1.03	4.10E-07	1.37E-06
3060	ENSMUSG00000110156	Gm45248	1.41	4.14E-07	1.38E-06
3061	ENSMUSG00000040026	Saa3	-1.42	4.14E-07	1.38E-06
3062	ENSMUSG00000022791	Tnk2	-1.07	4.15E-07	1.39E-06
3063	ENSMUSG00000041341	Atg2b	-1.00	4.18E-07	1.40E-06
3064	ENSMUSG00000031545	Gpat4	0.89	4.19E-07	1.40E-06
3065	ENSMUSG00000029650	Slc46a3	-0.94	4.20E-07	1.40E-06
3066	ENSMUSG00000020877	Scrn2	1.10	4.20E-07	1.40E-06
3067	ENSMUSG00000029119	Man2b2	-1.12	4.22E-07	1.41E-06
3068	ENSMUSG00000005370	Msh6	-1.51	4.22E-07	1.41E-06
3069	ENSMUSG00000029752	Asns	-0.82	4.27E-07	1.42E-06
3070	ENSMUSG00000001751	Naglu	-0.91	4.32E-07	1.44E-06
3071	ENSMUSG00000038975	Rabggtb	1.27	4.35E-07	1.45E-06
3072	ENSMUSG00000022098	Bmp1	-1.39	4.35E-07	1.45E-06
3073	ENSMUSG00000037275	Gemin5	-1.24	4.38E-07	1.46E-06
3074	ENSMUSG00000059796	Eif4a1	0.84	4.41E-07	1.47E-06
3075	ENSMUSG00000030409	Dmpk	-1.01	4.42E-07	1.47E-06
3076	ENSMUSG00000039477	Tnrc18	-1.19	4.42E-07	1.47E-06
3077	ENSMUSG00000037824	Tspan14	-0.85	4.42E-07	1.47E-06
3078	ENSMUSG00000066637	Ttc32	1.13	4.46E-07	1.48E-06
3079	ENSMUSG00000031812	Map1lc3b	0.80	4.46E-07	1.48E-06
3080	ENSMUSG00000006169	Clint1	-0.88	4.47E-07	1.48E-06
3081	ENSMUSG00000028013	Ppa2	0.81	4.48E-07	1.49E-06
3082	ENSMUSG00000051177	Plcb1	-1.26	4.50E-07	1.49E-06
3083	ENSMUSG00000034575	Papd7	-1.34	4.50E-07	1.49E-06
3084	ENSMUSG00000040236	Trappc5	0.95	4.50E-07	1.49E-06
3085	ENSMUSG00000024524	Gnal	-1.09	4.52E-07	1.50E-06
3086	ENSMUSG00000031299	Pdha1	0.89	4.54E-07	1.50E-06
3087	ENSMUSG00000079659	Tmem243	0.84	4.56E-07	1.51E-06
3088	ENSMUSG00000038429	Usp5	-0.87	4.56E-07	1.51E-06
3089	ENSMUSG00000060032	H2afj	0.95	4.58E-07	1.52E-06
3090	ENSMUSG00000060147	Serpinb6a	-1.12	4.59E-07	1.52E-06
3091	ENSMUSG00000039680	Mrps6	0.82	4.60E-07	1.52E-06
3092	ENSMUSG00000027222	Pex16	0.78	4.61E-07	1.53E-06
3093	ENSMUSG00000023156	Rpp14	0.82	4.63E-07	1.53E-06
3094	ENSMUSG00000040268	Plekha1	-0.98	4.63E-07	1.53E-06
3095	ENSMUSG00000040410	Fbxl4	-0.86	4.63E-07	1.53E-06
3096	ENSMUSG00000024074	Crim1	-1.25	4.65E-07	1.53E-06
3097	ENSMUSG00000031841	Cdh13	-1.13	4.66E-07	1.54E-06

3098	ENSMUSG00000040242	Fgfr1op2	0.78	4.69E-07	1.55E-06
3099	ENSMUSG00000030172	Erc1	-1.11	4.70E-07	1.55E-06
3100	ENSMUSG00000018599	Mief2	0.91	4.70E-07	1.55E-06
3101	ENSMUSG00000052776	Oas1a	-1.33	4.71E-07	1.55E-06
3102	ENSMUSG00000031303	Map3k15	0.86	4.71E-07	1.55E-06
3103	ENSMUSG00000059518	Znhit1	0.90	4.74E-07	1.56E-06
3104	ENSMUSG00000026504	Sdccag8	-1.28	4.76E-07	1.57E-06
3105	ENSMUSG00000046275	Tusc5	-0.98	4.78E-07	1.57E-06
3106	ENSMUSG00000039813	Tbc1d2	-1.23	4.78E-07	1.57E-06
3107	ENSMUSG00000089979	Gm15954	0.83	4.81E-07	1.58E-06
3108	ENSMUSG00000035770	Dync1li2	-0.84	4.81E-07	1.58E-06
3109	ENSMUSG00000026309	Ilkap	0.90	4.81E-07	1.58E-06
3110	ENSMUSG00000028949	Smarcd3	-0.96	4.86E-07	1.60E-06
3111	ENSMUSG00000020114	Cand1	-0.91	4.86E-07	1.60E-06
3112	ENSMUSG00000028234	Rps20	1.05	4.89E-07	1.61E-06
3113	ENSMUSG00000048218	Amigo2	-1.42	4.90E-07	1.61E-06
3114	ENSMUSG00000026839	Upp2	1.28	4.90E-07	1.61E-06
3115	ENSMUSG00000019302	Atp6v0a1	-0.87	4.92E-07	1.62E-06
3116	ENSMUSG00000031373	Car5b	-1.32	4.93E-07	1.62E-06
3117	ENSMUSG00000046532	Ar	-1.28	4.93E-07	1.62E-06
3118	ENSMUSG00000045503	Sys1	0.80	4.94E-07	1.62E-06
3119	ENSMUSG00000024085	Man2a1	-1.20	4.97E-07	1.63E-06
3120	ENSMUSG00000022041	Chrna2	1.36	5.02E-07	1.65E-06
3121	ENSMUSG00000026977	March7	-1.04	5.09E-07	1.67E-06
3122	ENSMUSG00000021796	Bmpr1a	-1.08	5.12E-07	1.68E-06
3123	ENSMUSG00000024896	Minpp1	-0.91	5.12E-07	1.68E-06
3124	ENSMUSG00000003813	Rad23a	0.76	5.17E-07	1.69E-06
3125	ENSMUSG00000072704	Smim10l1	0.78	5.21E-07	1.71E-06
3126	ENSMUSG00000035215	Lsm7	1.19	5.25E-07	1.72E-06
3127	ENSMUSG00000004771	Rab11a	0.81	5.29E-07	1.73E-06
3128	ENSMUSG00000033577	Myo6	-1.19	5.30E-07	1.73E-06
3129	ENSMUSG00000068250	Amn1	1.06	5.31E-07	1.74E-06
3130	ENSMUSG00000112880	AC127337.2	1.50	5.32E-07	1.74E-06
3131	ENSMUSG00000019647	Sema6a	-1.26	5.34E-07	1.74E-06
3132	ENSMUSG00000026104	Stat1	-1.06	5.38E-07	1.76E-06
3133	ENSMUSG00000054556	Gm4876	-1.35	5.40E-07	1.76E-06
3134	ENSMUSG00000071573	Rnls	1.20	5.40E-07	1.76E-06
3135	ENSMUSG00000039536	Stau1	-0.90	5.44E-07	1.77E-06
3136	ENSMUSG00000023828	Slc22a3	-0.92	5.45E-07	1.78E-06
3137	ENSMUSG00000109357	Gm45236	0.81	5.45E-07	1.78E-06
3138	ENSMUSG00000034845	Plvap	-1.53	5.51E-07	1.80E-06
3139	ENSMUSG00000038970	Lmtk2	-1.06	5.51E-07	1.80E-06
3140	ENSMUSG00000097828	6430562O15Rik	0.94	5.52E-07	1.80E-06
3141	ENSMUSG00000026958	Dpp7	0.91	5.52E-07	1.80E-06
3142	ENSMUSG00000026728	Vim	-0.78	5.55E-07	1.81E-06
3143	ENSMUSG00000024924	Vldlr	0.78	5.60E-07	1.82E-06
3144	ENSMUSG00000050335	Lgals3	-1.06	5.60E-07	1.82E-06
3145	ENSMUSG00000068245	Phf11d	-1.35	5.61E-07	1.82E-06
3146	ENSMUSG00000039159	Ube2h	-0.93	5.63E-07	1.83E-06
3147	ENSMUSG00000061286	Exosc5	0.82	5.63E-07	1.83E-06

3148	ENSMUSG00000032253	Phip	-1.17	5.73E-07	1.86E-06
3149	ENSMUSG00000058301	Upf1	-0.87	5.74E-07	1.86E-06
3150	ENSMUSG00000039483	Asb6	-1.09	5.74E-07	1.86E-06
3151	ENSMUSG00000027942	4933434E20Rik	0.84	5.74E-07	1.86E-06
3152	ENSMUSG00000031024	St5	-1.20	5.76E-07	1.87E-06
3153	ENSMUSG00000025764	Jade1	-1.01	5.78E-07	1.87E-06
3154	ENSMUSG00000040940	Arhgef1	-1.01	5.78E-07	1.88E-06
3155	ENSMUSG00000032309	Fbxo22	-0.82	5.82E-07	1.89E-06
3156	ENSMUSG00000031570	Plpp5	-1.11	5.85E-07	1.89E-06
3157	ENSMUSG00000052293	Taf9	0.92	5.85E-07	1.89E-06
3158	ENSMUSG00000033124	Atg9a	-0.85	5.86E-07	1.90E-06
3159	ENSMUSG00000027519	Rab22a	0.78	5.88E-07	1.90E-06
3160	ENSMUSG00000028609	Magoh	1.20	5.89E-07	1.91E-06
3161	ENSMUSG00000036620	Mgat4b	-0.77	5.94E-07	1.92E-06
3162	ENSMUSG00000094439	Gm21969	-0.84	5.97E-07	1.93E-06
3163	ENSMUSG00000030613	Ccdc90b	1.01	6.02E-07	1.95E-06
3164	ENSMUSG00000024538	Ppic	-0.98	6.08E-07	1.96E-06
3165	ENSMUSG00000039782	Cpeb2	-1.12	6.08E-07	1.97E-06
3166	ENSMUSG00000025979	Mob4	0.80	6.11E-07	1.97E-06
3167	ENSMUSG00000045594	Glb1	-0.99	6.13E-07	1.98E-06
3168	ENSMUSG00000087658	Hotairm1	1.31	6.14E-07	1.98E-06
3169	ENSMUSG00000021357	Exoc2	-1.23	6.17E-07	1.99E-06
3170	ENSMUSG00000101628	Gm28177	-1.05	6.18E-07	2.00E-06
3171	ENSMUSG00000018931	Natd1	-0.93	6.19E-07	2.00E-06
3172	ENSMUSG00000041263	Rusc1	1.16	6.20E-07	2.00E-06
3173	ENSMUSG00000022792	Yars2	1.02	6.21E-07	2.00E-06
3174	ENSMUSG00000039887	Alg14	0.92	6.27E-07	2.02E-06
3175	ENSMUSG00000031217	Efnb1	-1.10	6.28E-07	2.02E-06
3176	ENSMUSG00000090935	Synj2bp	0.81	6.28E-07	2.02E-06
3177	ENSMUSG00000030315	Vgll4	0.82	6.32E-07	2.04E-06
3178	ENSMUSG00000061540	Orm2	0.82	6.41E-07	2.06E-06
3179	ENSMUSG00000036270	Edc4	-0.99	6.42E-07	2.07E-06
3180	ENSMUSG00000021339	Mrs2	0.86	6.44E-07	2.07E-06
3181	ENSMUSG00000037348	Paqr7	-1.22	6.48E-07	2.08E-06
3182	ENSMUSG00000052926	Rnaseh2a	0.88	6.49E-07	2.09E-06
3183	ENSMUSG00000073437	D330041H03Rik	-1.16	6.53E-07	2.10E-06
3184	ENSMUSG00000090996	Gm20458	0.79	6.56E-07	2.11E-06
3185	ENSMUSG00000054021	Sirt5	1.02	6.57E-07	2.11E-06
3186	ENSMUSG00000003032	Klf4	-1.31	6.59E-07	2.12E-06
3187	ENSMUSG00000032507	Fbxl2	1.36	6.68E-07	2.14E-06
3188	ENSMUSG00000028633	Ctps	-1.30	6.69E-07	2.15E-06
3189	ENSMUSG00000049287	Iba57	0.92	6.72E-07	2.16E-06
3190	ENSMUSG00000042082	Arsb	-0.92	6.73E-07	2.16E-06
3191	ENSMUSG00000033684	Qsox1	-1.09	6.77E-07	2.17E-06
3192	ENSMUSG00000003662	Ciao1	0.79	6.78E-07	2.17E-06
3193	ENSMUSG00000031010	Usp9x	-1.15	6.84E-07	2.19E-06
3194	ENSMUSG00000019929	Dcn	-1.30	6.85E-07	2.19E-06
3195	ENSMUSG00000032803	Cdv3	0.86	6.86E-07	2.20E-06
3196	ENSMUSG00000092345	Gm20503	0.95	6.89E-07	2.21E-06
3197	ENSMUSG00000028741	Mrto4	1.12	6.89E-07	2.21E-06

3198	ENSMUSG00000006418	Rnf114	-0.89	7.00E-07	2.24E-06
3199	ENSMUSG00000039458	Mtmr12	-1.23	7.04E-07	2.25E-06
3200	ENSMUSG00000032714	Syde1	-1.32	7.06E-07	2.26E-06
3201	ENSMUSG00000022394	L3mbtl2	-0.95	7.15E-07	2.28E-06
3202	ENSMUSG00000040479	Dgkz	-1.11	7.19E-07	2.30E-06
3203	ENSMUSG00000020142	Slc1a4	-1.33	7.20E-07	2.30E-06
3204	ENSMUSG00000022636	Alcam	-1.79	7.21E-07	2.30E-06
3205	ENSMUSG00000031388	Naa10	0.84	7.25E-07	2.31E-06
3206	ENSMUSG00000022946	Dopey2	-0.98	7.30E-07	2.33E-06
3207	ENSMUSG00000027247	Arhgap1	-0.89	7.31E-07	2.33E-06
3208	ENSMUSG00000025986	Slc39a10	-1.50	7.32E-07	2.33E-06
3209	ENSMUSG00000026834	Acvr1c	-0.98	7.32E-07	2.33E-06
3210	ENSMUSG00000028556	Dock7	-1.36	7.35E-07	2.34E-06
3211	ENSMUSG00000002102	Psmc3	0.75	7.35E-07	2.34E-06
3212	ENSMUSG00000020300	Cpeb4	-1.30	7.36E-07	2.34E-06
3213	ENSMUSG00000003166	Dgcr2	-0.99	7.38E-07	2.35E-06
3214	ENSMUSG00000025373	Rnf41	-1.00	7.39E-07	2.35E-06
3215	ENSMUSG00000029649	Pomp	0.87	7.40E-07	2.35E-06
3216	ENSMUSG00000042364	Snx18	-1.07	7.41E-07	2.36E-06
3217	ENSMUSG00000024927	Rela	-0.79	7.44E-07	2.37E-06
3218	ENSMUSG00000113816	CT030159.3	0.87	7.44E-07	2.37E-06
3219	ENSMUSG00000106643	Gm43422	-1.31	7.44E-07	2.37E-06
3220	ENSMUSG00000021018	Polr2h	1.10	7.50E-07	2.38E-06
3221	ENSMUSG00000036636	Clcn7	-0.89	7.51E-07	2.38E-06
3222	ENSMUSG00000045667	Smtnl2	-1.08	7.51E-07	2.38E-06
3223	ENSMUSG00000051329	Nup160	-1.29	7.51E-07	2.38E-06
3224	ENSMUSG00000035413	Tmem98	-1.17	7.53E-07	2.39E-06
3225	ENSMUSG00000021109	Hif1a	-0.82	7.56E-07	2.40E-06
3226	ENSMUSG00000022884	Eif4a2	0.98	7.56E-07	2.40E-06
3227	ENSMUSG00000017428	Psmd11	0.76	7.57E-07	2.40E-06
3228	ENSMUSG00000022270	Fam134b	1.18	7.59E-07	2.40E-06
3229	ENSMUSG00000034271	Jdp2	-1.05	7.62E-07	2.41E-06
3230	ENSMUSG00000069662	Marcks	-1.09	7.62E-07	2.41E-06
3231	ENSMUSG00000047963	Stbd1	1.22	7.70E-07	2.44E-06
3232	ENSMUSG00000010057	Nprl2	0.90	7.78E-07	2.46E-06
3233	ENSMUSG00000036461	Elf1	-1.05	7.78E-07	2.46E-06
3234	ENSMUSG00000000001	Gnai3	-0.95	7.81E-07	2.47E-06
3235	ENSMUSG00000021306	Gpr137b	-1.01	7.84E-07	2.48E-06
3236	ENSMUSG00000037652	Phc3	-1.26	7.85E-07	2.48E-06
3237	ENSMUSG00000020832	Eral1	0.80	7.87E-07	2.49E-06
3238	ENSMUSG00000037071	Scd1	-1.45	7.88E-07	2.49E-06
3239	ENSMUSG00000031871	Cdh5	-1.04	7.89E-07	2.49E-06
3240	ENSMUSG00000022421	Nptxr	-1.40	7.98E-07	2.52E-06
3241	ENSMUSG00000040463	Mybbp1a	-0.85	8.01E-07	2.53E-06
3242	ENSMUSG00000024217	Snrpc	1.02	8.04E-07	2.54E-06
3243	ENSMUSG00000085695	Gm15243	-1.21	8.10E-07	2.55E-06
3244	ENSMUSG00000098889	Gm27206	-1.31	8.18E-07	2.58E-06
3245	ENSMUSG00000029482	Aacs	-1.01	8.18E-07	2.58E-06
3246	ENSMUSG00000063200	Nol7	0.94	8.22E-07	2.59E-06
3247	ENSMUSG00000022469	Rapgef3	-1.38	8.22E-07	2.59E-06

3248	ENSMUSG00000040997	Abhd4	0.77	8.38E-07	2.64E-06
3249	ENSMUSG00000000823	Zfp512b	-1.26	8.40E-07	2.64E-06
3250	ENSMUSG00000025369	Smarcc2	-1.06	8.40E-07	2.64E-06
3251	ENSMUSG00000024423	Impact	-1.12	8.45E-07	2.66E-06
3252	ENSMUSG00000002635	Pdcd2l	1.02	8.47E-07	2.66E-06
3253	ENSMUSG00000027937	Jtb	0.82	8.47E-07	2.66E-06
3254	ENSMUSG00000004568	Arhgef18	-1.01	8.50E-07	2.67E-06
3255	ENSMUSG00000047777	Phf13	-1.31	8.54E-07	2.68E-06
3256	ENSMUSG00000024566	Atp9b	-0.87	8.59E-07	2.70E-06
3257	ENSMUSG00000046573	Lyrm4	1.25	8.60E-07	2.70E-06
3258	ENSMUSG00000033585	Ndn	0.93	8.60E-07	2.70E-06
3259	ENSMUSG00000002345	Borcs8	0.81	8.66E-07	2.72E-06
3260	ENSMUSG00000084780	Gm15350	-1.37	8.68E-07	2.72E-06
3261	ENSMUSG00000022591	Gm9747	-1.03	8.74E-07	2.74E-06
3262	ENSMUSG00000006127	Inpp5k	-0.83	8.74E-07	2.74E-06
3263	ENSMUSG00000052488	Cherp	-0.97	8.99E-07	2.82E-06
3264	ENSMUSG00000032340	Neo1	-1.27	9.00E-07	2.82E-06
3265	ENSMUSG00000113647	AC131739.1	-1.25	9.00E-07	2.82E-06
3266	ENSMUSG00000060268	Gm1661	1.13	9.14E-07	2.86E-06
3267	ENSMUSG00000000740	Rpl13	0.83	9.17E-07	2.87E-06
3268	ENSMUSG00000022443	Myh9	-0.96	9.20E-07	2.88E-06
3269	ENSMUSG00000003546	Klc4	0.88	9.20E-07	2.88E-06
3270	ENSMUSG00000026576	Atp1b1	1.62	9.21E-07	2.88E-06
3271	ENSMUSG00000032560	Dnajc13	-1.06	9.27E-07	2.90E-06
3272	ENSMUSG00000027765	P2ry1	1.45	9.27E-07	2.90E-06
3273	ENSMUSG00000031360	Ctps2	-0.97	9.29E-07	2.90E-06
3274	ENSMUSG00000022721	Trmt2a	-1.08	9.29E-07	2.90E-06
3275	ENSMUSG00000048495	Tyw5	0.94	9.33E-07	2.91E-06
3276	ENSMUSG00000038457	Tmem255b	-1.25	9.36E-07	2.92E-06
3277	ENSMUSG00000033653	Vps8	-1.15	9.37E-07	2.93E-06
3278	ENSMUSG00000025793	Hgs	-0.79	9.46E-07	2.95E-06
3279	ENSMUSG00000033916	Chmp2a	0.88	9.51E-07	2.97E-06
3280	ENSMUSG00000032194	Kank2	-1.03	9.54E-07	2.98E-06
3281	ENSMUSG00000037949	Ano10	-0.77	9.55E-07	2.98E-06
3282	ENSMUSG00000022614	Lmf2	-0.85	9.56E-07	2.98E-06
3283	ENSMUSG00000037720	Tmem33	0.76	9.67E-07	3.01E-06
3284	ENSMUSG00000028649	Macf1	1.11	9.74E-07	3.03E-06
3285	ENSMUSG00000007721	Ccdc124	0.88	9.77E-07	3.04E-06
3286	ENSMUSG00000057103	Nat8f1	1.11	9.86E-07	3.07E-06
3287	ENSMUSG00000040550	Otud6b	0.76	9.87E-07	3.07E-06
3288	ENSMUSG00000027397	Slc20a1	-1.05	9.87E-07	3.07E-06
3289	ENSMUSG00000028467	Gba2	-1.17	9.87E-07	3.07E-06
3290	ENSMUSG00000050379	Sep-06	-1.35	9.90E-07	3.08E-06
3291	ENSMUSG00000022337	Emc2	0.76	1.01E-06	3.12E-06
3292	ENSMUSG00000031399	Fam3a	-1.01	1.01E-06	3.13E-06
3293	ENSMUSG00000062397	Zfp706	0.72	1.01E-06	3.14E-06
3294	ENSMUSG00000027429	Sec23b	-0.88	1.02E-06	3.16E-06
3295	ENSMUSG00000010307	Tmem86a	-1.45	1.02E-06	3.16E-06
3296	ENSMUSG00000062373	Tmem65	0.78	1.03E-06	3.21E-06
3297	ENSMUSG00000029581	Fscn1	-1.44	1.04E-06	3.21E-06

3298	ENSMUSG00000010358	Ifi35	-1.00	1.04E-06	3.22E-06
3299	ENSMUSG00000032508	Myd88	-0.88	1.04E-06	3.23E-06
3300	ENSMUSG00000021608	Lpcat1	-1.21	1.05E-06	3.24E-06
3301	ENSMUSG00000074466	Gm15417	0.88	1.05E-06	3.25E-06
3302	ENSMUSG00000024026	Glo1	0.75	1.06E-06	3.28E-06
3303	ENSMUSG00000090122	Kcne1l	-1.35	1.06E-06	3.28E-06
3304	ENSMUSG00000022972	1110004E09Rik	1.02	1.07E-06	3.30E-06
3305	ENSMUSG00000109807	Gm45244	0.80	1.07E-06	3.30E-06
3306	ENSMUSG00000040374	Pex2	0.77	1.07E-06	3.31E-06
3307	ENSMUSG00000009995	Taz	0.83	1.08E-06	3.33E-06
3308	ENSMUSG00000067274	Rplp0	0.74	1.08E-06	3.34E-06
3309	ENSMUSG00000021550	2210016F16Rik	0.79	1.08E-06	3.34E-06
3310	ENSMUSG00000028032	Papss1	-1.01	1.08E-06	3.35E-06
3311	ENSMUSG00000078440	Dohh	0.76	1.09E-06	3.37E-06
3312	ENSMUSG00000020801	Med31	1.20	1.10E-06	3.38E-06
3313	ENSMUSG00000033902	Mapkbp1	-1.30	1.10E-06	3.38E-06
3314	ENSMUSG00000057329	Bcl2	-1.32	1.11E-06	3.42E-06
3315	ENSMUSG00000045519	Zfp560	1.10	1.12E-06	3.46E-06
3316	ENSMUSG00000010048	Ifrd2	0.90	1.12E-06	3.46E-06
3317	ENSMUSG00000027951	Adar	-1.24	1.13E-06	3.48E-06
3318	ENSMUSG00000028399	Ptprd	-0.85	1.13E-06	3.49E-06
3319	ENSMUSG00000024740	Ddb1	-0.89	1.13E-06	3.49E-06
3320	ENSMUSG00000055652	Klhl25	-1.03	1.13E-06	3.49E-06
3321	ENSMUSG00000021012	Zc3h14	0.78	1.14E-06	3.51E-06
3322	ENSMUSG00000021374	Nup153	-1.09	1.15E-06	3.52E-06
3323	ENSMUSG00000007613	Tgfbr1	-1.08	1.16E-06	3.56E-06
3324	ENSMUSG00000098234	Snhg6	1.22	1.16E-06	3.56E-06
3325	ENSMUSG00000021745	Ptprg	-0.96	1.16E-06	3.57E-06
3326	ENSMUSG00000026784	Pdss1	1.06	1.16E-06	3.57E-06
3327	ENSMUSG00000031683	Lsm6	0.91	1.16E-06	3.57E-06
3328	ENSMUSG00000029145	Eif2b4	0.76	1.16E-06	3.57E-06
3329	ENSMUSG00000040297	Suco	-1.16	1.16E-06	3.57E-06
3330	ENSMUSG00000047098	Rnf31	-1.11	1.17E-06	3.60E-06
3331	ENSMUSG00000023707	Ogfod2	0.78	1.18E-06	3.62E-06
3332	ENSMUSG00000029370	Rassf6	1.17	1.18E-06	3.63E-06
3333	ENSMUSG00000025794	Rpl14	0.90	1.20E-06	3.67E-06
3334	ENSMUSG00000074829	2010315B03Rik	0.98	1.20E-06	3.68E-06
3335	ENSMUSG00000002550	Uck1	0.86	1.20E-06	3.68E-06
3336	ENSMUSG00000062031	Pgghg	-0.92	1.20E-06	3.69E-06
3337	ENSMUSG00000035745	Grin3b	1.40	1.20E-06	3.69E-06
3338	ENSMUSG00000042178	Armc5	-0.97	1.21E-06	3.70E-06
3339	ENSMUSG00000029776	Hibadh	0.74	1.23E-06	3.75E-06
3340	ENSMUSG00000025139	Tollip	-0.80	1.23E-06	3.75E-06
3341	ENSMUSG00000030536	Iqgap1	-0.81	1.23E-06	3.77E-06
3342	ENSMUSG00000085438	1700020I14Rik	0.75	1.23E-06	3.77E-06
3343	ENSMUSG00000036144	Meox2	-1.21	1.24E-06	3.78E-06
3344	ENSMUSG00000097407	4933408J17Rik	1.15	1.25E-06	3.83E-06
3345	ENSMUSG00000048807	Slc35e4	-1.38	1.25E-06	3.83E-06
3346	ENSMUSG00000034543	Morc2a	-1.07	1.27E-06	3.87E-06
3347	ENSMUSG00000058587	Tmod3	-0.84	1.27E-06	3.88E-06

3348	ENSMUSG00000033813	Tcea1	0.76	1.28E-06	3.90E-06
3349	ENSMUSG00000081534	Slc48a1	-0.83	1.28E-06	3.92E-06
3350	ENSMUSG00000078350	Smim1	-1.05	1.28E-06	3.92E-06
3351	ENSMUSG00000008855	Hdac5	-0.76	1.30E-06	3.96E-06
3352	ENSMUSG00000055491	Pprc1	-1.02	1.30E-06	3.97E-06
3353	ENSMUSG00000070034	Sp110	-1.29	1.31E-06	3.98E-06
3354	ENSMUSG00000010936	Vac14	-0.97	1.31E-06	4.00E-06
3355	ENSMUSG00000053907	Mat2a	-0.77	1.31E-06	4.01E-06
3356	ENSMUSG00000028967	Errfi1	-1.15	1.32E-06	4.01E-06
3357	ENSMUSG00000010051	Hyal1	0.75	1.32E-06	4.02E-06
3358	ENSMUSG00000032215	Rsl24d1	0.92	1.32E-06	4.03E-06
3359	ENSMUSG00000004880	Lbr	-1.25	1.32E-06	4.03E-06
3360	ENSMUSG00000054364	Rhob	-0.88	1.33E-06	4.03E-06
3361	ENSMUSG00000062867	Impdh2	-1.06	1.33E-06	4.04E-06
3362	ENSMUSG00000027163	Commd9	0.91	1.34E-06	4.06E-06
3363	ENSMUSG00000020089	Ppa1	0.74	1.35E-06	4.10E-06
3364	ENSMUSG00000030342	Cd9	-1.35	1.35E-06	4.11E-06
3365	ENSMUSG00000060703	Cd302	-0.79	1.36E-06	4.12E-06
3366	ENSMUSG00000113262	Dohh	0.77	1.36E-06	4.13E-06
3367	ENSMUSG00000026239	Pde6d	1.14	1.36E-06	4.14E-06
3368	ENSMUSG00000056050	Mia3	-0.92	1.38E-06	4.20E-06
3369	ENSMUSG00000019432	Ddx39b	-0.77	1.39E-06	4.22E-06
3370	ENSMUSG00000041736	Tspo	0.83	1.40E-06	4.23E-06
3371	ENSMUSG00000009575	Cbx5	-0.90	1.40E-06	4.24E-06
3372	ENSMUSG00000028847	Trappc3	0.78	1.40E-06	4.24E-06
3373	ENSMUSG00000059149	Mfsd4a	-1.07	1.40E-06	4.25E-06
3374	ENSMUSG00000027955	Fam198b	-1.27	1.41E-06	4.28E-06
3375	ENSMUSG00000037606	Osbpl5	-1.06	1.41E-06	4.28E-06
3376	ENSMUSG00000039087	Rreb1	-0.91	1.42E-06	4.29E-06
3377	ENSMUSG00000028412	Slc44a1	-0.79	1.42E-06	4.29E-06
3378	ENSMUSG00000017264	Exosc10	-0.94	1.42E-06	4.29E-06
3379	ENSMUSG00000034021	Pds5b	-1.16	1.42E-06	4.29E-06
3380	ENSMUSG00000024856	Cdk2ap2	0.73	1.43E-06	4.32E-06
3381	ENSMUSG00000039004	Bmp6	-1.24	1.43E-06	4.33E-06
3382	ENSMUSG00000026254	Eif4e2	0.79	1.44E-06	4.35E-06
3383	ENSMUSG00000018999	Slc35b4	-1.16	1.44E-06	4.36E-06
3384	ENSMUSG00000031758	Cdyl2	-1.28	1.45E-06	4.38E-06
3385	ENSMUSG00000019969	Psen1	-0.94	1.45E-06	4.39E-06
3386	ENSMUSG00000030972	Acsm5	-1.13	1.46E-06	4.40E-06
3387	ENSMUSG00000034620	Tmem5	0.75	1.46E-06	4.42E-06
3388	ENSMUSG00000026491	Ahctf1	-1.13	1.47E-06	4.42E-06
3389	ENSMUSG00000027868	Tbx15	-1.26	1.48E-06	4.46E-06
3390	ENSMUSG00000032754	Slc8b1	-0.98	1.48E-06	4.47E-06
3391	ENSMUSG00000028914	Casp9	-0.96	1.49E-06	4.49E-06
3392	ENSMUSG00000022546	Gpt	-0.83	1.49E-06	4.49E-06
3393	ENSMUSG00000039414	Heatr5b	-0.94	1.49E-06	4.49E-06
3394	ENSMUSG00000100838	Gm29094	1.09	1.50E-06	4.51E-06
3395	ENSMUSG00000024767	Otub1	0.84	1.50E-06	4.52E-06
3396	ENSMUSG00000016831	Tox4	-0.87	1.51E-06	4.54E-06
3397	ENSMUSG00000006021	Kptn	0.82	1.51E-06	4.54E-06

3398	ENSMUSG00000033697	Arhgap39	-1.29	1.51E-06	4.54E-06
3399	ENSMUSG00000085208	Brip1os	-1.01	1.51E-06	4.54E-06
3400	ENSMUSG00000029723	Tsc22d4	-0.91	1.51E-06	4.55E-06
3401	ENSMUSG00000030513	Pcsk6	-0.76	1.52E-06	4.56E-06
3402	ENSMUSG00000033918	Parl	0.79	1.52E-06	4.56E-06
3403	ENSMUSG00000048440	Cyp4f16	-1.02	1.53E-06	4.58E-06
3404	ENSMUSG00000039197	Adk	0.74	1.53E-06	4.60E-06
3405	ENSMUSG00000020803	Txndc17	0.81	1.55E-06	4.64E-06
3406	ENSMUSG00000028433	Ubap2	-0.95	1.55E-06	4.66E-06
3407	ENSMUSG00000008540	Mgst1	0.79	1.57E-06	4.70E-06
3408	ENSMUSG00000014503	Pkd2l2	1.07	1.57E-06	4.70E-06
3409	ENSMUSG00000078853	Igtp	-1.34	1.58E-06	4.73E-06
3410	ENSMUSG00000007029	Vars	-0.78	1.58E-06	4.74E-06
3411	ENSMUSG00000078578	Ube2d3	0.74	1.58E-06	4.74E-06
3412	ENSMUSG00000020900	Myh10	-1.20	1.58E-06	4.74E-06
3413	ENSMUSG00000022878	Adipoq	-0.86	1.59E-06	4.77E-06
3414	ENSMUSG00000021518	Ptdss1	-0.78	1.61E-06	4.82E-06
3415	ENSMUSG00000029360	Gm9754	-0.87	1.61E-06	4.82E-06
3416	ENSMUSG00000034981	Parm1	1.02	1.61E-06	4.82E-06
3417	ENSMUSG00000026922	Agpat2	0.87	1.62E-06	4.84E-06
3418	ENSMUSG00000112129	Pbld1	1.32	1.62E-06	4.86E-06
3419	ENSMUSG00000003435	Supt5	-0.76	1.62E-06	4.86E-06
3420	ENSMUSG00000089945	Pakap	-0.82	1.63E-06	4.87E-06
3421	ENSMUSG00000038685	Rtel1	-1.06	1.65E-06	4.92E-06
3422	ENSMUSG00000034401	Spata6	-1.07	1.66E-06	4.95E-06
3423	ENSMUSG00000091337	Eid1	-0.97	1.66E-06	4.97E-06
3424	ENSMUSG00000039347	Atp6v0e2	-1.45	1.67E-06	4.99E-06
3425	ENSMUSG00000022962	Gart	-0.78	1.68E-06	5.00E-06
3426	ENSMUSG00000005899	Smpd4	-0.97	1.68E-06	5.00E-06
3427	ENSMUSG00000086407	Gm14123	0.95	1.68E-06	5.02E-06
3428	ENSMUSG00000026621	Marc1	0.88	1.68E-06	5.02E-06
3429	ENSMUSG00000063511	Snrnp70	-0.97	1.69E-06	5.03E-06
3430	ENSMUSG00000027365	Trpm7	-0.88	1.69E-06	5.04E-06
3431	ENSMUSG00000025858	Get4	0.80	1.70E-06	5.06E-06
3432	ENSMUSG00000021215	Net1	-1.60	1.70E-06	5.06E-06
3433	ENSMUSG00000074030	Exoc8	-0.92	1.71E-06	5.08E-06
3434	ENSMUSG00000021466	Ptch1	-1.23	1.72E-06	5.11E-06
3435	ENSMUSG00000029176	Anapc4	-0.87	1.72E-06	5.13E-06
3436	ENSMUSG00000042492	Tbc1d10b	-0.84	1.72E-06	5.13E-06
3437	ENSMUSG00000020876	Snx11	-1.07	1.74E-06	5.16E-06
3438	ENSMUSG00000108466	Gm44771	0.82	1.76E-06	5.25E-06
3439	ENSMUSG00000038729	Akap2	-0.82	1.77E-06	5.26E-06
3440	ENSMUSG00000040883	Tmem205	0.78	1.78E-06	5.28E-06
3441	ENSMUSG00000042507	Elmsan1	-1.03	1.78E-06	5.28E-06
3442	ENSMUSG00000040813	Tex264	0.76	1.79E-06	5.31E-06
3443	ENSMUSG00000014554	Dguok	0.79	1.79E-06	5.31E-06
3444	ENSMUSG00000074264	Amy1	-0.78	1.80E-06	5.34E-06
3445	ENSMUSG00000017478	Zc3h18	-1.04	1.82E-06	5.39E-06
3446	ENSMUSG00000073131	Vma21	0.76	1.82E-06	5.39E-06
3447	ENSMUSG00000036908	Unc93b1	-0.89	1.83E-06	5.42E-06

3448	ENSMUSG00000039196	Orm1	0.76	1.83E-06	5.43E-06
3449	ENSMUSG00000022365	Derl1	0.74	1.84E-06	5.47E-06
3450	ENSMUSG00000047675	Rps8	0.93	1.85E-06	5.49E-06
3451	ENSMUSG00000010277	2610507B11Rik	-0.78	1.85E-06	5.49E-06
3452	ENSMUSG00000061482	Hist1h4d	1.26	1.86E-06	5.51E-06
3453	ENSMUSG00000024456	Diaph1	1.20	1.87E-06	5.53E-06
3454	ENSMUSG00000021175	Cdca7l	-1.21	1.88E-06	5.57E-06
3455	ENSMUSG00000032171	Pin1	0.91	1.88E-06	5.58E-06
3456	ENSMUSG00000044122	Proca1	0.85	1.90E-06	5.62E-06
3457	ENSMUSG00000085169	Gm10785	1.11	1.91E-06	5.64E-06
3458	ENSMUSG00000036459	Wtip	-1.26	1.91E-06	5.64E-06
3459	ENSMUSG00000041096	Tspyl2	-1.08	1.91E-06	5.65E-06
3460	ENSMUSG00000049470	Aff4	-0.80	1.91E-06	5.66E-06
3461	ENSMUSG00000029084	Cd38	-1.20	1.91E-06	5.66E-06
3462	ENSMUSG00000026121	Sema4c	-1.47	1.92E-06	5.67E-06
3463	ENSMUSG00000071359	Tbpl1	1.12	1.93E-06	5.69E-06
3464	ENSMUSG00000039623	Ap5z1	-1.01	1.93E-06	5.69E-06
3465	ENSMUSG00000001054	Rmnd5b	-0.81	1.94E-06	5.73E-06
3466	ENSMUSG00000001632	Brpf1	-0.97	1.99E-06	5.86E-06
3467	ENSMUSG00000009394	Syn2	-0.99	2.01E-06	5.93E-06
3468	ENSMUSG00000024359	Hspa9	0.74	2.02E-06	5.95E-06
3469	ENSMUSG00000038633	Degs1	-0.72	2.02E-06	5.95E-06
3470	ENSMUSG00000041278	Ttc1	0.82	2.04E-06	6.00E-06
3471	ENSMUSG00000035473	Galm	0.74	2.04E-06	6.00E-06
3472	ENSMUSG00000000275	Trim25	-1.16	2.04E-06	6.02E-06
3473	ENSMUSG00000002319	Ipo4	-0.88	2.06E-06	6.07E-06
3474	ENSMUSG00000029762	Akr1b8	0.89	2.07E-06	6.09E-06
3475	ENSMUSG00000040151	Hs2st1	-1.19	2.07E-06	6.09E-06
3476	ENSMUSG00000050965	Prkca	-1.41	2.07E-06	6.10E-06
3477	ENSMUSG00000032127	Vps11	-0.77	2.08E-06	6.11E-06
3478	ENSMUSG00000033096	Apmap	0.72	2.09E-06	6.14E-06
3479	ENSMUSG00000031133	Arhgef6	-0.90	2.11E-06	6.19E-06
3480	ENSMUSG00000004035	Gstm7	1.01	2.11E-06	6.19E-06
3481	ENSMUSG00000031897	Psmb10	-0.81	2.11E-06	6.21E-06
3482	ENSMUSG00000038781	Stap2	1.44	2.11E-06	6.21E-06
3483	ENSMUSG00000027513	Pck1	0.89	2.13E-06	6.25E-06
3484	ENSMUSG00000057440	Mpp7	-0.84	2.14E-06	6.27E-06
3485	ENSMUSG00000029017	Pmpcb	0.75	2.14E-06	6.28E-06
3486	ENSMUSG00000000441	Raf1	-0.84	2.15E-06	6.29E-06
3487	ENSMUSG00000030980	Knop1	1.00	2.15E-06	6.32E-06
3488	ENSMUSG00000042396	Rbm7	0.72	2.16E-06	6.34E-06
3489	ENSMUSG00000008690	Ncaph2	0.71	2.17E-06	6.37E-06
3490	ENSMUSG00000107379	Gm43126	-1.02	2.18E-06	6.38E-06
3491	ENSMUSG00000029048	Rer1	0.71	2.18E-06	6.39E-06
3492	ENSMUSG00000058396	Gpr182	-1.25	2.19E-06	6.42E-06
3493	ENSMUSG00000021044	Adck1	0.74	2.20E-06	6.43E-06
3494	ENSMUSG00000004085	Map3k20	-0.90	2.20E-06	6.45E-06
3495	ENSMUSG00000001105	Ift20	0.97	2.20E-06	6.45E-06
3496	ENSMUSG00000100164	2610306M01Rik	1.09	2.21E-06	6.45E-06
3497	ENSMUSG00000078656	Vps25	0.72	2.22E-06	6.49E-06

3498	ENSMUSG00000079242	C730034F03Rik	1.26	2.24E-06	6.55E-06
3499	ENSMUSG00000005936	Kctd20	-0.78	2.25E-06	6.57E-06
3500	ENSMUSG00000030201	Lrp6	-0.85	2.25E-06	6.58E-06
3501	ENSMUSG00000027198	Ext2	-0.82	2.31E-06	6.75E-06
3502	ENSMUSG00000032329	Hmg20a	-0.99	2.31E-06	6.75E-06
3503	ENSMUSG00000020696	Rffl	-0.99	2.32E-06	6.79E-06
3504	ENSMUSG00000021171	Esyt2	-1.04	2.33E-06	6.80E-06
3505	ENSMUSG00000027079	Clp1	0.99	2.33E-06	6.81E-06
3506	ENSMUSG00000074170	Plekhf1	1.08	2.34E-06	6.82E-06
3507	ENSMUSG00000054302	Eapp	0.92	2.34E-06	6.83E-06
3508	ENSMUSG00000025357	Dgka	-1.15	2.35E-06	6.85E-06
3509	ENSMUSG00000034612	Chst11	1.19	2.35E-06	6.85E-06
3510	ENSMUSG00000042548	Asxl1	-1.17	2.38E-06	6.92E-06
3511	ENSMUSG00000029777	Gars	0.69	2.39E-06	6.96E-06
3512	ENSMUSG00000104445	Rhbg	-1.39	2.39E-06	6.96E-06
3513	ENSMUSG00000018932	Map2k3	-0.70	2.41E-06	7.03E-06
3514	ENSMUSG00000038604	Fam65a	-0.89	2.42E-06	7.03E-06
3515	ENSMUSG00000032116	Stt3a	-0.76	2.42E-06	7.03E-06
3516	ENSMUSG00000033365	Ipo13	-0.77	2.43E-06	7.06E-06
3517	ENSMUSG00000032386	Trip4	-0.93	2.43E-06	7.06E-06
3518	ENSMUSG00000031146	Plp2	-0.88	2.44E-06	7.10E-06
3519	ENSMUSG00000039662	Icmt	-0.75	2.44E-06	7.10E-06
3520	ENSMUSG00000028214	Gem	-1.18	2.44E-06	7.10E-06
3521	ENSMUSG00000030515	Tarsl2	0.96	2.46E-06	7.13E-06
3522	ENSMUSG00000029036	Atad3a	0.70	2.47E-06	7.17E-06
3523	ENSMUSG00000029501	Ankle2	-0.97	2.47E-06	7.18E-06
3524	ENSMUSG00000028099	Polr3c	0.77	2.49E-06	7.21E-06
3525	ENSMUSG00000039063	Echdc3	0.79	2.49E-06	7.23E-06
3526	ENSMUSG00000017734	Dbndd2	0.88	2.50E-06	7.24E-06
3527	ENSMUSG00000097353	A430046D13Rik	0.89	2.52E-06	7.29E-06
3528	ENSMUSG00000038368	Focad	-1.01	2.52E-06	7.31E-06
3529	ENSMUSG00000056537	Rlim	-0.99	2.53E-06	7.34E-06
3530	ENSMUSG00000031242	2610002M06Rik	-1.29	2.54E-06	7.34E-06
3531	ENSMUSG00000071646	Mta2	-0.72	2.54E-06	7.37E-06
3532	ENSMUSG00000027206	Cops2	0.75	2.55E-06	7.37E-06
3533	ENSMUSG00000027364	Usp50	1.18	2.57E-06	7.42E-06
3534	ENSMUSG00000030930	Chst15	-1.02	2.57E-06	7.42E-06
3535	ENSMUSG00000002477	Snrpd1	1.02	2.57E-06	7.42E-06
3536	ENSMUSG00000113471	CT030657.2	0.99	2.57E-06	7.42E-06
3537	ENSMUSG00000015290	Ubl4a	0.74	2.57E-06	7.43E-06
3538	ENSMUSG00000039246	Lyplal1	0.76	2.59E-06	7.50E-06
3539	ENSMUSG00000037624	Kcnk2	-1.15	2.60E-06	7.53E-06
3540	ENSMUSG00000038759	Nup205	-1.04	2.61E-06	7.53E-06
3541	ENSMUSG00000032679	Cd59a	0.76	2.61E-06	7.54E-06
3542	ENSMUSG00000030316	Tamm41	1.02	2.62E-06	7.56E-06
3543	ENSMUSG00000020612	Prkar1a	0.69	2.62E-06	7.57E-06
3544	ENSMUSG00000021253	Tgfb3	-1.35	2.64E-06	7.61E-06
3545	ENSMUSG00000027750	Postn	-1.60	2.64E-06	7.62E-06
3546	ENSMUSG00000024169	Ift140	-1.15	2.64E-06	7.62E-06
3547	ENSMUSG00000028980	H6pd	-0.96	2.67E-06	7.70E-06

3548	ENSMUSG00000003970	Rpl8	0.83	2.67E-06	7.71E-06
3549	ENSMUSG00000006589	Aprt	1.02	2.68E-06	7.72E-06
3550	ENSMUSG00000004798	Ulk2	-0.89	2.70E-06	7.77E-06
3551	ENSMUSG00000009035	Tmem184b	-0.90	2.70E-06	7.78E-06
3552	ENSMUSG00000020357	Flt4	-1.74	2.71E-06	7.81E-06
3553	ENSMUSG00000058558	Rpl5	0.78	2.72E-06	7.82E-06
3554	ENSMUSG00000032366	Tpm1	0.94	2.72E-06	7.83E-06
3555	ENSMUSG00000067369	Trmt2b	0.87	2.74E-06	7.88E-06
3556	ENSMUSG00000021076	Actr10	0.73	2.75E-06	7.91E-06
3557	ENSMUSG00000014426	Map3k4	-0.97	2.76E-06	7.93E-06
3558	ENSMUSG00000036632	Alg5	0.75	2.76E-06	7.94E-06
3559	ENSMUSG00000027291	Vps39	-0.82	2.77E-06	7.95E-06
3560	ENSMUSG00000020929	Eftud2	-0.78	2.78E-06	7.97E-06
3561	ENSMUSG00000016495	Plgrkt	0.85	2.79E-06	8.00E-06
3562	ENSMUSG00000004446	Bid	-1.03	2.79E-06	8.00E-06
3563	ENSMUSG00000037236	Matr3	-0.77	2.79E-06	8.00E-06
3564	ENSMUSG00000032252	Glce	-1.07	2.79E-06	8.01E-06
3565	ENSMUSG00000060475	Wtap	0.79	2.80E-06	8.02E-06
3566	ENSMUSG00000033174	Mgll	0.79	2.80E-06	8.02E-06
3567	ENSMUSG00000044864	Ankrd50	-1.22	2.82E-06	8.07E-06
3568	ENSMUSG00000061111	Fam195b	-0.74	2.82E-06	8.09E-06
3569	ENSMUSG00000052033	Pfdn4	1.32	2.83E-06	8.10E-06
3570	ENSMUSG00000045975	C2cd2	-1.03	2.83E-06	8.10E-06
3571	ENSMUSG00000033191	Tie1	-0.97	2.84E-06	8.13E-06
3572	ENSMUSG00000045679	Pqlc3	-0.92	2.85E-06	8.16E-06
3573	ENSMUSG00000014353	Tmem87b	-0.88	2.85E-06	8.17E-06
3574	ENSMUSG00000025016	Tm9sf3	-0.83	2.86E-06	8.18E-06
3575	ENSMUSG00000079507	H2-Q1	-0.85	2.86E-06	8.18E-06
3576	ENSMUSG00000049907	Rasl11b	-1.28	2.90E-06	8.29E-06
3577	ENSMUSG00000033306	Lpp	-0.97	2.90E-06	8.30E-06
3578	ENSMUSG00000032405	Pias1	-1.01	2.92E-06	8.35E-06
3579	ENSMUSG00000023147	Wrb	-0.81	2.92E-06	8.35E-06
3580	ENSMUSG00000021824	Ap3m1	-0.82	2.93E-06	8.38E-06
3581	ENSMUSG00000053080	2700081O15Rik	-1.14	2.93E-06	8.38E-06
3582	ENSMUSG00000023938	Aars2	0.83	2.95E-06	8.43E-06
3583	ENSMUSG00000019809	Pex3	0.73	2.96E-06	8.43E-06
3584	ENSMUSG00000025724	Sec11a	0.79	2.97E-06	8.47E-06
3585	ENSMUSG00000029708	Gcc1	-1.09	2.99E-06	8.52E-06
3586	ENSMUSG00000105245	Gm43076	-1.38	3.00E-06	8.54E-06
3587	ENSMUSG00000022510	Trp63	-1.47	3.00E-06	8.57E-06
3588	ENSMUSG00000037003	Tns2	-1.24	3.05E-06	8.68E-06
3589	ENSMUSG00000016087	Fli1	-1.17	3.06E-06	8.71E-06
3590	ENSMUSG00000007415	Gatad1	0.80	3.09E-06	8.81E-06
3591	ENSMUSG00000026222	Sp100	-1.20	3.11E-06	8.86E-06
3592	ENSMUSG00000029462	Vps29	0.72	3.12E-06	8.89E-06
3593	ENSMUSG00000066829	Zfp810	-1.11	3.13E-06	8.90E-06
3594	ENSMUSG00000028580	Pum1	-0.83	3.14E-06	8.93E-06
3595	ENSMUSG00000045092	S1pr1	-0.91	3.15E-06	8.97E-06
3596	ENSMUSG00000093445	Lrch4	-0.88	3.16E-06	8.99E-06
3597	ENSMUSG00000022758	P2rx6	1.00	3.23E-06	9.18E-06

3598	ENSMUSG00000039183	Nubp2	0.74	3.23E-06	9.18E-06
3599	ENSMUSG00000113529	AC159649.3	0.71	3.26E-06	9.27E-06
3600	ENSMUSG00000033557	Fam20b	-0.86	3.29E-06	9.34E-06
3601	ENSMUSG00000070953	Rabepk	0.84	3.29E-06	9.35E-06
3602	ENSMUSG00000035248	Zcchc6	-0.85	3.31E-06	9.40E-06
3603	ENSMUSG00000031434	Morc4	-1.08	3.32E-06	9.41E-06
3604	ENSMUSG00000021733	Slc4a7	-1.11	3.36E-06	9.53E-06
3605	ENSMUSG00000036867	Smad6	-1.16	3.36E-06	9.54E-06
3606	ENSMUSG00000021809	Nudt13	0.98	3.38E-06	9.58E-06
3607	ENSMUSG00000031765	Mt1	1.04	3.38E-06	9.58E-06
3608	ENSMUSG00000029201	Ugdh	-0.91	3.38E-06	9.58E-06
3609	ENSMUSG00000057315	Arhgap24	-0.96	3.38E-06	9.58E-06
3610	ENSMUSG00000044813	Shb	0.96	3.39E-06	9.59E-06
3611	ENSMUSG00000028273	Pdlim5	-0.80	3.42E-06	9.68E-06
3612	ENSMUSG00000020605	Hs1bp3	-0.95	3.42E-06	9.68E-06
3613	ENSMUSG00000032735	Ablim3	-0.90	3.42E-06	9.69E-06
3614	ENSMUSG00000038467	Chmp4b	1.01	3.42E-06	9.69E-06
3615	ENSMUSG00000006920	Ezh1	-0.99	3.43E-06	9.70E-06
3616	ENSMUSG00000063889	Crem	0.90	3.43E-06	9.70E-06
3617	ENSMUSG00000070044	Fam149a	-0.92	3.43E-06	9.71E-06
3618	ENSMUSG00000004096	Cwc15	0.78	3.45E-06	9.74E-06
3619	ENSMUSG00000070972	Dnajc25	0.90	3.47E-06	9.82E-06
3620	ENSMUSG00000039765	Cc2d2a	-1.24	3.50E-06	9.88E-06
3621	ENSMUSG00000017677	Wsb1	-1.10	3.50E-06	9.89E-06
3622	ENSMUSG00000024963	Dnajc4	0.74	3.51E-06	9.90E-06
3623	ENSMUSG00000079671	2610203C22Rik	-0.72	3.51E-06	9.92E-06
3624	ENSMUSG00000003200	Sh3gl1	-0.81	3.51E-06	9.92E-06
3625	ENSMUSG00000057411	Fam173a	0.78	3.52E-06	9.93E-06
3626	ENSMUSG00000028567	Txndc12	0.95	3.52E-06	9.93E-06
3627	ENSMUSG00000001138	Cnnm3	-0.92	3.52E-06	9.93E-06
3628	ENSMUSG00000014077	Chp1	-0.78	3.53E-06	9.94E-06
3629	ENSMUSG00000041779	Tram2	-1.15	3.54E-06	9.97E-06
3630	ENSMUSG00000084974	Gm15567	-0.90	3.55E-06	1.00E-05
3631	ENSMUSG00000026944	Abca2	-0.85	3.56E-06	1.00E-05
3632	ENSMUSG00000032594	Ip6k1	-0.80	3.57E-06	1.01E-05
3633	ENSMUSG00000022967	Ifnar1	-0.82	3.62E-06	1.02E-05
3634	ENSMUSG00000026836	Acvr1	-1.44	3.62E-06	1.02E-05
3635	ENSMUSG00000021482	Aaed1	0.71	3.64E-06	1.02E-05
3636	ENSMUSG00000008435	Rdh13	0.95	3.64E-06	1.03E-05
3637	ENSMUSG00000029720	Gm20605	-0.87	3.66E-06	1.03E-05
3638	ENSMUSG00000031521	Aga	0.70	3.67E-06	1.03E-05
3639	ENSMUSG00000043019	Edem3	-1.00	3.67E-06	1.03E-05
3640	ENSMUSG00000038608	Dock10	-1.38	3.67E-06	1.03E-05
3641	ENSMUSG00000026857	Ntmt1	0.81	3.68E-06	1.03E-05
3642	ENSMUSG00000084957	Bbip1	0.88	3.71E-06	1.04E-05
3643	ENSMUSG00000028737	Aldh4a1	-0.78	3.71E-06	1.04E-05
3644	ENSMUSG00000017713	Tha1	0.96	3.71E-06	1.04E-05
3645	ENSMUSG00000078813	Leng1	0.87	3.74E-06	1.05E-05
3646	ENSMUSG00000039096	Rsad1	1.20	3.74E-06	1.05E-05
3647	ENSMUSG00000037885	Stk35	-0.96	3.78E-06	1.06E-05

3648	ENSMUSG00000021185	9030617O03Rik	-0.77	3.79E-06	1.06E-05
3649	ENSMUSG00000039156	Stim2	-1.13	3.83E-06	1.07E-05
3650	ENSMUSG00000028452	Vcp	-0.70	3.84E-06	1.08E-05
3651	ENSMUSG00000031954	Cfdp1	0.85	3.85E-06	1.08E-05
3652	ENSMUSG00000015224	Cyp2j9	-1.19	3.88E-06	1.09E-05
3653	ENSMUSG00000092470	Gm20518	-0.93	3.88E-06	1.09E-05
3654	ENSMUSG00000020519	Sap30l	0.86	3.89E-06	1.09E-05
3655	ENSMUSG00000026509	Capn2	-0.72	3.91E-06	1.09E-05
3656	ENSMUSG00000015889	Lta4h	-0.87	3.91E-06	1.09E-05
3657	ENSMUSG00000097162	2310010J17Rik	1.18	3.91E-06	1.09E-05
3658	ENSMUSG00000091514	Gm17484	-0.87	3.91E-06	1.09E-05
3659	ENSMUSG00000026914	Psmd14	0.68	3.93E-06	1.10E-05
3660	ENSMUSG00000027104	Atf2	0.84	3.93E-06	1.10E-05
3661	ENSMUSG00000060510	Zfp266	-0.99	3.94E-06	1.10E-05
3662	ENSMUSG00000048402	Gli2	-1.26	3.96E-06	1.11E-05
3663	ENSMUSG00000031311	Nono	-0.72	3.98E-06	1.11E-05
3664	ENSMUSG00000027506	Tpd52	1.01	3.99E-06	1.11E-05
3665	ENSMUSG00000025730	Rab40c	-1.03	4.00E-06	1.12E-05
3666	ENSMUSG00000032192	Gnb5	-0.79	4.01E-06	1.12E-05
3667	ENSMUSG00000024039	Cbs	-1.23	4.02E-06	1.12E-05
3668	ENSMUSG00000037902	Sirpa	-0.84	4.07E-06	1.14E-05
3669	ENSMUSG00000038332	Sesn1	-1.14	4.11E-06	1.15E-05
3670	ENSMUSG00000086289	Gm16933	-1.11	4.11E-06	1.15E-05
3671	ENSMUSG00000037110	Ralgapa2	-0.80	4.12E-06	1.15E-05
3672	ENSMUSG00000052144	Ppp4r2	0.79	4.12E-06	1.15E-05
3673	ENSMUSG00000024160	Spsb3	0.76	4.20E-06	1.17E-05
3674	ENSMUSG00000028199	Cryz	0.80	4.22E-06	1.18E-05
3675	ENSMUSG00000022307	Oxr1	-0.96	4.24E-06	1.18E-05
3676	ENSMUSG00000027533	Fabp5	0.89	4.28E-06	1.19E-05
3677	ENSMUSG00000021819	Zswim8	-0.91	4.28E-06	1.19E-05
3678	ENSMUSG00000037260	Hgsnat	-0.70	4.29E-06	1.19E-05
3679	ENSMUSG00000012422	Tmem167	0.74	4.29E-06	1.19E-05
3680	ENSMUSG00000027845	Dclre1b	1.17	4.31E-06	1.20E-05
3681	ENSMUSG00000026655	Fam107b	-1.01	4.31E-06	1.20E-05
3682	ENSMUSG00000097080	1700086O06Rik	1.11	4.32E-06	1.20E-05
3683	ENSMUSG00000022673	Mcm4	-1.26	4.33E-06	1.20E-05
3684	ENSMUSG00000107319	Gm42670	0.92	4.33E-06	1.20E-05
3685	ENSMUSG00000018171	Vmp1	0.69	4.33E-06	1.20E-05
3686	ENSMUSG00000019990	Pde7b	1.23	4.36E-06	1.21E-05
3687	ENSMUSG00000031902	Nfatc3	-0.75	4.37E-06	1.21E-05
3688	ENSMUSG00000028073	Pear1	-1.28	4.38E-06	1.21E-05
3689	ENSMUSG00000073435	Nme3	0.98	4.40E-06	1.22E-05
3690	ENSMUSG00000006095	Tbcb	0.71	4.40E-06	1.22E-05
3691	ENSMUSG00000048603	Gm9828	0.81	4.44E-06	1.23E-05
3692	ENSMUSG00000022634	Yaf2	0.71	4.48E-06	1.24E-05
3693	ENSMUSG00000031227	Magee1	-1.13	4.49E-06	1.24E-05
3694	ENSMUSG00000002015	Bcap31	0.69	4.50E-06	1.25E-05
3695	ENSMUSG00000033596	Rfwd3	-0.97	4.52E-06	1.25E-05
3696	ENSMUSG00000022096	Hr	-0.85	4.54E-06	1.26E-05
3697	ENSMUSG00000022822	Abcc5	-0.98	4.58E-06	1.27E-05

3698	ENSMUSG00000067150	Xpo5	-0.94	4.59E-06	1.27E-05
3699	ENSMUSG00000028668	Eloa	-0.81	4.61E-06	1.27E-05
3700	ENSMUSG00000035799	Twist1	-1.17	4.61E-06	1.27E-05
3701	ENSMUSG00000021699	Pde4d	1.00	4.62E-06	1.28E-05
3702	ENSMUSG00000027669	Gnb4	-1.16	4.63E-06	1.28E-05
3703	ENSMUSG00000027900	Dram2	0.70	4.66E-06	1.29E-05
3704	ENSMUSG00000036565	Ttyh3	-1.12	4.67E-06	1.29E-05
3705	ENSMUSG00000089774	Slc5a3	-0.73	4.69E-06	1.29E-05
3706	ENSMUSG00000030035	Wbp1	0.74	4.71E-06	1.30E-05
3707	ENSMUSG00000021033	Gstz1	-0.75	4.71E-06	1.30E-05
3708	ENSMUSG00000028179	Cth	-1.19	4.72E-06	1.30E-05
3709	ENSMUSG00000041058	Wwp1	0.75	4.72E-06	1.30E-05
3710	ENSMUSG00000006386	Tek	-1.12	4.74E-06	1.31E-05
3711	ENSMUSG00000027006	Dnajc10	-0.88	4.75E-06	1.31E-05
3712	ENSMUSG00000034109	Golim4	-0.90	4.75E-06	1.31E-05
3713	ENSMUSG00000025949	Pikfyve	-1.00	4.79E-06	1.32E-05
3714	ENSMUSG00000049401	Ogfr	-0.86	4.79E-06	1.32E-05
3715	ENSMUSG00000055932	Fto	-0.79	4.80E-06	1.32E-05
3716	ENSMUSG00000032898	Fbxo21	-1.23	4.82E-06	1.33E-05
3717	ENSMUSG00000032897	Nfyc	0.71	4.82E-06	1.33E-05
3718	ENSMUSG00000033917	Gde1	0.71	4.83E-06	1.33E-05
3719	ENSMUSG00000036591	Arhgap21	-0.94	4.88E-06	1.34E-05
3720	ENSMUSG00000020457	Drg1	0.70	4.89E-06	1.34E-05
3721	ENSMUSG00000090084	Srpx	-1.27	4.90E-06	1.35E-05
3722	ENSMUSG00000024006	Stk38	-0.70	4.90E-06	1.35E-05
3723	ENSMUSG00000023018	Smarcd1	0.99	4.90E-06	1.35E-05
3724	ENSMUSG00000027272	Ubr1	-1.00	4.94E-06	1.36E-05
3725	ENSMUSG00000025982	Sf3b1	-0.68	4.96E-06	1.36E-05
3726	ENSMUSG00000007833	Aldh16a1	0.71	4.98E-06	1.37E-05
3727	ENSMUSG00000021143	Pacs2	-0.93	5.04E-06	1.38E-05
3728	ENSMUSG00000032733	Snx33	-1.00	5.06E-06	1.39E-05
3729	ENSMUSG00000028970	Abcb1b	-1.32	5.10E-06	1.40E-05
3730	ENSMUSG00000097730	Gm26588	0.68	5.13E-06	1.41E-05
3731	ENSMUSG00000018474	Chd3	1.03	5.14E-06	1.41E-05
3732	ENSMUSG00000037331	Larp1	-0.71	5.15E-06	1.41E-05
3733	ENSMUSG00000020719	Ddx5	-0.71	5.19E-06	1.42E-05
3734	ENSMUSG00000020788	Atp2a3	-1.11	5.19E-06	1.42E-05
3735	ENSMUSG00000022797	Tfrc	1.01	5.21E-06	1.43E-05
3736	ENSMUSG00000029798	Herc6	-1.44	5.21E-06	1.43E-05
3737	ENSMUSG00000028451	1700022I11Rik	-0.68	5.23E-06	1.43E-05
3738	ENSMUSG00000031533	Mrps31	0.84	5.24E-06	1.43E-05
3739	ENSMUSG00000105875	Gm43518	1.01	5.31E-06	1.45E-05
3740	ENSMUSG00000014444	Piezo1	-0.77	5.36E-06	1.47E-05
3741	ENSMUSG00000026097	Ormdl1	0.99	5.39E-06	1.47E-05
3742	ENSMUSG00000025743	Sdc3	-0.91	5.39E-06	1.47E-05
3743	ENSMUSG00000036452	Arhgap26	0.88	5.40E-06	1.47E-05
3744	ENSMUSG00000030921	Trim30a	-1.21	5.40E-06	1.47E-05
3745	ENSMUSG00000023806	Rsph3b	0.81	5.43E-06	1.48E-05
3746	ENSMUSG00000024747	Aldh1a7	-0.81	5.43E-06	1.48E-05
3747	ENSMUSG00000044763	Trmt10c	0.87	5.45E-06	1.49E-05

3748	ENSMUSG00000041215	Yeats2	-1.35	5.47E-06	1.49E-05
3749	ENSMUSG00000042506	Usp22	-0.83	5.51E-06	1.50E-05
3750	ENSMUSG00000042042	Csgalnact2	-1.05	5.53E-06	1.51E-05
3751	ENSMUSG00000041815	Poldip3	-0.73	5.54E-06	1.51E-05
3752	ENSMUSG00000002103	Acp2	-0.72	5.55E-06	1.51E-05
3753	ENSMUSG00000031917	Nip7	0.91	5.55E-06	1.51E-05
3754	ENSMUSG00000057193	Slc44a2	-0.97	5.57E-06	1.52E-05
3755	ENSMUSG00000035437	Rabgap1	-0.85	5.60E-06	1.53E-05
3756	ENSMUSG00000030086	Chchd6	0.69	5.62E-06	1.53E-05
3757	ENSMUSG00000023456	Tpi1	0.74	5.63E-06	1.53E-05
3758	ENSMUSG00000031371	Haus7	-0.89	5.66E-06	1.54E-05
3759	ENSMUSG00000029162	Khk	0.83	5.67E-06	1.54E-05
3760	ENSMUSG00000051853	Arf3	-0.81	5.73E-06	1.56E-05
3761	ENSMUSG00000037722	Gnpnat1	0.83	5.74E-06	1.56E-05
3762	ENSMUSG00000087370	Tmem170b	-1.05	5.76E-06	1.56E-05
3763	ENSMUSG00000031221	Igbp1	0.89	5.76E-06	1.57E-05
3764	ENSMUSG00000066026	Dhrs3	-0.71	5.79E-06	1.57E-05
3765	ENSMUSG00000030002	Dusp11	-0.81	5.80E-06	1.57E-05
3766	ENSMUSG00000006442	Srm	-0.75	5.84E-06	1.59E-05
3767	ENSMUSG00000014592	Camta1	1.09	5.86E-06	1.59E-05
3768	ENSMUSG00000035024	Ncapd3	-1.18	5.88E-06	1.60E-05
3769	ENSMUSG00000028964	Park7	0.74	5.90E-06	1.60E-05
3770	ENSMUSG00000021068	Nin	-1.19	5.96E-06	1.62E-05
3771	ENSMUSG00000019863	Qrsl1	0.94	5.97E-06	1.62E-05
3772	ENSMUSG00000032377	Plscr4	-1.06	5.98E-06	1.62E-05
3773	ENSMUSG00000052298	Cdc42se2	-0.78	6.00E-06	1.63E-05
3774	ENSMUSG00000069678	Pcgf1	0.99	6.02E-06	1.63E-05
3775	ENSMUSG00000023345	Poc1a	0.99	6.06E-06	1.64E-05
3776	ENSMUSG00000036206	Sh3bp4	-1.10	6.06E-06	1.64E-05
3777	ENSMUSG00000020439	Smtn	-1.14	6.13E-06	1.66E-05
3778	ENSMUSG00000053226	Dand5	0.90	6.13E-06	1.66E-05
3779	ENSMUSG00000029028	Lrrc47	-0.76	6.15E-06	1.66E-05
3780	ENSMUSG00000038417	Fig4	-0.82	6.17E-06	1.67E-05
3781	ENSMUSG00000038550	Ciart	1.44	6.17E-06	1.67E-05
3782	ENSMUSG00000028641	P3h1	-1.06	6.27E-06	1.70E-05
3783	ENSMUSG00000051238	Swsap1	0.89	6.31E-06	1.70E-05
3784	ENSMUSG00000029163	Emilin1	-1.13	6.34E-06	1.71E-05
3785	ENSMUSG00000036646	Man1b1	-0.90	6.42E-06	1.73E-05
3786	ENSMUSG00000046897	Zfp740	-0.89	6.42E-06	1.74E-05
3787	ENSMUSG00000024622	Hmgxb3	-1.04	6.43E-06	1.74E-05
3788	ENSMUSG00000092021	Gbp11	-1.25	6.44E-06	1.74E-05
3789	ENSMUSG00000078348	Sf3b5	0.87	6.44E-06	1.74E-05
3790	ENSMUSG00000041577	Prelp	-0.84	6.44E-06	1.74E-05
3791	ENSMUSG00000029547	Ints1	-0.86	6.45E-06	1.74E-05
3792	ENSMUSG00000030103	Bhlhe40	-0.72	6.45E-06	1.74E-05
3793	ENSMUSG00000031546	Gins4	0.73	6.45E-06	1.74E-05
3794	ENSMUSG00000024537	Psmg2	0.87	6.48E-06	1.75E-05
3795	ENSMUSG00000029761	Cald1	-0.82	6.51E-06	1.75E-05
3796	ENSMUSG00000028108	Ecm1	-1.28	6.56E-06	1.77E-05
3797	ENSMUSG00000026670	Uap1	-0.81	6.68E-06	1.80E-05

3798	ENSMUSG00000038742	Angptl6	1.04	6.69E-06	1.80E-05
3799	ENSMUSG00000015542	Nat9	0.78	6.70E-06	1.80E-05
3800	ENSMUSG00000040774	Cept1	-0.82	6.74E-06	1.81E-05
3801	ENSMUSG00000020077	Srgn	-0.91	6.78E-06	1.82E-05
3802	ENSMUSG00000052305	Hbb-bs	0.86	6.81E-06	1.83E-05
3803	ENSMUSG00000062609	Kcnj15	-1.10	6.86E-06	1.85E-05
3804	ENSMUSG00000096740	Lbhd1	0.76	6.90E-06	1.86E-05
3805	ENSMUSG00000036513	Commd2	0.82	6.91E-06	1.86E-05
3806	ENSMUSG00000072568	Fam84b	-1.15	6.94E-06	1.86E-05
3807	ENSMUSG00000026335	Pam	-1.08	6.98E-06	1.87E-05
3808	ENSMUSG00000038936	Sccpdh	0.68	6.98E-06	1.87E-05
3809	ENSMUSG00000042642	Flad1	0.68	6.98E-06	1.88E-05
3810	ENSMUSG00000001034	Mapk7	-1.16	6.99E-06	1.88E-05
3811	ENSMUSG00000028800	Hdac1	-0.71	7.00E-06	1.88E-05
3812	ENSMUSG00000038180	Spag4	1.08	7.03E-06	1.89E-05
3813	ENSMUSG00000008305	Tle1	-0.96	7.09E-06	1.90E-05
3814	ENSMUSG00000063275	Hacd1	0.86	7.10E-06	1.90E-05
3815	ENSMUSG00000031169	Porcn	-1.05	7.11E-06	1.90E-05
3816	ENSMUSG00000102234	Gm37885	-0.73	7.11E-06	1.90E-05
3817	ENSMUSG00000054434	Tmem120b	-0.81	7.12E-06	1.91E-05
3818	ENSMUSG00000026353	Ubxn4	0.71	7.16E-06	1.92E-05
3819	ENSMUSG00000026083	Eif5b	0.97	7.17E-06	1.92E-05
3820	ENSMUSG00000039958	Etfbkmt	0.95	7.19E-06	1.93E-05
3821	ENSMUSG00000017756	Slc12a7	-0.82	7.19E-06	1.93E-05
3822	ENSMUSG00000061816	Myl1	0.80	7.25E-06	1.94E-05
3823	ENSMUSG00000079557	March2	0.68	7.33E-06	1.96E-05
3824	ENSMUSG00000019578	Ubxn6	0.68	7.42E-06	1.98E-05
3825	ENSMUSG00000023977	Ubr2	-0.78	7.46E-06	1.99E-05
3826	ENSMUSG00000016206	H2-M3	-1.17	7.48E-06	2.00E-05
3827	ENSMUSG00000039842	Mcph1	-1.20	7.51E-06	2.01E-05
3828	ENSMUSG00000074748	Atxn7l3b	0.72	7.55E-06	2.02E-05
3829	ENSMUSG00000033985	Tesk2	-0.94	7.58E-06	2.02E-05
3830	ENSMUSG00000038127	Ccdc50	-0.78	7.59E-06	2.03E-05
3831	ENSMUSG00000002808	Epdr1	-1.02	7.60E-06	2.03E-05
3832	ENSMUSG00000085875	Gm12905	1.07	7.64E-06	2.04E-05
3833	ENSMUSG00000079018	Ly6c1	-0.87	7.65E-06	2.04E-05
3834	ENSMUSG00000022816	Fstl1	-1.25	7.65E-06	2.04E-05
3835	ENSMUSG00000079057	Cyp4v3	-0.74	7.68E-06	2.05E-05
3836	ENSMUSG00000038375	Trp53inp2	-0.73	7.72E-06	2.06E-05
3837	ENSMUSG00000028603	Scp2	0.68	7.72E-06	2.06E-05
3838	ENSMUSG00000032293	Ireb2	-0.91	7.76E-06	2.07E-05
3839	ENSMUSG00000001642	Akr1b3	0.67	7.88E-06	2.10E-05
3840	ENSMUSG00000031201	Brcc3	0.71	7.89E-06	2.10E-05
3841	ENSMUSG00000028618	Tmem59	0.65	7.89E-06	2.10E-05
3842	ENSMUSG00000019872	Smpdl3a	0.67	7.92E-06	2.11E-05
3843	ENSMUSG00000032872	Cyb5r4	0.78	7.92E-06	2.11E-05
3844	ENSMUSG00000033083	Tbc1d4	-0.88	7.95E-06	2.12E-05
3845	ENSMUSG00000024613	Tcof1	-1.05	7.98E-06	2.12E-05
3846	ENSMUSG00000039182	AW209491	0.90	7.98E-06	2.12E-05
3847	ENSMUSG00000042570	Mier2	-1.04	8.02E-06	2.13E-05

3848	ENSMUSG00000032199	Polr2m	0.63	8.06E-06	2.14E-05
3849	ENSMUSG00000085563	2210411M09Rik	-1.18	8.06E-06	2.14E-05
3850	ENSMUSG00000021665	Hexb	-0.87	8.07E-06	2.14E-05
3851	ENSMUSG00000060450	Rnf14	-0.72	8.07E-06	2.14E-05
3852	ENSMUSG00000012114	Med15	-0.77	8.09E-06	2.15E-05
3853	ENSMUSG00000055053	Nfic	-0.67	8.09E-06	2.15E-05
3854	ENSMUSG00000020708	Psmc5	0.71	8.13E-06	2.16E-05
3855	ENSMUSG00000047061	Gm9817	1.07	8.17E-06	2.17E-05
3856	ENSMUSG00000025757	Hspa4l	1.01	8.18E-06	2.17E-05
3857	ENSMUSG00000046546	Fam43a	-1.10	8.21E-06	2.18E-05
3858	ENSMUSG00000025374	Nabp2	0.71	8.21E-06	2.18E-05
3859	ENSMUSG00000028552	Eps15	-1.00	8.24E-06	2.18E-05
3860	ENSMUSG00000052337	Immt	0.67	8.28E-06	2.19E-05
3861	ENSMUSG00000028007	Snx7	-0.83	8.29E-06	2.20E-05
3862	ENSMUSG00000035152	Ap2b1	-0.77	8.34E-06	2.21E-05
3863	ENSMUSG00000050332	Amer1	-1.12	8.45E-06	2.24E-05
3864	ENSMUSG00000042211	Fbxo38	-0.95	8.47E-06	2.24E-05
3865	ENSMUSG00000045664	Cdc42ep2	-1.20	8.48E-06	2.24E-05
3866	ENSMUSG00000097564	C430014B12Rik	0.86	8.48E-06	2.24E-05
3867	ENSMUSG00000024908	Ppp6r3	-0.80	8.51E-06	2.25E-05
3868	ENSMUSG00000044674	Fzd1	-1.04	8.51E-06	2.25E-05
3869	ENSMUSG00000068290	Ddrgk1	0.69	8.53E-06	2.26E-05
3870	ENSMUSG00000025132	Arhgdia	-0.66	8.56E-06	2.26E-05
3871	ENSMUSG00000024889	Rce1	0.78	8.64E-06	2.28E-05
3872	ENSMUSG00000041859	Mcm3	-1.07	8.65E-06	2.28E-05
3873	ENSMUSG00000025237	Parp6	0.77	8.68E-06	2.29E-05
3874	ENSMUSG00000021140	Pcnx	-0.91	8.69E-06	2.29E-05
3875	ENSMUSG00000022803	Popdc2	-0.92	8.72E-06	2.30E-05
3876	ENSMUSG00000058385	Hist1h2bg	1.12	8.80E-06	2.32E-05
3877	ENSMUSG00000055720	Ubl7	0.66	8.81E-06	2.32E-05
3878	ENSMUSG00000047371	Zfp768	0.77	8.86E-06	2.34E-05
3879	ENSMUSG00000021311	Mtr	-1.05	8.96E-06	2.36E-05
3880	ENSMUSG00000050912	Tmem123	-0.76	8.99E-06	2.37E-05
3881	ENSMUSG00000025779	Ly96	1.12	9.03E-06	2.38E-05
3882	ENSMUSG00000022131	Gpr180	-0.78	9.03E-06	2.38E-05
3883	ENSMUSG00000102836	Gm37013	-1.14	9.10E-06	2.40E-05
3884	ENSMUSG00000103125	Gm37388	-1.14	9.10E-06	2.40E-05
3885	ENSMUSG00000097582	Gm26527	-0.84	9.13E-06	2.40E-05
3886	ENSMUSG00000036977	Anapc10	1.05	9.15E-06	2.41E-05
3887	ENSMUSG00000020097	Sgpl1	0.70	9.18E-06	2.42E-05
3888	ENSMUSG00000022940	Pigp	0.72	9.23E-06	2.43E-05
3889	ENSMUSG00000034560	Washc4	-0.99	9.24E-06	2.43E-05
3890	ENSMUSG00000019726	Lyst	-1.08	9.26E-06	2.44E-05
3891	ENSMUSG00000111348	AC133947.2	-1.06	9.27E-06	2.44E-05
3892	ENSMUSG00000024812	Tjp2	-1.05	9.32E-06	2.45E-05
3893	ENSMUSG00000028252	Ccnc	0.77	9.34E-06	2.45E-05
3894	ENSMUSG00000028164	Manba	-1.08	9.43E-06	2.48E-05
3895	ENSMUSG00000090207	4930524O07Rik	1.02	9.44E-06	2.48E-05
3896	ENSMUSG00000001420	Tmem79	1.13	9.44E-06	2.48E-05
3897	ENSMUSG00000022584	Ly6c2	-0.93	9.54E-06	2.50E-05

3898	ENSMUSG00000024387	Csnk2b	0.73	9.59E-06	2.52E-05
3899	ENSMUSG00000066151	Fkbp15	-0.90	9.60E-06	2.52E-05
3900	ENSMUSG00000050271	Prag1	-1.16	9.62E-06	2.52E-05
3901	ENSMUSG00000105703	Gm43305	1.16	9.63E-06	2.52E-05
3902	ENSMUSG00000038564	Ift172	-0.95	9.65E-06	2.53E-05
3903	ENSMUSG00000020091	Eif4ebp2	0.70	9.66E-06	2.53E-05
3904	ENSMUSG00000024483	Ankhd1	-0.83	9.67E-06	2.53E-05
3905	ENSMUSG00000030805	Stx4a	0.83	9.70E-06	2.54E-05
3906	ENSMUSG00000025871	4833439L19Rik	0.68	9.72E-06	2.55E-05
3907	ENSMUSG00000025911	Adhfe1	-0.66	9.73E-06	2.55E-05
3908	ENSMUSG00000025795	Rassf3	0.87	9.76E-06	2.56E-05
3909	ENSMUSG00000021994	Wnt5a	-0.88	9.80E-06	2.56E-05
3910	ENSMUSG00000057506	Bloc1s2	1.07	9.81E-06	2.57E-05
3911	ENSMUSG00000043131	Mob1a	-0.77	9.83E-06	2.57E-05
3912	ENSMUSG00000090213	Tmem189	0.70	9.83E-06	2.57E-05
3913	ENSMUSG00000062234	Gak	-0.75	9.84E-06	2.57E-05
3914	ENSMUSG00000020834	Dhrs13	1.22	9.86E-06	2.58E-05
3915	ENSMUSG00000036097	Fam178a	-1.16	9.90E-06	2.59E-05
3916	ENSMUSG00000021731	Mrps30	0.67	9.91E-06	2.59E-05
3917	ENSMUSG00000019948	Actr6	0.96	9.93E-06	2.59E-05
3918	ENSMUSG00000050947	Amigo1	0.90	9.96E-06	2.60E-05
3919	ENSMUSG00000002504	Slc9a3r2	-0.73	1.01E-05	2.63E-05
3920	ENSMUSG00000032305	Fam219b	-0.88	1.01E-05	2.64E-05
3921	ENSMUSG00000044502	Bod1	0.69	1.02E-05	2.65E-05
3922	ENSMUSG00000024952	Rps6ka4	-0.73	1.02E-05	2.67E-05
3923	ENSMUSG00000035186	Ubd	-1.15	1.03E-05	2.68E-05
3924	ENSMUSG00000060029	4930473A02Rik	-0.78	1.03E-05	2.69E-05
3925	ENSMUSG00000017418	Arl5b	-1.11	1.03E-05	2.69E-05
3926	ENSMUSG00000032180	Tmed1	0.76	1.04E-05	2.71E-05
3927	ENSMUSG00000022201	Zfr	-0.67	1.04E-05	2.71E-05
3928	ENSMUSG00000020064	Herc4	-0.95	1.04E-05	2.71E-05
3929	ENSMUSG00000028076	Cd1d1	-0.65	1.04E-05	2.71E-05
3930	ENSMUSG00000031955	Bcar1	-0.72	1.05E-05	2.72E-05
3931	ENSMUSG00000035666	Gtf3c4	-0.95	1.05E-05	2.74E-05
3932	ENSMUSG00000025521	Tmem192	0.72	1.07E-05	2.78E-05
3933	ENSMUSG00000022010	Tsc22d1	0.90	1.08E-05	2.79E-05
3934	ENSMUSG00000025666	Tmem47	-1.11	1.08E-05	2.81E-05
3935	ENSMUSG00000104453	Gm37829	-1.05	1.08E-05	2.81E-05
3936	ENSMUSG00000027583	Zbtb46	-0.97	1.08E-05	2.81E-05
3937	ENSMUSG00000109644	0610005C13Rik	-1.08	1.08E-05	2.82E-05
3938	ENSMUSG00000015202	Cnksr3	-1.16	1.09E-05	2.82E-05
3939	ENSMUSG00000024072	Yipf4	0.70	1.09E-05	2.82E-05
3940	ENSMUSG00000032458	Copb2	-0.67	1.09E-05	2.83E-05
3941	ENSMUSG00000061374	Fiz1	-0.79	1.10E-05	2.84E-05
3942	ENSMUSG00000068798	Rap1a	0.73	1.10E-05	2.84E-05
3943	ENSMUSG00000032624	Eml4	-1.06	1.10E-05	2.85E-05
3944	ENSMUSG00000032840	2410131K14Rik	0.79	1.10E-05	2.86E-05
3945	ENSMUSG00000038515	Grtp1	0.63	1.11E-05	2.87E-05
3946	ENSMUSG00000054611	Kdm2a	-0.78	1.11E-05	2.88E-05
3947	ENSMUSG00000023923	Tbc1d5	-0.92	1.11E-05	2.88E-05

3948	ENSMUSG00000054226	Tprkb	0.97	1.11E-05	2.88E-05
3949	ENSMUSG00000040584	Abcb1a	-1.17	1.11E-05	2.88E-05
3950	ENSMUSG00000060675	Pla2g16	-0.68	1.12E-05	2.90E-05
3951	ENSMUSG00000004099	Dnmt1	-0.99	1.13E-05	2.92E-05
3952	ENSMUSG00000029203	Ube2k	0.68	1.13E-05	2.92E-05
3953	ENSMUSG00000040385	Ppp1ca	0.69	1.13E-05	2.92E-05
3954	ENSMUSG00000024007	Ppil1	0.90	1.13E-05	2.93E-05
3955	ENSMUSG00000060477	Irak2	-0.70	1.14E-05	2.95E-05
3956	ENSMUSG00000019297	Nop9	0.79	1.15E-05	2.96E-05
3957	ENSMUSG00000073802	Cdkn2b	-0.98	1.15E-05	2.98E-05
3958	ENSMUSG00000054091	1810037I17Rik	0.72	1.16E-05	2.98E-05
3959	ENSMUSG00000058056	Palld	-0.85	1.17E-05	3.02E-05
3960	ENSMUSG00000047710	Champ1	-1.09	1.18E-05	3.04E-05
3961	ENSMUSG00000026676	Ccdc3	-0.67	1.18E-05	3.04E-05
3962	ENSMUSG00000006728	Cdk4	0.62	1.18E-05	3.04E-05
3963	ENSMUSG00000031310	Zmym3	-1.03	1.19E-05	3.06E-05
3964	ENSMUSG00000021116	Eif2s1	0.63	1.19E-05	3.07E-05
3965	ENSMUSG00000027615	Hps3	-1.08	1.19E-05	3.08E-05
3966	ENSMUSG00000032434	Cmtm6	-0.79	1.20E-05	3.08E-05
3967	ENSMUSG00000071647	Eml3	-0.72	1.20E-05	3.08E-05
3968	ENSMUSG00000061759	Armt1	0.86	1.20E-05	3.09E-05
3969	ENSMUSG00000025280	Polr3a	-1.00	1.22E-05	3.14E-05
3970	ENSMUSG00000022707	Gbe1	0.75	1.22E-05	3.15E-05
3971	ENSMUSG00000050721	Plekho2	-1.13	1.22E-05	3.15E-05
3972	ENSMUSG00000038102	Trappc11	-0.85	1.23E-05	3.18E-05
3973	ENSMUSG00000087435	Gm16323	0.66	1.23E-05	3.18E-05
3974	ENSMUSG00000020086	H2afy2	0.83	1.24E-05	3.19E-05
3975	ENSMUSG00000031805	Jak3	-0.74	1.24E-05	3.19E-05
3976	ENSMUSG00000002052	Supt6	-0.87	1.25E-05	3.21E-05
3977	ENSMUSG00000027091	Zc3h15	0.76	1.25E-05	3.21E-05
3978	ENSMUSG00000030750	Nsmce1	0.86	1.25E-05	3.21E-05
3979	ENSMUSG00000040565	Btaf1	-0.87	1.25E-05	3.21E-05
3980	ENSMUSG00000030824	Nucb1	-0.66	1.25E-05	3.21E-05
3981	ENSMUSG00000021133	Susd6	-0.83	1.25E-05	3.22E-05
3982	ENSMUSG00000036078	Sigmar1	-0.71	1.26E-05	3.23E-05
3983	ENSMUSG00000039787	Cercam	-1.08	1.26E-05	3.23E-05
3984	ENSMUSG00000016510	Mtif3	0.69	1.27E-05	3.26E-05
3985	ENSMUSG00000032126	Hmbs	0.66	1.27E-05	3.26E-05
3986	ENSMUSG00000042185	Nfrkb	-1.04	1.28E-05	3.27E-05
3987	ENSMUSG00000073940	Hbb-bt	0.82	1.28E-05	3.27E-05
3988	ENSMUSG00000027490	E2f1	-1.09	1.28E-05	3.29E-05
3989	ENSMUSG00000043207	Zmpste24	-1.03	1.28E-05	3.29E-05
3990	ENSMUSG00000062929	Cfl2	0.64	1.28E-05	3.29E-05
3991	ENSMUSG00000038241	Cep250	-1.14	1.28E-05	3.29E-05
3992	ENSMUSG00000027598	Itch	-0.94	1.29E-05	3.29E-05
3993	ENSMUSG00000027130	Slc12a6	0.72	1.29E-05	3.29E-05
3994	ENSMUSG00000028527	Ak4	1.29	1.30E-05	3.32E-05
3995	ENSMUSG00000031068	Glrx3	0.67	1.30E-05	3.33E-05
3996	ENSMUSG00000027999	Pla2g12a	0.64	1.30E-05	3.33E-05
3997	ENSMUSG00000015944	Gatsl2	-1.13	1.31E-05	3.34E-05

3998	ENSMUSG00000044820	AY074887	0.81	1.32E-05	3.37E-05
3999	ENSMUSG00000026766	Mmadhc	0.68	1.33E-05	3.40E-05
4000	ENSMUSG00000035505	Cox18	0.81	1.33E-05	3.41E-05
4001	ENSMUSG00000097440	Gm6277	0.94	1.34E-05	3.42E-05
4002	ENSMUSG00000029545	Acads	0.68	1.34E-05	3.43E-05
4003	ENSMUSG00000054874	Pcnx3	-0.76	1.35E-05	3.44E-05
4004	ENSMUSG00000031803	B3gnt3	-1.18	1.35E-05	3.44E-05
4005	ENSMUSG00000097763	Gm26636	-0.83	1.35E-05	3.45E-05
4006	ENSMUSG00000055866	Per2	-0.99	1.35E-05	3.46E-05
4007	ENSMUSG00000085587	Gm14493	-1.08	1.35E-05	3.46E-05
4008	ENSMUSG00000024949	Sf1	-0.70	1.36E-05	3.46E-05
4009	ENSMUSG00000041926	Rnpep	-0.67	1.36E-05	3.46E-05
4010	ENSMUSG00000044308	Ubr3	-0.76	1.36E-05	3.47E-05
4011	ENSMUSG00000022947	Cbr3	1.07	1.36E-05	3.47E-05
4012	ENSMUSG00000020721	Helz	-0.84	1.36E-05	3.47E-05
4013	ENSMUSG00000020315	Sptbn1	-0.64	1.36E-05	3.47E-05
4014	ENSMUSG00000024104	Washc2	-0.70	1.36E-05	3.47E-05
4015	ENSMUSG00000086347	Gm11626	-0.92	1.37E-05	3.48E-05
4016	ENSMUSG00000030725	Lipt2	0.95	1.37E-05	3.48E-05
4017	ENSMUSG00000055538	Zcchc24	-0.87	1.38E-05	3.50E-05
4018	ENSMUSG00000054720	Lrrc8c	-0.91	1.39E-05	3.54E-05
4019	ENSMUSG00000027479	Mapre1	-0.66	1.40E-05	3.55E-05
4020	ENSMUSG00000039221	Rpl22l1	0.91	1.40E-05	3.56E-05
4021	ENSMUSG00000031389	Arhgap4	0.75	1.40E-05	3.56E-05
4022	ENSMUSG00000055116	Arntl	-1.71	1.40E-05	3.56E-05
4023	ENSMUSG00000042055	Wdr11	-0.81	1.41E-05	3.58E-05
4024	ENSMUSG00000030096	Slc6a6	-0.73	1.42E-05	3.60E-05
4025	ENSMUSG00000026608	Kctd3	-0.76	1.43E-05	3.62E-05
4026	ENSMUSG00000033793	Atp6v1h	0.66	1.43E-05	3.63E-05
4027	ENSMUSG00000036158	Prickle1	-0.95	1.43E-05	3.63E-05
4028	ENSMUSG00000035093	Secisbp2l	-0.70	1.44E-05	3.65E-05
4029	ENSMUSG00000026662	Sephs1	-0.81	1.44E-05	3.65E-05
4030	ENSMUSG00000037296	Lsm1	0.76	1.44E-05	3.65E-05
4031	ENSMUSG00000028843	Sh3bgrl3	-0.85	1.44E-05	3.66E-05
4032	ENSMUSG00000097174	Gm4890	1.18	1.45E-05	3.67E-05
4033	ENSMUSG00000030726	Pold3	-1.04	1.45E-05	3.67E-05
4034	ENSMUSG00000024135	Srbd1	-0.99	1.45E-05	3.67E-05
4035	ENSMUSG00000051339	2900026A02Rik	-1.13	1.46E-05	3.69E-05
4036	ENSMUSG00000057236	Rbbp4	-0.75	1.47E-05	3.71E-05
4037	ENSMUSG00000042363	Lgalsl	-0.85	1.47E-05	3.72E-05
4038	ENSMUSG00000030042	Pole4	0.67	1.47E-05	3.73E-05
4039	ENSMUSG00000111856	AC163623.3	-0.64	1.47E-05	3.73E-05
4040	ENSMUSG00000030057	Cnbp	0.64	1.48E-05	3.74E-05
4041	ENSMUSG00000012535	Tnpo3	-0.70	1.48E-05	3.76E-05
4042	ENSMUSG00000020308	Tpgs1	0.70	1.49E-05	3.77E-05
4043	ENSMUSG00000024457	Trim26	-0.76	1.50E-05	3.80E-05
4044	ENSMUSG00000026401	Cd55b	-1.11	1.50E-05	3.80E-05
4045	ENSMUSG00000002980	Bcam	-1.05	1.51E-05	3.81E-05
4046	ENSMUSG00000038280	Ostm1	-0.83	1.51E-05	3.82E-05
4047	ENSMUSG00000089824	Rbm12	-0.85	1.52E-05	3.84E-05

4048	ENSMUSG00000027668	Mfn1	0.68	1.53E-05	3.87E-05
4049	ENSMUSG00000025786	Zdhhc3	-0.67	1.53E-05	3.87E-05
4050	ENSMUSG00000019943	Atp2b1	-0.87	1.54E-05	3.90E-05
4051	ENSMUSG00000021254	Gpatch2l	-1.13	1.55E-05	3.90E-05
4052	ENSMUSG00000021884	Hacl1	0.62	1.55E-05	3.90E-05
4053	ENSMUSG00000046675	Tmem251	0.93	1.55E-05	3.92E-05
4054	ENSMUSG00000026883	Dab2ip	-0.92	1.57E-05	3.95E-05
4055	ENSMUSG00000018001	Cyth3	-0.98	1.57E-05	3.96E-05
4056	ENSMUSG00000044465	Fam160a2	-0.68	1.58E-05	3.99E-05
4057	ENSMUSG00000031993	Snx19	-0.98	1.60E-05	4.03E-05
4058	ENSMUSG00000075701	Selenos	0.72	1.60E-05	4.04E-05
4059	ENSMUSG00000028134	Ptbp2	1.08	1.61E-05	4.05E-05
4060	ENSMUSG00000028035	Dnajb4	-0.70	1.61E-05	4.05E-05
4061	ENSMUSG00000021497	Txndc15	0.68	1.61E-05	4.05E-05
4062	ENSMUSG00000028101	Pias3	-1.05	1.64E-05	4.12E-05
4063	ENSMUSG00000026510	Trp53bp2	-1.08	1.65E-05	4.15E-05
4064	ENSMUSG00000024180	Tmem8	1.00	1.65E-05	4.16E-05
4065	ENSMUSG00000038623	Tm6sf1	1.14	1.66E-05	4.16E-05
4066	ENSMUSG00000010538	Tsacc	1.27	1.66E-05	4.17E-05
4067	ENSMUSG00000025702	March8	-0.77	1.66E-05	4.18E-05
4068	ENSMUSG00000031563	Wwc2	-0.73	1.67E-05	4.19E-05
4069	ENSMUSG00000033769	Exoc6b	-0.99	1.68E-05	4.21E-05
4070	ENSMUSG00000037049	Smpd1	0.63	1.68E-05	4.21E-05
4071	ENSMUSG00000046229	Scand1	0.76	1.68E-05	4.23E-05
4072	ENSMUSG00000032942	Ucp3	0.71	1.69E-05	4.24E-05
4073	ENSMUSG00000031583	Wrn	-1.09	1.69E-05	4.25E-05
4074	ENSMUSG00000071176	Arhgef10	-1.05	1.70E-05	4.28E-05
4075	ENSMUSG00000098950	Gm28036	-0.63	1.71E-05	4.30E-05
4076	ENSMUSG00000092243	Gm7030	-1.13	1.72E-05	4.32E-05
4077	ENSMUSG00000028909	Ptpru	-1.17	1.73E-05	4.33E-05
4078	ENSMUSG00000022827	Rabl3	0.90	1.74E-05	4.35E-05
4079	ENSMUSG00000000440	Pparg	-0.65	1.74E-05	4.36E-05
4080	ENSMUSG00000023460	Rab12	0.71	1.75E-05	4.37E-05
4081	ENSMUSG00000020962	Gtf2a1	-0.82	1.75E-05	4.38E-05
4082	ENSMUSG00000020472	Zkscan17	-1.10	1.75E-05	4.38E-05
4083	ENSMUSG00000028920	Fbxo42	-0.81	1.75E-05	4.38E-05
4084	ENSMUSG00000086587	Gm11837	0.93	1.76E-05	4.40E-05
4085	ENSMUSG00000048355	Arxes1	-0.78	1.76E-05	4.41E-05
4086	ENSMUSG00000022442	Ttll1	-1.00	1.76E-05	4.41E-05
4087	ENSMUSG00000068328	Aup1	0.62	1.76E-05	4.41E-05
4088	ENSMUSG00000052539	Magi3	-1.13	1.76E-05	4.41E-05
4089	ENSMUSG00000034832	Tet3	-1.01	1.77E-05	4.42E-05
4090	ENSMUSG00000034893	Cog3	-0.88	1.77E-05	4.43E-05
4091	ENSMUSG00000026341	Actr3	0.63	1.78E-05	4.44E-05
4092	ENSMUSG00000030433	Sbk2	1.08	1.79E-05	4.47E-05
4093	ENSMUSG00000056999	Ide	0.66	1.79E-05	4.48E-05
4094	ENSMUSG00000014349	Ube2z	-0.78	1.80E-05	4.51E-05
4095	ENSMUSG00000036893	Ehmt1	-0.89	1.81E-05	4.52E-05
4096	ENSMUSG00000035314	Gdpd5	-0.93	1.81E-05	4.52E-05
4097	ENSMUSG00000078920	Ifi47	-1.02	1.81E-05	4.53E-05

4098	ENSMUSG00000111329	AC122273.1	-0.88	1.82E-05	4.53E-05
4099	ENSMUSG00000035969	Rusc2	-0.81	1.82E-05	4.53E-05
4100	ENSMUSG00000086392	Mccc1os	-0.84	1.82E-05	4.54E-05
4101	ENSMUSG00000051375	Pcdh1	-1.02	1.82E-05	4.54E-05
4102	ENSMUSG00000021754	Map3k1	-1.02	1.82E-05	4.54E-05
4103	ENSMUSG00000019470	Xab2	-0.78	1.82E-05	4.54E-05
4104	ENSMUSG00000028698	Pik3r3	-1.04	1.83E-05	4.56E-05
4105	ENSMUSG00000055302	Mrfap1	0.62	1.83E-05	4.57E-05
4106	ENSMUSG00000074916	Chst14	-1.08	1.84E-05	4.58E-05
4107	ENSMUSG00000032640	Chsy1	-1.00	1.84E-05	4.59E-05
4108	ENSMUSG00000015843	Rxrg	0.70	1.85E-05	4.60E-05
4109	ENSMUSG00000027562	Car2	1.08	1.85E-05	4.60E-05
4110	ENSMUSG00000003680	Taf6l	0.66	1.87E-05	4.65E-05
4111	ENSMUSG00000033488	BC026585	-0.68	1.87E-05	4.66E-05
4112	ENSMUSG00000036503	Rnf13	0.64	1.87E-05	4.66E-05
4113	ENSMUSG00000020154	Ptprb	-0.84	1.87E-05	4.66E-05
4114	ENSMUSG00000028645	Slc2a1	-1.12	1.88E-05	4.68E-05
4115	ENSMUSG00000028654	Mycl	-1.86	1.89E-05	4.70E-05
4116	ENSMUSG00000073402	Gm8909	-1.12	1.90E-05	4.72E-05
4117	ENSMUSG00000002221	Paxip1	-0.87	1.92E-05	4.76E-05
4118	ENSMUSG00000030101	Sumf1	-0.67	1.92E-05	4.77E-05
4119	ENSMUSG00000035047	Kri1	0.86	1.92E-05	4.77E-05
4120	ENSMUSG00000029247	Paics	0.65	1.93E-05	4.78E-05
4121	ENSMUSG00000025393	Atp5b	0.73	1.94E-05	4.81E-05
4122	ENSMUSG00000074305	Peak1	-1.01	1.94E-05	4.81E-05
4123	ENSMUSG00000001964	Emd	1.06	1.96E-05	4.87E-05
4124	ENSMUSG00000066621	Tecpr1	0.67	1.96E-05	4.87E-05
4125	ENSMUSG00000028337	Coro2a	-1.08	1.97E-05	4.87E-05
4126	ENSMUSG00000022377	Asap1	-0.85	1.97E-05	4.89E-05
4127	ENSMUSG00000028708	Mknk1	-0.85	1.99E-05	4.93E-05
4128	ENSMUSG00000040712	Camta2	-0.76	1.99E-05	4.93E-05
4129	ENSMUSG00000006281	Tep1	-0.72	1.99E-05	4.93E-05
4130	ENSMUSG00000097770	Gm26776	-0.65	1.99E-05	4.93E-05
4131	ENSMUSG00000022750	Klhl22	-0.64	1.99E-05	4.93E-05
4132	ENSMUSG00000039795	Zfand1	0.82	2.00E-05	4.94E-05
4133	ENSMUSG00000042772	Smg7	-0.79	2.01E-05	4.97E-05
4134	ENSMUSG00000045136	Tubb2b	-1.11	2.01E-05	4.98E-05
4135	ENSMUSG00000062604	Srpk2	-0.78	2.04E-05	5.03E-05
4136	ENSMUSG00000022676	Snai2	-1.09	2.04E-05	5.04E-05
4137	ENSMUSG00000030967	Zranb1	-0.87	2.05E-05	5.06E-05
4138	ENSMUSG00000036676	Tmtc3	-1.12	2.06E-05	5.08E-05
4139	ENSMUSG00000023286	Ube2j2	0.68	2.06E-05	5.09E-05
4140	ENSMUSG00000040843	Tiprl	0.70	2.07E-05	5.11E-05
4141	ENSMUSG00000079427	Mthfsl	0.80	2.07E-05	5.11E-05
4142	ENSMUSG00000002718	Cse1l	-0.70	2.07E-05	5.12E-05
4143	ENSMUSG00000031568	Rwdd4a	0.80	2.10E-05	5.17E-05
4144	ENSMUSG00000026457	Adipor1	0.63	2.11E-05	5.20E-05
4145	ENSMUSG00000035232	Pdk3	-1.06	2.13E-05	5.26E-05
4146	ENSMUSG00000020526	Znhit3	0.81	2.14E-05	5.28E-05
4147	ENSMUSG00000024900	Cpt1a	-0.81	2.14E-05	5.28E-05

4148	ENSMUSG00000030827	Fgf21	1.03	2.14E-05	5.29E-05
4149	ENSMUSG00000053334	Ficd	-0.88	2.16E-05	5.33E-05
4150	ENSMUSG00000005514	Por	-0.76	2.20E-05	5.42E-05
4151	ENSMUSG00000036473	Tbc1d24	-0.88	2.20E-05	5.42E-05
4152	ENSMUSG00000029093	Sorcs2	1.16	2.22E-05	5.47E-05
4153	ENSMUSG00000028152	Tspan5	-0.99	2.22E-05	5.47E-05
4154	ENSMUSG00000069769	Msi2	1.14	2.23E-05	5.50E-05
4155	ENSMUSG00000026279	Thap4	0.80	2.23E-05	5.50E-05
4156	ENSMUSG00000008035	Mid1ip1	-1.04	2.25E-05	5.53E-05
4157	ENSMUSG00000021371	Mcur1	0.60	2.25E-05	5.53E-05
4158	ENSMUSG00000001661	Hoxc6	-0.87	2.26E-05	5.57E-05
4159	ENSMUSG00000039753	Fbxl5	-0.75	2.27E-05	5.58E-05
4160	ENSMUSG00000085151	1110018N20Rik	1.02	2.27E-05	5.59E-05
4161	ENSMUSG00000105096	Gbp10	-0.89	2.28E-05	5.60E-05
4162	ENSMUSG00000060002	Chpt1	0.68	2.28E-05	5.61E-05
4163	ENSMUSG00000048040	Arxes2	-0.83	2.29E-05	5.63E-05
4164	ENSMUSG00000024968	Rcor2	-1.00	2.32E-05	5.70E-05
4165	ENSMUSG00000021048	Mthfd1	-0.67	2.32E-05	5.71E-05
4166	ENSMUSG00000022533	Atp13a3	-0.73	2.35E-05	5.76E-05
4167	ENSMUSG00000092607	Scnm1	0.92	2.35E-05	5.77E-05
4168	ENSMUSG00000101225	1700008J07Rik	0.91	2.37E-05	5.81E-05
4169	ENSMUSG00000075590	Nrbp2	-0.78	2.37E-05	5.82E-05
4170	ENSMUSG00000054426	A930005H10Rik	0.99	2.38E-05	5.83E-05
4171	ENSMUSG00000035297	Cops4	0.64	2.38E-05	5.84E-05
4172	ENSMUSG00000078862	Gm14326	0.77	2.38E-05	5.84E-05
4173	ENSMUSG00000000168	Dlat	0.71	2.38E-05	5.84E-05
4174	ENSMUSG00000024413	Npc1	0.79	2.39E-05	5.85E-05
4175	ENSMUSG00000000317	Bcl6b	-1.06	2.42E-05	5.91E-05
4176	ENSMUSG00000046562	Unc119b	0.71	2.42E-05	5.91E-05
4177	ENSMUSG00000027961	Lrrc39	0.76	2.42E-05	5.92E-05
4178	ENSMUSG00000066306	Numa1	-0.68	2.42E-05	5.92E-05
4179	ENSMUSG00000105383	Gm42641	-0.74	2.42E-05	5.92E-05
4180	ENSMUSG00000035941	Ibtk	-0.91	2.44E-05	5.97E-05
4181	ENSMUSG00000028911	Srsf4	-0.78	2.44E-05	5.98E-05
4182	ENSMUSG00000063480	Snu13	0.63	2.45E-05	5.99E-05
4183	ENSMUSG00000038607	Gng10	0.77	2.46E-05	6.01E-05
4184	ENSMUSG00000058886	Deaf1	-0.79	2.46E-05	6.02E-05
4185	ENSMUSG00000035493	Tgfbi	-1.28	2.48E-05	6.06E-05
4186	ENSMUSG00000035173	Ccdc186	-0.95	2.49E-05	6.07E-05
4187	ENSMUSG00000049091	Sephs2	0.62	2.50E-05	6.11E-05
4188	ENSMUSG00000029178	Klf3	-0.71	2.51E-05	6.12E-05
4189	ENSMUSG00000000530	Acvrl1	-1.03	2.52E-05	6.14E-05
4190	ENSMUSG00000044442	N6amt1	0.75	2.52E-05	6.16E-05
4191	ENSMUSG00000039585	Myo9a	-1.01	2.53E-05	6.16E-05
4192	ENSMUSG00000027411	Vps16	-0.74	2.55E-05	6.23E-05
4193	ENSMUSG00000030452	Nipa2	0.71	2.56E-05	6.23E-05
4194	ENSMUSG00000028894	Inpp5b	-0.87	2.56E-05	6.24E-05
4195	ENSMUSG00000045103	Dmd	-1.07	2.58E-05	6.28E-05
4196	ENSMUSG00000022957	Itsn1	-0.83	2.59E-05	6.30E-05
4197	ENSMUSG00000091705	H2-Q2	-1.07	2.59E-05	6.31E-05

4198	ENSMUSG00000041845	Rhod	-0.92	2.59E-05	6.32E-05
4199	ENSMUSG00000029635	Cdk8	0.87	2.61E-05	6.36E-05
4200	ENSMUSG00000031723	Txnl4b	0.82	2.62E-05	6.37E-05
4201	ENSMUSG00000042675	Ypel3	0.74	2.62E-05	6.38E-05
4202	ENSMUSG00000027751	Supt20	-1.00	2.62E-05	6.39E-05
4203	ENSMUSG00000020628	Trappc12	-0.74	2.63E-05	6.40E-05
4204	ENSMUSG00000028029	Aimp1	0.85	2.63E-05	6.40E-05
4205	ENSMUSG00000111128	AC126804.2	-0.93	2.64E-05	6.41E-05
4206	ENSMUSG00000039763	Dnajc28	0.79	2.64E-05	6.42E-05
4207	ENSMUSG00000028651	Ppie	0.88	2.66E-05	6.47E-05
4208	ENSMUSG00000032244	Fem1b	-0.83	2.67E-05	6.49E-05
4209	ENSMUSG00000024370	Cdc23	-0.92	2.68E-05	6.50E-05
4210	ENSMUSG00000015790	Surf1	0.67	2.69E-05	6.54E-05
4211	ENSMUSG00000020486	Sep-04	-1.08	2.69E-05	6.54E-05
4212	ENSMUSG00000021911	Parg	-0.79	2.71E-05	6.57E-05
4213	ENSMUSG00000033075	Senp1	-0.89	2.71E-05	6.57E-05
4214	ENSMUSG00000078630	Tomt	0.75	2.71E-05	6.57E-05
4215	ENSMUSG00000037286	Stag1	-0.93	2.71E-05	6.57E-05
4216	ENSMUSG00000048232	Fbxo10	-1.09	2.72E-05	6.59E-05
4217	ENSMUSG00000103030	E330011M16Rik	0.93	2.72E-05	6.59E-05
4218	ENSMUSG00000026200	Glb1l	-0.99	2.72E-05	6.59E-05
4219	ENSMUSG00000018906	P4ha2	-0.84	2.74E-05	6.64E-05
4220	ENSMUSG00000043998	Mgat2	-0.73	2.75E-05	6.65E-05
4221	ENSMUSG00000023068	Nus1	0.66	2.75E-05	6.66E-05
4222	ENSMUSG00000026988	Wdsub1	-0.82	2.76E-05	6.67E-05
4223	ENSMUSG00000032652	Crebl2	-1.04	2.76E-05	6.68E-05
4224	ENSMUSG00000030120	Mlf2	0.62	2.76E-05	6.69E-05
4225	ENSMUSG00000010067	Rassf1	-0.87	2.77E-05	6.70E-05
4226	ENSMUSG00000061882	Ccdc62	0.94	2.79E-05	6.76E-05
4227	ENSMUSG00000078867	Gm14418	1.00	2.80E-05	6.76E-05
4228	ENSMUSG00000101856	1700096K18Rik	0.86	2.80E-05	6.78E-05
4229	ENSMUSG00000029554	Mad1l1	-0.94	2.81E-05	6.79E-05
4230	ENSMUSG00000020922	Lsm12	0.65	2.81E-05	6.80E-05
4231	ENSMUSG00000017670	Elmo2	-0.77	2.83E-05	6.83E-05
4232	ENSMUSG00000002778	Kdelr1	0.70	2.85E-05	6.88E-05
4233	ENSMUSG00000021131	Erh	0.74	2.86E-05	6.91E-05
4234	ENSMUSG00000024381	Bin1	-0.81	2.87E-05	6.92E-05
4235	ENSMUSG00000030960	Mettl10	0.93	2.87E-05	6.94E-05
4236	ENSMUSG00000025188	Hps1	-0.90	2.90E-05	7.00E-05
4237	ENSMUSG00000040327	Cul9	-0.85	2.90E-05	7.00E-05
4238	ENSMUSG00000025474	Tubgcp2	-0.90	2.90E-05	7.00E-05
4239	ENSMUSG00000028599	Tnfrsf1b	-1.27	2.91E-05	7.01E-05
4240	ENSMUSG00000025572	Tmc6	0.83	2.91E-05	7.02E-05
4241	ENSMUSG00000028958	Tmub1	0.92	2.92E-05	7.04E-05
4242	ENSMUSG00000026078	Pdcl3	0.74	2.96E-05	7.13E-05
4243	ENSMUSG00000000148	Brat1	-0.79	2.98E-05	7.18E-05
4244	ENSMUSG00000074771	Ankef1	-0.76	2.98E-05	7.19E-05
4245	ENSMUSG00000042626	Shc1	-0.61	2.99E-05	7.19E-05
4246	ENSMUSG00000004849	Ap1s1	0.65	2.99E-05	7.20E-05
4247	ENSMUSG00000054855	Rnd1	-1.04	2.99E-05	7.20E-05

4248	ENSMUSG00000001348	Acp5	-0.62	2.99E-05	7.21E-05
4249	ENSMUSG00000027963	Extl2	-0.96	3.01E-05	7.25E-05
4250	ENSMUSG00000024219	Anks1	-0.89	3.01E-05	7.25E-05
4251	ENSMUSG00000028397	Kdm4c	-0.94	3.01E-05	7.25E-05
4252	ENSMUSG00000034297	Med13	-0.85	3.03E-05	7.30E-05
4253	ENSMUSG00000039069	Mtg2	0.70	3.04E-05	7.32E-05
4254	ENSMUSG00000042446	Zmym4	-0.97	3.06E-05	7.36E-05
4255	ENSMUSG00000018604	Tbx3	-1.11	3.08E-05	7.40E-05
4256	ENSMUSG00000024959	Bad	0.69	3.10E-05	7.45E-05
4257	ENSMUSG00000020687	Cdc27	-0.72	3.10E-05	7.45E-05
4258	ENSMUSG00000008682	Rpl10	0.65	3.11E-05	7.47E-05
4259	ENSMUSG00000064138	Fam172a	-0.94	3.12E-05	7.48E-05
4260	ENSMUSG00000028447	Dctn3	0.67	3.12E-05	7.48E-05
4261	ENSMUSG00000022253	Nadk2	0.66	3.13E-05	7.52E-05
4262	ENSMUSG00000034165	Ccnd3	-0.64	3.15E-05	7.56E-05
4263	ENSMUSG00000020572	Nampt	0.62	3.16E-05	7.57E-05
4264	ENSMUSG00000062515	Fabp4	0.74	3.17E-05	7.60E-05
4265	ENSMUSG00000031479	Vps36	0.65	3.19E-05	7.65E-05
4266	ENSMUSG00000025993	Slc40a1	1.02	3.20E-05	7.67E-05
4267	ENSMUSG00000038806	Sde2	-0.78	3.22E-05	7.72E-05
4268	ENSMUSG00000040760	Appl1	-0.90	3.23E-05	7.74E-05
4269	ENSMUSG00000015668	Pdzd11	0.72	3.23E-05	7.74E-05
4270	ENSMUSG00000022125	Cln5	0.68	3.23E-05	7.74E-05
4271	ENSMUSG00000061455	Stx17	-0.73	3.24E-05	7.76E-05
4272	ENSMUSG00000029614	Rpl6	0.72	3.24E-05	7.76E-05
4273	ENSMUSG00000040102	Klhl42	-0.91	3.24E-05	7.76E-05
4274	ENSMUSG00000074922	Fam122a	0.70	3.25E-05	7.79E-05
4275	ENSMUSG00000024811	Tnks2	-0.75	3.26E-05	7.79E-05
4276	ENSMUSG00000028864	Hgf	-1.09	3.27E-05	7.82E-05
4277	ENSMUSG00000034636	Zyg11b	-0.76	3.27E-05	7.82E-05
4278	ENSMUSG00000073639	Rab18	0.62	3.28E-05	7.83E-05
4279	ENSMUSG00000069303	Hist1h2br	0.96	3.28E-05	7.84E-05
4280	ENSMUSG00000068732	Tmem167b	-0.69	3.29E-05	7.86E-05
4281	ENSMUSG00000036197	Gxylt1	-0.94	3.30E-05	7.88E-05
4282	ENSMUSG00000025584	Pde8a	0.64	3.31E-05	7.90E-05
4283	ENSMUSG00000022305	Lrp12	-1.02	3.31E-05	7.90E-05
4284	ENSMUSG00000087291	Gm11946	-1.11	3.31E-05	7.91E-05
4285	ENSMUSG00000022838	Eaf2	1.05	3.31E-05	7.91E-05
4286	ENSMUSG00000031586	Rbpms	0.65	3.32E-05	7.91E-05
4287	ENSMUSG00000032038	St3gal4	-1.21	3.32E-05	7.92E-05
4288	ENSMUSG00000024251	Thada	-1.01	3.34E-05	7.96E-05
4289	ENSMUSG00000033323	Ctdp1	-0.85	3.34E-05	7.97E-05
4290	ENSMUSG00000056267	Cep70	-0.86	3.34E-05	7.97E-05
4291	ENSMUSG00000060121	Gemin2	0.94	3.35E-05	7.99E-05
4292	ENSMUSG00000034940	Synrg	-0.76	3.38E-05	8.05E-05
4293	ENSMUSG00000043923	Ccdc84	0.95	3.38E-05	8.05E-05
4294	ENSMUSG00000009079	Ewsr1	-0.60	3.38E-05	8.05E-05
4295	ENSMUSG00000031590	Frg1	0.80	3.39E-05	8.07E-05
4296	ENSMUSG00000053931	Cnn3	-0.93	3.41E-05	8.11E-05
4297	ENSMUSG00000039699	Batf2	0.82	3.41E-05	8.12E-05

4298	ENSMUSG00000022236	Ropn1l	0.94	3.42E-05	8.15E-05
4299	ENSMUSG00000028975	Pex14	0.59	3.43E-05	8.16E-05
4300	ENSMUSG00000029710	Ephb4	-0.89	3.44E-05	8.17E-05
4301	ENSMUSG00000022672	Prkdc	-0.96	3.44E-05	8.17E-05
4302	ENSMUSG00000038611	Phrf1	-0.83	3.47E-05	8.24E-05
4303	ENSMUSG00000043488	Frmd8os	1.05	3.48E-05	8.28E-05
4304	ENSMUSG00000064127	Med14	-0.75	3.49E-05	8.28E-05
4305	ENSMUSG00000031347	Cetn2	0.86	3.49E-05	8.28E-05
4306	ENSMUSG00000036990	Otud4	-0.78	3.49E-05	8.29E-05
4307	ENSMUSG00000030059	Tmf1	0.85	3.50E-05	8.31E-05
4308	ENSMUSG00000021177	Tdp1	-0.99	3.51E-05	8.32E-05
4309	ENSMUSG00000108037	Gm44597	0.67	3.51E-05	8.32E-05
4310	ENSMUSG00000026201	Stk16	0.64	3.51E-05	8.33E-05
4311	ENSMUSG00000029449	Rhof	-0.78	3.51E-05	8.33E-05
4312	ENSMUSG00000026209	Dnpep	0.61	3.52E-05	8.34E-05
4313	ENSMUSG00000022124	Fbxl3	-0.78	3.52E-05	8.34E-05
4314	ENSMUSG00000001418	Glmp	0.63	3.52E-05	8.35E-05
4315	ENSMUSG00000068264	Ap5s1	0.67	3.53E-05	8.36E-05
4316	ENSMUSG00000032239	Rp9	0.79	3.53E-05	8.36E-05
4317	ENSMUSG00000035109	Shc4	-0.89	3.54E-05	8.39E-05
4318	ENSMUSG00000005836	Gata6	-1.11	3.54E-05	8.39E-05
4319	ENSMUSG00000075415	Fnbp1	-0.73	3.55E-05	8.40E-05
4320	ENSMUSG00000022641	Bbx	-0.90	3.58E-05	8.48E-05
4321	ENSMUSG00000097414	B130046B21Rik	0.60	3.59E-05	8.49E-05
4322	ENSMUSG00000022983	Scaf4	-0.86	3.60E-05	8.51E-05
4323	ENSMUSG00000025223	Ldb1	-0.81	3.60E-05	8.53E-05
4324	ENSMUSG00000021643	Serf1	1.07	3.61E-05	8.53E-05
4325	ENSMUSG00000035027	Map2k2	0.64	3.62E-05	8.55E-05
4326	ENSMUSG00000013787	Ehmt2	-0.69	3.65E-05	8.62E-05
4327	ENSMUSG00000022637	Cblb	-0.92	3.66E-05	8.66E-05
4328	ENSMUSG00000026792	Lrsam1	-0.87	3.67E-05	8.66E-05
4329	ENSMUSG00000027309	4930402H24Rik	-0.62	3.68E-05	8.69E-05
4330	ENSMUSG00000006641	Slc5a6	-1.19	3.68E-05	8.70E-05
4331	ENSMUSG00000030649	Anapc15	0.73	3.69E-05	8.71E-05
4332	ENSMUSG00000027165	B230118H07Rik	0.70	3.70E-05	8.74E-05
4333	ENSMUSG00000032470	Mras	-0.94	3.72E-05	8.77E-05
4334	ENSMUSG00000019849	Prep	-0.63	3.73E-05	8.80E-05
4335	ENSMUSG00000022684	Bfar	0.61	3.73E-05	8.80E-05
4336	ENSMUSG00000037075	Rnf139	0.65	3.76E-05	8.86E-05
4337	ENSMUSG00000033032	Afap1l1	-0.99	3.78E-05	8.91E-05
4338	ENSMUSG00000040848	Sft2d2	-0.65	3.78E-05	8.92E-05
4339	ENSMUSG00000026814	Eng	-0.77	3.81E-05	8.97E-05
4340	ENSMUSG00000025607	Copg2	-0.83	3.81E-05	8.98E-05
4341	ENSMUSG00000047205	Dusp18	-0.79	3.83E-05	9.03E-05
4342	ENSMUSG00000021890	Eaf1	-0.89	3.83E-05	9.03E-05
4343	ENSMUSG00000000594	Gm2a	-0.80	3.84E-05	9.04E-05
4344	ENSMUSG00000022508	Bcl6	-0.88	3.85E-05	9.06E-05
4345	ENSMUSG00000043535	Setx	-0.98	3.87E-05	9.12E-05
4346	ENSMUSG00000097060	Gm26759	-1.08	3.88E-05	9.14E-05
4347	ENSMUSG00000025171	Ubtd1	-0.68	3.89E-05	9.16E-05

4348	ENSMUSG00000085337	Gm15964	-0.84	3.90E-05	9.18E-05
4349	ENSMUSG00000027947	Il6ra	-1.21	3.94E-05	9.26E-05
4350	ENSMUSG00000024639	Gnaq	-0.68	3.97E-05	9.32E-05
4351	ENSMUSG00000038212	Mfsd14b	-0.73	3.98E-05	9.35E-05
4352	ENSMUSG00000079357	Gm11100	0.86	3.99E-05	9.38E-05
4353	ENSMUSG00000031904	Slc7a6	0.67	4.01E-05	9.42E-05
4354	ENSMUSG00000024960	Plcb3	-0.71	4.01E-05	9.42E-05
4355	ENSMUSG00000030403	Vasp	-1.02	4.01E-05	9.42E-05
4356	ENSMUSG00000024220	Zfp523	-0.75	4.02E-05	9.44E-05
4357	ENSMUSG00000063870	Chd4	-0.65	4.05E-05	9.50E-05
4358	ENSMUSG00000034111	Tmed8	-0.86	4.05E-05	9.50E-05
4359	ENSMUSG00000019471	Cdc37	0.61	4.05E-05	9.51E-05
4360	ENSMUSG00000033088	Triobp	-0.73	4.06E-05	9.52E-05
4361	ENSMUSG00000063160	Numbl	-1.01	4.07E-05	9.53E-05
4362	ENSMUSG00000057137	Tmem140	0.68	4.07E-05	9.54E-05
4363	ENSMUSG00000001911	Nfix	-0.64	4.08E-05	9.56E-05
4364	ENSMUSG00000047434	Xxylt1	-0.95	4.09E-05	9.59E-05
4365	ENSMUSG00000024065	Ehd3	-1.13	4.10E-05	9.60E-05
4366	ENSMUSG00000023008	Fmnl3	-1.09	4.10E-05	9.60E-05
4367	ENSMUSG00000026554	Dcaf8	-0.62	4.10E-05	9.61E-05
4368	ENSMUSG00000027185	Nat10	-0.86	4.11E-05	9.62E-05
4369	ENSMUSG00000001924	Uba1	-0.64	4.13E-05	9.66E-05
4370	ENSMUSG00000040841	Six5	-0.95	4.13E-05	9.66E-05
4371	ENSMUSG00000051184	Zfp524	0.75	4.13E-05	9.66E-05
4372	ENSMUSG00000034037	Fgd5	-0.93	4.14E-05	9.69E-05
4373	ENSMUSG00000025006	Sorbs1	-0.65	4.15E-05	9.71E-05
4374	ENSMUSG00000056121	Fez2	0.63	4.16E-05	9.72E-05
4375	ENSMUSG00000035890	Rnf126	0.69	4.17E-05	9.75E-05
4376	ENSMUSG00000046785	Epm2aip1	-0.75	4.18E-05	9.76E-05
4377	ENSMUSG00000020903	Stx8	0.81	4.20E-05	9.82E-05
4378	ENSMUSG00000032000	Birc3	-1.11	4.21E-05	9.84E-05
4379	ENSMUSG00000038175	Mylip	-0.94	4.21E-05	9.84E-05
4380	ENSMUSG00000034118	Tpst1	-1.03	4.23E-05	9.88E-05
4381	ENSMUSG00000097737	Gm26530	0.74	4.24E-05	9.91E-05
4382	ENSMUSG00000026156	B3gat2	0.77	4.27E-05	9.95E-05
4383	ENSMUSG00000024735	Prpf19	-0.68	4.27E-05	9.97E-05
4384	ENSMUSG00000021686	Ap3b1	-0.73	4.28E-05	9.98E-05
4385	ENSMUSG00000048376	F2r	0.98	4.30E-05	1.00E-04
4386	ENSMUSG00000024498	Tcerg1	-0.75	4.31E-05	1.01E-04
4387	ENSMUSG00000019461	Plscr3	-0.72	4.31E-05	1.01E-04
4388	ENSMUSG00000040006	Ginm1	0.59	4.33E-05	1.01E-04
4389	ENSMUSG00000022964	Tmem50b	0.60	4.37E-05	1.02E-04
4390	ENSMUSG00000016494	Cd34	-1.23	4.37E-05	1.02E-04
4391	ENSMUSG00000036833	Pnpla7	-0.86	4.37E-05	1.02E-04
4392	ENSMUSG00000005204	Senp3	0.69	4.38E-05	1.02E-04
4393	ENSMUSG00000040688	Tbl3	-0.63	4.41E-05	1.03E-04
4394	ENSMUSG00000033965	Slc16a2	0.84	4.43E-05	1.03E-04
4395	ENSMUSG00000042535	Gtpbp1	-0.77	4.43E-05	1.03E-04
4396	ENSMUSG00000030330	Ing4	0.66	4.43E-05	1.03E-04
4397	ENSMUSG00000070737	Tmem35b	0.75	4.44E-05	1.03E-04

4398	ENSMUSG00000039285	Azi2	0.63	4.46E-05	1.04E-04
4399	ENSMUSG00000034574	Daam1	-1.09	4.50E-05	1.05E-04
4400	ENSMUSG00000056687	Gm11696	0.93	4.50E-05	1.05E-04
4401	ENSMUSG00000028709	Mob3c	-1.02	4.50E-05	1.05E-04
4402	ENSMUSG00000019718	L3hypdh	0.65	4.53E-05	1.05E-04
4403	ENSMUSG00000036534	Slc38a7	-0.84	4.54E-05	1.05E-04
4404	ENSMUSG00000108806	Gm44729	0.59	4.54E-05	1.05E-04
4405	ENSMUSG00000067713	Prkag1	0.59	4.54E-05	1.05E-04
4406	ENSMUSG00000030630	Fah	0.58	4.55E-05	1.06E-04
4407	ENSMUSG00000020592	Sdc1	-1.07	4.58E-05	1.06E-04
4408	ENSMUSG00000068039	Tcp1	0.61	4.59E-05	1.06E-04
4409	ENSMUSG00000061536	Sec22c	-1.11	4.60E-05	1.07E-04
4410	ENSMUSG00000027257	Pacsin3	-0.82	4.60E-05	1.07E-04
4411	ENSMUSG00000027901	Dennd2d	-0.91	4.62E-05	1.07E-04
4412	ENSMUSG00000039367	Sec24c	-0.70	4.62E-05	1.07E-04
4413	ENSMUSG00000027746	Ufm1	0.59	4.66E-05	1.08E-04
4414	ENSMUSG00000034795	Ccdc122	0.81	4.68E-05	1.09E-04
4415	ENSMUSG00000022235	Cmbl	-0.59	4.69E-05	1.09E-04
4416	ENSMUSG00000092124	B930094E09Rik	0.72	4.74E-05	1.10E-04
4417	ENSMUSG00000028889	Yrdc	0.73	4.74E-05	1.10E-04
4418	ENSMUSG00000043411	Usp48	-0.72	4.75E-05	1.10E-04
4419	ENSMUSG00000025245	Lztfl1	0.91	4.75E-05	1.10E-04
4420	ENSMUSG00000001962	Fam50a	0.69	4.76E-05	1.10E-04
4421	ENSMUSG00000074272	Ceacam1	-0.98	4.78E-05	1.11E-04
4422	ENSMUSG00000074203	G430095P16Rik	-0.72	4.78E-05	1.11E-04
4423	ENSMUSG00000048445	Ccdc57	0.91	4.81E-05	1.11E-04
4424	ENSMUSG00000022008	Gpalpp1	-0.92	4.81E-05	1.11E-04
4425	ENSMUSG00000025040	Fundc1	0.65	4.83E-05	1.12E-04
4426	ENSMUSG00000028936	Rpl22	0.84	4.83E-05	1.12E-04
4427	ENSMUSG00000097350	4732491K20Rik	1.20	4.87E-05	1.12E-04
4428	ENSMUSG00000029538	Srsf9	0.62	4.91E-05	1.13E-04
4429	ENSMUSG00000037415	Ranbp10	-0.72	4.91E-05	1.13E-04
4430	ENSMUSG00000034926	Dhcr24	1.00	4.91E-05	1.13E-04
4431	ENSMUSG00000079559	Gm684	0.91	4.92E-05	1.14E-04
4432	ENSMUSG00000030541	Idh2	0.67	4.93E-05	1.14E-04
4433	ENSMUSG00000041355	Ssr2	0.63	4.93E-05	1.14E-04
4434	ENSMUSG00000028081	Rps3a1	0.66	4.94E-05	1.14E-04
4435	ENSMUSG00000039208	Metrnl	0.90	4.96E-05	1.14E-04
4436	ENSMUSG00000064302	Clasp1	-0.78	4.98E-05	1.15E-04
4437	ENSMUSG00000034471	Caskin2	-0.90	5.00E-05	1.15E-04
4438	ENSMUSG00000013997	Nit1	0.59	5.02E-05	1.16E-04
4439	ENSMUSG00000022811	Zfp148	-0.72	5.02E-05	1.16E-04
4440	ENSMUSG00000028822	Tmem50a	0.61	5.03E-05	1.16E-04
4441	ENSMUSG00000030341	Tnfrsf1a	-0.56	5.03E-05	1.16E-04
4442	ENSMUSG00000026249	Serpine2	-0.70	5.06E-05	1.16E-04
4443	ENSMUSG00000033308	Dpyd	-0.87	5.08E-05	1.17E-04
4444	ENSMUSG00000005950	P2rx5	0.81	5.08E-05	1.17E-04
4445	ENSMUSG00000051502	Ufsp1	0.85	5.09E-05	1.17E-04
4446	ENSMUSG00000020826	Nos2	-1.15	5.09E-05	1.17E-04
4447	ENSMUSG00000042632	Pla2g6	-0.67	5.10E-05	1.17E-04

4448	ENSMUSG00000030781	Slc5a2	-0.83	5.11E-05	1.18E-04
4449	ENSMUSG00000049969	Plekhf2	0.67	5.12E-05	1.18E-04
4450	ENSMUSG00000005483	Dnajb1	-0.88	5.13E-05	1.18E-04
4451	ENSMUSG00000036555	Iqce	-1.05	5.16E-05	1.19E-04
4452	ENSMUSG00000068921	Dap3	0.64	5.16E-05	1.19E-04
4453	ENSMUSG00000106427	Gm42820	-0.98	5.16E-05	1.19E-04
4454	ENSMUSG00000026571	Dcaf6	0.62	5.19E-05	1.19E-04
4455	ENSMUSG00000108888	Gm45231	0.90	5.22E-05	1.20E-04
4456	ENSMUSG00000020919	Stat5b	-0.65	5.24E-05	1.20E-04
4457	ENSMUSG00000108112	Gm45193	-1.22	5.25E-05	1.20E-04
4458	ENSMUSG00000031256	Cstf2	-0.82	5.26E-05	1.21E-04
4459	ENSMUSG00000047182	Irs3	-0.63	5.27E-05	1.21E-04
4460	ENSMUSG00000031446	Cul4a	-0.73	5.32E-05	1.22E-04
4461	ENSMUSG00000030256	Bhlhe41	0.95	5.33E-05	1.22E-04
4462	ENSMUSG00000047459	Dynlrb1	0.58	5.33E-05	1.22E-04
4463	ENSMUSG00000025939	Ube2w	0.60	5.36E-05	1.23E-04
4464	ENSMUSG00000032481	Smarcc1	-0.84	5.39E-05	1.24E-04
4465	ENSMUSG00000068220	Lgals1	0.64	5.42E-05	1.24E-04
4466	ENSMUSG00000038141	Tmem181a	-1.01	5.42E-05	1.24E-04
4467	ENSMUSG00000041607	Mbp	-0.65	5.43E-05	1.24E-04
4468	ENSMUSG00000042616	Oscp1	-1.09	5.44E-05	1.25E-04
4469	ENSMUSG00000004360	9330159F19Rik	-1.34	5.45E-05	1.25E-04
4470	ENSMUSG00000045690	Wdr89	0.90	5.47E-05	1.25E-04
4471	ENSMUSG00000020263	Appl2	-0.86	5.53E-05	1.26E-04
4472	ENSMUSG00000063856	Gpx1	0.62	5.55E-05	1.27E-04
4473	ENSMUSG00000092595	Gm20427	0.60	5.55E-05	1.27E-04
4474	ENSMUSG00000092341	Malat1	0.73	5.57E-05	1.27E-04
4475	ENSMUSG00000030123	Plxnd1	-0.82	5.57E-05	1.27E-04
4476	ENSMUSG00000026932	Nacc2	-0.56	5.58E-05	1.28E-04
4477	ENSMUSG00000097509	B230322F03Rik	0.81	5.60E-05	1.28E-04
4478	ENSMUSG00000028861	Mrps15	0.66	5.60E-05	1.28E-04
4479	ENSMUSG00000095990	Zfp97	-0.94	5.64E-05	1.29E-04
4480	ENSMUSG00000029802	Abcg2	-0.90	5.65E-05	1.29E-04
4481	ENSMUSG00000029233	Srd5a3	-0.74	5.66E-05	1.29E-04
4482	ENSMUSG00000030127	Cops7a	0.59	5.67E-05	1.29E-04
4483	ENSMUSG00000026826	Nr4a2	0.93	5.67E-05	1.29E-04
4484	ENSMUSG00000031232	Magt1	0.62	5.68E-05	1.29E-04
4485	ENSMUSG00000025364	Pa2g4	0.63	5.68E-05	1.30E-04
4486	ENSMUSG00000027381	Bcl2l11	-1.01	5.68E-05	1.30E-04
4487	ENSMUSG00000028954	Nub1	-0.71	5.71E-05	1.30E-04
4488	ENSMUSG00000025968	Ndufs1	0.58	5.75E-05	1.31E-04
4489	ENSMUSG00000024299	Adamts10	-0.88	5.76E-05	1.31E-04
4490	ENSMUSG00000086327	Slfn5os	-0.99	5.78E-05	1.32E-04
4491	ENSMUSG00000029221	Slc30a9	0.59	5.78E-05	1.32E-04
4492	ENSMUSG00000005268	Prlr	-1.08	5.81E-05	1.32E-04
4493	ENSMUSG00000041444	Arhgap32	-0.93	5.83E-05	1.33E-04
4494	ENSMUSG00000018820	Zfyve27	-0.65	5.84E-05	1.33E-04
4495	ENSMUSG00000027340	Slc23a2	-0.74	5.86E-05	1.33E-04
4496	ENSMUSG00000027933	Ints3	-0.78	5.88E-05	1.34E-04
4497	ENSMUSG00000030804	Gm21974	0.64	5.94E-05	1.35E-04

4498	ENSMUSG00000047030	Spata2	-0.71	5.97E-05	1.36E-04
4499	ENSMUSG00000035392	Dennd1a	-0.80	5.97E-05	1.36E-04
4500	ENSMUSG00000035778	Ggta1	-0.90	5.98E-05	1.36E-04
4501	ENSMUSG00000029570	Lfng	-0.98	5.99E-05	1.36E-04
4502	ENSMUSG00000034761	Map4k5	-0.88	6.07E-05	1.38E-04
4503	ENSMUSG00000058638	Zfp110	-0.78	6.11E-05	1.39E-04
4504	ENSMUSG00000020392	Cdkn2aipnl	-0.76	6.12E-05	1.39E-04
4505	ENSMUSG00000047045	Tmem164	-0.91	6.16E-05	1.40E-04
4506	ENSMUSG00000069049	Eif2s3y	0.65	6.17E-05	1.40E-04
4507	ENSMUSG00000030852	Tacc2	-0.98	6.21E-05	1.41E-04
4508	ENSMUSG00000059355	Wdr83os	0.63	6.21E-05	1.41E-04
4509	ENSMUSG00000024515	Smad4	-0.66	6.22E-05	1.41E-04
4510	ENSMUSG00000097347	Gm17275	0.61	6.26E-05	1.42E-04
4511	ENSMUSG00000020149	Rab1a	0.57	6.30E-05	1.43E-04
4512	ENSMUSG00000048755	Mcat	0.58	6.31E-05	1.43E-04
4513	ENSMUSG00000053062	Jam2	-0.92	6.33E-05	1.43E-04
4514	ENSMUSG00000021893	Capn7	-0.80	6.38E-05	1.45E-04
4515	ENSMUSG00000024583	Txnl1	0.59	6.39E-05	1.45E-04
4516	ENSMUSG00000060743	H3f3a	0.60	6.39E-05	1.45E-04
4517	ENSMUSG00000096145	Vkorc1	0.64	6.39E-05	1.45E-04
4518	ENSMUSG00000018921	Pelp1	-0.92	6.41E-05	1.45E-04
4519	ENSMUSG00000053617	Sh3pxd2a	-0.86	6.42E-05	1.45E-04
4520	ENSMUSG00000099083	Atf7	-0.70	6.45E-05	1.46E-04
4521	ENSMUSG00000042613	Pbxip1	0.67	6.48E-05	1.47E-04
4522	ENSMUSG00000005225	Plekha8	0.79	6.52E-05	1.47E-04
4523	ENSMUSG00000038546	Ranbp9	-0.60	6.52E-05	1.48E-04
4524	ENSMUSG00000024168	Tmem204	-0.83	6.53E-05	1.48E-04
4525	ENSMUSG00000097722	Gm26841	0.63	6.54E-05	1.48E-04
4526	ENSMUSG00000021400	Wrnip1	-0.83	6.57E-05	1.48E-04
4527	ENSMUSG00000066233	Tmem42	0.80	6.59E-05	1.49E-04
4528	ENSMUSG00000003604	Aven	0.81	6.60E-05	1.49E-04
4529	ENSMUSG00000026483	Fam129a	0.98	6.61E-05	1.49E-04
4530	ENSMUSG00000059323	Tonsl	0.58	6.61E-05	1.49E-04
4531	ENSMUSG00000020792	Exoc7	-0.64	6.64E-05	1.50E-04
4532	ENSMUSG00000023973	Cnpy3	-0.63	6.64E-05	1.50E-04
4533	ENSMUSG00000097487	Ptges3l	0.91	6.68E-05	1.51E-04
4534	ENSMUSG00000085327	Gm16104	-0.68	6.70E-05	1.51E-04
4535	ENSMUSG00000058230	Arhgap35	-0.67	6.70E-05	1.51E-04
4536	ENSMUSG00000041762	Gpr155	-0.97	6.79E-05	1.53E-04
4537	ENSMUSG00000062075	Lmnb2	-0.99	6.82E-05	1.54E-04
4538	ENSMUSG00000042328	Hps4	-0.81	6.82E-05	1.54E-04
4539	ENSMUSG00000069300	Hist1h2bj	0.90	6.86E-05	1.55E-04
4540	ENSMUSG00000039740	Alg2	-0.79	6.89E-05	1.55E-04
4541	ENSMUSG00000053012	Krcc1	0.66	6.89E-05	1.55E-04
4542	ENSMUSG00000043079	Synpo	0.92	6.90E-05	1.55E-04
4543	ENSMUSG00000040701	Ap1g2	0.62	6.92E-05	1.56E-04
4544	ENSMUSG00000029782	Tmem209	-0.72	6.96E-05	1.57E-04
4545	ENSMUSG00000062949	Atp11c	-0.85	6.96E-05	1.57E-04
4546	ENSMUSG00000020682	Mmp28	-0.77	6.97E-05	1.57E-04
4547	ENSMUSG00000001082	Mfsd10	-0.93	6.99E-05	1.57E-04

4548	ENSMUSG00000109904	Gm45819	-0.98	7.01E-05	1.58E-04
4549	ENSMUSG00000020132	Rab21	0.64	7.02E-05	1.58E-04
4550	ENSMUSG00000029718	Pcolce	-0.89	7.03E-05	1.58E-04
4551	ENSMUSG00000042249	Grk3	0.86	7.04E-05	1.58E-04
4552	ENSMUSG00000022471	Xrcc6	0.71	7.07E-05	1.59E-04
4553	ENSMUSG00000011382	Dhdh	0.58	7.08E-05	1.59E-04
4554	ENSMUSG00000045078	Rnf216	-0.75	7.08E-05	1.59E-04
4555	ENSMUSG00000020752	Recql5	-0.97	7.09E-05	1.59E-04
4556	ENSMUSG00000110378	Gm45242	0.85	7.09E-05	1.59E-04
4557	ENSMUSG00000056476	Med12l	-0.92	7.15E-05	1.60E-04
4558	ENSMUSG00000042444	Fam63b	0.68	7.16E-05	1.61E-04
4559	ENSMUSG00000031770	Herpud1	0.68	7.16E-05	1.61E-04
4560	ENSMUSG00000006463	Zdhhc24	-0.89	7.16E-05	1.61E-04
4561	ENSMUSG00000027656	Wisp2	-1.32	7.19E-05	1.61E-04
4562	ENSMUSG00000002748	Baz1b	-0.74	7.24E-05	1.62E-04
4563	ENSMUSG00000044317	Gpr4	-0.97	7.25E-05	1.62E-04
4564	ENSMUSG00000022681	Ntan1	0.59	7.30E-05	1.64E-04
4565	ENSMUSG00000019837	Gtf3c6	0.66	7.31E-05	1.64E-04
4566	ENSMUSG00000018678	Sp2	-0.79	7.32E-05	1.64E-04
4567	ENSMUSG00000066568	Lsm14a	-0.62	7.32E-05	1.64E-04
4568	ENSMUSG00000020319	Wdpcp	-0.90	7.35E-05	1.65E-04
4569	ENSMUSG00000108378	Gm44641	-0.81	7.38E-05	1.65E-04
4570	ENSMUSG00000055371	Stam2	-0.63	7.40E-05	1.66E-04
4571	ENSMUSG00000041939	Mvk	-0.70	7.44E-05	1.66E-04
4572	ENSMUSG00000024581	Napg	-0.70	7.48E-05	1.67E-04
4573	ENSMUSG00000046768	Rhoj	-1.00	7.49E-05	1.67E-04
4574	ENSMUSG00000047242	Taf9b	-0.91	7.50E-05	1.68E-04
4575	ENSMUSG00000031930	Wwp2	-0.76	7.51E-05	1.68E-04
4576	ENSMUSG00000065954	Tacc1	-0.57	7.51E-05	1.68E-04
4577	ENSMUSG00000059588	Calcrl	-1.06	7.53E-05	1.68E-04
4578	ENSMUSG00000037622	Wdtc1	0.83	7.54E-05	1.68E-04
4579	ENSMUSG00000030681	Mvp	-0.62	7.54E-05	1.68E-04
4580	ENSMUSG00000034210	Efcab14	-0.62	7.56E-05	1.69E-04
4581	ENSMUSG00000038828	Tmem214	-0.58	7.60E-05	1.70E-04
4582	ENSMUSG00000026810	Dpm2	0.67	7.62E-05	1.70E-04
4583	ENSMUSG00000029003	Mad2l2	0.62	7.63E-05	1.70E-04
4584	ENSMUSG00000078201	Tmem203	0.76	7.69E-05	1.72E-04
4585	ENSMUSG00000025575	Cant1	-0.70	7.70E-05	1.72E-04
4586	ENSMUSG00000018861	Fdxr	0.75	7.72E-05	1.72E-04
4587	ENSMUSG00000032175	Tyk2	-0.98	7.73E-05	1.72E-04
4588	ENSMUSG00000027860	Vangl1	-0.85	7.78E-05	1.73E-04
4589	ENSMUSG00000002307	Daxx	-0.76	7.79E-05	1.74E-04
4590	ENSMUSG00000039865	Slc44a3	-0.71	7.85E-05	1.75E-04
4591	ENSMUSG00000090458	Gm17122	-0.85	7.87E-05	1.75E-04
4592	ENSMUSG00000020280	Pus10	-0.69	8.05E-05	1.79E-04
4593	ENSMUSG00000033319	Fem1c	-0.92	8.09E-05	1.80E-04
4594	ENSMUSG00000005873	Reep5	0.58	8.10E-05	1.80E-04
4595	ENSMUSG00000039523	Cep104	-0.72	8.10E-05	1.80E-04
4596	ENSMUSG00000030352	Tspan9	-1.05	8.18E-05	1.82E-04
4597	ENSMUSG00000086134	Gm16159	-1.11	8.18E-05	1.82E-04

4598	ENSMUSG00000036752	Tubb4b	0.64	8.20E-05	1.82E-04
4599	ENSMUSG00000112652	AC160411.2	-0.63	8.26E-05	1.84E-04
4600	ENSMUSG00000022094	Slc39a14	0.93	8.26E-05	1.84E-04
4601	ENSMUSG00000000579	Dynlt1c	0.70	8.27E-05	1.84E-04
4602	ENSMUSG00000023050	Map3k12	0.79	8.28E-05	1.84E-04
4603	ENSMUSG00000003518	Dusp3	0.62	8.29E-05	1.84E-04
4604	ENSMUSG00000025917	Cops5	0.56	8.33E-05	1.85E-04
4605	ENSMUSG00000096255	Dynlt1b	0.61	8.35E-05	1.86E-04
4606	ENSMUSG00000024055	Cyp4f13	0.66	8.40E-05	1.86E-04
4607	ENSMUSG00000026933	Camsap1	-0.86	8.43E-05	1.87E-04
4608	ENSMUSG00000035686	Thrsp	0.84	8.46E-05	1.88E-04
4609	ENSMUSG00000024360	Etf1	0.55	8.48E-05	1.88E-04
4610	ENSMUSG00000032423	Syncrip	-0.69	8.48E-05	1.88E-04
4611	ENSMUSG00000025209	Twnk	0.69	8.53E-05	1.89E-04
4612	ENSMUSG00000027680	Fxr1	0.68	8.53E-05	1.89E-04
4613	ENSMUSG00000051391	Ywhag	-0.65	8.54E-05	1.89E-04
4614	ENSMUSG00000057572	Zbtb8os	0.68	8.55E-05	1.90E-04
4615	ENSMUSG00000023022	Lima1	-0.81	8.56E-05	1.90E-04
4616	ENSMUSG00000028521	Slc35d1	-0.94	8.56E-05	1.90E-04
4617	ENSMUSG00000040720	1110037F02Rik	-0.84	8.67E-05	1.92E-04
4618	ENSMUSG00000023452	Pisd	0.57	8.71E-05	1.93E-04
4619	ENSMUSG00000018199	Trove2	-0.87	8.76E-05	1.94E-04
4620	ENSMUSG00000079334	Nat6	0.58	8.77E-05	1.94E-04
4621	ENSMUSG00000035495	Tstd2	-0.67	8.80E-05	1.95E-04
4622	ENSMUSG00000033953	Ppp3r1	-0.58	8.81E-05	1.95E-04
4623	ENSMUSG00000035575	Utp6	-0.72	8.83E-05	1.95E-04
4624	ENSMUSG00000014195	Dnajc7	0.60	8.83E-05	1.95E-04
4625	ENSMUSG00000027255	Arfgap2	-0.58	8.84E-05	1.95E-04
4626	ENSMUSG00000018672	Copz2	0.57	8.88E-05	1.96E-04
4627	ENSMUSG00000024098	Twsg1	-0.67	8.91E-05	1.97E-04
4628	ENSMUSG00000034751	Mast4	-0.99	8.95E-05	1.98E-04
4629	ENSMUSG00000014771	Pdcd2	0.82	8.96E-05	1.98E-04
4630	ENSMUSG00000006373	Pgrmc1	0.55	8.98E-05	1.98E-04
4631	ENSMUSG00000078619	Smarcd2	-0.64	8.99E-05	1.98E-04
4632	ENSMUSG00000026848	Tor1b	-0.71	8.99E-05	1.98E-04
4633	ENSMUSG00000029703	Lrwd1	0.72	9.00E-05	1.99E-04
4634	ENSMUSG00000026822	Lcn2	-1.11	9.01E-05	1.99E-04
4635	ENSMUSG00000001227	Sema6b	-0.85	9.07E-05	2.00E-04
4636	ENSMUSG00000029192	Tbc1d14	-0.81	9.07E-05	2.00E-04
4637	ENSMUSG00000036533	Cdc42ep3	-0.94	9.10E-05	2.01E-04
4638	ENSMUSG00000053175	Bcl3	0.79	9.10E-05	2.01E-04
4639	ENSMUSG00000034192	Lsm3	0.72	9.11E-05	2.01E-04
4640	ENSMUSG00000030880	Polr3e	-0.75	9.12E-05	2.01E-04
4641	ENSMUSG00000007041	Clic1	-0.88	9.12E-05	2.01E-04
4642	ENSMUSG00000022092	Ppp3cc	0.81	9.13E-05	2.01E-04
4643	ENSMUSG00000034593	Myo5a	0.61	9.17E-05	2.02E-04
4644	ENSMUSG00000063888	Rpl7l1	0.60	9.20E-05	2.03E-04
4645	ENSMUSG00000030662	Ipo5	-0.67	9.21E-05	2.03E-04
4646	ENSMUSG00000032667	Pon2	-0.64	9.23E-05	2.03E-04
4647	ENSMUSG00000044600	Smim7	0.58	9.27E-05	2.04E-04

4648	ENSMUSG00000069899	Gm12166	0.86	9.28E-05	2.04E-04
4649	ENSMUSG00000025888	Casp1	-1.11	9.31E-05	2.05E-04
4650	ENSMUSG00000031314	Taf1	-0.92	9.31E-05	2.05E-04
4651	ENSMUSG00000029199	Lias	0.56	9.33E-05	2.05E-04
4652	ENSMUSG00000042770	Hebp1	-0.75	9.35E-05	2.06E-04
4653	ENSMUSG00000097786	4933429H19Rik	0.77	9.35E-05	2.06E-04
4654	ENSMUSG00000090208	Gm15851	-0.67	9.37E-05	2.06E-04
4655	ENSMUSG00000031584	Gsr	-0.59	9.39E-05	2.06E-04
4656	ENSMUSG00000054428	Atpif1	0.81	9.44E-05	2.07E-04
4657	ENSMUSG00000051373	Plpp7	1.04	9.45E-05	2.08E-04
4658	ENSMUSG00000039176	Polg	-0.60	9.52E-05	2.09E-04
4659	ENSMUSG00000006464	Bbs1	-0.98	9.52E-05	2.09E-04
4660	ENSMUSG00000030720	Cln3	0.70	9.57E-05	2.10E-04
4661	ENSMUSG00000046794	Ppp1r3b	0.83	9.63E-05	2.11E-04
4662	ENSMUSG00000035754	Wdr18	0.57	9.67E-05	2.12E-04
4663	ENSMUSG00000029173	Sepsecs	-0.70	9.69E-05	2.12E-04
4664	ENSMUSG00000022040	Ephx2	0.58	9.70E-05	2.13E-04
4665	ENSMUSG00000020260	Pofut2	0.58	9.71E-05	2.13E-04
4666	ENSMUSG00000042046	Dstyk	-0.84	9.71E-05	2.13E-04
4667	ENSMUSG00000041203	2310036O22Rik	0.73	9.73E-05	2.13E-04
4668	ENSMUSG00000060708	Bloc1s4	0.74	9.73E-05	2.13E-04
4669	ENSMUSG00000033487	Fndc3a	-0.75	9.82E-05	2.15E-04
4670	ENSMUSG00000027774	Gfm1	0.57	9.85E-05	2.16E-04
4671	ENSMUSG00000053110	Yap1	-0.76	9.85E-05	2.16E-04
4672	ENSMUSG00000058997	Vwa8	0.56	9.90E-05	2.17E-04
4673	ENSMUSG00000013539	Tango2	0.63	9.91E-05	2.17E-04
4674	ENSMUSG00000000266	Mid2	-0.99	9.93E-05	2.17E-04
4675	ENSMUSG00000032998	Foxj3	-0.80	1.01E-04	2.20E-04
4676	ENSMUSG00000037257	Aagab	-0.65	1.01E-04	2.20E-04
4677	ENSMUSG00000009076	Zmat5	0.72	1.01E-04	2.20E-04
4678	ENSMUSG00000047921	Trappc9	-0.58	1.01E-04	2.21E-04
4679	ENSMUSG00000097048	1600020E01Rik	0.96	1.01E-04	2.21E-04
4680	ENSMUSG00000047793	Sned1	-0.92	1.01E-04	2.21E-04
4681	ENSMUSG00000023353	Agap3	-0.60	1.01E-04	2.21E-04
4682	ENSMUSG00000020776	Fbf1	-0.97	1.02E-04	2.22E-04
4683	ENSMUSG00000011179	Odc1	0.56	1.02E-04	2.22E-04
4684	ENSMUSG00000020706	Ftsj3	-0.69	1.02E-04	2.23E-04
4685	ENSMUSG00000014850	Msh3	-0.88	1.02E-04	2.23E-04
4686	ENSMUSG00000029780	Nt5c3	0.79	1.02E-04	2.23E-04
4687	ENSMUSG00000028538	St3gal3	0.70	1.02E-04	2.23E-04
4688	ENSMUSG00000039509	Nup133	-0.82	1.03E-04	2.24E-04
4689	ENSMUSG00000026082	Rev1	-0.98	1.03E-04	2.24E-04
4690	ENSMUSG00000031090	Nadsyn1	0.76	1.03E-04	2.24E-04
4691	ENSMUSG00000036376	Abt1	0.78	1.04E-04	2.26E-04
4692	ENSMUSG00000016503	Gtf3a	0.58	1.04E-04	2.26E-04
4693	ENSMUSG00000075602	Ly6a	-0.79	1.04E-04	2.26E-04
4694	ENSMUSG00000044496	2510039O18Rik	-0.62	1.04E-04	2.27E-04
4695	ENSMUSG00000022234	Cct5	0.54	1.04E-04	2.27E-04
4696	ENSMUSG00000037455	Slc18b1	-0.68	1.05E-04	2.28E-04
4697	ENSMUSG00000057130	Txnl4a	0.57	1.05E-04	2.28E-04

4698	ENSMUSG00000001053	N4bp3	-0.93	1.05E-04	2.29E-04
4699	ENSMUSG00000029790	Cep41	-1.08	1.06E-04	2.31E-04
4700	ENSMUSG00000031197	Vbp1	0.70	1.06E-04	2.31E-04
4701	ENSMUSG00000076435	Acsf2	0.57	1.07E-04	2.33E-04
4702	ENSMUSG00000085667	Gm12992	-0.76	1.08E-04	2.34E-04
4703	ENSMUSG00000106864	Gtf3c2	-0.60	1.08E-04	2.34E-04
4704	ENSMUSG00000085636	Gm11769	-0.73	1.08E-04	2.34E-04
4705	ENSMUSG00000079418	Atg4a	0.68	1.08E-04	2.35E-04
4706	ENSMUSG00000057367	Birc2	-0.71	1.08E-04	2.35E-04
4707	ENSMUSG00000030224	Strap	0.56	1.08E-04	2.35E-04
4708	ENSMUSG00000025813	Homer2	-1.00	1.08E-04	2.35E-04
4709	ENSMUSG00000024831	Ighmbp2	-0.92	1.09E-04	2.36E-04
4710	ENSMUSG00000004937	Sgta	0.54	1.09E-04	2.36E-04
4711	ENSMUSG00000018965	Ywhah	-0.59	1.10E-04	2.39E-04
4712	ENSMUSG00000030374	Strn4	-0.63	1.10E-04	2.39E-04
4713	ENSMUSG00000024045	Akap8	-0.74	1.10E-04	2.39E-04
4714	ENSMUSG00000031447	Lamp1	0.56	1.10E-04	2.39E-04
4715	ENSMUSG00000040738	Ints8	-0.83	1.10E-04	2.40E-04
4716	ENSMUSG00000109941	Exosc6	0.78	1.11E-04	2.40E-04
4717	ENSMUSG00000084970	1700060J05Rik	-0.87	1.11E-04	2.41E-04
4718	ENSMUSG00000022710	Usp7	-0.65	1.11E-04	2.41E-04
4719	ENSMUSG00000003226	Ranbp2	-0.65	1.11E-04	2.41E-04
4720	ENSMUSG00000078427	Sarnp	0.65	1.12E-04	2.42E-04
4721	ENSMUSG00000102191	Gm36569	-0.97	1.12E-04	2.42E-04
4722	ENSMUSG00000022807	Osbpl11	-0.73	1.12E-04	2.43E-04
4723	ENSMUSG00000041774	Ydjc	0.63	1.13E-04	2.44E-04
4724	ENSMUSG00000037816	Fbxw17	-0.84	1.13E-04	2.45E-04
4725	ENSMUSG00000002409	Dyrk1b	0.60	1.13E-04	2.45E-04
4726	ENSMUSG00000047539	Fbxo28	-0.69	1.14E-04	2.47E-04
4727	ENSMUSG00000008167	Fbxw9	-0.79	1.14E-04	2.47E-04
4728	ENSMUSG00000026355	Mcm6	-1.05	1.14E-04	2.48E-04
4729	ENSMUSG00000029470	P2rx4	0.62	1.15E-04	2.49E-04
4730	ENSMUSG00000018405	Mrm1	0.78	1.15E-04	2.49E-04
4731	ENSMUSG00000087579	1500017E21Rik	-1.05	1.15E-04	2.49E-04
4732	ENSMUSG00000029705	Cux1	0.60	1.15E-04	2.49E-04
4733	ENSMUSG00000029234	Tmem165	-0.80	1.16E-04	2.50E-04
4734	ENSMUSG00000045098	Kmt5b	-0.88	1.16E-04	2.50E-04
4735	ENSMUSG00000024998	Plce1	-0.93	1.16E-04	2.50E-04
4736	ENSMUSG00000030583	Sipa1l3	-0.92	1.16E-04	2.50E-04
4737	ENSMUSG00000019487	Trip10	-0.78	1.16E-04	2.51E-04
4738	ENSMUSG00000039450	Dcxr	-0.65	1.17E-04	2.52E-04
4739	ENSMUSG00000031691	Tnpo2	-0.65	1.17E-04	2.53E-04
4740	ENSMUSG00000026615	Eprs	-0.54	1.18E-04	2.54E-04
4741	ENSMUSG00000040591	1110051M20Rik	-0.57	1.19E-04	2.58E-04
4742	ENSMUSG00000022538	Lsg1	-0.76	1.20E-04	2.58E-04
4743	ENSMUSG00000072673	Gm10392	-0.64	1.20E-04	2.59E-04
4744	ENSMUSG00000041268	Dmxl2	-1.03	1.20E-04	2.59E-04
4745	ENSMUSG00000018666	Cbx1	0.83	1.20E-04	2.59E-04
4746	ENSMUSG00000028458	Tesk1	0.57	1.20E-04	2.59E-04
4747	ENSMUSG00000015759	Cnih1	0.53	1.21E-04	2.60E-04

4748	ENSMUSG00000034320	Slc26a2	-0.82	1.21E-04	2.61E-04
4749	ENSMUSG00000042410	Agps	-0.70	1.22E-04	2.62E-04
4750	ENSMUSG00000023965	Fbxl17	-0.73	1.22E-04	2.62E-04
4751	ENSMUSG00000022553	Maf1	0.58	1.22E-04	2.62E-04
4752	ENSMUSG00000016946	Kctd5	-0.75	1.22E-04	2.62E-04
4753	ENSMUSG00000030016	Zfp638	-0.90	1.22E-04	2.63E-04
4754	ENSMUSG00000032860	P2ry2	0.77	1.22E-04	2.63E-04
4755	ENSMUSG00000066235	Pomgnt2	-0.74	1.23E-04	2.64E-04
4756	ENSMUSG00000018501	Ncor1	-0.61	1.23E-04	2.64E-04
4757	ENSMUSG00000025193	Cutc	0.77	1.24E-04	2.66E-04
4758	ENSMUSG00000029569	Tmem168	-0.75	1.24E-04	2.67E-04
4759	ENSMUSG00000047417	Rexo1	-0.71	1.24E-04	2.67E-04
4760	ENSMUSG00000019842	Traf3ip2	-1.04	1.24E-04	2.67E-04
4761	ENSMUSG00000086158	Ccpg1os	0.93	1.24E-04	2.67E-04
4762	ENSMUSG00000030983	Bccip	0.61	1.24E-04	2.67E-04
4763	ENSMUSG00000086514	Gm11747	-1.03	1.25E-04	2.67E-04
4764	ENSMUSG00000018800	Abca5	-0.93	1.25E-04	2.69E-04
4765	ENSMUSG00000015341	Golga7	0.57	1.26E-04	2.69E-04
4766	ENSMUSG00000020840	Blmh	-0.60	1.26E-04	2.69E-04
4767	ENSMUSG00000049775	Tmsb4x	-0.81	1.27E-04	2.72E-04
4768	ENSMUSG00000001416	Cct3	0.53	1.27E-04	2.72E-04
4769	ENSMUSG00000030498	Gas2	-0.89	1.28E-04	2.74E-04
4770	ENSMUSG00000033792	Atp7a	-0.90	1.28E-04	2.74E-04
4771	ENSMUSG00000048758	Rpl29	0.63	1.28E-04	2.74E-04
4772	ENSMUSG00000066880	Zfp617	0.68	1.28E-04	2.75E-04
4773	ENSMUSG00000039533	Mmd2	1.00	1.28E-04	2.75E-04
4774	ENSMUSG00000070047	Fat1	-0.91	1.28E-04	2.75E-04
4775	ENSMUSG00000031304	Il2rg	-1.02	1.29E-04	2.75E-04
4776	ENSMUSG00000029309	Sparcl1	-0.71	1.29E-04	2.76E-04
4777	ENSMUSG00000015605	Srf	-0.68	1.29E-04	2.77E-04
4778	ENSMUSG00000018750	Zbtb4	-0.83	1.30E-04	2.78E-04
4779	ENSMUSG00000023235	Ccl25	0.88	1.30E-04	2.78E-04
4780	ENSMUSG00000032410	Xrn1	-0.91	1.30E-04	2.78E-04
4781	ENSMUSG00000020424	Gatsl3	-0.90	1.31E-04	2.80E-04
4782	ENSMUSG00000024667	Tmem216	-0.82	1.31E-04	2.80E-04
4783	ENSMUSG00000027366	Sppl2a	0.59	1.31E-04	2.81E-04
4784	ENSMUSG00000042251	Pm20d1	0.70	1.32E-04	2.81E-04
4785	ENSMUSG00000035521	Gnptg	0.60	1.32E-04	2.82E-04
4786	ENSMUSG00000068854	Hist2h2be	0.97	1.32E-04	2.82E-04
4787	ENSMUSG00000025437	Usp33	-0.58	1.32E-04	2.83E-04
4788	ENSMUSG00000036327	Qsox2	-0.79	1.33E-04	2.84E-04
4789	ENSMUSG00000009073	Nf2	-0.62	1.34E-04	2.86E-04
4790	ENSMUSG00000019732	Calr3	0.85	1.35E-04	2.88E-04
4791	ENSMUSG00000041130	Zfp598	-0.96	1.35E-04	2.88E-04
4792	ENSMUSG00000018923	Med11	0.82	1.35E-04	2.89E-04
4793	ENSMUSG00000087341	0610040F04Rik	-0.71	1.36E-04	2.89E-04
4794	ENSMUSG00000025417	Pip4k2c	-0.68	1.36E-04	2.90E-04
4795	ENSMUSG00000058254	Tspan7	-0.61	1.36E-04	2.90E-04
4796	ENSMUSG00000114046	AC124712.1	-0.84	1.36E-04	2.91E-04
4797	ENSMUSG00000030008	Pradc1	0.69	1.37E-04	2.91E-04

4798	ENSMUSG00000044991	1110034G24Rik	0.86	1.37E-04	2.92E-04
4799	ENSMUSG00000038914	Dido1	-0.77	1.37E-04	2.93E-04
4800	ENSMUSG00000031974	Abcb10	-0.62	1.38E-04	2.95E-04
4801	ENSMUSG00000066877	Nck2	-0.86	1.38E-04	2.95E-04
4802	ENSMUSG00000013150	Gfod2	-0.74	1.40E-04	2.97E-04
4803	ENSMUSG00000007480	Mc5r	-1.00	1.40E-04	2.97E-04
4804	ENSMUSG00000020079	Supv3l1	0.64	1.40E-04	2.98E-04
4805	ENSMUSG00000056429	Tgoln1	0.55	1.40E-04	2.98E-04
4806	ENSMUSG00000100679	Gm28778	0.88	1.41E-04	2.99E-04
4807	ENSMUSG00000053749	Gm9920	0.83	1.41E-04	2.99E-04
4808	ENSMUSG00000020650	Bcap29	0.61	1.41E-04	2.99E-04
4809	ENSMUSG00000038042	Ptpdc1	-0.88	1.41E-04	3.00E-04
4810	ENSMUSG00000029068	Ccnl2	0.58	1.42E-04	3.02E-04
4811	ENSMUSG00000035545	Leng8	-0.78	1.43E-04	3.03E-04
4812	ENSMUSG00000032232	Cgnl1	0.60	1.43E-04	3.03E-04
4813	ENSMUSG00000029202	Pds5a	-0.69	1.43E-04	3.04E-04
4814	ENSMUSG00000020601	Trib2	-0.93	1.44E-04	3.05E-04
4815	ENSMUSG00000028976	Slc2a5	0.90	1.44E-04	3.05E-04
4816	ENSMUSG00000022401	Xpnpep3	-0.68	1.44E-04	3.06E-04
4817	ENSMUSG00000022536	Glyr1	-0.60	1.45E-04	3.07E-04
4818	ENSMUSG00000027284	Cdan1	-0.85	1.45E-04	3.07E-04
4819	ENSMUSG00000031627	Irf2	-0.92	1.45E-04	3.07E-04
4820	ENSMUSG00000033016	Nfatc1	-0.70	1.45E-04	3.08E-04
4821	ENSMUSG00000017760	Ctsa	-0.52	1.45E-04	3.08E-04
4822	ENSMUSG00000025555	Farp1	-0.81	1.45E-04	3.08E-04
4823	ENSMUSG00000029535	Triap1	0.61	1.46E-04	3.09E-04
4824	ENSMUSG00000032878	Ccdc85a	-0.87	1.46E-04	3.09E-04
4825	ENSMUSG00000033411	Ctdspl2	-0.90	1.46E-04	3.10E-04
4826	ENSMUSG00000034485	Uaca	-0.84	1.46E-04	3.10E-04
4827	ENSMUSG00000005774	Rfx5	-0.93	1.47E-04	3.11E-04
4828	ENSMUSG00000006335	Tfpt	0.79	1.47E-04	3.11E-04
4829	ENSMUSG00000030532	Hddc3	0.70	1.47E-04	3.12E-04
4830	ENSMUSG00000038205	Prkab2	-0.79	1.47E-04	3.12E-04
4831	ENSMUSG00000037369	Kdm6a	-0.86	1.48E-04	3.13E-04
4832	ENSMUSG00000040483	Xaf1	-0.92	1.48E-04	3.13E-04
4833	ENSMUSG00000034064	Poglut1	-0.64	1.49E-04	3.14E-04
4834	ENSMUSG00000032939	Nup93	-0.90	1.49E-04	3.14E-04
4835	ENSMUSG00000037523	Mavs	0.57	1.49E-04	3.15E-04
4836	ENSMUSG00000048832	Vps37c	-0.64	1.50E-04	3.16E-04
4837	ENSMUSG00000030410	Dmwd	-0.63	1.50E-04	3.16E-04
4838	ENSMUSG00000001229	Dpp9	-0.67	1.50E-04	3.17E-04
4839	ENSMUSG00000037997	Parp11	-0.89	1.50E-04	3.17E-04
4840	ENSMUSG00000013419	Zfp651	-0.74	1.50E-04	3.18E-04
4841	ENSMUSG00000019961	Tmpo	-0.88	1.51E-04	3.18E-04
4842	ENSMUSG00000032849	Abcc4	0.86	1.51E-04	3.20E-04
4843	ENSMUSG00000068267	Cenpb	0.60	1.52E-04	3.20E-04
4844	ENSMUSG00000036810	Cnep1r1	0.66	1.52E-04	3.21E-04
4845	ENSMUSG00000031161	Hdac6	-0.67	1.54E-04	3.26E-04
4846	ENSMUSG00000025854	Fam20c	-0.65	1.55E-04	3.27E-04
4847	ENSMUSG00000014074	Rnf168	-0.98	1.57E-04	3.30E-04

4848	ENSMUSG00000024948	Map4k2	-0.75	1.57E-04	3.32E-04
4849	ENSMUSG00000050229	Pigm	-0.78	1.58E-04	3.33E-04
4850	ENSMUSG00000035476	Tab3	-0.99	1.58E-04	3.34E-04
4851	ENSMUSG00000037234	Hook3	-0.68	1.59E-04	3.35E-04
4852	ENSMUSG00000079426	Arpc4	0.53	1.60E-04	3.37E-04
4853	ENSMUSG00000025145	Lrrc45	-1.01	1.60E-04	3.38E-04
4854	ENSMUSG00000019952	Poc1b	-0.82	1.62E-04	3.41E-04
4855	ENSMUSG00000024182	Axin1	-0.71	1.62E-04	3.41E-04
4856	ENSMUSG00000028957	Per3	-0.81	1.63E-04	3.44E-04
4857	ENSMUSG00000087367	Gm15491	-0.92	1.63E-04	3.44E-04
4858	ENSMUSG00000097762	4732463B04Rik	0.65	1.64E-04	3.44E-04
4859	ENSMUSG00000028907	Utp11	0.91	1.64E-04	3.45E-04
4860	ENSMUSG00000046806	3110062M04Rik	0.62	1.64E-04	3.45E-04
4861	ENSMUSG00000034032	Rpap1	-0.77	1.65E-04	3.46E-04
4862	ENSMUSG00000055912	Tmem150a	0.76	1.65E-04	3.46E-04
4863	ENSMUSG00000028797	Tmem234	0.60	1.66E-04	3.48E-04
4864	ENSMUSG00000025862	Stag2	-0.67	1.66E-04	3.48E-04
4865	ENSMUSG00000054387	Mdm4	-0.78	1.66E-04	3.49E-04
4866	ENSMUSG00000032315	Cyp1a1	-0.94	1.66E-04	3.50E-04
4867	ENSMUSG00000027575	Arfgap1	-0.62	1.67E-04	3.50E-04
4868	ENSMUSG00000024095	Hnrnpll	-0.70	1.67E-04	3.52E-04
4869	ENSMUSG00000033712	Ccar2	-0.63	1.68E-04	3.54E-04
4870	ENSMUSG00000039834	Zfp335	-0.71	1.69E-04	3.54E-04
4871	ENSMUSG00000025537	Phkg1	-0.88	1.69E-04	3.56E-04
4872	ENSMUSG00000022808	Snx4	-0.58	1.70E-04	3.56E-04
4873	ENSMUSG00000026921	Egfl7	0.59	1.70E-04	3.56E-04
4874	ENSMUSG00000048170	Mcmbp	-0.60	1.72E-04	3.60E-04
4875	ENSMUSG00000024271	Elp2	-0.57	1.72E-04	3.60E-04
4876	ENSMUSG00000037703	Lzts3	-0.92	1.72E-04	3.61E-04
4877	ENSMUSG00000008763	Man1a2	-0.72	1.72E-04	3.61E-04
4878	ENSMUSG00000074656	Eif2s2	0.62	1.72E-04	3.61E-04
4879	ENSMUSG00000097493	9930014A18Rik	-0.92	1.74E-04	3.64E-04
4880	ENSMUSG00000054693	Adam10	-0.73	1.74E-04	3.65E-04
4881	ENSMUSG00000020349	Ppp2ca	0.54	1.74E-04	3.65E-04
4882	ENSMUSG00000090698	Apold1	-1.02	1.75E-04	3.66E-04
4883	ENSMUSG00000018442	Derl2	0.59	1.75E-04	3.66E-04
4884	ENSMUSG00000099966	2810402E24Rik	0.77	1.75E-04	3.67E-04
4885	ENSMUSG00000021709	Erbin	-0.78	1.76E-04	3.68E-04
4886	ENSMUSG00000030806	Stx1b	-0.73	1.77E-04	3.70E-04
4887	ENSMUSG00000030304	Ergic2	0.57	1.77E-04	3.70E-04
4888	ENSMUSG00000021646	Mccc2	-0.52	1.77E-04	3.70E-04
4889	ENSMUSG00000020715	Ern1	-0.89	1.79E-04	3.74E-04
4890	ENSMUSG00000027030	Stk39	-0.72	1.79E-04	3.75E-04
4891	ENSMUSG00000105203	4933428P19Rik	-0.76	1.79E-04	3.75E-04
4892	ENSMUSG00000079610	Ankrd39	0.76	1.79E-04	3.75E-04
4893	ENSMUSG00000039128	Cdc123	0.53	1.80E-04	3.75E-04
4894	ENSMUSG00000044155	Lsm8	0.87	1.80E-04	3.77E-04
4895	ENSMUSG00000095362	Gm14325	0.72	1.81E-04	3.77E-04
4896	ENSMUSG00000020311	Erlec1	0.77	1.81E-04	3.77E-04
4897	ENSMUSG00000002732	Fkbp7	0.62	1.81E-04	3.78E-04

4898	ENSMUSG00000004054	Map3k11	-0.64	1.83E-04	3.81E-04
4899	ENSMUSG00000008384	Sertad1	-0.66	1.83E-04	3.81E-04
4900	ENSMUSG00000009621	Vav2	-0.75	1.83E-04	3.81E-04
4901	ENSMUSG00000042228	Lyn	-0.80	1.83E-04	3.82E-04
4902	ENSMUSG00000052392	Acot4	0.59	1.83E-04	3.82E-04
4903	ENSMUSG00000007805	Twist2	-1.04	1.83E-04	3.83E-04
4904	ENSMUSG00000055799	Tcf7l1	-0.90	1.84E-04	3.83E-04
4905	ENSMUSG00000097731	2700016F22Rik	-0.67	1.86E-04	3.87E-04
4906	ENSMUSG00000019188	H13	0.52	1.86E-04	3.87E-04
4907	ENSMUSG00000032480	Dhx30	-0.57	1.86E-04	3.88E-04
4908	ENSMUSG00000018209	Stk4	-0.68	1.87E-04	3.90E-04
4909	ENSMUSG00000038416	Cdc16	-0.55	1.88E-04	3.92E-04
4910	ENSMUSG00000032121	Tmem218	0.88	1.89E-04	3.93E-04
4911	ENSMUSG00000004071	Cdip1	0.55	1.89E-04	3.94E-04
4912	ENSMUSG00000037805	Rpl10a	0.66	1.90E-04	3.95E-04
4913	ENSMUSG00000078866	Zfp970	-0.72	1.92E-04	4.00E-04
4914	ENSMUSG00000019966	Kitl	-0.83	1.93E-04	4.01E-04
4915	ENSMUSG00000028893	Sesn2	-0.68	1.93E-04	4.01E-04
4916	ENSMUSG00000021903	Galnt15	-0.93	1.93E-04	4.01E-04
4917	ENSMUSG00000021224	Numb	-0.77	1.93E-04	4.02E-04
4918	ENSMUSG00000034403	Pja1	-0.60	1.93E-04	4.02E-04
4919	ENSMUSG00000030557	Mef2a	-0.55	1.94E-04	4.02E-04
4920	ENSMUSG00000042305	Tmem183a	0.56	1.94E-04	4.02E-04
4921	ENSMUSG00000033991	Ttc37	-0.79	1.95E-04	4.05E-04
4922	ENSMUSG00000010660	Plcd1	-0.67	1.95E-04	4.05E-04
4923	ENSMUSG00000003402	Prkcsh	0.58	1.96E-04	4.08E-04
4924	ENSMUSG00000048264	Dip2c	-0.89	1.97E-04	4.09E-04
4925	ENSMUSG00000029550	Sppl3	-0.67	1.97E-04	4.10E-04
4926	ENSMUSG00000028381	Ugcg	-0.86	1.98E-04	4.10E-04
4927	ENSMUSG00000051344	Plekhm3	-0.90	1.98E-04	4.11E-04
4928	ENSMUSG00000007739	Cct4	0.53	1.98E-04	4.11E-04
4929	ENSMUSG00000052751	Repin1	-0.68	1.98E-04	4.11E-04
4930	ENSMUSG00000075327	Zbtb2	-0.88	1.98E-04	4.11E-04
4931	ENSMUSG00000022142	Nup155	-1.14	2.00E-04	4.14E-04
4932	ENSMUSG00000038213	Tapbpl	-0.63	2.01E-04	4.16E-04
4933	ENSMUSG00000035478	Mbd3	0.52	2.01E-04	4.17E-04
4934	ENSMUSG00000042284	Itga1	-0.64	2.02E-04	4.19E-04
4935	ENSMUSG00000029007	Agtrap	0.79	2.03E-04	4.20E-04
4936	ENSMUSG00000000827	Tpd52l2	0.54	2.03E-04	4.21E-04
4937	ENSMUSG00000026342	Slc35f5	0.62	2.03E-04	4.21E-04
4938	ENSMUSG00000025810	Nrp1	-0.54	2.04E-04	4.22E-04
4939	ENSMUSG00000040771	Oard1	0.63	2.05E-04	4.24E-04
4940	ENSMUSG00000035529	Prdm4	-0.81	2.05E-04	4.24E-04
4941	ENSMUSG00000019795	Pcmt1	0.53	2.06E-04	4.26E-04
4942	ENSMUSG00000086805	4932443L11Rik	-0.91	2.06E-04	4.27E-04
4943	ENSMUSG00000049047	Armcx3	-0.93	2.07E-04	4.28E-04
4944	ENSMUSG00000021108	Prkch	-0.90	2.08E-04	4.30E-04
4945	ENSMUSG00000099579	1700120K04Rik	0.86	2.08E-04	4.31E-04
4946	ENSMUSG00000066037	Hnrnpr	-0.66	2.08E-04	4.31E-04
4947	ENSMUSG00000022503	Nubp1	0.61	2.09E-04	4.31E-04

4948	ENSMUSG00000034255	Arhgap27	-0.82	2.09E-04	4.32E-04
4949	ENSMUSG00000003847	Nfat5	-0.82	2.10E-04	4.33E-04
4950	ENSMUSG00000040451	Sgms1	-0.61	2.10E-04	4.33E-04
4951	ENSMUSG00000022350	Washc5	-0.61	2.10E-04	4.33E-04
4952	ENSMUSG00000029009	Mthfr	-0.74	2.11E-04	4.35E-04
4953	ENSMUSG00000038271	Iffo1	-0.96	2.11E-04	4.35E-04
4954	ENSMUSG00000037225	Fgf2	-0.68	2.11E-04	4.36E-04
4955	ENSMUSG00000102349	Gm37376	0.66	2.13E-04	4.40E-04
4956	ENSMUSG00000027808	Serp1	0.57	2.14E-04	4.42E-04
4957	ENSMUSG00000027650	Tti1	-0.64	2.17E-04	4.48E-04
4958	ENSMUSG00000025159	Mms19	-0.72	2.17E-04	4.48E-04
4959	ENSMUSG00000085213	Gm13091	-0.96	2.17E-04	4.48E-04
4960	ENSMUSG00000022300	Dcaf13	0.59	2.18E-04	4.49E-04
4961	ENSMUSG00000024241	Sos1	-0.66	2.18E-04	4.49E-04
4962	ENSMUSG00000026248	Mrpl44	0.64	2.18E-04	4.50E-04
4963	ENSMUSG00000002475	Abhd3	-0.99	2.19E-04	4.51E-04
4964	ENSMUSG00000015711	Prune1	-0.67	2.19E-04	4.51E-04
4965	ENSMUSG00000003273	Car11	0.83	2.19E-04	4.51E-04
4966	ENSMUSG00000038594	Cep85l	-0.86	2.19E-04	4.51E-04
4967	ENSMUSG00000097099	Gm9917	0.57	2.21E-04	4.55E-04
4968	ENSMUSG00000021244	Ylpm1	-0.83	2.23E-04	4.58E-04
4969	ENSMUSG00000025184	R3hcc1l	-0.86	2.23E-04	4.59E-04
4970	ENSMUSG00000001240	Ramp2	0.52	2.23E-04	4.60E-04
4971	ENSMUSG00000030802	Bckdk	0.55	2.24E-04	4.60E-04
4972	ENSMUSG00000049672	Zbtb14	-0.77	2.26E-04	4.64E-04
4973	ENSMUSG00000035382	Pcsk7	-0.59	2.26E-04	4.64E-04
4974	ENSMUSG00000020275	Rel	-0.97	2.27E-04	4.68E-04
4975	ENSMUSG00000025217	Btrc	-0.71	2.28E-04	4.69E-04
4976	ENSMUSG00000026193	Fn1	-1.59	2.28E-04	4.69E-04
4977	ENSMUSG00000040681	Hmgn1	-0.60	2.29E-04	4.70E-04
4978	ENSMUSG00000078515	Ddi2	-0.59	2.31E-04	4.74E-04
4979	ENSMUSG00000032097	Ddx6	-0.62	2.32E-04	4.76E-04
4980	ENSMUSG00000028107	Tars2	0.58	2.32E-04	4.77E-04
4981	ENSMUSG00000009406	Elk1	-0.78	2.33E-04	4.79E-04
4982	ENSMUSG00000007891	Ctsd	0.59	2.34E-04	4.79E-04
4983	ENSMUSG00000040007	Bahd1	-0.89	2.34E-04	4.80E-04
4984	ENSMUSG00000002428	Hltf	-0.94	2.34E-04	4.81E-04
4985	ENSMUSG00000090327	Gm17111	0.89	2.35E-04	4.81E-04
4986	ENSMUSG00000060681	Slc9a6	-0.85	2.35E-04	4.82E-04
4987	ENSMUSG00000026131	Dst	-0.68	2.35E-04	4.83E-04
4988	ENSMUSG00000020737	Hn1	-0.62	2.36E-04	4.84E-04
4989	ENSMUSG00000090026	Gm15996	0.69	2.36E-04	4.84E-04
4990	ENSMUSG00000032285	Dnaja4	0.63	2.38E-04	4.87E-04
4991	ENSMUSG00000022200	Golph3	0.54	2.38E-04	4.88E-04
4992	ENSMUSG00000110040	AC034099.1	0.57	2.41E-04	4.93E-04
4993	ENSMUSG00000109493	Gm45208	0.58	2.41E-04	4.94E-04
4994	ENSMUSG00000025894	Aasdhppt	0.59	2.42E-04	4.95E-04
4995	ENSMUSG00000025147	Mob2	0.61	2.42E-04	4.96E-04
4996	ENSMUSG00000091661	Gm17021	-0.90	2.44E-04	4.99E-04
4997	ENSMUSG00000028495	Rps6	0.54	2.44E-04	4.99E-04

4998	ENSMUSG00000037965	Zc3h7a	-0.76	2.45E-04	5.01E-04
4999	ENSMUSG00000037993	Dhx38	-0.72	2.45E-04	5.01E-04
5000	ENSMUSG00000000686	Abhd15	-0.55	2.46E-04	5.04E-04
5001	ENSMUSG00000028986	Klhl7	-0.64	2.50E-04	5.10E-04
5002	ENSMUSG00000019841	Rev3l	-0.90	2.50E-04	5.10E-04
5003	ENSMUSG00000028884	Rpa2	-0.85	2.50E-04	5.11E-04
5004	ENSMUSG00000058812	0610039K10Rik	-0.73	2.51E-04	5.12E-04
5005	ENSMUSG00000003549	Ercc1	0.64	2.51E-04	5.12E-04
5006	ENSMUSG00000028423	Nfx1	-0.60	2.52E-04	5.14E-04
5007	ENSMUSG00000022516	Nudt16l1	-0.52	2.52E-04	5.14E-04
5008	ENSMUSG00000042348	Arl15	-0.70	2.54E-04	5.18E-04
5009	ENSMUSG00000031012	Cask	-0.74	2.54E-04	5.19E-04
5010	ENSMUSG00000030232	Aebp2	-0.69	2.55E-04	5.20E-04
5011	ENSMUSG00000026387	Sctr	-0.81	2.55E-04	5.21E-04
5012	ENSMUSG00000035171	1110059E24Rik	0.67	2.56E-04	5.23E-04
5013	ENSMUSG00000025355	Mmp19	-0.69	2.57E-04	5.24E-04
5014	ENSMUSG00000026031	Cflar	-0.56	2.58E-04	5.27E-04
5015	ENSMUSG00000039634	Zfp189	0.90	2.59E-04	5.28E-04
5016	ENSMUSG00000027936	Crtc2	-0.64	2.59E-04	5.28E-04
5017	ENSMUSG00000022387	Brd1	-0.69	2.60E-04	5.30E-04
5018	ENSMUSG00000022003	Slc25a30	-1.01	2.60E-04	5.30E-04
5019	ENSMUSG00000002944	Cd36	-0.57	2.61E-04	5.31E-04
5020	ENSMUSG00000045752	Tssc4	0.65	2.61E-04	5.32E-04
5021	ENSMUSG00000024870	Rab1b	0.52	2.64E-04	5.38E-04
5022	ENSMUSG00000018412	Kansl1	-0.66	2.64E-04	5.38E-04
5023	ENSMUSG00000056234	Ncoa4	-0.51	2.65E-04	5.39E-04
5024	ENSMUSG00000030654	Arl6ip1	0.51	2.66E-04	5.40E-04
5025	ENSMUSG00000107023	Gm42715	-0.61	2.66E-04	5.40E-04
5026	ENSMUSG00000092274	Neat1	0.67	2.66E-04	5.40E-04
5027	ENSMUSG00000003039	Fam32a	0.52	2.66E-04	5.42E-04
5028	ENSMUSG00000030082	Sec61a1	-0.59	2.67E-04	5.44E-04
5029	ENSMUSG00000050199	Lgr4	-0.82	2.70E-04	5.48E-04
5030	ENSMUSG00000031928	Mre11a	-0.83	2.70E-04	5.49E-04
5031	ENSMUSG00000071604	Fam189a2	-0.89	2.72E-04	5.52E-04
5032	ENSMUSG00000093574	Gm20671	0.54	2.72E-04	5.52E-04
5033	ENSMUSG00000073609	D2hgdh	-0.56	2.73E-04	5.54E-04
5034	ENSMUSG00000035960	Apex1	-0.56	2.74E-04	5.56E-04
5035	ENSMUSG00000034088	Hdlbp	-0.55	2.75E-04	5.58E-04
5036	ENSMUSG00000069045	Ddx3y	-0.57	2.75E-04	5.58E-04
5037	ENSMUSG00000041408	Wapl	-0.60	2.76E-04	5.61E-04
5038	ENSMUSG00000020366	Mapk9	-0.55	2.77E-04	5.62E-04
5039	ENSMUSG00000035142	Nubpl	0.66	2.78E-04	5.63E-04
5040	ENSMUSG00000034265	Zdhhc14	-0.77	2.78E-04	5.65E-04
5041	ENSMUSG00000022837	Iqcb1	0.76	2.78E-04	5.65E-04
5042	ENSMUSG00000030109	Slc6a12	-0.92	2.79E-04	5.65E-04
5043	ENSMUSG00000030087	Klf15	-0.61	2.79E-04	5.66E-04
5044	ENSMUSG00000030204	Ddx47	0.58	2.80E-04	5.67E-04
5045	ENSMUSG00000041187	Prkd2	-0.96	2.80E-04	5.68E-04
5046	ENSMUSG00000067235	H2-Q10	0.60	2.81E-04	5.70E-04
5047	ENSMUSG00000052921	Arhgef15	-0.74	2.82E-04	5.71E-04

5048	ENSMUSG00000032612	Usp4	-0.52	2.84E-04	5.75E-04
5049	ENSMUSG00000038563	Efl1	-0.78	2.84E-04	5.75E-04
5050	ENSMUSG00000015994	Fnta	0.49	2.86E-04	5.80E-04
5051	ENSMUSG00000073599	Ecscr	-0.91	2.87E-04	5.81E-04
5052	ENSMUSG00000033933	Vhl	-0.60	2.87E-04	5.81E-04
5053	ENSMUSG00000071347	C1qtnf9	-0.84	2.88E-04	5.83E-04
5054	ENSMUSG00000002803	Btbd6	0.67	2.89E-04	5.84E-04
5055	ENSMUSG00000022220	Adcy4	-0.82	2.89E-04	5.84E-04
5056	ENSMUSG00000001025	S100a6	-1.07	2.89E-04	5.84E-04
5057	ENSMUSG00000087703	Gm15650	-0.84	2.89E-04	5.85E-04
5058	ENSMUSG00000038775	Vill	-1.08	2.91E-04	5.88E-04
5059	ENSMUSG00000000976	Heatr6	-0.67	2.93E-04	5.91E-04
5060	ENSMUSG00000104444	Gm33051	0.82	2.94E-04	5.94E-04
5061	ENSMUSG00000032788	Pdxk	-0.52	2.94E-04	5.94E-04
5062	ENSMUSG00000060166	Zdhhc8	-0.81	2.95E-04	5.95E-04
5063	ENSMUSG00000042426	Dhx29	-0.82	2.95E-04	5.96E-04
5064	ENSMUSG00000019194	Scn1b	-0.68	3.00E-04	6.06E-04
5065	ENSMUSG00000028437	Ubap1	-0.52	3.00E-04	6.06E-04
5066	ENSMUSG00000019868	Vta1	0.58	3.01E-04	6.07E-04
5067	ENSMUSG00000028459	Cd72	0.57	3.02E-04	6.10E-04
5068	ENSMUSG00000054894	Atp5s	0.65	3.02E-04	6.10E-04
5069	ENSMUSG00000020611	Gna13	0.53	3.04E-04	6.13E-04
5070	ENSMUSG00000002205	Vrk3	0.65	3.05E-04	6.15E-04
5071	ENSMUSG00000030747	Dgat2	-0.61	3.05E-04	6.15E-04
5072	ENSMUSG00000020576	Nbas	-0.77	3.06E-04	6.17E-04
5073	ENSMUSG00000027660	Skil	-0.70	3.06E-04	6.18E-04
5074	ENSMUSG00000096751	Gm28373	0.82	3.07E-04	6.19E-04
5075	ENSMUSG00000050697	Prkaa1	-0.57	3.11E-04	6.27E-04
5076	ENSMUSG00000062980	Cped1	-0.55	3.11E-04	6.27E-04
5077	ENSMUSG00000048310	Pskh1	-0.57	3.12E-04	6.29E-04
5078	ENSMUSG00000073758	Sh3d21	-0.82	3.12E-04	6.29E-04
5079	ENSMUSG00000022562	Oplah	0.52	3.13E-04	6.30E-04
5080	ENSMUSG00000039201	Tbc1d25	-0.74	3.15E-04	6.34E-04
5081	ENSMUSG00000030301	Ccdc91	0.52	3.16E-04	6.35E-04
5082	ENSMUSG00000047824	Pygo2	-0.72	3.18E-04	6.39E-04
5083	ENSMUSG00000041415	Dicer1	-0.78	3.19E-04	6.41E-04
5084	ENSMUSG00000028322	Exosc3	0.64	3.19E-04	6.42E-04
5085	ENSMUSG00000034189	Hsdl1	-0.68	3.19E-04	6.42E-04
5086	ENSMUSG00000025287	Acot9	0.53	3.21E-04	6.45E-04
5087	ENSMUSG00000030431	Tmem238	0.65	3.21E-04	6.46E-04
5088	ENSMUSG00000063430	Wscd2	-1.03	3.21E-04	6.46E-04
5089	ENSMUSG00000070939	Tgfbrap1	-0.58	3.22E-04	6.47E-04
5090	ENSMUSG00000021945	Zmym2	-0.80	3.23E-04	6.48E-04
5091	ENSMUSG00000029190	D5Ertd579e	-0.65	3.23E-04	6.49E-04
5092	ENSMUSG00000063894	Zkscan8	-0.91	3.27E-04	6.57E-04
5093	ENSMUSG00000046079	Lrrc8d	-0.58	3.28E-04	6.58E-04
5094	ENSMUSG00000037570	Mcrs1	0.52	3.28E-04	6.58E-04
5095	ENSMUSG00000078922	Tgtp1	-0.85	3.28E-04	6.59E-04
5096	ENSMUSG00000047613	A430005L14Rik	0.73	3.28E-04	6.59E-04
5097	ENSMUSG00000061136	Prpf40a	-0.59	3.28E-04	6.59E-04

5098	ENSMUSG00000109510	Gm42417	0.86	3.29E-04	6.59E-04
5099	ENSMUSG00000053293	Pom121	-0.59	3.30E-04	6.61E-04
5100	ENSMUSG00000024785	Rcl1	-0.67	3.30E-04	6.61E-04
5101	ENSMUSG00000032349	Elovl5	0.71	3.31E-04	6.64E-04
5102	ENSMUSG00000024383	Map3k2	-0.80	3.31E-04	6.64E-04
5103	ENSMUSG00000042197	Zfp451	-0.82	3.33E-04	6.67E-04
5104	ENSMUSG00000038965	Ube2l3	0.50	3.34E-04	6.70E-04
5105	ENSMUSG00000038372	Gmds	-0.81	3.34E-04	6.70E-04
5106	ENSMUSG00000036686	Cc2d1a	-0.75	3.35E-04	6.71E-04
5107	ENSMUSG00000018293	Pfn1	0.50	3.35E-04	6.71E-04
5108	ENSMUSG00000050812	AI314180	-0.56	3.39E-04	6.79E-04
5109	ENSMUSG00000094483	Purb	0.55	3.39E-04	6.79E-04
5110	ENSMUSG00000049734	Trex1	-0.66	3.41E-04	6.82E-04
5111	ENSMUSG00000038861	Pi4kb	-0.67	3.41E-04	6.82E-04
5112	ENSMUSG00000033819	Ppp1r16a	-0.53	3.41E-04	6.82E-04
5113	ENSMUSG00000018796	Acsl1	-0.67	3.44E-04	6.87E-04
5114	ENSMUSG00000040097	Flywch1	-0.57	3.44E-04	6.87E-04
5115	ENSMUSG00000040482	Dxo	-0.60	3.44E-04	6.87E-04
5116	ENSMUSG00000029298	Gbp9	-0.88	3.44E-04	6.88E-04
5117	ENSMUSG00000000439	Mkrn2	-0.56	3.44E-04	6.88E-04
5118	ENSMUSG00000032883	Acsl3	0.56	3.48E-04	6.94E-04
5119	ENSMUSG00000037608	Bclaf1	-0.68	3.48E-04	6.95E-04
5120	ENSMUSG00000035878	Hykk	-0.93	3.49E-04	6.96E-04
5121	ENSMUSG00000017132	Cyth1	0.53	3.50E-04	6.98E-04
5122	ENSMUSG00000052632	Asap2	-0.74	3.50E-04	6.98E-04
5123	ENSMUSG00000020642	Rnf144a	-0.58	3.51E-04	7.00E-04
5124	ENSMUSG00000095567	Noc2l	-0.56	3.51E-04	7.01E-04
5125	ENSMUSG00000024479	Mal2	0.89	3.52E-04	7.03E-04
5126	ENSMUSG00000038160	Atg5	0.54	3.53E-04	7.05E-04
5127	ENSMUSG00000036114	Rpp25l	0.70	3.53E-04	7.05E-04
5128	ENSMUSG00000027405	Nop56	-0.63	3.54E-04	7.05E-04
5129	ENSMUSG00000053641	Dennd4a	-0.76	3.54E-04	7.06E-04
5130	ENSMUSG00000087356	Gm13856	-0.81	3.56E-04	7.09E-04
5131	ENSMUSG00000051674	Dcun1d4	-0.57	3.58E-04	7.13E-04
5132	ENSMUSG00000025162	Csnk1d	-0.52	3.58E-04	7.13E-04
5133	ENSMUSG00000021340	Gpld1	-0.74	3.59E-04	7.15E-04
5134	ENSMUSG00000035198	Tubg1	0.54	3.59E-04	7.15E-04
5135	ENSMUSG00000049124	Gm8186	0.87	3.59E-04	7.16E-04
5136	ENSMUSG00000028974	Dffa	0.71	3.60E-04	7.16E-04
5137	ENSMUSG00000015869	Prpsap1	-0.61	3.60E-04	7.16E-04
5138	ENSMUSG00000036918	Ttc7	-0.66	3.62E-04	7.21E-04
5139	ENSMUSG00000007880	Arid1a	-0.73	3.62E-04	7.21E-04
5140	ENSMUSG00000038290	Smg6	-0.80	3.64E-04	7.24E-04
5141	ENSMUSG00000045620	Odf3l1	-0.62	3.66E-04	7.27E-04
5142	ENSMUSG00000004677	Myo9b	-0.98	3.66E-04	7.28E-04
5143	ENSMUSG00000056167	Cnot10	-0.61	3.66E-04	7.28E-04
5144	ENSMUSG00000027879	Sec22b	0.51	3.67E-04	7.29E-04
5145	ENSMUSG00000060538	Tmem219	0.64	3.67E-04	7.30E-04
5146	ENSMUSG00000029104	Htt	-0.81	3.67E-04	7.30E-04
5147	ENSMUSG00000027514	Zbp1	-1.13	3.68E-04	7.32E-04

5148	ENSMUSG00000100980	Gm29100	-0.95	3.69E-04	7.33E-04
5149	ENSMUSG00000026663	Atf6	-0.58	3.69E-04	7.33E-04
5150	ENSMUSG00000015568	Lpl	-0.57	3.71E-04	7.38E-04
5151	ENSMUSG00000006517	Mvd	-0.75	3.72E-04	7.39E-04
5152	ENSMUSG00000054555	Adam12	0.59	3.75E-04	7.45E-04
5153	ENSMUSG00000047379	B4gat1	-0.58	3.78E-04	7.50E-04
5154	ENSMUSG00000020262	Adarb1	-0.82	3.81E-04	7.56E-04
5155	ENSMUSG00000020393	Kremen1	-0.76	3.81E-04	7.56E-04
5156	ENSMUSG00000074733	Zfp950	0.89	3.82E-04	7.58E-04
5157	ENSMUSG00000026969	Fam166a	0.59	3.84E-04	7.61E-04
5158	ENSMUSG00000059586	Nsmce2	0.62	3.85E-04	7.63E-04
5159	ENSMUSG00000027593	Raly	0.74	3.85E-04	7.63E-04
5160	ENSMUSG00000069020	Urm1	0.78	3.85E-04	7.64E-04
5161	ENSMUSG00000021759	Plpp1	-0.66	3.87E-04	7.67E-04
5162	ENSMUSG00000002332	Dhrs1	0.51	3.87E-04	7.67E-04
5163	ENSMUSG00000085211	B430219N15Rik	0.52	3.88E-04	7.68E-04
5164	ENSMUSG00000024620	Pdgfrb	-1.00	3.88E-04	7.68E-04
5165	ENSMUSG00000025271	Pfkfb1	-0.66	3.90E-04	7.72E-04
5166	ENSMUSG00000025728	Pigq	0.50	3.91E-04	7.73E-04
5167	ENSMUSG00000027522	Stx16	-0.71	3.93E-04	7.78E-04
5168	ENSMUSG00000020773	Trim47	-0.77	3.93E-04	7.78E-04
5169	ENSMUSG00000061904	Slc25a3	0.51	3.95E-04	7.81E-04
5170	ENSMUSG00000031591	Asah1	-0.57	3.95E-04	7.82E-04
5171	ENSMUSG00000040990	Sh3kbp1	0.50	3.96E-04	7.82E-04
5172	ENSMUSG00000024048	Myl12a	-0.50	3.96E-04	7.84E-04
5173	ENSMUSG00000024925	Rnaseh2c	0.70	3.97E-04	7.84E-04
5174	ENSMUSG00000045210	Vcpip1	-0.72	3.97E-04	7.84E-04
5175	ENSMUSG00000041483	Zfp281	-0.80	3.97E-04	7.84E-04
5176	ENSMUSG00000058569	Tmed9	-0.69	3.98E-04	7.86E-04
5177	ENSMUSG00000109926	Gm45808	0.53	3.99E-04	7.89E-04
5178	ENSMUSG00000024120	Lrpprc	0.50	4.00E-04	7.89E-04
5179	ENSMUSG00000044475	Ascc1	0.59	4.02E-04	7.93E-04
5180	ENSMUSG00000044783	Hjurp	-0.78	4.02E-04	7.93E-04
5181	ENSMUSG00000050312	Nsun3	0.60	4.03E-04	7.96E-04
5182	ENSMUSG00000025935	Tram1	-0.54	4.05E-04	8.00E-04
5183	ENSMUSG00000027002	Nckap1	-0.60	4.05E-04	8.00E-04
5184	ENSMUSG00000086322	E130218I03Rik	0.86	4.07E-04	8.02E-04
5185	ENSMUSG00000019802	Sec63	-0.55	4.10E-04	8.08E-04
5186	ENSMUSG00000029390	Tmed2	0.50	4.10E-04	8.08E-04
5187	ENSMUSG00000003778	Brd8	-0.74	4.10E-04	8.09E-04
5188	ENSMUSG00000040699	Limd2	-0.84	4.13E-04	8.14E-04
5189	ENSMUSG00000031176	Dynlt3	0.49	4.13E-04	8.14E-04
5190	ENSMUSG00000038497	Tmco3	-0.61	4.15E-04	8.19E-04
5191	ENSMUSG00000033972	Zfp944	0.79	4.17E-04	8.21E-04
5192	ENSMUSG00000017421	Zfp207	-0.51	4.21E-04	8.30E-04
5193	ENSMUSG00000019731	Slc35e1	-0.58	4.22E-04	8.30E-04
5194	ENSMUSG00000002326	Gmpr2	0.62	4.23E-04	8.32E-04
5195	ENSMUSG00000037174	Elf2	0.69	4.25E-04	8.37E-04
5196	ENSMUSG00000074238	Ap1ar	-0.71	4.25E-04	8.37E-04
5197	ENSMUSG00000039770	Ypel5	-0.51	4.27E-04	8.40E-04

5198	ENSMUSG00000028608	0610037L13Rik	0.58	4.27E-04	8.41E-04
5199	ENSMUSG00000086839	Gm11973	0.76	4.29E-04	8.43E-04
5200	ENSMUSG00000037344	Slc12a9	-0.83	4.29E-04	8.43E-04
5201	ENSMUSG00000060216	Arrb2	-0.90	4.30E-04	8.45E-04
5202	ENSMUSG00000031527	Eri1	-0.84	4.30E-04	8.46E-04
5203	ENSMUSG00000040325	Vprbp	-0.72	4.30E-04	8.46E-04
5204	ENSMUSG00000090093	Gm14399	0.79	4.31E-04	8.46E-04
5205	ENSMUSG00000045534	Kcna5	-0.85	4.31E-04	8.48E-04
5206	ENSMUSG00000005312	Ubqln1	-0.55	4.32E-04	8.48E-04
5207	ENSMUSG00000029505	Ep400	-0.71	4.32E-04	8.49E-04
5208	ENSMUSG00000039427	Alg1	0.51	4.33E-04	8.51E-04
5209	ENSMUSG00000026558	Uck2	0.66	4.34E-04	8.51E-04
5210	ENSMUSG00000029478	Ncor2	-0.65	4.34E-04	8.52E-04
5211	ENSMUSG00000040918	Slc19a2	0.84	4.36E-04	8.56E-04
5212	ENSMUSG00000042389	Tsen2	-0.72	4.37E-04	8.57E-04
5213	ENSMUSG00000029208	Guf1	0.53	4.38E-04	8.59E-04
5214	ENSMUSG00000038024	Dennd4c	-0.75	4.38E-04	8.60E-04
5215	ENSMUSG00000049553	Polr1a	-0.70	4.39E-04	8.62E-04
5216	ENSMUSG00000034430	Zxdc	-0.80	4.41E-04	8.64E-04
5217	ENSMUSG00000029401	Rilpl2	0.55	4.41E-04	8.65E-04
5218	ENSMUSG00000033400	Agl	0.63	4.42E-04	8.66E-04
5219	ENSMUSG00000039068	Zzz3	-0.59	4.42E-04	8.67E-04
5220	ENSMUSG00000061959	Ces1e	-1.14	4.44E-04	8.70E-04
5221	ENSMUSG00000058325	Dock1	-0.68	4.47E-04	8.75E-04
5222	ENSMUSG00000043004	Gng2	-0.82	4.47E-04	8.75E-04
5223	ENSMUSG00000022718	Dgcr8	-0.86	4.48E-04	8.77E-04
5224	ENSMUSG00000059208	Hnrnpm	-0.51	4.49E-04	8.78E-04
5225	ENSMUSG00000045441	Gprin3	-0.87	4.49E-04	8.79E-04
5226	ENSMUSG00000110138	Gm45291	-0.83	4.51E-04	8.82E-04
5227	ENSMUSG00000066861	Oas1g	-0.89	4.54E-04	8.88E-04
5228	ENSMUSG00000004356	Utp20	-0.90	4.54E-04	8.89E-04
5229	ENSMUSG00000085105	Gm12758	-0.89	4.56E-04	8.92E-04
5230	ENSMUSG00000030435	U2af2	-0.64	4.59E-04	8.97E-04
5231	ENSMUSG00000043262	Uevld	-0.73	4.60E-04	8.98E-04
5232	ENSMUSG00000041974	Spidr	-0.80	4.60E-04	8.99E-04
5233	ENSMUSG00000025268	Maged2	-0.61	4.61E-04	9.00E-04
5234	ENSMUSG00000059689	Zfp637	0.56	4.61E-04	9.00E-04
5235	ENSMUSG00000021786	Oxsm	0.61	4.62E-04	9.03E-04
5236	ENSMUSG00000048911	Rnf24	-0.56	4.64E-04	9.05E-04
5237	ENSMUSG00000038736	Nudcd1	0.62	4.64E-04	9.07E-04
5238	ENSMUSG00000027597	Ahcy	0.64	4.66E-04	9.10E-04
5239	ENSMUSG00000040725	Hnrnpul1	-0.55	4.68E-04	9.12E-04
5240	ENSMUSG00000024776	Stambpl1	-0.78	4.68E-04	9.12E-04
5241	ENSMUSG00000040446	Rprd1a	0.79	4.69E-04	9.15E-04
5242	ENSMUSG00000021461	Fancc	-0.74	4.69E-04	9.15E-04
5243	ENSMUSG00000042579	4632404H12Rik	0.76	4.69E-04	9.15E-04
5244	ENSMUSG00000019087	Atp6ap1	-0.50	4.69E-04	9.15E-04
5245	ENSMUSG00000020109	Dnajb12	0.58	4.71E-04	9.17E-04
5246	ENSMUSG00000049957	Ccdc137	-0.72	4.71E-04	9.18E-04
5247	ENSMUSG00000024773	Atg2a	-0.55	4.74E-04	9.24E-04

5248	ENSMUSG00000030138	Bms1	-0.80	4.75E-04	9.25E-04
5249	ENSMUSG00000020170	Frs2	-0.69	4.76E-04	9.27E-04
5250	ENSMUSG00000097284	4930480K23Rik	0.84	4.77E-04	9.30E-04
5251	ENSMUSG00000039007	Cpq	-0.56	4.79E-04	9.33E-04
5252	ENSMUSG00000026096	Osgepl1	0.58	4.79E-04	9.33E-04
5253	ENSMUSG00000027722	Spata5	-0.85	4.80E-04	9.34E-04
5254	ENSMUSG00000052934	Fbxo31	0.49	4.81E-04	9.35E-04
5255	ENSMUSG00000044982	Sft2d3	0.75	4.81E-04	9.35E-04
5256	ENSMUSG00000032688	Malt1	-0.99	4.82E-04	9.38E-04
5257	ENSMUSG00000005687	Bcas2	0.63	4.90E-04	9.54E-04
5258	ENSMUSG00000014177	Tvp23b	0.57	4.94E-04	9.61E-04
5259	ENSMUSG00000045854	Lyrm2	0.81	4.95E-04	9.63E-04
5260	ENSMUSG00000055254	Ntrk2	-0.51	4.96E-04	9.64E-04
5261	ENSMUSG00000006998	Psmd2	-0.49	4.96E-04	9.64E-04
5262	ENSMUSG00000029228	Lnx1	-0.81	5.00E-04	9.72E-04
5263	ENSMUSG00000038696	Mapkap1	0.51	5.01E-04	9.73E-04
5264	ENSMUSG00000110711	Gm45760	0.83	5.04E-04	9.79E-04
5265	ENSMUSG00000027109	Sp3	-0.65	5.05E-04	9.81E-04
5266	ENSMUSG00000027108	Ola1	0.60	5.09E-04	9.87E-04
5267	ENSMUSG00000030084	Plxna1	-0.59	5.10E-04	9.91E-04
5268	ENSMUSG00000028410	Dnaja1	-0.51	5.12E-04	9.94E-04
5269	ENSMUSG00000022389	Tef	-0.66	5.12E-04	9.94E-04
5270	ENSMUSG00000054013	Tmem179	-0.94	5.12E-04	9.94E-04
5271	ENSMUSG00000030282	Cmas	-0.59	5.14E-04	9.98E-04
5272	ENSMUSG00000061410	Zcchc14	-0.79	5.16E-04	1.00E-03
5273	ENSMUSG00000037139	Myom3	-0.70	5.18E-04	1.01E-03
5274	ENSMUSG00000025583	Rptor	-0.59	5.18E-04	1.01E-03
5275	ENSMUSG00000034681	Rnps1	0.54	5.20E-04	1.01E-03
5276	ENSMUSG00000033763	Mtss1l	-0.80	5.23E-04	1.01E-03
5277	ENSMUSG00000055067	Smyd3	-0.81	5.23E-04	1.01E-03
5278	ENSMUSG00000022951	Rcan1	-0.63	5.24E-04	1.02E-03
5279	ENSMUSG00000001436	Slc19a1	-0.62	5.24E-04	1.02E-03
5280	ENSMUSG00000027678	Ncoa3	-0.79	5.25E-04	1.02E-03
5281	ENSMUSG00000025812	Pard3	-0.66	5.28E-04	1.02E-03
5282	ENSMUSG00000037788	Vopp1	0.80	5.30E-04	1.03E-03
5283	ENSMUSG00000022570	Tsta3	0.53	5.32E-04	1.03E-03
5284	ENSMUSG00000042207	Kdm5b	-0.74	5.34E-04	1.03E-03
5285	ENSMUSG00000026229	Psmd1	-0.51	5.35E-04	1.04E-03
5286	ENSMUSG00000047141	Zfp654	-0.61	5.37E-04	1.04E-03
5287	ENSMUSG00000097234	Gm26518	0.71	5.39E-04	1.04E-03
5288	ENSMUSG00000013033	Adgrl1	-0.83	5.39E-04	1.04E-03
5289	ENSMUSG00000023348	Trip6	-0.54	5.40E-04	1.04E-03
5290	ENSMUSG00000010554	Mettl16	-0.61	5.42E-04	1.05E-03
5291	ENSMUSG00000020409	Slu7	-0.58	5.42E-04	1.05E-03
5292	ENSMUSG00000028345	Tex10	-0.77	5.44E-04	1.05E-03
5293	ENSMUSG00000041935	AW549877	-0.61	5.45E-04	1.05E-03
5294	ENSMUSG00000030942	Thumpd1	-0.52	5.45E-04	1.05E-03
5295	ENSMUSG00000028899	Taf12	0.57	5.46E-04	1.05E-03
5296	ENSMUSG00000053846	Lipg	0.96	5.46E-04	1.05E-03
5297	ENSMUSG00000024665	Fads2	-1.06	5.47E-04	1.06E-03

5298	ENSMUSG00000021470	Ercc6l2	-0.71	5.48E-04	1.06E-03
5299	ENSMUSG00000026566	Mpzl1	-0.89	5.50E-04	1.06E-03
5300	ENSMUSG00000021189	Atxn3	-0.70	5.50E-04	1.06E-03
5301	ENSMUSG00000053411	Cbx7	-0.93	5.54E-04	1.07E-03
5302	ENSMUSG00000029759	Pon3	0.47	5.56E-04	1.07E-03
5303	ENSMUSG00000036840	Siah1a	0.61	5.57E-04	1.07E-03
5304	ENSMUSG00000005699	Pard6a	0.80	5.57E-04	1.07E-03
5305	ENSMUSG00000084799	Ino80dos	0.52	5.58E-04	1.08E-03
5306	ENSMUSG00000029270	Fam69a	-0.52	5.62E-04	1.08E-03
5307	ENSMUSG00000033166	Dis3	-0.80	5.63E-04	1.09E-03
5308	ENSMUSG00000038056	Kmt2c	-0.72	5.63E-04	1.09E-03
5309	ENSMUSG00000024854	Pold4	0.59	5.63E-04	1.09E-03
5310	ENSMUSG00000041650	Pcca	0.47	5.64E-04	1.09E-03
5311	ENSMUSG00000005610	Eif4g2	-0.52	5.64E-04	1.09E-03
5312	ENSMUSG00000026425	Srgap2	-0.80	5.64E-04	1.09E-03
5313	ENSMUSG00000027189	Trim44	-0.54	5.67E-04	1.09E-03
5314	ENSMUSG00000020088	Sar1a	-0.49	5.71E-04	1.10E-03
5315	ENSMUSG00000037857	Nufip2	-0.74	5.72E-04	1.10E-03
5316	ENSMUSG00000025332	Kdm5c	-0.59	5.73E-04	1.10E-03
5317	ENSMUSG00000028973	Abcb8	0.48	5.74E-04	1.10E-03
5318	ENSMUSG00000023959	Clic5	0.69	5.76E-04	1.11E-03
5319	ENSMUSG00000037278	Tmem97	0.60	5.77E-04	1.11E-03
5320	ENSMUSG00000020707	Rnf135	-0.76	5.78E-04	1.11E-03
5321	ENSMUSG00000092310	Gm20509	0.78	5.78E-04	1.11E-03
5322	ENSMUSG00000079084	Ccdc82	-0.81	5.79E-04	1.11E-03
5323	ENSMUSG00000028675	Pnrc2	0.50	5.80E-04	1.11E-03
5324	ENSMUSG00000086679	Gm15551	-0.73	5.83E-04	1.12E-03
5325	ENSMUSG00000026584	Scyl3	-0.85	5.83E-04	1.12E-03
5326	ENSMUSG00000018189	Uchl5	0.52	5.89E-04	1.13E-03
5327	ENSMUSG00000060288	Ppih	0.80	5.90E-04	1.13E-03
5328	ENSMUSG00000025950	Idh1	-0.51	5.90E-04	1.13E-03
5329	ENSMUSG00000048661	Lemd3	-0.85	5.93E-04	1.14E-03
5330	ENSMUSG00000022843	Clcn2	-1.10	5.94E-04	1.14E-03
5331	ENSMUSG00000021368	Tbc1d7	0.54	5.96E-04	1.14E-03
5332	ENSMUSG00000050244	Heatr1	-0.63	6.00E-04	1.15E-03
5333	ENSMUSG00000057176	Ccdc189	0.60	6.01E-04	1.15E-03
5334	ENSMUSG00000085425	Gm15648	0.49	6.01E-04	1.15E-03
5335	ENSMUSG00000028688	Toe1	-0.71	6.01E-04	1.15E-03
5336	ENSMUSG00000061979	Rcc1l	0.57	6.02E-04	1.15E-03
5337	ENSMUSG00000063281	Zfp35	0.71	6.02E-04	1.15E-03
5338	ENSMUSG00000038005	Hpf1	0.77	6.04E-04	1.16E-03
5339	ENSMUSG00000105647	Gm43695	0.56	6.06E-04	1.16E-03
5340	ENSMUSG00000097411	B430218F22Rik	0.54	6.07E-04	1.16E-03
5341	ENSMUSG00000027665	Pik3ca	-0.58	6.07E-04	1.16E-03
5342	ENSMUSG00000019790	Stxbp5	-0.77	6.07E-04	1.16E-03
5343	ENSMUSG00000020521	Rnft1	0.58	6.09E-04	1.17E-03
5344	ENSMUSG00000021022	Ppp2r3c	0.61	6.10E-04	1.17E-03
5345	ENSMUSG00000068823	Csde1	0.51	6.10E-04	1.17E-03
5346	ENSMUSG00000039901	9130011E15Rik	-0.69	6.11E-04	1.17E-03
5347	ENSMUSG00000058388	Phtf1	-0.73	6.11E-04	1.17E-03

5348	ENSMUSG00000031214	Ophn1	-0.69	6.12E-04	1.17E-03
5349	ENSMUSG00000097680	Gm26642	-0.65	6.13E-04	1.17E-03
5350	ENSMUSG00000050310	Rictor	-0.70	6.14E-04	1.17E-03
5351	ENSMUSG00000042628	Zfyve1	-0.83	6.15E-04	1.18E-03
5352	ENSMUSG00000039308	Ndst2	-0.78	6.16E-04	1.18E-03
5353	ENSMUSG00000047648	Fbxo30	-0.70	6.17E-04	1.18E-03
5354	ENSMUSG00000037295	Ldlrap1	0.56	6.18E-04	1.18E-03
5355	ENSMUSG00000034544	Rsrc1	-0.61	6.19E-04	1.18E-03
5356	ENSMUSG00000032342	Mto1	0.62	6.20E-04	1.18E-03
5357	ENSMUSG00000074627	Mroh8	-0.76	6.23E-04	1.19E-03
5358	ENSMUSG00000024614	Tmx3	-0.67	6.24E-04	1.19E-03
5359	ENSMUSG00000015837	Sqstm1	-0.51	6.27E-04	1.20E-03
5360	ENSMUSG00000039382	Wdr45	0.53	6.27E-04	1.20E-03
5361	ENSMUSG00000006024	Napa	0.47	6.28E-04	1.20E-03
5362	ENSMUSG00000092137	Gcom1	0.48	6.28E-04	1.20E-03
5363	ENSMUSG00000029169	Dhx15	-0.52	6.29E-04	1.20E-03
5364	ENSMUSG00000000134	Tfe3	-0.64	6.29E-04	1.20E-03
5365	ENSMUSG00000058317	Ube2e2	-0.57	6.32E-04	1.21E-03
5366	ENSMUSG00000078908	Mon1b	-0.63	6.37E-04	1.21E-03
5367	ENSMUSG00000049739	Zfp646	-0.77	6.39E-04	1.22E-03
5368	ENSMUSG00000105552	Gm19710	-0.75	6.41E-04	1.22E-03
5369	ENSMUSG00000023010	Tmbim6	0.47	6.43E-04	1.22E-03
5370	ENSMUSG00000029684	Wasl	-0.53	6.45E-04	1.23E-03
5371	ENSMUSG00000062127	Cttnbp2nl	-0.86	6.46E-04	1.23E-03
5372	ENSMUSG00000037031	Tspan15	0.49	6.47E-04	1.23E-03
5373	ENSMUSG00000002957	Ap2a2	-0.48	6.49E-04	1.23E-03
5374	ENSMUSG00000109168	Gm44709	-0.62	6.50E-04	1.24E-03
5375	ENSMUSG00000002812	Flii	-0.48	6.51E-04	1.24E-03
5376	ENSMUSG00000029263	Pigg	-0.69	6.52E-04	1.24E-03
5377	ENSMUSG00000030822	Prr14	-0.63	6.53E-04	1.24E-03
5378	ENSMUSG00000078877	Gm14295	0.71	6.57E-04	1.25E-03
5379	ENSMUSG00000024451	Arap3	-0.74	6.57E-04	1.25E-03
5380	ENSMUSG00000048200	Cracr2b	-0.79	6.60E-04	1.26E-03
5381	ENSMUSG00000028343	Erp44	0.53	6.62E-04	1.26E-03
5382	ENSMUSG00000021134	Srsf5	-0.53	6.62E-04	1.26E-03
5383	ENSMUSG00000032946	Rasgrp2	-0.75	6.63E-04	1.26E-03
5384	ENSMUSG00000029386	Tctn2	-0.79	6.64E-04	1.26E-03
5385	ENSMUSG00000005893	Nr2c2	-0.74	6.65E-04	1.26E-03
5386	ENSMUSG00000027194	Ttc17	-0.65	6.66E-04	1.26E-03
5387	ENSMUSG00000000751	Rpa1	-0.62	6.66E-04	1.26E-03
5388	ENSMUSG00000041702	Btbd7	-0.78	6.68E-04	1.27E-03
5389	ENSMUSG00000022881	Rfc4	0.77	6.69E-04	1.27E-03
5390	ENSMUSG00000051346	Spryd4	0.66	6.72E-04	1.28E-03
5391	ENSMUSG00000028945	Rheb	0.47	6.74E-04	1.28E-03
5392	ENSMUSG00000039737	Prkrip1	0.80	6.75E-04	1.28E-03
5393	ENSMUSG00000032712	2810474O19Rik	0.66	6.75E-04	1.28E-03
5394	ENSMUSG00000026374	Tsn	0.48	6.77E-04	1.28E-03
5395	ENSMUSG00000015149	Sirt2	0.47	6.77E-04	1.28E-03
5396	ENSMUSG00000020782	Llgl2	-0.73	6.78E-04	1.28E-03
5397	ENSMUSG00000053119	Chmp3	0.49	6.78E-04	1.28E-03

5398	ENSMUSG00000024556	Me2	-0.66	6.81E-04	1.29E-03
5399	ENSMUSG00000026918	Brd3	0.68	6.82E-04	1.29E-03
5400	ENSMUSG00000026893	Gca	-0.73	6.83E-04	1.29E-03
5401	ENSMUSG00000045294	Insig1	-0.60	6.83E-04	1.29E-03
5402	ENSMUSG00000034216	Vps18	-0.67	6.83E-04	1.29E-03
5403	ENSMUSG00000028854	Slc9a1	-0.73	6.84E-04	1.29E-03
5404	ENSMUSG00000020166	Cnot2	-0.57	6.84E-04	1.29E-03
5405	ENSMUSG00000051518	Rps19bp1	0.64	6.84E-04	1.29E-03
5406	ENSMUSG00000031858	Mau2	-0.59	6.84E-04	1.29E-03
5407	ENSMUSG00000047554	Tmem41b	0.57	6.87E-04	1.30E-03
5408	ENSMUSG00000039512	Uhrf1bp1	-0.67	6.87E-04	1.30E-03
5409	ENSMUSG00000048175	Asb8	0.49	6.88E-04	1.30E-03
5410	ENSMUSG00000109129	Gm44973	0.65	6.91E-04	1.31E-03
5411	ENSMUSG00000089902	Gm13625	0.77	6.93E-04	1.31E-03
5412	ENSMUSG00000043962	Thrap3	-0.60	6.94E-04	1.31E-03
5413	ENSMUSG00000057637	Prdm2	-0.83	6.95E-04	1.31E-03
5414	ENSMUSG00000102299	Gm37539	-0.72	6.97E-04	1.32E-03
5415	ENSMUSG00000062232	Rapgef2	-0.78	6.99E-04	1.32E-03
5416	ENSMUSG00000020770	Unk	-0.77	7.01E-04	1.32E-03
5417	ENSMUSG00000032290	Ptpn9	-0.65	7.01E-04	1.32E-03
5418	ENSMUSG00000097472	Gm26586	-0.73	7.02E-04	1.32E-03
5419	ENSMUSG00000061665	Cd2ap	-0.70	7.06E-04	1.33E-03
5420	ENSMUSG00000015733	Capza2	0.47	7.10E-04	1.34E-03
5421	ENSMUSG00000086382	Chrna1os	0.83	7.12E-04	1.34E-03
5422	ENSMUSG00000029924	Slc37a3	-0.70	7.12E-04	1.34E-03
5423	ENSMUSG00000054263	Lifr	-0.67	7.14E-04	1.35E-03
5424	ENSMUSG00000024610	Cd74	-1.07	7.18E-04	1.35E-03
5425	ENSMUSG00000054455	Vapb	0.50	7.18E-04	1.35E-03
5426	ENSMUSG00000050428	Fbxo46	-0.75	7.19E-04	1.36E-03
5427	ENSMUSG00000004565	Pnpla6	-0.61	7.20E-04	1.36E-03
5428	ENSMUSG00000021519	Mterf3	0.58	7.23E-04	1.36E-03
5429	ENSMUSG00000073775	Kti12	0.55	7.29E-04	1.37E-03
5430	ENSMUSG00000033285	Wdr3	-0.83	7.31E-04	1.38E-03
5431	ENSMUSG00000039983	Ccdc32	0.61	7.31E-04	1.38E-03
5432	ENSMUSG00000040599	Mis12	-0.70	7.36E-04	1.39E-03
5433	ENSMUSG00000025487	Psmd13	0.49	7.36E-04	1.39E-03
5434	ENSMUSG00000001017	Chtop	-0.50	7.38E-04	1.39E-03
5435	ENSMUSG00000022426	Josd1	0.49	7.38E-04	1.39E-03
5436	ENSMUSG00000025269	Apex2	-0.63	7.40E-04	1.39E-03
5437	ENSMUSG00000034911	Ushbp1	-0.76	7.40E-04	1.39E-03
5438	ENSMUSG00000042229	Rabif	0.49	7.41E-04	1.39E-03
5439	ENSMUSG00000032324	Tspan3	0.47	7.43E-04	1.40E-03
5440	ENSMUSG00000030276	Ttll3	0.52	7.44E-04	1.40E-03
5441	ENSMUSG00000086968	4933431E20Rik	0.59	7.44E-04	1.40E-03
5442	ENSMUSG00000020561	Twistnb	0.63	7.49E-04	1.41E-03
5443	ENSMUSG00000053291	Rab4b	-0.58	7.50E-04	1.41E-03
5444	ENSMUSG00000029992	Gfpt1	-0.59	7.56E-04	1.42E-03
5445	ENSMUSG00000046985	Tapt1	-0.70	7.58E-04	1.42E-03
5446	ENSMUSG00000089872	Rps6kc1	-0.76	7.58E-04	1.42E-03
5447	ENSMUSG00000034951	Cog7	-0.49	7.62E-04	1.43E-03

5448	ENSMUSG00000023921	Mut	-0.45	7.63E-04	1.43E-03
5449	ENSMUSG00000022564	Grina	-0.46	7.65E-04	1.44E-03
5450	ENSMUSG00000062627	Mysm1	-0.82	7.65E-04	1.44E-03
5451	ENSMUSG00000015776	Med22	-0.57	7.68E-04	1.44E-03
5452	ENSMUSG00000048142	Nat8l	0.48	7.69E-04	1.44E-03
5453	ENSMUSG00000107406	1700040L08Rik	0.75	7.71E-04	1.45E-03
5454	ENSMUSG00000015363	Trabd	0.49	7.73E-04	1.45E-03
5455	ENSMUSG00000006262	Mob1b	0.54	7.75E-04	1.45E-03
5456	ENSMUSG00000048058	Ldlrad3	-0.68	7.76E-04	1.45E-03
5457	ENSMUSG00000044573	Acp1	0.48	7.77E-04	1.46E-03
5458	ENSMUSG00000058207	Serpina3k	-1.12	7.78E-04	1.46E-03
5459	ENSMUSG00000042286	Stab1	-0.89	7.79E-04	1.46E-03
5460	ENSMUSG00000054766	Set	0.49	7.81E-04	1.46E-03
5461	ENSMUSG00000023026	Dip2b	-0.71	7.81E-04	1.46E-03
5462	ENSMUSG00000021494	Ddx41	0.52	7.82E-04	1.46E-03
5463	ENSMUSG00000023044	Csad	-0.49	7.82E-04	1.46E-03
5464	ENSMUSG00000002020	Ltbp2	0.49	7.82E-04	1.46E-03
5465	ENSMUSG00000029623	Pdap1	0.60	7.83E-04	1.46E-03
5466	ENSMUSG00000061024	Rrs1	-0.60	7.85E-04	1.47E-03
5467	ENSMUSG00000034042	Gpbp1l1	0.59	7.85E-04	1.47E-03
5468	ENSMUSG00000027712	Anxa5	0.47	7.86E-04	1.47E-03
5469	ENSMUSG00000026687	Aldh9a1	0.46	7.87E-04	1.47E-03
5470	ENSMUSG00000030697	Ppp4c	0.50	7.92E-04	1.48E-03
5471	ENSMUSG00000030055	Rab43	-0.74	7.95E-04	1.49E-03
5472	ENSMUSG00000024091	Vapa	0.48	7.98E-04	1.49E-03
5473	ENSMUSG00000021629	Slc30a5	-0.55	7.98E-04	1.49E-03
5474	ENSMUSG00000035310	Lin54	-0.81	7.98E-04	1.49E-03
5475	ENSMUSG00000019794	Katna1	0.59	7.99E-04	1.49E-03
5476	ENSMUSG00000027833	Shox2	-0.76	8.01E-04	1.50E-03
5477	ENSMUSG00000026409	Pfkfb2	-0.72	8.01E-04	1.50E-03
5478	ENSMUSG00000006932	Ctnnb1	-0.47	8.03E-04	1.50E-03
5479	ENSMUSG00000001999	Blvra	0.52	8.04E-04	1.50E-03
5480	ENSMUSG00000041959	S100a10	0.51	8.04E-04	1.50E-03
5481	ENSMUSG00000037656	Slc20a2	-0.52	8.05E-04	1.50E-03
5482	ENSMUSG00000021993	Mipep	0.50	8.07E-04	1.51E-03
5483	ENSMUSG00000002910	Arrdc2	-0.86	8.12E-04	1.51E-03
5484	ENSMUSG00000020591	Ntsr2	0.70	8.15E-04	1.52E-03
5485	ENSMUSG00000051255	Gm6563	0.71	8.16E-04	1.52E-03
5486	ENSMUSG00000032598	Nckipsd	-0.60	8.17E-04	1.52E-03
5487	ENSMUSG00000111497	Raver1	0.49	8.19E-04	1.53E-03
5488	ENSMUSG00000026496	Parp1	0.52	8.19E-04	1.53E-03
5489	ENSMUSG00000097170	Gm16982	-0.66	8.20E-04	1.53E-03
5490	ENSMUSG00000005043	Sgsh	-0.66	8.25E-04	1.54E-03
5491	ENSMUSG00000005732	Ranbp1	0.52	8.26E-04	1.54E-03
5492	ENSMUSG00000036968	Cnpy4	-0.77	8.29E-04	1.54E-03
5493	ENSMUSG00000030614	Tmem126b	0.50	8.30E-04	1.54E-03
5494	ENSMUSG00000032513	Gorasp1	-0.50	8.33E-04	1.55E-03
5495	ENSMUSG00000010476	Ebf3	0.63	8.34E-04	1.55E-03
5496	ENSMUSG00000030835	Nomo1	-0.59	8.34E-04	1.55E-03
5497	ENSMUSG00000048249	Crebrf	-0.77	8.40E-04	1.56E-03

5498	ENSMUSG00000049657	Zbtb5	-0.72	8.40E-04	1.56E-03
5499	ENSMUSG00000018425	Dhx40	-0.64	8.42E-04	1.57E-03
5500	ENSMUSG00000017386	Traf4	0.54	8.43E-04	1.57E-03
5501	ENSMUSG00000036181	Hist1h1c	0.59	8.44E-04	1.57E-03
5502	ENSMUSG00000036529	Sbf1	-0.52	8.44E-04	1.57E-03
5503	ENSMUSG00000024800	Rpp30	0.76	8.44E-04	1.57E-03
5504	ENSMUSG00000030872	Gga2	-0.53	8.46E-04	1.57E-03
5505	ENSMUSG00000098912	1500004A13Rik	-0.74	8.47E-04	1.57E-03
5506	ENSMUSG00000107969	Gm44085	-0.79	8.49E-04	1.58E-03
5507	ENSMUSG00000071042	Rasgrp3	-0.76	8.50E-04	1.58E-03
5508	ENSMUSG00000041977	Arhgef11	0.50	8.50E-04	1.58E-03
5509	ENSMUSG00000003037	Rab8a	0.48	8.50E-04	1.58E-03
5510	ENSMUSG00000021589	Rhobtb3	-0.78	8.52E-04	1.58E-03
5511	ENSMUSG00000019857	Asf1a	0.67	8.54E-04	1.58E-03
5512	ENSMUSG00000025747	Tyms	0.77	8.55E-04	1.59E-03
5513	ENSMUSG00000013495	Tmem175	-0.56	8.57E-04	1.59E-03
5514	ENSMUSG00000030551	Nr2f2	-0.78	8.65E-04	1.61E-03
5515	ENSMUSG00000024127	Prepl	-0.71	8.67E-04	1.61E-03
5516	ENSMUSG00000006906	Stambp	-0.58	8.67E-04	1.61E-03
5517	ENSMUSG00000097032	4930539J05Rik	0.76	8.69E-04	1.61E-03
5518	ENSMUSG00000021687	Scamp1	-0.52	8.70E-04	1.61E-03
5519	ENSMUSG00000068079	Tcf15	-0.76	8.72E-04	1.62E-03
5520	ENSMUSG00000026816	Gtf3c5	-0.72	8.72E-04	1.62E-03
5521	ENSMUSG00000089762	Ier5l	-0.74	8.73E-04	1.62E-03
5522	ENSMUSG00000084781	D930015M05Rik	-0.79	8.76E-04	1.62E-03
5523	ENSMUSG00000025646	Atrip	-0.68	8.76E-04	1.62E-03
5524	ENSMUSG00000036054	Sugp2	-0.79	8.83E-04	1.64E-03
5525	ENSMUSG00000015501	Hivep2	-0.76	8.85E-04	1.64E-03
5526	ENSMUSG00000078676	Casc3	-0.62	8.87E-04	1.64E-03
5527	ENSMUSG00000033883	D3Ertd254e	-0.80	8.88E-04	1.64E-03
5528	ENSMUSG00000060279	Ap2a1	-0.49	8.91E-04	1.65E-03
5529	ENSMUSG00000053580	Tanc2	-0.79	8.92E-04	1.65E-03
5530	ENSMUSG00000039067	Psmd7	0.48	8.93E-04	1.65E-03
5531	ENSMUSG00000058690	Ccser2	-0.49	8.94E-04	1.65E-03
5532	ENSMUSG00000041685	Fcho2	-0.59	8.94E-04	1.65E-03
5533	ENSMUSG00000041343	Ankrd42	0.71	8.98E-04	1.66E-03
5534	ENSMUSG00000030105	Arl8b	0.46	9.01E-04	1.67E-03
5535	ENSMUSG00000038482	Tfdp1	-0.52	9.06E-04	1.67E-03
5536	ENSMUSG00000060860	Ube2s	0.61	9.09E-04	1.68E-03
5537	ENSMUSG00000022579	Gpihbp1	-0.58	9.11E-04	1.68E-03
5538	ENSMUSG00000056144	Trim34a	-0.86	9.14E-04	1.69E-03
5539	ENSMUSG00000039967	Zfp292	-0.76	9.15E-04	1.69E-03
5540	ENSMUSG00000028933	Xrcc2	-0.76	9.17E-04	1.69E-03
5541	ENSMUSG00000063954	Hist2h2aa2	0.54	9.17E-04	1.69E-03
5542	ENSMUSG00000015247	Nipsnap3b	-0.52	9.19E-04	1.70E-03
5543	ENSMUSG00000013622	Atraid	0.49	9.19E-04	1.70E-03
5544	ENSMUSG00000087177	E130307A14Rik	-0.87	9.20E-04	1.70E-03
5545	ENSMUSG00000113209	CT030142.1	0.64	9.34E-04	1.72E-03
5546	ENSMUSG00000028431	Ikbkap	-0.78	9.36E-04	1.73E-03
5547	ENSMUSG00000026782	Abi2	-0.82	9.39E-04	1.73E-03

5548	ENSMUSG00000036202	Rif1	-0.79	9.41E-04	1.74E-03
5549	ENSMUSG00000028898	Trnau1ap	0.50	9.47E-04	1.75E-03
5550	ENSMUSG00000026761	Orc4	0.56	9.47E-04	1.75E-03
5551	ENSMUSG00000086237	Gm15591	0.78	9.48E-04	1.75E-03
5552	ENSMUSG00000024083	Pja2	-0.49	9.48E-04	1.75E-03
5553	ENSMUSG00000095538	Gm21983	-0.56	9.54E-04	1.76E-03
5554	ENSMUSG00000086561	Gm15540	0.66	9.54E-04	1.76E-03
5555	ENSMUSG00000096993	Gm26787	0.65	9.55E-04	1.76E-03
5556	ENSMUSG00000028840	Zfp593	0.81	9.55E-04	1.76E-03
5557	ENSMUSG00000057133	Chd6	-0.73	9.56E-04	1.76E-03
5558	ENSMUSG00000025880	Smad7	-0.83	9.56E-04	1.76E-03
5559	ENSMUSG00000041891	Lman1	0.50	9.57E-04	1.76E-03
5560	ENSMUSG00000039850	Endov	-0.65	9.61E-04	1.77E-03
5561	ENSMUSG00000025538	Sumf2	-0.54	9.64E-04	1.77E-03
5562	ENSMUSG00000020859	Spag9	-0.51	9.67E-04	1.78E-03
5563	ENSMUSG00000022022	Mtrf1	0.62	9.74E-04	1.79E-03
5564	ENSMUSG00000069520	Tmem19	-0.53	9.79E-04	1.80E-03
5565	ENSMUSG00000089875	Etohd2	0.72	9.81E-04	1.80E-03
5566	ENSMUSG00000046556	Zfp319	-0.76	9.82E-04	1.80E-03
5567	ENSMUSG00000087026	A230103J11Rik	-0.73	9.84E-04	1.81E-03
5568	ENSMUSG00000024787	Snx15	0.66	9.86E-04	1.81E-03
5569	ENSMUSG00000028879	Stx12	0.47	9.88E-04	1.81E-03
5570	ENSMUSG00000028141	Oaz3	0.70	9.91E-04	1.82E-03
5571	ENSMUSG00000020720	Psmd12	0.45	9.91E-04	1.82E-03
5572	ENSMUSG00000046876	Atxn1	-0.74	9.94E-04	1.82E-03
5573	ENSMUSG00000022191	Drosha	-0.59	9.97E-04	1.83E-03
5574	ENSMUSG00000036155	Mgat5	-0.86	9.98E-04	1.83E-03
5575	ENSMUSG00000047221	Fam185a	0.70	1.00E-03	1.83E-03
5576	ENSMUSG00000005150	Wdr83	0.48	1.00E-03	1.83E-03
5577	ENSMUSG00000041035	Gm17018	0.54	1.00E-03	1.84E-03
5578	ENSMUSG00000042116	Vwa1	-0.80	1.00E-03	1.84E-03
5579	ENSMUSG00000024283	Wac	-0.53	1.00E-03	1.84E-03
5580	ENSMUSG00000037251	Pomk	-0.65	1.00E-03	1.84E-03
5581	ENSMUSG00000020085	Aifm2	0.45	1.01E-03	1.84E-03
5582	ENSMUSG00000043991	Pura	0.58	1.02E-03	1.86E-03
5583	ENSMUSG00000057778	Cyb5d2	0.55	1.02E-03	1.87E-03
5584	ENSMUSG00000026319	2310035C23Rik	-0.66	1.02E-03	1.87E-03
5585	ENSMUSG00000029415	Sdad1	-0.68	1.02E-03	1.87E-03
5586	ENSMUSG00000024410	3110002H16Rik	-0.50	1.02E-03	1.87E-03
5587	ENSMUSG00000097221	1810049J17Rik	0.68	1.02E-03	1.87E-03
5588	ENSMUSG00000038517	Tbkbp1	0.60	1.02E-03	1.88E-03
5589	ENSMUSG00000026618	Iars2	-0.46	1.03E-03	1.88E-03
5590	ENSMUSG00000046668	Cxxc5	-0.63	1.03E-03	1.88E-03
5591	ENSMUSG00000028413	B4galt1	-0.66	1.03E-03	1.88E-03
5592	ENSMUSG00000036333	Kidins220	-0.55	1.04E-03	1.90E-03
5593	ENSMUSG00000041528	Rnf123	-0.48	1.04E-03	1.90E-03
5594	ENSMUSG00000053253	Ndfip2	0.51	1.04E-03	1.91E-03
5595	ENSMUSG00000037826	Ppm1k	0.71	1.05E-03	1.91E-03
5596	ENSMUSG00000022867	Usp25	-0.50	1.05E-03	1.91E-03
5597	ENSMUSG00000024050	Wiz	-0.64	1.05E-03	1.91E-03

5598	ENSMUSG00000001750	Tcirg1	-0.69	1.05E-03	1.92E-03
5599	ENSMUSG00000038250	Usp38	-0.57	1.05E-03	1.92E-03
5600	ENSMUSG00000020864	Ankrd40	0.45	1.06E-03	1.93E-03
5601	ENSMUSG00000027799	Nbea	-0.74	1.06E-03	1.93E-03
5602	ENSMUSG00000059182	Skap2	0.50	1.06E-03	1.93E-03
5603	ENSMUSG00000052155	Acvr2a	-0.61	1.06E-03	1.93E-03
5604	ENSMUSG00000029472	Anapc5	-0.44	1.06E-03	1.93E-03
5605	ENSMUSG00000039231	Suv39h1	-0.82	1.06E-03	1.93E-03
5606	ENSMUSG00000040506	Ambra1	-0.72	1.06E-03	1.93E-03
5607	ENSMUSG00000048706	Lurap1l	-0.84	1.06E-03	1.94E-03
5608	ENSMUSG00000024847	Aip	0.47	1.06E-03	1.94E-03
5609	ENSMUSG00000024191	Bnip1	0.82	1.07E-03	1.94E-03
5610	ENSMUSG00000050148	Ubqln2	-0.71	1.07E-03	1.94E-03
5611	ENSMUSG00000033107	Rnf125	-0.51	1.07E-03	1.94E-03
5612	ENSMUSG00000027422	Rrbp1	-0.52	1.07E-03	1.95E-03
5613	ENSMUSG00000026869	Psmd5	-0.50	1.07E-03	1.95E-03
5614	ENSMUSG00000035372	1810055G02Rik	-0.67	1.07E-03	1.95E-03
5615	ENSMUSG00000039018	Mtg1	0.45	1.07E-03	1.96E-03
5616	ENSMUSG00000007458	M6pr	0.44	1.08E-03	1.96E-03
5617	ENSMUSG00000054408	Spcs3	-0.68	1.08E-03	1.97E-03
5618	ENSMUSG00000067787	Blcap	-0.50	1.08E-03	1.97E-03
5619	ENSMUSG00000039089	L3mbtl3	-0.79	1.09E-03	1.98E-03
5620	ENSMUSG00000027613	Eif6	0.45	1.09E-03	1.98E-03
5621	ENSMUSG00000009739	Pou6f1	-0.70	1.09E-03	1.98E-03
5622	ENSMUSG00000001288	Rarg	-0.87	1.09E-03	1.99E-03
5623	ENSMUSG00000047123	Ticam1	0.72	1.09E-03	1.99E-03
5624	ENSMUSG00000020328	Nudcd2	0.58	1.09E-03	1.99E-03
5625	ENSMUSG00000109336	Samd4b	-0.52	1.11E-03	2.01E-03
5626	ENSMUSG00000056724	Nbeal2	-0.78	1.11E-03	2.02E-03
5627	ENSMUSG00000030102	Itpr1	0.60	1.11E-03	2.02E-03
5628	ENSMUSG00000101970	1810026B05Rik	0.74	1.11E-03	2.02E-03
5629	ENSMUSG00000029578	Wipi2	0.46	1.12E-03	2.03E-03
5630	ENSMUSG00000039046	Usp6nl	-0.76	1.12E-03	2.03E-03
5631	ENSMUSG00000045427	Hnrnph2	-0.45	1.12E-03	2.03E-03
5632	ENSMUSG00000026694	Mettl13	-0.71	1.12E-03	2.04E-03
5633	ENSMUSG00000033392	Clasp2	-0.64	1.13E-03	2.04E-03
5634	ENSMUSG00000031828	Klhl36	-0.75	1.13E-03	2.05E-03
5635	ENSMUSG00000020918	Kat2a	-0.64	1.13E-03	2.06E-03
5636	ENSMUSG00000031021	Tmem9b	0.46	1.13E-03	2.06E-03
5637	ENSMUSG00000034659	Tmem109	-0.46	1.14E-03	2.07E-03
5638	ENSMUSG00000029310	Nudt9	0.45	1.15E-03	2.08E-03
5639	ENSMUSG00000048799	Cep120	-0.69	1.15E-03	2.09E-03
5640	ENSMUSG00000020196	Cabin1	-0.67	1.15E-03	2.09E-03
5641	ENSMUSG00000022329	Stk3	-0.60	1.16E-03	2.10E-03
5642	ENSMUSG00000026024	Als2	-0.69	1.16E-03	2.10E-03
5643	ENSMUSG00000031166	Wdr13	-0.53	1.16E-03	2.10E-03
5644	ENSMUSG00000026088	Mitd1	0.71	1.16E-03	2.10E-03
5645	ENSMUSG00000068855	Hist2h2ac	0.53	1.16E-03	2.11E-03
5646	ENSMUSG00000093577	Gm20632	0.53	1.17E-03	2.11E-03
5647	ENSMUSG00000041236	Vps41	-0.50	1.17E-03	2.12E-03

5648	ENSMUSG00000005951	Shpk	-0.50	1.17E-03	2.12E-03
5649	ENSMUSG00000010205	Raver1	-0.62	1.17E-03	2.13E-03
5650	ENSMUSG00000018378	Cuedc1	-0.62	1.18E-03	2.13E-03
5651	ENSMUSG00000029076	Sdf4	-0.44	1.18E-03	2.13E-03
5652	ENSMUSG00000049764	Zfp280b	-0.73	1.18E-03	2.13E-03
5653	ENSMUSG00000003873	Bax	0.63	1.19E-03	2.16E-03
5654	ENSMUSG00000002372	Ranbp3	-0.56	1.19E-03	2.16E-03
5655	ENSMUSG00000049327	Kmt5a	0.47	1.20E-03	2.16E-03
5656	ENSMUSG00000033237	Arid2	-0.77	1.20E-03	2.16E-03
5657	ENSMUSG00000029387	Gtf2h3	0.50	1.20E-03	2.17E-03
5658	ENSMUSG00000073176	Zfp449	-0.72	1.20E-03	2.18E-03
5659	ENSMUSG00000055835	Zfp1	0.71	1.21E-03	2.18E-03
5660	ENSMUSG00000045795	Whamm	-0.64	1.21E-03	2.19E-03
5661	ENSMUSG00000021418	Rpp40	0.68	1.21E-03	2.19E-03
5662	ENSMUSG00000014905	Dnajb9	0.51	1.21E-03	2.19E-03
5663	ENSMUSG00000026878	Rab14	0.43	1.22E-03	2.20E-03
5664	ENSMUSG00000015966	Il17rb	-0.75	1.22E-03	2.20E-03
5665	ENSMUSG00000041548	Hspb8	0.48	1.23E-03	2.22E-03
5666	ENSMUSG00000022114	Spry2	-0.80	1.23E-03	2.23E-03
5667	ENSMUSG00000037640	Zfp60	0.68	1.23E-03	2.23E-03
5668	ENSMUSG00000013663	Pten	-0.53	1.24E-03	2.23E-03
5669	ENSMUSG00000107999	Gm44123	-0.65	1.24E-03	2.23E-03
5670	ENSMUSG00000097325	Gm16897	-0.80	1.24E-03	2.24E-03
5671	ENSMUSG00000008333	Snrpb2	0.68	1.25E-03	2.25E-03
5672	ENSMUSG00000001173	Ocrl	-0.78	1.25E-03	2.25E-03
5673	ENSMUSG00000028832	Stmn1	-0.78	1.25E-03	2.26E-03
5674	ENSMUSG00000034863	Ano8	-0.61	1.25E-03	2.26E-03
5675	ENSMUSG00000025085	Ablim1	-0.47	1.26E-03	2.26E-03
5676	ENSMUSG00000045576	St7l	0.53	1.26E-03	2.27E-03
5677	ENSMUSG00000019975	Ikbip	-0.70	1.26E-03	2.27E-03
5678	ENSMUSG00000031357	Syap1	-0.43	1.26E-03	2.27E-03
5679	ENSMUSG00000021846	Peli2	-0.52	1.26E-03	2.27E-03
5680	ENSMUSG00000042073	Abhd14b	-0.48	1.27E-03	2.28E-03
5681	ENSMUSG00000078921	Tgtp2	-0.73	1.27E-03	2.29E-03
5682	ENSMUSG00000029669	Tspan12	0.44	1.28E-03	2.30E-03
5683	ENSMUSG00000029863	Casp2	-0.75	1.28E-03	2.30E-03
5684	ENSMUSG00000011096	Akt1s1	0.48	1.28E-03	2.30E-03
5685	ENSMUSG00000053128	Rnf26	-0.61	1.28E-03	2.30E-03
5686	ENSMUSG00000021635	Rad17	0.60	1.28E-03	2.30E-03
5687	ENSMUSG00000054309	Cpsf3	-0.47	1.28E-03	2.30E-03
5688	ENSMUSG00000019039	Dalrd3	0.46	1.28E-03	2.31E-03
5689	ENSMUSG00000027187	Cat	-0.47	1.29E-03	2.31E-03
5690	ENSMUSG00000025759	Mfsd8	-0.50	1.30E-03	2.34E-03
5691	ENSMUSG00000043090	Zfp866	0.72	1.30E-03	2.34E-03
5692	ENSMUSG00000074994	Qser1	0.70	1.31E-03	2.35E-03
5693	ENSMUSG00000038206	Fbxo8	0.49	1.31E-03	2.35E-03
5694	ENSMUSG00000021360	Gcnt2	-0.73	1.31E-03	2.35E-03
5695	ENSMUSG00000068551	Zfp467	-0.47	1.32E-03	2.36E-03
5696	ENSMUSG00000093456	Gm20662	-0.68	1.32E-03	2.37E-03
5697	ENSMUSG00000020462	Cfap36	-0.62	1.32E-03	2.38E-03

5698	ENSMUSG00000040659	Efhd2	-0.53	1.34E-03	2.40E-03
5699	ENSMUSG00000040648	Ppip5k2	-0.70	1.35E-03	2.42E-03
5700	ENSMUSG00000058392	Rrp1b	-0.69	1.35E-03	2.42E-03
5701	ENSMUSG00000007670	Khsrp	-0.60	1.36E-03	2.43E-03
5702	ENSMUSG00000032375	Aph1b	-0.58	1.37E-03	2.46E-03
5703	ENSMUSG00000014763	Fam120b	-0.55	1.37E-03	2.46E-03
5704	ENSMUSG00000034789	Rab24	0.50	1.37E-03	2.46E-03
5705	ENSMUSG00000036275	9530068E07Rik	-0.46	1.38E-03	2.47E-03
5706	ENSMUSG00000002908	Kcnn1	0.68	1.38E-03	2.47E-03
5707	ENSMUSG00000041895	Wipi1	-0.59	1.38E-03	2.47E-03
5708	ENSMUSG00000004936	Map2k1	-0.45	1.38E-03	2.47E-03
5709	ENSMUSG00000066175	2510046G10Rik	0.72	1.38E-03	2.47E-03
5710	ENSMUSG00000029175	Slc35f6	0.48	1.38E-03	2.47E-03
5711	ENSMUSG00000002320	Tm9sf1	-0.48	1.38E-03	2.48E-03
5712	ENSMUSG00000027394	Ttl	0.68	1.39E-03	2.48E-03
5713	ENSMUSG00000022682	Rrn3	-0.58	1.39E-03	2.48E-03
5714	ENSMUSG00000043542	Zc2hc1a	-0.62	1.39E-03	2.49E-03
5715	ENSMUSG00000054604	Cggbp1	-0.56	1.39E-03	2.49E-03
5716	ENSMUSG00000027931	Npr1	0.66	1.39E-03	2.50E-03
5717	ENSMUSG00000015804	Med28	0.48	1.40E-03	2.50E-03
5718	ENSMUSG00000071369	Map3k5	-0.67	1.40E-03	2.50E-03
5719	ENSMUSG00000029290	Zfp326	0.76	1.40E-03	2.51E-03
5720	ENSMUSG00000046201	Scaf8	-0.66	1.40E-03	2.51E-03
5721	ENSMUSG00000086586	Gm11192	-0.74	1.40E-03	2.51E-03
5722	ENSMUSG00000020850	Prpf8	-0.50	1.40E-03	2.51E-03
5723	ENSMUSG00000060098	Prmt7	0.53	1.41E-03	2.52E-03
5724	ENSMUSG00000102615	Gm37844	-0.74	1.41E-03	2.53E-03
5725	ENSMUSG00000051232	Tmem199	0.52	1.41E-03	2.53E-03
5726	ENSMUSG00000027316	Gfra4	-0.63	1.42E-03	2.53E-03
5727	ENSMUSG00000000902	Smarcb1	0.47	1.43E-03	2.55E-03
5728	ENSMUSG00000031504	Rab20	-0.72	1.43E-03	2.55E-03
5729	ENSMUSG00000049285	Mblac1	0.68	1.44E-03	2.56E-03
5730	ENSMUSG00000042694	Stn1	-0.76	1.44E-03	2.57E-03
5731	ENSMUSG00000022336	Eif3e	0.46	1.44E-03	2.58E-03
5732	ENSMUSG00000066357	Wdr6	-0.44	1.45E-03	2.58E-03
5733	ENSMUSG00000020780	Srp68	-0.53	1.45E-03	2.59E-03
5734	ENSMUSG00000020869	Lrrc59	-0.51	1.45E-03	2.59E-03
5735	ENSMUSG00000031156	Slc35a2	-0.59	1.46E-03	2.60E-03
5736	ENSMUSG00000043384	Gprasp1	-0.62	1.46E-03	2.61E-03
5737	ENSMUSG00000042558	Adprhl2	0.47	1.47E-03	2.61E-03
5738	ENSMUSG00000032555	Topbp1	-0.74	1.47E-03	2.62E-03
5739	ENSMUSG00000030727	Rabep2	-0.71	1.47E-03	2.62E-03
5740	ENSMUSG00000026277	Stk25	-0.45	1.49E-03	2.65E-03
5741	ENSMUSG00000039191	Rbpj	-0.66	1.49E-03	2.65E-03
5742	ENSMUSG00000093768	Gm20650	0.61	1.49E-03	2.66E-03
5743	ENSMUSG00000024054	Smchd1	-0.71	1.50E-03	2.67E-03
5744	ENSMUSG00000024778	Fas	0.51	1.50E-03	2.68E-03
5745	ENSMUSG00000038072	Galnt11	-0.59	1.52E-03	2.70E-03
5746	ENSMUSG00000000276	Dgke	-0.79	1.52E-03	2.71E-03
5747	ENSMUSG00000028211	Trp53inp1	-0.72	1.53E-03	2.72E-03

5748	ENSMUSG00000031216	Stard8	-0.79	1.54E-03	2.74E-03
5749	ENSMUSG00000003099	Ppp5c	0.46	1.56E-03	2.77E-03
5750	ENSMUSG00000054414	Slc30a7	-0.72	1.56E-03	2.77E-03
5751	ENSMUSG00000020993	Trappc6b	-0.45	1.56E-03	2.78E-03
5752	ENSMUSG00000032350	Gclc	-0.53	1.56E-03	2.78E-03
5753	ENSMUSG00000108591	Particl	-0.53	1.57E-03	2.79E-03
5754	ENSMUSG00000028347	Tmeff1	0.54	1.57E-03	2.79E-03
5755	ENSMUSG00000024941	Scyl1	0.44	1.57E-03	2.79E-03
5756	ENSMUSG00000090264	Eif4ebp3	-0.79	1.58E-03	2.81E-03
5757	ENSMUSG00000044857	Lemd2	-0.48	1.58E-03	2.81E-03
5758	ENSMUSG00000045193	Cirbp	-0.60	1.59E-03	2.83E-03
5759	ENSMUSG00000066735	Vkorc1l1	-0.57	1.59E-03	2.83E-03
5760	ENSMUSG00000041124	Msantd4	0.58	1.60E-03	2.83E-03
5761	ENSMUSG00000027395	Polr1b	-0.69	1.60E-03	2.84E-03
5762	ENSMUSG00000000194	Gpr107	-0.52	1.60E-03	2.84E-03
5763	ENSMUSG00000013833	Med16	0.52	1.60E-03	2.85E-03
5764	ENSMUSG00000031540	Kat6a	-0.70	1.60E-03	2.85E-03
5765	ENSMUSG00000026842	Abl1	-0.60	1.61E-03	2.85E-03
5766	ENSMUSG00000043866	Taf10	0.43	1.61E-03	2.86E-03
5767	ENSMUSG00000045007	Tubg2	0.55	1.61E-03	2.86E-03
5768	ENSMUSG00000054199	Gon4l	-0.56	1.62E-03	2.86E-03
5769	ENSMUSG00000070733	Fryl	-0.66	1.62E-03	2.87E-03
5770	ENSMUSG00000034126	Pomt2	-0.61	1.63E-03	2.88E-03
5771	ENSMUSG00000032705	Exd2	-0.67	1.63E-03	2.88E-03
5772	ENSMUSG00000027655	Dhx35	0.82	1.63E-03	2.90E-03
5773	ENSMUSG00000056536	Pign	-0.69	1.64E-03	2.90E-03
5774	ENSMUSG00000062590	Armc9	-0.68	1.64E-03	2.90E-03
5775	ENSMUSG00000033454	Zbtb1	-0.66	1.64E-03	2.90E-03
5776	ENSMUSG00000039849	Pcif1	-0.46	1.64E-03	2.90E-03
5777	ENSMUSG00000025060	Slk	-0.54	1.64E-03	2.90E-03
5778	ENSMUSG00000005982	Naa60	-0.45	1.64E-03	2.91E-03
5779	ENSMUSG00000024350	Dnajc18	-0.75	1.65E-03	2.92E-03
5780	ENSMUSG00000035824	Tk2	-0.56	1.65E-03	2.92E-03
5781	ENSMUSG00000046722	Cdc42se1	-0.48	1.66E-03	2.93E-03
5782	ENSMUSG00000022217	Emc9	0.46	1.66E-03	2.94E-03
5783	ENSMUSG00000009470	Tnpo1	-0.54	1.66E-03	2.94E-03
5784	ENSMUSG00000035161	Ints6	-0.61	1.67E-03	2.95E-03
5785	ENSMUSG00000040029	Ipo8	-0.52	1.67E-03	2.95E-03
5786	ENSMUSG00000009681	Bcr	-0.70	1.68E-03	2.97E-03
5787	ENSMUSG00000012126	Ubxn11	-0.72	1.68E-03	2.97E-03
5788	ENSMUSG00000034187	Nsf	-0.48	1.68E-03	2.98E-03
5789	ENSMUSG00000027219	Slc28a2	-0.70	1.69E-03	2.98E-03
5790	ENSMUSG00000009293	Ube2g2	-0.46	1.69E-03	2.98E-03
5791	ENSMUSG00000109350	Gm44805	0.65	1.69E-03	2.99E-03
5792	ENSMUSG00000026469	Xpr1	-0.68	1.69E-03	2.99E-03
5793	ENSMUSG00000032235	Ice2	-0.72	1.69E-03	2.99E-03
5794	ENSMUSG00000029471	Camkk2	-0.53	1.70E-03	3.01E-03
5795	ENSMUSG00000003500	Impdh1	0.48	1.71E-03	3.02E-03
5796	ENSMUSG00000039844	Rapgef1	-0.49	1.72E-03	3.03E-03
5797	ENSMUSG00000051427	Ccdc157	-0.88	1.72E-03	3.03E-03

5798	ENSMUSG00000022668	Gtpbp8	0.49	1.72E-03	3.03E-03
5799	ENSMUSG00000107283	Mpv17	0.42	1.72E-03	3.03E-03
5800	ENSMUSG00000037982	Gm9725	-0.75	1.72E-03	3.03E-03
5801	ENSMUSG00000029599	Ddx54	-0.47	1.72E-03	3.03E-03
5802	ENSMUSG00000051579	Tceal8	-0.63	1.72E-03	3.04E-03
5803	ENSMUSG00000025534	Gusb	-0.47	1.73E-03	3.05E-03
5804	ENSMUSG00000022160	Mettl3	-0.63	1.73E-03	3.06E-03
5805	ENSMUSG00000036446	Lum	-0.90	1.74E-03	3.06E-03
5806	ENSMUSG00000041750	Cd1d2	-0.57	1.74E-03	3.07E-03
5807	ENSMUSG00000111409	Rnf26	-0.57	1.74E-03	3.07E-03
5808	ENSMUSG00000036435	Exoc1	-0.65	1.74E-03	3.07E-03
5809	ENSMUSG00000043252	Tmem64	0.48	1.74E-03	3.07E-03
5810	ENSMUSG00000020898	Ctc1	-0.78	1.75E-03	3.08E-03
5811	ENSMUSG00000028621	Cyb5rl	0.49	1.75E-03	3.08E-03
5812	ENSMUSG00000059811	Atl2	-0.48	1.75E-03	3.09E-03
5813	ENSMUSG00000025262	Fam120c	-0.78	1.76E-03	3.09E-03
5814	ENSMUSG00000042121	Ssh1	-0.60	1.76E-03	3.10E-03
5815	ENSMUSG00000020111	Micu1	0.44	1.76E-03	3.10E-03
5816	ENSMUSG00000044328	Trp53i13	-0.48	1.77E-03	3.12E-03
5817	ENSMUSG00000040618	Pck2	-0.69	1.78E-03	3.12E-03
5818	ENSMUSG00000094103	1700047I17Rik2	0.44	1.79E-03	3.14E-03
5819	ENSMUSG00000038028	Tigar	0.65	1.81E-03	3.18E-03
5820	ENSMUSG00000033728	Lrrc14	-0.51	1.81E-03	3.19E-03
5821	ENSMUSG00000021156	Zmynd11	-0.53	1.82E-03	3.19E-03
5822	ENSMUSG00000006476	Nsmf	-0.60	1.82E-03	3.19E-03
5823	ENSMUSG00000040549	Ckap5	-0.64	1.82E-03	3.20E-03
5824	ENSMUSG00000038495	Otud7b	-0.57	1.83E-03	3.21E-03
5825	ENSMUSG00000006423	C330007P06Rik	0.43	1.83E-03	3.21E-03
5826	ENSMUSG00000023572	Ccndbp1	0.47	1.83E-03	3.21E-03
5827	ENSMUSG00000073542	Cep76	-0.71	1.83E-03	3.22E-03
5828	ENSMUSG00000026349	Ccnt2	-0.62	1.83E-03	3.22E-03
5829	ENSMUSG00000085408	C530005A16Rik	0.70	1.83E-03	3.22E-03
5830	ENSMUSG00000028136	Snx27	0.52	1.84E-03	3.22E-03
5831	ENSMUSG00000036214	Znrd1as	0.76	1.84E-03	3.22E-03
5832	ENSMUSG00000036160	Surf6	-0.62	1.84E-03	3.22E-03
5833	ENSMUSG00000043091	Tuba1c	0.55	1.84E-03	3.23E-03
5834	ENSMUSG00000026705	Klhl20	-0.77	1.85E-03	3.24E-03
5835	ENSMUSG00000003559	As3mt	0.72	1.86E-03	3.26E-03
5836	ENSMUSG00000034931	Dhx8	-0.58	1.87E-03	3.28E-03
5837	ENSMUSG00000070643	Sox13	-0.75	1.87E-03	3.28E-03
5838	ENSMUSG00000026339	Ccdc93	-0.72	1.87E-03	3.28E-03
5839	ENSMUSG00000021179	Nrde2	-0.64	1.88E-03	3.29E-03
5840	ENSMUSG00000030986	Dhx32	-0.62	1.88E-03	3.30E-03
5841	ENSMUSG00000090290	Tarbp1	-0.70	1.88E-03	3.30E-03
5842	ENSMUSG00000041057	Wdr43	-0.53	1.89E-03	3.30E-03
5843	ENSMUSG00000041846	Ppp4r3a	-0.49	1.89E-03	3.31E-03
5844	ENSMUSG00000037742	Eef1a1	0.43	1.89E-03	3.31E-03
5845	ENSMUSG00000022359	Wdyhv1	0.76	1.90E-03	3.32E-03
5846	ENSMUSG00000020376	Rnf130	0.44	1.90E-03	3.33E-03
5847	ENSMUSG00000039952	Dag1	-0.50	1.90E-03	3.33E-03

5848	ENSMUSG00000026111	Unc50	0.47	1.90E-03	3.33E-03
5849	ENSMUSG00000033739	Fkbpl	0.68	1.91E-03	3.33E-03
5850	ENSMUSG00000027905	Ddx20	-0.62	1.91E-03	3.34E-03
5851	ENSMUSG00000029103	Lrpap1	-0.68	1.91E-03	3.34E-03
5852	ENSMUSG00000004535	Tax1bp1	-0.41	1.92E-03	3.35E-03
5853	ENSMUSG00000038095	Sbno1	0.44	1.92E-03	3.35E-03
5854	ENSMUSG00000092386	Gm20536	0.44	1.92E-03	3.35E-03
5855	ENSMUSG00000000811	Txnrd3	-0.52	1.92E-03	3.35E-03
5856	ENSMUSG00000029095	Ablim2	-0.58	1.92E-03	3.36E-03
5857	ENSMUSG00000022453	Naga	-0.44	1.93E-03	3.37E-03
5858	ENSMUSG00000036291	Ap5m1	0.60	1.93E-03	3.37E-03
5859	ENSMUSG00000095595	Fam177a	0.43	1.94E-03	3.38E-03
5860	ENSMUSG00000059119	Nap1l4	0.42	1.94E-03	3.38E-03
5861	ENSMUSG00000034334	Fam151b	0.76	1.94E-03	3.39E-03
5862	ENSMUSG00000040146	Rgl3	0.68	1.95E-03	3.40E-03
5863	ENSMUSG00000037062	Sh3glb1	0.42	1.95E-03	3.41E-03
5864	ENSMUSG00000002625	Akap8l	-0.69	1.95E-03	3.41E-03
5865	ENSMUSG00000056737	Capg	-0.87	1.96E-03	3.41E-03
5866	ENSMUSG00000021779	Thrb	-0.54	1.96E-03	3.42E-03
5867	ENSMUSG00000020455	Trim11	-0.60	1.97E-03	3.43E-03
5868	ENSMUSG00000029388	Eif2b1	0.43	1.97E-03	3.44E-03
5869	ENSMUSG00000037933	Bicd2	-0.49	1.97E-03	3.44E-03
5870	ENSMUSG00000073436	Eme2	0.44	1.98E-03	3.45E-03
5871	ENSMUSG00000066278	Vps37b	-0.68	1.98E-03	3.45E-03
5872	ENSMUSG00000018669	Cdk5rap3	-0.42	1.98E-03	3.45E-03
5873	ENSMUSG00000027770	Dhx36	-0.53	1.99E-03	3.46E-03
5874	ENSMUSG00000021395	Spin1	-0.53	1.99E-03	3.47E-03
5875	ENSMUSG00000029059	Fam213b	-0.51	2.00E-03	3.49E-03
5876	ENSMUSG00000019370	Calm3	0.48	2.01E-03	3.49E-03
5877	ENSMUSG00000061397	Krt79	0.69	2.02E-03	3.52E-03
5878	ENSMUSG00000004929	Thop1	-0.53	2.02E-03	3.52E-03
5879	ENSMUSG00000030259	Rassf8	-0.68	2.03E-03	3.52E-03
5880	ENSMUSG00000034263	Ints14	-0.48	2.03E-03	3.52E-03
5881	ENSMUSG00000042487	Leo1	-0.69	2.03E-03	3.54E-03
5882	ENSMUSG00000070372	Capza1	0.46	2.03E-03	3.54E-03
5883	ENSMUSG00000002741	Ykt6	-0.50	2.05E-03	3.56E-03
5884	ENSMUSG00000062110	Scfd2	-0.54	2.06E-03	3.58E-03
5885	ENSMUSG00000054659	Pm20d2	-0.76	2.07E-03	3.60E-03
5886	ENSMUSG00000026966	Ssna1	0.54	2.08E-03	3.61E-03
5887	ENSMUSG00000074918	Inafm2	-0.44	2.08E-03	3.62E-03
5888	ENSMUSG00000031575	Ash2l	-0.52	2.09E-03	3.62E-03
5889	ENSMUSG00000039263	Npepl1	0.41	2.09E-03	3.64E-03
5890	ENSMUSG00000047090	Tmem198b	-0.58	2.10E-03	3.64E-03
5891	ENSMUSG00000028826	Tmem57	-0.50	2.10E-03	3.64E-03
5892	ENSMUSG00000056211	R3hdm1	0.49	2.10E-03	3.65E-03
5893	ENSMUSG00000015488	Cacfd1	0.44	2.11E-03	3.67E-03
5894	ENSMUSG00000002496	Tsc2	-0.45	2.13E-03	3.69E-03
5895	ENSMUSG00000078864	Gm14322	0.71	2.13E-03	3.70E-03
5896	ENSMUSG00000019814	Ltv1	-0.64	2.14E-03	3.71E-03
5897	ENSMUSG00000037740	Mrps26	0.45	2.14E-03	3.71E-03

5898	ENSMUSG00000024943	Smc5	-0.72	2.15E-03	3.72E-03
5899	ENSMUSG00000050556	Kcnb1	0.60	2.16E-03	3.74E-03
5900	ENSMUSG00000033396	Spg11	-0.55	2.18E-03	3.77E-03
5901	ENSMUSG00000022106	Rcbtb2	0.42	2.18E-03	3.78E-03
5902	ENSMUSG00000037270	4932438A13Rik	-0.59	2.18E-03	3.78E-03
5903	ENSMUSG00000105084	Gm43365	-0.68	2.19E-03	3.79E-03
5904	ENSMUSG00000052906	Ubxn8	0.48	2.19E-03	3.79E-03
5905	ENSMUSG00000071379	Hpcal1	-0.69	2.19E-03	3.79E-03
5906	ENSMUSG00000020647	Ncoa1	-0.58	2.19E-03	3.79E-03
5907	ENSMUSG00000026974	Zmynd19	0.69	2.19E-03	3.79E-03
5908	ENSMUSG00000002455	Prpf6	-0.45	2.20E-03	3.80E-03
5909	ENSMUSG00000035898	Uba6	-0.64	2.21E-03	3.82E-03
5910	ENSMUSG00000035278	Plekhj1	0.51	2.21E-03	3.83E-03
5911	ENSMUSG00000026767	Fam188a	0.49	2.22E-03	3.83E-03
5912	ENSMUSG00000068329	Htra2	0.42	2.23E-03	3.85E-03
5913	ENSMUSG00000001576	Ergic1	0.48	2.23E-03	3.86E-03
5914	ENSMUSG00000045302	Preb	-0.44	2.24E-03	3.88E-03
5915	ENSMUSG00000028744	Pqlc2	0.55	2.24E-03	3.88E-03
5916	ENSMUSG00000025959	Klf7	-0.71	2.24E-03	3.88E-03
5917	ENSMUSG00000005469	Prkaca	0.45	2.25E-03	3.90E-03
5918	ENSMUSG00000086816	Gm16726	-0.67	2.26E-03	3.90E-03
5919	ENSMUSG00000000085	Scmh1	-0.66	2.26E-03	3.90E-03
5920	ENSMUSG00000057561	Eif1a	-0.43	2.27E-03	3.93E-03
5921	ENSMUSG00000045930	Clec14a	-0.81	2.28E-03	3.93E-03
5922	ENSMUSG00000022848	Dirc2	-0.51	2.28E-03	3.94E-03
5923	ENSMUSG00000063873	Slc24a3	0.43	2.28E-03	3.94E-03
5924	ENSMUSG00000015189	Casd1	-0.59	2.30E-03	3.97E-03
5925	ENSMUSG00000029238	Clock	-0.74	2.30E-03	3.97E-03
5926	ENSMUSG00000027854	Sike1	0.53	2.30E-03	3.97E-03
5927	ENSMUSG00000018398	Sep-08	-0.75	2.30E-03	3.98E-03
5928	ENSMUSG00000061589	Dot1l	-0.63	2.31E-03	3.99E-03
5929	ENSMUSG00000031640	Gm45753	-0.62	2.31E-03	3.99E-03
5930	ENSMUSG00000059479	B3gnt8	0.43	2.32E-03	4.00E-03
5931	ENSMUSG00000030278	Cidec	-0.52	2.32E-03	4.00E-03
5932	ENSMUSG00000097124	A530020G20Rik	-0.70	2.32E-03	4.01E-03
5933	ENSMUSG00000070002	Ell	-0.61	2.32E-03	4.01E-03
5934	ENSMUSG00000031634	Ufsp2	0.45	2.33E-03	4.01E-03
5935	ENSMUSG00000035370	Adat3	-0.43	2.33E-03	4.01E-03
5936	ENSMUSG00000036052	Dnajb5	-0.73	2.34E-03	4.02E-03
5937	ENSMUSG00000034327	Kctd9	0.62	2.35E-03	4.04E-03
5938	ENSMUSG00000057230	Aak1	-0.61	2.36E-03	4.06E-03
5939	ENSMUSG00000024758	Rtn3	-0.45	2.36E-03	4.06E-03
5940	ENSMUSG00000025375	Aatk	-0.69	2.36E-03	4.06E-03
5941	ENSMUSG00000032288	Imp3	0.50	2.36E-03	4.06E-03
5942	ENSMUSG00000020133	2310011J03Rik	0.45	2.37E-03	4.08E-03
5943	ENSMUSG00000021009	Ptpn21	-0.67	2.37E-03	4.08E-03
5944	ENSMUSG00000026999	Nup35	0.58	2.37E-03	4.08E-03
5945	ENSMUSG00000030753	Thap12	-0.50	2.38E-03	4.09E-03
5946	ENSMUSG00000045374	Wdr81	-0.73	2.38E-03	4.10E-03
5947	ENSMUSG00000009418	Nav1	-0.75	2.38E-03	4.10E-03

5948	ENSMUSG00000066152	Slc31a2	-0.57	2.39E-03	4.11E-03
5949	ENSMUSG00000027164	Traf6	-0.62	2.40E-03	4.12E-03
5950	ENSMUSG00000033294	Noc4l	-0.54	2.40E-03	4.13E-03
5951	ENSMUSG00000020178	Adora2a	-0.68	2.40E-03	4.13E-03
5952	ENSMUSG00000022836	Mylk	0.62	2.41E-03	4.14E-03
5953	ENSMUSG00000053877	Srcap	-0.47	2.41E-03	4.15E-03
5954	ENSMUSG00000036120	Rfxank	0.54	2.41E-03	4.15E-03
5955	ENSMUSG00000024052	Lpin2	0.67	2.42E-03	4.15E-03
5956	ENSMUSG00000022407	Adsl	0.42	2.43E-03	4.17E-03
5957	ENSMUSG00000087403	Kantr	-0.74	2.43E-03	4.17E-03
5958	ENSMUSG00000033530	Ttc7b	-0.49	2.43E-03	4.17E-03
5959	ENSMUSG00000040383	Aqr	-0.52	2.43E-03	4.18E-03
5960	ENSMUSG00000055817	Mta3	-0.48	2.43E-03	4.18E-03
5961	ENSMUSG00000036941	Elac1	-0.64	2.44E-03	4.19E-03
5962	ENSMUSG00000087352	Gm12999	0.68	2.44E-03	4.19E-03
5963	ENSMUSG00000031601	Cnot7	0.43	2.44E-03	4.19E-03
5964	ENSMUSG00000035901	Dennd5a	0.42	2.45E-03	4.19E-03
5965	ENSMUSG00000038084	Opa1	0.41	2.45E-03	4.20E-03
5966	ENSMUSG00000036615	Rfxap	-0.62	2.45E-03	4.21E-03
5967	ENSMUSG00000004897	Hdgf	0.40	2.46E-03	4.21E-03
5968	ENSMUSG00000039470	Zdhhc2	0.53	2.46E-03	4.21E-03
5969	ENSMUSG00000046844	Vat1l	-0.70	2.46E-03	4.22E-03
5970	ENSMUSG00000001729	Akt1	-0.43	2.46E-03	4.22E-03
5971	ENSMUSG00000038369	Ncoa6	-0.70	2.47E-03	4.23E-03
5972	ENSMUSG00000005378	Wbscr22	0.42	2.47E-03	4.23E-03
5973	ENSMUSG00000038085	Cnbd2	0.55	2.47E-03	4.23E-03
5974	ENSMUSG00000060739	Nsa2	0.63	2.47E-03	4.23E-03
5975	ENSMUSG00000033272	Slc35a4	0.41	2.47E-03	4.23E-03
5976	ENSMUSG00000040423	Rc3h1	-0.58	2.47E-03	4.23E-03
5977	ENSMUSG00000097397	Gm16861	-0.52	2.48E-03	4.25E-03
5978	ENSMUSG00000042015	Wdr41	-0.57	2.49E-03	4.26E-03
5979	ENSMUSG00000038622	Med30	0.54	2.49E-03	4.26E-03
5980	ENSMUSG00000025234	Arih1	-0.45	2.51E-03	4.28E-03
5981	ENSMUSG00000028601	Echdc2	-0.51	2.51E-03	4.28E-03
5982	ENSMUSG00000042063	Zfp386	0.54	2.51E-03	4.28E-03
5983	ENSMUSG00000003184	Irf3	0.45	2.51E-03	4.30E-03
5984	ENSMUSG00000029407	Uso1	-0.52	2.52E-03	4.31E-03
5985	ENSMUSG00000010453	Kansl3	-0.44	2.54E-03	4.34E-03
5986	ENSMUSG00000034928	Rnf44	0.48	2.54E-03	4.34E-03
5987	ENSMUSG00000028093	Acp6	-0.43	2.55E-03	4.36E-03
5988	ENSMUSG00000028706	Nsun4	0.43	2.56E-03	4.37E-03
5989	ENSMUSG00000020107	Anapc16	0.49	2.56E-03	4.37E-03
5990	ENSMUSG00000029617	Ccz1	0.43	2.56E-03	4.38E-03
5991	ENSMUSG00000004462	Tbccd1	-0.65	2.57E-03	4.38E-03
5992	ENSMUSG00000040181	Fmo1	0.52	2.57E-03	4.39E-03
5993	ENSMUSG00000038286	Bphl	0.43	2.58E-03	4.39E-03
5994	ENSMUSG00000032307	Ube2q2	0.44	2.58E-03	4.40E-03
5995	ENSMUSG00000000708	Kat2b	-0.56	2.60E-03	4.44E-03
5996	ENSMUSG00000033128	Gga1	-0.43	2.61E-03	4.46E-03
5997	ENSMUSG00000028809	Srrm1	-0.49	2.62E-03	4.47E-03

5998	ENSMUSG00000032370	Lactb	0.61	2.63E-03	4.48E-03
5999	ENSMUSG00000097275	Gm26648	-0.61	2.65E-03	4.51E-03
6000	ENSMUSG00000019132	BC005537	0.76	2.65E-03	4.52E-03
6001	ENSMUSG00000022100	Xpo7	-0.51	2.66E-03	4.53E-03
6002	ENSMUSG00000024799	Tm7sf2	0.54	2.67E-03	4.55E-03
6003	ENSMUSG00000051043	Gprc5c	0.52	2.69E-03	4.58E-03
6004	ENSMUSG00000040811	Eml2	-0.70	2.69E-03	4.58E-03
6005	ENSMUSG00000031309	Rps6ka3	-0.64	2.69E-03	4.58E-03
6006	ENSMUSG00000046865	Fbl	0.51	2.69E-03	4.58E-03
6007	ENSMUSG00000035469	Rcbtb1	-0.58	2.70E-03	4.59E-03
6008	ENSMUSG00000021962	Dcp1a	-0.61	2.70E-03	4.60E-03
6009	ENSMUSG00000024079	Eif2ak2	-0.71	2.70E-03	4.60E-03
6010	ENSMUSG00000032096	Arcn1	-0.48	2.71E-03	4.61E-03
6011	ENSMUSG00000031851	Ntpcr	-0.53	2.71E-03	4.61E-03
6012	ENSMUSG00000028180	Zranb2	0.49	2.71E-03	4.62E-03
6013	ENSMUSG00000070509	Rgma	-0.77	2.72E-03	4.62E-03
6014	ENSMUSG00000104195	B230377A18Rik	0.70	2.74E-03	4.66E-03
6015	ENSMUSG00000052656	Rnf103	-0.46	2.76E-03	4.69E-03
6016	ENSMUSG00000029624	Ptcd1	0.43	2.76E-03	4.70E-03
6017	ENSMUSG00000060181	Slc35e3	-0.61	2.77E-03	4.70E-03
6018	ENSMUSG00000024493	Lars	-0.54	2.77E-03	4.70E-03
6019	ENSMUSG00000040351	Ankib1	-0.60	2.79E-03	4.73E-03
6020	ENSMUSG00000058979	Hdhd5	0.57	2.80E-03	4.75E-03
6021	ENSMUSG00000050908	Tvp23a	0.57	2.80E-03	4.75E-03
6022	ENSMUSG00000028483	Snapc3	-0.68	2.81E-03	4.77E-03
6023	ENSMUSG00000030766	Arhgap17	-0.57	2.81E-03	4.77E-03
6024	ENSMUSG00000035845	Alg12	0.61	2.81E-03	4.77E-03
6025	ENSMUSG00000019579	Mydgf	0.44	2.82E-03	4.78E-03
6026	ENSMUSG00000031333	Abcb7	0.56	2.82E-03	4.79E-03
6027	ENSMUSG00000113949	Scamp4	-0.43	2.83E-03	4.80E-03
6028	ENSMUSG00000035696	Rnf38	-0.70	2.83E-03	4.80E-03
6029	ENSMUSG00000020048	Hsp90b1	-0.40	2.83E-03	4.81E-03
6030	ENSMUSG00000031245	Hmgn5	0.69	2.84E-03	4.81E-03
6031	ENSMUSG00000089704	Galnt2	-0.44	2.85E-03	4.83E-03
6032	ENSMUSG00000011114	Tbrg1	-0.56	2.86E-03	4.84E-03
6033	ENSMUSG00000072825	Cep170b	-0.67	2.87E-03	4.86E-03
6034	ENSMUSG00000075010	AW112010	-0.44	2.87E-03	4.87E-03
6035	ENSMUSG00000074797	Itpa	0.49	2.87E-03	4.87E-03
6036	ENSMUSG00000040331	Nsmce4a	0.46	2.88E-03	4.88E-03
6037	ENSMUSG00000027412	Lpin3	-0.68	2.88E-03	4.88E-03
6038	ENSMUSG00000047264	Zfp358	-0.55	2.89E-03	4.90E-03
6039	ENSMUSG00000024069	Slc30a6	0.54	2.91E-03	4.93E-03
6040	ENSMUSG00000040158	Tax1bp3	0.43	2.92E-03	4.93E-03
6041	ENSMUSG00000020309	Chac2	0.63	2.92E-03	4.95E-03
6042	ENSMUSG00000022265	Ank	0.57	2.93E-03	4.96E-03
6043	ENSMUSG00000034902	Pip5k1c	-0.58	2.93E-03	4.97E-03
6044	ENSMUSG00000021938	Pspc1	-0.65	2.94E-03	4.97E-03
6045	ENSMUSG00000003623	Crot	0.41	2.94E-03	4.97E-03
6046	ENSMUSG00000000759	Tubgcp3	-0.60	2.94E-03	4.98E-03
6047	ENSMUSG00000036315	Znrd1	0.63	2.95E-03	4.98E-03

6048	ENSMUSG00000025875	Tspan17	-0.44	2.95E-03	4.99E-03
6049	ENSMUSG00000086740	Gm17029	0.42	2.96E-03	5.00E-03
6050	ENSMUSG00000026283	Ing5	-0.64	2.97E-03	5.02E-03
6051	ENSMUSG00000017801	Mlx	0.49	2.98E-03	5.03E-03
6052	ENSMUSG00000026915	Strbp	0.61	2.98E-03	5.04E-03
6053	ENSMUSG00000031523	Dlc1	-0.48	3.00E-03	5.06E-03
6054	ENSMUSG00000001774	Chordc1	0.44	3.00E-03	5.06E-03
6055	ENSMUSG00000047454	Gphn	-0.46	3.00E-03	5.07E-03
6056	ENSMUSG00000022386	Trmu	0.48	3.00E-03	5.07E-03
6057	ENSMUSG00000098374	Gm28043	-0.43	3.01E-03	5.08E-03
6058	ENSMUSG00000041491	Cep78	-0.71	3.01E-03	5.08E-03
6059	ENSMUSG00000002083	Bbc3	-0.69	3.01E-03	5.08E-03
6060	ENSMUSG00000060376	Bckdha	0.42	3.06E-03	5.17E-03
6061	ENSMUSG00000063445	Nmral1	-0.67	3.06E-03	5.17E-03
6062	ENSMUSG00000026484	Rnf2	-0.58	3.07E-03	5.18E-03
6063	ENSMUSG00000022433	Csnk1e	-0.53	3.07E-03	5.18E-03
6064	ENSMUSG00000026072	Il1r1	-0.68	3.08E-03	5.20E-03
6065	ENSMUSG00000031016	Wee1	-0.64	3.08E-03	5.20E-03
6066	ENSMUSG00000017929	B4galt5	0.70	3.08E-03	5.20E-03
6067	ENSMUSG00000019254	Ppp1r12c	-0.46	3.09E-03	5.20E-03
6068	ENSMUSG00000035206	Sppl2b	-0.50	3.10E-03	5.23E-03
6069	ENSMUSG00000092329	Gm20388	-0.43	3.11E-03	5.24E-03
6070	ENSMUSG00000032485	Scap	-0.44	3.11E-03	5.24E-03
6071	ENSMUSG00000008398	Elk3	-0.74	3.11E-03	5.25E-03
6072	ENSMUSG00000021577	Sdha	0.42	3.12E-03	5.26E-03
6073	ENSMUSG00000024942	Capn1	-0.54	3.14E-03	5.28E-03
6074	ENSMUSG00000021265	Slc25a29	-0.61	3.14E-03	5.28E-03
6075	ENSMUSG00000027341	Tmem230	0.48	3.15E-03	5.30E-03
6076	ENSMUSG00000037851	Iars	-0.45	3.16E-03	5.31E-03
6077	ENSMUSG00000036940	Kdm1a	-0.54	3.16E-03	5.32E-03
6078	ENSMUSG00000062421	Arf2	-0.54	3.17E-03	5.33E-03
6079	ENSMUSG00000031731	Ap1g1	-0.43	3.18E-03	5.35E-03
6080	ENSMUSG00000042111	Ccdc115	0.59	3.18E-03	5.36E-03
6081	ENSMUSG00000042472	Zfp410	-0.59	3.19E-03	5.37E-03
6082	ENSMUSG00000039599	Fam149b	0.44	3.19E-03	5.37E-03
6083	ENSMUSG00000108677	Gm44759	-0.77	3.21E-03	5.39E-03
6084	ENSMUSG00000000127	Fer	-0.62	3.21E-03	5.39E-03
6085	ENSMUSG00000029504	Ddx51	-0.60	3.21E-03	5.40E-03
6086	ENSMUSG00000029833	Trim24	-0.62	3.21E-03	5.40E-03
6087	ENSMUSG00000014767	Tbp	0.53	3.22E-03	5.41E-03
6088	ENSMUSG00000029686	Cul1	-0.44	3.22E-03	5.41E-03
6089	ENSMUSG00000059897	Zfp930	0.64	3.22E-03	5.41E-03
6090	ENSMUSG00000003721	Insig2	-0.43	3.22E-03	5.41E-03
6091	ENSMUSG00000036943	Rab8b	-0.61	3.22E-03	5.41E-03
6092	ENSMUSG00000033439	Trmt13	0.64	3.22E-03	5.41E-03
6093	ENSMUSG00000097620	4921514A10Rik	0.57	3.23E-03	5.42E-03
6094	ENSMUSG00000052062	Pard3b	-0.65	3.23E-03	5.42E-03
6095	ENSMUSG00000037674	Rfx7	-0.65	3.26E-03	5.47E-03
6096	ENSMUSG00000010751	Tnfrsf22	-0.67	3.26E-03	5.47E-03
6097	ENSMUSG00000049232	Tigd2	0.56	3.27E-03	5.48E-03

6098	ENSMUSG00000041132	N4bp2l1	-0.50	3.30E-03	5.53E-03
6099	ENSMUSG00000015697	Setdb1	-0.57	3.30E-03	5.53E-03
6100	ENSMUSG00000028790	Khdrbs1	-0.48	3.30E-03	5.54E-03
6101	ENSMUSG00000025225	Nfkb2	-0.64	3.31E-03	5.55E-03
6102	ENSMUSG00000028865	Cd164l2	-0.64	3.31E-03	5.55E-03
6103	ENSMUSG00000046139	Patl1	0.54	3.33E-03	5.58E-03
6104	ENSMUSG00000110357	A030001D20Rik	-0.64	3.37E-03	5.64E-03
6105	ENSMUSG00000090706	Gm17233	0.45	3.37E-03	5.64E-03
6106	ENSMUSG00000032306	Mpi	0.54	3.37E-03	5.65E-03
6107	ENSMUSG00000030522	Mtmr10	-0.55	3.41E-03	5.71E-03
6108	ENSMUSG00000056820	Tsnax	-0.46	3.41E-03	5.71E-03
6109	ENSMUSG00000039810	Zc3h10	0.61	3.41E-03	5.71E-03
6110	ENSMUSG00000021338	Carmil1	-0.68	3.42E-03	5.72E-03
6111	ENSMUSG00000021356	Irf4	-0.71	3.42E-03	5.73E-03
6112	ENSMUSG00000032582	Rbm6	-0.63	3.42E-03	5.73E-03
6113	ENSMUSG00000021843	Ktn1	-0.60	3.43E-03	5.73E-03
6114	ENSMUSG00000042323	Pbrm1	-0.45	3.44E-03	5.75E-03
6115	ENSMUSG00000030061	Uba3	0.42	3.45E-03	5.76E-03
6116	ENSMUSG00000020198	Ap3d1	-0.39	3.45E-03	5.76E-03
6117	ENSMUSG00000020513	Tubd1	-0.60	3.45E-03	5.77E-03
6118	ENSMUSG00000099703	Gm28285	-0.52	3.48E-03	5.81E-03
6119	ENSMUSG00000018377	Vezf1	-0.53	3.48E-03	5.82E-03
6120	ENSMUSG00000032047	Acat1	0.40	3.49E-03	5.83E-03
6121	ENSMUSG00000029016	Clcn6	-0.61	3.49E-03	5.84E-03
6122	ENSMUSG00000037089	Slc35b2	0.42	3.50E-03	5.85E-03
6123	ENSMUSG00000025470	Zfp511	0.61	3.50E-03	5.85E-03
6124	ENSMUSG00000047656	Trpt1	0.55	3.52E-03	5.88E-03
6125	ENSMUSG00000041773	Enc1	-0.65	3.53E-03	5.89E-03
6126	ENSMUSG00000092463	Gm20489	-0.81	3.53E-03	5.89E-03
6127	ENSMUSG00000044791	Setd2	-0.64	3.53E-03	5.89E-03
6128	ENSMUSG00000031833	Mast3	-0.69	3.55E-03	5.92E-03
6129	ENSMUSG00000021096	Ppm1a	0.41	3.55E-03	5.93E-03
6130	ENSMUSG00000028497	Hacd4	-0.67	3.57E-03	5.95E-03
6131	ENSMUSG00000062078	Qk	-0.42	3.58E-03	5.96E-03
6132	ENSMUSG00000026317	Cln8	0.45	3.58E-03	5.96E-03
6133	ENSMUSG00000104548	Gm43857	-0.54	3.58E-03	5.97E-03
6134	ENSMUSG00000001285	Myg1	0.45	3.58E-03	5.97E-03
6135	ENSMUSG00000017747	Ghdc	0.52	3.61E-03	6.01E-03
6136	ENSMUSG00000031666	Rbl2	-0.46	3.63E-03	6.04E-03
6137	ENSMUSG00000041912	Tdrkh	-0.59	3.64E-03	6.06E-03
6138	ENSMUSG00000040907	Atp1a3	0.72	3.64E-03	6.06E-03
6139	ENSMUSG00000021772	Nkiras1	0.42	3.65E-03	6.08E-03
6140	ENSMUSG00000028703	Lrrc41	-0.41	3.65E-03	6.09E-03
6141	ENSMUSG00000040631	Dok4	-0.56	3.66E-03	6.09E-03
6142	ENSMUSG00000028577	Plaa	0.41	3.66E-03	6.09E-03
6143	ENSMUSG00000063108	Zfp26	-0.73	3.66E-03	6.10E-03
6144	ENSMUSG00000044452	Zfp507	-0.63	3.67E-03	6.10E-03
6145	ENSMUSG00000058318	Phf21a	-0.64	3.67E-03	6.10E-03
6146	ENSMUSG00000043881	Kbtbd7	-0.68	3.67E-03	6.11E-03
6147	ENSMUSG00000066640	Fbxl18	-0.63	3.68E-03	6.13E-03

6148	ENSMUSG00000027708	Dcun1d1	0.41	3.69E-03	6.13E-03
6149	ENSMUSG00000021595	Nsun2	-0.43	3.70E-03	6.15E-03
6150	ENSMUSG00000057982	Zfp809	-0.59	3.71E-03	6.16E-03
6151	ENSMUSG00000032727	Mier3	-0.66	3.71E-03	6.16E-03
6152	ENSMUSG00000020271	Fbxw11	-0.47	3.71E-03	6.17E-03
6153	ENSMUSG00000042557	Sin3a	-0.67	3.73E-03	6.20E-03
6154	ENSMUSG00000042050	Wdr60	0.59	3.73E-03	6.20E-03
6155	ENSMUSG00000037326	Capn15	-0.57	3.74E-03	6.21E-03
6156	ENSMUSG00000025178	Pi4k2a	-0.42	3.74E-03	6.22E-03
6157	ENSMUSG00000020537	Drg2	0.39	3.75E-03	6.22E-03
6158	ENSMUSG00000038344	Txlng	0.52	3.75E-03	6.22E-03
6159	ENSMUSG00000030022	Adamts9	-0.65	3.75E-03	6.23E-03
6160	ENSMUSG00000060950	Trmt61a	-0.56	3.76E-03	6.25E-03
6161	ENSMUSG00000009075	Cabp7	0.62	3.77E-03	6.25E-03
6162	ENSMUSG00000023259	Slc26a6	0.60	3.77E-03	6.25E-03
6163	ENSMUSG00000042419	Nfkbil1	-0.64	3.78E-03	6.27E-03
6164	ENSMUSG00000039157	Fam102a	-0.50	3.79E-03	6.28E-03
6165	ENSMUSG00000047368	Abhd17b	0.43	3.80E-03	6.30E-03
6166	ENSMUSG00000102961	Gm19918	-0.71	3.80E-03	6.30E-03
6167	ENSMUSG00000031148	Gpkow	-0.48	3.81E-03	6.32E-03
6168	ENSMUSG00000039218	Srrm2	-0.41	3.81E-03	6.32E-03
6169	ENSMUSG00000021428	Riok1	-0.67	3.82E-03	6.33E-03
6170	ENSMUSG00000078652	Psme3	0.41	3.82E-03	6.33E-03
6171	ENSMUSG00000030284	Creld1	-0.42	3.84E-03	6.36E-03
6172	ENSMUSG00000028138	Adh5	0.39	3.89E-03	6.44E-03
6173	ENSMUSG00000031171	Ftsj1	0.43	3.90E-03	6.46E-03
6174	ENSMUSG00000034799	Unc13a	-0.62	3.93E-03	6.51E-03
6175	ENSMUSG00000025860	Xiap	0.59	3.94E-03	6.52E-03
6176	ENSMUSG00000040389	Wdr47	-0.81	3.94E-03	6.53E-03
6177	ENSMUSG00000043940	Wdfy3	-0.52	3.97E-03	6.57E-03
6178	ENSMUSG00000040396	Abhd13	-0.46	3.97E-03	6.57E-03
6179	ENSMUSG00000020653	Klf11	-0.58	3.98E-03	6.59E-03
6180	ENSMUSG00000036281	Snapc4	-0.63	3.99E-03	6.60E-03
6181	ENSMUSG00000027162	Lin7c	-0.50	3.99E-03	6.61E-03
6182	ENSMUSG00000020747	Tmem94	0.41	3.99E-03	6.61E-03
6183	ENSMUSG00000046056	Sbsn	-0.75	4.00E-03	6.61E-03
6184	ENSMUSG00000004207	Psap	0.40	4.01E-03	6.63E-03
6185	ENSMUSG00000062202	Btbd9	-0.54	4.03E-03	6.66E-03
6186	ENSMUSG00000068739	Sars	0.40	4.03E-03	6.66E-03
6187	ENSMUSG00000020516	Rps6kb1	-0.45	4.04E-03	6.68E-03
6188	ENSMUSG00000060090	Rp2	-0.70	4.05E-03	6.68E-03
6189	ENSMUSG00000020432	Tcn2	-0.44	4.05E-03	6.69E-03
6190	ENSMUSG00000027649	Ctnnbl1	0.49	4.05E-03	6.69E-03
6191	ENSMUSG00000020956	Dtd2	0.43	4.06E-03	6.70E-03
6192	ENSMUSG00000031751	Amfr	-0.41	4.06E-03	6.71E-03
6193	ENSMUSG00000032187	Smarca4	-0.40	4.11E-03	6.78E-03
6194	ENSMUSG00000052798	Nup107	-0.78	4.11E-03	6.79E-03
6195	ENSMUSG00000028788	Ptp4a2	0.37	4.12E-03	6.80E-03
6196	ENSMUSG00000028243	Ubxn2b	-0.63	4.12E-03	6.80E-03
6197	ENSMUSG00000007036	Abhd16a	-0.40	4.12E-03	6.80E-03

6198	ENSMUSG00000020593	Lpin1	-0.44	4.13E-03	6.81E-03
6199	ENSMUSG00000053436	Mapk14	-0.44	4.13E-03	6.81E-03
6200	ENSMUSG00000024563	Smad2	-0.44	4.15E-03	6.84E-03
6201	ENSMUSG00000050052	Tdrp	-0.48	4.16E-03	6.85E-03
6202	ENSMUSG00000109727	Gm45464	-0.67	4.16E-03	6.86E-03
6203	ENSMUSG00000026617	Bpnt1	0.38	4.17E-03	6.87E-03
6204	ENSMUSG00000004056	Akt2	-0.44	4.17E-03	6.87E-03
6205	ENSMUSG00000002733	Plekha3	-0.49	4.17E-03	6.88E-03
6206	ENSMUSG00000040652	Oaz2	0.39	4.18E-03	6.88E-03
6207	ENSMUSG00000021000	Ctage5	-0.40	4.18E-03	6.89E-03
6208	ENSMUSG00000051615	Rap2a	-0.61	4.19E-03	6.90E-03
6209	ENSMUSG00000044795	Cyb5d1	-0.63	4.23E-03	6.97E-03
6210	ENSMUSG00000030314	Atg7	-0.41	4.24E-03	6.99E-03
6211	ENSMUSG00000015647	Lama5	-0.77	4.25E-03	7.00E-03
6212	ENSMUSG00000025544	Tm9sf2	-0.42	4.26E-03	7.02E-03
6213	ENSMUSG00000015597	Zfp318	-0.65	4.27E-03	7.03E-03
6214	ENSMUSG00000022204	Ngdn	0.49	4.28E-03	7.04E-03
6215	ENSMUSG00000030060	Hmces	-0.53	4.28E-03	7.05E-03
6216	ENSMUSG00000041765	Ubac2	0.42	4.31E-03	7.09E-03
6217	ENSMUSG00000037487	Ubr5	-0.45	4.32E-03	7.11E-03
6218	ENSMUSG00000026972	Arrdc1	-0.68	4.33E-03	7.12E-03
6219	ENSMUSG00000019916	P4ha1	0.51	4.35E-03	7.16E-03
6220	ENSMUSG00000002608	Ccdc97	-0.45	4.37E-03	7.19E-03
6221	ENSMUSG00000044433	Camsap3	0.65	4.37E-03	7.19E-03
6222	ENSMUSG00000022498	Txndc11	-0.47	4.39E-03	7.22E-03
6223	ENSMUSG00000041747	Utp15	-0.67	4.39E-03	7.22E-03
6224	ENSMUSG00000052748	Swt1	0.58	4.42E-03	7.26E-03
6225	ENSMUSG00000031935	Med17	-0.60	4.43E-03	7.27E-03
6226	ENSMUSG00000002763	Pex6	0.38	4.43E-03	7.28E-03
6227	ENSMUSG00000040043	Rbms2	-0.42	4.45E-03	7.30E-03
6228	ENSMUSG00000030545	Pex11a	0.42	4.45E-03	7.30E-03
6229	ENSMUSG00000021928	Ebpl	-0.57	4.48E-03	7.35E-03
6230	ENSMUSG00000020290	Xpo1	-0.49	4.48E-03	7.35E-03
6231	ENSMUSG00000030881	Arfip2	0.41	4.48E-03	7.36E-03
6232	ENSMUSG00000023277	Twf2	-0.56	4.49E-03	7.37E-03
6233	ENSMUSG00000075273	Ttc30b	0.46	4.50E-03	7.38E-03
6234	ENSMUSG00000032217	Rnf111	-0.51	4.50E-03	7.38E-03
6235	ENSMUSG00000033326	Kdm4a	-0.56	4.51E-03	7.40E-03
6236	ENSMUSG00000033499	Larp4b	-0.43	4.52E-03	7.40E-03
6237	ENSMUSG00000036983	Tfb1m	0.62	4.53E-03	7.43E-03
6238	ENSMUSG00000028896	Rcc1	0.54	4.56E-03	7.47E-03
6239	ENSMUSG00000028710	Atpaf1	0.46	4.57E-03	7.49E-03
6240	ENSMUSG00000029816	Gpnmb	-0.97	4.57E-03	7.49E-03
6241	ENSMUSG00000028643	Svbp	0.55	4.58E-03	7.50E-03
6242	ENSMUSG00000020255	D10Wsu102e	-0.66	4.58E-03	7.50E-03
6243	ENSMUSG00000023048	Prr13	0.52	4.58E-03	7.50E-03
6244	ENSMUSG00000067377	Tspan6	-0.56	4.58E-03	7.50E-03
6245	ENSMUSG00000006736	Tspan31	0.38	4.58E-03	7.50E-03
6246	ENSMUSG00000024270	Slc39a6	-0.71	4.59E-03	7.52E-03
6247	ENSMUSG00000024858	Grk2	-0.47	4.60E-03	7.53E-03

6248	ENSMUSG00000022586	Ly6i	-0.73	4.60E-03	7.53E-03
6249	ENSMUSG00000087515	Gm13554	-0.46	4.60E-03	7.53E-03
6250	ENSMUSG00000017969	Ptgis	0.65	4.62E-03	7.56E-03
6251	ENSMUSG00000040904	Gm21988	0.39	4.62E-03	7.56E-03
6252	ENSMUSG00000023971	Rrp36	0.48	4.64E-03	7.59E-03
6253	ENSMUSG00000019907	Ppp1r12a	-0.58	4.65E-03	7.60E-03
6254	ENSMUSG00000028559	Osbpl9	0.38	4.65E-03	7.61E-03
6255	ENSMUSG00000018995	Nars2	0.46	4.66E-03	7.62E-03
6256	ENSMUSG00000005413	Hmox1	-0.49	4.68E-03	7.64E-03
6257	ENSMUSG00000021013	Ttc8	-0.54	4.73E-03	7.72E-03
6258	ENSMUSG00000029141	Slc4a1ap	-0.60	4.73E-03	7.72E-03
6259	ENSMUSG00000006050	Sra1	0.39	4.74E-03	7.74E-03
6260	ENSMUSG00000012187	Mogat1	0.43	4.74E-03	7.74E-03
6261	ENSMUSG00000028670	Lypla2	0.40	4.76E-03	7.77E-03
6262	ENSMUSG00000035011	Zbtb7a	0.45	4.76E-03	7.77E-03
6263	ENSMUSG00000066036	Ubr4	0.44	4.77E-03	7.78E-03
6264	ENSMUSG00000028187	Rpf1	0.58	4.77E-03	7.78E-03
6265	ENSMUSG00000027223	Mapk8ip1	0.59	4.77E-03	7.79E-03
6266	ENSMUSG00000059486	Kbtbd2	-0.46	4.79E-03	7.82E-03
6267	ENSMUSG00000027742	Cog6	-0.41	4.80E-03	7.83E-03
6268	ENSMUSG00000029625	Cpsf4	0.56	4.80E-03	7.83E-03
6269	ENSMUSG00000076432	Ywhaq	-0.39	4.81E-03	7.85E-03
6270	ENSMUSG00000008604	Ubqln4	0.40	4.82E-03	7.86E-03
6271	ENSMUSG00000030265	Kras	0.44	4.83E-03	7.88E-03
6272	ENSMUSG00000006494	Pdk1	0.50	4.85E-03	7.91E-03
6273	ENSMUSG00000027293	Ehd4	-0.65	4.85E-03	7.91E-03
6274	ENSMUSG00000021703	Serinc5	-0.77	4.87E-03	7.93E-03
6275	ENSMUSG00000002992	Apoc2	0.58	4.88E-03	7.96E-03
6276	ENSMUSG00000040746	Rnf167	0.39	4.88E-03	7.96E-03
6277	ENSMUSG00000033658	Ddx19b	-0.58	4.89E-03	7.97E-03
6278	ENSMUSG00000042133	Ppig	0.55	4.89E-03	7.97E-03
6279	ENSMUSG00000079442	St6galnac4	-0.69	4.89E-03	7.97E-03
6280	ENSMUSG00000059475	Zfp426	-0.60	4.90E-03	7.97E-03
6281	ENSMUSG00000022680	Pdxdc1	-0.38	4.91E-03	7.99E-03
6282	ENSMUSG00000063317	Usp31	-0.62	4.91E-03	7.99E-03
6283	ENSMUSG00000035234	Fam175a	0.59	4.91E-03	8.00E-03
6284	ENSMUSG00000038895	Zfp653	-0.76	4.92E-03	8.00E-03
6285	ENSMUSG00000020794	Ube2g1	0.45	4.92E-03	8.00E-03
6286	ENSMUSG00000030298	Sec13	0.40	4.93E-03	8.02E-03
6287	ENSMUSG00000042350	Arel1	-0.44	4.93E-03	8.02E-03
6288	ENSMUSG00000005583	Mef2c	-0.70	4.93E-03	8.02E-03
6289	ENSMUSG00000032570	Atp2c1	-0.43	4.94E-03	8.03E-03
6290	ENSMUSG00000107012	4930528J11Rik	-0.64	4.96E-03	8.06E-03
6291	ENSMUSG00000033554	Dph5	0.50	4.97E-03	8.08E-03
6292	ENSMUSG00000030546	Plin1	0.42	4.98E-03	8.10E-03
6293	ENSMUSG00000054836	Elp6	0.55	4.99E-03	8.11E-03
6294	ENSMUSG00000046861	Hectd3	-0.41	5.01E-03	8.14E-03
6295	ENSMUSG00000020923	Ubtf	-0.42	5.03E-03	8.16E-03
6296	ENSMUSG00000086149	Gm15598	-0.65	5.03E-03	8.16E-03
6297	ENSMUSG00000056310	Tyw1	-0.43	5.03E-03	8.17E-03

6298	ENSMUSG00000051306	Usp42	-0.66	5.04E-03	8.18E-03
6299	ENSMUSG00000031422	Morf4l2	-0.39	5.05E-03	8.19E-03
6300	ENSMUSG00000051890	Klhdc1	-0.66	5.05E-03	8.20E-03
6301	ENSMUSG00000030421	Uri1	-0.49	5.05E-03	8.20E-03
6302	ENSMUSG00000015980	Lrrc27	0.44	5.07E-03	8.23E-03
6303	ENSMUSG00000037253	Mex3c	-0.59	5.08E-03	8.24E-03
6304	ENSMUSG00000010095	Slc3a2	-0.38	5.08E-03	8.24E-03
6305	ENSMUSG00000041594	Tmtc4	-0.60	5.11E-03	8.29E-03
6306	ENSMUSG00000092417	Gpank1	0.67	5.12E-03	8.30E-03
6307	ENSMUSG00000069255	Dusp22	0.39	5.13E-03	8.31E-03
6308	ENSMUSG00000029787	Avl9	-0.54	5.14E-03	8.33E-03
6309	ENSMUSG00000020610	Amz2	0.39	5.14E-03	8.33E-03
6310	ENSMUSG00000044350	Lacc1	-0.64	5.16E-03	8.36E-03
6311	ENSMUSG00000029125	Stx18	0.41	5.18E-03	8.39E-03
6312	ENSMUSG00000056770	Setd3	-0.38	5.19E-03	8.40E-03
6313	ENSMUSG00000041654	Slc39a11	-0.52	5.22E-03	8.46E-03
6314	ENSMUSG00000070934	Rraga	0.45	5.23E-03	8.46E-03
6315	ENSMUSG00000051351	Zfp46	-0.59	5.23E-03	8.47E-03
6316	ENSMUSG00000001175	Calm1	0.41	5.24E-03	8.49E-03
6317	ENSMUSG00000028932	Psmc2	0.38	5.26E-03	8.51E-03
6318	ENSMUSG00000031379	Pir	0.51	5.27E-03	8.52E-03
6319	ENSMUSG00000025857	Dnaaf5	-0.47	5.28E-03	8.55E-03
6320	ENSMUSG00000025785	Exosc7	0.48	5.30E-03	8.57E-03
6321	ENSMUSG00000054690	Emcn	-0.62	5.30E-03	8.58E-03
6322	ENSMUSG00000073139	Tmem185a	-0.41	5.32E-03	8.60E-03
6323	ENSMUSG00000068040	Tm9sf4	-0.43	5.32E-03	8.61E-03
6324	ENSMUSG00000053289	Ddx10	-0.59	5.32E-03	8.61E-03
6325	ENSMUSG00000003528	Slc25a1	0.52	5.32E-03	8.61E-03
6326	ENSMUSG00000006705	Pknox1	-0.54	5.33E-03	8.62E-03
6327	ENSMUSG00000024937	Ehbp1l1	-0.40	5.37E-03	8.68E-03
6328	ENSMUSG00000028292	Rars2	0.40	5.39E-03	8.71E-03
6329	ENSMUSG00000029366	Dck	-0.67	5.43E-03	8.77E-03
6330	ENSMUSG00000030199	Etv6	-0.51	5.44E-03	8.80E-03
6331	ENSMUSG00000038807	Rap1gap2	-0.69	5.45E-03	8.80E-03
6332	ENSMUSG00000038957	Edc3	-0.42	5.45E-03	8.80E-03
6333	ENSMUSG00000020495	Smg8	-0.58	5.45E-03	8.80E-03
6334	ENSMUSG00000034175	Rhbdd3	0.39	5.46E-03	8.81E-03
6335	ENSMUSG00000031153	Gripap1	-0.57	5.47E-03	8.83E-03
6336	ENSMUSG00000000916	Nsun5	0.45	5.48E-03	8.85E-03
6337	ENSMUSG00000041936	Agrn	-0.58	5.49E-03	8.87E-03
6338	ENSMUSG00000027425	Csrp2bp	-0.48	5.53E-03	8.93E-03
6339	ENSMUSG00000056579	Tug1	-0.54	5.56E-03	8.96E-03
6340	ENSMUSG00000030924	2610020H08Rik	-0.60	5.57E-03	8.98E-03
6341	ENSMUSG00000071645	Tut1	-0.51	5.64E-03	9.10E-03
6342	ENSMUSG00000049606	Zfp644	0.52	5.66E-03	9.13E-03
6343	ENSMUSG00000112241	Sumo3	-0.44	5.70E-03	9.19E-03
6344	ENSMUSG00000006378	Gcat	0.40	5.72E-03	9.21E-03
6345	ENSMUSG00000024422	Dhx16	-0.44	5.72E-03	9.21E-03
6346	ENSMUSG00000092609	Gm20481	0.62	5.74E-03	9.25E-03
6347	ENSMUSG00000015474	Ppt2	0.41	5.74E-03	9.25E-03

6348	ENSMUSG00000030935	Acsm3	0.54	5.75E-03	9.26E-03
6349	ENSMUSG00000034667	Xpot	-0.41	5.78E-03	9.32E-03
6350	ENSMUSG00000024325	Ring1	-0.53	5.79E-03	9.32E-03
6351	ENSMUSG00000040888	Gfer	0.42	5.83E-03	9.39E-03
6352	ENSMUSG00000016253	Nelfcd	-0.40	5.83E-03	9.39E-03
6353	ENSMUSG00000002814	Top3a	-0.69	5.84E-03	9.39E-03
6354	ENSMUSG00000031787	Katnb1	-0.44	5.84E-03	9.40E-03
6355	ENSMUSG00000019256	Ahr	-0.77	5.85E-03	9.42E-03
6356	ENSMUSG00000018379	Srsf1	-0.42	5.88E-03	9.46E-03
6357	ENSMUSG00000029681	Bcl7b	0.39	5.88E-03	9.46E-03
6358	ENSMUSG00000020034	Tcp11l2	-0.52	5.90E-03	9.49E-03
6359	ENSMUSG00000038240	Pdss2	0.52	5.90E-03	9.49E-03
6360	ENSMUSG00000031530	Dusp4	-0.41	5.94E-03	9.55E-03
6361	ENSMUSG00000030089	Slc41a3	-0.64	5.94E-03	9.55E-03
6362	ENSMUSG00000021432	Slc35b3	-0.64	5.94E-03	9.55E-03
6363	ENSMUSG00000034563	Ccpg1	-0.37	5.95E-03	9.56E-03
6364	ENSMUSG00000030471	Zdhhc13	-0.58	5.97E-03	9.59E-03
6365	ENSMUSG00000021257	Angel1	-0.69	5.97E-03	9.59E-03
6366	ENSMUSG00000022472	Desi1	-0.48	5.98E-03	9.61E-03
6367	ENSMUSG00000025737	Wdr24	-0.48	5.99E-03	9.61E-03
6368	ENSMUSG00000039202	Abhd2	-0.70	5.99E-03	9.62E-03
6369	ENSMUSG00000031976	Urb2	-0.69	6.00E-03	9.64E-03
6370	ENSMUSG00000050174	Nudt6	0.50	6.01E-03	9.64E-03
6371	ENSMUSG00000035958	Tdp2	0.61	6.02E-03	9.67E-03
6372	ENSMUSG00000039478	Micu3	0.63	6.03E-03	9.68E-03
6373	ENSMUSG00000029763	Exoc4	-0.54	6.04E-03	9.68E-03
6374	ENSMUSG00000001300	Efnb2	-0.63	6.05E-03	9.71E-03
6375	ENSMUSG00000012429	Mplkip	0.57	6.07E-03	9.73E-03
6376	ENSMUSG00000045038	Prkce	-0.45	6.09E-03	9.77E-03
6377	ENSMUSG00000030718	Ppme1	0.41	6.10E-03	9.78E-03
6378	ENSMUSG00000003779	Kif20a	-0.70	6.11E-03	9.80E-03
6379	ENSMUSG00000026737	Pip4k2a	-0.65	6.13E-03	9.82E-03
6380	ENSMUSG00000074213	Gm10642	0.50	6.13E-03	9.82E-03
6381	ENSMUSG00000016757	Ttll12	-0.49	6.18E-03	9.90E-03
6382	ENSMUSG00000006732	Mettl1	0.57	6.19E-03	9.91E-03
6383	ENSMUSG00000038170	Pde4dip	-0.38	6.19E-03	9.92E-03
6384	ENSMUSG00000025571	Tnrc6c	-0.64	6.23E-03	9.98E-03
6385	ENSMUSG00000015023	Ddx19a	-0.47	6.25E-03	1.00E-02
6386	ENSMUSG00000023110	Prmt5	-0.41	6.25E-03	1.00E-02
6387	ENSMUSG00000087644	Gm14703	0.57	6.26E-03	1.00E-02
6388	ENSMUSG00000085714	Gm13008	-0.52	6.27E-03	1.00E-02
6389	ENSMUSG00000041740	Rnf10	0.38	6.27E-03	1.00E-02
6390	ENSMUSG00000000340	Dbt	0.36	6.28E-03	1.01E-02
6391	ENSMUSG00000029598	Plbd2	-0.36	6.29E-03	1.01E-02
6392	ENSMUSG00000107881	Gm44250	0.58	6.29E-03	1.01E-02
6393	ENSMUSG00000005804	Bloc1s6	0.42	6.31E-03	1.01E-02
6394	ENSMUSG00000031167	Rbm3	-0.40	6.33E-03	1.01E-02
6395	ENSMUSG00000027601	Mtfr1	0.40	6.34E-03	1.01E-02
6396	ENSMUSG00000001157	Gmcl1	-0.54	6.36E-03	1.02E-02
6397	ENSMUSG00000026142	Rhbdd1	-0.51	6.37E-03	1.02E-02

6398	ENSMUSG00000025025	Mxi1	0.45	6.38E-03	1.02E-02
6399	ENSMUSG00000041623	D11Wsu47e	-0.61	6.39E-03	1.02E-02
6400	ENSMUSG00000037935	Smarce1	-0.48	6.39E-03	1.02E-02
6401	ENSMUSG00000086448	9330162012Rik	-0.58	6.39E-03	1.02E-02
6402	ENSMUSG00000030407	Qpctl	-0.42	6.40E-03	1.02E-02
6403	ENSMUSG00000029376	Mthfd2l	0.52	6.41E-03	1.02E-02
6404	ENSMUSG00000027502	Rtfdc1	0.36	6.41E-03	1.02E-02
6405	ENSMUSG00000027881	Prpf38b	-0.45	6.41E-03	1.02E-02
6406	ENSMUSG00000024539	Ptpn2	0.51	6.42E-03	1.02E-02
6407	ENSMUSG00000063895	Nupl1	-0.58	6.42E-03	1.03E-02
6408	ENSMUSG00000013846	St3gal1	-0.56	6.42E-03	1.03E-02
6409	ENSMUSG00000001674	Ddx18	-0.53	6.46E-03	1.03E-02
6410	ENSMUSG00000010517	Faf1	0.39	6.49E-03	1.04E-02
6411	ENSMUSG00000107608	Gm43863	-0.48	6.56E-03	1.05E-02
6412	ENSMUSG00000020641	Rsad2	-0.67	6.59E-03	1.05E-02
6413	ENSMUSG00000044700	Tmem201	-0.41	6.59E-03	1.05E-02
6414	ENSMUSG00000022858	Tra2b	-0.39	6.60E-03	1.05E-02
6415	ENSMUSG00000056216	Cebpg	0.39	6.60E-03	1.05E-02
6416	ENSMUSG00000038312	Edem2	-0.40	6.67E-03	1.06E-02
6417	ENSMUSG00000070462	Mesdc1	-0.51	6.68E-03	1.06E-02
6418	ENSMUSG00000057375	Yipf1	0.40	6.68E-03	1.06E-02
6419	ENSMUSG00000041718	Alg13	0.63	6.69E-03	1.07E-02
6420	ENSMUSG00000093989	Rnasek	0.37	6.70E-03	1.07E-02
6421	ENSMUSG00000028869	Gnl2	-0.48	6.75E-03	1.07E-02
6422	ENSMUSG00000008575	Nfib	-0.37	6.77E-03	1.08E-02
6423	ENSMUSG00000096954	Gdap10	-0.61	6.79E-03	1.08E-02
6424	ENSMUSG00000001248	Gramd1a	-0.45	6.81E-03	1.08E-02
6425	ENSMUSG00000048878	Hexim1	0.72	6.81E-03	1.08E-02
6426	ENSMUSG00000026749	Nek6	0.36	6.83E-03	1.09E-02
6427	ENSMUSG00000028403	Zdhhc21	-0.57	6.84E-03	1.09E-02
6428	ENSMUSG00000020115	Tbk1	-0.46	6.85E-03	1.09E-02
6429	ENSMUSG00000039178	Tbc1d19	-0.54	6.87E-03	1.09E-02
6430	ENSMUSG00000015757	Ppil4	-0.50	6.87E-03	1.09E-02
6431	ENSMUSG00000030815	Phkg2	0.43	6.92E-03	1.10E-02
6432	ENSMUSG00000022507	1810013L24Rik	-0.45	6.95E-03	1.11E-02
6433	ENSMUSG00000032527	Pccb	-0.41	6.95E-03	1.11E-02
6434	ENSMUSG00000074807	Gm10762	0.60	6.97E-03	1.11E-02
6435	ENSMUSG00000040715	Rsc1a1	-0.45	6.98E-03	1.11E-02
6436	ENSMUSG00000038244	Mical2	0.58	6.98E-03	1.11E-02
6437	ENSMUSG00000029345	Tfip11	-0.41	7.01E-03	1.11E-02
6438	ENSMUSG00000030680	Pagr1a	0.50	7.01E-03	1.11E-02
6439	ENSMUSG00000043415	Otud1	-0.63	7.02E-03	1.12E-02
6440	ENSMUSG00000042810	Krba1	-0.63	7.07E-03	1.12E-02
6441	ENSMUSG00000055435	Maf	-0.70	7.08E-03	1.12E-02
6442	ENSMUSG00000027433	Xrn2	-0.45	7.09E-03	1.13E-02
6443	ENSMUSG00000021831	Ero1l	0.46	7.12E-03	1.13E-02
6444	ENSMUSG00000048827	Pkd1l3	0.53	7.13E-03	1.13E-02
6445	ENSMUSG00000032407	U2surp	0.42	7.15E-03	1.14E-02
6446	ENSMUSG00000039205	Ciz1	-0.50	7.16E-03	1.14E-02
6447	ENSMUSG00000027540	Ptpn1	-0.44	7.16E-03	1.14E-02

6448	ENSMUSG00000040669	Phc1	-0.66	7.16E-03	1.14E-02
6449	ENSMUSG00000059439	Bcas3	-0.37	7.18E-03	1.14E-02
6450	ENSMUSG00000037752	Xkr8	-0.70	7.18E-03	1.14E-02
6451	ENSMUSG00000063849	Ppcdc	0.46	7.19E-03	1.14E-02
6452	ENSMUSG00000086995	Gm13544	-0.62	7.21E-03	1.14E-02
6453	ENSMUSG00000038644	Pold1	-0.67	7.21E-03	1.14E-02
6454	ENSMUSG00000033382	Trappc8	-0.48	7.24E-03	1.15E-02
6455	ENSMUSG00000025037	Maoa	-0.44	7.28E-03	1.15E-02
6456	ENSMUSG00000001366	Fbxo9	-0.37	7.30E-03	1.16E-02
6457	ENSMUSG00000039000	Ube3c	-0.43	7.30E-03	1.16E-02
6458	ENSMUSG00000040447	Spns2	-0.62	7.31E-03	1.16E-02
6459	ENSMUSG00000046962	Zbtb21	-0.59	7.33E-03	1.16E-02
6460	ENSMUSG00000044098	Rsbn1	-0.59	7.34E-03	1.16E-02
6461	ENSMUSG00000039824	Myl6b	-0.79	7.34E-03	1.16E-02
6462	ENSMUSG00000001440	Kpnb1	-0.42	7.39E-03	1.17E-02
6463	ENSMUSG00000092405	Gm20402	-0.49	7.42E-03	1.17E-02
6464	ENSMUSG00000041625	Ggact	0.53	7.44E-03	1.18E-02
6465	ENSMUSG00000021537	Cetn3	0.48	7.44E-03	1.18E-02
6466	ENSMUSG00000027954	Efna1	-0.64	7.44E-03	1.18E-02
6467	ENSMUSG00000035401	Emsy	-0.57	7.45E-03	1.18E-02
6468	ENSMUSG00000107068	Gm42742	0.50	7.45E-03	1.18E-02
6469	ENSMUSG00000058440	Nrf1	-0.50	7.46E-03	1.18E-02
6470	ENSMUSG00000084899	Gm15344	-0.48	7.49E-03	1.18E-02
6471	ENSMUSG00000037149	Ddx1	-0.36	7.49E-03	1.18E-02
6472	ENSMUSG00000022545	Ercc4	-0.54	7.51E-03	1.19E-02
6473	ENSMUSG00000024797	Vps51	0.39	7.51E-03	1.19E-02
6474	ENSMUSG00000063568	Jazf1	-0.53	7.52E-03	1.19E-02
6475	ENSMUSG00000039065	Fam173b	0.47	7.54E-03	1.19E-02
6476	ENSMUSG00000010362	Rdm1	0.46	7.55E-03	1.19E-02
6477	ENSMUSG00000020224	Llph	0.54	7.56E-03	1.19E-02
6478	ENSMUSG00000002308	Cd320	0.38	7.58E-03	1.20E-02
6479	ENSMUSG00000029817	Tra2a	-0.60	7.60E-03	1.20E-02
6480	ENSMUSG00000018322	Tomm34	-0.48	7.60E-03	1.20E-02
6481	ENSMUSG00000034473	Sec22a	0.39	7.61E-03	1.20E-02
6482	ENSMUSG00000085414	Sspnos	-0.56	7.62E-03	1.20E-02
6483	ENSMUSG00000034075	Zdhhc5	-0.40	7.65E-03	1.21E-02
6484	ENSMUSG00000078762	Haus5	-0.72	7.65E-03	1.21E-02
6485	ENSMUSG00000018848	Rars	-0.43	7.66E-03	1.21E-02
6486	ENSMUSG00000038831	Ralgps1	-0.61	7.68E-03	1.21E-02
6487	ENSMUSG00000073787	Gm10575	0.52	7.68E-03	1.21E-02
6488	ENSMUSG00000034525	Ice1	-0.59	7.68E-03	1.21E-02
6489	ENSMUSG00000035181	Heatr5a	-0.70	7.70E-03	1.21E-02
6490	ENSMUSG00000038646	Fam103a1	0.45	7.70E-03	1.21E-02
6491	ENSMUSG00000036737	Oxsr1	-0.46	7.71E-03	1.21E-02
6492	ENSMUSG00000037458	Azin1	-0.43	7.72E-03	1.22E-02
6493	ENSMUSG00000028042	Zbtb7b	0.43	7.75E-03	1.22E-02
6494	ENSMUSG00000066148	Prpf4	-0.52	7.76E-03	1.22E-02
6495	ENSMUSG00000022661	Cd200	-0.48	7.78E-03	1.23E-02
6496	ENSMUSG00000031684	Slc10a7	-0.59	7.80E-03	1.23E-02
6497	ENSMUSG00000045980	Tmem104	-0.44	7.81E-03	1.23E-02

6498	ENSMUSG00000024030	Abcg1	-0.64	7.82E-03	1.23E-02
6499	ENSMUSG00000019873	Reep3	0.36	7.86E-03	1.24E-02
6500	ENSMUSG00000038836	Agbl3	0.55	7.87E-03	1.24E-02
6501	ENSMUSG00000026740	Dnajc1	-0.52	7.87E-03	1.24E-02
6502	ENSMUSG00000019518	Ap4m1	-0.43	7.88E-03	1.24E-02
6503	ENSMUSG00000030107	Usp18	0.62	7.89E-03	1.24E-02
6504	ENSMUSG00000031939	Taf1d	0.55	7.90E-03	1.24E-02
6505	ENSMUSG00000032477	Cdc25a	0.55	7.90E-03	1.24E-02
6506	ENSMUSG00000020831	0610010K14Rik	0.44	7.90E-03	1.24E-02
6507	ENSMUSG00000109901	Chmp1b	-0.39	7.93E-03	1.25E-02
6508	ENSMUSG00000020326	Ccng1	0.41	7.94E-03	1.25E-02
6509	ENSMUSG00000063362	Alg11	-0.53	7.95E-03	1.25E-02
6510	ENSMUSG00000029071	Dvl1	-0.39	7.96E-03	1.25E-02
6511	ENSMUSG00000105195	Gm43584	-0.66	7.96E-03	1.25E-02
6512	ENSMUSG00000019505	Ubb	-0.37	7.97E-03	1.25E-02
6513	ENSMUSG00000031979	Cog2	-0.43	8.00E-03	1.26E-02
6514	ENSMUSG00000032834	Pwp2	-0.52	8.02E-03	1.26E-02
6515	ENSMUSG00000052833	Sae1	-0.41	8.06E-03	1.27E-02
6516	ENSMUSG00000022184	Fbxo4	-0.44	8.07E-03	1.27E-02
6517	ENSMUSG00000018126	Baiap2l2	-0.65	8.09E-03	1.27E-02
6518	ENSMUSG00000030663	1110004F10Rik	0.37	8.09E-03	1.27E-02
6519	ENSMUSG00000074746	Pdzd8	-0.51	8.10E-03	1.27E-02
6520	ENSMUSG00000071650	Ganab	-0.38	8.17E-03	1.28E-02
6521	ENSMUSG00000013465	Nelfb	-0.39	8.19E-03	1.29E-02
6522	ENSMUSG00000035443	Thyn1	0.47	8.21E-03	1.29E-02
6523	ENSMUSG00000020640	Itsn2	-0.47	8.21E-03	1.29E-02
6524	ENSMUSG00000023153	Tmem52	-0.60	8.23E-03	1.29E-02
6525	ENSMUSG00000029771	Irf5	-0.57	8.25E-03	1.29E-02
6526	ENSMUSG00000075703	Selenoi	-0.48	8.26E-03	1.29E-02
6527	ENSMUSG00000029073	Cptp	-0.43	8.26E-03	1.30E-02
6528	ENSMUSG00000021895	Arhgef3	-0.58	8.30E-03	1.30E-02
6529	ENSMUSG00000036820	Amdhd2	0.41	8.33E-03	1.30E-02
6530	ENSMUSG00000041570	Camsap2	-0.49	8.34E-03	1.31E-02
6531	ENSMUSG00000035629	Rubcn	-0.48	8.34E-03	1.31E-02
6532	ENSMUSG00000051510	Mafg	-0.42	8.37E-03	1.31E-02
6533	ENSMUSG00000022914	Brwd1	0.44	8.43E-03	1.32E-02
6534	ENSMUSG00000020092	Pald1	0.38	8.44E-03	1.32E-02
6535	ENSMUSG00000038039	Gcc2	-0.66	8.45E-03	1.32E-02
6536	ENSMUSG00000035354	Uvrag	-0.44	8.45E-03	1.32E-02
6537	ENSMUSG00000003575	Crtc1	-0.66	8.45E-03	1.32E-02
6538	ENSMUSG00000028034	Fubp1	-0.43	8.46E-03	1.32E-02
6539	ENSMUSG00000041438	Utp4	-0.37	8.49E-03	1.33E-02
6540	ENSMUSG00000030243	Recql	-0.52	8.50E-03	1.33E-02
6541	ENSMUSG00000026213	Stk11ip	-0.56	8.51E-03	1.33E-02
6542	ENSMUSG00000062937	Mtap	-0.41	8.54E-03	1.33E-02
6543	ENSMUSG00000028394	Pole3	0.40	8.54E-03	1.34E-02
6544	ENSMUSG00000021751	Acox2	0.71	8.54E-03	1.34E-02
6545	ENSMUSG00000033257	Ttll4	-0.60	8.56E-03	1.34E-02
6546	ENSMUSG00000020264	Slc36a2	0.37	8.57E-03	1.34E-02
6547	ENSMUSG00000031667	Aktip	0.35	8.58E-03	1.34E-02

6548	ENSMUSG00000044224	Dnajc21	0.44	8.58E-03	1.34E-02
6549	ENSMUSG00000020496	Rnf187	0.34	8.61E-03	1.34E-02
6550	ENSMUSG00000021460	Auh	0.35	8.61E-03	1.34E-02
6551	ENSMUSG00000028409	Smu1	0.36	8.62E-03	1.35E-02
6552	ENSMUSG00000021670	Hmgcr	-0.60	8.63E-03	1.35E-02
6553	ENSMUSG00000018428	Akap1	0.39	8.63E-03	1.35E-02
6554	ENSMUSG00000030180	Kdm5a	-0.44	8.64E-03	1.35E-02
6555	ENSMUSG00000090523	Gypc	-0.55	8.66E-03	1.35E-02
6556	ENSMUSG00000054008	Ndst1	-0.42	8.68E-03	1.35E-02
6557	ENSMUSG00000038500	Prr3	-0.57	8.69E-03	1.35E-02
6558	ENSMUSG00000030816	Rnf40	-0.39	8.71E-03	1.36E-02
6559	ENSMUSG00000089865	Gm44503	-0.35	8.73E-03	1.36E-02
6560	ENSMUSG00000057406	Nsd2	-0.63	8.74E-03	1.36E-02
6561	ENSMUSG00000055531	Cpsf6	-0.48	8.75E-03	1.36E-02
6562	ENSMUSG00000075419	Dolk	-0.36	8.76E-03	1.36E-02
6563	ENSMUSG00000062376	Borcs7	0.43	8.76E-03	1.37E-02
6564	ENSMUSG00000053510	Nrd1	-0.35	8.79E-03	1.37E-02
6565	ENSMUSG00000060036	Rpl3	0.37	8.79E-03	1.37E-02
6566	ENSMUSG00000021264	Yy1	0.53	8.80E-03	1.37E-02
6567	ENSMUSG00000022841	Ap2m1	0.34	8.89E-03	1.38E-02
6568	ENSMUSG00000026888	Grb14	0.45	8.89E-03	1.38E-02
6569	ENSMUSG00000052609	Plekhg3	-0.65	8.89E-03	1.38E-02
6570	ENSMUSG00000015217	Hmgb3	0.49	8.89E-03	1.38E-02
6571	ENSMUSG00000029676	Pot1a	-0.59	8.92E-03	1.39E-02
6572	ENSMUSG00000037197	Rbm17	0.42	8.99E-03	1.40E-02
6573	ENSMUSG00000030678	Maz	-0.49	9.01E-03	1.40E-02
6574	ENSMUSG00000032392	Parp16	-0.49	9.03E-03	1.40E-02
6575	ENSMUSG00000042105	Inpp5f	-0.57	9.05E-03	1.41E-02
6576	ENSMUSG00000002059	Rab34	-0.34	9.06E-03	1.41E-02
6577	ENSMUSG00000024969	Mark2	-0.35	9.08E-03	1.41E-02
6578	ENSMUSG00000035078	Mtmr9	-0.50	9.18E-03	1.43E-02
6579	ENSMUSG00000051396	Gm45902	-0.51	9.22E-03	1.43E-02
6580	ENSMUSG00000029440	Psmd9	0.39	9.25E-03	1.44E-02
6581	ENSMUSG00000027455	Nsfl1c	0.36	9.25E-03	1.44E-02
6582	ENSMUSG00000021916	Glt8d1	-0.54	9.33E-03	1.45E-02
6583	ENSMUSG00000029128	Rab28	0.44	9.38E-03	1.46E-02
6584	ENSMUSG00000017615	Tnfaip1	-0.37	9.39E-03	1.46E-02
6585	ENSMUSG00000022241	Tars	-0.40	9.42E-03	1.46E-02
6586	ENSMUSG00000034445	Cyb561a3	-0.48	9.43E-03	1.46E-02
6587	ENSMUSG00000004561	Mettl17	0.53	9.44E-03	1.47E-02
6588	ENSMUSG00000090860	Gm17134	-0.49	9.45E-03	1.47E-02
6589	ENSMUSG00000021500	Ddx46	0.47	9.45E-03	1.47E-02
6590	ENSMUSG00000032827	Ppp1r9a	-0.67	9.46E-03	1.47E-02
6591	ENSMUSG00000055407	Map6	-0.50	9.51E-03	1.48E-02
6592	ENSMUSG00000048874	Phf3	-0.48	9.52E-03	1.48E-02
6593	ENSMUSG00000090952	Gm17251	0.42	9.53E-03	1.48E-02
6594	ENSMUSG00000043061	Tmem18	-0.45	9.53E-03	1.48E-02
6595	ENSMUSG00000006782	Cnp	-0.42	9.59E-03	1.49E-02
6596	ENSMUSG00000062040	Zfp27	0.60	9.62E-03	1.49E-02
6597	ENSMUSG00000048027	Rgmb	-0.49	9.70E-03	1.50E-02

6598	ENSMUSG00000107659	Gm44170	-0.39	9.72E-03	1.51E-02
6599	ENSMUSG00000042647	Acad12	-0.38	9.74E-03	1.51E-02
6600	ENSMUSG00000097177	9330159M07Rik	0.44	9.74E-03	1.51E-02
6601	ENSMUSG00000024238	Zeb1	-0.51	9.74E-03	1.51E-02
6602	ENSMUSG00000021792	Fam213a	0.34	9.77E-03	1.51E-02
6603	ENSMUSG00000032353	Tmed3	-0.37	9.79E-03	1.52E-02
6604	ENSMUSG00000030556	Lrrc28	0.48	9.82E-03	1.52E-02
6605	ENSMUSG00000026864	Hspa5	-0.35	9.85E-03	1.52E-02
6606	ENSMUSG00000034201	Gas2l1	-0.36	9.85E-03	1.52E-02
6607	ENSMUSG00000040272	Accs	-0.59	9.85E-03	1.52E-02
6608	ENSMUSG00000031532	Saraf	0.33	9.86E-03	1.53E-02
6609	ENSMUSG00000062906	Hdac10	-0.42	9.88E-03	1.53E-02
6610	ENSMUSG00000024382	Ercc3	-0.54	9.88E-03	1.53E-02
6611	ENSMUSG00000042303	Sgsm3	-0.50	9.89E-03	1.53E-02
6612	ENSMUSG00000026849	Tor1a	0.37	9.94E-03	1.54E-02
6613	ENSMUSG00000021115	Vrk1	-0.56	9.95E-03	1.54E-02
6614	ENSMUSG00000046280	She	-0.55	9.97E-03	1.54E-02
6615	ENSMUSG00000037533	Rapgef6	-0.54	9.97E-03	1.54E-02
6616	ENSMUSG00000032512	Wdr48	-0.40	1.00E-02	1.55E-02
6617	ENSMUSG00000049439	Cyp20a1	-0.62	1.01E-02	1.55E-02
6618	ENSMUSG00000036585	Fgf1	0.47	1.01E-02	1.56E-02
6619	ENSMUSG00000025612	Bach1	-0.71	1.01E-02	1.56E-02
6620	ENSMUSG00000024687	Osbp	-0.38	1.01E-02	1.56E-02
6621	ENSMUSG00000037818	Abhd18	0.48	1.02E-02	1.57E-02
6622	ENSMUSG00000070319	Eif3g	0.38	1.02E-02	1.58E-02
6623	ENSMUSG00000050017	Pitpnb	-0.37	1.02E-02	1.58E-02
6624	ENSMUSG00000026281	Dtymk	0.46	1.02E-02	1.58E-02
6625	ENSMUSG00000019818	Cd164	-0.40	1.03E-02	1.59E-02
6626	ENSMUSG00000029179	Zcchc4	0.54	1.03E-02	1.59E-02
6627	ENSMUSG00000028124	Gclm	0.35	1.03E-02	1.59E-02
6628	ENSMUSG00000029640	Usp12	-0.47	1.03E-02	1.59E-02
6629	ENSMUSG00000054237	Fra10ac1	0.52	1.04E-02	1.60E-02
6630	ENSMUSG00000057691	Zfp746	-0.47	1.04E-02	1.60E-02
6631	ENSMUSG00000097331	F420014N23Rik	-0.62	1.04E-02	1.61E-02
6632	ENSMUSG00000019889	Ptprk	-0.70	1.05E-02	1.61E-02
6633	ENSMUSG00000021427	Ssr1	-0.42	1.05E-02	1.62E-02
6634	ENSMUSG00000015714	Cers2	-0.36	1.05E-02	1.62E-02
6635	ENSMUSG00000014959	Gorasp2	-0.34	1.05E-02	1.62E-02
6636	ENSMUSG00000097410	Gm26668	-0.56	1.06E-02	1.63E-02
6637	ENSMUSG00000031862	Atp13a1	-0.41	1.06E-02	1.63E-02
6638	ENSMUSG00000071652	Ints5	0.36	1.06E-02	1.63E-02
6639	ENSMUSG00000047153	Khnyn	-0.58	1.06E-02	1.63E-02
6640	ENSMUSG00000042700	Sipa1l1	-0.45	1.07E-02	1.64E-02
6641	ENSMUSG00000066258	Trim12a	-0.57	1.07E-02	1.64E-02
6642	ENSMUSG00000000693	Loxl3	0.44	1.07E-02	1.64E-02
6643	ENSMUSG00000032557	Uba5	-0.44	1.07E-02	1.65E-02
6644	ENSMUSG00000028990	Lzic	0.40	1.07E-02	1.65E-02
6645	ENSMUSG00000015002	Efr3a	-0.38	1.08E-02	1.66E-02
6646	ENSMUSG00000018449	Rpain	0.52	1.08E-02	1.66E-02
6647	ENSMUSG00000035757	Selenoo	0.35	1.08E-02	1.66E-02

6648	ENSMUSG00000085148	Mir22hg	0.39	1.08E-02	1.66E-02
6649	ENSMUSG00000032431	Crtap	-0.36	1.08E-02	1.66E-02
6650	ENSMUSG00000035954	Dock4	-0.73	1.08E-02	1.66E-02
6651	ENSMUSG00000041346	Wrap53	0.52	1.08E-02	1.66E-02
6652	ENSMUSG00000036568	Gltscr1l	-0.57	1.08E-02	1.66E-02
6653	ENSMUSG00000085802	Gm16059	-0.62	1.08E-02	1.67E-02
6654	ENSMUSG00000054931	Zkscan4	-0.60	1.09E-02	1.67E-02
6655	ENSMUSG00000002546	Golga2	-0.42	1.09E-02	1.67E-02
6656	ENSMUSG00000024824	Rad9a	0.36	1.09E-02	1.68E-02
6657	ENSMUSG00000018661	Cog1	-0.39	1.09E-02	1.68E-02
6658	ENSMUSG00000014470	Rnf166	0.37	1.09E-02	1.68E-02
6659	ENSMUSG00000033126	Ybey	0.55	1.10E-02	1.68E-02
6660	ENSMUSG00000028344	Invs	-0.54	1.10E-02	1.68E-02
6661	ENSMUSG00000042650	Alkbh5	-0.37	1.10E-02	1.69E-02
6662	ENSMUSG00000049562	Ap5b1	-0.59	1.10E-02	1.69E-02
6663	ENSMUSG00000021782	Dlg5	-0.63	1.10E-02	1.70E-02
6664	ENSMUSG00000042087	4933440N22Rik	-0.52	1.11E-02	1.70E-02
6665	ENSMUSG00000042010	Acacb	0.37	1.11E-02	1.70E-02
6666	ENSMUSG00000029775	Klhdc10	-0.36	1.11E-02	1.71E-02
6667	ENSMUSG00000032870	Smap2	-0.52	1.11E-02	1.71E-02
6668	ENSMUSG00000026577	Blzf1	-0.44	1.12E-02	1.72E-02
6669	ENSMUSG00000056938	Acbd4	-0.34	1.13E-02	1.73E-02
6670	ENSMUSG00000030982	9030624J02Rik	-0.37	1.14E-02	1.74E-02
6671	ENSMUSG00000040105	Plpp6	-0.35	1.14E-02	1.74E-02
6672	ENSMUSG00000001280	Sp1	-0.63	1.14E-02	1.74E-02
6673	ENSMUSG00000030602	Pak4	-0.55	1.14E-02	1.74E-02
6674	ENSMUSG00000108584	Gm45216	0.55	1.14E-02	1.75E-02
6675	ENSMUSG00000027671	Actl6a	0.37	1.14E-02	1.75E-02
6676	ENSMUSG00000025239	Limd1	-0.37	1.16E-02	1.77E-02
6677	ENSMUSG00000008226	Scrn3	0.41	1.16E-02	1.78E-02
6678	ENSMUSG00000028702	Rad54l	-0.38	1.16E-02	1.78E-02
6679	ENSMUSG00000108878	AC154141.1	0.44	1.16E-02	1.78E-02
6680	ENSMUSG00000037791	Phf12	-0.54	1.16E-02	1.78E-02
6681	ENSMUSG00000111789	AC133650.3	0.56	1.17E-02	1.79E-02
6682	ENSMUSG00000025873	Faf2	-0.37	1.17E-02	1.79E-02
6683	ENSMUSG00000014980	Tsen15	0.53	1.17E-02	1.79E-02
6684	ENSMUSG00000021113	Snapc1	-0.61	1.17E-02	1.79E-02
6685	ENSMUSG00000049323	Smcr8	-0.52	1.17E-02	1.79E-02
6686	ENSMUSG00000043421	Hilpda	-0.50	1.17E-02	1.79E-02
6687	ENSMUSG00000030019	Fbxl14	-0.44	1.17E-02	1.79E-02
6688	ENSMUSG00000108701	Gm44616	-0.59	1.18E-02	1.80E-02
6689	ENSMUSG00000048307	Ankrd46	-0.36	1.18E-02	1.80E-02
6690	ENSMUSG00000079477	Rab7	0.33	1.19E-02	1.82E-02
6691	ENSMUSG00000021023	1110008L16Rik	-0.44	1.19E-02	1.82E-02
6692	ENSMUSG00000075289	Carns1	-0.53	1.19E-02	1.82E-02
6693	ENSMUSG00000031060	Rbm10	-0.44	1.19E-02	1.82E-02
6694	ENSMUSG00000084862	Gm16278	0.43	1.19E-02	1.82E-02
6695	ENSMUSG00000012609	Ttll5	-0.54	1.19E-02	1.82E-02
6696	ENSMUSG00000030590	Fam98c	0.47	1.19E-02	1.82E-02
6697	ENSMUSG00000085572	Gm16234	0.50	1.20E-02	1.83E-02

6698	ENSMUSG00000025278	Flnb	-0.64	1.20E-02	1.83E-02
6699	ENSMUSG00000009013	Dynll1	0.42	1.20E-02	1.83E-02
6700	ENSMUSG00000056268	Dennd1b	-0.54	1.20E-02	1.83E-02
6701	ENSMUSG00000041390	Mdfic	-0.54	1.20E-02	1.84E-02
6702	ENSMUSG00000033790	Tubgcp5	-0.58	1.21E-02	1.84E-02
6703	ENSMUSG00000032478	Nme6	0.50	1.21E-02	1.85E-02
6704	ENSMUSG00000109559	Gm34280	-0.64	1.21E-02	1.85E-02
6705	ENSMUSG00000040520	Manea	-0.50	1.22E-02	1.85E-02
6706	ENSMUSG00000062761	Zfp512	-0.58	1.22E-02	1.86E-02
6707	ENSMUSG00000024974	Smc3	-0.49	1.22E-02	1.86E-02
6708	ENSMUSG00000024228	Nudt12	0.37	1.22E-02	1.86E-02
6709	ENSMUSG00000110256	Gm45412	0.51	1.23E-02	1.87E-02
6710	ENSMUSG00000027184	Caprin1	-0.36	1.23E-02	1.88E-02
6711	ENSMUSG00000024588	Fech	-0.34	1.24E-02	1.88E-02
6712	ENSMUSG00000068015	Lrch1	-0.63	1.24E-02	1.88E-02
6713	ENSMUSG00000001783	Rtcb	-0.33	1.24E-02	1.89E-02
6714	ENSMUSG00000026110	Mgat4a	-0.62	1.24E-02	1.89E-02
6715	ENSMUSG00000045252	Zfp574	0.38	1.25E-02	1.90E-02
6716	ENSMUSG00000037395	Rcor3	-0.43	1.25E-02	1.90E-02
6717	ENSMUSG00000074622	Mafb	0.47	1.25E-02	1.90E-02
6718	ENSMUSG00000031790	Mmp15	-0.59	1.25E-02	1.90E-02
6719	ENSMUSG00000022364	Tbc1d31	-0.63	1.25E-02	1.91E-02
6720	ENSMUSG00000031983	2310022B05Rik	-0.49	1.26E-02	1.91E-02
6721	ENSMUSG00000026667	Uhmk1	-0.46	1.26E-02	1.91E-02
6722	ENSMUSG00000021814	Anxa7	0.33	1.26E-02	1.91E-02
6723	ENSMUSG00000020818	Mfsd11	0.34	1.26E-02	1.91E-02
6724	ENSMUSG00000034663	Bmp2k	-0.49	1.26E-02	1.91E-02
6725	ENSMUSG00000007837	Prrg2	0.37	1.26E-02	1.92E-02
6726	ENSMUSG00000038235	F11r	0.36	1.26E-02	1.92E-02
6727	ENSMUSG00000029587	Zfp12	-0.54	1.26E-02	1.92E-02
6728	ENSMUSG00000026035	Ppil3	0.46	1.27E-02	1.92E-02
6729	ENSMUSG00000012296	Tjap1	-0.54	1.27E-02	1.92E-02
6730	ENSMUSG00000021816	Ppp3cb	0.41	1.27E-02	1.92E-02
6731	ENSMUSG00000025505	Tmem80	0.42	1.27E-02	1.93E-02
6732	ENSMUSG00000019796	Lrp11	-0.60	1.28E-02	1.94E-02
6733	ENSMUSG00000108955	Gm44775	0.43	1.28E-02	1.94E-02
6734	ENSMUSG00000027487	Cdk5rap1	0.48	1.28E-02	1.94E-02
6735	ENSMUSG00000029729	Zkscan1	-0.50	1.28E-02	1.95E-02
6736	ENSMUSG00000043183	Simc1	-0.54	1.28E-02	1.95E-02
6737	ENSMUSG00000039568	Ubald1	-0.45	1.29E-02	1.95E-02
6738	ENSMUSG00000057054	Inca1	0.42	1.29E-02	1.96E-02
6739	ENSMUSG00000036854	Hspb6	0.34	1.29E-02	1.96E-02
6740	ENSMUSG00000024766	Lipo3	0.42	1.29E-02	1.96E-02
6741	ENSMUSG00000053460	Ggcx	-0.40	1.29E-02	1.96E-02
6742	ENSMUSG00000026333	Gin1	0.54	1.29E-02	1.96E-02
6743	ENSMUSG00000031226	Pbdc1	0.55	1.30E-02	1.97E-02
6744	ENSMUSG00000037134	Prmt9	-0.42	1.30E-02	1.97E-02
6745	ENSMUSG00000021302	Ggps1	-0.41	1.31E-02	1.98E-02
6746	ENSMUSG00000028333	Anp32b	0.38	1.31E-02	1.98E-02
6747	ENSMUSG00000040936	Ulk4	-0.34	1.31E-02	1.98E-02

6748	ENSMUSG00000002129	Sf3a1	-0.49	1.31E-02	1.99E-02
6749	ENSMUSG00000032010	Usp2	0.36	1.31E-02	1.99E-02
6750	ENSMUSG00000004451	Ralb	0.37	1.31E-02	1.99E-02
6751	ENSMUSG00000061086	Myl4	-0.53	1.32E-02	2.00E-02
6752	ENSMUSG00000030275	Etnk1	-0.41	1.32E-02	2.00E-02
6753	ENSMUSG00000044456	Rin3	-0.56	1.32E-02	2.00E-02
6754	ENSMUSG00000028414	Fktn	0.45	1.32E-02	2.01E-02
6755	ENSMUSG00000005534	Insr	0.34	1.33E-02	2.01E-02
6756	ENSMUSG00000079481	Nhsl2	-0.64	1.33E-02	2.02E-02
6757	ENSMUSG00000025958	Creb1	-0.49	1.33E-02	2.02E-02
6758	ENSMUSG00000021621	Zcchc9	0.48	1.34E-02	2.02E-02
6759	ENSMUSG00000051675	Trim32	-0.48	1.34E-02	2.02E-02
6760	ENSMUSG00000007039	Ddah2	-0.58	1.34E-02	2.02E-02
6761	ENSMUSG00000047084	Ngrn	0.47	1.34E-02	2.03E-02
6762	ENSMUSG00000026436	Elk4	-0.47	1.35E-02	2.03E-02
6763	ENSMUSG00000021038	Vipas39	0.35	1.35E-02	2.04E-02
6764	ENSMUSG00000048280	Zfp738	0.61	1.35E-02	2.04E-02
6765	ENSMUSG00000028848	Gpn2	0.51	1.35E-02	2.04E-02
6766	ENSMUSG00000029823	Luc7l2	0.49	1.36E-02	2.05E-02
6767	ENSMUSG00000031489	Adrb3	-0.36	1.36E-02	2.05E-02
6768	ENSMUSG00000030589	Rasgrp4	0.50	1.36E-02	2.05E-02
6769	ENSMUSG00000007097	Atp1a2	0.35	1.36E-02	2.05E-02
6770	ENSMUSG00000042759	Apobr	0.53	1.36E-02	2.05E-02
6771	ENSMUSG00000040054	Baz2a	-0.50	1.37E-02	2.06E-02
6772	ENSMUSG00000017286	Glod4	0.33	1.37E-02	2.07E-02
6773	ENSMUSG00000030400	Ercc2	-0.55	1.37E-02	2.07E-02
6774	ENSMUSG00000091896	Ube2d2a	0.34	1.38E-02	2.08E-02
6775	ENSMUSG00000026356	Dars	0.36	1.38E-02	2.08E-02
6776	ENSMUSG00000020284	1810043G02Rik	0.35	1.38E-02	2.09E-02
6777	ENSMUSG00000048486	Fitm2	-0.36	1.38E-02	2.09E-02
6778	ENSMUSG00000018363	Smurf2	-0.51	1.38E-02	2.09E-02
6779	ENSMUSG00000027363	Usp8	0.33	1.39E-02	2.09E-02
6780	ENSMUSG00000009291	Pttg1ip	-0.33	1.39E-02	2.10E-02
6781	ENSMUSG00000047714	Ppp1r2	-0.34	1.39E-02	2.10E-02
6782	ENSMUSG00000032553	Srprb	-0.35	1.40E-02	2.11E-02
6783	ENSMUSG00000030286	Emc3	0.32	1.41E-02	2.13E-02
6784	ENSMUSG00000020283	Pex13	0.31	1.42E-02	2.13E-02
6785	ENSMUSG00000049038	Mterf2	0.48	1.42E-02	2.13E-02
6786	ENSMUSG00000029557	Mrm2	0.48	1.42E-02	2.14E-02
6787	ENSMUSG00000020777	Acox1	-0.36	1.42E-02	2.14E-02
6788	ENSMUSG00000013275	Slc41a1	-0.46	1.42E-02	2.14E-02
6789	ENSMUSG00000075054	Yae1d1	0.37	1.43E-02	2.15E-02
6790	ENSMUSG00000060904	Arl1	0.32	1.43E-02	2.15E-02
6791	ENSMUSG00000038023	Atp6v0a2	0.34	1.43E-02	2.16E-02
6792	ENSMUSG00000033352	Map2k4	-0.40	1.43E-02	2.16E-02
6793	ENSMUSG00000038451	Spsb2	-0.53	1.43E-02	2.16E-02
6794	ENSMUSG00000031887	Tradd	-0.53	1.44E-02	2.16E-02
6795	ENSMUSG00000048332	Lhfp	-0.49	1.44E-02	2.17E-02
6796	ENSMUSG00000038069	Cdkn2aip	-0.48	1.44E-02	2.17E-02
6797	ENSMUSG00000096956	Snhg18	-0.48	1.44E-02	2.17E-02

6798	ENSMUSG00000032567	Aste1	0.50	1.45E-02	2.18E-02
6799	ENSMUSG00000002731	Prkra	0.46	1.46E-02	2.19E-02
6800	ENSMUSG00000019088	Dnase1l1	-0.50	1.46E-02	2.19E-02
6801	ENSMUSG00000039990	Edrf1	-0.54	1.46E-02	2.19E-02
6802	ENSMUSG00000024830	Rps6kb2	-0.42	1.46E-02	2.19E-02
6803	ENSMUSG00000101089	2610016A17Rik	-0.49	1.46E-02	2.20E-02
6804	ENSMUSG00000003131	Pafah1b2	-0.35	1.47E-02	2.21E-02
6805	ENSMUSG00000023092	Fhl1	0.33	1.48E-02	2.22E-02
6806	ENSMUSG00000029432	Gbas	0.32	1.48E-02	2.22E-02
6807	ENSMUSG00000039908	Slc26a11	-0.58	1.48E-02	2.22E-02
6808	ENSMUSG00000100039	Gm28959	-0.41	1.48E-02	2.23E-02
6809	ENSMUSG00000040139	9430038I01Rik	0.48	1.48E-02	2.23E-02
6810	ENSMUSG00000048234	Rnf149	-0.35	1.49E-02	2.23E-02
6811	ENSMUSG00000032018	Sc5d	-0.40	1.49E-02	2.23E-02
6812	ENSMUSG00000029730	Mcm7	-0.52	1.49E-02	2.23E-02
6813	ENSMUSG00000066643	Wdr35	-0.53	1.49E-02	2.24E-02
6814	ENSMUSG00000036093	Arl5a	-0.41	1.49E-02	2.24E-02
6815	ENSMUSG00000019988	Nedd1	-0.49	1.50E-02	2.25E-02
6816	ENSMUSG00000058881	Zfp516	-0.60	1.50E-02	2.25E-02
6817	ENSMUSG00000046798	Cldn12	-0.37	1.50E-02	2.26E-02
6818	ENSMUSG00000023072	Cep89	-0.56	1.51E-02	2.26E-02
6819	ENSMUSG00000038252	Ncapd2	-0.54	1.51E-02	2.26E-02
6820	ENSMUSG00000023467	Tulp2	0.42	1.51E-02	2.26E-02
6821	ENSMUSG00000062931	Zfp938	0.50	1.51E-02	2.27E-02
6822	ENSMUSG00000025408	Ddit3	0.45	1.52E-02	2.28E-02
6823	ENSMUSG00000057156	Homez	-0.56	1.52E-02	2.28E-02
6824	ENSMUSG00000028906	Epb41	-0.36	1.53E-02	2.29E-02
6825	ENSMUSG00000042677	Zc3h12a	-0.57	1.53E-02	2.29E-02
6826	ENSMUSG00000025270	Alas2	-0.54	1.53E-02	2.29E-02
6827	ENSMUSG00000041153	Osgin2	-0.43	1.55E-02	2.32E-02
6828	ENSMUSG00000047187	Rab2a	0.31	1.55E-02	2.32E-02
6829	ENSMUSG00000002108	Nr1h3	0.31	1.56E-02	2.33E-02
6830	ENSMUSG00000035268	Pkig	0.43	1.56E-02	2.34E-02
6831	ENSMUSG00000037553	Zdhhc18	-0.38	1.56E-02	2.34E-02
6832	ENSMUSG00000014837	4931428F04Rik	0.54	1.57E-02	2.35E-02
6833	ENSMUSG00000042275	Pelo	0.55	1.57E-02	2.35E-02
6834	ENSMUSG00000109881	Gm45507	0.34	1.58E-02	2.36E-02
6835	ENSMUSG00000001065	Zfp276	-0.51	1.58E-02	2.37E-02
6836	ENSMUSG00000004891	Nes	-0.56	1.59E-02	2.38E-02
6837	ENSMUSG00000036894	Rap2b	-0.50	1.59E-02	2.38E-02
6838	ENSMUSG00000073982	Rhog	-0.40	1.59E-02	2.38E-02
6839	ENSMUSG00000000056	Narf	0.41	1.60E-02	2.39E-02
6840	ENSMUSG00000026289	Atg16l1	-0.37	1.60E-02	2.39E-02
6841	ENSMUSG00000067148	Polr1c	0.41	1.60E-02	2.39E-02
6842	ENSMUSG00000002109	Ddb2	-0.48	1.61E-02	2.40E-02
6843	ENSMUSG00000038855	Itpkb	0.53	1.61E-02	2.40E-02
6844	ENSMUSG00000047284	Neurl4	0.53	1.61E-02	2.41E-02
6845	ENSMUSG00000021591	Glrx	0.63	1.62E-02	2.42E-02
6846	ENSMUSG00000038299	Wdr36	-0.37	1.62E-02	2.43E-02
6847	ENSMUSG00000042712	Tceal9	0.58	1.62E-02	2.43E-02

6848	ENSMUSG00000046916	Myct1	-0.62	1.64E-02	2.45E-02
6849	ENSMUSG00000020075	Ddx21	-0.46	1.64E-02	2.45E-02
6850	ENSMUSG00000038187	Btbd10	-0.43	1.64E-02	2.46E-02
6851	ENSMUSG00000078735	Il11ra2	-0.36	1.65E-02	2.46E-02
6852	ENSMUSG00000052533	Nup188	-0.42	1.65E-02	2.46E-02
6853	ENSMUSG00000061306	Slc38a10	-0.36	1.65E-02	2.46E-02
6854	ENSMUSG00000039745	Htatip2	0.39	1.65E-02	2.47E-02
6855	ENSMUSG00000033386	Frrs1	0.43	1.66E-02	2.47E-02
6856	ENSMUSG00000036282	Naa30	0.38	1.66E-02	2.47E-02
6857	ENSMUSG00000058655	Eif4b	-0.33	1.66E-02	2.47E-02
6858	ENSMUSG00000104559	Gm43118	-0.39	1.66E-02	2.48E-02
6859	ENSMUSG00000022228	Zscan26	-0.54	1.67E-02	2.49E-02
6860	ENSMUSG00000020238	Ncln	-0.34	1.67E-02	2.49E-02
6861	ENSMUSG00000039428	Tmem135	0.35	1.67E-02	2.49E-02
6862	ENSMUSG00000031622	Sin3b	0.32	1.67E-02	2.49E-02
6863	ENSMUSG00000034269	Setd5	-0.44	1.68E-02	2.50E-02
6864	ENSMUSG00000019923	Zwint	0.32	1.68E-02	2.50E-02
6865	ENSMUSG00000063904	Dpp3	-0.36	1.69E-02	2.51E-02
6866	ENSMUSG00000022016	Akap11	-0.51	1.69E-02	2.51E-02
6867	ENSMUSG00000036707	Cab39	0.34	1.69E-02	2.52E-02
6868	ENSMUSG00000032492	Pth1r	-0.43	1.69E-02	2.52E-02
6869	ENSMUSG00000025586	Cpeb1	-0.48	1.69E-02	2.52E-02
6870	ENSMUSG00000010110	Stx5a	-0.37	1.70E-02	2.54E-02
6871	ENSMUSG00000033960	9430020K01Rik	-0.49	1.71E-02	2.54E-02
6872	ENSMUSG00000029167	Ppargc1a	0.47	1.71E-02	2.54E-02
6873	ENSMUSG00000001383	Zmat2	0.40	1.71E-02	2.54E-02
6874	ENSMUSG00000037111	Setd7	-0.50	1.71E-02	2.54E-02
6875	ENSMUSG00000058454	Dhcr7	-0.55	1.71E-02	2.55E-02
6876	ENSMUSG00000042595	Fam199x	-0.51	1.71E-02	2.55E-02
6877	ENSMUSG00000071533	Pcnp	0.35	1.71E-02	2.55E-02
6878	ENSMUSG00000055188	Rbm3os	-0.52	1.71E-02	2.55E-02
6879	ENSMUSG00000020265	Sumo3	-0.34	1.72E-02	2.55E-02
6880	ENSMUSG00000026812	Tsc1	-0.45	1.72E-02	2.56E-02
6881	ENSMUSG00000057113	Npm1	0.35	1.72E-02	2.56E-02
6882	ENSMUSG00000020152	Actr2	-0.37	1.72E-02	2.56E-02
6883	ENSMUSG00000019984	Med23	-0.51	1.73E-02	2.57E-02
6884	ENSMUSG00000062825	Actg1	-0.39	1.73E-02	2.57E-02
6885	ENSMUSG00000038014	Fam120a	-0.35	1.74E-02	2.58E-02
6886	ENSMUSG00000022048	Dpysl2	-0.51	1.74E-02	2.59E-02
6887	ENSMUSG00000090394	4930523C07Rik	-0.40	1.74E-02	2.59E-02
6888	ENSMUSG00000024269	Tpgs2	0.39	1.75E-02	2.60E-02
6889	ENSMUSG00000001482	Def8	-0.36	1.75E-02	2.60E-02
6890	ENSMUSG00000048833	Slc39a9	-0.46	1.75E-02	2.60E-02
6891	ENSMUSG00000028868	Wasf2	-0.36	1.75E-02	2.60E-02
6892	ENSMUSG00000028086	Fbxw7	-0.56	1.76E-02	2.60E-02
6893	ENSMUSG00000044636	Csrnp2	-0.56	1.76E-02	2.61E-02
6894	ENSMUSG00000003437	Paf1	-0.38	1.76E-02	2.61E-02
6895	ENSMUSG00000016940	Kctd2	0.33	1.76E-02	2.61E-02
6896	ENSMUSG00000022475	Hdac7	-0.54	1.77E-02	2.62E-02
6897	ENSMUSG00000020925	Ccdc43	0.45	1.77E-02	2.62E-02

6898	ENSMUSG00000097290	1300002E11Rik	0.39	1.77E-02	2.62E-02
6899	ENSMUSG00000090112	Shprh	-0.48	1.77E-02	2.62E-02
6900	ENSMUSG00000048271	Rbm33	-0.47	1.77E-02	2.63E-02
6901	ENSMUSG00000027763	Mbnl1	-0.31	1.77E-02	2.63E-02
6902	ENSMUSG00000100594	2810414N06Rik	0.42	1.77E-02	2.63E-02
6903	ENSMUSG00000047213	Ythdf3	-0.35	1.78E-02	2.63E-02
6904	ENSMUSG00000097518	Gm26694	-0.51	1.78E-02	2.63E-02
6905	ENSMUSG00000031298	Adgrg2	0.45	1.78E-02	2.64E-02
6906	ENSMUSG00000025532	Crcp	0.38	1.78E-02	2.64E-02
6907	ENSMUSG00000021774	Ube2e1	0.34	1.78E-02	2.64E-02
6908	ENSMUSG00000025439	Clns1a	0.33	1.79E-02	2.65E-02
6909	ENSMUSG00000068966	Zbtb34	-0.59	1.79E-02	2.65E-02
6910	ENSMUSG00000024780	Cdc37l1	0.40	1.79E-02	2.66E-02
6911	ENSMUSG00000022855	Senp2	0.38	1.79E-02	2.66E-02
6912	ENSMUSG00000037761	Actr5	-0.56	1.80E-02	2.66E-02
6913	ENSMUSG00000002413	Braf	-0.45	1.80E-02	2.66E-02
6914	ENSMUSG00000066232	Ipo7	0.38	1.81E-02	2.67E-02
6915	ENSMUSG00000092356	Gm20532	-0.37	1.81E-02	2.67E-02
6916	ENSMUSG00000025758	Plk4	-0.60	1.81E-02	2.67E-02
6917	ENSMUSG00000079478	Sssca1	0.32	1.82E-02	2.70E-02
6918	ENSMUSG00000018765	Fxr2	-0.37	1.83E-02	2.70E-02
6919	ENSMUSG00000030643	Rab30	-0.59	1.83E-02	2.71E-02
6920	ENSMUSG00000061273	Mmgt1	0.36	1.84E-02	2.71E-02
6921	ENSMUSG00000078566	Bnip3	0.34	1.84E-02	2.72E-02
6922	ENSMUSG00000020253	Ppm1m	-0.33	1.84E-02	2.72E-02
6923	ENSMUSG00000041143	Tmco4	0.39	1.84E-02	2.72E-02
6924	ENSMUSG00000028902	Sf3a3	-0.38	1.85E-02	2.72E-02
6925	ENSMUSG00000061118	Dnajc30	0.34	1.85E-02	2.73E-02
6926	ENSMUSG00000044167	Foxo1	-0.39	1.85E-02	2.73E-02
6927	ENSMUSG00000039828	Wdr70	0.44	1.85E-02	2.73E-02
6928	ENSMUSG00000037815	Ctnna1	0.34	1.86E-02	2.75E-02
6929	ENSMUSG00000059277	R74862	-0.34	1.86E-02	2.75E-02
6930	ENSMUSG00000044030	Irf2bp1	-0.39	1.86E-02	2.75E-02
6931	ENSMUSG00000026019	Wdr12	0.37	1.87E-02	2.76E-02
6932	ENSMUSG00000039715	Wdr34	0.40	1.87E-02	2.76E-02
6933	ENSMUSG00000029110	Rnf4	-0.34	1.87E-02	2.76E-02
6934	ENSMUSG00000021770	Samd8	-0.43	1.87E-02	2.76E-02
6935	ENSMUSG00000032119	Hinfp	-0.51	1.88E-02	2.77E-02
6936	ENSMUSG00000033628	Pik3c3	-0.44	1.88E-02	2.77E-02
6937	ENSMUSG00000027998	Plrg1	-0.36	1.88E-02	2.77E-02
6938	ENSMUSG00000031617	Tmem184c	-0.34	1.89E-02	2.79E-02
6939	ENSMUSG00000054499	Dedd2	0.40	1.90E-02	2.80E-02
6940	ENSMUSG00000059273	Zc3h4	-0.49	1.90E-02	2.80E-02
6941	ENSMUSG00000026174	Cnot9	-0.42	1.90E-02	2.80E-02
6942	ENSMUSG00000028284	Map3k7	-0.33	1.90E-02	2.80E-02
6943	ENSMUSG00000057554	Lgals8	-0.36	1.91E-02	2.81E-02
6944	ENSMUSG00000026696	Vamp4	0.48	1.92E-02	2.82E-02
6945	ENSMUSG00000026455	Klhl12	-0.38	1.92E-02	2.83E-02
6946	ENSMUSG00000004393	Ddx56	-0.33	1.93E-02	2.84E-02
6947	ENSMUSG00000028745	Capzb	0.30	1.94E-02	2.85E-02

6948	ENSMUSG00000017764	Zswim1	-0.43	1.94E-02	2.85E-02
6949	ENSMUSG00000035007	Rundc1	-0.40	1.94E-02	2.85E-02
6950	ENSMUSG00000093575	Gm20695	-0.38	1.94E-02	2.85E-02
6951	ENSMUSG00000092176	Gm20460	0.37	1.94E-02	2.85E-02
6952	ENSMUSG00000022095	Fam160b2	0.37	1.94E-02	2.85E-02
6953	ENSMUSG00000038615	Nfe2l1	-0.31	1.94E-02	2.86E-02
6954	ENSMUSG00000020873	Slc35b1	0.33	1.95E-02	2.86E-02
6955	ENSMUSG00000033228	Scaf11	-0.36	1.95E-02	2.86E-02
6956	ENSMUSG00000042208	0610010F05Rik	-0.37	1.95E-02	2.87E-02
6957	ENSMUSG00000031889	D230025D16Rik	-0.37	1.96E-02	2.89E-02
6958	ENSMUSG00000049686	Orai1	0.38	1.97E-02	2.89E-02
6959	ENSMUSG00000040021	Lats1	-0.40	1.97E-02	2.90E-02
6960	ENSMUSG00000045691	Thtpa	-0.37	1.97E-02	2.90E-02
6961	ENSMUSG00000037736	Limch1	-0.55	1.98E-02	2.90E-02
6962	ENSMUSG00000069633	Pex11g	0.36	1.98E-02	2.91E-02
6963	ENSMUSG00000036966	Spryd3	-0.43	1.98E-02	2.91E-02
6964	ENSMUSG00000040818	Dennd6a	0.37	1.99E-02	2.92E-02
6965	ENSMUSG00000033781	Asb13	-0.34	1.99E-02	2.92E-02
6966	ENSMUSG00000079737	3110001I22Rik	0.43	1.99E-02	2.92E-02
6967	ENSMUSG00000028729	Ebna1bp2	0.41	1.99E-02	2.92E-02
6968	ENSMUSG00000110615	Gm45890	-0.47	1.99E-02	2.92E-02
6969	ENSMUSG00000030272	Camk1	-0.33	2.00E-02	2.93E-02
6970	ENSMUSG00000036992	Nxt1	0.48	2.01E-02	2.94E-02
6971	ENSMUSG00000023104	Rfc2	0.39	2.01E-02	2.94E-02
6972	ENSMUSG00000006276	Eps15l1	-0.42	2.02E-02	2.96E-02
6973	ENSMUSG00000109998	Gm45437	-0.55	2.02E-02	2.96E-02
6974	ENSMUSG00000037536	Fbxo34	-0.43	2.02E-02	2.96E-02
6975	ENSMUSG00000066979	Bub3	-0.32	2.02E-02	2.96E-02
6976	ENSMUSG00000040945	Rcc2	-0.37	2.02E-02	2.97E-02
6977	ENSMUSG00000035762	Tmem161b	0.44	2.03E-02	2.97E-02
6978	ENSMUSG00000021477	Ctsl	-0.30	2.03E-02	2.98E-02
6979	ENSMUSG00000012117	Dhdds	-0.36	2.03E-02	2.98E-02
6980	ENSMUSG00000011960	Ccnt1	0.39	2.04E-02	2.99E-02
6981	ENSMUSG00000078485	Plekhn1	-0.38	2.05E-02	3.00E-02
6982	ENSMUSG00000005069	Pex5	-0.31	2.05E-02	3.01E-02
6983	ENSMUSG00000034820	Cpsf7	-0.46	2.05E-02	3.01E-02
6984	ENSMUSG00000046020	Pofut1	-0.36	2.05E-02	3.01E-02
6985	ENSMUSG00000030339	Ltbr	-0.31	2.06E-02	3.01E-02
6986	ENSMUSG00000045237	1110012L19Rik	0.44	2.07E-02	3.03E-02
6987	ENSMUSG00000028393	Alad	0.30	2.07E-02	3.03E-02
6988	ENSMUSG00000107846	Gm43963	-0.46	2.08E-02	3.04E-02
6989	ENSMUSG00000063450	Syne2	-0.44	2.09E-02	3.05E-02
6990	ENSMUSG00000005949	Ctns	-0.44	2.09E-02	3.06E-02
6991	ENSMUSG00000019866	Aim1	-0.55	2.09E-02	3.06E-02
6992	ENSMUSG00000014498	Ankrd52	0.36	2.09E-02	3.06E-02
6993	ENSMUSG00000025995	Wdr75	-0.48	2.10E-02	3.07E-02
6994	ENSMUSG00000042745	Id1	0.47	2.11E-02	3.08E-02
6995	ENSMUSG00000025878	Uimc1	-0.47	2.11E-02	3.08E-02
6996	ENSMUSG00000058835	Abi1	-0.41	2.11E-02	3.08E-02
6997	ENSMUSG00000045776	Lrtm1	-0.50	2.12E-02	3.10E-02

6998	ENSMUSG00000018415	Gid4	0.31	2.12E-02	3.10E-02
6999	ENSMUSG00000034522	Zfp395	-0.48	2.12E-02	3.10E-02
7000	ENSMUSG00000020258	Glyctk	-0.52	2.13E-02	3.11E-02
7001	ENSMUSG00000027952	Pmvk	0.42	2.13E-02	3.11E-02
7002	ENSMUSG00000056131	Pgm3	-0.50	2.13E-02	3.12E-02
7003	ENSMUSG00000073876	Gm13305	-0.34	2.14E-02	3.12E-02
7004	ENSMUSG00000052565	Hist1h1d	0.50	2.14E-02	3.12E-02
7005	ENSMUSG00000031145	Prickle3	-0.39	2.14E-02	3.12E-02
7006	ENSMUSG00000038324	Trpc4ap	-0.30	2.14E-02	3.13E-02
7007	ENSMUSG00000022742	Cpox	-0.46	2.14E-02	3.13E-02
7008	ENSMUSG00000027865	Gdap2	-0.38	2.15E-02	3.14E-02
7009	ENSMUSG00000003992	Ssbp2	-0.38	2.17E-02	3.17E-02
7010	ENSMUSG00000040213	Kyat3	0.32	2.17E-02	3.17E-02
7011	ENSMUSG00000038025	Phf2	-0.48	2.17E-02	3.17E-02
7012	ENSMUSG00000024164	C3	-0.37	2.18E-02	3.17E-02
7013	ENSMUSG00000100629	Gm28192	-0.46	2.18E-02	3.17E-02
7014	ENSMUSG00000097121	D130020L05Rik	-0.45	2.18E-02	3.18E-02
7015	ENSMUSG00000015377	Dennd6b	0.54	2.18E-02	3.18E-02
7016	ENSMUSG00000017288	Vps53	-0.37	2.19E-02	3.19E-02
7017	ENSMUSG00000037514	Pank2	-0.33	2.19E-02	3.19E-02
7018	ENSMUSG00000041429	Nthl1	-0.52	2.19E-02	3.20E-02
7019	ENSMUSG00000047879	Usp14	0.31	2.20E-02	3.20E-02
7020	ENSMUSG00000037613	Tnfrsf23	0.48	2.20E-02	3.21E-02
7021	ENSMUSG00000074649	BC029722	0.33	2.21E-02	3.22E-02
7022	ENSMUSG00000089917	Uckl1	0.32	2.22E-02	3.24E-02
7023	ENSMUSG00000027706	Sec62	0.32	2.23E-02	3.24E-02
7024	ENSMUSG00000032295	Man2c1	-0.32	2.23E-02	3.25E-02
7025	ENSMUSG00000104822	Gm42967	-0.52	2.25E-02	3.28E-02
7026	ENSMUSG00000039480	Nt5dc1	0.38	2.26E-02	3.29E-02
7027	ENSMUSG00000032498	Mlh1	-0.50	2.26E-02	3.29E-02
7028	ENSMUSG00000001123	Lgals9	0.33	2.27E-02	3.30E-02
7029	ENSMUSG00000035992	Fnip1	0.46	2.27E-02	3.30E-02
7030	ENSMUSG00000026307	Scly	-0.46	2.27E-02	3.30E-02
7031	ENSMUSG00000097537	2610020C07Rik	-0.41	2.27E-02	3.31E-02
7032	ENSMUSG00000036188	Ankmy2	-0.36	2.28E-02	3.31E-02
7033	ENSMUSG00000029191	Rfc1	-0.39	2.28E-02	3.31E-02
7034	ENSMUSG00000046034	Otulin	0.34	2.28E-02	3.31E-02
7035	ENSMUSG00000020134	Peli1	-0.46	2.28E-02	3.32E-02
7036	ENSMUSG00000020883	Fbxl20	-0.42	2.28E-02	3.32E-02
7037	ENSMUSG00000020585	Laptm4a	0.30	2.28E-02	3.32E-02
7038	ENSMUSG00000073147	5031425E22Rik	0.49	2.29E-02	3.32E-02
7039	ENSMUSG00000034321	Exosc1	0.45	2.30E-02	3.34E-02
7040	ENSMUSG00000029864	Gstk1	0.34	2.33E-02	3.38E-02
7041	ENSMUSG00000083282	Ctsf	0.31	2.33E-02	3.38E-02
7042	ENSMUSG00000009630	Ppp2cb	0.32	2.33E-02	3.38E-02
7043	ENSMUSG00000034212	Ankmy1	-0.51	2.33E-02	3.39E-02
7044	ENSMUSG00000026074	Map4k4	0.35	2.34E-02	3.40E-02
7045	ENSMUSG00000078931	Pdf	0.31	2.34E-02	3.40E-02
7046	ENSMUSG00000033022	Cdo1	0.33	2.35E-02	3.41E-02
7047	ENSMUSG00000030609	Aen	-0.38	2.35E-02	3.41E-02

7048	ENSMUSG00000033885	Pxk	-0.34	2.37E-02	3.44E-02
7049	ENSMUSG00000027782	Kpna4	-0.34	2.38E-02	3.45E-02
7050	ENSMUSG00000097025	Gm26558	-0.48	2.38E-02	3.45E-02
7051	ENSMUSG00000051412	Vamp7	0.47	2.38E-02	3.45E-02
7052	ENSMUSG00000032401	Lctl	-0.43	2.38E-02	3.46E-02
7053	ENSMUSG00000039942	Ptger4	-0.48	2.39E-02	3.46E-02
7054	ENSMUSG00000062901	Klhl24	-0.42	2.39E-02	3.46E-02
7055	ENSMUSG00000021213	Akr1c13	0.44	2.40E-02	3.48E-02
7056	ENSMUSG00000031029	Eif3f	0.30	2.40E-02	3.48E-02
7057	ENSMUSG00000016409	Nkap	-0.50	2.41E-02	3.49E-02
7058	ENSMUSG00000045094	Arhgef37	-0.52	2.41E-02	3.49E-02
7059	ENSMUSG00000038374	Rbm8a	0.34	2.42E-02	3.50E-02
7060	ENSMUSG00000036985	Zdhhc9	-0.42	2.43E-02	3.52E-02
7061	ENSMUSG00000041915	Ammecr1l	-0.33	2.44E-02	3.54E-02
7062	ENSMUSG00000002845	Tmem39a	-0.45	2.45E-02	3.54E-02
7063	ENSMUSG00000029022	Miip	0.50	2.45E-02	3.54E-02
7064	ENSMUSG00000031353	Rbbp7	-0.31	2.46E-02	3.56E-02
7065	ENSMUSG00000033713	Foxn3	0.32	2.47E-02	3.57E-02
7066	ENSMUSG00000038119	Cdon	-0.55	2.47E-02	3.57E-02
7067	ENSMUSG00000022774	Ncbp2	0.36	2.48E-02	3.59E-02
7068	ENSMUSG00000024644	Cndp2	-0.31	2.48E-02	3.59E-02
7069	ENSMUSG00000056487	Mettl7a2	0.32	2.48E-02	3.59E-02
7070	ENSMUSG00000037887	Dusp8	-0.60	2.50E-02	3.62E-02
7071	ENSMUSG00000032456	Nmnat3	0.37	2.50E-02	3.62E-02
7072	ENSMUSG00000035226	Rims4	-0.47	2.50E-02	3.62E-02
7073	ENSMUSG00000029648	Flt1	-0.41	2.51E-02	3.62E-02
7074	ENSMUSG00000070476	Fam217b	0.50	2.51E-02	3.63E-02
7075	ENSMUSG00000107928	Gm45140	-0.39	2.52E-02	3.64E-02
7076	ENSMUSG00000029209	Gnpda2	-0.36	2.52E-02	3.65E-02
7077	ENSMUSG00000012076	Brms1l	-0.41	2.52E-02	3.65E-02
7078	ENSMUSG00000042203	Tbc1d22b	-0.43	2.53E-02	3.66E-02
7079	ENSMUSG00000028634	Hivep3	-0.47	2.54E-02	3.66E-02
7080	ENSMUSG00000032422	Snx14	0.37	2.54E-02	3.67E-02
7081	ENSMUSG00000044408	Sptssa	0.31	2.54E-02	3.67E-02
7082	ENSMUSG00000001127	Araf	-0.29	2.55E-02	3.68E-02
7083	ENSMUSG00000005907	Pex1	-0.42	2.55E-02	3.68E-02
7084	ENSMUSG00000047822	Angptl8	0.53	2.55E-02	3.68E-02
7085	ENSMUSG00000026709	Dars2	-0.42	2.55E-02	3.69E-02
7086	ENSMUSG00000034432	Cops8	0.29	2.55E-02	3.69E-02
7087	ENSMUSG00000020063	Sirt1	-0.47	2.56E-02	3.70E-02
7088	ENSMUSG00000024754	Tmem2	-0.53	2.56E-02	3.70E-02
7089	ENSMUSG00000005034	Prkacb	-0.33	2.56E-02	3.70E-02
7090	ENSMUSG00000036241	Ube2r2	-0.29	2.57E-02	3.71E-02
7091	ENSMUSG00000041696	Rasl12	-0.33	2.57E-02	3.71E-02
7092	ENSMUSG00000053040	Aph1c	-0.51	2.57E-02	3.71E-02
7093	ENSMUSG00000075376	Rc3h2	-0.40	2.58E-02	3.72E-02
7094	ENSMUSG00000002274	Metrn	-0.48	2.59E-02	3.74E-02
7095	ENSMUSG00000045962	Wnk1	0.39	2.59E-02	3.74E-02
7096	ENSMUSG00000033111	3830406C13Rik	0.34	2.61E-02	3.76E-02
7097	ENSMUSG00000030530	Furin	0.38	2.62E-02	3.77E-02

7098	ENSMUSG00000053706	B430305J03Rik	-0.47	2.63E-02	3.79E-02
7099	ENSMUSG00000022000	Zc3h13	-0.43	2.64E-02	3.81E-02
7100	ENSMUSG00000041037	Irgq	-0.32	2.65E-02	3.82E-02
7101	ENSMUSG00000059495	Arhgef12	0.32	2.66E-02	3.82E-02
7102	ENSMUSG00000059436	Max	0.48	2.66E-02	3.83E-02
7103	ENSMUSG00000047003	Zfp41	-0.49	2.66E-02	3.83E-02
7104	ENSMUSG00000057522	Spop	0.29	2.67E-02	3.85E-02
7105	ENSMUSG00000078923	Ube2v1	0.31	2.69E-02	3.87E-02
7106	ENSMUSG00000086826	Gm11739	-0.52	2.69E-02	3.87E-02
7107	ENSMUSG00000031349	Nsdhl	0.36	2.69E-02	3.87E-02
7108	ENSMUSG00000038866	Zcchc2	-0.40	2.70E-02	3.88E-02
7109	ENSMUSG00000021610	Clptm1l	-0.30	2.70E-02	3.88E-02
7110	ENSMUSG00000039220	Ppp1r10	-0.43	2.71E-02	3.89E-02
7111	ENSMUSG00000032435	Dync1li1	-0.33	2.71E-02	3.90E-02
7112	ENSMUSG00000009741	Ubp1	0.34	2.72E-02	3.90E-02
7113	ENSMUSG00000029004	Kmt2e	-0.33	2.72E-02	3.91E-02
7114	ENSMUSG00000078789	Dph1	0.32	2.72E-02	3.91E-02
7115	ENSMUSG00000092190	Gm20470	0.42	2.73E-02	3.92E-02
7116	ENSMUSG00000024258	Polr2d	0.41	2.73E-02	3.93E-02
7117	ENSMUSG00000055675	Kbtbd11	-0.38	2.73E-02	3.93E-02
7118	ENSMUSG00000034453	Polr3b	-0.44	2.74E-02	3.93E-02
7119	ENSMUSG00000032300	1700017B05Rik	0.34	2.74E-02	3.93E-02
7120	ENSMUSG00000018583	G3bp1	-0.29	2.74E-02	3.94E-02
7121	ENSMUSG00000030509	Asb7	-0.49	2.75E-02	3.95E-02
7122	ENSMUSG00000041890	Git2	-0.40	2.75E-02	3.95E-02
7123	ENSMUSG00000028792	Ak2	0.30	2.76E-02	3.96E-02
7124	ENSMUSG00000047248	C2cd3	-0.55	2.76E-02	3.96E-02
7125	ENSMUSG00000044952	Kctd21	-0.45	2.77E-02	3.97E-02
7126	ENSMUSG00000020677	Ddx52	-0.41	2.77E-02	3.97E-02
7127	ENSMUSG00000021180	Rps6ka5	-0.54	2.77E-02	3.98E-02
7128	ENSMUSG00000042524	Sun2	0.40	2.78E-02	3.98E-02
7129	ENSMUSG00000022557	Bop1	-0.31	2.78E-02	3.99E-02
7130	ENSMUSG00000008958	Vps72	0.36	2.79E-02	4.01E-02
7131	ENSMUSG00000034120	Srsf2	-0.31	2.80E-02	4.01E-02
7132	ENSMUSG00000027667	Zfp639	0.36	2.80E-02	4.02E-02
7133	ENSMUSG00000006456	Rbm14	-0.34	2.81E-02	4.02E-02
7134	ENSMUSG00000027236	Eif3j1	0.33	2.81E-02	4.02E-02
7135	ENSMUSG00000044477	Zfand3	0.36	2.81E-02	4.03E-02
7136	ENSMUSG00000041837	Pdcd7	-0.44	2.82E-02	4.04E-02
7137	ENSMUSG00000005102	Eif2ak4	-0.52	2.82E-02	4.04E-02
7138	ENSMUSG00000059669	Taf1b	-0.43	2.82E-02	4.04E-02
7139	ENSMUSG00000029647	Pan3	-0.49	2.83E-02	4.05E-02
7140	ENSMUSG00000052446	Zfp961	0.45	2.83E-02	4.06E-02
7141	ENSMUSG00000019883	Echdc1	0.37	2.84E-02	4.07E-02
7142	ENSMUSG00000079509	Zfx	-0.42	2.84E-02	4.07E-02
7143	ENSMUSG00000055024	Ep300	-0.46	2.85E-02	4.07E-02
7144	ENSMUSG00000031755	Bbs2	-0.49	2.85E-02	4.08E-02
7145	ENSMUSG00000029229	Chic2	0.37	2.85E-02	4.09E-02
7146	ENSMUSG00000038888	Ctu1	-0.51	2.86E-02	4.09E-02
7147	ENSMUSG00000040455	Usp45	-0.45	2.86E-02	4.09E-02

7148	ENSMUSG00000018362	Kpna2	-0.36	2.87E-02	4.10E-02
7149	ENSMUSG00000022529	Zfp263	-0.46	2.87E-02	4.10E-02
7150	ENSMUSG00000025371	Chmp6	0.32	2.87E-02	4.10E-02
7151	ENSMUSG00000010755	Cars	-0.33	2.87E-02	4.11E-02
7152	ENSMUSG00000019826	Zbtb24	-0.46	2.87E-02	4.11E-02
7153	ENSMUSG00000026854	Usp20	-0.39	2.87E-02	4.11E-02
7154	ENSMUSG00000043831	Lysmd4	-0.42	2.88E-02	4.11E-02
7155	ENSMUSG00000040795	Iqcc	0.44	2.88E-02	4.12E-02
7156	ENSMUSG00000069919	Hba-a1	0.35	2.89E-02	4.13E-02
7157	ENSMUSG00000038545	Cul7	-0.42	2.90E-02	4.15E-02
7158	ENSMUSG00000021972	Hmbox1	0.45	2.91E-02	4.15E-02
7159	ENSMUSG00000002524	Puf60	0.29	2.91E-02	4.16E-02
7160	ENSMUSG00000028249	Sdcbp	0.32	2.91E-02	4.16E-02
7161	ENSMUSG00000021785	Ngly1	0.29	2.91E-02	4.16E-02
7162	ENSMUSG00000038876	Rnf146	0.36	2.92E-02	4.18E-02
7163	ENSMUSG00000063015	Ccni	0.29	2.94E-02	4.19E-02
7164	ENSMUSG00000041360	Pum3	-0.38	2.94E-02	4.20E-02
7165	ENSMUSG00000040359	Ufl1	-0.40	2.94E-02	4.20E-02
7166	ENSMUSG00000057421	Las1l	-0.32	2.95E-02	4.21E-02
7167	ENSMUSG00000015095	Fbxw5	0.29	2.95E-02	4.21E-02
7168	ENSMUSG00000028478	Clta	0.29	2.96E-02	4.22E-02
7169	ENSMUSG00000000600	Krit1	-0.42	2.96E-02	4.22E-02
7170	ENSMUSG00000021549	Rasa1	-0.36	2.97E-02	4.23E-02
7171	ENSMUSG00000009566	Fpgs	-0.46	2.97E-02	4.23E-02
7172	ENSMUSG00000035151	Elmod2	-0.36	2.97E-02	4.23E-02
7173	ENSMUSG00000070427	Il18bp	-0.48	2.98E-02	4.24E-02
7174	ENSMUSG00000020946	Gosr2	-0.31	2.98E-02	4.25E-02
7175	ENSMUSG00000059995	Atxn7l3	-0.51	2.99E-02	4.26E-02
7176	ENSMUSG00000086914	Gm16124	0.45	3.00E-02	4.27E-02
7177	ENSMUSG00000025578	Cbx8	0.45	3.00E-02	4.28E-02
7178	ENSMUSG00000033105	Lss	-0.38	3.00E-02	4.28E-02
7179	ENSMUSG00000049411	Tmem241	0.35	3.01E-02	4.29E-02
7180	ENSMUSG00000029475	Kdm2b	-0.48	3.02E-02	4.31E-02
7181	ENSMUSG00000021900	Btd	-0.30	3.03E-02	4.32E-02
7182	ENSMUSG00000024742	Fen1	-0.48	3.04E-02	4.33E-02
7183	ENSMUSG00000030533	Unc45a	-0.34	3.05E-02	4.34E-02
7184	ENSMUSG00000003345	Csnk1g2	-0.29	3.05E-02	4.35E-02
7185	ENSMUSG00000004980	Hnrnpa2b1	-0.28	3.06E-02	4.35E-02
7186	ENSMUSG00000036819	Jmjd4	-0.47	3.06E-02	4.35E-02
7187	ENSMUSG00000020741	Cluh	-0.29	3.08E-02	4.38E-02
7188	ENSMUSG00000058594	Fbxo18	-0.31	3.08E-02	4.38E-02
7189	ENSMUSG00000039616	Mocos	-0.45	3.09E-02	4.39E-02
7190	ENSMUSG00000034412	Tbc1d10a	-0.45	3.09E-02	4.40E-02
7191	ENSMUSG00000040167	Ikzf5	-0.50	3.10E-02	4.41E-02
7192	ENSMUSG00000047528	Als2cr12	0.48	3.11E-02	4.42E-02
7193	ENSMUSG00000043059	Zfp513	-0.48	3.12E-02	4.43E-02
7194	ENSMUSG00000035637	Grhpr	0.28	3.13E-02	4.44E-02
7195	ENSMUSG00000034480	Diaph2	-0.46	3.13E-02	4.45E-02
7196	ENSMUSG00000113030	CT009754.1	0.34	3.14E-02	4.47E-02
7197	ENSMUSG00000031023	Akip1	0.33	3.15E-02	4.47E-02

7198	ENSMUSG00000044197	Gpr146	-0.35	3.15E-02	4.48E-02
7199	ENSMUSG00000004233	Wars2	0.43	3.16E-02	4.49E-02
7200	ENSMUSG00000028878	Fam76a	-0.35	3.17E-02	4.50E-02
7201	ENSMUSG00000014245	Pigl	-0.38	3.17E-02	4.50E-02
7202	ENSMUSG00000026721	Rabgap1l	-0.38	3.17E-02	4.50E-02
7203	ENSMUSG00000032601	Prkar2a	-0.34	3.18E-02	4.51E-02
7204	ENSMUSG00000029524	Sirt4	0.42	3.18E-02	4.52E-02
7205	ENSMUSG00000024843	Chka	-0.45	3.19E-02	4.52E-02
7206	ENSMUSG00000018548	Trim37	-0.40	3.19E-02	4.53E-02
7207	ENSMUSG00000041231	Ublcp1	0.31	3.19E-02	4.53E-02
7208	ENSMUSG00000045409	Trim39	-0.47	3.20E-02	4.54E-02
7209	ENSMUSG00000017686	Rhot1	-0.32	3.20E-02	4.54E-02
7210	ENSMUSG00000006304	Arpc2	0.28	3.20E-02	4.54E-02
7211	ENSMUSG00000027684	Mecom	-0.52	3.21E-02	4.55E-02
7212	ENSMUSG00000006941	Eif1b	0.36	3.21E-02	4.56E-02
7213	ENSMUSG00000031467	Agpat5	0.35	3.22E-02	4.56E-02
7214	ENSMUSG00000052248	Zeb2os	-0.56	3.26E-02	4.63E-02
7215	ENSMUSG00000097577	6230400D17Rik	-0.46	3.27E-02	4.63E-02
7216	ENSMUSG00000037486	Asxl2	-0.45	3.27E-02	4.64E-02
7217	ENSMUSG00000000631	Myo18a	-0.35	3.28E-02	4.64E-02
7218	ENSMUSG00000027552	E2f5	0.42	3.29E-02	4.66E-02
7219	ENSMUSG00000022194	Pabpn1	0.32	3.30E-02	4.68E-02
7220	ENSMUSG00000036167	Pphln1	-0.36	3.31E-02	4.69E-02
7221	ENSMUSG00000032178	Ilf3	-0.38	3.31E-02	4.69E-02
7222	ENSMUSG00000041040	Fam117b	-0.49	3.36E-02	4.76E-02
7223	ENSMUSG00000022119	Rbm26	-0.42	3.37E-02	4.77E-02
7224	ENSMUSG00000069917	Hba-a2	0.34	3.38E-02	4.78E-02
7225	ENSMUSG00000090570	Gm17041	0.48	3.38E-02	4.79E-02
7226	ENSMUSG00000048647	Exd1	-0.53	3.39E-02	4.79E-02
7227	ENSMUSG00000033799	Fam208b	-0.54	3.39E-02	4.80E-02
7228	ENSMUSG00000094365	Gm21982	-0.30	3.40E-02	4.80E-02
7229	ENSMUSG00000029577	Ube3b	-0.30	3.42E-02	4.84E-02
7230	ENSMUSG00000054716	Zfp771	0.32	3.42E-02	4.84E-02
7231	ENSMUSG00000031823	Zdhhc7	0.28	3.43E-02	4.85E-02
7232	ENSMUSG00000027166	Dnajc24	0.40	3.43E-02	4.85E-02
7233	ENSMUSG00000042870	Tom1	-0.31	3.43E-02	4.85E-02
7234	ENSMUSG00000022863	Btg3	0.45	3.43E-02	4.85E-02
7235	ENSMUSG00000011958	Bnip2	-0.28	3.44E-02	4.86E-02
7236	ENSMUSG00000031754	Nudt21	0.38	3.45E-02	4.87E-02
7237	ENSMUSG00000056305	Usp39	-0.36	3.45E-02	4.87E-02
7238	ENSMUSG00000111371	AC164123.1	0.38	3.46E-02	4.88E-02
7239	ENSMUSG00000027351	Spred1	-0.48	3.46E-02	4.88E-02
7240	ENSMUSG00000032745	Gpbp1	-0.35	3.46E-02	4.89E-02
7241	ENSMUSG00000002948	Map2k7	-0.32	3.46E-02	4.89E-02
7242	ENSMUSG00000023919	Cenpq	-0.45	3.47E-02	4.91E-02
7243	ENSMUSG00000021681	Aggf1	0.33	3.48E-02	4.91E-02
7244	ENSMUSG00000033054	Npat	-0.52	3.48E-02	4.91E-02
7245	ENSMUSG00000087013	2610027K06Rik	-0.43	3.48E-02	4.91E-02
7246	ENSMUSG00000031442	Mcf2l	-0.47	3.48E-02	4.92E-02
7247	ENSMUSG00000021810	Ecd	-0.29	3.49E-02	4.92E-02

7248	ENSMUSG00000035725	Prkx	-0.39	3.49E-02	4.93E-02
7249	ENSMUSG00000024477	Pggt1b	-0.44	3.49E-02	4.93E-02
7250	ENSMUSG00000037287	Tbcel	-0.46	3.50E-02	4.93E-02
7251	ENSMUSG00000036391	Sec24a	0.39	3.50E-02	4.93E-02
7252	ENSMUSG00000018509	Cenpv	0.32	3.50E-02	4.94E-02
7253	ENSMUSG00000002968	Med25	-0.32	3.51E-02	4.94E-02
7254	ENSMUSG00000027580	Helz2	-0.45	3.52E-02	4.96E-02
7255	ENSMUSG00000045248	Med26	-0.46	3.52E-02	4.96E-02
7256	ENSMUSG00000028096	Gpr89	0.32	3.52E-02	4.96E-02
7257	ENSMUSG00000038705	Gmeb2	-0.45	3.52E-02	4.97E-02
7258	ENSMUSG00000029291	Rufy3	-0.41	3.52E-02	4.97E-02
7259	ENSMUSG00000013646	Sh3bp5l	-0.29	3.53E-02	4.97E-02
7260	ENSMUSG00000021051	Ppp2r5e	-0.44	3.53E-02	4.98E-02
7261	ENSMUSG00000052997	Uba2	-0.30	3.53E-02	4.98E-02
7262	ENSMUSG00000078786	BC024978	-0.42	3.54E-02	4.98E-02
7263	ENSMUSG00000042447	Mios	-0.40	3.54E-02	4.98E-02
7264	ENSMUSG00000020235	Fzr1	-0.31	3.54E-02	4.98E-02
7265	ENSMUSG00000031144	Syp	0.42	3.54E-02	4.99E-02

Supplementary Table 4. List of overlapping differentially expressed genes among all 3 datasets.

number	ID human	ID mouse	Gene symbol
1	ENSG00000128274	ENSMUSG00000047878	*A4GALT*
2	ENSG00000107331	ENSMUSG00000026944	*ABCA2*
3	ENSG00000131269	ENSMUSG00000031333	*ABCB7*
4	ENSG00000114770	ENSMUSG00000022822	*ABCC5*
5	ENSG00000117528	ENSMUSG00000028127	*ABCD3*
6	ENSG00000060971	ENSMUSG00000010651	*ACAA1*
7	ENSG00000167315	ENSMUSG00000036880	*ACAA2*
8	ENSG00000115361	ENSMUSG00000026003	*ACADL*
9	ENSG00000117054	ENSMUSG00000062908	*ACADM*
10	ENSG00000122971	ENSMUSG00000029545	*ACADS*
11	ENSG00000196177	ENSMUSG00000030861	*ACADSB*
12	ENSG00000072778	ENSMUSG00000018574	*ACADVL*
13	ENSG00000114331	ENSMUSG00000049076	*ACAP2*
14	ENSG00000100412	ENSMUSG00000022477	*ACO2*
15	ENSG00000167107	ENSMUSG00000076435	*ACSF2*
16	ENSG00000197142	ENSMUSG00000024981	*ACSL5*
17	ENSG00000115170	ENSMUSG00000026836	*ACVR1*
18	ENSG00000123612	ENSMUSG00000026834	*ACVR1C*
19	ENSG00000142303	ENSMUSG00000024299	*ADAMTS10*
20	ENSG00000158859	ENSMUSG00000006403	*ADAMTS4*
21	ENSG00000163638	ENSMUSG00000030022	*ADAMTS9*
22	ENSG00000138031	ENSMUSG00000020654	*ADCY3*
23	ENSG00000185100	ENSMUSG00000011148	*ADSSL1*
24	ENSG00000141385	ENSMUSG00000024527	*AFG3L2*
25	ENSG00000187546	ENSMUSG00000050103	*AGMO*
26	ENSG00000124942	ENSMUSG00000069833	*AHNAK*
27	ENSG00000156709	ENSMUSG00000036932	*AIFM1*
28	ENSG00000121057	ENSMUSG00000018428	*AKAP1*
29	ENSG00000131016	ENSMUSG00000038587	*AKAP12*
30	ENSG00000085662	ENSMUSG00000001642	*AKR1B1*
31	ENSG00000023330	ENSMUSG00000032786	*ALAS1*
32	ENSG00000144908	ENSMUSG00000030088	*ALDH1L1*
33	ENSG00000119711	ENSMUSG00000021238	*ALDH6A1*
34	ENSG00000242110	ENSMUSG00000022244	*AMACR*
35	ENSG00000166025	ENSMUSG00000013076	*AMOTL1*
36	ENSG00000114019	ENSMUSG00000032531	*AMOTL2*
37	ENSG00000154122	ENSMUSG00000022265	*ANKH*
38	ENSG00000186106	ENSMUSG00000048307	*ANKRD46*
39	ENSG00000177119	ENSMUSG00000064210	*ANO6*
40	ENSG00000154856	ENSMUSG00000071847	*APCDD1*
41	ENSG00000169083	ENSMUSG00000046532	*AR*
42	ENSG00000107863	ENSMUSG00000036591	*ARHGAP21*
43	ENSG00000275832	ENSMUSG00000049807	*ARHGAP23*

44	ENSG00000031081	ENSMUSG00000022799	*ARHGAP31*
45	ENSG00000129675	ENSMUSG00000031133	*ARHGEF6*
46	ENSG00000150347	ENSMUSG00000019947	*ARID5B*
47	ENSG00000135931	ENSMUSG00000062590	*ARMC9*
48	ENSG00000143437	ENSMUSG00000015522	*ARNT*
49	ENSG00000104763	ENSMUSG00000031591	*ASAH1*
50	ENSG00000133657	ENSMUSG00000022533	*ATP13A3*
51	ENSG00000110955	ENSMUSG00000025393	*ATP5F1B*
52	ENSG00000165629	ENSMUSG00000025781	*ATP5F1C*
53	ENSG00000124172	ENSMUSG00000016252	*ATP5F1E*
54	ENSG00000169020	ENSMUSG00000050856	*ATP5ME*
55	ENSG00000156411	ENSMUSG00000021290	*ATP5MPL*
56	ENSG00000116459	ENSMUSG00000000563	*ATP5PB*
57	ENSG00000154723	ENSMUSG00000022890	*ATP5PF*
58	ENSG00000123472	ENSMUSG00000028710	*ATPAF1*
59	ENSG00000148090	ENSMUSG00000021460	*AUH*
60	ENSG00000115307	ENSMUSG00000068328	*AUP1*
61	ENSG00000185825	ENSMUSG00000002015	*BCAP31*
62	ENSG00000083123	ENSMUSG00000032263	*BCKDHB*
63	ENSG00000186174	ENSMUSG00000063382	*BCL9L*
64	ENSG00000176171	ENSMUSG00000078566	*BNIP3*
65	ENSG00000163170	ENSMUSG00000045160	*BOLA3*
66	ENSG00000137274	ENSMUSG00000038286	*BPHL*
67	ENSG00000169925	ENSMUSG00000026918	*BRD3*
68	ENSG00000172270	ENSMUSG00000023175	*BSG*
69	ENSG00000154640	ENSMUSG00000022863	*BTG3*
70	ENSG00000197982	ENSMUSG00000078570	*C1orf122*
71	ENSG00000119280	ENSMUSG00000031983	*C1orf198*
72	ENSG00000143612	ENSMUSG00000027942	*C1orf43*
73	ENSG00000108561	ENSMUSG00000018446	*C1QBP*
74	ENSG00000173918	ENSMUSG00000017446	*C1QTNF1*
75	ENSG00000159403	ENSMUSG00000055172	*C1R*
76	ENSG00000182326	ENSMUSG00000038521	*C1S*
77	ENSG00000244731	ENSMUSG00000073418	*C4A*
78	ENSG00000224389	ENSMUSG00000073418	*C4B*
79	ENSG00000113583	ENSMUSG00000036275	*C5orf15*
80	ENSG00000134508	ENSMUSG00000040957	*CABLES1*
81	ENSG00000158966	ENSMUSG00000028532	*CACHD1*
82	ENSG00000182389	ENSMUSG00000017412	*CACNB4*
83	ENSG00000014216	ENSMUSG00000024942	*CAPN1*
84	ENSG00000077549	ENSMUSG00000028745	*CAPZB*
85	ENSG00000177469	ENSMUSG00000004044	*CAVIN1*
86	ENSG00000168497	ENSMUSG00000045954	*CAVIN2*
87	ENSG00000253276	ENSMUSG00000090946	*CCDC71L*
88	ENSG00000091986	ENSMUSG00000022665	*CCDC80*
89	ENSG00000128283	ENSMUSG00000049521	*CDC42EP1*

90	ENSG00000147883	ENSMUSG00000073802	*CDKN2B*
91	ENSG00000129596	ENSMUSG00000033022	*CDO1*
92	ENSG00000064309	ENSMUSG00000038119	*CDON*
93	ENSG00000250479	ENSMUSG00000049422	*CHCHD10*
94	ENSG00000106554	ENSMUSG00000053768	*CHCHD3*
95	ENSG00000111666	ENSMUSG00000060002	*CHPT1*
96	ENSG00000166595	ENSMUSG00000031879	*CIAO2B*
97	ENSG00000176194	ENSMUSG00000024526	*CIDEA*
98	ENSG00000164442	ENSMUSG00000039910	*CITED2*
99	ENSG00000166165	ENSMUSG00000001270	*CKB*
100	ENSG00000139182	ENSMUSG00000008153	*CLSTN3*
101	ENSG00000132361	ENSMUSG00000020741	*CLUH*
102	ENSG00000183978	ENSMUSG00000017188	*COA3*
103	ENSG00000162377	ENSMUSG00000048351	*COA7*
104	ENSG00000082438	ENSMUSG00000034903	*COBLL1*
105	ENSG00000108821	ENSMUSG00000001506	*COL1A1*
106	ENSG00000164692	ENSMUSG00000029661	*COL1A2*
107	ENSG00000168542	ENSMUSG00000026043	*COL3A1*
108	ENSG00000142156	ENSMUSG00000001119	*COL6A1*
109	ENSG00000142173	ENSMUSG00000020241	*COL6A2*
110	ENSG00000163359	ENSMUSG00000048126	*COL6A3*
111	ENSG00000141030	ENSMUSG00000019373	*COPS3*
112	ENSG00000110871	ENSMUSG00000041733	*COQ5*
113	ENSG00000119723	ENSMUSG00000021235	*COQ6*
114	ENSG00000167186	ENSMUSG00000030652	*COQ7*
115	ENSG00000088682	ENSMUSG00000031782	*COQ9*
116	ENSG00000138495	ENSMUSG00000095464	*COX17*
117	ENSG00000163626	ENSMUSG00000035505	*COX18*
118	ENSG00000131143	ENSMUSG00000031818	*COX4I1*
119	ENSG00000178741	ENSMUSG00000000088	*COX5A*
120	ENSG00000135940	ENSMUSG00000079941	*COX5B*
121	ENSG00000111775	ENSMUSG00000041697	*COX6A1*
122	ENSG00000126267	ENSMUSG00000036751	*COX6B1*
123	ENSG00000164919	ENSMUSG00000014313	*COX6C*
124	ENSG00000161281	ENSMUSG00000074218	*COX7A1*
125	ENSG00000112695	ENSMUSG00000032330	*COX7A2*
126	ENSG00000131174	ENSMUSG00000031231	*COX7B*
127	ENSG00000127184	ENSMUSG00000017778	*COX7C*
128	ENSG00000176340	ENSMUSG00000035885	*COX8A*
129	ENSG00000140848	ENSMUSG00000034361	*CPNE2*
130	ENSG00000205560	ENSMUSG00000078937	*CPT1B*
131	ENSG00000157184	ENSMUSG00000028607	*CPT2*
132	ENSG00000095321	ENSMUSG00000026853	*CRAT*
133	ENSG00000182158	ENSMUSG00000038648	*CREB3L2*
134	ENSG00000150938	ENSMUSG00000024074	*CRIM1*
135	ENSG00000088766	ENSMUSG00000027357	*CRLS1*

136	ENSG00000121671	ENSMUSG00000068742	*CRY2*
137	ENSG00000062485	ENSMUSG00000005683	*CS*
138	ENSG00000109861	ENSMUSG00000030560	*CTSC*
139	ENSG00000117984	ENSMUSG00000007891	*CTSD*
140	ENSG00000085733	ENSMUSG00000031078	*CTTN*
141	ENSG00000257923	ENSMUSG00000029705	*CUX1*
142	ENSG00000179091	ENSMUSG00000022551	*CYC1*
143	ENSG00000172115	ENSMUSG00000063694	*CYCS*
144	ENSG00000153071	ENSMUSG00000022150	*DAB2*
145	ENSG00000132676	ENSMUSG00000068921	*DAP3*
146	ENSG00000117593	ENSMUSG00000026709	*DARS2*
147	ENSG00000011465	ENSMUSG00000019929	*DCN*
148	ENSG00000204843	ENSMUSG00000031865	*DCTN1*
149	ENSG00000203797	ENSMUSG00000063428	*DDO*
150	ENSG00000162733	ENSMUSG00000026674	*DDR2*
151	ENSG00000104325	ENSMUSG00000028223	*DECR1*
152	ENSG00000100418	ENSMUSG00000022472	*DESI1*
153	ENSG00000128185	ENSMUSG00000003531	*DGCR6L*
154	ENSG00000172893	ENSMUSG00000058454	*DHCR7*
155	ENSG00000278535	ENSMUSG00000034449	*DHRS11*
156	ENSG00000100612	ENSMUSG00000021094	*DHRS7*
157	ENSG00000109016	ENSMUSG00000042569	*DHRS7B*
158	ENSG00000150768	ENSMUSG00000000168	*DLAT*
159	ENSG00000091140	ENSMUSG00000020664	*DLD*
160	ENSG00000119689	ENSMUSG00000004789	*DLST*
161	ENSG00000137038	ENSMUSG00000028398	*DMAC1*
162	ENSG00000173253	ENSMUSG00000048138	*DMRT2*
163	ENSG00000103423	ENSMUSG00000004069	*DNAJA3*
164	ENSG00000162616	ENSMUSG00000028035	*DNAJB4*
165	ENSG00000175550	ENSMUSG00000024914	*DRAP1*
166	ENSG00000111817	ENSMUSG00000039497	*DSE*
167	ENSG00000110042	ENSMUSG00000039982	*DTX4*
168	ENSG00000143507	ENSMUSG00000039384	*DUSP10*
169	ENSG00000104823	ENSMUSG00000053898	*ECH1*
170	ENSG00000127884	ENSMUSG00000025465	*ECHS1*
171	ENSG00000167969	ENSMUSG00000024132	*ECI1*
172	ENSG00000130159	ENSMUSG00000066839	*ECSIT*
173	ENSG00000115380	ENSMUSG00000020467	*EFEMP1*
174	ENSG00000103966	ENSMUSG00000027293	*EHD4*
175	ENSG00000113790	ENSMUSG00000022853	*EHHADH*
176	ENSG00000149547	ENSMUSG00000062762	*EI24*
177	ENSG00000148730	ENSMUSG00000020091	*EIF4EBP2*
178	ENSG00000167136	ENSMUSG00000015337	*ENDOG*
179	ENSG00000106991	ENSMUSG00000026814	*ENG*
180	ENSG00000136960	ENSMUSG00000022425	*ENPP2*
181	ENSG00000164307	ENSMUSG00000021583	*ERAP1*

182	ENSG00000091831	ENSMUSG00000019768	*ESR1*
183	ENSG00000173153	ENSMUSG00000024955	*ESRRA*
184	ENSG00000140374	ENSMUSG00000032314	*ETFA*
185	ENSG00000105379	ENSMUSG00000004610	*ETFB*
186	ENSG00000171503	ENSMUSG00000027809	*ETFDH*
187	ENSG00000205707	ENSMUSG00000040370	*ETFRF1*
188	ENSG00000121769	ENSMUSG00000028773	*FABP3*
189	ENSG00000170323	ENSMUSG00000062515	*FABP4*
190	ENSG00000180185	ENSMUSG00000045316	*FAHD1*
191	ENSG00000197712	ENSMUSG00000029185	*FAM114A1*
192	ENSG00000035141	ENSMUSG00000057497	*FAM136A*
193	ENSG00000162391	ENSMUSG00000034871	*FAM151A*
194	ENSG00000123575	ENSMUSG00000042595	*FAM199X*
195	ENSG00000177150	ENSMUSG00000038121	*FAM210A*
196	ENSG00000178761	ENSMUSG00000032305	*FAM219B*
197	ENSG00000164896	ENSMUSG00000028959	*FASTK*
198	ENSG00000134452	ENSMUSG00000058594	*FBH1*
199	ENSG00000163520	ENSMUSG00000064080	*FBLN2*
200	ENSG00000140092	ENSMUSG00000021186	*FBLN5*
201	ENSG00000137714	ENSMUSG00000032051	*FDX1*
202	ENSG00000066926	ENSMUSG00000024588	*FECH*
203	ENSG00000066468	ENSMUSG00000030849	*FGFR2*
204	ENSG00000091483	ENSMUSG00000026526	*FH*
205	ENSG00000022267	ENSMUSG00000023092	*FHL1*
206	ENSG00000214253	ENSMUSG00000019054	*FIS1*
207	ENSG00000122642	ENSMUSG00000029781	*FKBP9*
208	ENSG00000160688	ENSMUSG00000042642	*FLAD1*
209	ENSG00000102755	ENSMUSG00000029648	*FLT1*
210	ENSG00000257365	ENSMUSG00000033373	*FNTB*
211	ENSG00000110074	ENSMUSG00000039048	*FOXRED1*
212	ENSG00000163430	ENSMUSG00000022816	*FSTL1*
213	ENSG00000140564	ENSMUSG00000030530	*FURIN*
214	ENSG00000139112	ENSMUSG00000030161	*GABARAPL1*
215	ENSG00000116717	ENSMUSG00000036390	*GADD45A*
216	ENSG00000280071	ENSMUSG00000053329	*GATD3B*
217	ENSG00000006007	ENSMUSG00000033917	*GDE1*
218	ENSG00000168827	ENSMUSG00000027774	*GFM1*
219	ENSG00000165678	ENSMUSG00000041028	*GHITM*
220	ENSG00000198814	ENSMUSG00000025059	*GK*
221	ENSG00000090863	ENSMUSG00000003316	*GLG1*
222	ENSG00000126603	ENSMUSG00000014303	*GLIS2*
223	ENSG00000182512	ENSMUSG00000021102	*GLRX5*
224	ENSG00000115419	ENSMUSG00000026103	*GLS*
225	ENSG00000148672	ENSMUSG00000021794	*GLUD1*
226	ENSG00000087460	ENSMUSG00000027523	*GNAS*
227	ENSG00000186469	ENSMUSG00000043004	*GNG2*

228	ENSG00000152642	ENSMUSG00000050627	*GPD1L*
229	ENSG00000115159	ENSMUSG00000026827	*GPD2*
230	ENSG00000167701	ENSMUSG00000022546	*GPT*
231	ENSG00000109519	ENSMUSG00000029198	*GRPEL1*
232	ENSG00000148180	ENSMUSG00000026879	*GSN*
233	ENSG00000104687	ENSMUSG00000031584	*GSR*
234	ENSG00000100983	ENSMUSG00000027610	*GSS*
235	ENSG00000197448	ENSMUSG00000029864	*GSTK1*
236	ENSG00000151806	ENSMUSG00000029208	*GUF1*
237	ENSG00000144366	ENSMUSG00000056870	*GULP1*
238	ENSG00000049239	ENSMUSG00000028980	*H6PD*
239	ENSG00000138796	ENSMUSG00000027984	*HADH*
240	ENSG00000084754	ENSMUSG00000025745	*HADHA*
241	ENSG00000138029	ENSMUSG00000059447	*HADHB*
242	ENSG00000063854	ENSMUSG00000024158	*HAGH*
243	ENSG00000214367	ENSMUSG00000079555	*HAUS3*
244	ENSG00000061273	ENSMUSG00000022475	*HDAC7*
245	ENSG00000111906	ENSMUSG00000000295	*HDDC2*
246	ENSG00000198130	ENSMUSG00000041426	*HIBCH*
247	ENSG00000100644	ENSMUSG00000021109	*HIF1A*
248	ENSG00000146066	ENSMUSG00000025868	*HIGD2A*
249	ENSG00000064393	ENSMUSG00000061436	*HIPK2*
250	ENSG00000071794	ENSMUSG00000002428	*HLTF*
251	ENSG00000117305	ENSMUSG00000028672	*HMGCL*
252	ENSG00000134240	ENSMUSG00000027875	*HMGCS2*
253	ENSG00000106004	ENSMUSG00000038253	*HOXA5*
254	ENSG00000197757	ENSMUSG00000001661	*HOXC6*
255	ENSG00000164120	ENSMUSG00000031613	*HPGD*
256	ENSG00000196196	ENSMUSG00000071001	*HRCT1*
257	ENSG00000072506	ENSMUSG00000025260	*HSD17B10*
258	ENSG00000149084	ENSMUSG00000027195	*HSD17B12*
259	ENSG00000119471	ENSMUSG00000028383	*HSDL2*
260	ENSG00000113013	ENSMUSG00000024359	*HSPA9*
261	ENSG00000152137	ENSMUSG00000041548	*HSPB8*
262	ENSG00000142798	ENSMUSG00000028763	*HSPG2*
263	ENSG00000166033	ENSMUSG00000006205	*HTRA1*
264	ENSG00000067704	ENSMUSG00000026618	*IARS2*
265	ENSG00000115738	ENSMUSG00000020644	*ID2*
266	ENSG00000166411	ENSMUSG00000032279	*IDH3A*
267	ENSG00000101365	ENSMUSG00000027406	*IDH3B*
268	ENSG00000067829	ENSMUSG00000002010	*IDH3G*
269	ENSG00000017427	ENSMUSG00000020053	*IGF1*
270	ENSG00000077238	ENSMUSG00000030748	*IL4R*
271	ENSG00000160712	ENSMUSG00000027947	*IL6R*
272	ENSG00000134352	ENSMUSG00000021756	*IL6ST*
273	ENSG00000105135	ENSMUSG00000032763	*ILVBL*

274	ENSG00000132305	ENSMUSG00000052337	*IMMT*
275	ENSG00000163083	ENSMUSG00000037035	*INHBB*
276	ENSG00000135070	ENSMUSG00000044792	*ISCA1*
277	ENSG00000129009	ENSMUSG00000037206	*ISLR*
278	ENSG00000066583	ENSMUSG00000024601	*ISOC1*
279	ENSG00000161638	ENSMUSG00000000555	*ITGA5*
280	ENSG00000123243	ENSMUSG00000025780	*ITIH5*
281	ENSG00000205726	ENSMUSG00000022957	*ITSN1*
282	ENSG00000008083	ENSMUSG00000038518	*JARID2*
283	ENSG00000153814	ENSMUSG00000063568	*JAZF1*
284	ENSG00000140044	ENSMUSG00000034271	*JDP2*
285	ENSG00000158445	ENSMUSG00000050556	*KCNB1*
286	ENSG00000100890	ENSMUSG00000021023	*KIAA0391*
287	ENSG00000122203	ENSMUSG00000025871	*KIAA1191*
288	ENSG00000174718	ENSMUSG00000032712	*KIAA1551*
289	ENSG00000129250	ENSMUSG00000020821	*KIF1C*
290	ENSG00000147592	ENSMUSG00000025937	*LACTB2*
291	ENSG00000172037	ENSMUSG00000052911	*LAMB2*
292	ENSG00000011376	ENSMUSG00000035202	*LARS2*
293	ENSG00000002834	ENSMUSG00000038366	*LASP1*
294	ENSG00000150457	ENSMUSG00000021959	*LATS2*
295	ENSG00000111716	ENSMUSG00000030246	*LDHB*
296	ENSG00000179241	ENSMUSG00000048058	*LDLRAD3*
297	ENSG00000168924	ENSMUSG00000005299	*LETM1*
298	ENSG00000050426	ENSMUSG00000037353	*LETMD1*
299	ENSG00000145685	ENSMUSG00000045312	*LHFPL2*
300	ENSG00000121897	ENSMUSG00000029199	*LIAS*
301	ENSG00000050405	ENSMUSG00000023022	*LIMA1*
302	ENSG00000064042	ENSMUSG00000037736	*LIMCH1*
303	ENSG00000160789	ENSMUSG00000028063	*LMNA*
304	ENSG00000143013	ENSMUSG00000028266	*LMO4*
305	ENSG00000175445	ENSMUSG00000015568	*LPL*
306	ENSG00000144749	ENSMUSG00000030029	*LRIG1*
307	ENSG00000139263	ENSMUSG00000020105	*LRIG3*
308	ENSG00000123384	ENSMUSG00000040249	*LRP1*
309	ENSG00000138095	ENSMUSG00000024120	*LRPPRC*
310	ENSG00000139329	ENSMUSG00000036446	*LUM*
311	ENSG00000180155	ENSMUSG00000022594	*LYNX1*
312	ENSG00000120992	ENSMUSG00000025903	*LYPLA1*
313	ENSG00000156928	ENSMUSG00000029815	*MALSU1*
314	ENSG00000112893	ENSMUSG00000024085	*MAN2A1*
315	ENSG00000189221	ENSMUSG00000025037	*MAOA*
316	ENSG00000095015	ENSMUSG00000021754	*MAP3K1*
317	ENSG00000047849	ENSMUSG00000032479	*MAP4*
318	ENSG00000099308	ENSMUSG00000031833	*MAST3*
319	ENSG00000078070	ENSMUSG00000027709	*MCCC1*

320	ENSG00000131844	ENSMUSG00000021646	*MCCC2*
321	ENSG00000172366	ENSMUSG00000025732	*MCRIP2*
322	ENSG00000014641	ENSMUSG00000020321	*MDH1*
323	ENSG00000146701	ENSMUSG00000019179	*MDH2*
324	ENSG00000151376	ENSMUSG00000030621	*ME3*
325	ENSG00000116353	ENSMUSG00000028910	*MECR*
326	ENSG00000104973	ENSMUSG00000002968	*MED25*
327	ENSG00000102802	ENSMUSG00000029659	*MEDAG*
328	ENSG00000068305	ENSMUSG00000030557	*MEF2A*
329	ENSG00000105976	ENSMUSG00000009376	*MET*
330	ENSG00000140545	ENSMUSG00000030605	*MFGE8*
331	ENSG00000154305	ENSMUSG00000056050	*MIA3*
332	ENSG00000125863	ENSMUSG00000027274	*MKKS*
333	ENSG00000103150	ENSMUSG00000074064	*MLYCD*
334	ENSG00000102996	ENSMUSG00000031790	*MMP15*
335	ENSG00000124615	ENSMUSG00000064120	*MOCS1*
336	ENSG00000143158	ENSMUSG00000026568	*MPC2*
337	ENSG00000129255	ENSMUSG00000018761	*MPDU1*
338	ENSG00000150054	ENSMUSG00000057440	*MPP7*
339	ENSG00000169288	ENSMUSG00000029486	*MRPL1*
340	ENSG00000262814	ENSMUSG00000039640	*MRPL12*
341	ENSG00000166902	ENSMUSG00000024683	*MRPL16*
342	ENSG00000115364	ENSMUSG00000030045	*MRPL19*
343	ENSG00000112651	ENSMUSG00000002767	*MRPL2*
344	ENSG00000082515	ENSMUSG00000020514	*MRPL22*
345	ENSG00000086504	ENSMUSG00000024181	*MRPL28*
346	ENSG00000114686	ENSMUSG00000032563	*MRPL3*
347	ENSG00000130312	ENSMUSG00000034880	*MRPL34*
348	ENSG00000171421	ENSMUSG00000021607	*MRPL36*
349	ENSG00000116221	ENSMUSG00000028622	*MRPL37*
350	ENSG00000204316	ENSMUSG00000020775	*MRPL38*
351	ENSG00000154719	ENSMUSG00000022889	*MRPL39*
352	ENSG00000185608	ENSMUSG00000022706	*MRPL40*
353	ENSG00000182154	ENSMUSG00000036850	*MRPL41*
354	ENSG00000135900	ENSMUSG00000026248	*MRPL44*
355	ENSG00000259494	ENSMUSG00000030612	*MRPL46*
356	ENSG00000136522	ENSMUSG00000037531	*MRPL47*
357	ENSG00000136897	ENSMUSG00000044018	*MRPL50*
358	ENSG00000111639	ENSMUSG00000030335	*MRPL51*
359	ENSG00000204822	ENSMUSG00000030037	*MRPL53*
360	ENSG00000167862	ENSMUSG00000018858	*MRPL58*
361	ENSG00000048544	ENSMUSG00000034729	*MRPS10*
362	ENSG00000181991	ENSMUSG00000030611	*MRPS11*
363	ENSG00000116898	ENSMUSG00000028861	*MRPS15*
364	ENSG00000182180	ENSMUSG00000049960	*MRPS16*
365	ENSG00000204568	ENSMUSG00000024436	*MRPS18B*

366	ENSG00000175110	ENSMUSG00000032459	*MRPS22*
367	ENSG00000181610	ENSMUSG00000023723	*MRPS23*
368	ENSG00000062582	ENSMUSG00000020477	*MRPS24*
369	ENSG00000131368	ENSMUSG00000014551	*MRPS25*
370	ENSG00000113048	ENSMUSG00000041632	*MRPS27*
371	ENSG00000147586	ENSMUSG00000040269	*MRPS28*
372	ENSG00000102738	ENSMUSG00000031533	*MRPS31*
373	ENSG00000090263	ENSMUSG00000029918	*MRPS33*
374	ENSG00000074071	ENSMUSG00000038880	*MRPS34*
375	ENSG00000144029	ENSMUSG00000027374	*MRPS5*
376	ENSG00000125445	ENSMUSG00000046756	*MRPS7*
377	ENSG00000148187	ENSMUSG00000026887	*MRRF*
378	ENSG00000153944	ENSMUSG00000069769	*MSI2*
379	ENSG00000175806	ENSMUSG00000054733	*MSRA*
380	ENSG00000198736	ENSMUSG00000075705	*MSRB1*
381	ENSG00000148450	ENSMUSG00000023094	*MSRB2*
382	ENSG00000174099	ENSMUSG00000051236	*MSRB3*
383	ENSG00000149480	ENSMUSG00000071646	*MTA2*
384	ENSG00000198899	ENSMUSG00000064357	*MT-ATP6*
385	ENSG00000228253	ENSMUSG00000064356	*MT-ATP8*
386	ENSG00000137409	ENSMUSG00000024012	*MTCH1*
387	ENSG00000109919	ENSMUSG00000027282	*MTCH2*
388	ENSG00000198712	ENSMUSG00000064354	*MT-CO2*
389	ENSG00000198938	ENSMUSG00000064358	*MT-CO3*
390	ENSG00000156469	ENSMUSG00000021519	*MTERF3*
391	ENSG00000242114	ENSMUSG00000004748	*MTFP1*
392	ENSG00000085760	ENSMUSG00000020459	*MTIF2*
393	ENSG00000175701	ENSMUSG00000051319	*MTLN*
394	ENSG00000162576	ENSMUSG00000029070	*MXRA8*
395	ENSG00000179820	ENSMUSG00000068566	*MYADM*
396	ENSG00000196465	ENSMUSG00000039824	*MYL6B*
397	ENSG00000196535	ENSMUSG00000000631	*MYO18A*
398	ENSG00000152620	ENSMUSG00000022253	*NADK2*
399	ENSG00000105835	ENSMUSG00000020572	*NAMPT*
400	ENSG00000187109	ENSMUSG00000058799	*NAP1L1*
401	ENSG00000270170	ENSMUSG00000107002	*NCBP2-AS2*
402	ENSG00000104419	ENSMUSG00000005125	*NDRG1*
403	ENSG00000165795	ENSMUSG00000004558	*NDRG2*
404	ENSG00000130414	ENSMUSG00000026260	*NDUFA10*
405	ENSG00000186010	ENSMUSG00000036199	*NDUFA13*
406	ENSG00000131495	ENSMUSG00000014294	*NDUFA2*
407	ENSG00000170906	ENSMUSG00000035674	*NDUFA3*
408	ENSG00000189043	ENSMUSG00000029632	*NDUFA4*
409	ENSG00000139180	ENSMUSG00000000399	*NDUFA9*
410	ENSG00000137806	ENSMUSG00000027305	*NDUFAF1*
411	ENSG00000164182	ENSMUSG00000068184	*NDUFAF2*

412	ENSG00000140990	ENSMUSG00000040048	*NDUFB10*
413	ENSG00000147123	ENSMUSG00000031059	*NDUFB11*
414	ENSG00000090266	ENSMUSG00000002416	*NDUFB2*
415	ENSG00000166136	ENSMUSG00000025204	*NDUFB8*
416	ENSG00000147684	ENSMUSG00000022354	*NDUFB9*
417	ENSG00000151366	ENSMUSG00000030647	*NDUFC2*
418	ENSG00000023228	ENSMUSG00000025968	*NDUFS1*
419	ENSG00000158864	ENSMUSG00000013593	*NDUFS2*
420	ENSG00000213619	ENSMUSG00000005510	*NDUFS3*
421	ENSG00000168653	ENSMUSG00000028648	*NDUFS5*
422	ENSG00000178127	ENSMUSG00000024099	*NDUFV2*
423	ENSG00000160194	ENSMUSG00000024038	*NDUFV3*
424	ENSG00000172260	ENSMUSG00000040037	*NEGR1*
425	ENSG00000141905	ENSMUSG00000055053	*NFIC*
426	ENSG00000165030	ENSMUSG00000056749	*NFIL3*
427	ENSG00000008441	ENSMUSG00000001911	*NFIX*
428	ENSG00000169599	ENSMUSG00000029993	*NFU1*
429	ENSG00000146729	ENSMUSG00000029432	*NIPSNAP2*
430	ENSG00000103226	ENSMUSG00000030835	*NOMO3*
431	ENSG00000134250	ENSMUSG00000027878	*NOTCH2*
432	ENSG00000113580	ENSMUSG00000024431	*NR3C1*
433	ENSG00000185189	ENSMUSG00000075590	*NRBP2*
434	ENSG00000180530	ENSMUSG00000048490	*NRIP1*
435	ENSG00000178425	ENSMUSG00000039480	*NT5DC1*
436	ENSG00000148053	ENSMUSG00000055254	*NTRK2*
437	ENSG00000105245	ENSMUSG00000063160	*NUMBL*
438	ENSG00000176046	ENSMUSG00000030717	*NUPR1*
439	ENSG00000105953	ENSMUSG00000020456	*OGDH*
440	ENSG00000144909	ENSMUSG00000022807	*OSBPL11*
441	ENSG00000155463	ENSMUSG00000000959	*OXA1L*
442	ENSG00000154814	ENSMUSG00000021906	*OXNAD1*
443	ENSG00000099864	ENSMUSG00000035863	*PALM*
444	ENSG00000145730	ENSMUSG00000026335	*PAM*
445	ENSG00000152782	ENSMUSG00000033610	*PANK1*
446	ENSG00000138801	ENSMUSG00000028032	*PAPSS1*
447	ENSG00000116117	ENSMUSG00000052062	*PARD3B*
448	ENSG00000114054	ENSMUSG00000032527	*PCCB*
449	ENSG00000169851	ENSMUSG00000029108	*PCDH7*
450	ENSG00000240184	ENSMUSG00000102918	*PCDHGC3*
451	ENSG00000124253	ENSMUSG00000027513	*PCK1*
452	ENSG00000106333	ENSMUSG00000029718	*PCOLCE*
453	ENSG00000152270	ENSMUSG00000030671	*PDE3B*
454	ENSG00000145431	ENSMUSG00000028019	*PDGFC*
455	ENSG00000131828	ENSMUSG00000031299	*PDHA1*
456	ENSG00000168291	ENSMUSG00000021748	*PDHB*
457	ENSG00000110435	ENSMUSG00000010914	*PDHX*

458	ENSG00000152256	ENSMUSG00000006494	*PDK1*
459	ENSG00000005882	ENSMUSG00000038967	*PDK2*
460	ENSG00000172840	ENSMUSG00000048371	*PDP2*
461	ENSG00000164494	ENSMUSG00000038240	*PDSS2*
462	ENSG00000133401	ENSMUSG00000022197	*PDZD2*
463	ENSG00000121440	ENSMUSG00000035357	*PDZRN3*
464	ENSG00000173517	ENSMUSG00000074305	*PEAK1*
465	ENSG00000139946	ENSMUSG00000021846	*PELI2*
466	ENSG00000034693	ENSMUSG00000019809	*PEX3*
467	ENSG00000152556	ENSMUSG00000033065	*PFKM*
468	ENSG00000142657	ENSMUSG00000028961	*PGD*
469	ENSG00000101856	ENSMUSG00000006373	*PGRMC1*
470	ENSG00000167085	ENSMUSG00000038845	*PHB*
471	ENSG00000164776	ENSMUSG00000025537	*PHKG1*
472	ENSG00000019144	ENSMUSG00000048537	*PHLDB1*
473	ENSG00000107537	ENSMUSG00000026664	*PHYH*
474	ENSG00000153823	ENSMUSG00000045658	*PID1*
475	ENSG00000087111	ENSMUSG00000041958	*PIGS*
476	ENSG00000107959	ENSMUSG00000021193	*PITRM1*
477	ENSG00000171033	ENSMUSG00000027499	*PKIA*
478	ENSG00000067225	ENSMUSG00000032294	*PKM*
479	ENSG00000057294	ENSMUSG00000041957	*PKP2*
480	ENSG00000123739	ENSMUSG00000027999	*PLA2G12A*
481	ENSG00000171680	ENSMUSG00000039713	*PLEKHG5*
482	ENSG00000105355	ENSMUSG00000024197	*PLIN3*
483	ENSG00000214456	ENSMUSG00000011305	*PLIN5*
484	ENSG00000145632	ENSMUSG00000021701	*PLK2*
485	ENSG00000105819	ENSMUSG00000029017	*PMPCB*
486	ENSG00000163344	ENSMUSG00000027952	*PMVK*
487	ENSG00000127838	ENSMUSG00000026179	*PNKD*
488	ENSG00000004142	ENSMUSG00000001100	*POLDIP2*
489	ENSG00000130997	ENSMUSG00000045102	*POLN*
490	ENSG00000013503	ENSMUSG00000034453	*POLR3B*
491	ENSG00000085998	ENSMUSG00000028700	*POMGNT1*
492	ENSG00000127948	ENSMUSG00000005514	*POR*
493	ENSG00000180817	ENSMUSG00000020089	*PPA1*
494	ENSG00000108179	ENSMUSG00000021868	*PPIF*
495	ENSG00000146112	ENSMUSG00000034595	*PPP1R18*
496	ENSG00000175470	ENSMUSG00000041769	*PPP2R2D*
497	ENSG00000196850	ENSMUSG00000038582	*PPTC7*
498	ENSG00000135617	ENSMUSG00000030008	*PRADC1*
499	ENSG00000167815	ENSMUSG00000005161	*PRDX2*
500	ENSG00000165672	ENSMUSG00000024997	*PRDX3*
501	ENSG00000126432	ENSMUSG00000024953	*PRDX5*
502	ENSG00000138073	ENSMUSG00000045302	*PREB*
503	ENSG00000101166	ENSMUSG00000016257	*PRELID3B*

504	ENSG00000188783	ENSMUSG00000041577	*PRELP*
505	ENSG00000072062	ENSMUSG00000005469	*PRKACA*
506	ENSG00000171132	ENSMUSG00000045038	*PRKCE*
507	ENSG00000100033	ENSMUSG00000003526	*PRODH*
508	ENSG00000184838	ENSMUSG00000073565	*PRR16*
509	ENSG00000186654	ENSMUSG00000036106	*PRR5*
510	ENSG00000106588	ENSMUSG00000015671	*PSMA2*
511	ENSG00000101182	ENSMUSG00000027566	*PSMA7*
512	ENSG00000132300	ENSMUSG00000063884	*PTCD3*
513	ENSG00000112531	ENSMUSG00000062078	*QKI*
514	ENSG00000116260	ENSMUSG00000033684	*QSOX1*
515	ENSG00000153179	ENSMUSG00000025795	*RASSF3*
516	ENSG00000169435	ENSMUSG00000029370	*RASSF6*
517	ENSG00000138207	ENSMUSG00000024990	*RBP4*
518	ENSG00000166831	ENSMUSG00000032387	*RBPMS2*
519	ENSG00000274523	ENSMUSG00000061979	*RCC1L*
520	ENSG00000115255	ENSMUSG00000035504	*REEP6*
521	ENSG00000165731	ENSMUSG00000030110	*RET*
522	ENSG00000042445	ENSMUSG00000056666	*RETSAT*
523	ENSG00000205517	ENSMUSG00000040146	*RGL3*
524	ENSG00000182175	ENSMUSG00000070509	*RGMA*
525	ENSG00000167705	ENSMUSG00000038195	*RILP*
526	ENSG00000137824	ENSMUSG00000070730	*RMDN3*
527	ENSG00000155906	ENSMUSG00000019763	*RMND1*
528	ENSG00000176641	ENSMUSG00000047496	*RNF152*
529	ENSG00000184719	ENSMUSG00000071573	*RNLS*
530	ENSG00000164967	ENSMUSG00000036114	*RPP25L*
531	ENSG00000085721	ENSMUSG00000022682	*RRN3*
532	ENSG00000133318	ENSMUSG00000024758	*RTN3*
533	ENSG00000143171	ENSMUSG00000015843	*RXRG*
534	ENSG00000100347	ENSMUSG00000022437	*SAMM50*
535	ENSG00000152700	ENSMUSG00000020386	*SAR1B*
536	ENSG00000143653	ENSMUSG00000038936	*SCCPDH*
537	ENSG00000133028	ENSMUSG00000069844	*SCO1*
538	ENSG00000073578	ENSMUSG00000021577	*SDHA*
539	ENSG00000117118	ENSMUSG00000009863	*SDHB*
540	ENSG00000143416	ENSMUSG00000068877	*SELENBP1*
541	ENSG00000138018	ENSMUSG00000075703	*SELENOI*
542	ENSG00000179918	ENSMUSG00000049091	*SEPHS2*
543	ENSG00000132824	ENSMUSG00000017707	*SERINC3*
544	ENSG00000149131	ENSMUSG00000023224	*SERPING1*
545	ENSG00000169976	ENSMUSG00000078348	*SF3B5*
546	ENSG00000101049	ENSMUSG00000017868	*SGK2*
547	ENSG00000147010	ENSMUSG00000040990	*SH3KBP1*
548	ENSG00000107957	ENSMUSG00000053617	*SH3PXD2A*
549	ENSG00000142082	ENSMUSG00000025486	*SIRT3*

550	ENSG00000155380	ENSMUSG00000032902	*SLC16A1*
551	ENSG00000146477	ENSMUSG00000023828	*SLC22A3*
552	ENSG00000197375	ENSMUSG00000018900	*SLC22A5*
553	ENSG00000108528	ENSMUSG00000014606	*SLC25A11*
554	ENSG00000125454	ENSMUSG00000020744	*SLC25A19*
555	ENSG00000178537	ENSMUSG00000032602	*SLC25A20*
556	ENSG00000171612	ENSMUSG00000028982	*SLC25A33*
557	ENSG00000162461	ENSMUSG00000040740	*SLC25A34*
558	ENSG00000013306	ENSMUSG00000018677	*SLC25A39*
559	ENSG00000151729	ENSMUSG00000031633	*SLC25A4*
560	ENSG00000181035	ENSMUSG00000002346	*SLC25A42*
561	ENSG00000005022	ENSMUSG00000016319	*SLC25A5*
562	ENSG00000122696	ENSMUSG00000045973	*SLC25A51*
563	ENSG00000186335	ENSMUSG00000020264	*SLC36A2*
564	ENSG00000133065	ENSMUSG00000013275	*SLC41A1*
565	ENSG00000166750	ENSMUSG00000054404	*SLFN5*
566	ENSG00000184347	ENSMUSG00000056427	*SLIT3*
567	ENSG00000166949	ENSMUSG00000032402	*SMAD3*
568	ENSG00000080503	ENSMUSG00000024921	*SMARCA2*
569	ENSG00000183172	ENSMUSG00000022452	*SMDT1*
570	ENSG00000198732	ENSMUSG00000021136	*SMOC1*
571	ENSG00000161981	ENSMUSG00000040767	*SNRNP25*
572	ENSG00000143376	ENSMUSG00000028136	*SNX27*
573	ENSG00000112335	ENSMUSG00000019804	*SNX3*
574	ENSG00000162627	ENSMUSG00000028007	*SNX7*
575	ENSG00000112096	ENSMUSG00000006818	*SOD2*
576	ENSG00000113140	ENSMUSG00000018593	*SPARC*
577	ENSG00000262655	ENSMUSG00000038156	*SPON1*
578	ENSG00000116096	ENSMUSG00000033735	*SPR*
579	ENSG00000164056	ENSMUSG00000037211	*SPRY1*
580	ENSG00000141298	ENSMUSG00000037926	*SSH2*
581	ENSG00000008513	ENSMUSG00000013846	*ST3GAL1*
582	ENSG00000138134	ENSMUSG00000024776	*STAMBPL1*
583	ENSG00000115415	ENSMUSG00000026104	*STAT1*
584	ENSG00000166888	ENSMUSG00000002147	*STAT6*
585	ENSG00000107960	ENSMUSG00000042694	*STN1*
586	ENSG00000165283	ENSMUSG00000028455	*STOML2*
587	ENSG00000023734	ENSMUSG00000030224	*STRAP*
588	ENSG00000136143	ENSMUSG00000022110	*SUCLA2*
589	ENSG00000163541	ENSMUSG00000052738	*SUCLG1*
590	ENSG00000064607	ENSMUSG00000036054	*SUGP2*
591	ENSG00000100242	ENSMUSG00000042524	*SUN2*
592	ENSG00000156502	ENSMUSG00000020079	*SUPV3L1*
593	ENSG00000148290	ENSMUSG00000015790	*SURF1*
594	ENSG00000165124	ENSMUSG00000028369	*SVEP1*
595	ENSG00000136463	ENSMUSG00000001983	*TACO1*

596	ENSG00000113407	ENSMUSG00000022241	*TARS*
597	ENSG00000143374	ENSMUSG00000028107	*TARS2*
598	ENSG00000145979	ENSMUSG00000021368	*TBC1D7*
599	ENSG00000136270	ENSMUSG00000000384	*TBRG4*
600	ENSG00000092607	ENSMUSG00000027868	*TBX15*
601	ENSG00000135111	ENSMUSG00000018604	*TBX3*
602	ENSG00000198420	ENSMUSG00000036667	*TCAF1*
603	ENSG00000148737	ENSMUSG00000024985	*TCF7L2*
604	ENSG00000166046	ENSMUSG00000020034	*TCP11L2*
605	ENSG00000099797	ENSMUSG00000031708	*TECR*
606	ENSG00000198959	ENSMUSG00000037820	*TGM2*
607	ENSG00000137801	ENSMUSG00000040152	*THBS1*
608	ENSG00000186340	ENSMUSG00000023885	*THBS2*
609	ENSG00000104980	ENSMUSG00000002949	*TIMM44*
610	ENSG00000105197	ENSMUSG00000003438	*TIMM50*
611	ENSG00000126953	ENSMUSG00000048007	*TIMM8A*
612	ENSG00000150779	ENSMUSG00000039016	*TIMM8B*
613	ENSG00000113845	ENSMUSG00000002846	*TIMMDC1*
614	ENSG00000035862	ENSMUSG00000017466	*TIMP2*
615	ENSG00000163931	ENSMUSG00000021957	*TKT*
616	ENSG00000137076	ENSMUSG00000028465	*TLN1*
617	ENSG00000171914	ENSMUSG00000052698	*TLN2*
618	ENSG00000135926	ENSMUSG00000006301	*TMBIM1*
619	ENSG00000117500	ENSMUSG00000063406	*TMED5*
620	ENSG00000100580	ENSMUSG00000034111	*TMED8*
621	ENSG00000166575	ENSMUSG00000039428	*TMEM135*
622	ENSG00000146859	ENSMUSG00000057137	*TMEM140*
623	ENSG00000244187	ENSMUSG00000026939	*TMEM141*
624	ENSG00000157600	ENSMUSG00000047045	*TMEM164*
625	ENSG00000150433	ENSMUSG00000032121	*TMEM218*
626	ENSG00000089063	ENSMUSG00000027341	*TMEM230*
627	ENSG00000153485	ENSMUSG00000046675	*TMEM251*
628	ENSG00000095209	ENSMUSG00000028420	*TMEM38B*
629	ENSG00000170876	ENSMUSG00000030095	*TMEM43*
630	ENSG00000109084	ENSMUSG00000037278	*TMEM97*
631	ENSG00000006042	ENSMUSG00000035413	*TMEM98*
632	ENSG00000182095	ENSMUSG00000039477	*TNRC18*
633	ENSG00000079308	ENSMUSG00000055322	*TNS1*
634	ENSG00000158882	ENSMUSG00000005674	*TOMM40L*
635	ENSG00000111669	ENSMUSG00000023456	*TPI1*
636	ENSG00000126602	ENSMUSG00000005981	*TRAP1*
637	ENSG00000072657	ENSMUSG00000050663	*TRHDE*
638	ENSG00000100106	ENSMUSG00000033088	*TRIOBP*
639	ENSG00000174173	ENSMUSG00000044763	*TRMT10C*
640	ENSG00000123297	ENSMUSG00000040521	*TSFM*
641	ENSG00000182463	ENSMUSG00000047907	*TSHZ2*

642	ENSG00000168234	ENSMUSG00000024424	*TTC39C*
643	ENSG00000114999	ENSMUSG00000027394	*TTL*
644	ENSG00000127824	ENSMUSG00000026202	*TUBA4A*
645	ENSG00000178952	ENSMUSG00000073838	*TUFM*
646	ENSG00000122691	ENSMUSG00000035799	*TWIST1*
647	ENSG00000233608	ENSMUSG00000007805	*TWIST2*
648	ENSG00000184470	ENSMUSG00000075704	*TXNRD2*
649	ENSG00000126261	ENSMUSG00000052997	*UBA2*
650	ENSG00000158062	ENSMUSG00000012126	*UBXN11*
651	ENSG00000109424	ENSMUSG00000031710	*UCP1*
652	ENSG00000101019	ENSMUSG00000005882	*UQCC1*
653	ENSG00000204922	ENSMUSG00000071654	*UQCC3*
654	ENSG00000184076	ENSMUSG00000059534	*UQCR10*
655	ENSG00000127540	ENSMUSG00000020163	*UQCR11*
656	ENSG00000156467	ENSMUSG00000021520	*UQCRB*
657	ENSG00000010256	ENSMUSG00000025651	*UQCRC1*
658	ENSG00000140740	ENSMUSG00000030884	*UQCRC2*
659	ENSG00000169021	ENSMUSG00000038462	*UQCRFS1*
660	ENSG00000173660	ENSMUSG00000063882	*UQCRH*
661	ENSG00000164405	ENSMUSG00000044894	*UQCRQ*
662	ENSG00000188690	ENSMUSG00000030979	*UROS*
663	ENSG00000111962	ENSMUSG00000047712	*UST*
664	ENSG00000165197	ENSMUSG00000031380	*VEGFD*
665	ENSG00000136059	ENSMUSG00000038775	*VILL*
666	ENSG00000100568	ENSMUSG00000021124	*VTI1B*
667	ENSG00000102763	ENSMUSG00000058997	*VWA8*
668	ENSG00000116729	ENSMUSG00000028173	*WLS*
669	ENSG00000114251	ENSMUSG00000021994	*WNT5A*
670	ENSG00000142279	ENSMUSG00000036459	*WTIP*
671	ENSG00000154767	ENSMUSG00000030094	*XPC*
672	ENSG00000173950	ENSMUSG00000047434	*XXYLT1*
673	ENSG00000137693	ENSMUSG00000053110	*YAP1*
674	ENSG00000065978	ENSMUSG00000028639	*YBX1*
675	ENSG00000163812	ENSMUSG00000025786	*ZDHHC3*
676	ENSG00000188706	ENSMUSG00000036985	*ZDHHC9*
677	ENSG00000186918	ENSMUSG00000034522	*ZNF395*
678	ENSG00000019995	ENSMUSG00000030967	*ZRANB1*
679	ENSG00000162378	ENSMUSG00000034636	*ZYG11B*

Supplementary Table 5. List of gene candidates selected for functional screening.

number	ID human	ID mouse	Gene symbol
1	ENSG00000167315	ENSMUSG00000036880	*ACAA2*
2	ENSG00000117054	ENSMUSG00000062908	*ACADM*
3	ENSG00000072778	ENSMUSG00000018574	*ACADVL*
4	ENSG00000100412	ENSMUSG00000022477	*ACO2*
5	ENSG00000197142	ENSMUSG00000024981	*ACSL5*
6	ENSG00000141385	ENSMUSG00000024527	*AFG3L2*
7	ENSG00000121057	ENSMUSG00000018428	*AKAP1*
8	ENSG00000163170	ENSMUSG00000045160	*BOLA3*
9	ENSG00000106554	ENSMUSG00000053768	*CHCHD3*
10	ENSG00000183978	ENSMUSG00000017188	*COA3*
11	ENSG00000110871	ENSMUSG00000041733	*COQ5*
12	ENSG00000119723	ENSMUSG00000021235	*COQ6*
13	ENSG00000167186	ENSMUSG00000030652	*COQ7*
14	ENSG00000088682	ENSMUSG00000031782	*COQ9*
15	ENSG00000111775	ENSMUSG00000041697	*COX6A1*
16	ENSG00000126267	ENSMUSG00000036751	*COX6B1*
17	ENSG00000164919	ENSMUSG00000014313	*COX6C*
18	ENSG00000112695	ENSMUSG00000032330	*COX7A2*
19	ENSG00000205560	ENSMUSG00000078937	*CPT1B*
20	ENSG00000157184	ENSMUSG00000028607	*CPT2*
21	ENSG00000095321	ENSMUSG00000026853	*CRAT*
22	ENSG00000088766	ENSMUSG00000027357	*CRLS1*
23	ENSG00000062485	ENSMUSG00000005683	*CS*
24	ENSG00000179091	ENSMUSG00000022551	*CYC1*
25	ENSG00000104325	ENSMUSG00000028223	*DECR1*
26	ENSG00000150768	ENSMUSG00000000168	*DLAT*
27	ENSG00000091140	ENSMUSG00000020664	*DLD*
28	ENSG00000119689	ENSMUSG00000004789	*DLST*
29	ENSG00000103423	ENSMUSG00000004069	*DNAJA3*
30	ENSG00000104823	ENSMUSG00000053898	*ECH1*
31	ENSG00000127884	ENSMUSG00000025465	*ECHS1*
32	ENSG00000115380	ENSMUSG00000020467	*EFEMP1*
33	ENSG00000136960	ENSMUSG00000022425	*ENPP2*
34	ENSG00000140374	ENSMUSG00000032314	*ETFA*
35	ENSG00000171503	ENSMUSG00000027809	*ETFDH*
36	ENSG00000180185	ENSMUSG00000045316	*FAHD1*
37	ENSG00000091483	ENSMUSG00000026526	*FH*
38	ENSG00000122642	ENSMUSG00000029781	*FKBP9*
39	ENSG00000160688	ENSMUSG00000042642	*FLAD1*
40	ENSG00000110074	ENSMUSG00000039048	*FOXRED1*
41	ENSG00000163430	ENSMUSG00000022816	*FSTL1*
42	ENSG00000168827	ENSMUSG00000027774	*GFM1*

43	ENSG00000165678	ENSMUSG00000041028	*GHITM*
44	ENSG00000198814	ENSMUSG00000025059	*GK*
45	ENSG00000115159	ENSMUSG00000026827	*GPD2*
46	ENSG00000084754	ENSMUSG00000025745	*HADHA*
47	ENSG00000138029	ENSMUSG00000059447	*HADHB*
48	ENSG00000166411	ENSMUSG00000032279	*IDH3A*
49	ENSG00000101365	ENSMUSG00000027406	*IDH3B*
50	ENSG00000067829	ENSMUSG00000002010	*IDH3G*
51	ENSG00000011376	ENSMUSG00000035202	*LARS2*
52	ENSG00000050426	ENSMUSG00000037353	*LETMD1*
53	ENSG00000138095	ENSMUSG00000024120	*LRPPRC*
54	ENSG00000146701	ENSMUSG00000019179	*MDH2*
55	ENSG00000151376	ENSMUSG00000030621	*ME3*
56	ENSG00000116353	ENSMUSG00000028910	*MECR*
57	ENSG00000103150	ENSMUSG00000074064	*MLYCD*
58	ENSG00000130312	ENSMUSG00000034880	*MRPL34*
59	ENSG00000135900	ENSMUSG00000026248	*MRPL44*
60	ENSG00000062582	ENSMUSG00000020477	*MRPS24*
61	ENSG00000198899	ENSMUSG00000064357	*MT-ATP6*
62	ENSG00000228253	ENSMUSG00000064356	*MT-ATP8*
63	ENSG00000109919	ENSMUSG00000027282	*MTCH2*
64	ENSG00000198712	ENSMUSG00000064354	*MT-CO2*
65	ENSG00000198938	ENSMUSG00000064358	*MT-CO3*
66	ENSG00000162576	ENSMUSG00000029070	*MXRA8*
67	ENSG00000105835	ENSMUSG00000020572	*NAMPT*
68	ENSG00000130414	ENSMUSG00000026260	*NDUFA10*
69	ENSG00000170906	ENSMUSG00000035674	*NDUFA3*
70	ENSG00000189043	ENSMUSG00000029632	*NDUFA4*
71	ENSG00000139180	ENSMUSG00000000399	*NDUFA9*
72	ENSG00000147123	ENSMUSG00000031059	*NDUFB11*
73	ENSG00000147684	ENSMUSG00000022354	*NDUFB9*
74	ENSG00000023228	ENSMUSG00000025968	*NDUFS1*
75	ENSG00000158864	ENSMUSG00000013593	*NDUFS2*
76	ENSG00000213619	ENSMUSG00000005510	*NDUFS3*
77	ENSG00000178127	ENSMUSG00000024099	*NDUFV2*
78	ENSG00000105953	ENSMUSG00000020456	*OGDH*
79	ENSG00000154814	ENSMUSG00000021906	*OXNAD1*
80	ENSG00000152782	ENSMUSG00000033610	*PANK1*
81	ENSG00000131828	ENSMUSG00000031299	*PDHA1*
82	ENSG00000168291	ENSMUSG00000021748	*PDHB*
83	ENSG00000005882	ENSMUSG00000038967	*PDK2*
84	ENSG00000167085	ENSMUSG00000038845	*PHB*
85	ENSG00000214456	ENSMUSG00000011305	*PLIN5*
86	ENSG00000105819	ENSMUSG00000029017	*PMPCB*
87	ENSG00000165672	ENSMUSG00000024997	*PRDX3*
88	ENSG00000042445	ENSMUSG00000056666	*RETSAT*

89	ENSG00000073578	ENSMUSG00000021577	*SDHA*
90	ENSG00000117118	ENSMUSG00000009863	*SDHB*
91	ENSG00000108528	ENSMUSG00000014606	*SLC25A11*
92	ENSG00000125454	ENSMUSG00000020744	*SLC25A19*
93	ENSG00000178537	ENSMUSG00000032602	*SLC25A20*
94	ENSG00000151729	ENSMUSG00000031633	*SLC25A4*
95	ENSG00000181035	ENSMUSG00000002346	*SLC25A42*
96	ENSG00000005022	ENSMUSG00000016319	*SLC25A5*
97	ENSG00000136143	ENSMUSG00000022110	*SUCLA2*
98	ENSG00000163541	ENSMUSG00000052738	*SUCLG1*
99	ENSG00000156502	ENSMUSG00000020079	*SUPV3L1*
100	ENSG00000165124	ENSMUSG00000028369	*SVEP1*
101	ENSG00000166575	ENSMUSG00000039428	*TMEM135*
102	ENSG00000178952	ENSMUSG00000073838	*TUFM*
103	ENSG00000184470	ENSMUSG00000075704	*TXNRD2*
104	ENSG00000109424	ENSMUSG00000031710	*UCP1*
105	ENSG00000101019	ENSMUSG00000005882	*UQCC1*
106	ENSG00000156467	ENSMUSG00000021520	*UQCRB*
107	ENSG00000010256	ENSMUSG00000025651	*UQCRC1*
108	ENSG00000140740	ENSMUSG00000030884	*UQCRC2*
109	ENSG00000169021	ENSMUSG00000038462	*UQCRFS1*
110	ENSG00000164405	ENSMUSG00000044894	*UQCRQ*

Supplementary Table 6. Sequence of siRNAs used in screening (single siRNA) and functional studies (siRNA pools).

Gene symbol	Sense siRNA sequence
ACAA2	GCUACUGACUUGUCUGAAU
ACADM	GCUCUGAUGUAGCUGGUAU
ACADVL	GCCUUUGCAACACCCAGUA
ACO2	GCCCAACGAGUACAUCCAU
ACSL5	GCUUGUUACACGUACUCUA
AFG3L2	GGAGAAACCUUACAGUGAA
AKAP1	CCAAGGACAGUAAGCCAAA
BOLA3	GUGACCCAAAUUCUCAAAG
CHCHD3	GCCACCCAGUAUAUGCACU
COA3	GCGUUUCCUAGAUGAGCUA
COQ5	GCAUUCCGGUUCCUUAAUU
COQ6	GGGACCAUAUCUGCAACAU
COQ7	GCAGUUCAGGAAUGACUUU
COQ9	GGGUUAAUGAUGCAAUGAA
COX6A1	CCAUAACCCUCAUGUGAAU
COX6B1	CCGCUAAAGGAGGCGAUAU
COX6C	GCUGUAGCAUUCGUGCUAU
COX7A2	GCUGGCUCUUCGUCAGAUU
CPT1B	CCAAAGAAUUCCAGGACAA
CPT2	GCAAAGAAUUCCUGAAGAA
CRAT	CCUUCAUCCAGAUGGCUUU
CRLS1	GGACAAUCCCGAAUAUGUU
CS	GGUCUCACAAUUUCACCAA
CYC1	CCAUCUACACAGAUGUCUU
DECR1	GGAAAUAAGGUUCAUGCAA
DLAT	GCAGAGGUUGAAACUGAUA
DLD	CCAAGAGAGGCCAUUUCAA
DLST	CCCUAACAGCAGGAAGGUU
DNAJA3	CCCUGACACAAAUAAGGAU
ECH1	GGUACCUCCGUGACAUCAU
ECHS1	GCCUGAUUGACGAGCUCAA
EFEMP1	CCACCAAAGAUGCGUGAAU
ENPP2	CCAAUUAUCCAGGGAUUAU
ETFA	GGAAAUAUCAGAGUGGCUU
ETFDH	CCACUUAACACUCCUGUAA
FAHD1	CCUUGGAAGAAGGAGAUAU
FH	GGUGCCAAAUGAUAAGUAU
FKBP9	GCAUUAUUGGACCUCCAUA
FLAD1	CCACUCAAAGGAGCUAUAU
FOXRED1	CCAGCUUUCUACGGAACAU
FSTL1	GCAGUAAUGGCAAGACCUA
GFM1	GGAGCUAAUUGAAUGUGUU

GHITM	GCAGGGAGUAUUGGUUUAA
GK	CUUCGUUGGCUCCUUGACA
GPD2	GCCUAUAAUGCUUCCAGUU
HADHA	GCUGACUGGUAGAAGCAUU
HADHB	GGUGUUGGCUUGAUUGCUU
IDH3A	GGUGGUGUUCAGACAGUAA
IDH3B	GCUGCGGCAUCUUAAUCUU
IDH3G	CCAACGUCAUCCACUGUAA
LARS2	GCUUUCCAUGUUCCCUUAU
LETMD1	CGUUUGAAGACUCAUACAA
LRPPRC	GGAGGAAGCAAACAUUCAA
MDH2	GGAGAAGAUGAUCUCGGAU
ME3	CCUCCGAAUCAUGAGAUAU
MECR	GGAAGCACUGAUCCAAGUU
MLYCD	CAGAAACAGAAGAGAAGAA
MRPL34	CCUGAGUGCUCUCCAUAUU
MRPL44	GCUGUUCUUCUGAAUCUUA
MRPS24	GCCCUCAAAGGUUGUGUAU
MT-ATP6	CCUACUCAUGCACCUAAUU
MT-ATP8	CCGUAUGGCCCACCAUAAU
MTCH2	GCUCAUCCAGGUGGGAUAU
MT-CO2	GCAAUUCCCGGACGUCUAA
MT-CO3	GCUCAACUUUCCUCACUAU
MXRA8	CCCGAAUCCUGCUUUGGAA
NAMPT	GGGCCGAUUAUCUUUACAU
NDUFA10	GCGCGUGAGAGGAAUUCAU
NDUFA3	UCCGUGAUGAUGGGAACAU
NDUFA4	CCAAGAAGCAUCCGAGCUU
NDUFA9	CCGGAAGCCAUUAUCGUAA
NDUFB11	GCUGUUUGGUUUGAGCGCU
NDUFB9	GCUUUAUAAGCGGGCGCUA
NDUFS1	GCAAGUCAAGUAGCUGCUU
NDUFS2	GGAAGUGUGAUCCUCACAU
NDUFS3	GCCCAAGUAUGUCCAACAA
NDUFV2	CCAUCUCUGCUAUGAACAA
OGDH	GGAACAGAUCUUCUGUCAA
OXNAD1	UAGAAUUGGCAGUGAAAUA
PANK1	CCAUGAAGCUGCUGGCAUA
PDHA1	GCUUACAACAUGGCAGCUU
PDHB	CCAUUAGACCAAUGGACAU
PDK2	GCUCCUGUGUGACAAGUAU
PHB	GCUGAGCUGAUUGCCAACU
PLIN5	CCCUUUCUCCAGCAACCUU
PMPCB	GCACACUUUCUGGAGCAUA
PRDX3	GGUUCUGGUCUUGCACUAA
RETSAT	CCAGGAAGAAGGUUCUCAA

SDHA	CUGAAGAUGGGAAGAUUUA
SDHB	GCUGGAGACAAACCUCAUA
SLC25A11	GGACGUGCUGUUCAAAGUU
SLC25A19	GCUAUGGAGCUGUCCAAUU
SLC25A20	GCGUAUUCACCACAGGAAU
SLC25A4	GCAGUUCUGGCGCUACUUU
SLC25A42	CCUCGUCCGUCUCAUCAAA
SLC25A5	UCGUCAUCAGCUGGAUGAU
SUCLA2	CCAGAUGAAGCUUAUGCAA
SUCLG1	GCAACGGCUUCUGUCAUUU
SUPV3L1	GCGGAUGAUUUAUUCCCAU
SVEP1	GCACAUGUGUGAAAGGAUU
TMEM135	CCUACAAUCCGCUUCAUUU
TUFM	GCGGCUCAUGUGGAGUAUA
TXNRD2	CCAUCAUGAUGCGCAGCAU
UCP1	GGUCGUGAAAGUCAGACUU
UQCC1	GCCUGUUGAGGAGAAGGUU
UQCRB	GCAAGUGGCUGGAUGGUAU
UQCRC1	GGUCUUUAACUACCUGCAU
UQCRC2	CCAAGUUACCAAAUGGCUU
UQCRFS1	CCCACAGCAUGAUCUAGAU
UQCRQ	UCGAGAGAUCCAAGAGGAA
Non-targeting control siRNA	#1 UGGUUUACAUGUCGACUAA<br>#2 UGGUUUACAUGUUUUCUGA<br>#3 UGGUUUACAUGUUUUCCUA
MTCH2	#1 GCUCAUCCAGGUGGGAUAU<br>#2 GUGCUUGUCUCCAAUCUUA<br>#3 GUUAGGACCUGGAAAUGUA
Mtch2	#1 UAAGUACUGUGGACUGUGU<br>#2 GGAAGAAGGCAUCGUAGGA<br>#3 GGAAATATGAGCCGAGGAA

Supplementary Table 7. Lists of primer sequences used in this study.

Gene symbol	Primer sequence
MTCH2	FWD: TCCGGTCTCACCATCCTG<br>REV: GAAGAGGCTCATATCCCACCT
UCP1	FWD: AGGTCCAAGGTGAATGCCC<br>REV: GCGGTGATTGTTCCCAGGA
CIDEA	FWD: GGCAGGTTCACGTGTGGATA<br>REV: GAAACACAGTGTTTGGCTCAAGA
COX7A1	FWD: TGACATCCCGTTGTACCTGAAG<br>REV: ACAGTGCCGCCCAGACA
TBP	FWD: CACGAACCACGGCACTGATT<br>REV: TTTTCTTGCTGCCAGTCTGGAC
RPL13A	FWD: GGACCGTGCGAGGTATGCT<br>REV: ATGCCGTCAAACACCTTGAG A
Mtch2	FWD: GCTCATGTACGTGAAAGTGCT<br>REV: TGACATACTTGTCGCCCAAA
Ucp1	FWD: GGGCATTCAGAGGCAAATCAGCTT<br>REV: ACACTGCCACACCTCCAGTCATTA
Tbp	FWD: GAAGCTGCGGTACAATTCCAG<br>REV: CCCCTTGTACCCTTCACCAAT
FABP4	FWD: CCACCATAAAGAGAAAACGAGAG<br>REV: GTGGAAGTGACGCCTTTCAT
ADIPOQ	FWD: GGTGAGAAGGGTGAGAAAGGA<br>REV: TTTCACCGATGTCTCCCTTAG
PPARG	FWD: CGTGGCCGCAGAAATGA<br>REV: TCAAAGGAGTGGGAGTGGTC
PPARGC1A	FWD: CAGCCTCTTTGCCCAGATCTT<br>REV: TCACTGCACCACTTGAGTCCAC
