# Supplementary material for: MTCH2 modulates CPT1 activity to regulate lipid metabolism of adipocytes
Source: Nat Commun. 2025 Oct 3;16:8831. doi: 10.1038/s41467-025-63880-7 (PMC12494973; doi:10.1038/s41467-025-63880-7)
Supplement: Supplementary file 2 — Reporting summary [file 41467_2025_63880_MOESM2_ESM.pdf]

nature portfolio

Corresponding author(s): Christian Wolfrum

Last updated by author(s): Aug 5, 2025

Reporting Summary

Nature Portfolio wishes to improve the reproducibility of the work that we publish. This form provides structure for consistency and transparency in reporting. For further information on Nature Portfolio policies, see our Editorial Policies and the Editorial Policy Checklist.

Statistics

For all statistical analyses, confirm that the following items are present in the figure legend, table legend, main text, or Methods section.

n/a	Confirmed	
☐	☒	The exact sample size (*n*) for each experimental group/condition, given as a discrete number and unit of measurement
☐	☒	A statement on whether measurements were taken from distinct samples or whether the same sample was measured repeatedly
☐	☒	The statistical test(s) used AND whether they are one- or two-sided<br>*Only common tests should be described solely by name; describe more complex techniques in the Methods section.*
☒	☐	A description of all covariates tested
☐	☒	A description of any assumptions or corrections, such as tests of normality and adjustment for multiple comparisons
☐	☒	A full description of the statistical parameters including central tendency (e.g. means) or other basic estimates (e.g. regression coefficient) AND variation (e.g. standard deviation) or associated estimates of uncertainty (e.g. confidence intervals)
☐	☒	For null hypothesis testing, the test statistic (e.g. *F*, *t*, *r*) with confidence intervals, effect sizes, degrees of freedom and *P* value noted<br>*Give P values as exact values whenever suitable.*
☒	☐	For Bayesian analysis, information on the choice of priors and Markov chain Monte Carlo settings
☒	☐	For hierarchical and complex designs, identification of the appropriate level for tests and full reporting of outcomes
☐	☒	Estimates of effect sizes (e.g. Cohen's *d*, Pearson's *r*), indicating how they were calculated

Our web collection on statistics for biologists contains articles on many of the points above.

Software and code

Policy information about availability of computer code

Data collection	Western blots were visualized by the Image Quant system (GE Healthcare Life Sciences) and ChemiDoc MP Imaging System (Bio-Rad). Quantitative PCR was performed using ViiA7 realtime PCR system (Applied Biosystems). Biochemical colorimetric assays were detected by SynergyMx plate reader (BioTek). Cellular respiration was monitored by XF96 Extracellular Flux Analyzer (Agilent Seahorse). Indirect calorimetry measurement was performed by Promethion Core system(Sable systems). Live mice body composition was measured with a magnetic resonance imaging technique (EchoMRI130, Echo Medical Systems). Electron Microscopy images were taken in a TFS Magellan 400i FE-SEM by back scatter electron detection at 1.8 kV and 0.8 nA. ^{1}H-NMR spectra were acquired using a Bruker AVANCE II 600 MHz spectrometer equipped with a 5 mm TCI cryoprobe, employing the noesygppr1d pulse sequence (TopSpin 3.5, Bruker).Comprehensive targeted lipidomics was accomplished using a flow-injection assay based on lipid class separation by differential mobility spectroscopy (DMS) and selective multiple reaction monitoring (MRM) per lipid species (Lipidyzer platform), employing a Shimadzu Nexera series HPLC and a Sciex QTrap 5500 mass spectrometer. Mass spectrometry analysis of peptides was performed on an Orbitrap Fusion Lumos (Thermo Scientific) equipped with a Digital PicoView source (New Objective) and coupled to an M-Class UPLC (Waters). The mass spectrometer was operated in data-independent mode (DIA), acquiring a full-scan MS (396–1000 m/z) at a resolution of 60000 at 200 m/z.
Data analysis	Image Lab 6 and imageJ 1 were used to analyze western blots and images. Cell respiratory analysis was done by Wave 2.6.0. (Agilent Seahorse). Real-time PCR was analyzed by ViiA7 Ruo v1.2.3 (ThermoFisher). Energy expenditure ANCOVA analysis was performed by R version 4.5.0 and RStudio 2024. Proteomics data was done by DIA-NN (Version 16). FastQC program (Babraham Bioinformatics, Babraham Institute). DESeq2 package for differential gene expression analysis. Metabolites were quantified using the Chenomx NMR Suite (Chenomx Inc.)), with the TSP peak at 0.00 ppm serving as the chemical shift and concentration reference. The absolute concentration of TSP was calibrated using the ERETIC2 method (TopSpin 3.5, Bruker). Acquired lipid data were processed using the Shotgun Lipidomics Assistant (SLA) software. The acquired Ppeptide data independent acquisition spectra were processed with DIA-NN (Version 16) using a library free approach. Graphpad

prism 10 was used to perform statistical analyses. The R package prolfqua [doi: 10.1021/acs.jproteome.2c00441] was used to analyze the differential expression and to determine group differences, confidence intervals, and false discovery rates for all quantifiable proteins.

For manuscripts utilizing custom algorithms or software that are central to the research but not yet described in published literature, software must be made available to editors and reviewers. We strongly encourage code deposition in a community repository (e.g. GitHub). See the Nature Portfolio guidelines for submitting code & software for further information.

Data

Policy information about availability of data

All manuscripts must include a data availability statement. This statement should provide the following information, where applicable:

- Accession codes, unique identifiers, or web links for publicly available datasets
- A description of any restrictions on data availability
- For clinical datasets or third party data, please ensure that the statement adheres to our policy

The RNA-seq data used in this study for the human BAT/WAT and pure murine adipocytes transcriptome are available in the European Nucleotide Archive under accession code PRJEB20634. The hMADS RNA-seq data used in this study are available in the European Nucleotide Archive under accession code PRJEB38756. The Proteomics data generated in this study have been deposited in ProteomeXchange under accession code PXD054881. Metabolomics and lipidomics data generated in this study have been deposited in Mendeley Data under accession codes (https://data.mendeley.com/datasets/cmhzgdr4fb/1). The human RNA-seq data for the LOBB cohort and the ACTIBATE cohort reported in this study data are available under restricted access due to patient consent agreements, access can be obtained from the lead contact upon request. All other data generated in this study are provided in the Supplementary Information/Source Data file. Source data are provided with this paper.

Research involving human participants, their data, or biological material

Policy information about studies with human participants or human data. See also policy information about sex, gender (identity/presentation), and sexual orientation and race, ethnicity and racism.

Reporting on sex and gender	Sex and/or gender of participants was determined based on self-report. No sex- and gender-based analyses have been performed.
Reporting on race, ethnicity, or other socially relevant groupings	n.a.
Population characteristics	All subjects for clinical study for transcriptome (age 40.2 ± 9.4 years, BMI 22.8 ± 2.2 kg/m2, 20 females and 7 males).The LOBB CSC comprises 1479 individuals categorized as either normal/overweight (n= 31; 52% women; age: 55.8 ± 13.4 years old; BMI: 25.7 ± 2.7 kg/m2) or obese (n = 1448; 71% women; age: 46.9 ± 11.7 years old; BMI: 49.2 ± 8.3 kg/m2). The LOBB MHUO comprises 29 individuals classified as insulin sensitive (IS; 80% female; age: 46.0 ± 7.1 years old; BMI: 46.0 ± 7.1 kg/m2 ; FPG: 5.2 ±0.2 mmol/L; FPI: 27.6 ± 13.8 pmol/L) and 39 individuals classified as insulin resistant (IR; 74.4% female; age: 47 ± 7.6 years old; BMI: 46.6 ± 7.1 kg/m2; FPG: 5.7 ± 0.3 mmol/L; FPI: 106.4 ± 31.6 pmol/L). ACTIBATE study: 60 individuals (80% women; age: 22.1 ± 2.1 years old; BMI: 25.5 ± 4.4 kg/m2).
Recruitment	The participants recruitment for clinical transcriptome study were screened for medical history and status, and only healthy volunteers were enrolled in the study, more detailed information provided in Orava et al., 2011 Cell Metab. The participants recruitment for the LOBB CSC and MUO/MHO study: participants were recruited between 2008 and 2018 during elective laparoscopic abdominal surgery. Tissue samples were taken from adult men and women aged 18 and older who underwent elective abdominal surgery, and who consented to study participation. ACTIBATE study: a total of 145 young sedentary adults aged 18–25 years old participated in the study (ClinicalTrials.gov ID: NCT02365129). Eligible participants were randomly assigned into three groups: a non-exercise group or one of two exercise groups (moderate-intensity exercise and vigorous-intensity exercise). More detailed information is provided in Sanchez-Delgado, G. et al., 2015 Contemp. Clin. Trials.
Ethics oversight	The clinical study for transcriptome was approved by the Ethics Committee of the Hospital District of Southwest Finland and conducted according to the principles of the Declaration of Helsinki. Approval for LOBB studies was obtained from the Ethics Committee of the University of Leipzig (approval no: 159-12-21052012) before the study, and acquisition was performed in accordance with the Declaration of Helsinki. The ACTIBATE study received approval from the Ethics Committee on Human Research of the University of Granada (no. 924) and the Servicio Andaluz de Salud (Centro de Granada, CEI-Granada, Spain).

Note that full information on the approval of the study protocol must also be provided in the manuscript.

Field-specific reporting

Please select the one below that is the best fit for your research. If you are not sure, read the appropriate sections before making your selection.

☒ Life sciences ☐ Behavioural & social sciences ☐ Ecological, evolutionary & environmental sciences

For a reference copy of the document with all sections, see nature.com/documents/nr-reporting-summary-flat.pdf

Life sciences study design

All studies must disclose on these points even when the disclosure is negative.

Sample size	All samples sizes used and analyzed are described in each Figure (legends) and/or in the corresponding methods part. Sample sizes were determined on the basis of previous experiments using similar methods.
Data exclusions	No samples were excluded from any analysis.
Replication	Except Omics analyses (metabolomics, lipidomics and proteomics), all cell culture experiments and animal experiments were repeated independently 2 or 3 times, with the exact biological replicates indicated in the figure legends.
Randomization	All experiments were randomized.
Blinding	The investigators were not blinded to the mice as they themselves were treating and sacrificing the mice. However, the investigators were blinded for sample processing.

Reporting for specific materials, systems and methods

We require information from authors about some types of materials, experimental systems and methods used in many studies. Here, indicate whether each material, system or method listed is relevant to your study. If you are not sure if a list item applies to your research, read the appropriate section before selecting a response.

Materials & experimental systems

n/a	Involved in the study	
☐	☒	Antibodies
☐	☒	Eukaryotic cell lines
☒	☐	Palaeontology and archaeology
☐	☒	Animals and other organisms
☒	☐	Clinical data
☒	☐	Dual use research of concern
☒	☐	Plants

Methods

n/a	Involved in the study	
☒	☐	ChIP-seq
☒	☐	Flow cytometry
☒	☐	MRI-based neuroimaging

Antibodies

Antibodies used

primary antibodies:
MTCH2(1:1000, Invitrogen: PA5-88873), phosphor-HSL(Ser660) (1:1000, Cell Signaling:45804S), HSL (1:1000, Cell Signaling:4107S), γ-tubulin (1:10000, Sigma:T6557), FLAG (1:1000, Cell Signaling:2368S), HSP90 (1:1000, Cell Signaling:4877S), UCP1 (1:1000, ThermoFisher: PA1-24894), OXPHOS (1:1000, Abcam: ab110413), CPT1a (1:1000, Proteintech: 66039-1), CPT1a (1:1000, Cell Signaling: 12252S), CPT1b (1:1000, Invitrogen: CF810597), CPT1b (1:1000, Cell Signaling: 41803S),phospho-DRP1 (Ser616)(1:1000, Thermo Fisher: PA5-64821), phospho-DRP1 (Ser637)(1:1000, Thermo Fisher: PA5-37534), DRP1 (1:1000, Cell Signaling: 8570), OPA1 (1:1000, Cell Signaling: 80471), MFN1 (1:1000, Proteintech: 13798-1-AP), MFN2 (1:1000, Proteintech: 12186-1-AP), VDAC (1:1000, Proteintech:55259-1-AP), p70S6 kinase (1:1000, Cell Signaling:9202S)
Secondary antibodies:
HRP-conjugated anti-rabbit IgG (1:10000, Cell signaling: 7074), HRP-conjugated anti-mouse IgG (1:10000, Cell signaling:7076).

Validation

- Validation MTCH2 (PA5-88873): https://www.thermofisher.com/antibody/product/MTCH2-Antibody-Polyclonal/PA5-88873 and Figure 3b,d,e in our manuscript.
- Validation phosphor-HSL(Ser660)(45804S): https://doi.org/10.1038/s42255-021-00489-2, https://www.cellsignal.com/products/primary-antibodies/phospho-hsl-ser660-antibody/45804?
- Validation HSL(4107S): https://doi.org/10.1038/s42255-021-00489-2, https://www.cellsignal.com/products/primary-antibodies/hsl-antibody/4107?
- Validation γ-tubulin (T6557): https://www.sigmaaldrich.com/CH/en/product/sigma/t6557?
- Validation FLAG (2368S): https://doi.org/10.1038/s42255-021-00489-2, and Figure 6i in our manuscript.
- Validation HSP90 (4877S): https://www.cellsignal.com/products/primary-antibodies/hsp90-c45g5-rabbit-mab/4877?
- Validation UCP1 (PA1-24894): https://www.thermofisher.com/antibody/product/UCP1-Antibody-Polyclonal/PA1-24894?
- Validation OXPHOS (ab110413): https://www.abcam.com/en-us/products/panels/total-oxphos-rodent-wb-antibody-cocktail-ab110413?
- Validation CPT1a (66039-1): https://www.ptglab.com/products/CPT1A-Antibody-66039-1-Ig.htm?
- Validation CPT1a (12252S): https://www.cellsignal.com/products/primary-antibodies/cpt1a-d3b3-rabbit-mab/12252?
- Validation CPT1b (CF810597): https://www.thermofisher.com/antibody/product/CPT1B-Antibody-clone-OTI2A6-Monoclonal/CF810597
- Validation CPT1b (41803S): https://www.cellsignal.com/products/primary-antibodies/cpt1b-e6m5m-rabbit-mab/41803?
- Validation phospho-DRP1 (Ser616)(PA5-64821): https://www.thermofisher.com/antibody/product/Phospho-DRP1-Ser616-Antibody-Polyclonal/PA5-64821
- Validation phospho-DRP1 (Ser637)(PA5-37534): https://www.thermofisher.com/antibody/product/Phospho-DRP1-Ser637-Antibody-Polyclonal/PA5-37534

- Validation DRP1 (8570): https://www.cellsignal.com/products/primary-antibodies/drp1-d6c7-rabbit-mab/8570?
- Validation OPA1 (80471): https://www.cellsignal.com/products/primary-antibodies/opa1-d6u6n-rabbit-mab/80471?
- Validation MFN1 (13798-1-AP): https://www.ptglab.com/products/MFN1-Antibody-13798-1-AP.htm?
- Validation MFN2 (12186-1-AP): https://www.ptglab.com/products/MFN2-Antibody-12186-1-AP.htm?
- Validation VDAC (55259-1-AP): https://www.ptglab.com/products/VDAC1-Antibody-55259-1-AP.htm?
- Validation p70S6 kinase (9202S): https://www.cellsignal.com/products/primary-antibodies/p70-s6-kinase-antibody/9202?

Eukaryotic cell lines

Policy information about cell lines and Sex and Gender in Research

Cell line source(s)	hMADS cells derived from the prepubic fat pad of a 4-month-old male were kindly provided by Dr. Ez-Zoubir Amri, University of Nice, France. Murine immortalized brown adipocytes (iBAs) were provided by prof. Klein, which were derived from the iBAT stromal-vascular fraction of late fetal and newborn C57Bl/6 mice (both genders). HEK293-LTV cell lines were purchased from Cell Biolabs Inc. (LTV-100); HEK293T cell line was purchased from abcam (ab255449). Mouse primary brown adipocytes were isolated from iBAT depots of male Mtch2fl/fl and of Mtch2fl/flAdip-CreERT2 (6 week old) mice.
Authentication	hMADS and iBA cell lines are commonly used to study adipocyte physiology and function, and were widely tested in our previous studies and by other groups. These cell lines were authenicated by PCR assay using species-specific primers to determine expression level of key thermogenic protein UCP1. HEK293T cell line is widely used in cell biology research. HEK293LTV cell line is derived from HEK293T and used for higher lentiviral yields. None of HEK293 cell lines was authenticated.
Mycoplasma contamination	All cell lines were regularly tested negative for mycoplasma contamination.
Commonly misidentified lines (See ICLAC register)	No commonly misidentified cell lines were used in our study.

Animals and other research organisms

Policy information about studies involving animals; ARRIVE guidelines recommended for reporting animal research, and Sex and Gender in Research

Laboratory animals	The MTCH2fl/fl mouse strain was kindly provided by Prof. Atan Gross Lab (Weizmann Institute of Science, Israel) in 129/SVJ l background. Adip-CreERT2 mice were generated by Dr. Evan Rosen lab (Beth Israel Deaconess Medical Center and Harvard Medical School) in C57BL/6J background. Inducible adipose tissue-specific ablation of MTCH2 was achieved by crossing MTCH2fl/fl mice with Adip-CreERT2 mice. For primary brown adipocytes isolation, 6 weeks old male mice were used. For other animal experiments, 7-9 weeks male mice were orally administered tamoxifen (2 mg/mouse in sunflower oil, Sigma–Aldrich) to induce recombination of the floxed allele for further study on chow diet or HFD. The exact numbers for mice experiments were indicated in each figure legend.
Wild animals	No wild animals were used in the study.
Reporting on sex	All male mice were used in the study.
Field-collected samples	No field-collected samples were used in the study.
Ethics oversight	All animal experiments in our study were approved by the Veterinary Office of the Canton of Zürich, Switzerland (ZH114/2024).

Note that full information on the approval of the study protocol must also be provided in the manuscript.

Plants

Seed stocks	n.a.
Novel plant genotypes	n.a.
Authentication	n.a.
